# Supplementary material for: Mangrove Tirucallane- and Apotirucallane-Type Triterpenoids: Structure Diversity of the C-17 Side-Chain and Natural Agonists of Human Farnesoid/Pregnane–X–Receptor
Source: Mar Drugs. 2018 Dec 6;16(12):488. doi: 10.3390/md16120488 (PMC6315889; doi:10.3390/md16120488)

Supporting Information

Copies of HR-ESIMS of **1–10**, **4s**/**r**, **6s**/**r**, and **7s**/**r**; 1D and 2D NMR spectra of **1–10**; and ^{1}H NMR spectra of **4s**/**r**, **6s**/**r**, and **7s**/**r**.

Table of Contents:

1. HR-ESIMS for compound **1** ······ S6-S7
2. ^{1}H NMR (400 MHz) spectrum of compound **1** in CDCl3 ······ S8-S10
3. ^{13}C NMR (100 MHz) spectrum of compound **1** in CDCl3 ······ S11-S13
4. DEPT135 (100 MHz) spectrum of compound **1** in CDCl3 ······ S14-S15
5. ^{1}H-^{1}H COSY (400 MHz) spectrum of compound **1** in CDCl3 ······ S16-S18
6. HSQC (400 MHz) spectrum of compound **1** in CDCl3 ······ S19-S21
7. HMBC (400 MHz) spectrum of compound **1** in CDCl3 ······ S22-S27
8. NOESY (400 MHz) spectrum of compound **1** in CDCl3 ······ S28-S32
9. HR-ESIMS for compound **2** ······ S33
10. ^{1}H NMR (400 MHz) spectrum of compound **2** in CDCl3 ······ S34-S36
11. ^{13}C NMR (100 MHz) spectrum of compound **2** in CDCl3 ······ S37-S39
12. DEPT135 (100 MHz) spectrum of compound **2** in CDCl3 ······ S40-S41
13. ^{1}H-^{1}H COSY (400 MHz) spectrum of compound **2** in CDCl3 ······ S42-S44
14. HSQC (400 MHz) spectrum of compound **2** in CDCl3 ······ S45-S46
15. HMBC (400 MHz) spectrum of compound **2** in CDCl3 ······ S47-S52
16. NOESY (400 MHz) spectrum of compound **2** in CDCl3 ······ S53-S58
17. HR-ESIMS for compound **3** ······ S59
18. ^{1}H NMR (400 MHz) spectrum of compound **3** in CDCl3 ······ S60-S62
19. ^{13}C NMR (100 MHz) spectrum of compound **3** in CDCl3 ······ S63-S64
20. DEPT135 (100 MHz) spectrum of compound **3** in CDCl3 ······ S65-S66
21. ^{1}H-^{1}H COSY (400 MHz) spectrum of compound **3** in CDCl3 ······ S67-S69
22. HSQC (400 MHz) spectrum of compound **3** in CDCl3 ······ S70-S71
23. HMBC (400 MHz) spectrum of compound **3** in CDCl3 ······ S72-S77
24. NOESY (400 MHz) spectrum of compound **3** in CDCl3 ······ S78-S82

Table of Contents:

25. HR-ESIMS for compound **4** ···· S83-S84
26. ^{1}H NMR (400 MHz) spectrum of compound **4** in CDCl3 ···· S85-S87
27. ^{13}C NMR (100 MHz) spectrum of compound **4** in CDCl3 ···· S88-S90
28. DEPT135 (100 MHz) spectrum of compound **4** in CDCl3 ···· S91-S94
29. ^{1}H-^{1}H COSY (400 MHz) spectrum of compound **4** in CDCl3 ···· S95-S97
30. HSQC (400 MHz) spectrum of compound **4** in CDCl3 ···· S98-S100
31. HMBC (400 MHz) spectrum of compound **4** in CDCl3 ···· S101-S106
32. NOESY (400 MHz) spectrum of compound **4** in CDCl3 ···· S107-S110
33. HR-ESIMS for compound **5** ···· S111-S112
34. ^{1}H NMR (400 MHz) spectrum of compound **5** in CDCl3 ···· S113-S115
35. ^{13}C NMR (100 MHz) spectrum of compound **5** in CDCl3 ···· S116-S118
36. DEPT135 (100 MHz) spectrum of compound **5** in CDCl3 ···· S119-S121
37. ^{1}H-^{1}H COSY (400 MHz) spectrum of compound **5** in CDCl3 ···· S122-S124
38. HSQC (400 MHz) spectrum of compound **5** in CDCl3 ···· S125-S127
39. HMBC (400 MHz) spectrum of compound **5** in CDCl3 ···· S128-S133
40. NOESY (400 MHz) spectrum of compound **5** in CDCl3 ···· S134-S137
41. HR-ESIMS for compound **6** ···· S138-S139
42. ^{1}H NMR (400 MHz) spectrum of compound **6** in CDCl3 ···· S140-S142
43. ^{13}C NMR (100 MHz) spectrum of compound **6** in CDCl3 ···· S143-S146
44. DEPT135 (100 MHz) spectrum of compound **6** in CDCl3 ···· S147-S148
45. ^{1}H-^{1}H COSY (400 MHz) spectrum of compound **6** in CDCl3 ···· S149-S152
46. HSQC (400 MHz) spectrum of compound **6** in CDCl3 ···· S153-S156
47. HMBC (400 MHz) spectrum of compound **6** in CDCl3 ···· S157-S164
48. NOESY (400 MHz) spectrum of compound **6** in CDCl3 ···· S165-S168

Table of Contents:

49. HR-ESIMS for compound **7** ······ S169-S170
50. ^{1}H NMR (400 MHz) spectrum of compound **7** in CDCl3 ······ S171-S173
51. ^{13}C NMR (100 MHz) spectrum of compound **7** in CDCl3 ······ S174-S175
52. DEPT135 (100 MHz) spectrum of compound **7** in CDCl3 ······ S176-S177
53. ^{1}H-^{1}H COSY (400 MHz) spectrum of compound **7** in CDCl3 ······ S178-S180
54. HSQC (400 MHz) spectrum of compound **7** in CDCl3 ······ S181-S183
55. HMBC (400 MHz) spectrum of compound **7** in CDCl3 ······ S184-S189
56. NOESY (400 MHz) spectrum of compound **7** in CDCl3 ······ S190-S193
57. HR-ESIMS for compound **8** ······ S194
58. ^{1}H NMR (400 MHz) spectrum of compound **8** in CDCl3 ······ S195-S197
59. ^{13}C NMR (100 MHz) spectrum of compound **8** in CDCl3 ······ S198-S200
60. DEPT135 (100 MHz) spectrum of compound **8** in CDCl3 ······ S201-S202
61. ^{1}H-^{1}H COSY (400 MHz) spectrum of compound **8** in CDCl3 ······ S203-S206
62. HSQC (400 MHz) spectrum of compound **8** in CDCl3 ······ S207-S210
63. HMBC (400 MHz) spectrum of compound **8** in CDCl3 ······ S211-S217
64. NOESY (400 MHz) spectrum of compound **8** in CDCl3 ······ S218-S222
65. HR-ESIMS for compound **9** ······ S223-S224
66. ^{1}H NMR (400 MHz) spectrum of compound **9** in CDCl3 ······ S225-S227
67. ^{13}C NMR (100 MHz) spectrum of compound **9** in CDCl3 ······ S228-S231
68. DEPT135 (100 MHz) spectrum of compound **9** in CDCl3 ······ S232-S233
69. ^{1}H-^{1}H COSY (400 MHz) spectrum of compound **9** in CDCl3 ······ S234-S237
70. HSQC (400 MHz) spectrum of compound **9** in CDCl3 ······ S238-S240
71. HMBC (400 MHz) spectrum of compound **9** in CDCl3 ······ S241-S246
72. NOESY (400 MHz) spectrum of compound **9** in CDCl3 ······ S247-S250

Table of Contents:

73. HR-ESIMS for compound **10** ······S251
74. ^{1}H NMR (400 MHz) spectrum of compound **10** in CDCl3······S252-S254
75. ^{13}C NMR (100 MHz) spectrum of compound **10** in CDCl3······S245-S258
76. DEPT135 (100 MHz) spectrum of compound **10** in CDCl3 ······S259-S261
77. ^{1}H-^{1}H COSY (400 MHz) spectrum of compound **10** in CDCl3 ······S262-S264
78. HSQC (400 MHz) spectrum of compound **10** in CDCl3 ······S265-S267
79. HMBC (400 MHz) spectrum of compound **10** in CDCl3 ······S268-S274
80. NOESY (400 MHz) spectrum of compound **10** in CDCl3 ······S275-S279
81. HR-ESIMS for compound **4s** ······S280-S281
82. ^{1}H NMR (400 MHz) spectrum of compound **4s** in CDCl3······S282-S284
83. HR-ESIMS for compound **4r** ······S285-S286
84. ^{1}H NMR (400 MHz) spectrum of compound **4r** in CDCl3······S287-S289
85. HR-ESIMS for compound **6s** ······S290
86. ^{1}H NMR (400 MHz) spectrum of compound **6s** in CDCl3······S291-S293
87. HR-ESIMS for compound **6r** ······S294
88. ^{1}H NMR (400 MHz) spectrum of compound **6r** in CDCl3······S295-S297
89. HR-ESIMS for compound **7s** ······S298
90. ^{1}H NMR (400 MHz) spectrum of compound **7s** in CDCl3······S299-S2301
91. HR-ESIMS for compound **7r** ······S302
92. ^{1}H NMR (400 MHz) spectrum of compound **7r** in CDCl3······S303-S305

HR-ESIMS for compound **1**

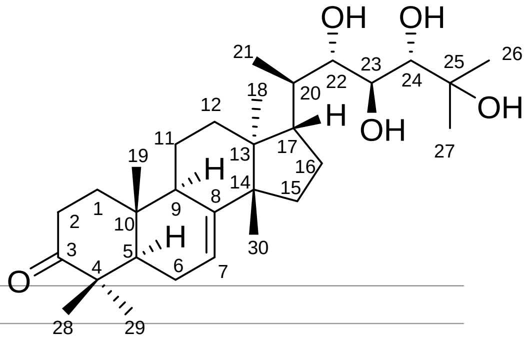

Generic Display Report

Analysis Info

Analysis Name	D:\Data\MS\data\201804\jiangzhongping_XG-52-3_neg_50_01_4495.d	Acquisition Date	4/3/2018 10:52:52 AM
Method	LC_Direct Infusion_neg_100-1000mz.m	Operator	SCSIO
Sample Name	jiangzhongping_XG-52-3_neg	Instrument	maXis
Comment			

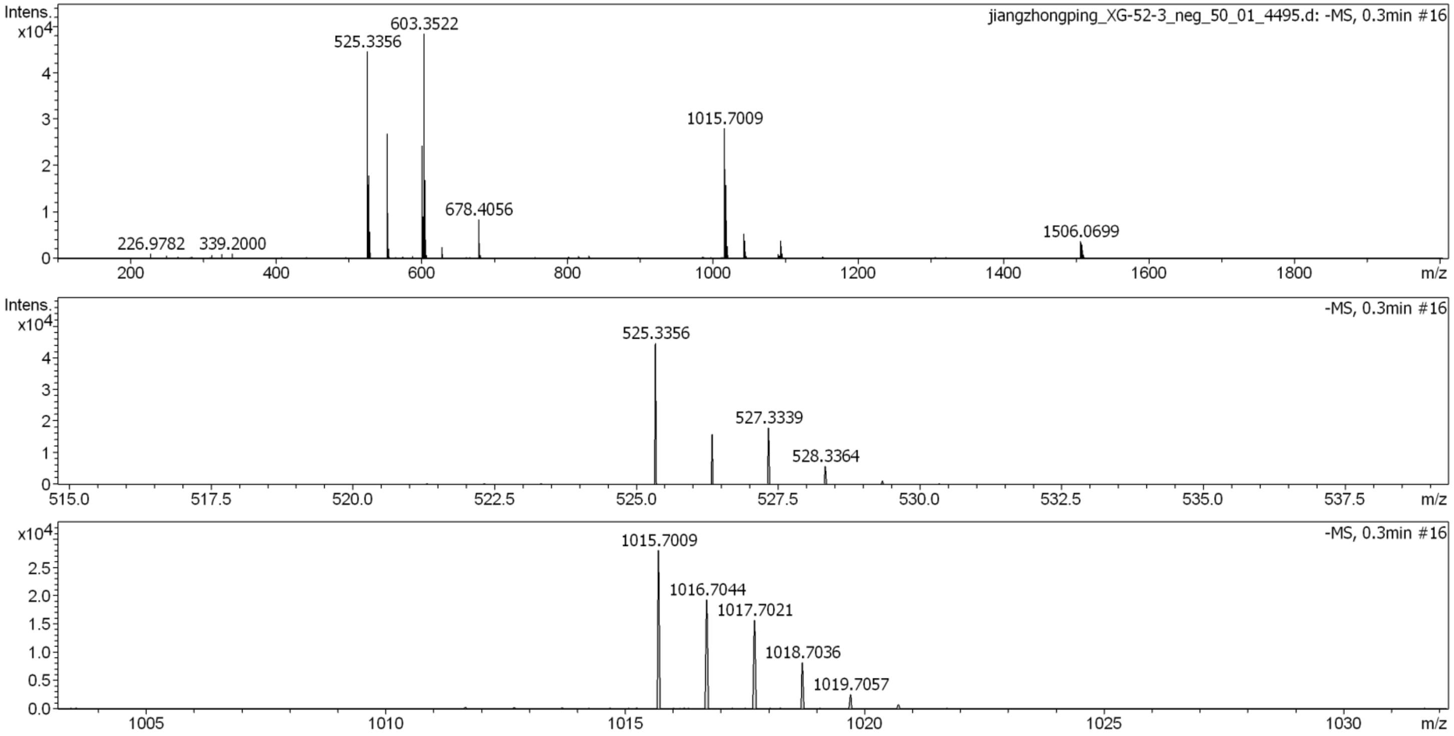

HR-ESIMS for compound **1**

Mass Spectrum SmartFormula Report

Analysis Info

Analysis Name	D:\Data\MS\data\201804\jiangzhongping_XG-52-3_neg_50_01_4495.d	Acquisition Date	4/3/2018 10:52:52 AM
Method	LC_Direct Infusion_neg_100-1000mz.m	Operator	SCSIO
Sample Name	jiangzhongping_XG-52-3_neg	Instrument	maXis 255552.00029
Comment			

Acquisition Parameter

Source Type	ESI	Ion Polarity	Negative	Set Nebulizer	0.4 Bar
Focus	Active	Set Capillary	4000 V	Set Dry Heater	180 °C
Scan Begin	100 m/z	Set End Plate Offset	-500 V	Set Dry Gas	4.0 l/min
Scan End	2000 m/z	Set Charging Voltage	0 V	Set Divert Valve	Waste
		Set Corona	0 nA	Set APCI Heater	0 °C

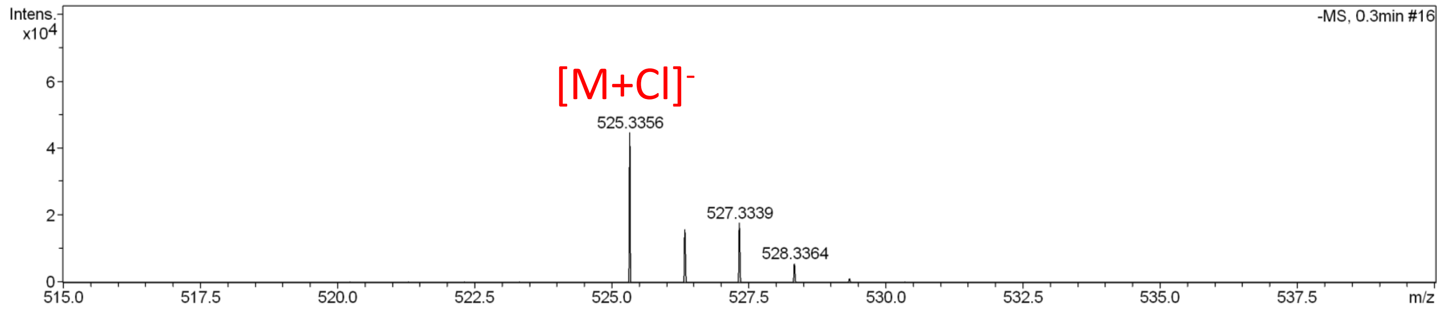

Meas. m/z	#	Ion Formula	Score	m/z	err [ppm]	err [mDa]	mSigma	rdb	e⁻ Conf	N-Rule
525.3356	1	C30H50ClO5	100.00	525.3352	-0.8	-0.4	14.0	5.5	even	ok
1015.7009	1	C60H100ClO10	100.00	1015.7011	0.1	0.1	10.4	10.5	even	ok

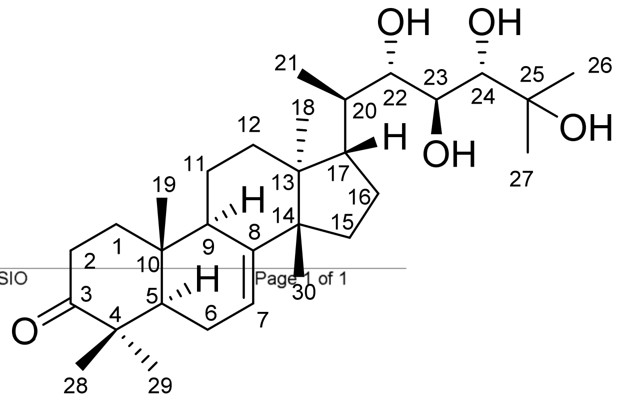

28 29

^{1}H NMR (400 MHz) spectrum of compound **1** in $CDCl_3$

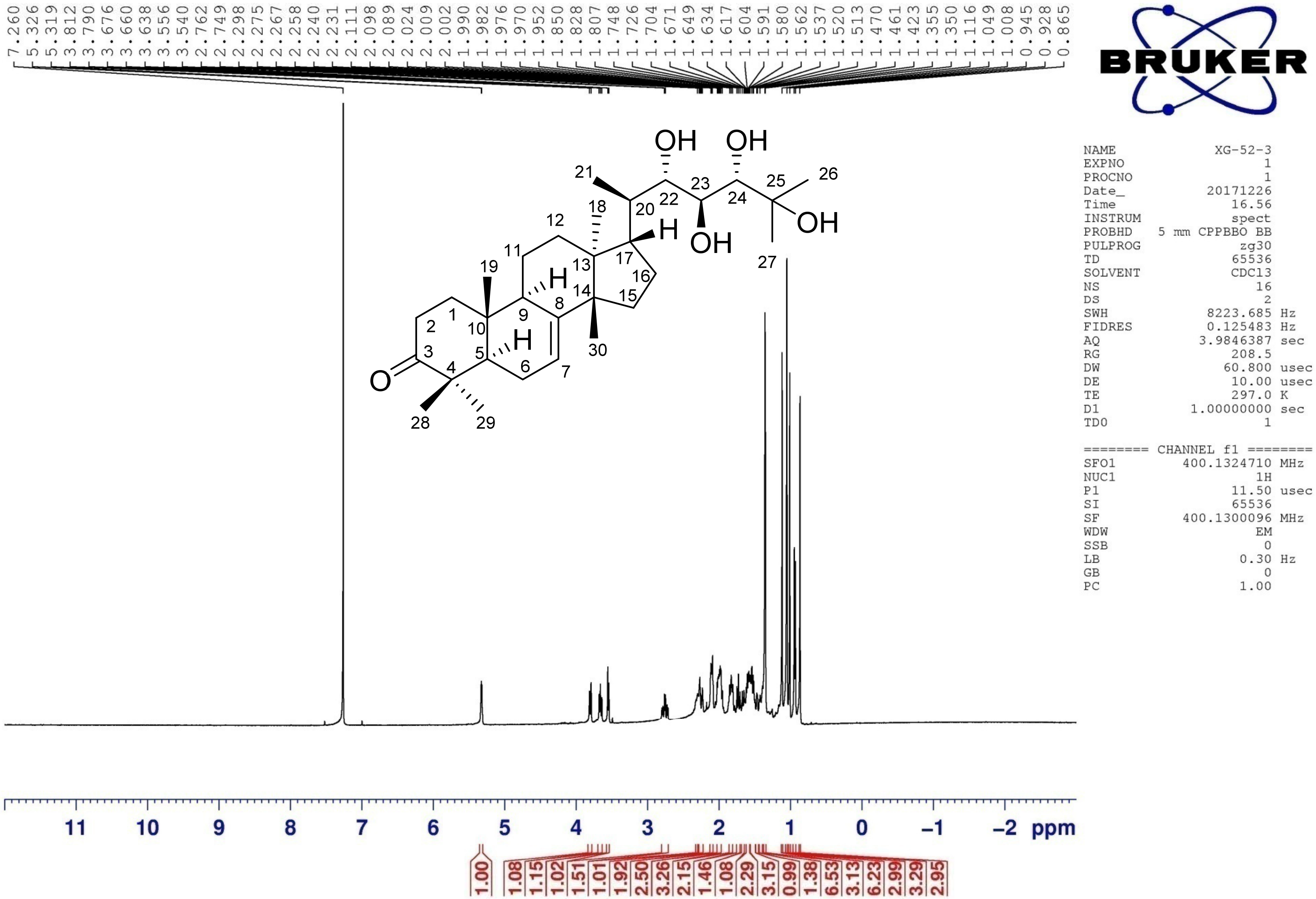

^{1}H NMR (400 MHz) spectrum of compound **1** in $CDCl_3$

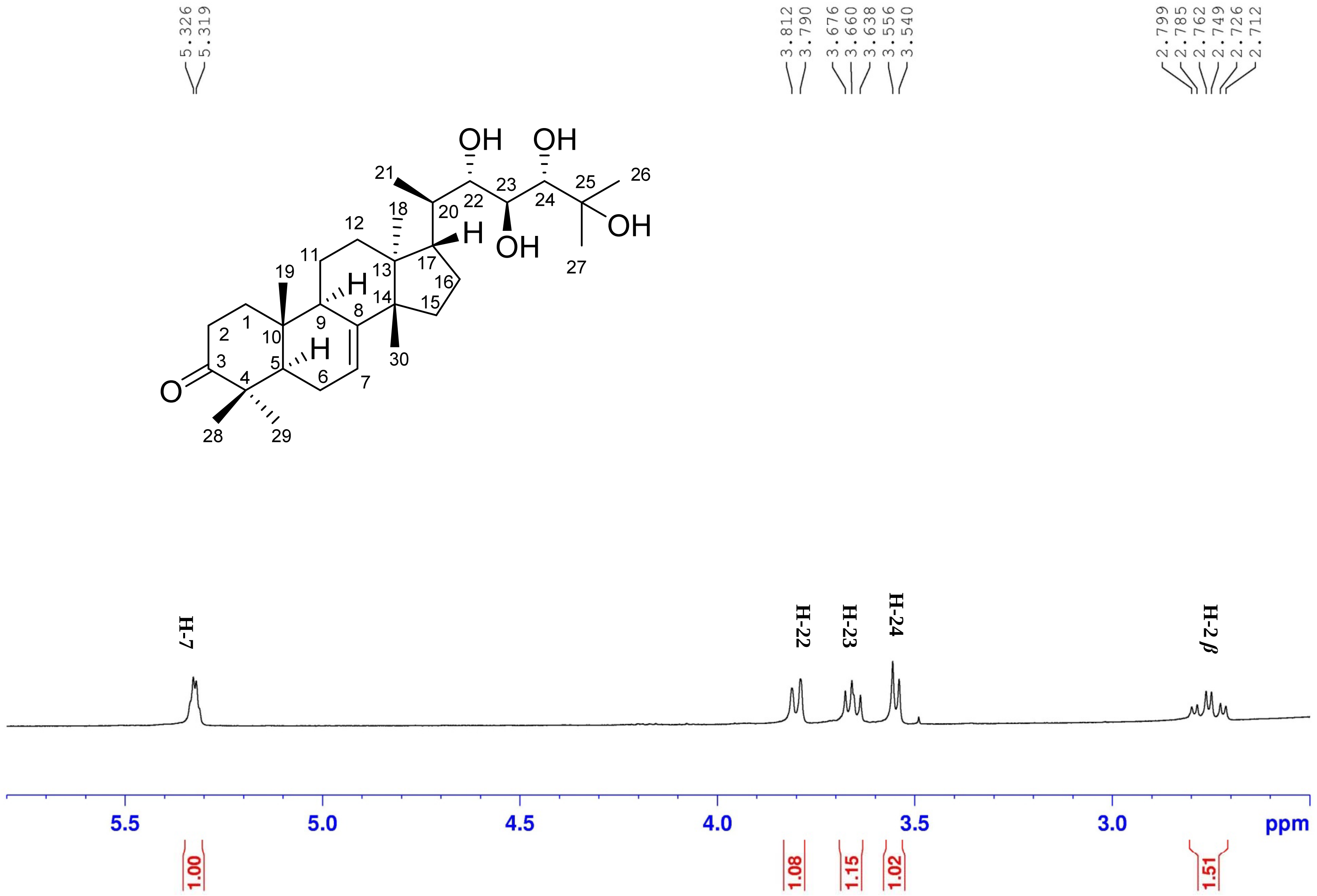

^{1}H NMR (400 MHz) spectrum of compound **1** in $CDCl_3$

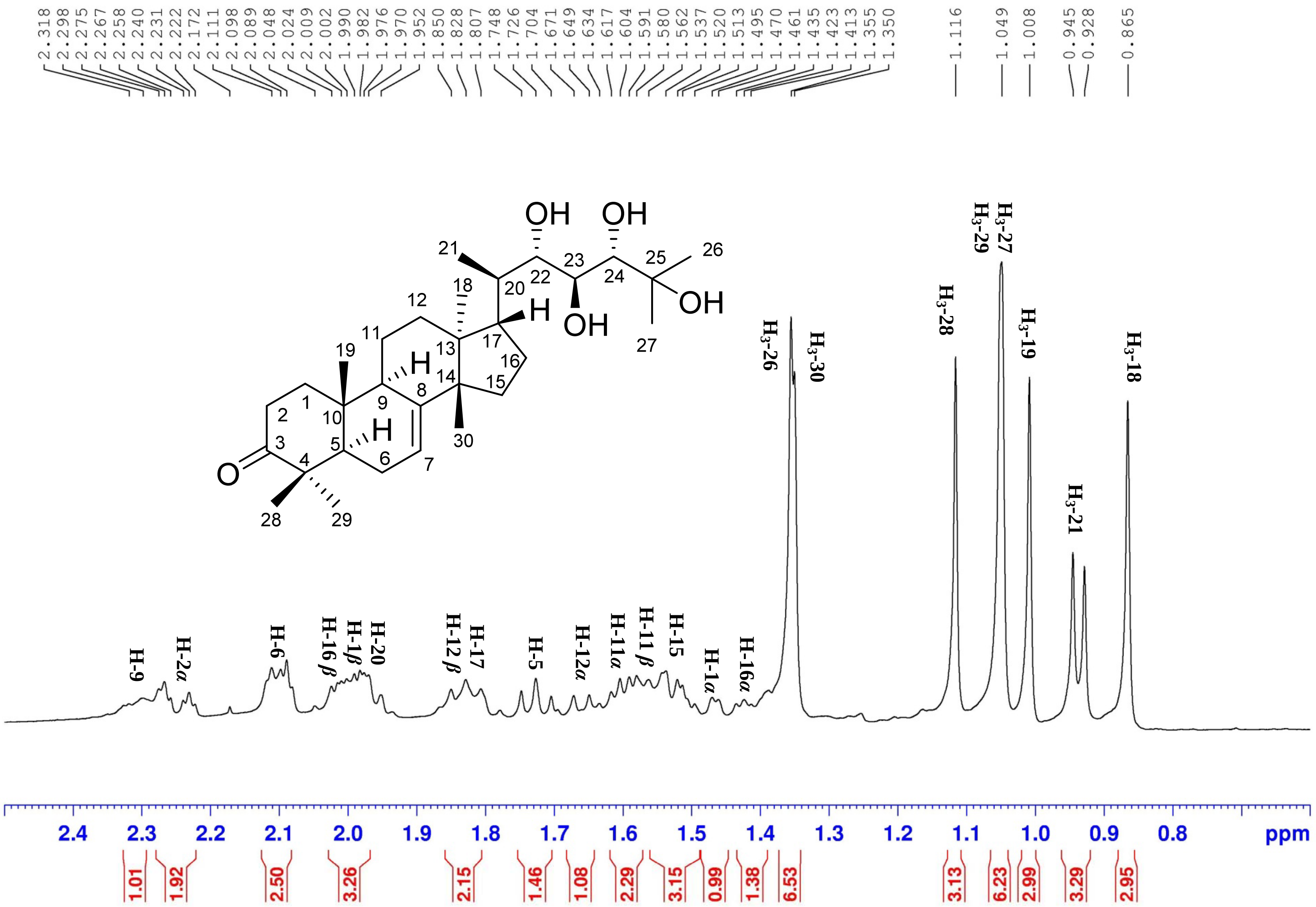

^{13}C NMR (100 MHz) spectrum of compound **1** in $CDCl_3$

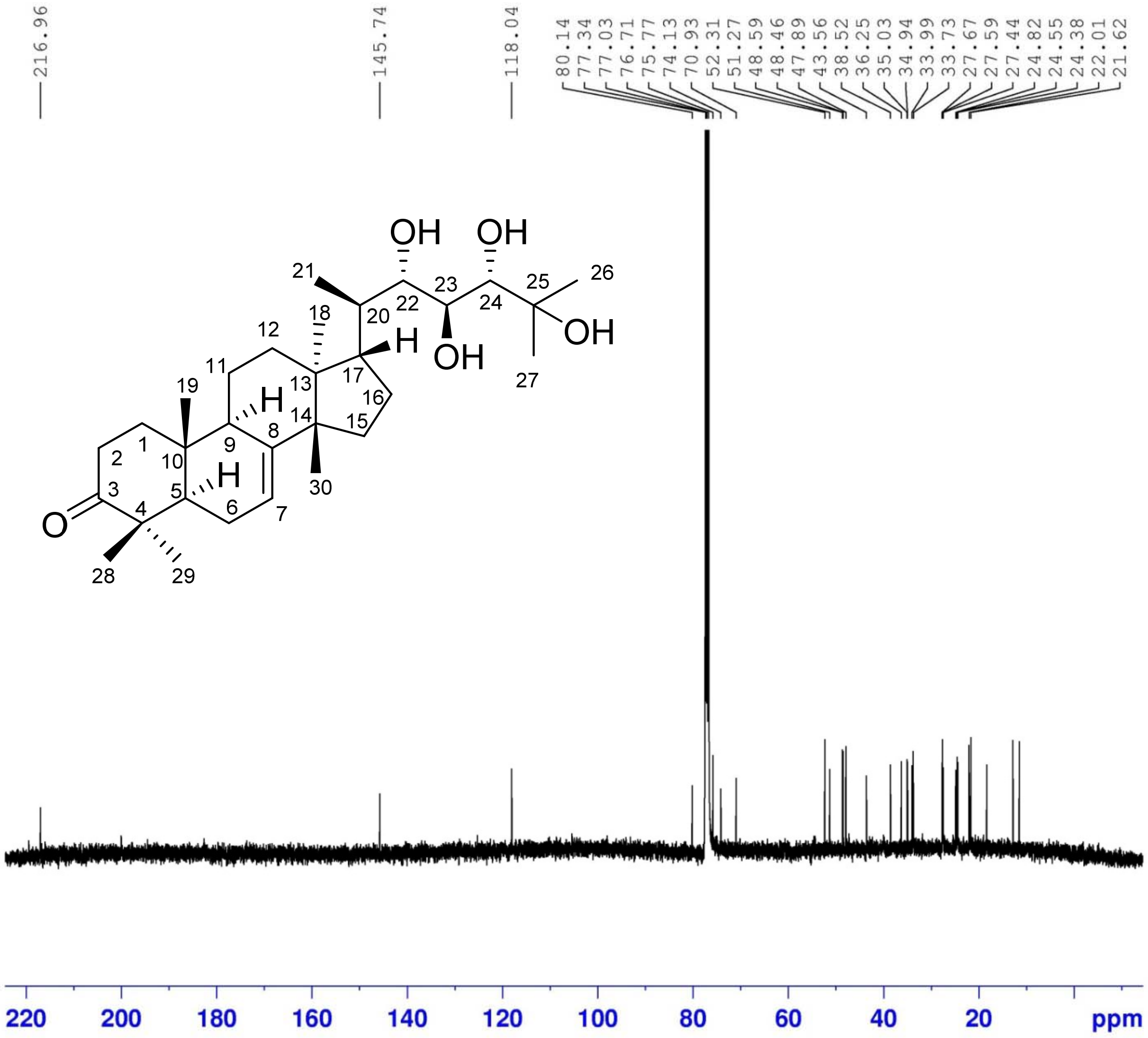

NAME	XG-52-3	
EXPNO	2	
PROCNO	1	
Date_	20171226	
Time	17.56	
INSTRUM	spect	
PROBHD	5 mm CPPBBO BB	
PULPROG	zgpg30	
TD	65536	
SOLVENT	CDCl3	
NS	1024	
DS	4	
SWH	24038.461	Hz
FIDRES	0.366798	Hz
AQ	1.3631988	sec
RG	85.34	
DW	20.800	usec
DE	18.00	usec
TE	297.0	K
D1	2.00000000	sec
D11	0.03000000	sec
TD0	1	

======== CHANNEL f1 ========

SFO1	100.6233324	MHz
NUC1	13C	
P1	10.00	usec
SI	32768	
SF	100.6127685	MHz
WDW	EM	
SSB	0	
LB	1.00	Hz
GB	0	
PC	1.40	

^{13}C NMR (100 MHz) spectrum of compound **1** in $CDCl_3$

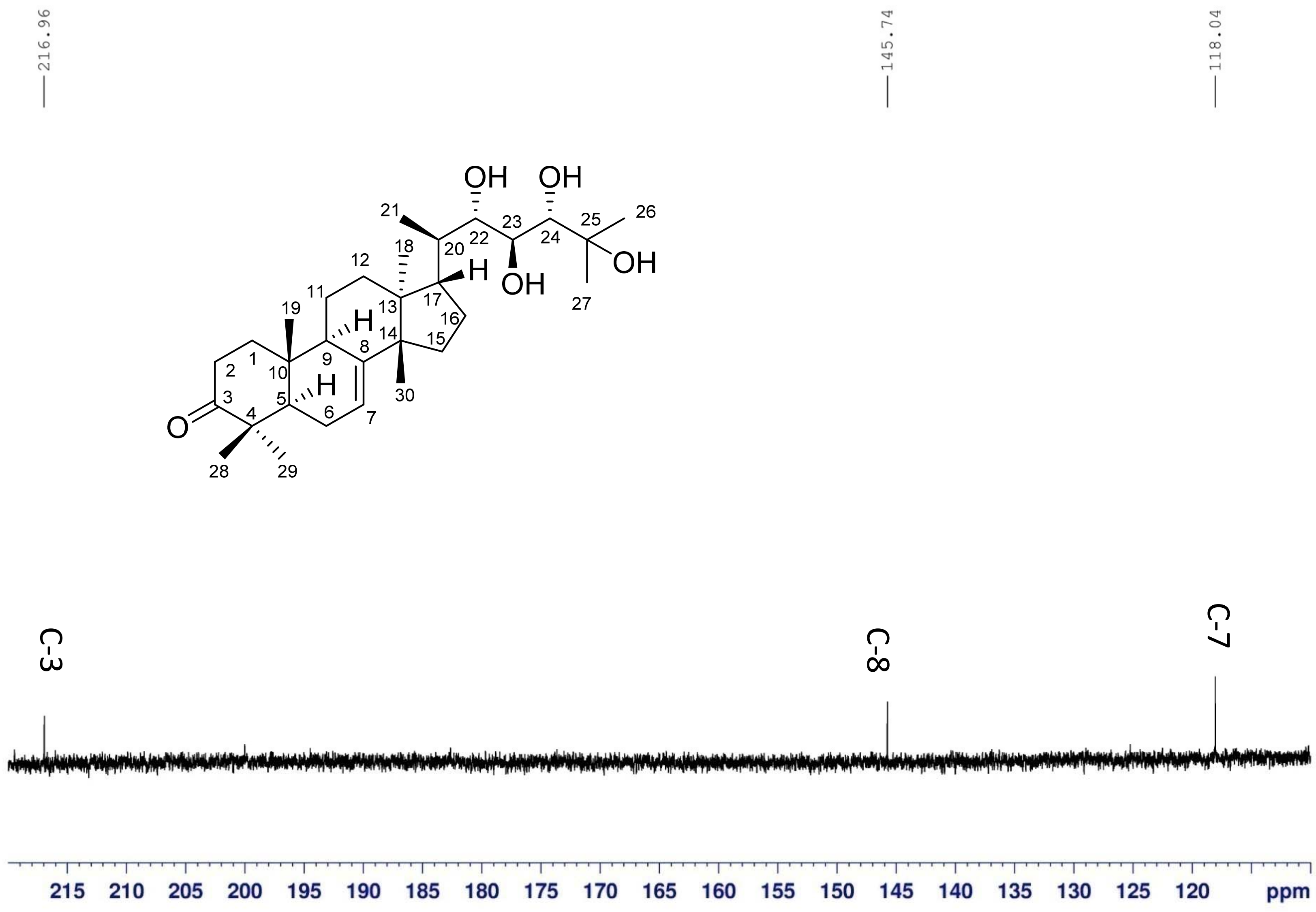

^{13}C NMR (100 MHz) spectrum of compound **1** in $CDCl_3$

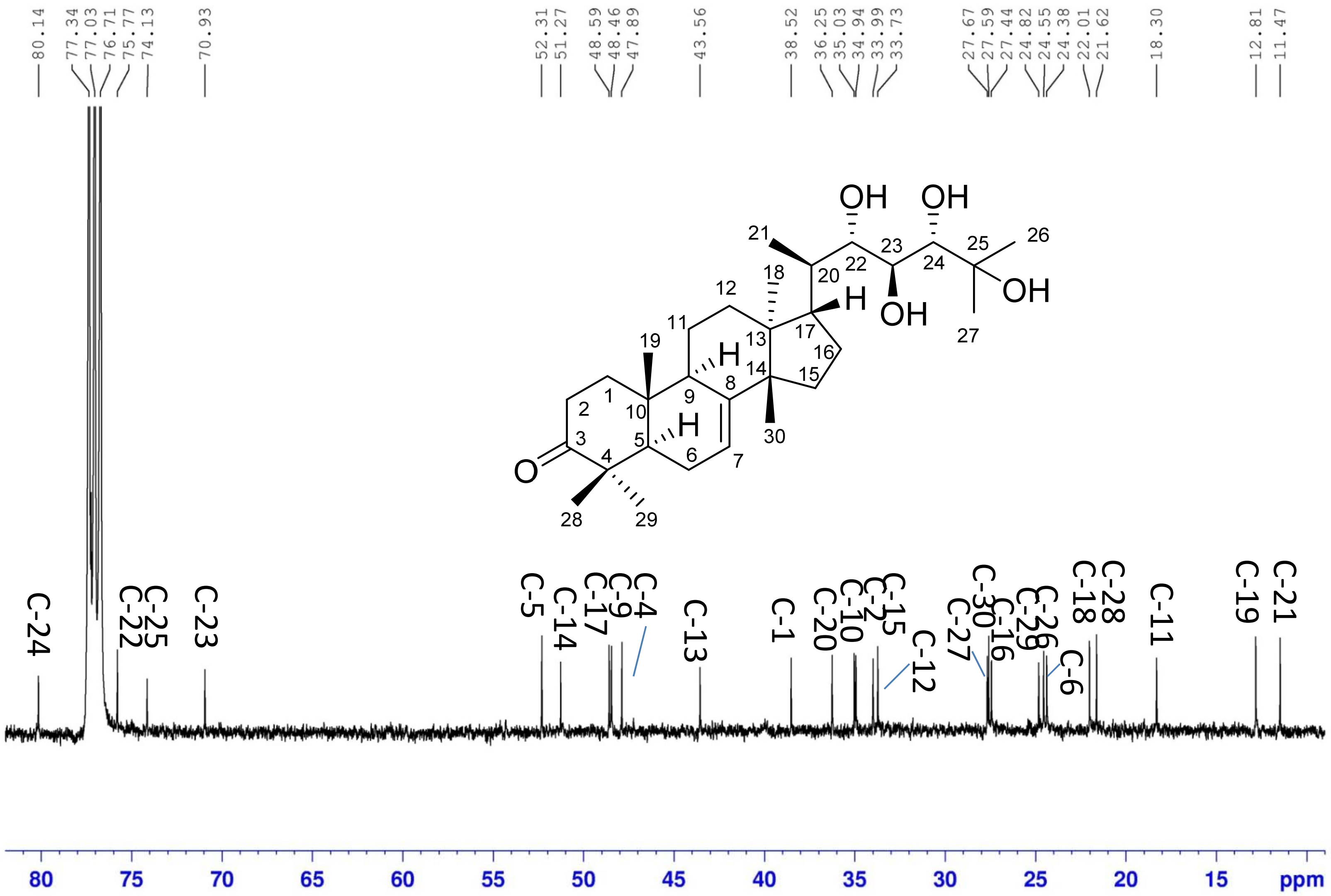

DEPT135 (100 MHz) spectrum of compound **1** in $CDCl_3$

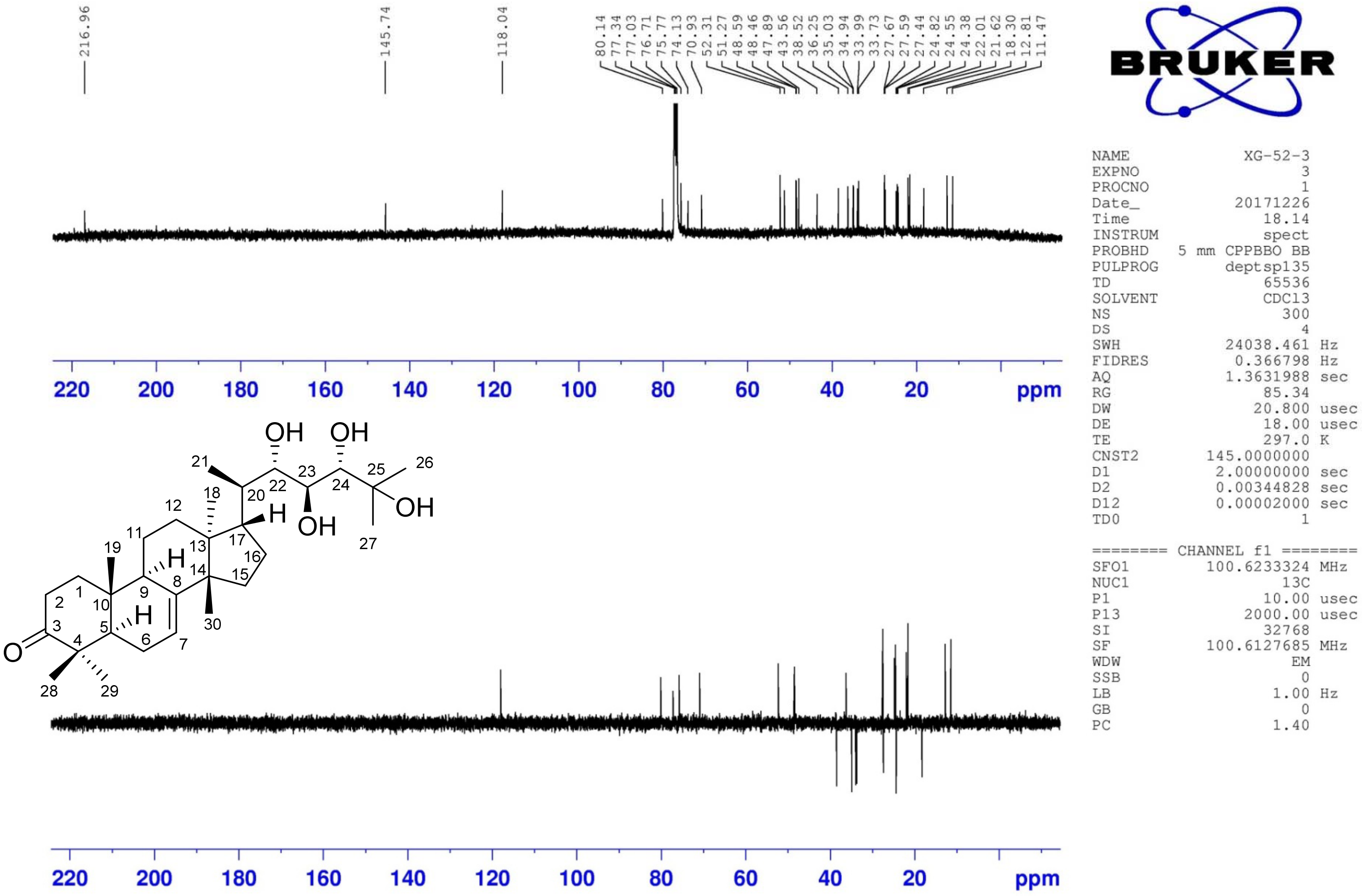

DEPT135 (100 MHz) spectrum of compound **1** in $CDCl_3$

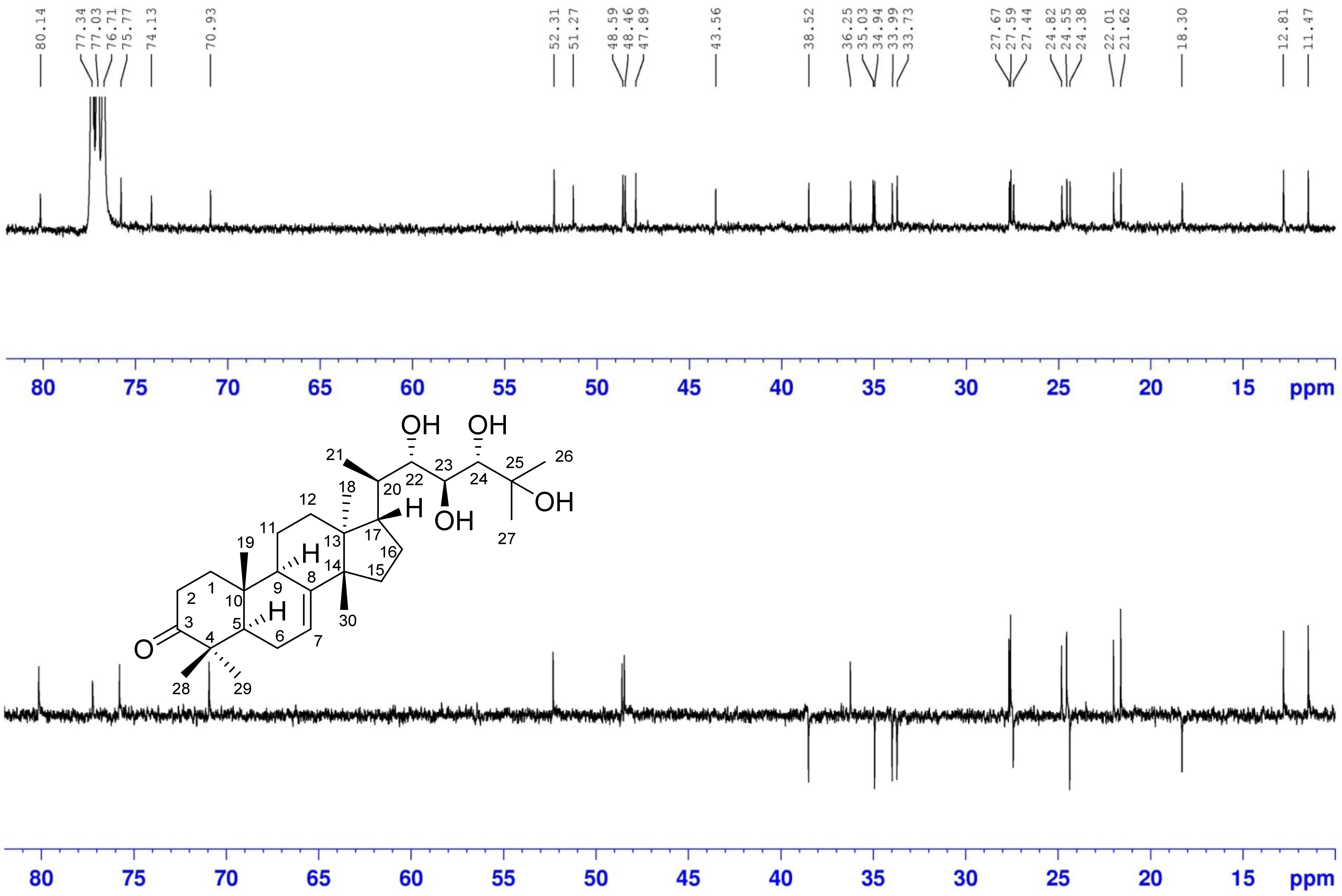

^{1}H-^{1}H COSY (400 MHz) spectrum of compound **1** in $CDCl_3$

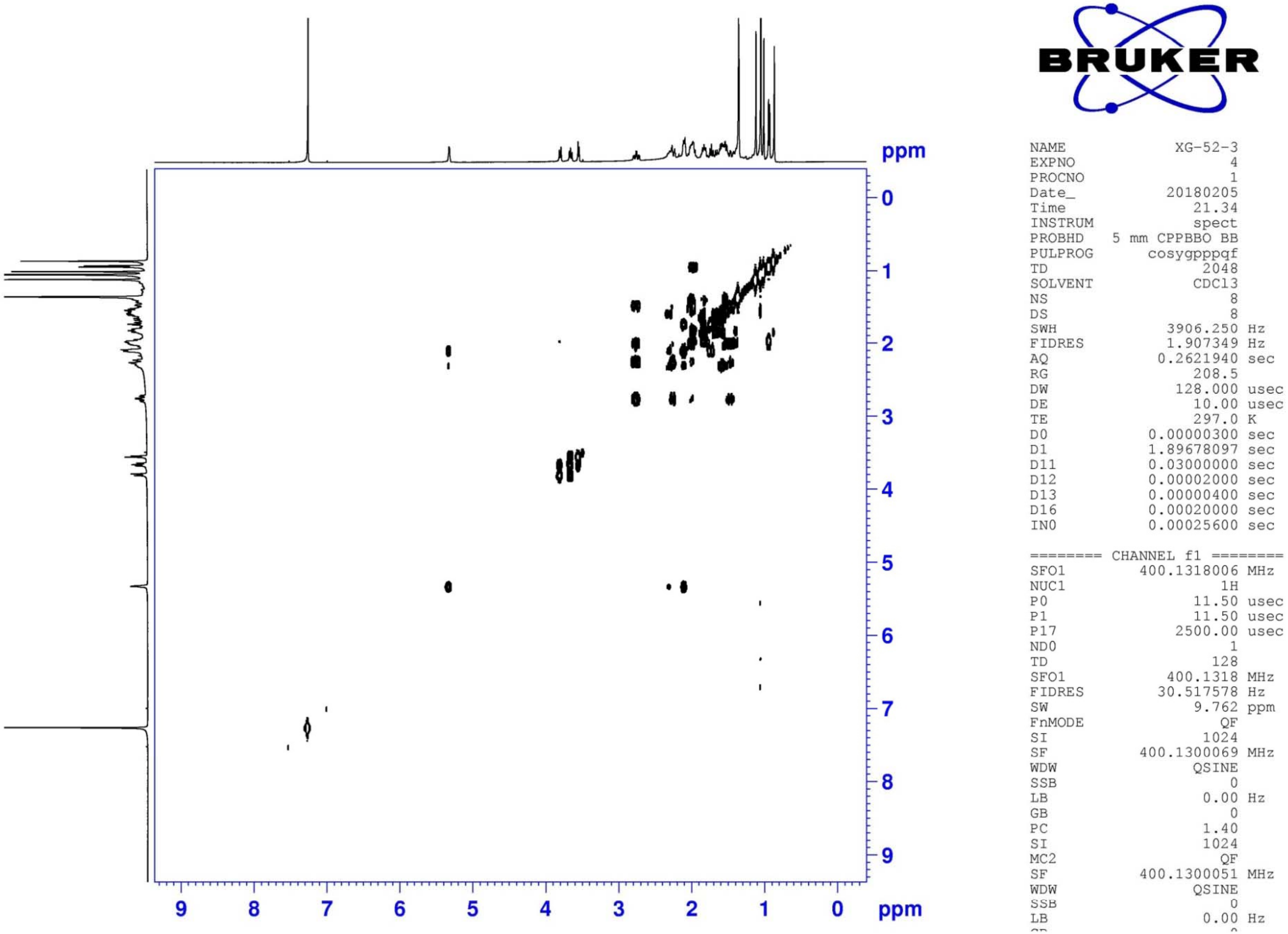

^{1}H-^{1}H COSY (400 MHz) spectrum of compound **1** in $CDCl_3$

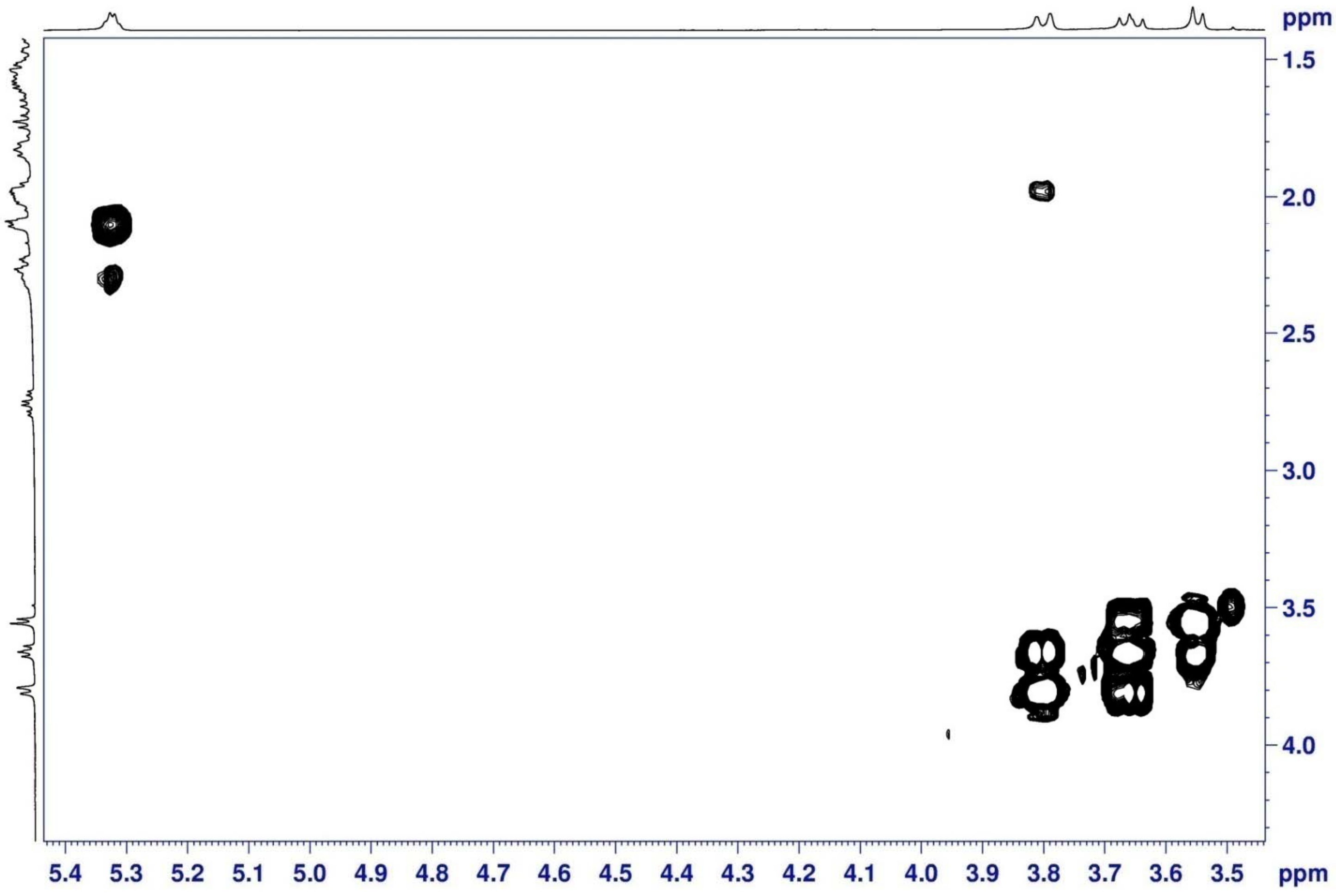

^{1}H-^{1}H COSY (400 MHz) spectrum of compound **1** in $CDCl_3$

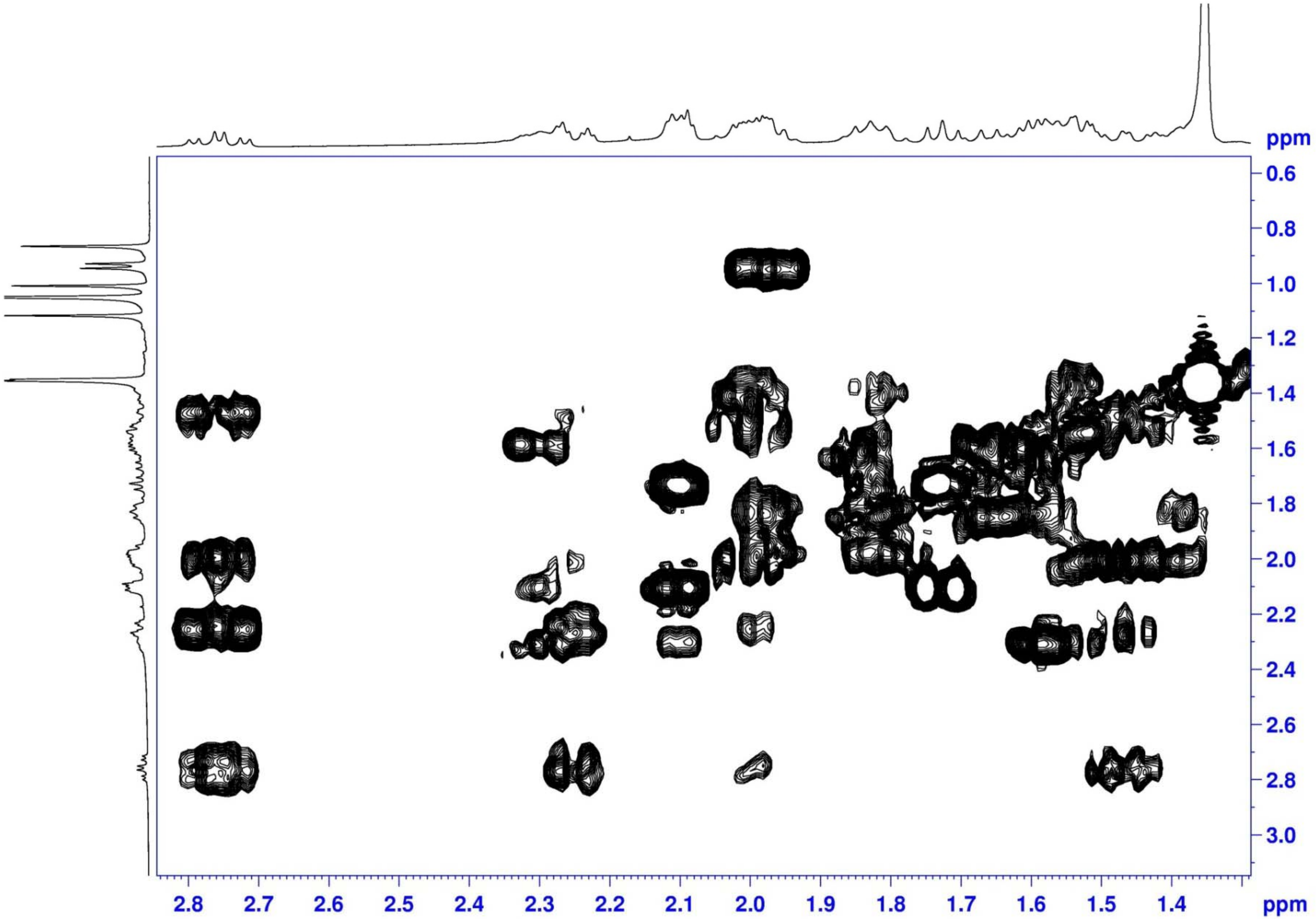

HSQC (400 MHz) spectrum of compound **1** in $CDCl_3$

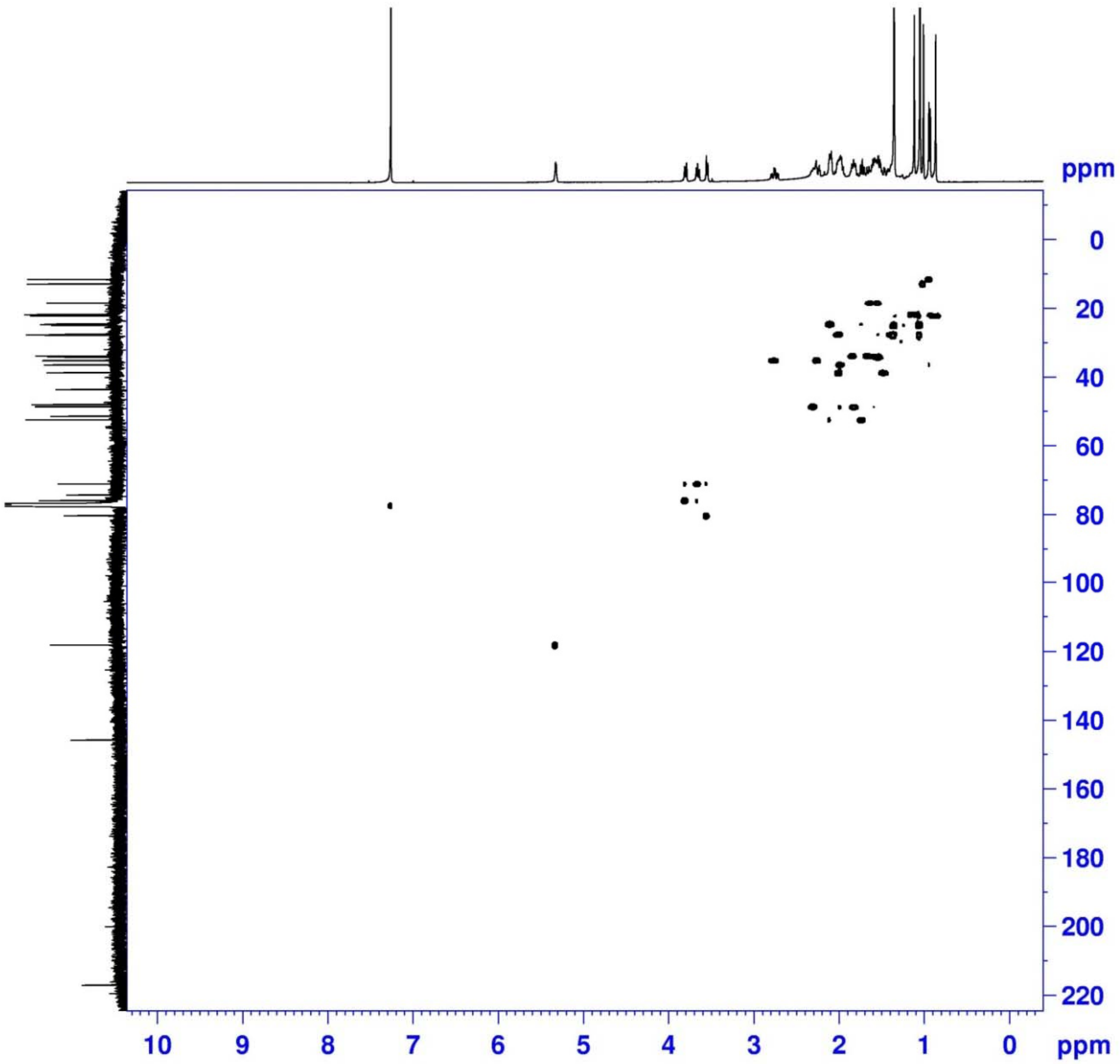


```
NAME            XG-52-3
EXPNO                 5
PROCNO                1
Date_          20180205
Time              22.14
INSTRUM           spect
PROBHD   5 mm CPPBBO BB
PULPROG     hsqcetgpsi2
TD                 1024
SOLVENT           CDCl3
NS                   16
DS                   16
SWH            4302.926 Hz
FIDRES         4.202076 Hz
AQ            0.1190388 sec
RG                208.5
DW              116.200 usec
DE                10.00 usec
TE                297.0 K
CNST2       145.0000000
D0           0.00000300 sec
D1           1.46497905 sec
D4           0.00172414 sec
D11          0.03000000 sec
D16          0.00020000 sec
D24          0.00086207 sec
IN0          0.00002080 sec
ZGOPTNS

======== CHANNEL f1 ========
SFO1        400.1320007 MHz
NUC1                 1H
P1                11.50 usec
P2                23.00 usec
P28                0.00 usec
ND0                   2
TD                  256
SFO1           100.6233 MHz
FIDRES        93.900238 Hz
SW              238.896 ppm
FnMODE    Echo-Antiecho
SI                 1024
SF          400.1300070 MHz
WDW               QSINE
SSB                   2
LB                 0.00 Hz
GB                    0
PC                 1.40
SI                 1024
MC2       echo-antiecho
SF          100.6127585 MHz
WDW               QSINE
SSB                   2
LB                 0.00 Hz
```

HSQC (400 MHz) spectrum of compound **1** in $CDCl_3$

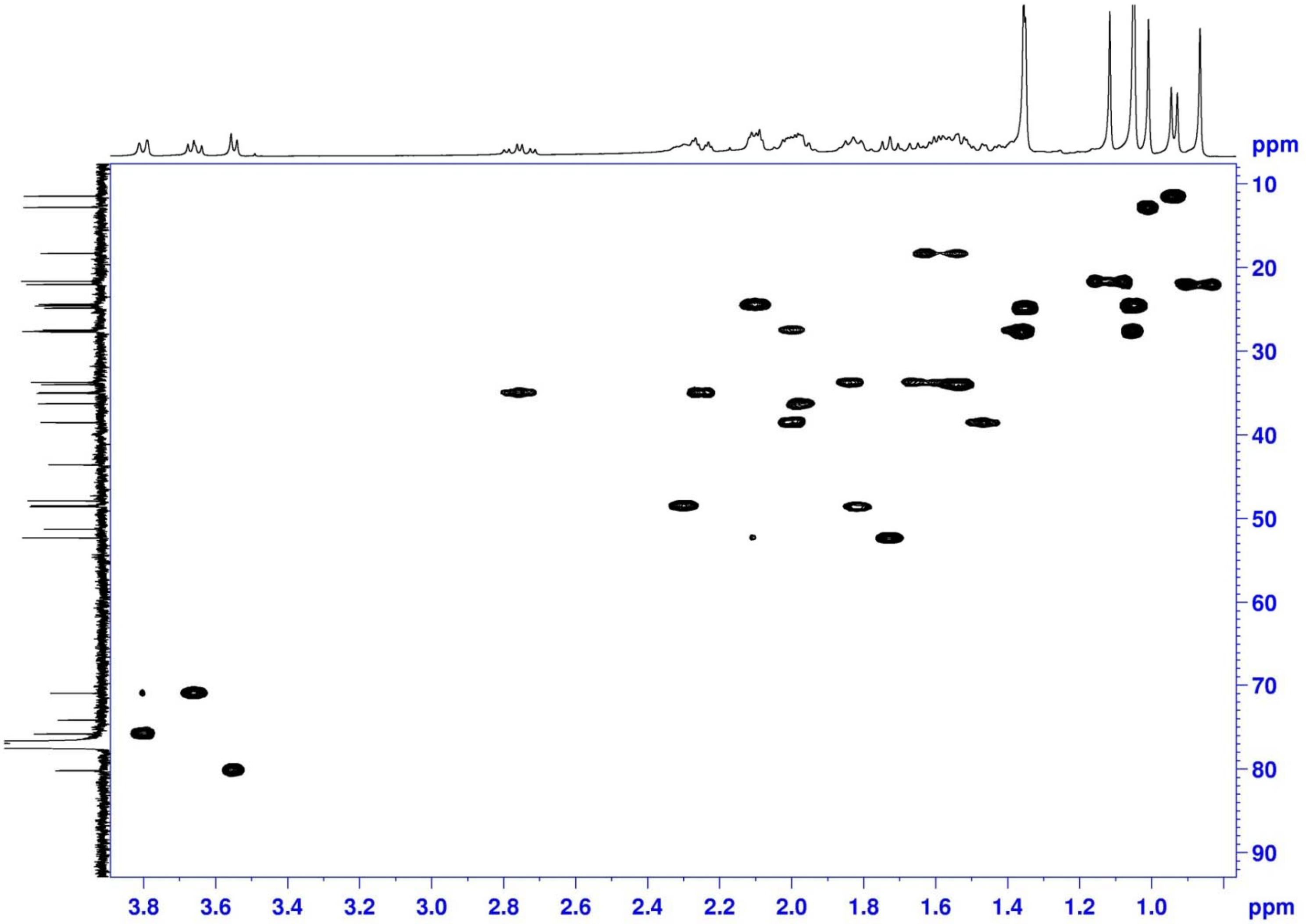

HSQC (400 MHz) spectrum of compound **1** in $CDCl_3$

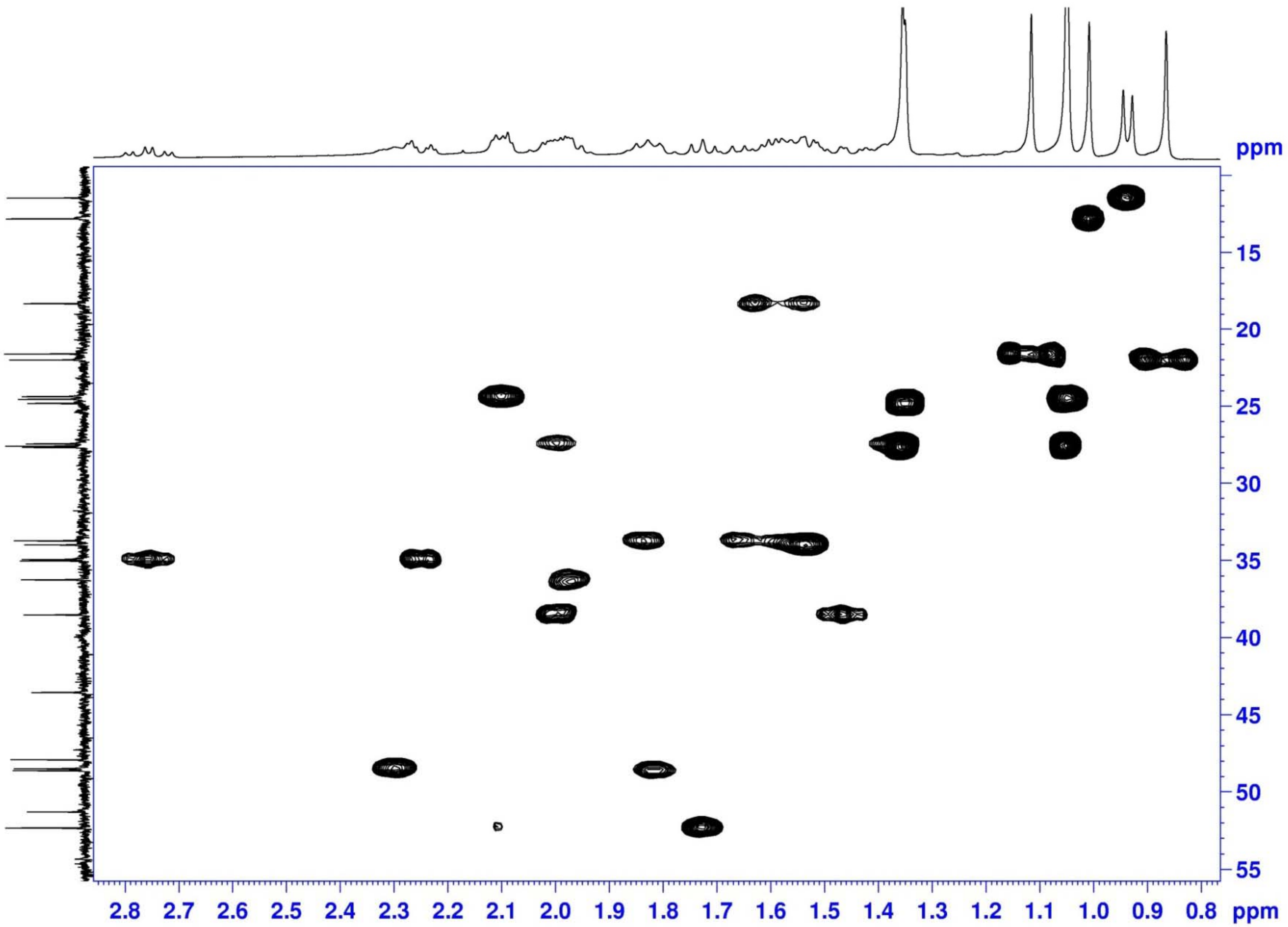

HMBC (400 MHz) spectrum of compound **1** in $CDCl_3$

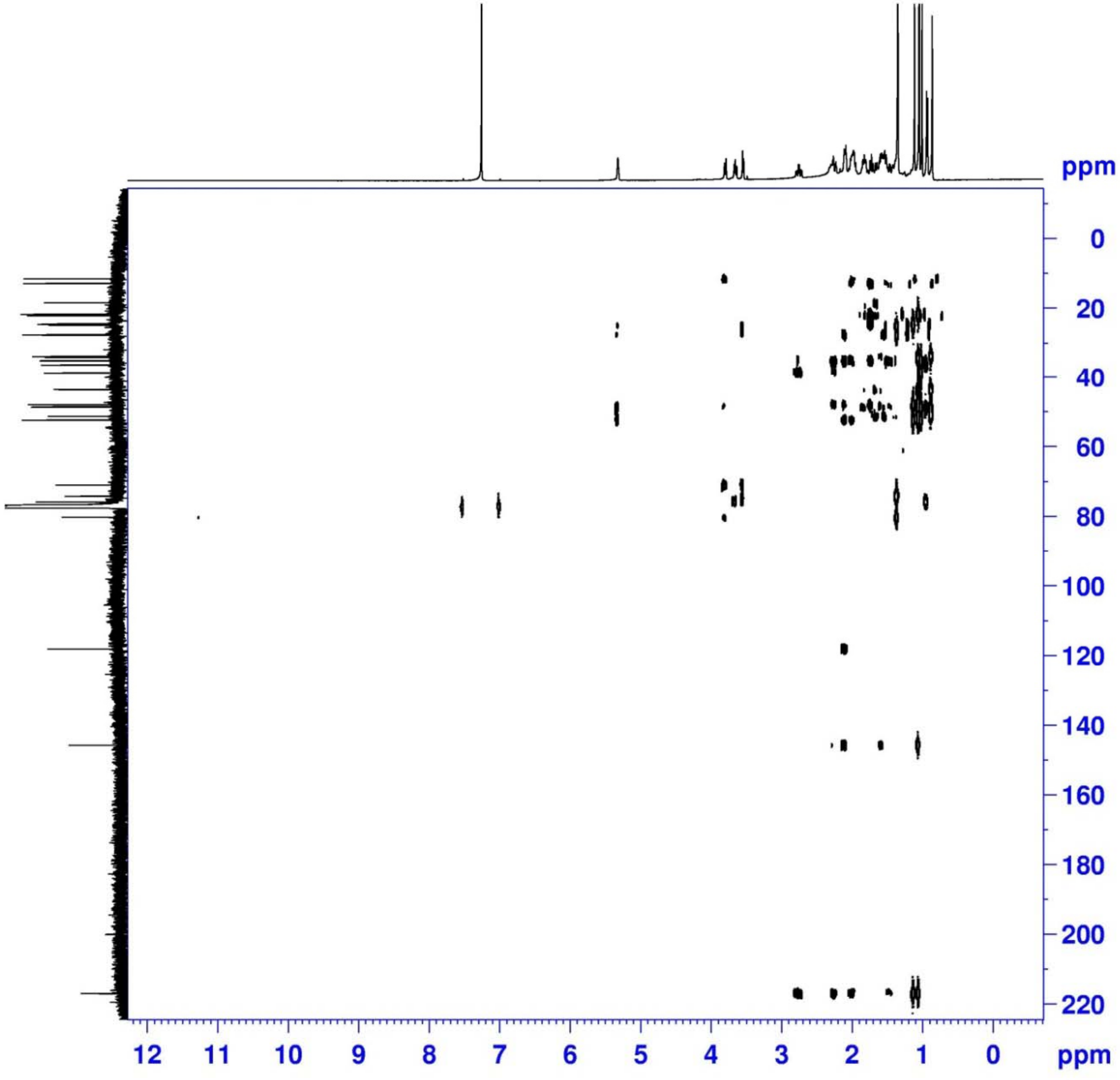

NAME	XG-52-3
EXPNO	6
PROCNO	1
Date_	20180206
Time	0.05
INSTRUM	spect
PROBHD	5 mm CPPBBO BB
PULPROG	hmbcgplpndqf
TD	4096
SOLVENT	CDCl3
NS	32
DS	16
SWH	5197.505 Hz
FIDRES	1.268922 Hz
AQ	0.3940852 sec
RG	208.5
DW	96.200 usec
DE	10.00 usec
TE	297.0 K
CNST2	145.0000000
CNST13	10.0000000
D0	0.00000300 sec
D1	1.50000000 sec
D2	0.00344828 sec
D6	0.05000000 sec
D16	0.00020000 sec
IN0	0.00002080 sec

======== CHANNEL f1 ========

SFO1	400.1323208 MHz
NUC1	1H
P1	11.50 usec
P2	23.00 usec
ND0	2
TD	128
SFO1	100.6233 MHz
FIDRES	187.800476 Hz
SW	238.896 ppm
FnMODE	QF
SI	2048
SF	400.1300070 MHz
WDW	SINE
SSB	0
LB	0.00 Hz
GB	0
PC	1.40
SI	1024
MC2	QF
SF	100.6127630 MHz
WDW	SINE
SSB	0

HMBC (400 MHz) spectrum of compound **1** in $CDCl_3$

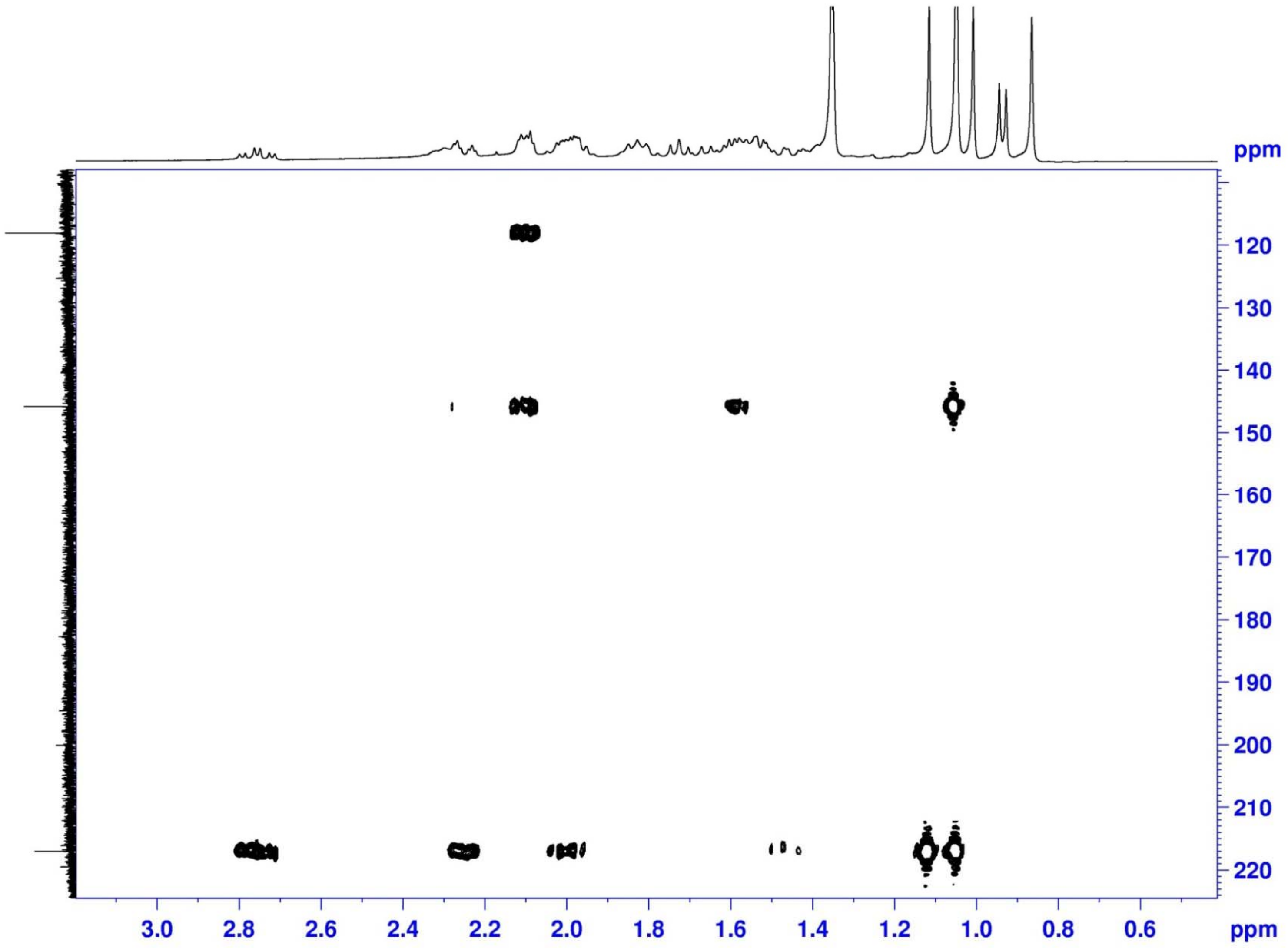

HMBC (400 MHz) spectrum of compound **1** in $CDCl_3$

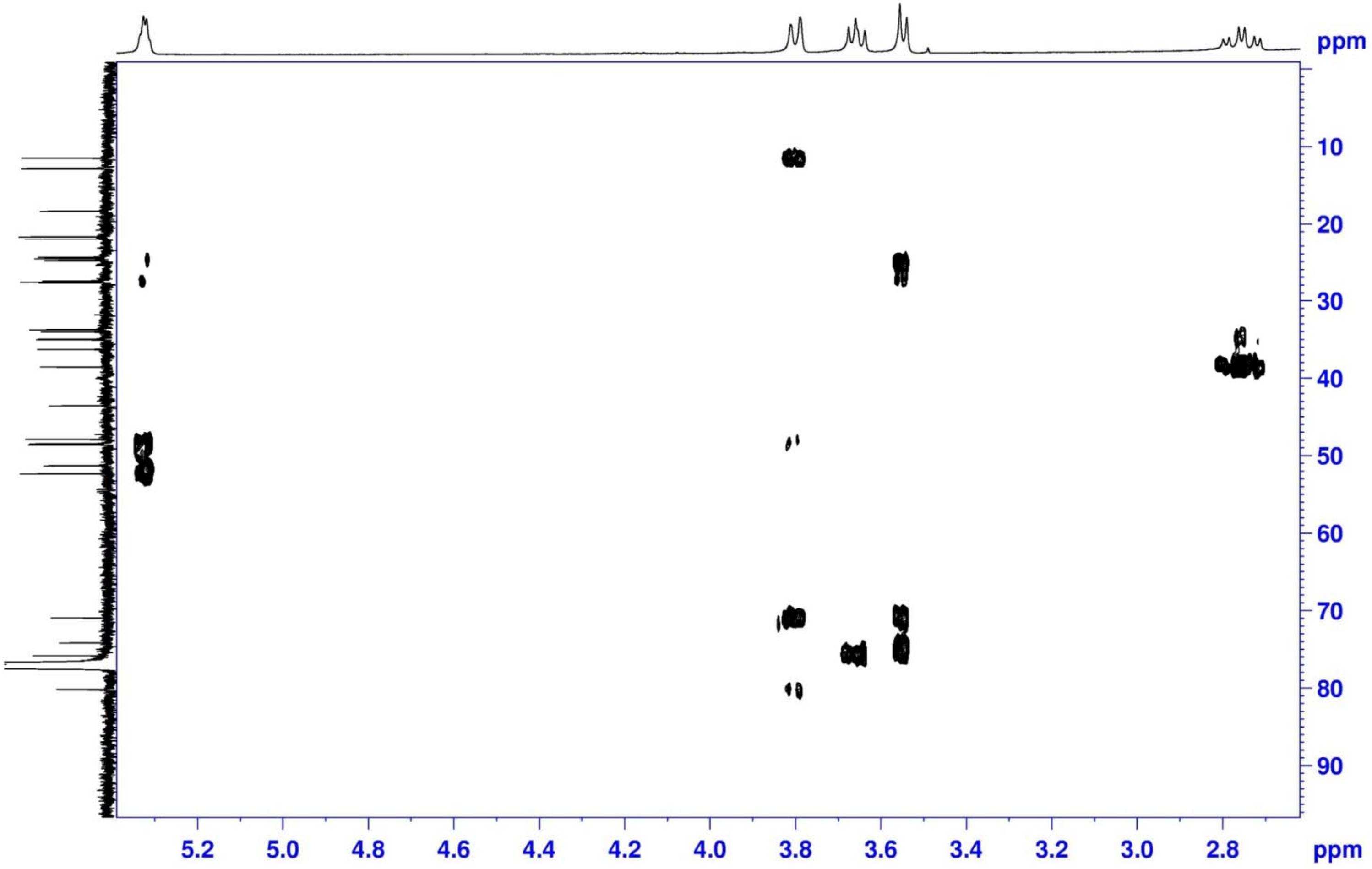

HMBC (400 MHz) spectrum of compound **1** in $CDCl_3$

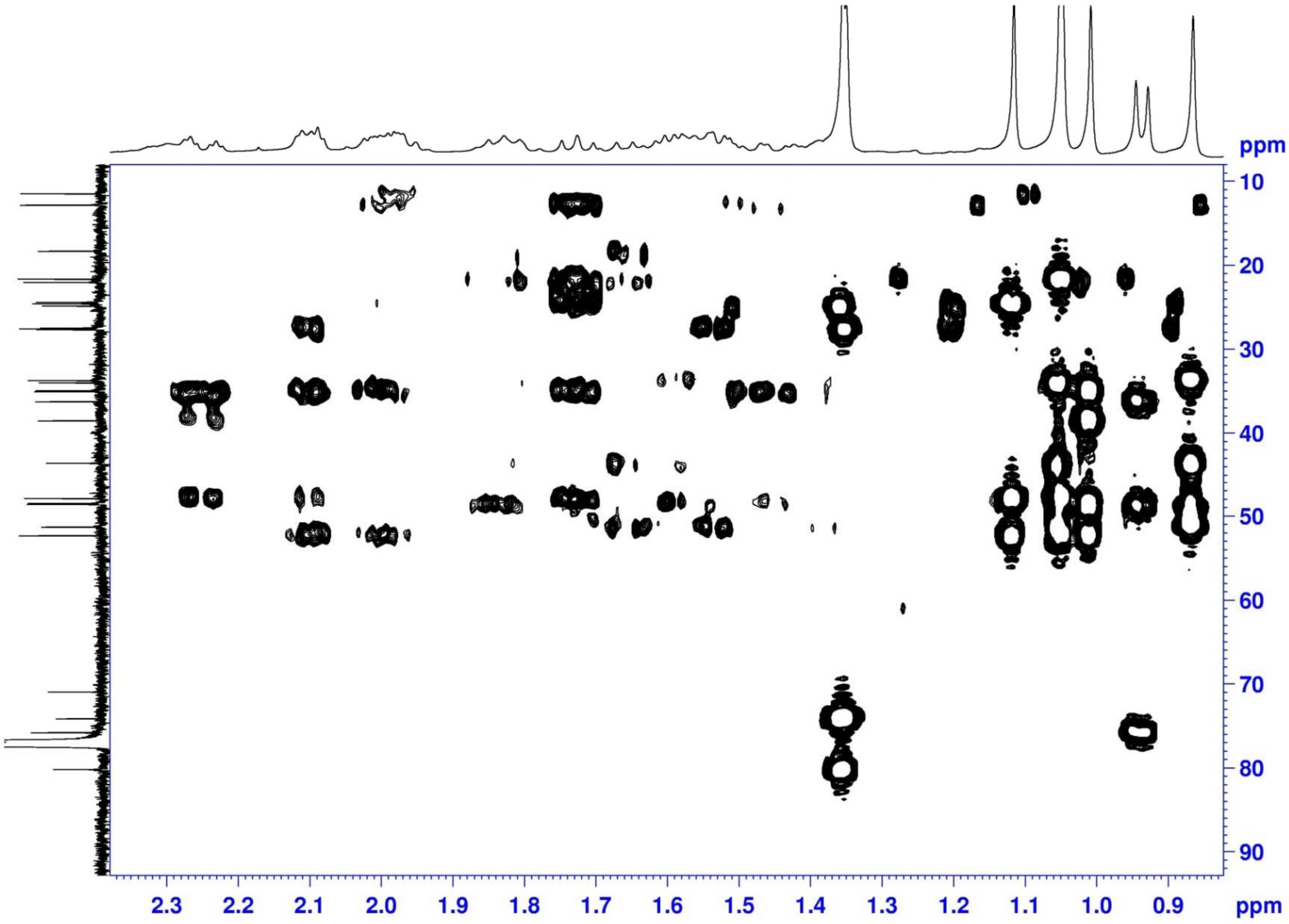

HMBC (400 MHz) spectrum of compound **1** in $CDCl_3$

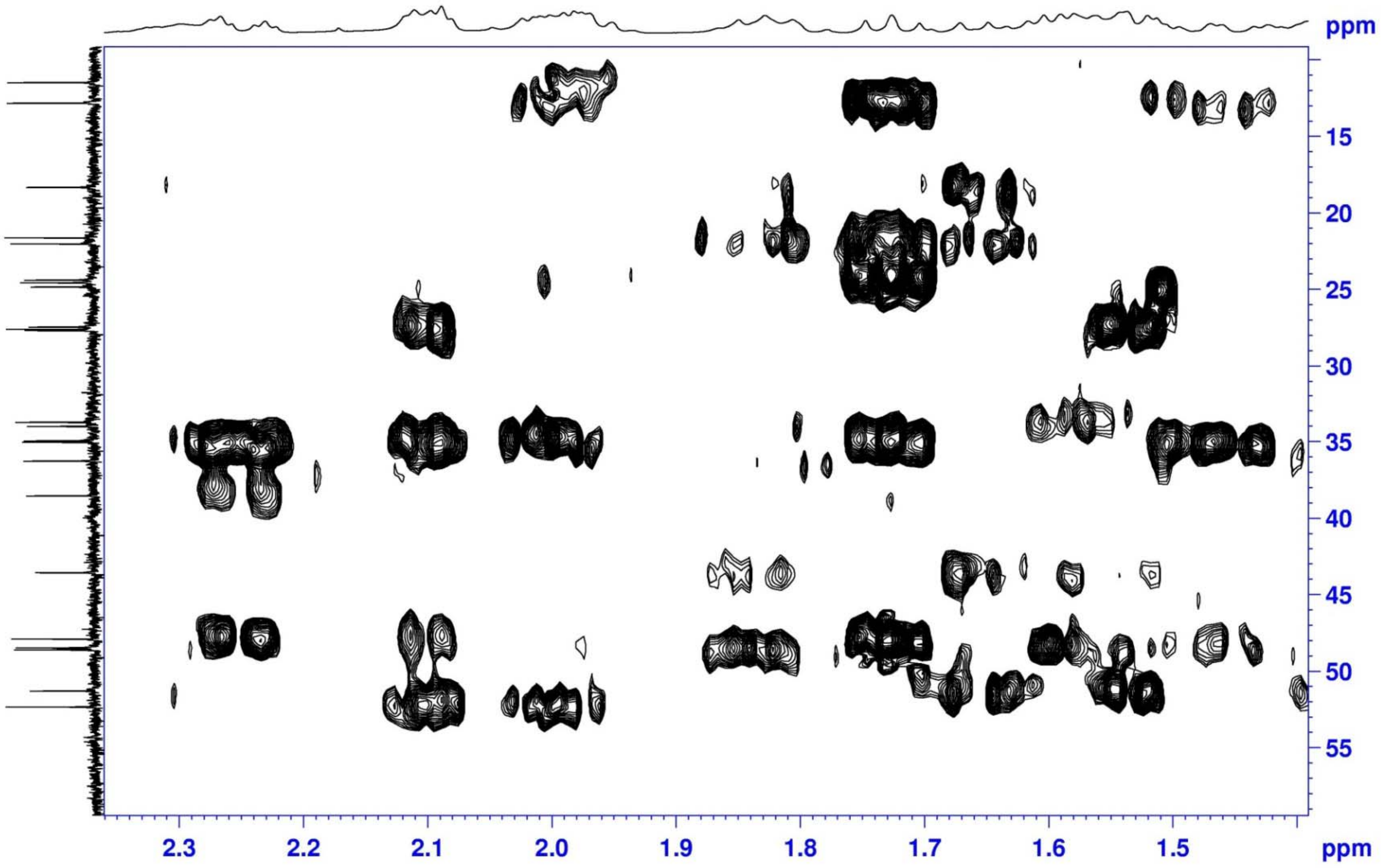

HMBC (400 MHz) spectrum of compound **1** in $CDCl_3$

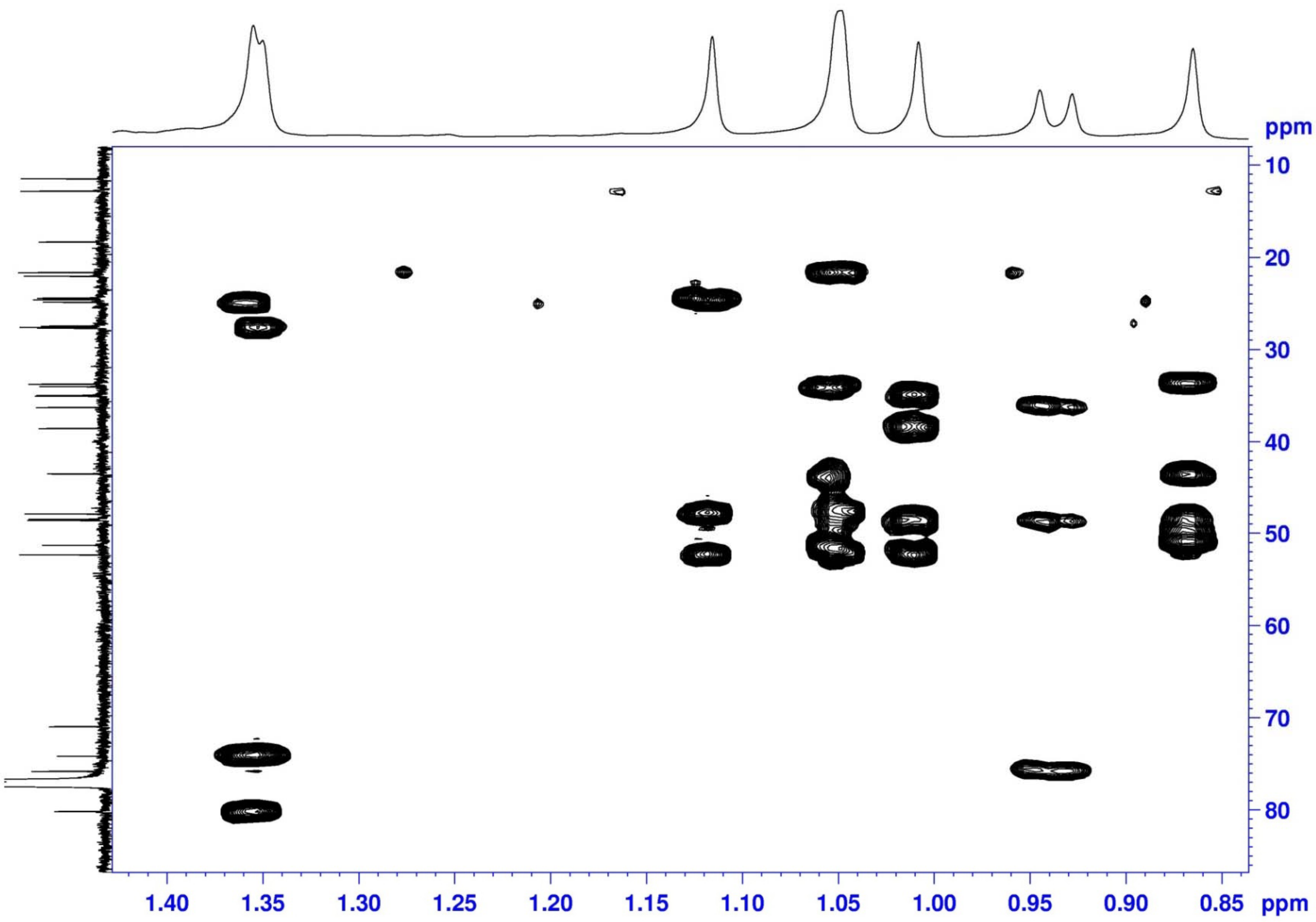

NOESY (400 MHz) spectrum of compound **1** in $CDCl_3$

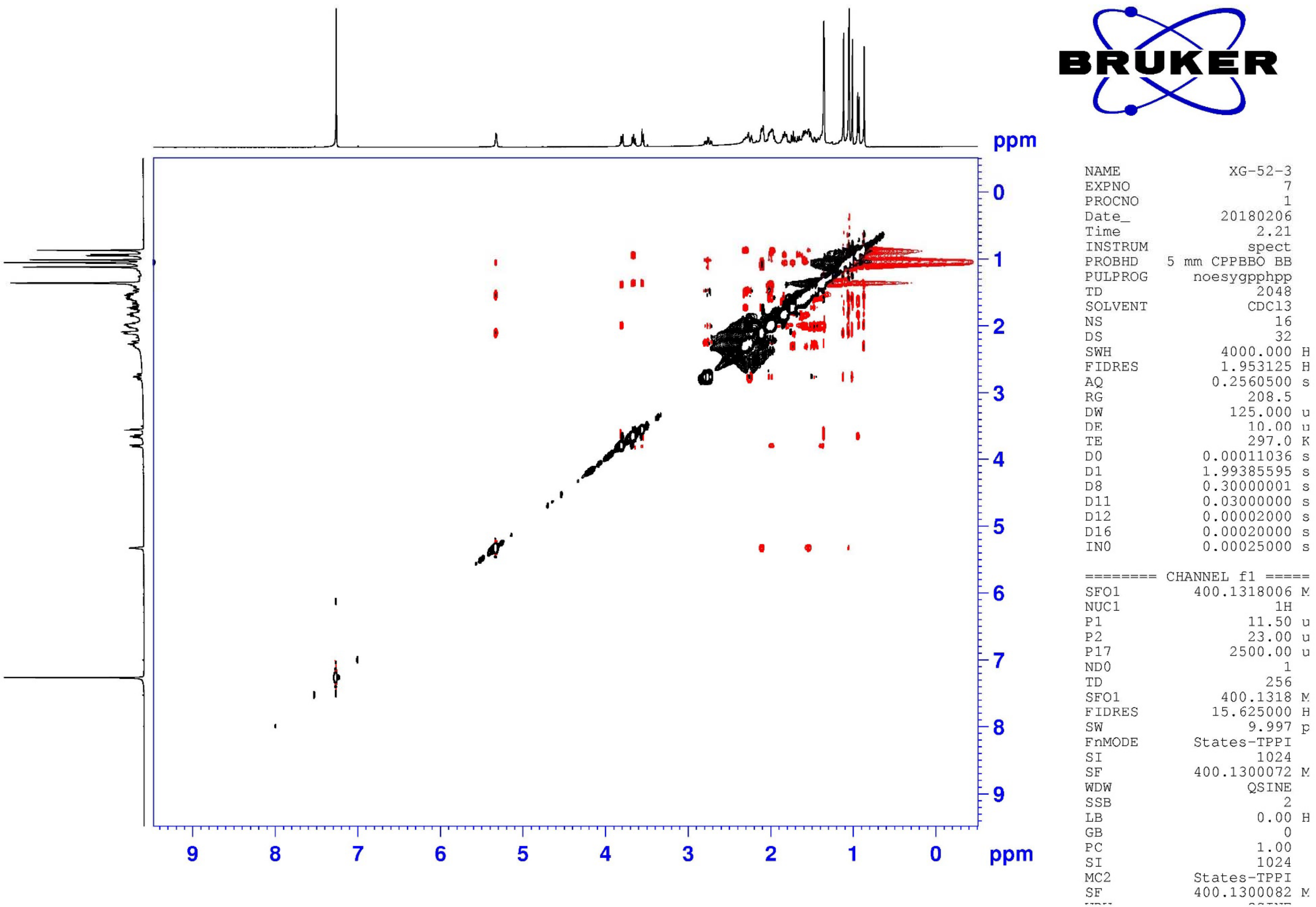

NOESY (400 MHz) spectrum of compound **1** in $CDCl_3$

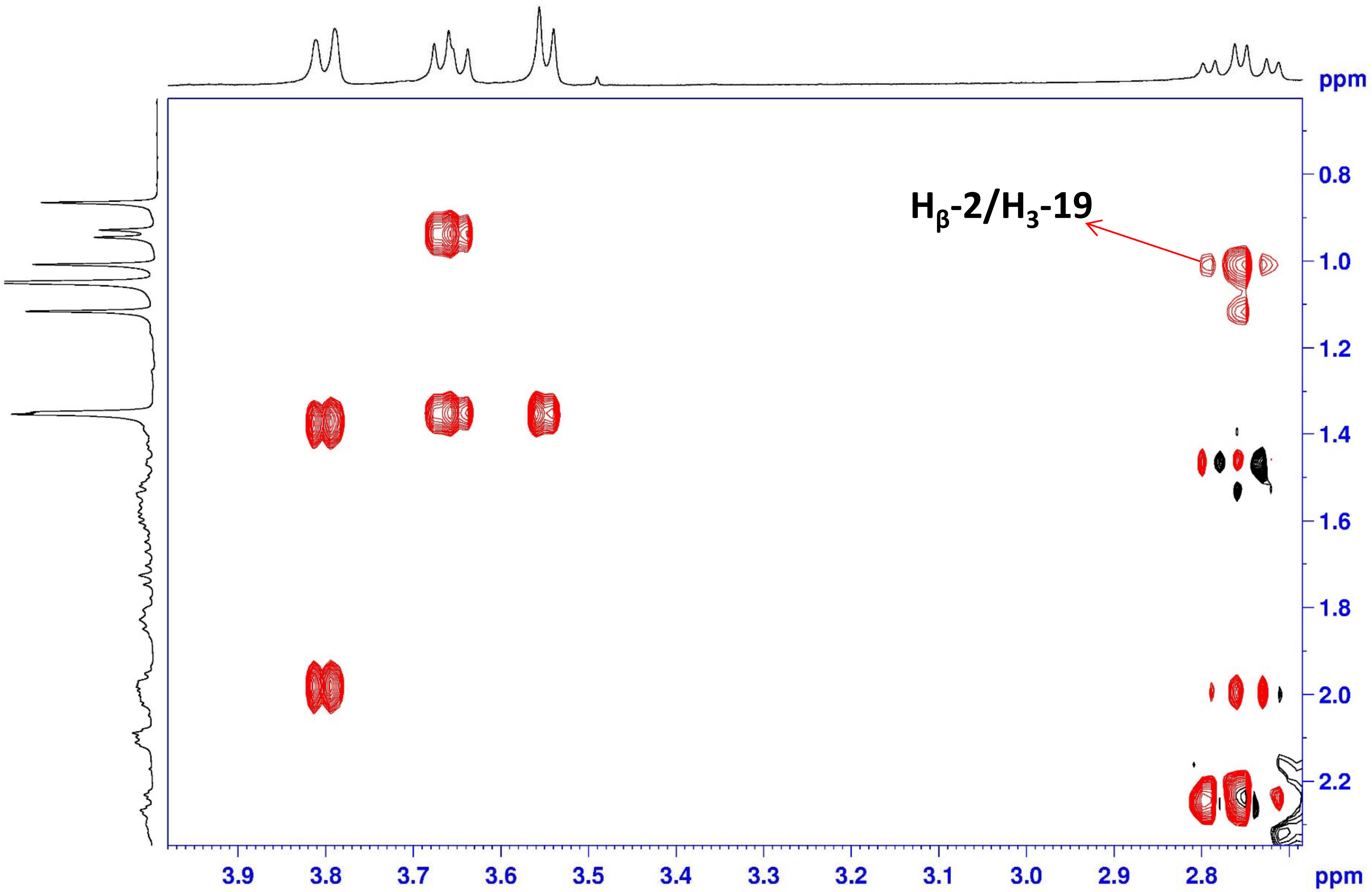

NOESY (400 MHz) spectrum of compound **1** in $CDCl_3$

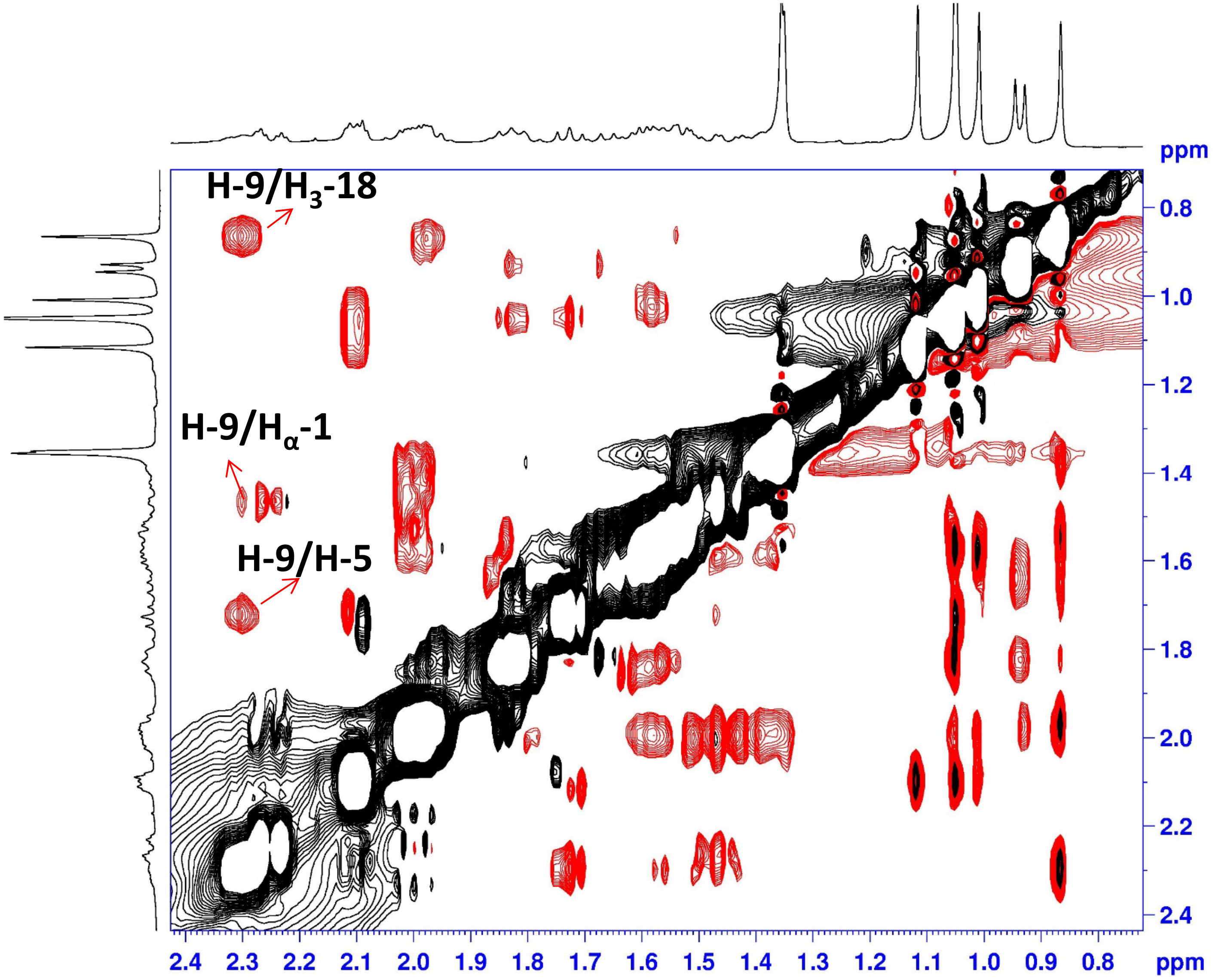

NOESY (400 MHz) spectrum of compound **1** in $CDCl_3$

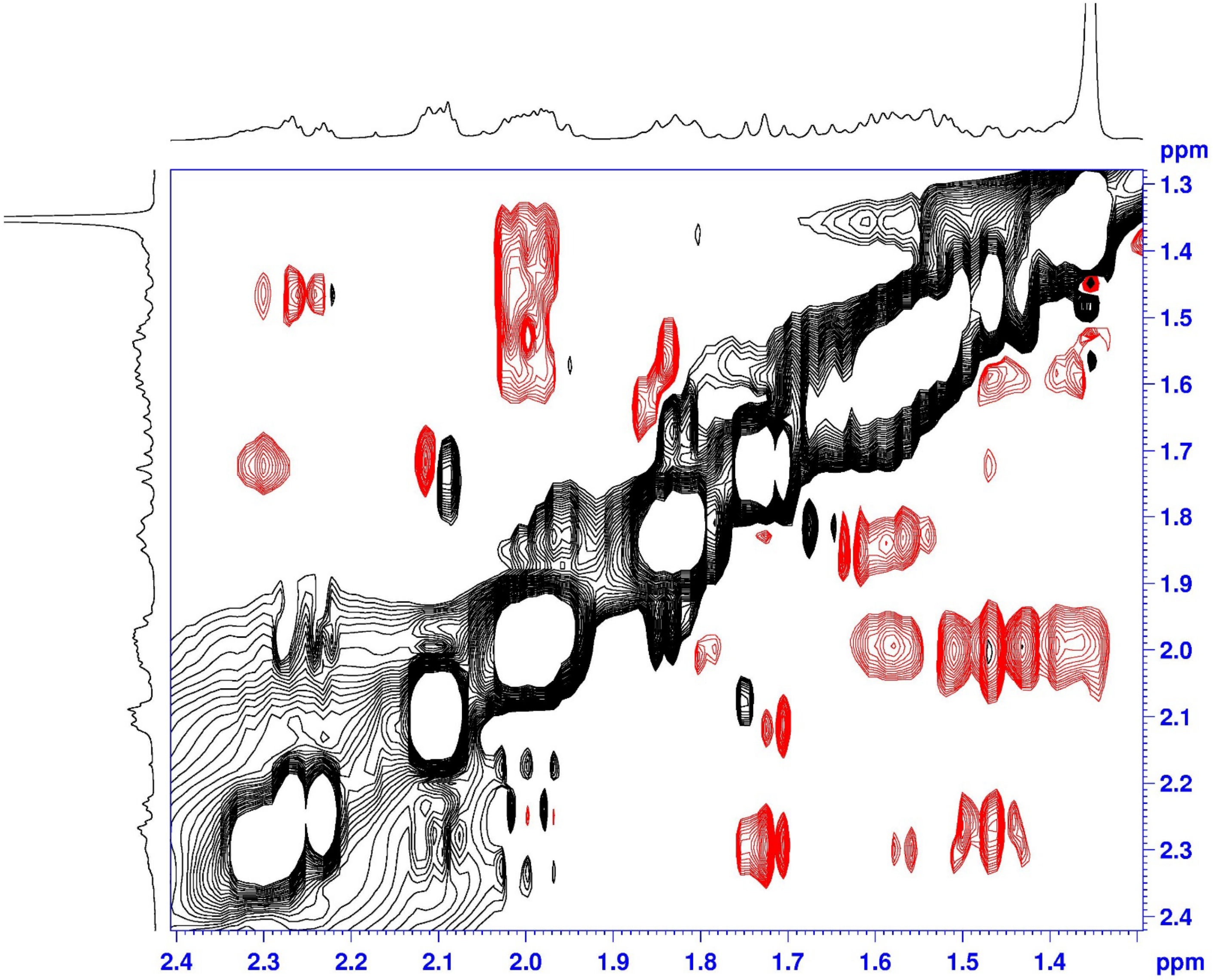

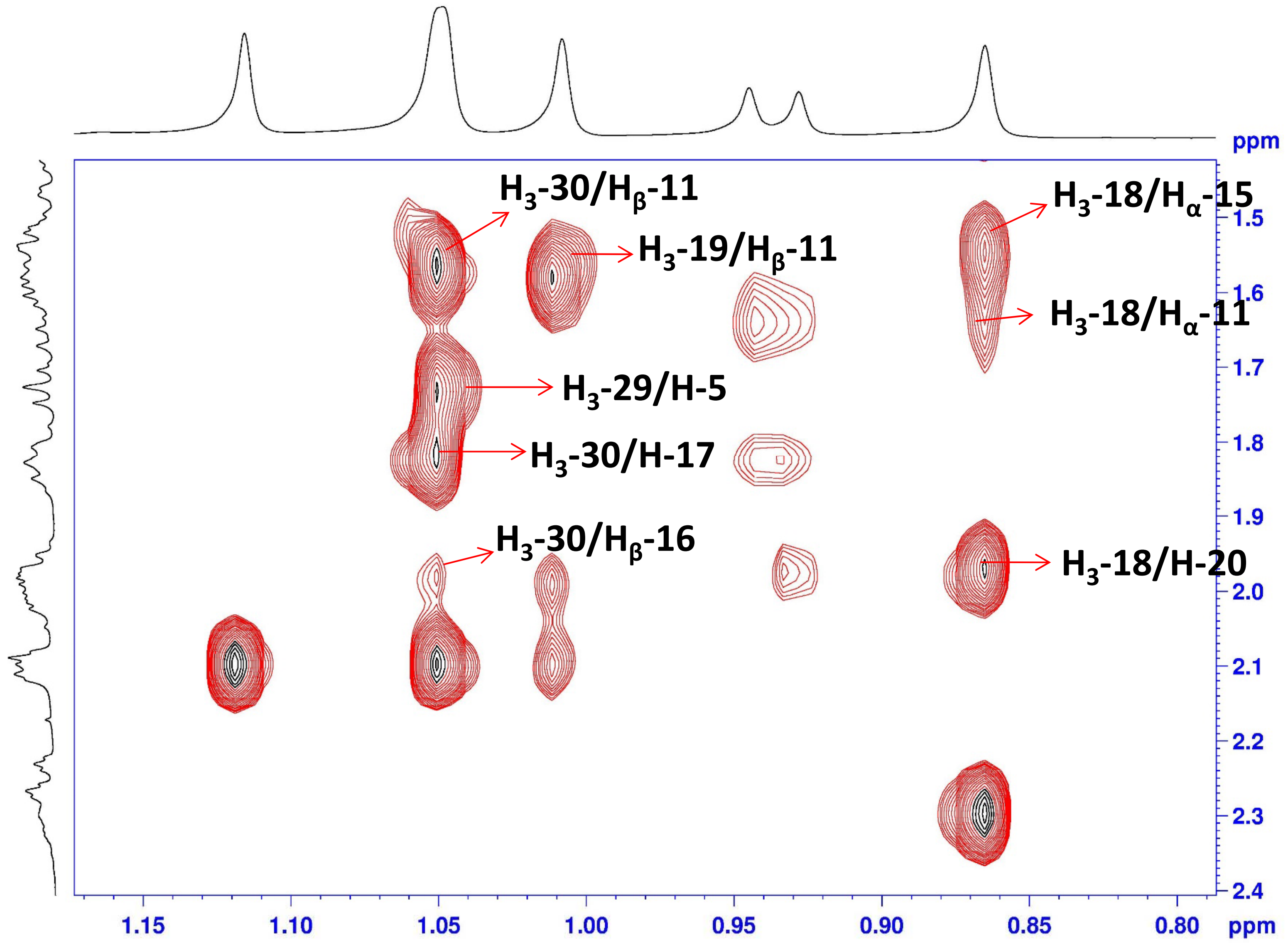

H_3-30/H_β-11
H_3-19/H_β-11
H_3-18/H_α-15
H_3-18/H_α-11
H_3-29/H-5
H_3-30/H-17
H_3-30/H_β-16
H_3-18/H-20
ppm
1.5
1.6
1.7
1.8
1.9
2.0
2.1
2.2
2.3
2.4
1.15
1.10
1.05
1.00
0.95
0.90
0.85
0.80
ppm

HR-ESIMS for compound **2**

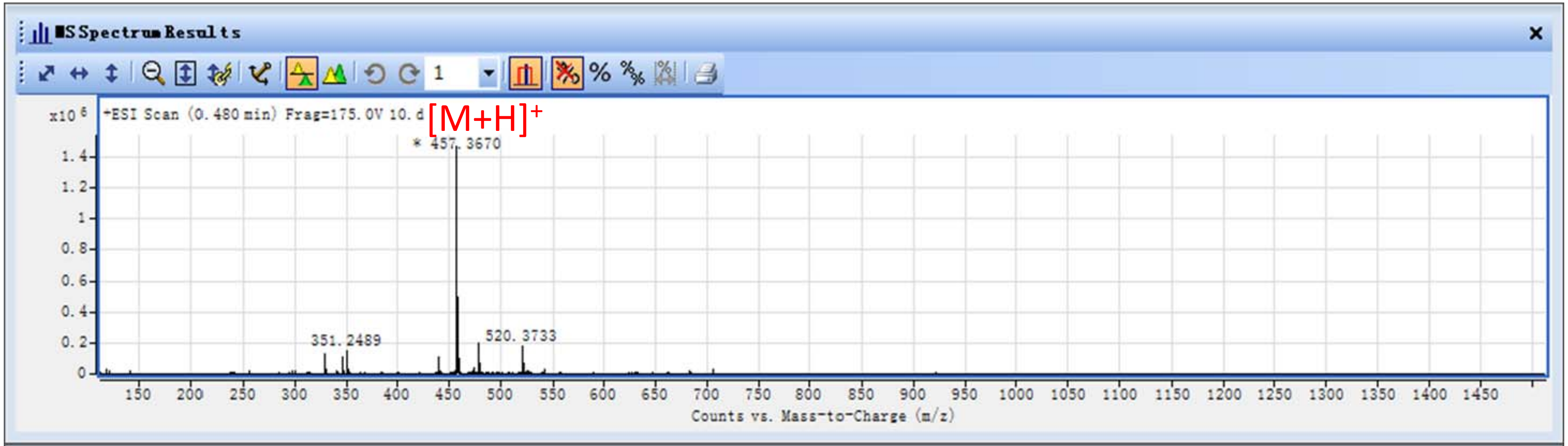

MS Formula Results: + Scan (0.480 min)

m/z	Ion	Formula	Abundance
457.367	(M+H)+	C30 H49 O3	1494171.9

Best	Formula (M)	Ion Formula	Score	Cross Score	Calc m/z	Diff (ppm)	Mass Match	Abund Match	Spacing Match
☑	C30 H48 O3	C30 H49 O3	95.4		457.3676	1.47	97.86	99.01	86.12

Isotope	Abund Sum%	Calc Abund Sum%	m/z	Calc m/z	Diff (ppm)
1	70.36	71.53	457.367	457.3676	1.31
2	24.32	23.69	458.3686	458.371	5.21
3	4.57	4.24	459.3715	459.3742	5.87
4	0.75	0.54	460.3715	460.3772	12.39

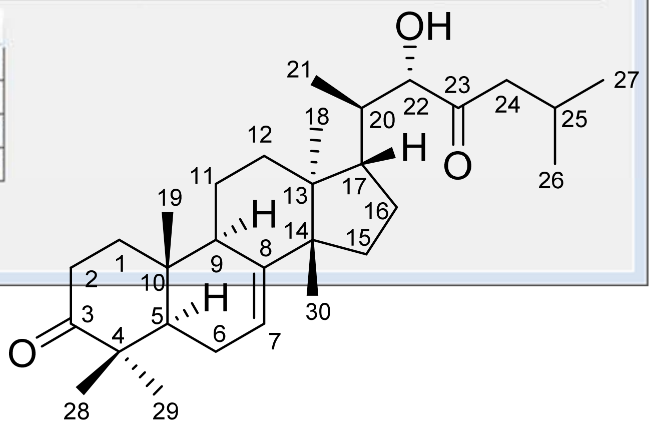

^{1}H NMR (400 MHz) spectrum of compound **2** in $CDCl_3$

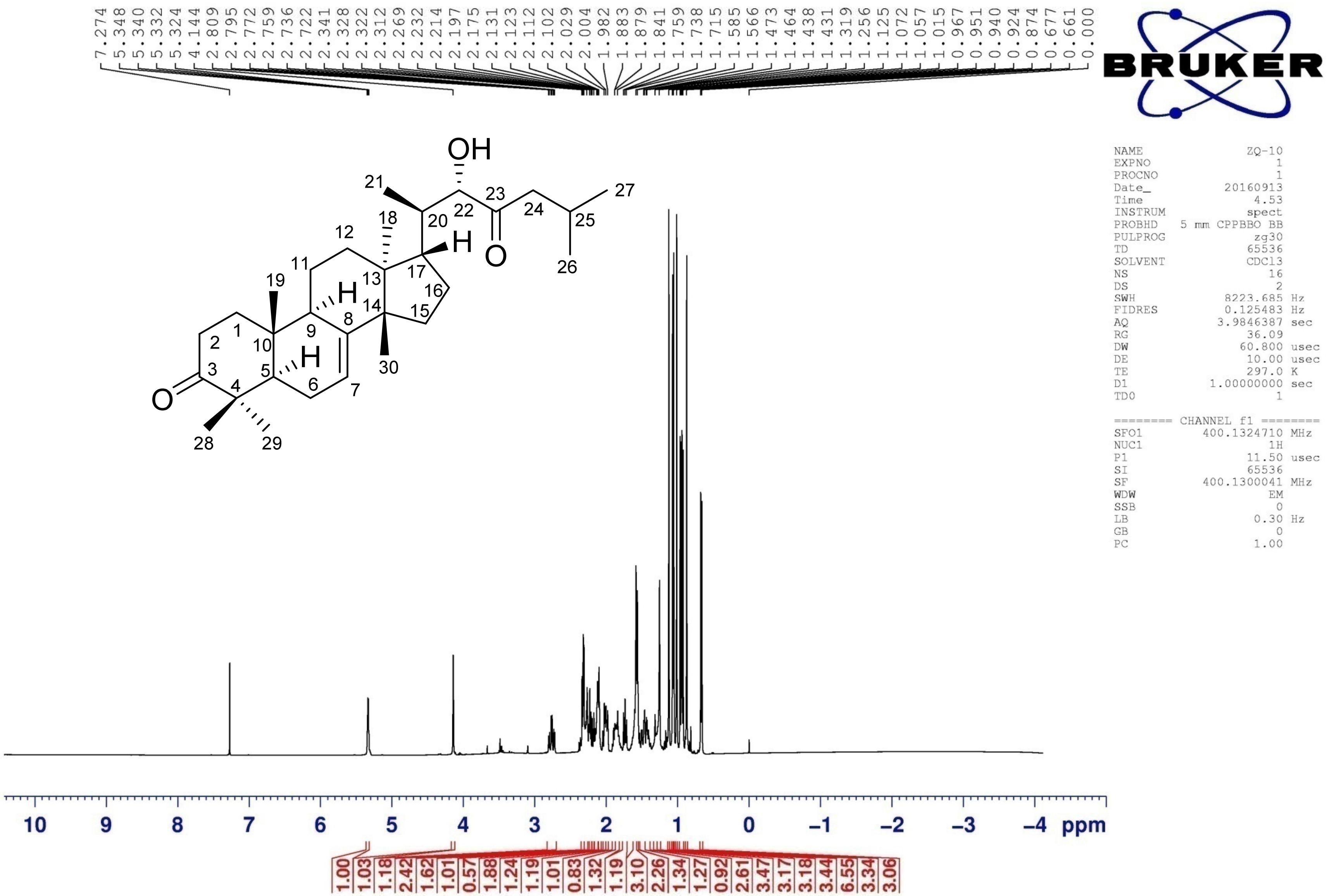

^{1}H NMR (400 MHz) spectrum of compound **2** in $CDCl_3$

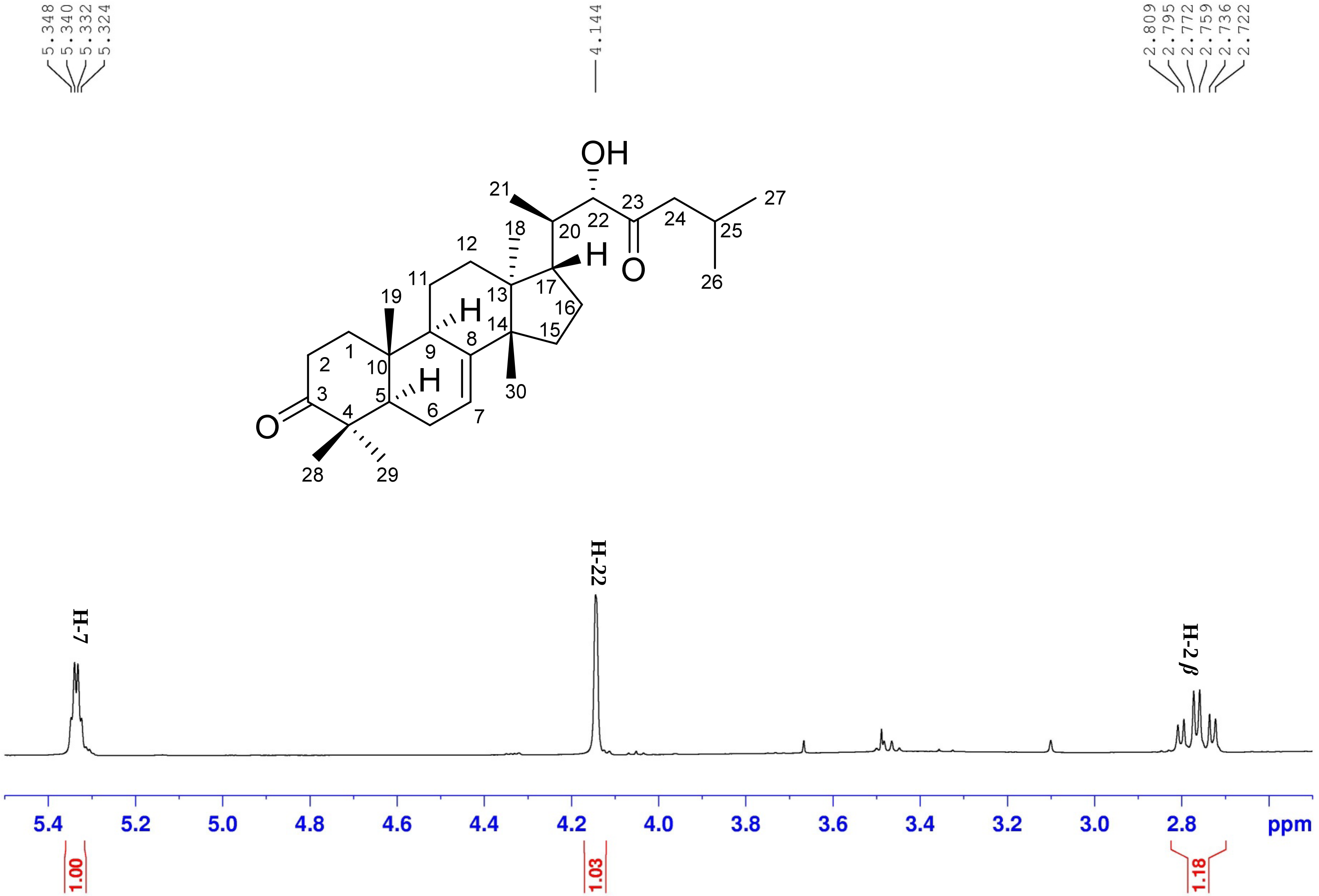

^{1}H NMR (400 MHz) spectrum of compound **2** in $CDCl_3$

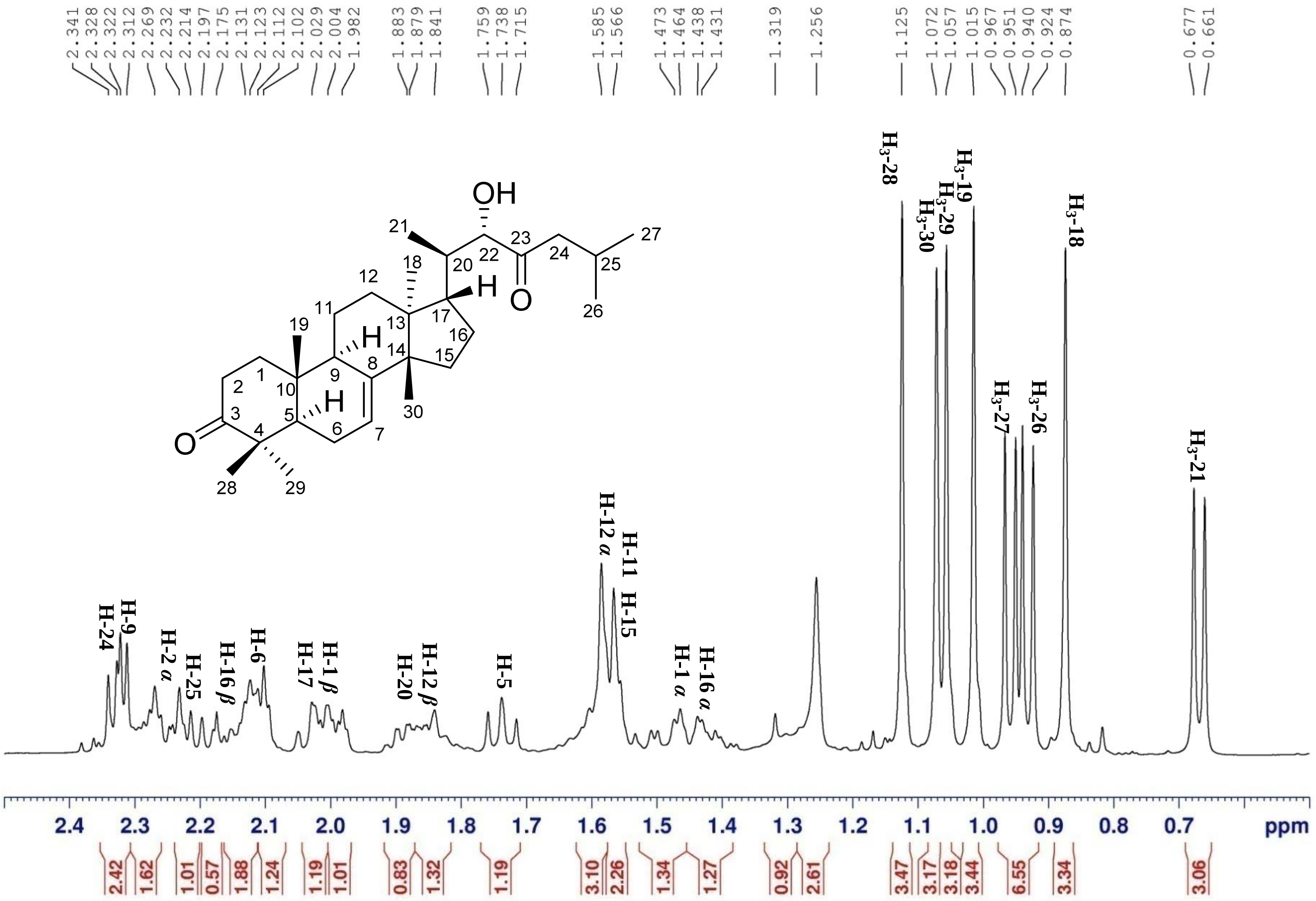

^{13}C NMR (100 MHz) spectrum of compound **2** in $CDCl_3$

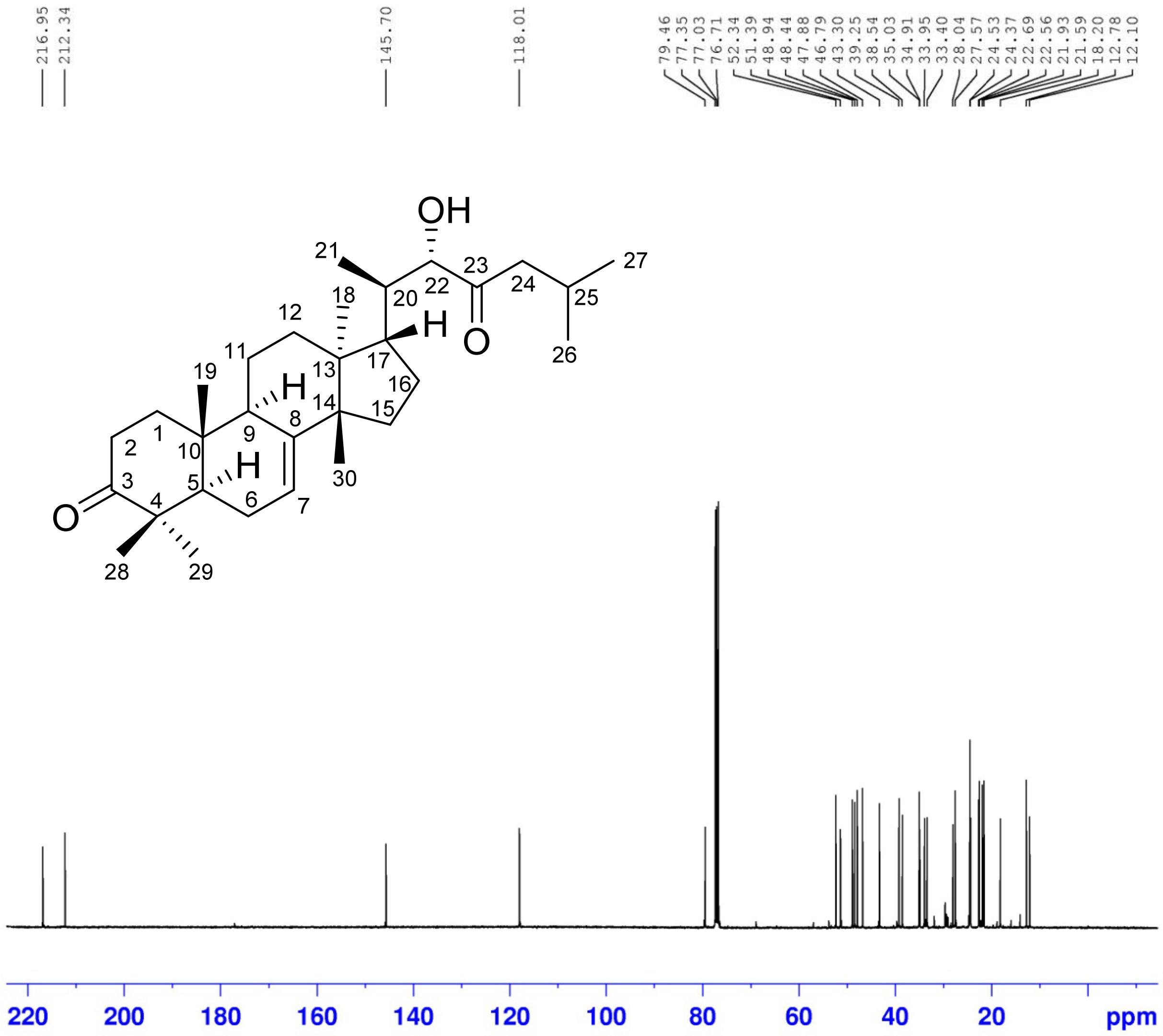

NAME	ZQ-10-1	
EXPNO	2	
PROCNO	1	
Date_	20160913	
Time	5.53	
INSTRUM	spect	
PROBHD	5 mm CPPBBO BB	
PULPROG	zgpg30	
TD	65536	
SOLVENT	CDCl3	
NS	1024	
DS	4	
SWH	24038.461	Hz
FIDRES	0.366798	Hz
AQ	1.3631988	sec
RG	73.92	
DW	20.800	usec
DE	18.00	usec
TE	297.0	K
D1	2.00000000	sec
D11	0.03000000	sec
TD0	1	

======== CHANNEL f1 ========

SFO1	100.6233324	MHz
NUC1	13C	
P1	10.00	usec
SI	32768	
SF	100.6127698	MHz
WDW	EM	
SSB	0	
LB	1.00	Hz
GB	0	
PC	1.40	

^{13}C NMR (100 MHz) spectrum of compound **2** in $CDCl_3$

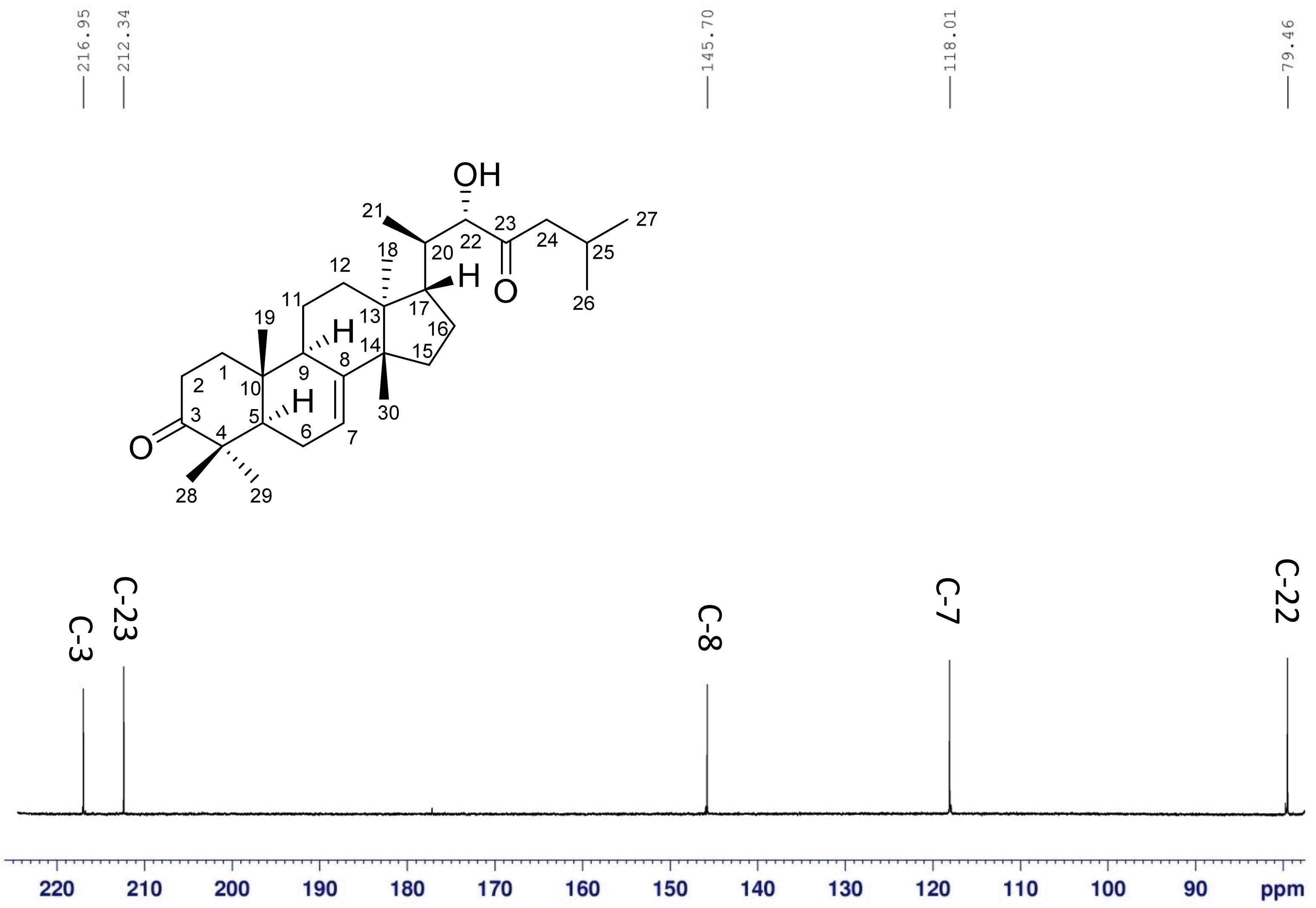

^{13}C NMR (100 MHz) spectrum of compound **2** in $CDCl_3$

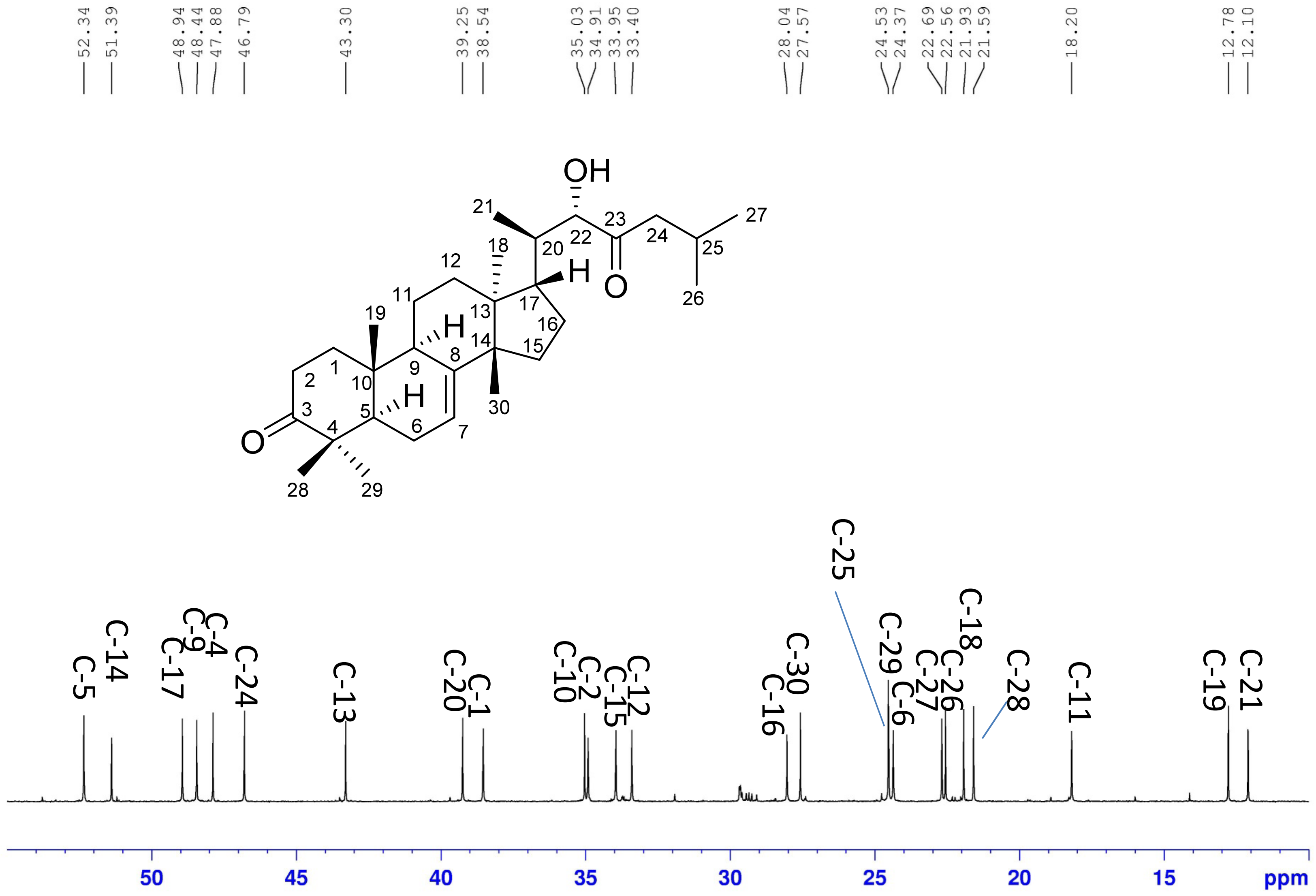

DEPT135 (100 MHz) spectrum of compound **2** in $CDCl_3$

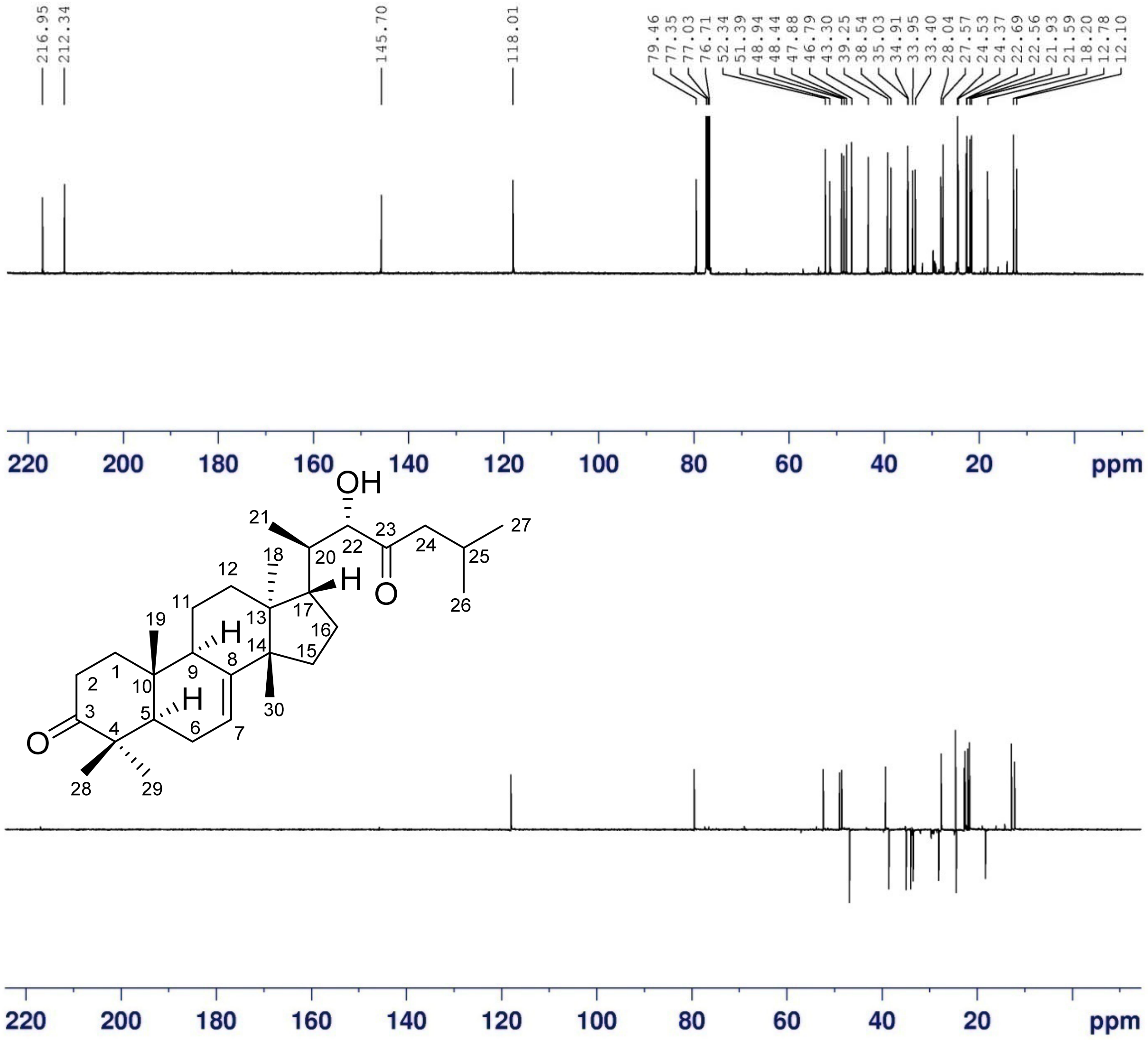

NAME	ZQ-10-1
EXPNO	3
PROCNO	1
Date_	20160913
Time	6.11
INSTRUM	spect
PROBHD	5 mm CPPBBO BB
PULPROG	deptsp135
TD	65536
SOLVENT	CDCl3
NS	300
DS	4
SWH	24038.461 Hz
FIDRES	0.366798 Hz
AQ	1.3631988 sec
RG	91.64
DW	20.800 usec
DE	18.00 usec
TE	297.0 K
CNST2	145.0000000
D1	2.00000000 sec
D2	0.00344828 sec
D12	0.00002000 sec
TD0	1
======== CHANNEL f1 ========	
SFO1	100.6233324 MHz
NUC1	13C
P1	10.00 usec
P13	2000.00 usec
SI	32768
SF	100.6127698 MHz
WDW	EM
SSB	0
LB	1.00 Hz
GB	0
PC	1.40

DEPT135 (100 MHz) spectrum of compound **2** in $CDCl_3$

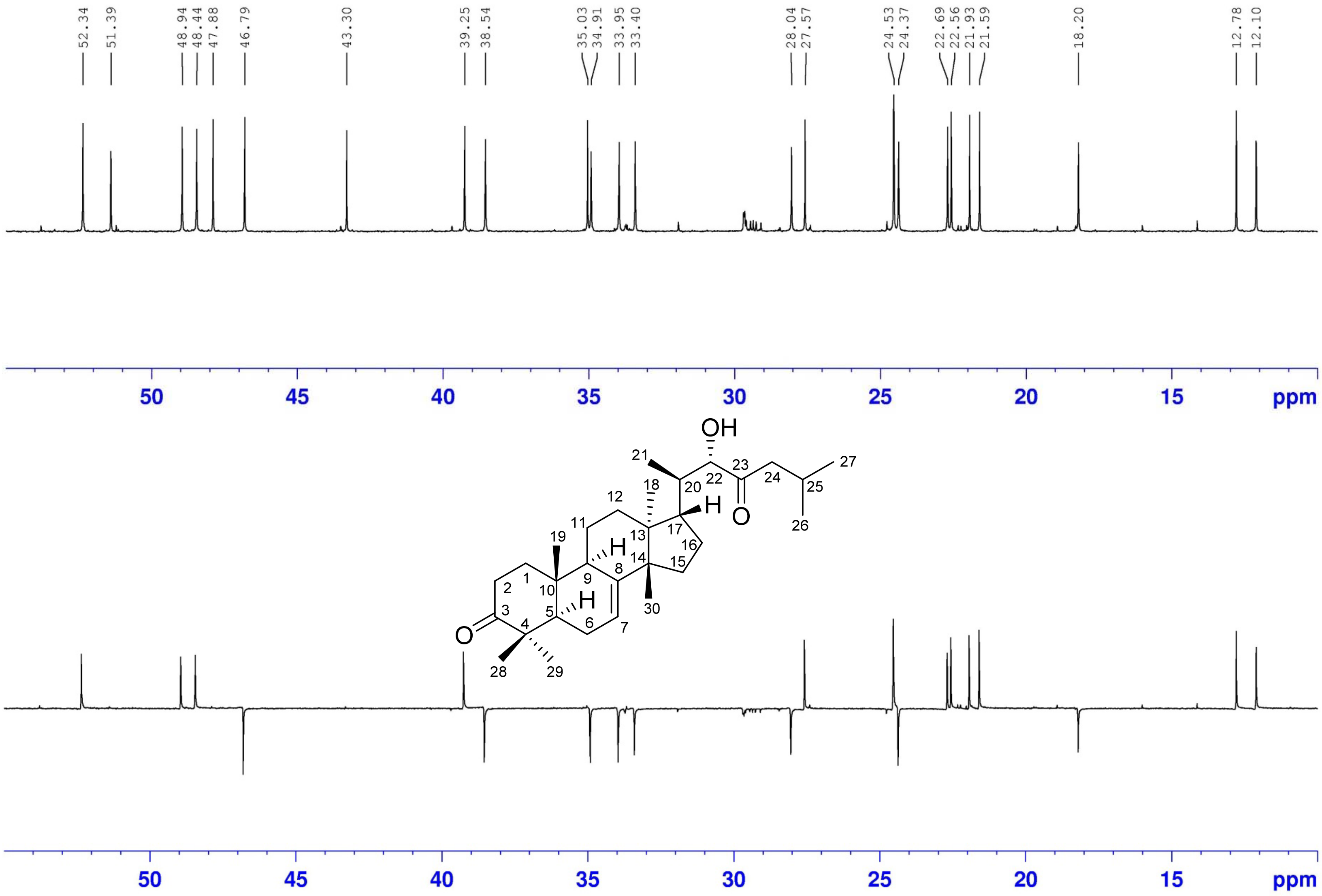

^1H-^1H COSY (400 MHz) spectrum of compound **2** in $CDCl_3$

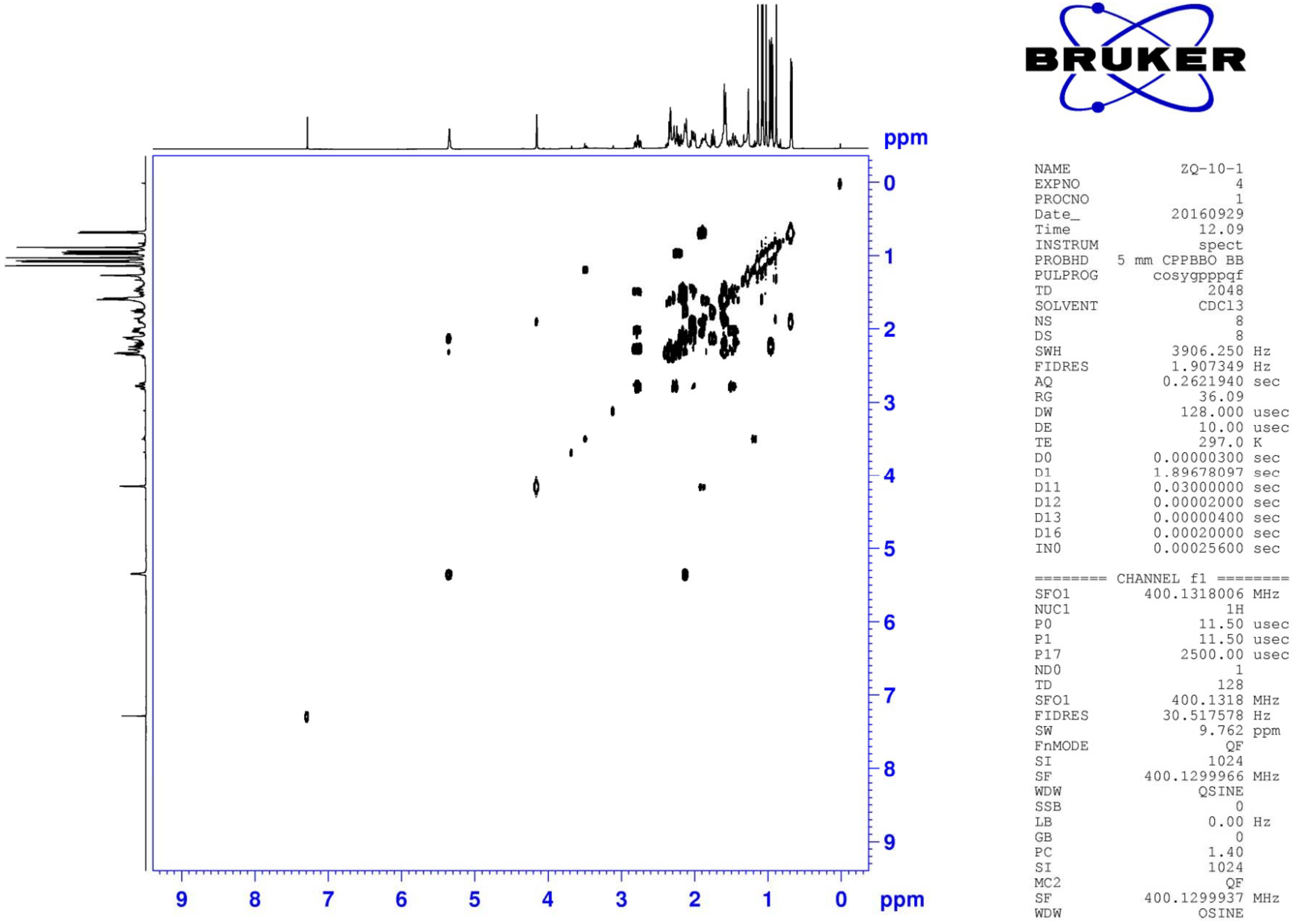

^{1}H-^{1}H COSY (400 MHz) spectrum of compound **2** in $CDCl_3$

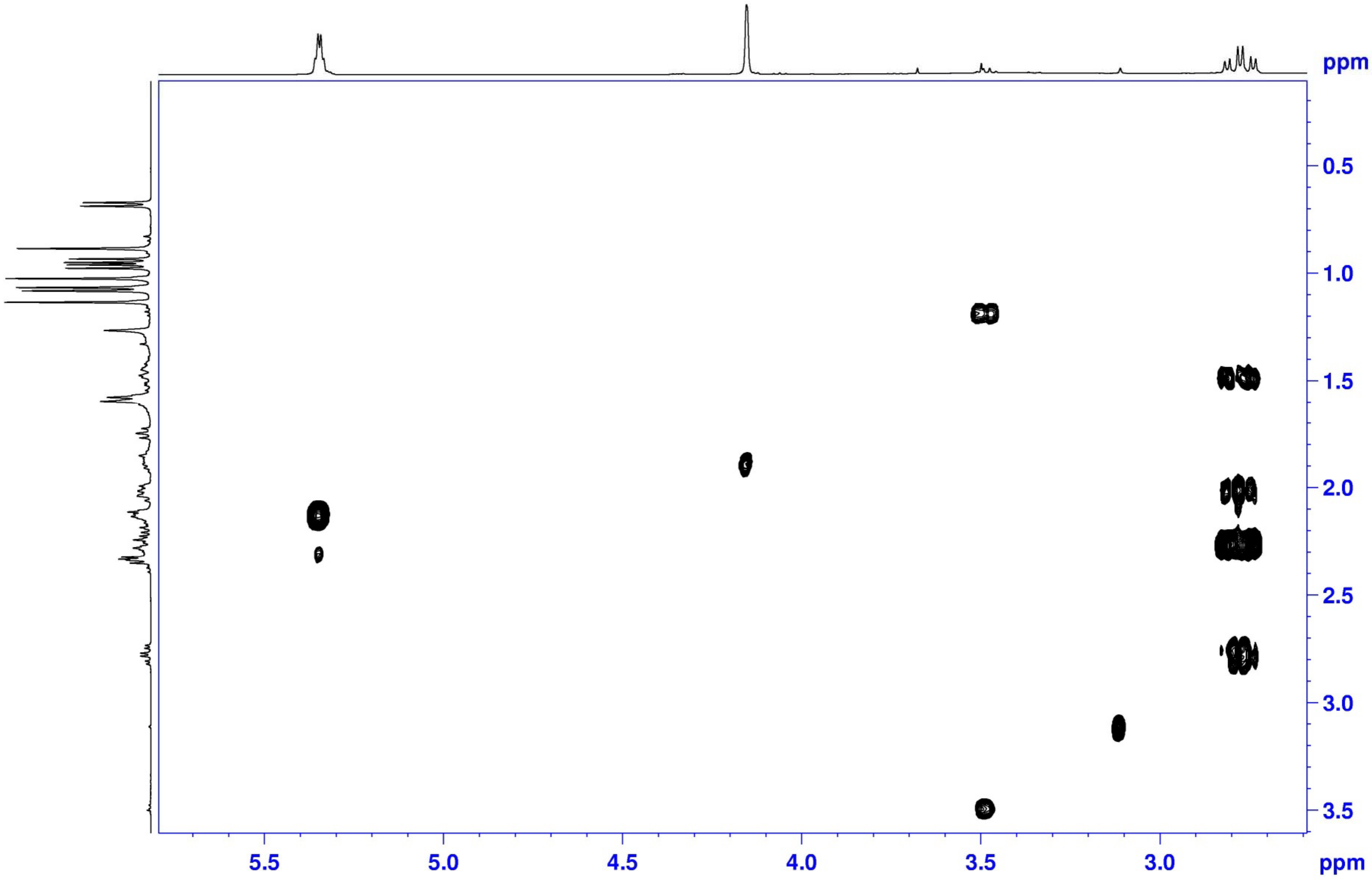

^1H-^1H COSY (400 MHz) spectrum of compound **2** in $CDCl_3$

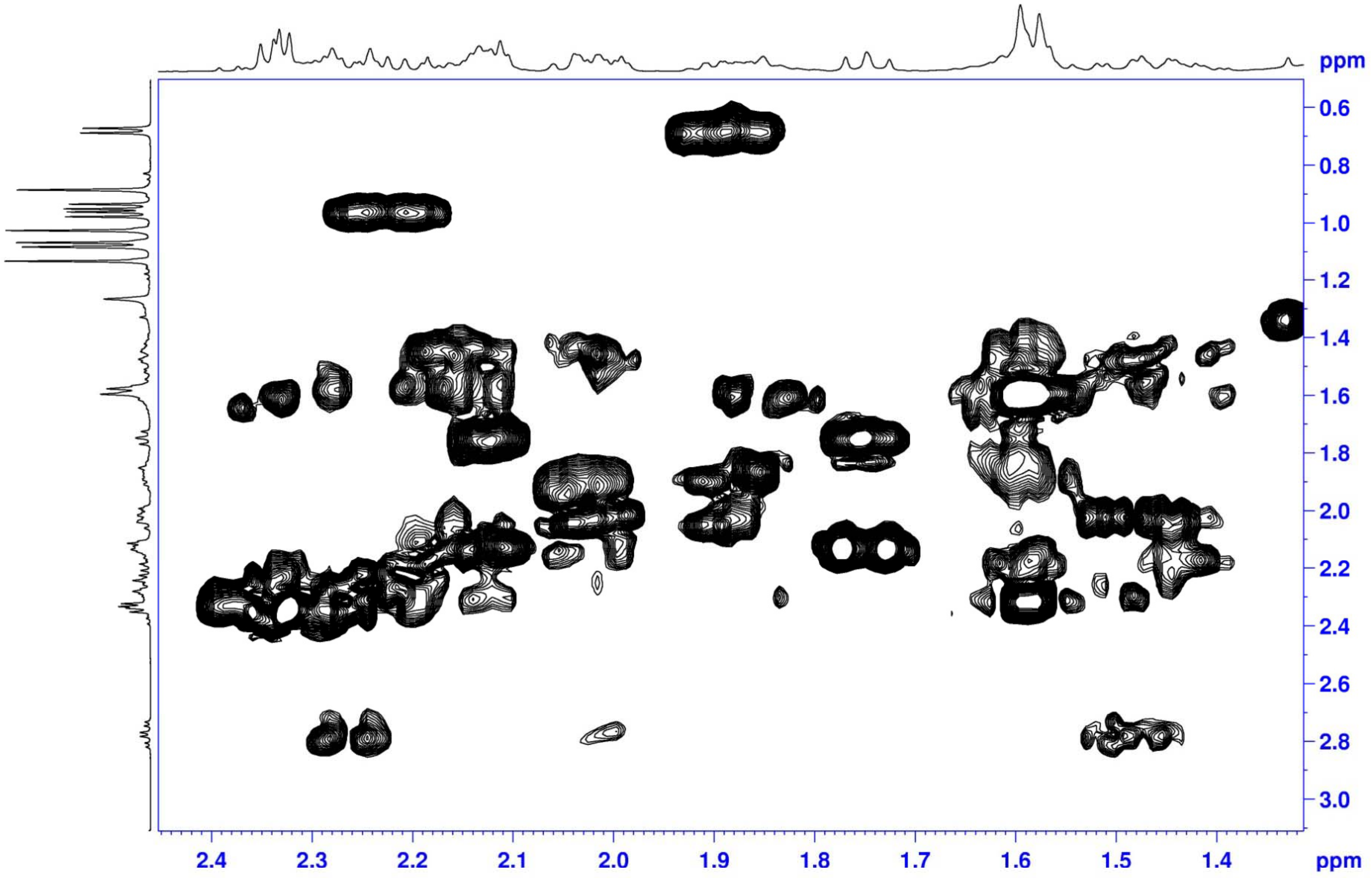

HSQC (400 MHz) spectrum of compound **2** in $CDCl_3$

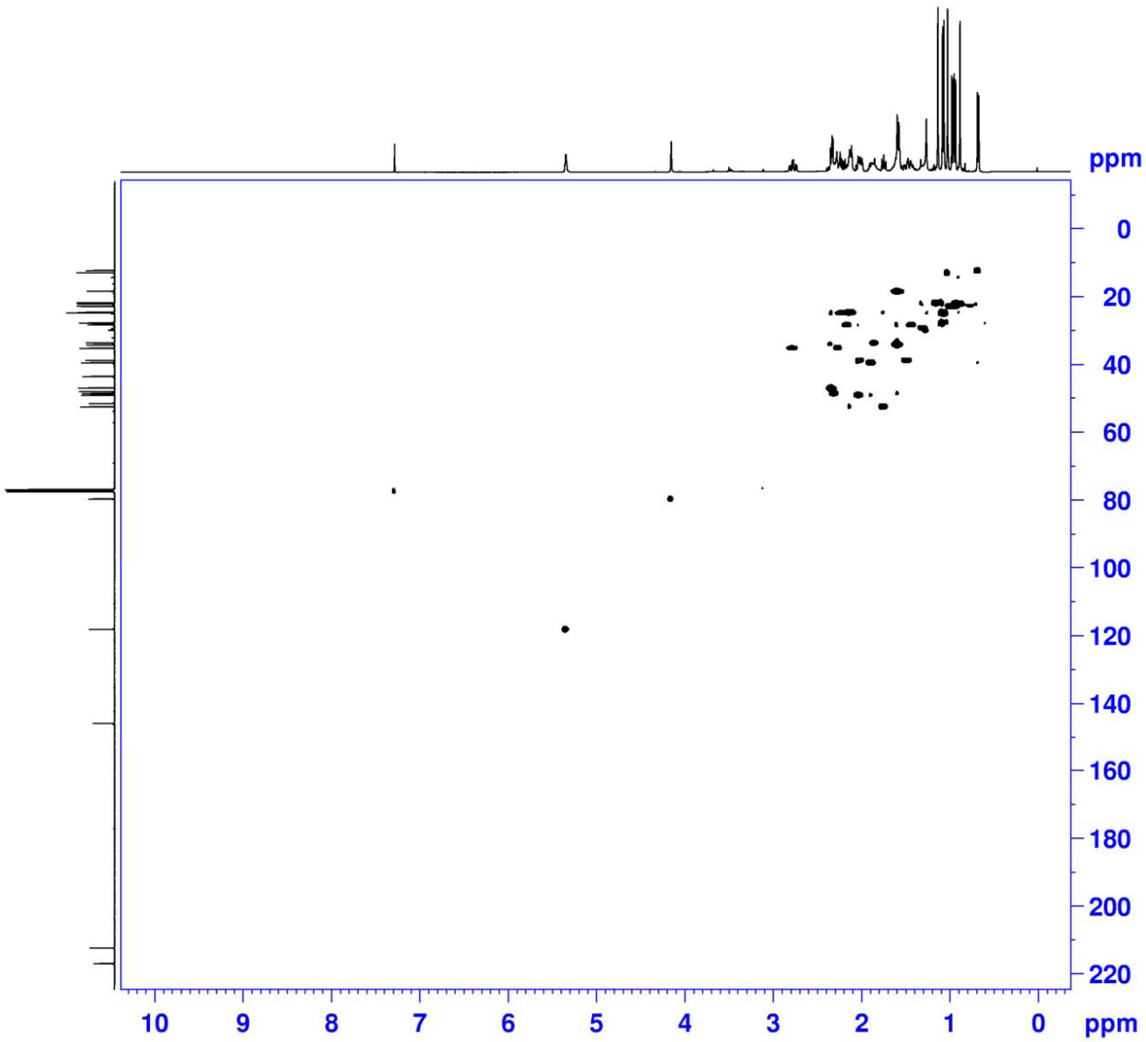


```
NAME              ZQ-10-1
EXPNO                   5
PROCNO                  1
Date_            20160929
Time                12.48
INSTRUM             spect
PROBHD   5 mm CPPBBO BB
PULPROG       hsqcetgpsi2
TD                   1024
SOLVENT             CDCl3
NS                     16
DS                     16
SWH              4302.926 Hz
FIDRES           4.202076 Hz
AQ              0.1190388 sec
RG                  208.5
DW                116.200 usec
DE                  10.00 usec
TE                  297.0 K
CNST2         145.0000000
D0             0.00000300 sec
D1             1.46497905 sec
D4             0.00172414 sec
D11            0.03000000 sec
D16            0.00020000 sec
D24            0.00086207 sec
IN0            0.00002080 sec
ZGOPTNS

======== CHANNEL f1 ========
SFO1          400.1320007 MHz
NUC1                   1H
P1                  11.50 usec
P2                  23.00 usec
P28                  0.00 usec
ND0                     2
TD                    256
SFO1             100.6233 MHz
FIDRES          93.900238 Hz
SW                238.896 ppm
FnMODE       Echo-Antiecho
SI                   1024
SF            400.1299963 MHz
WDW                 QSINE
SSB                     2
LB                   0.00 Hz
GB                      0
PC                   1.40
SI                   1024
MC2          echo-antiecho
SF            100.6127561 MHz
WDW                 QSINE
SSB                     2
LB                   0.00 Hz
```

HSQC (400 MHz) spectrum of compound **2** in $CDCl_3$

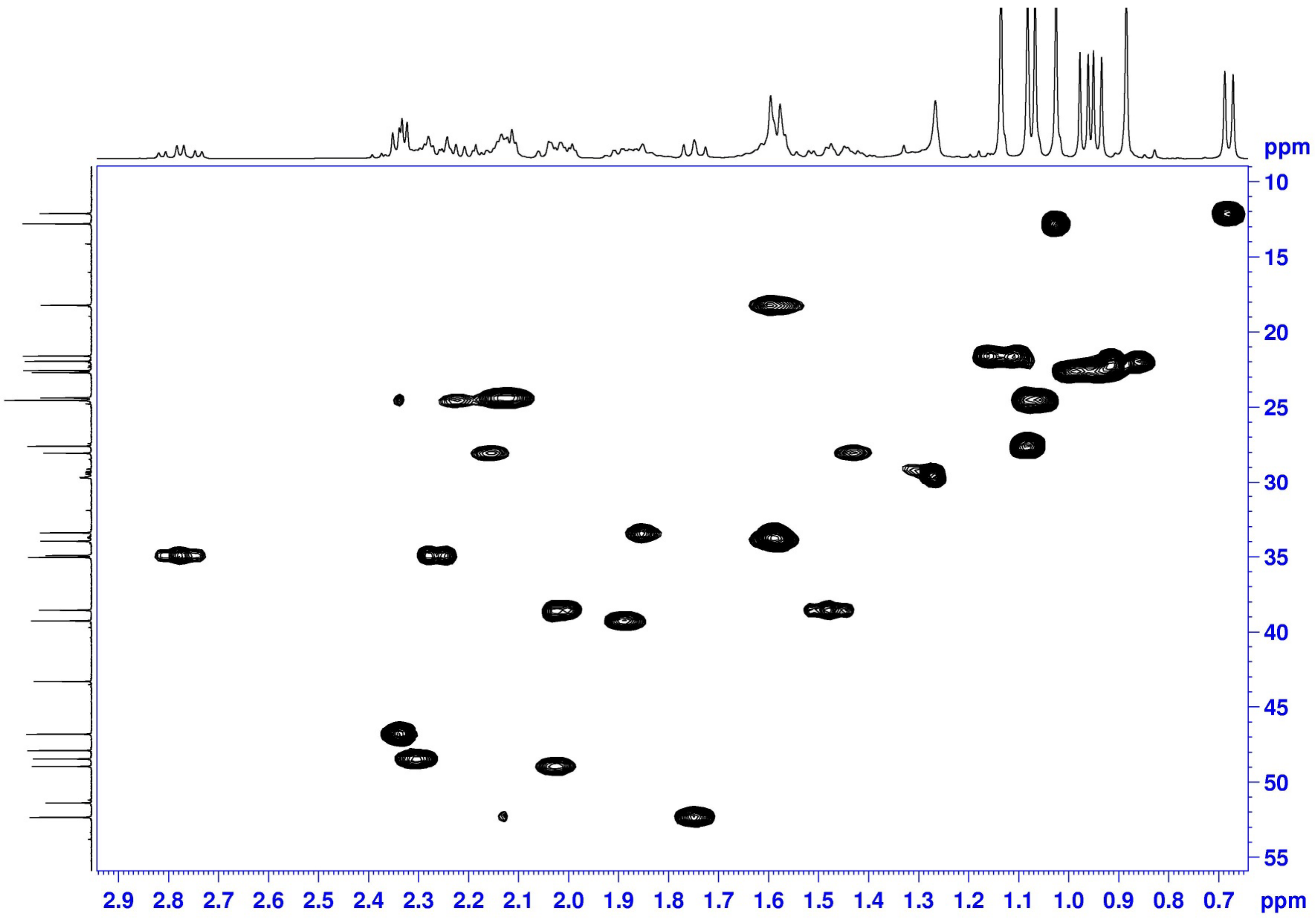

HMBC (400 MHz) spectrum of compound **2** in $CDCl_3$

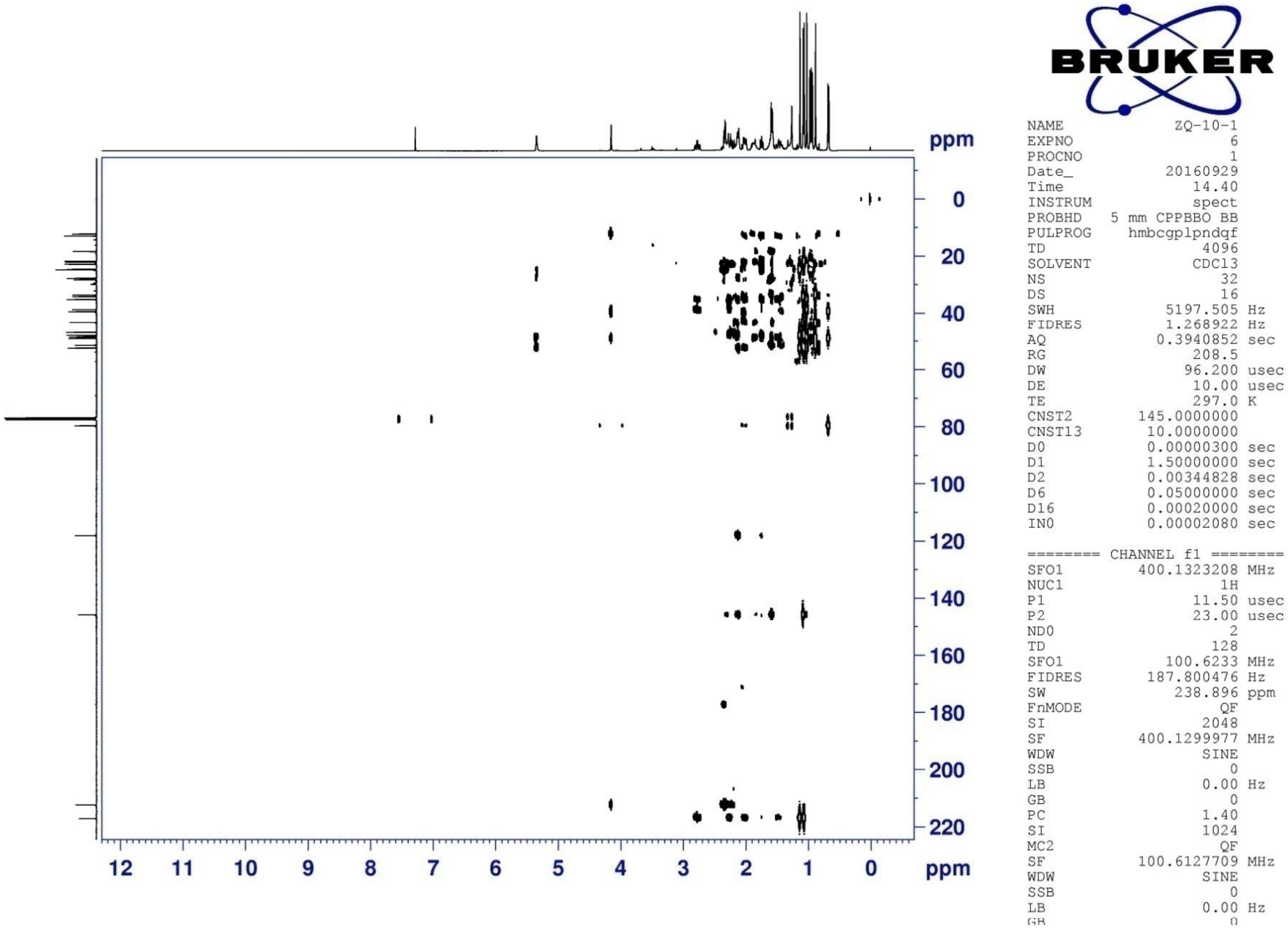

HMBC (400 MHz) spectrum of compound **2** in $CDCl_3$

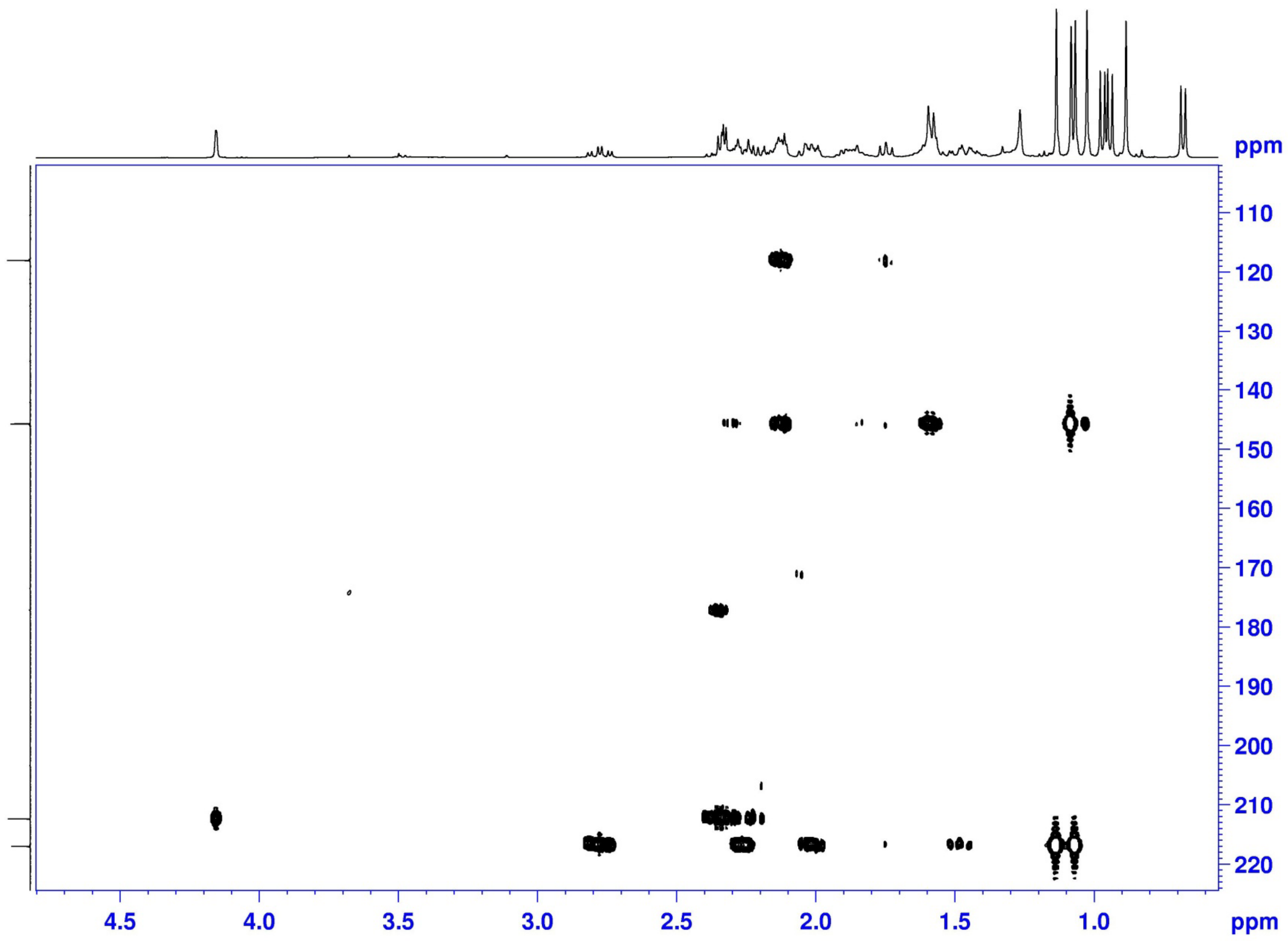

HMBC (400 MHz) spectrum of compound **2** in $CDCl_3$

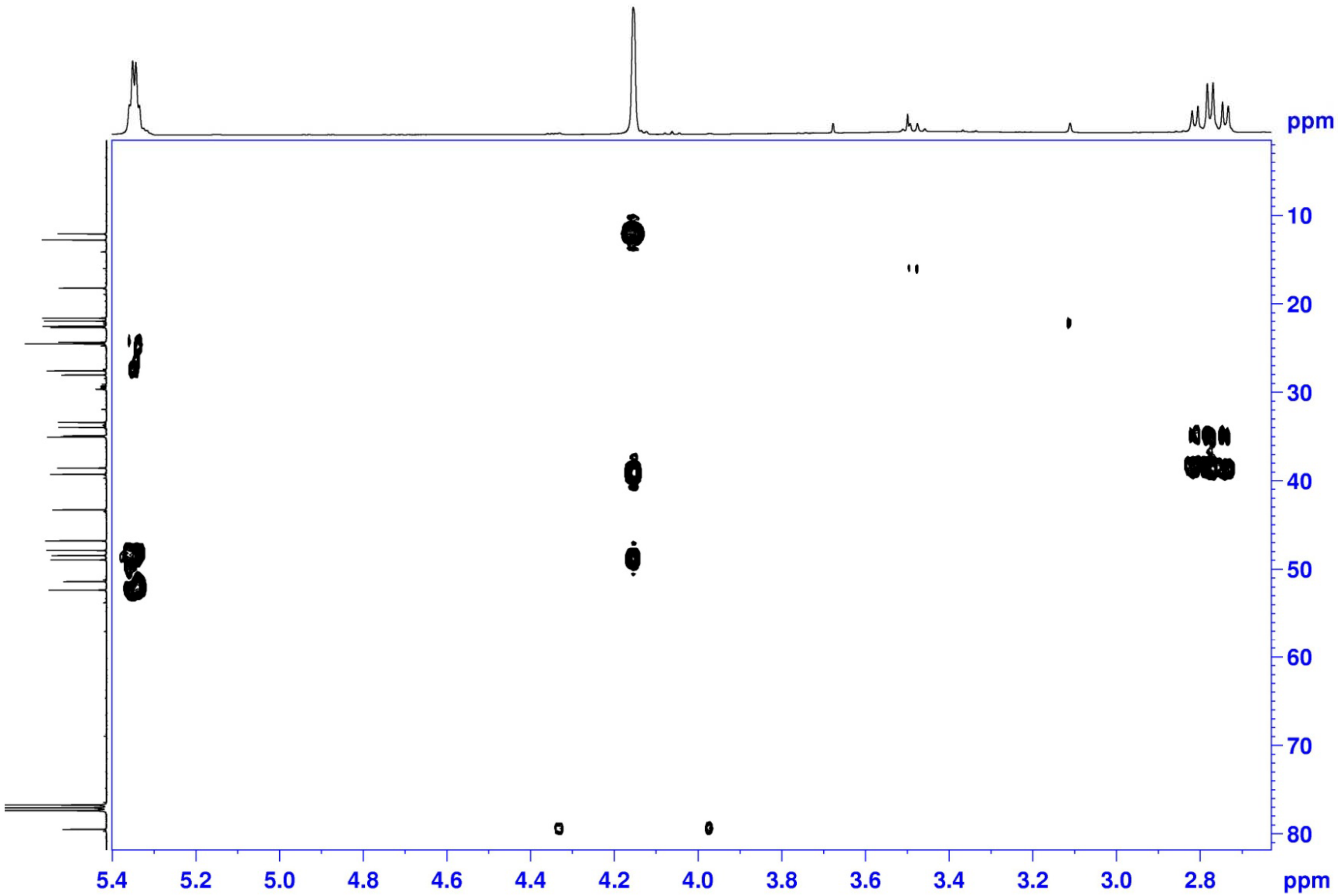

HMBC (400 MHz) spectrum of compound **2** in $CDCl_3$

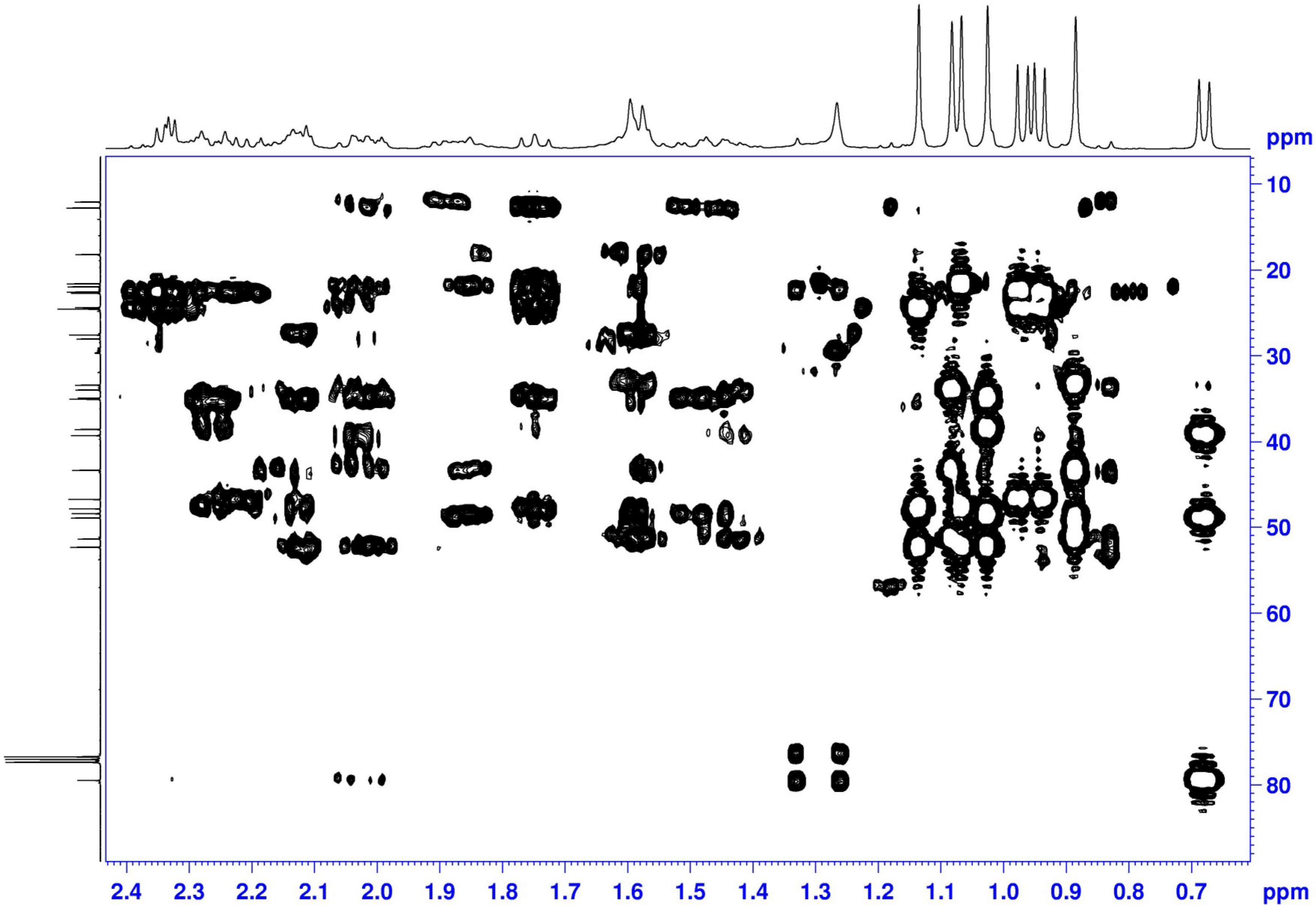

HMBC (400 MHz) spectrum of compound **2** in $CDCl_3$

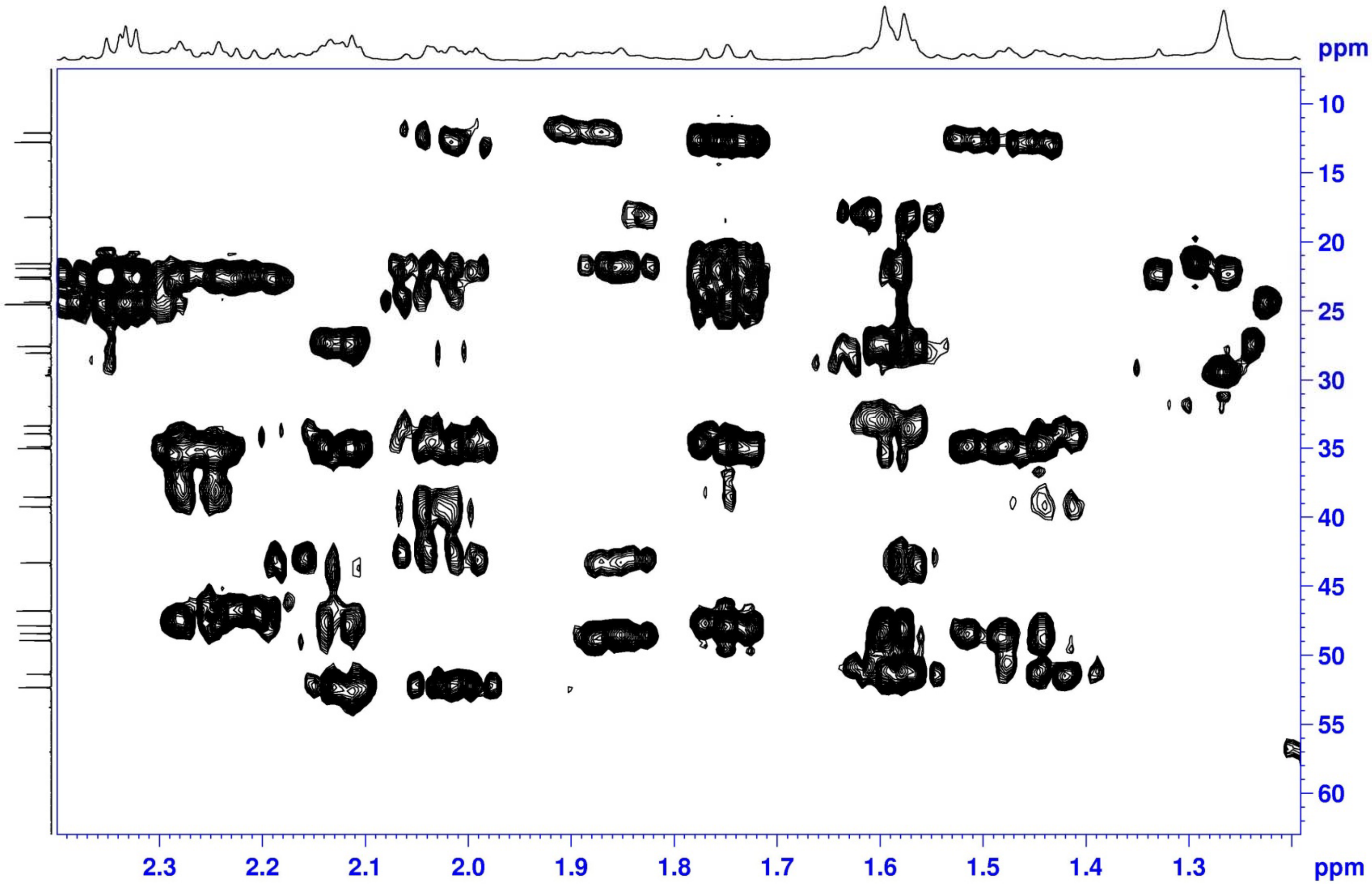

HMBC (400 MHz) spectrum of compound **2** in $CDCl_3$

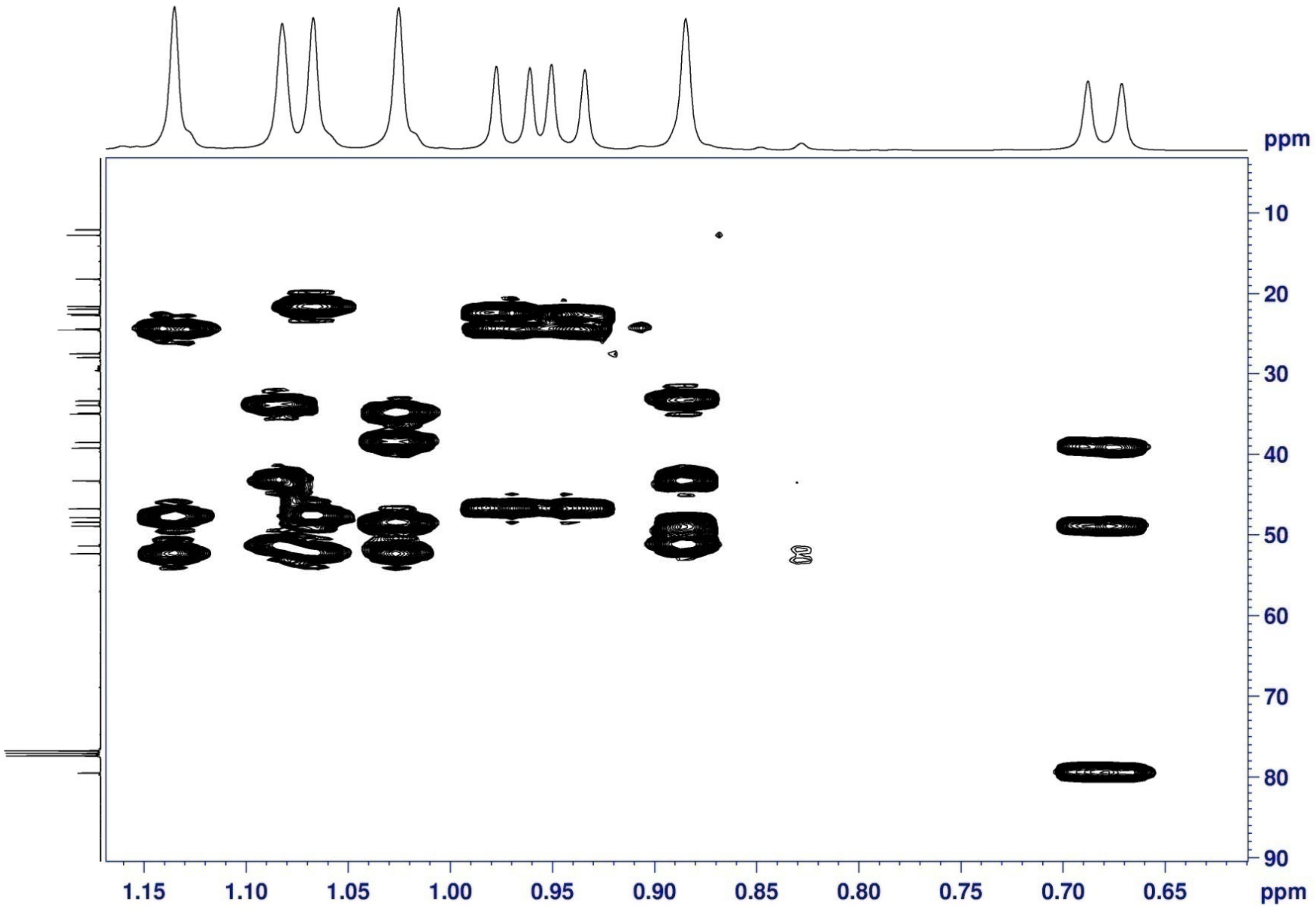

NOESY (400 MHz) spectrum of compound **2** in $CDCl_3$

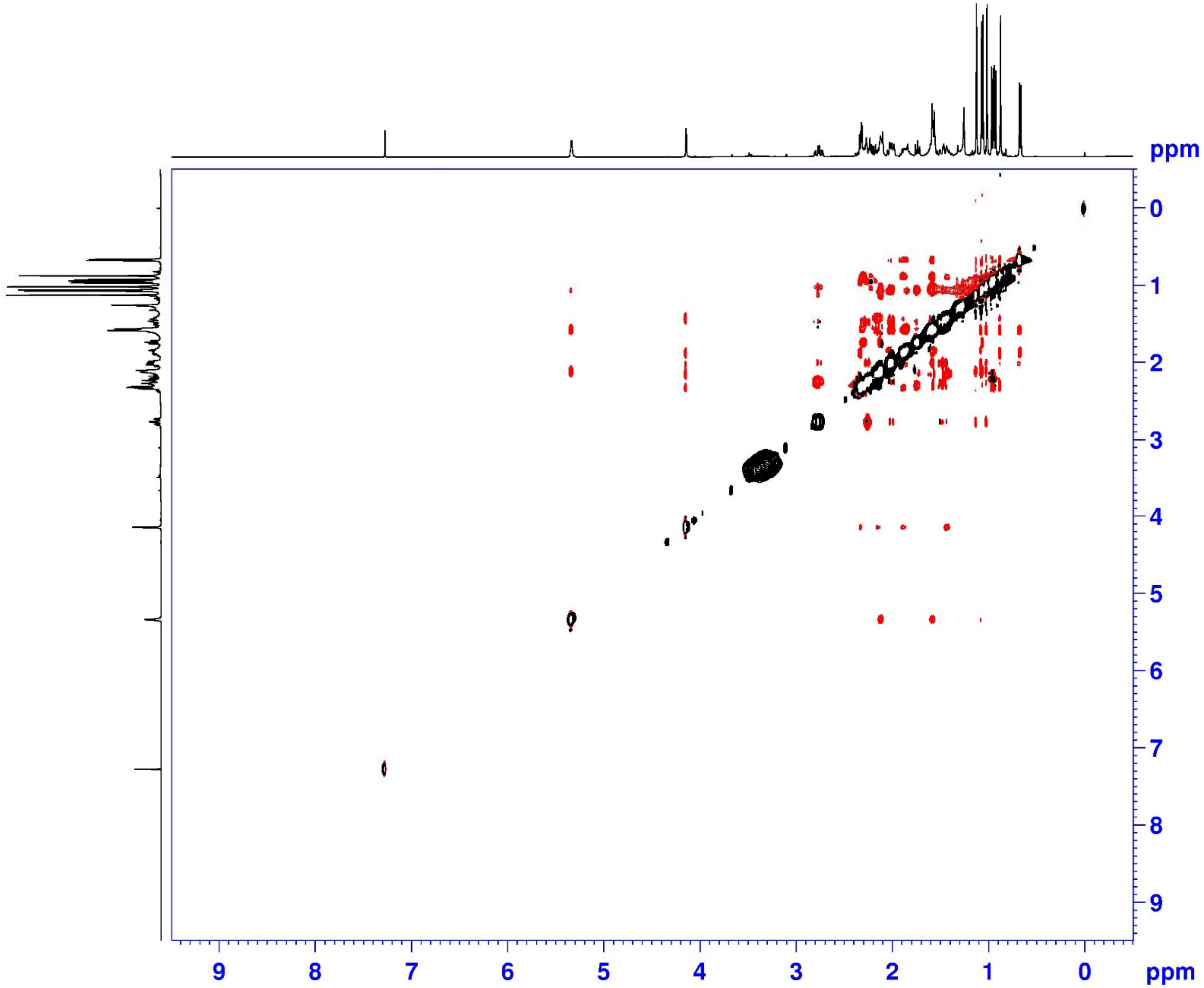

NOESY (400 MHz) spectrum of compound **2** in $CDCl_3$

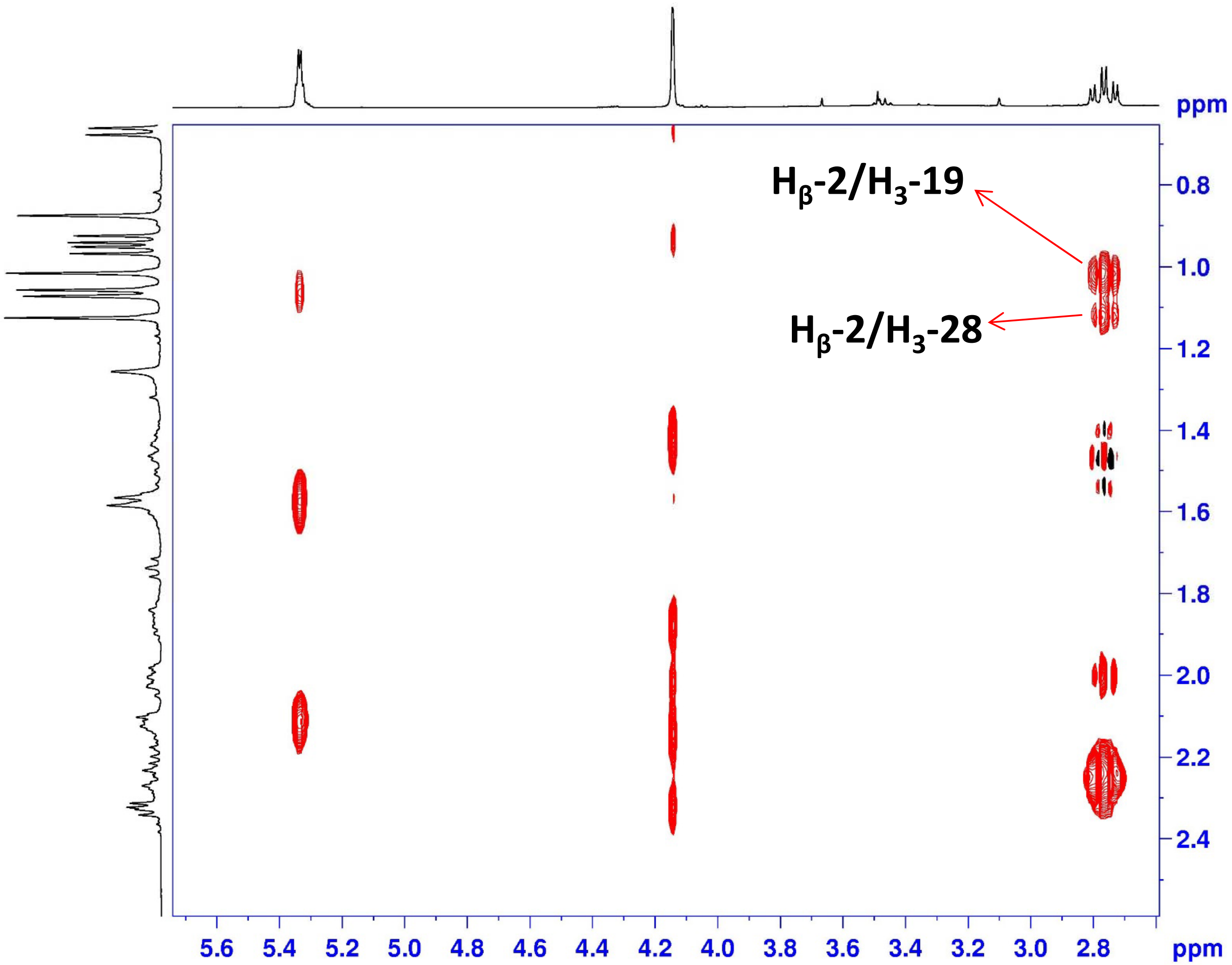

NOESY (400 MHz) spectrum of compound **2** in $CDCl_3$

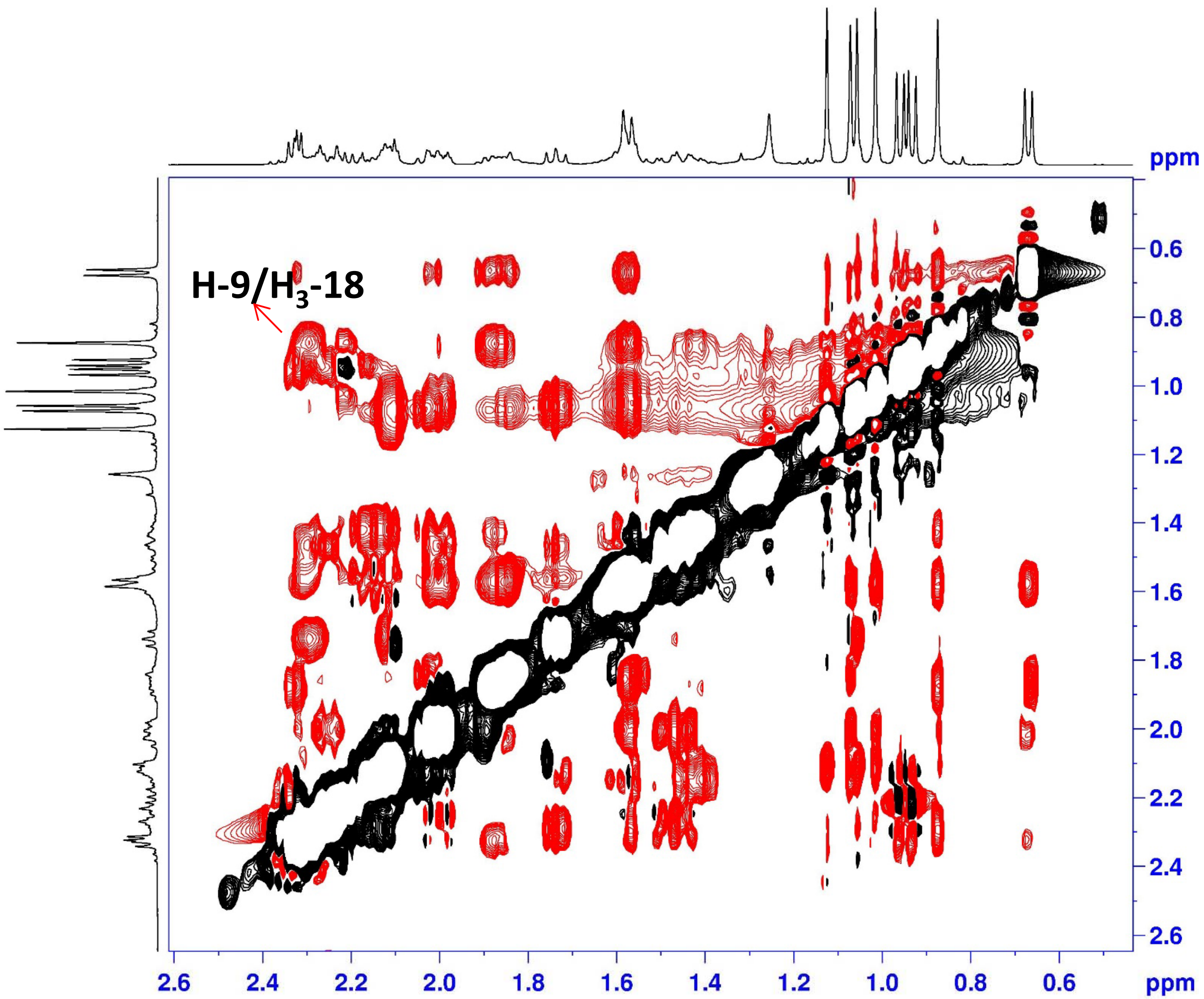

NOESY (400 MHz) spectrum of compound **2** in $CDCl_3$

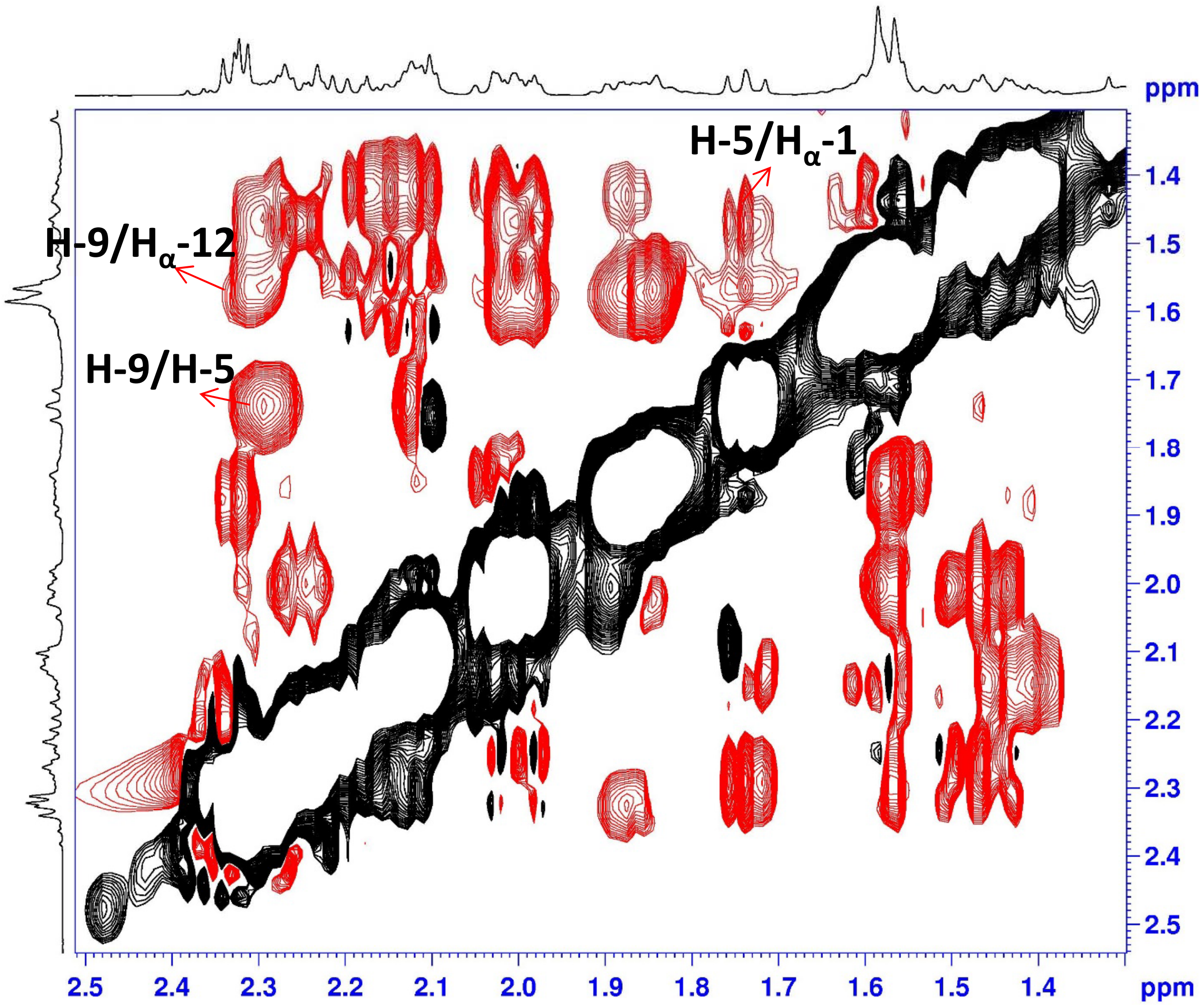

NOESY (400 MHz) spectrum of compound **2** in $CDCl_3$

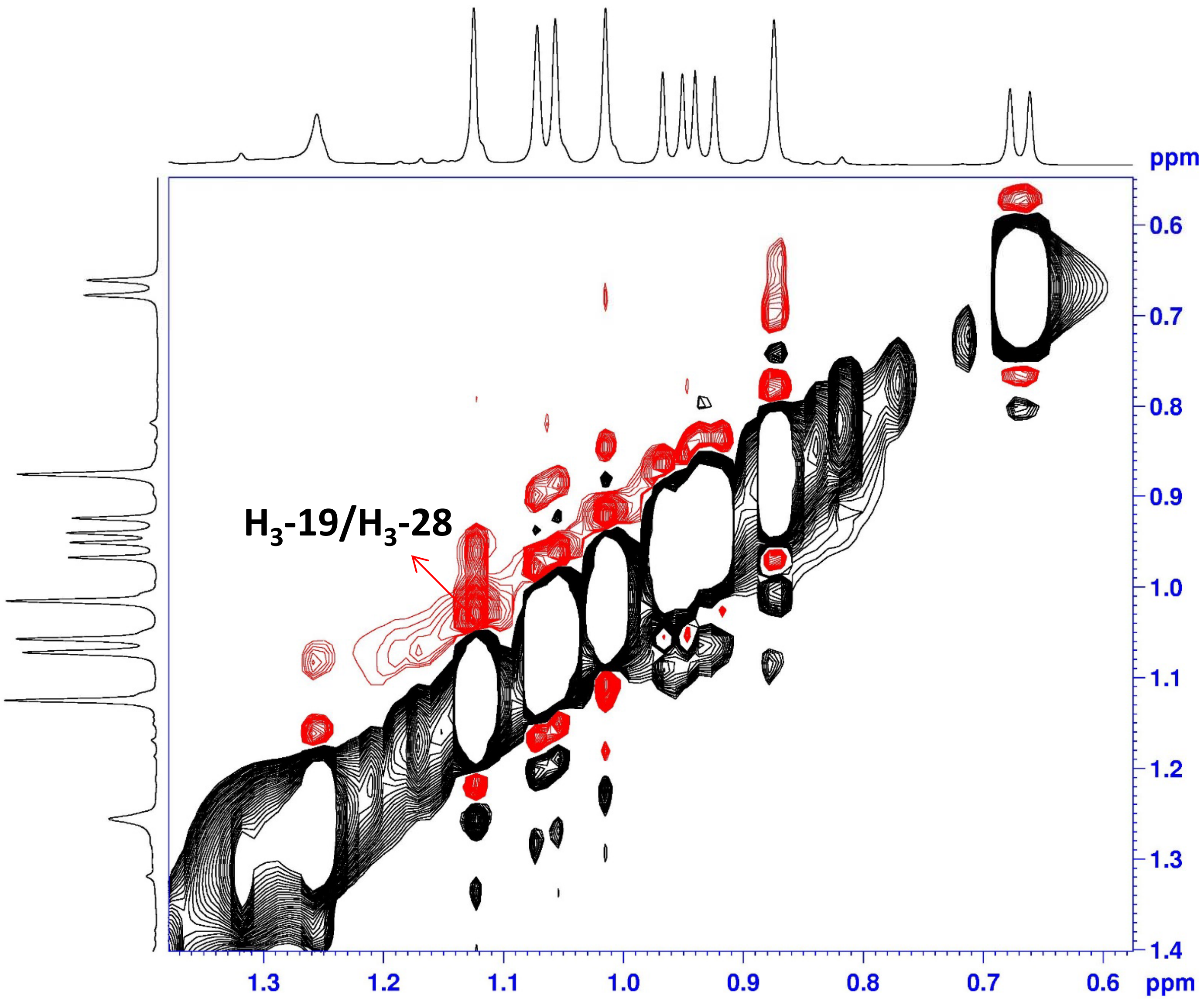

NOESY (400 MHz) spectrum of compound **2** in $CDCl_3$

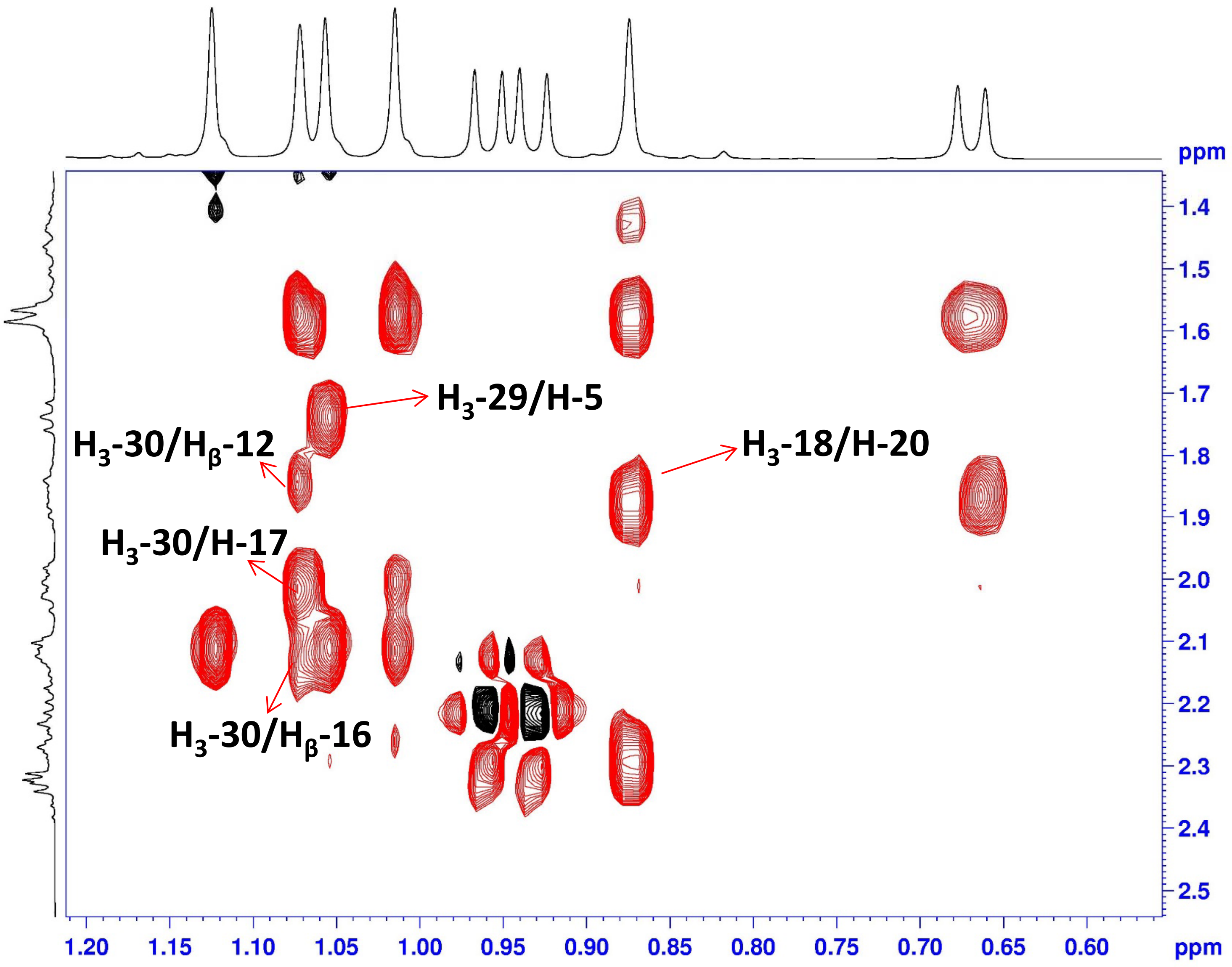

HR-ESIMS for compound **3**

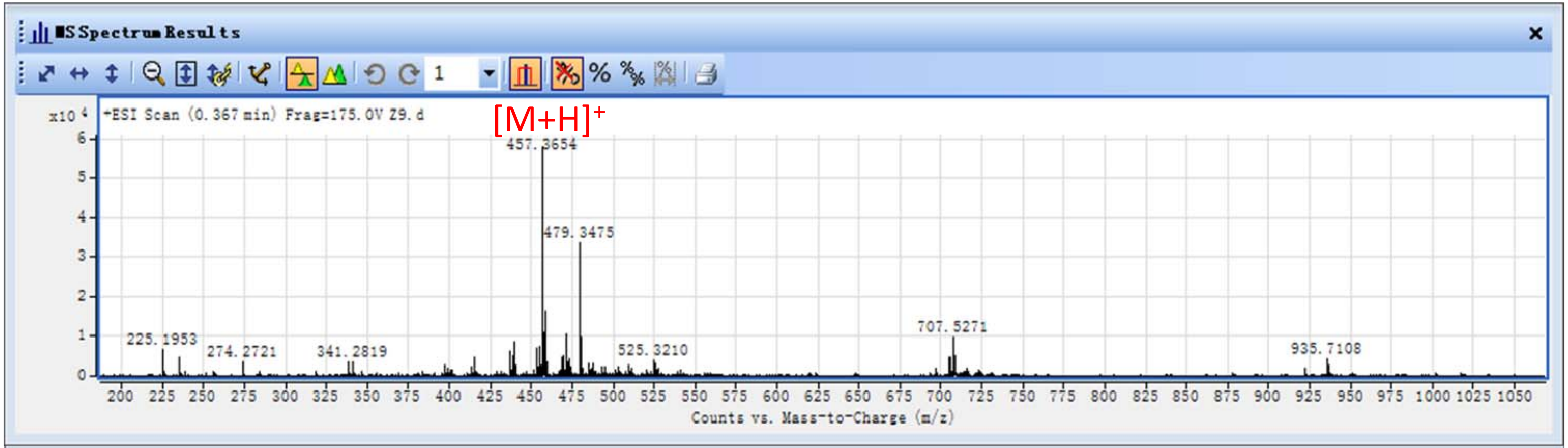

MS Formula Results: + Scan (0.367 min)

m/z	Ion	Formula	Abundance
457.3654	(M+H)+	C30 H49 O3	59344.7

Best	Formula (M)	Ion Formula	Score	Cross Score	Calc m/z	Diff (ppm)	Mass Match	Abund Match	Spacing Match
☑	C30 H48 O3	C30 H49 O3	86.45		457.3676	4.97	78.06	92.06	96.48

Isotope	Abund Sum%	Calc Abund Sum%	m/z	Calc m/z	Diff (ppm)
1	73.47	71.53	457.3654	457.3676	4.94
2	21.53	23.69	458.3684	458.371	5.75
3	4.41	4.24	459.3697	459.3742	9.75
4	0.59	0.54	460.3634	460.3772	29.79

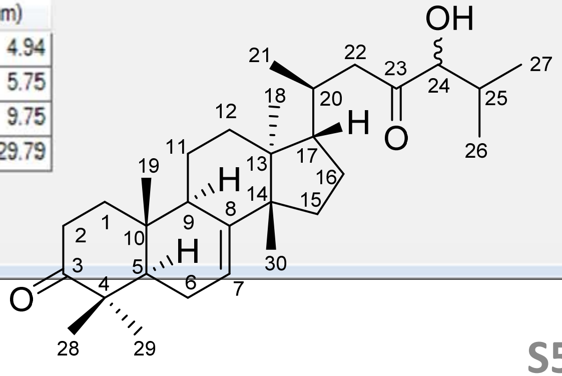

^{1}H NMR (400 MHz) spectrum of compound **3** in $CDCl_3$

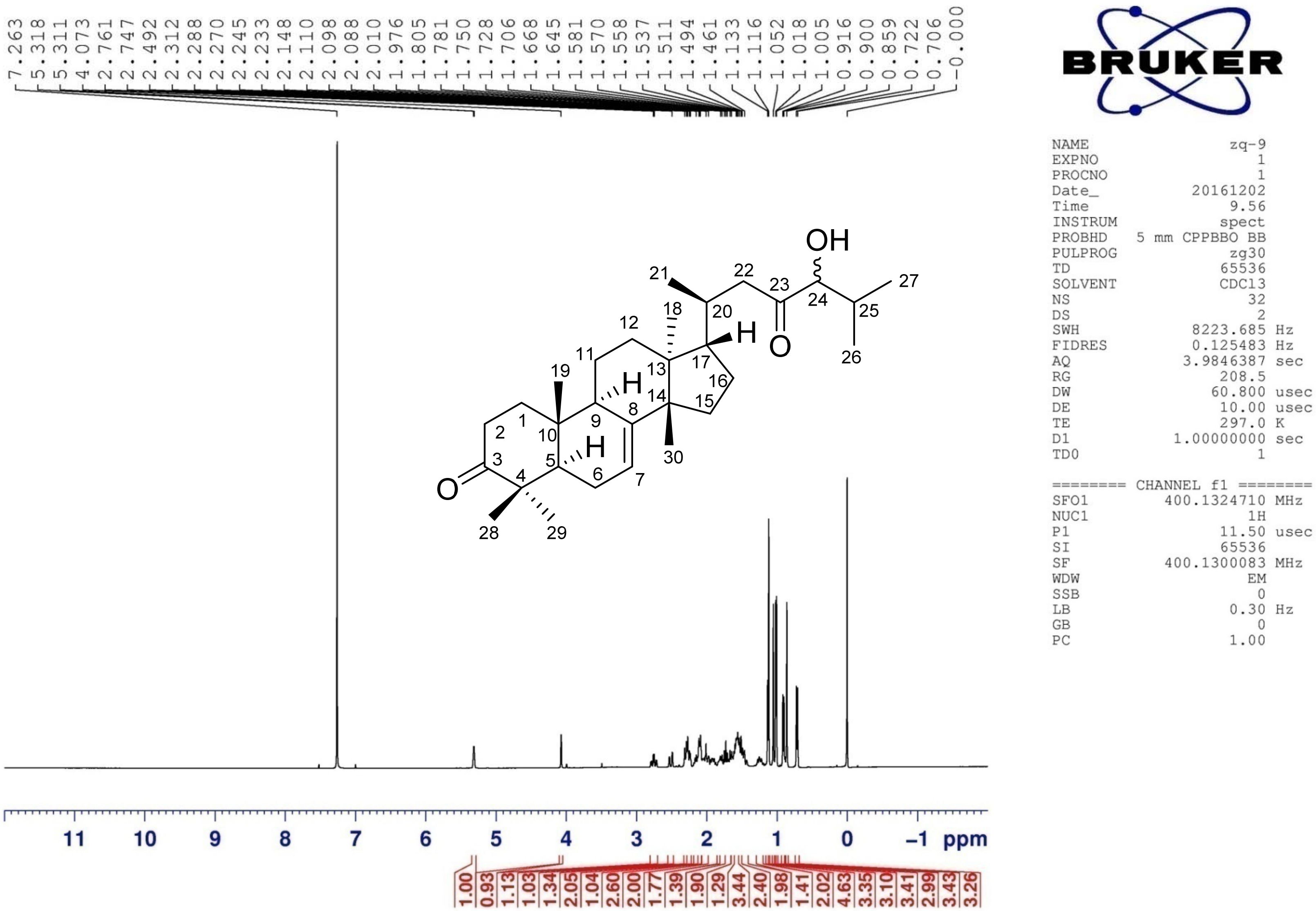

^{1}H NMR (400 MHz) spectrum of compound **3** in $CDCl_3$

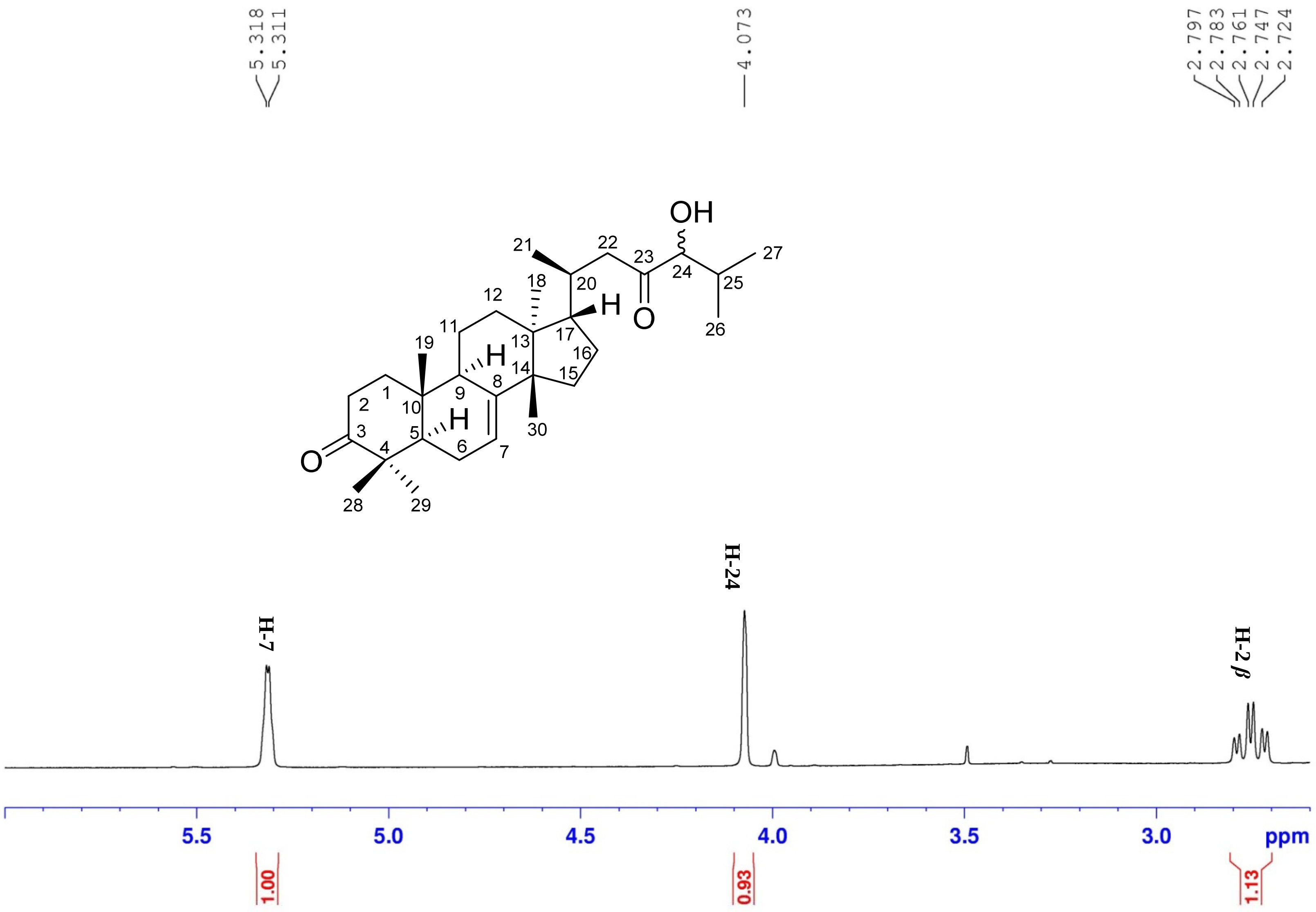

^{1}H NMR (400 MHz) spectrum of compound **3** in $CDCl_3$

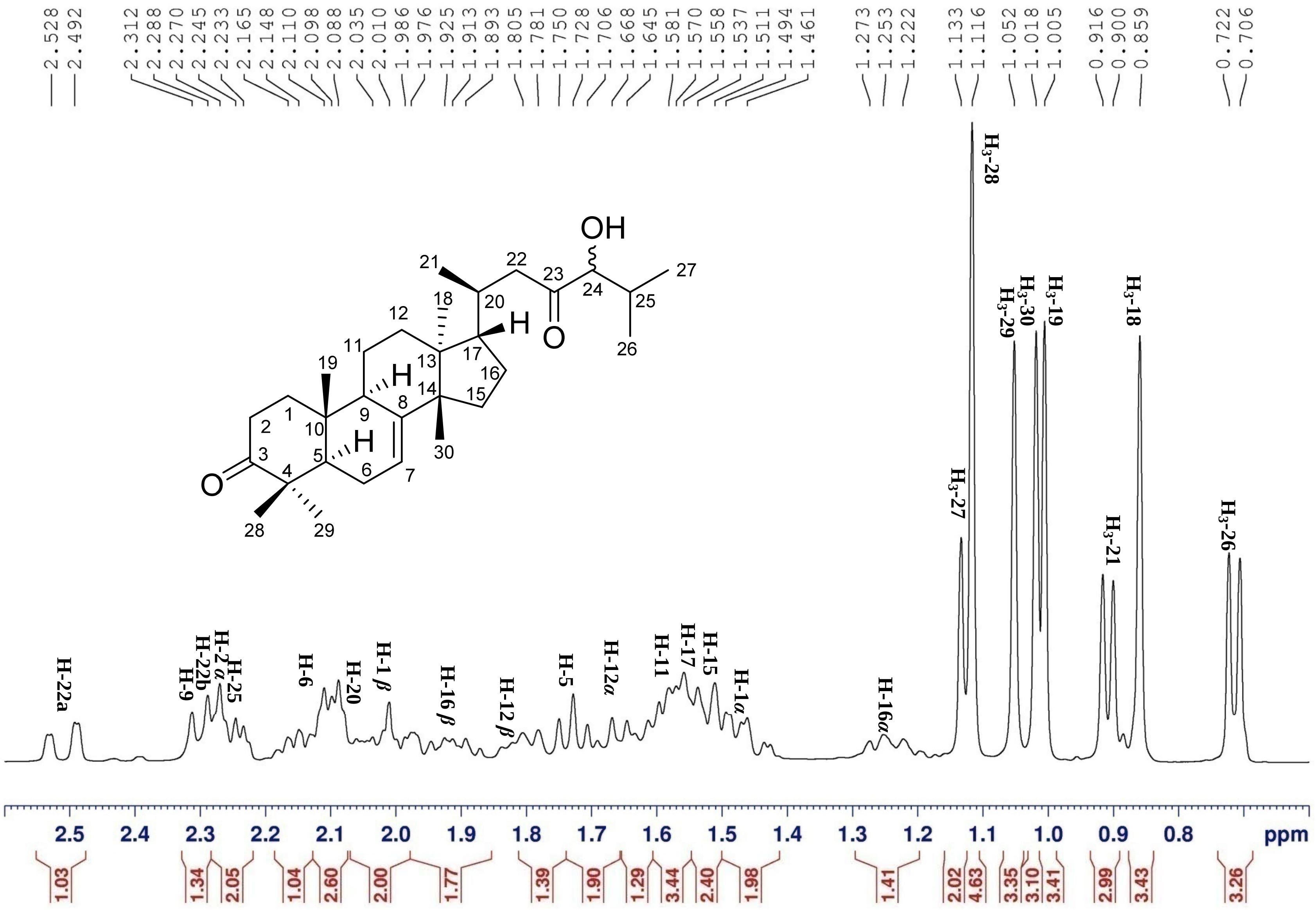

^{13}C NMR (100 MHz) spectrum of compound **3** in $CDCl_3$

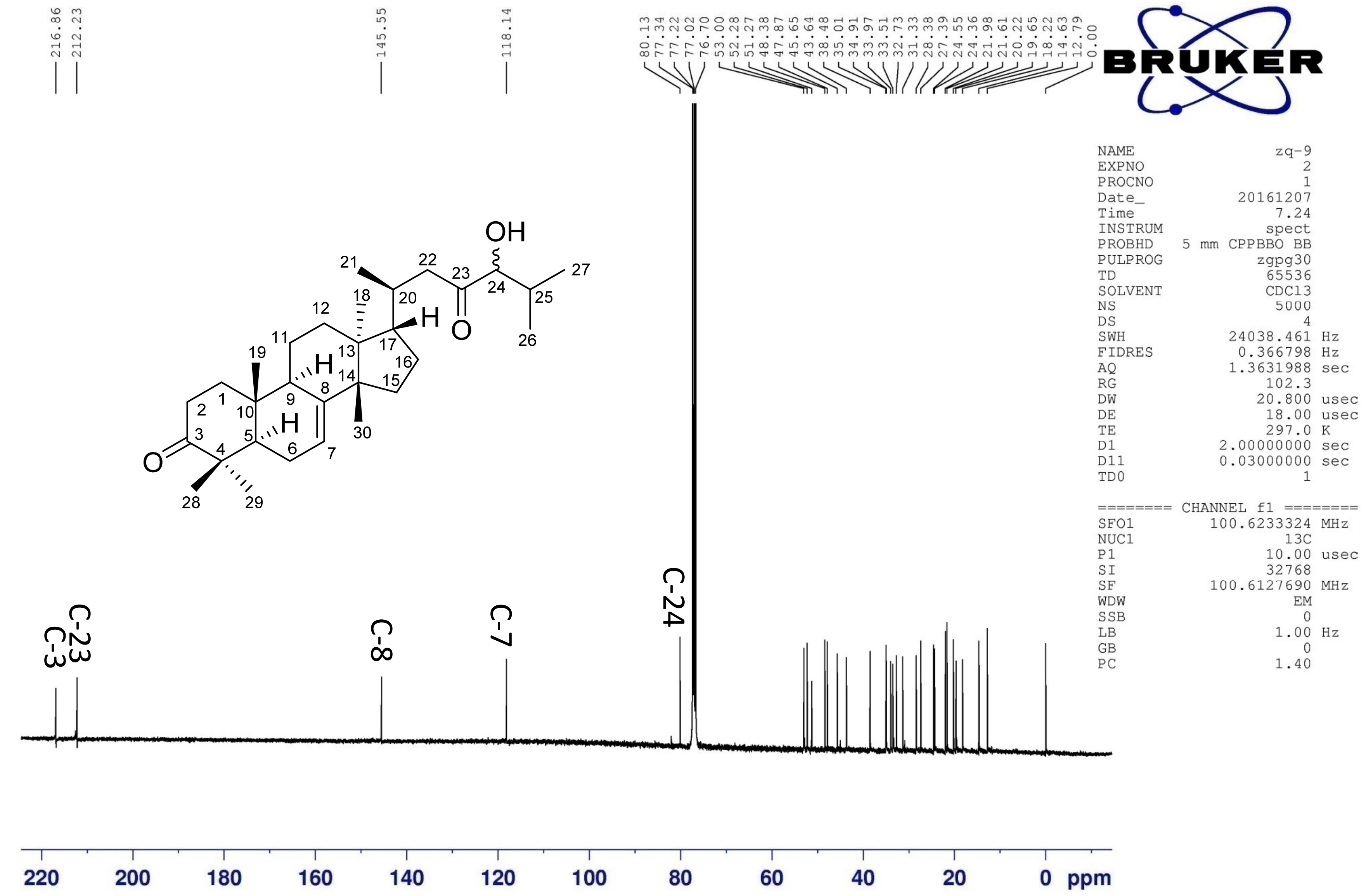

^{13}C NMR (100 MHz) spectrum of compound **3** in $CDCl_3$

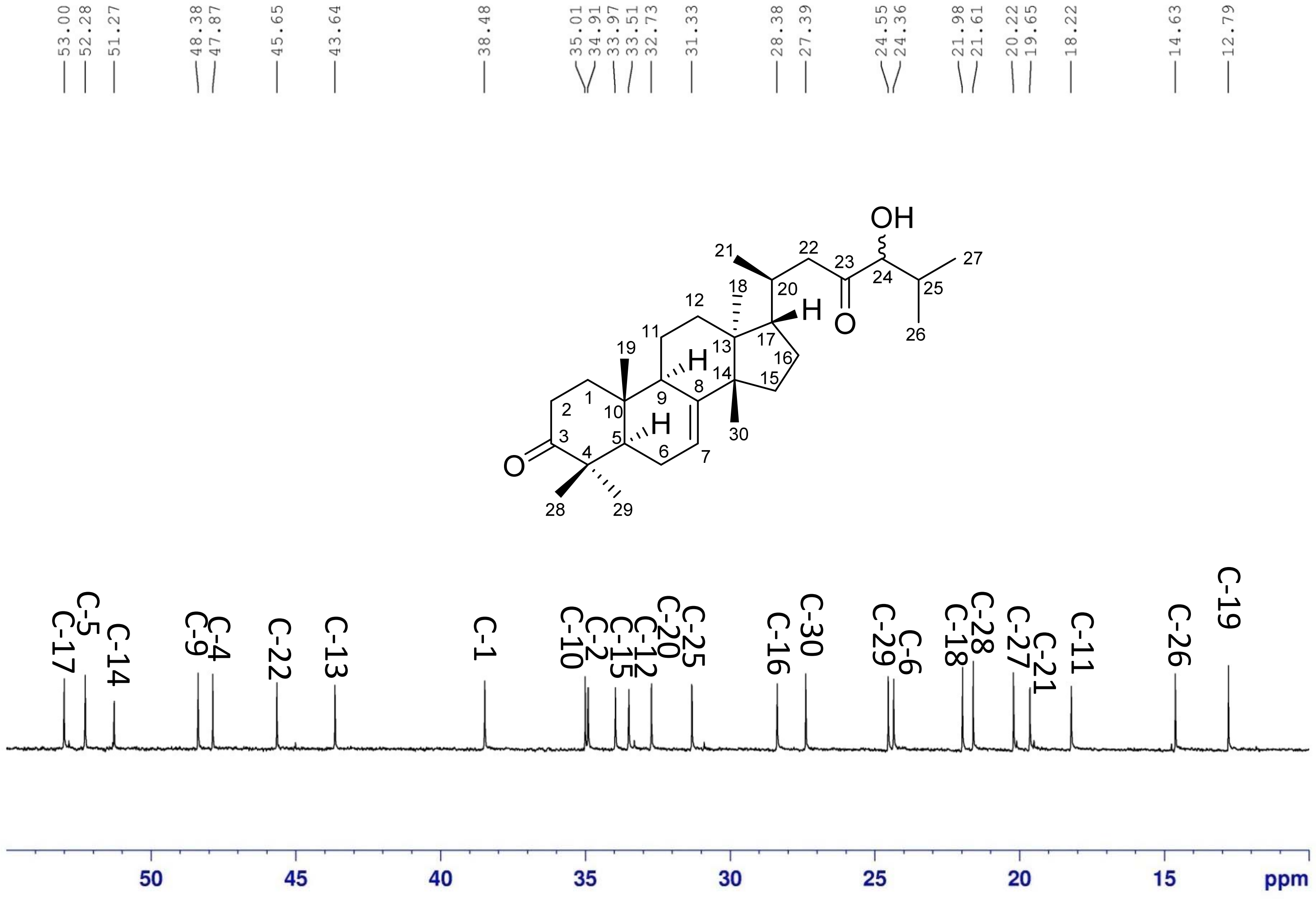

DEPT135 (100 MHz) spectrum of compound **3** in $CDCl_3$

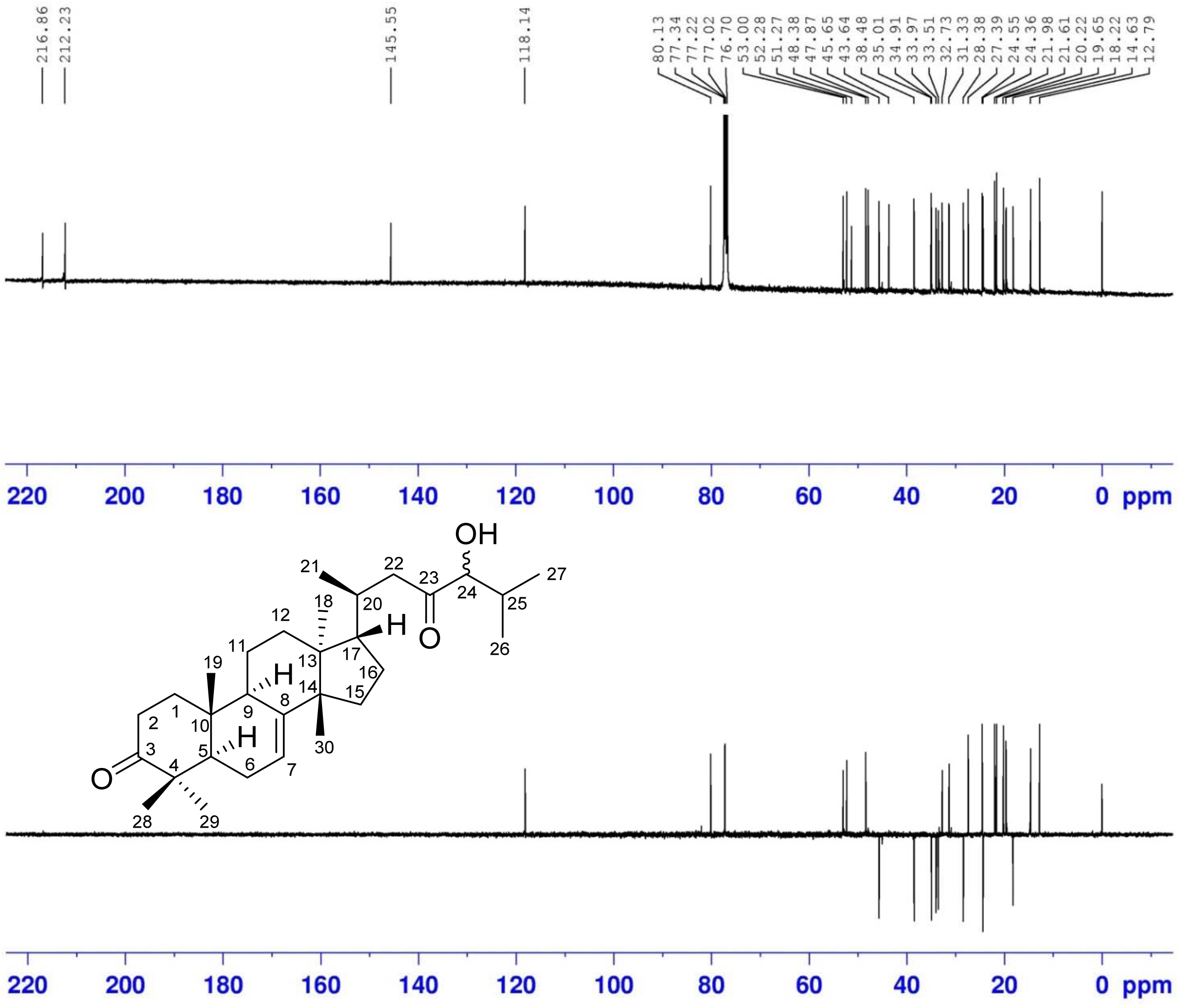


```
NAME                zq-9
EXPNO                  3
PROCNO                 1
Date_           20161207
Time                8.51
INSTRUM            spect
PROBHD   5 mm CPPBBO BB
PULPROG        deptsp135
TD                 65536
SOLVENT            CDCl3
NS                  1500
DS                     4
SWH            24038.461 Hz
FIDRES          0.366798 Hz
AQ             1.3631988 sec
RG                 91.64
DW                20.800 usec
DE                 18.00 usec
TE                 297.0 K
CNST2        145.0000000
D1            2.00000000 sec
D2            0.00344828 sec
D12           0.00002000 sec
TD0                    1

======== CHANNEL f1 ========
SFO1         100.6233324 MHz
NUC1                 13C
P1                 10.00 usec
P13              2000.00 usec
SI                 32768
SF           100.6127690 MHz
WDW                   EM
SSB                    0
LB                  1.00 Hz
GB                     0
PC                  1.40
```

DEPT135 (100 MHz) spectrum of compound **3** in $CDCl_3$

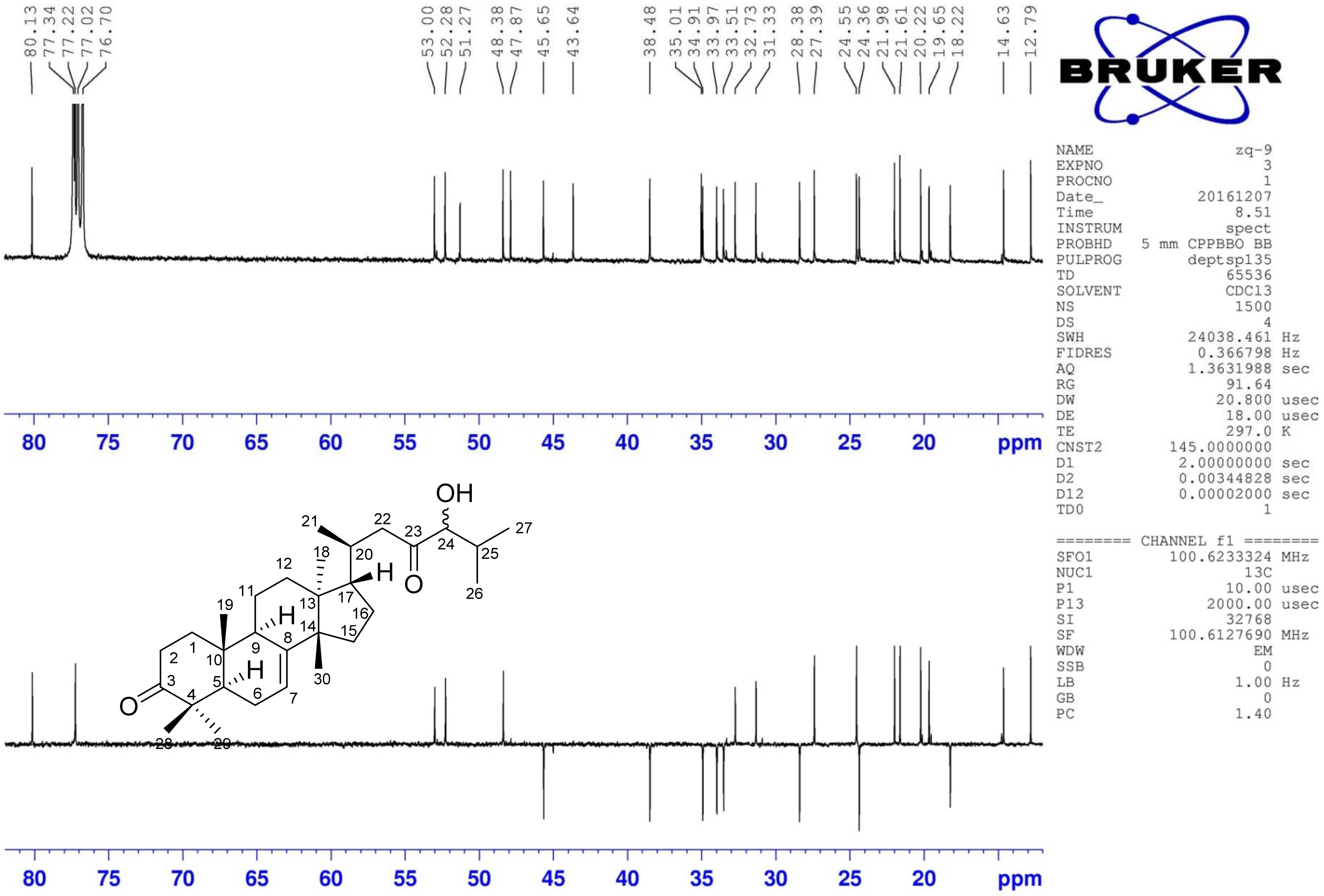

^{1}H-^{1}H COSY (400 MHz) spectrum of compound **3** in $CDCl_3$

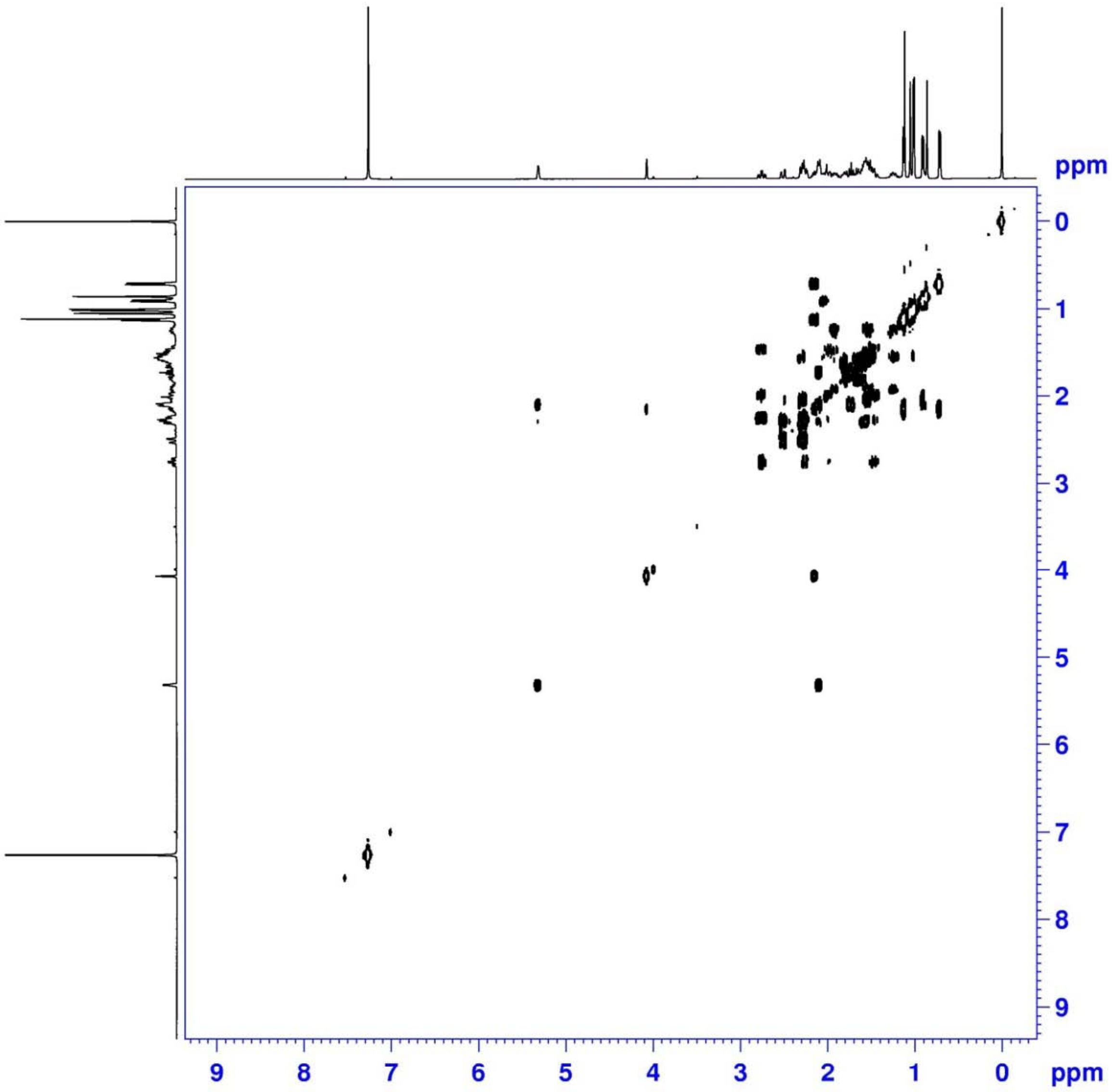


```
NAME                ZQ-9-1
EXPNO                    4
PROCNO                   1
Date_             20161208
Time                 15.26
INSTRUM              spect
PROBHD   5 mm CPPBBO BB
PULPROG         cosygpppqf
TD                    2048
SOLVENT              CDCl3
NS                      32
DS                       8
SWH               3906.250 Hz
FIDRES            1.907349 Hz
AQ               0.2621940 sec
RG                   208.5
DW                 128.000 usec
DE                   10.00 usec
TE                   297.0 K
D0              0.00000300 sec
D1              1.89678097 sec
D11             0.03000000 sec
D12             0.00002000 sec
D13             0.00000400 sec
D16             0.00020000 sec
IN0             0.00025600 sec

======== CHANNEL f1 ========
SFO1           400.1318006 MHz
NUC1                    1H
P0                   11.50 usec
P1                   11.50 usec
P17                2500.00 usec
ND0                      1
TD                     128
SFO1              400.1318 MHz
FIDRES           30.517578 Hz
SW                   9.762 ppm
FnMODE                  QF
SI                    1024
SF             400.1300064 MHz
WDW                  QSINE
SSB                      0
LB                    0.00 Hz
GB                       0
PC                    1.40
SI                    1024
MC2                     QF
SF             400.1300061 MHz
WDW                  QSINE
SSB                      0
LB                    0.00 Hz
```

^1H-^1H COSY (400 MHz) spectrum of compound **3** in $CDCl_3$

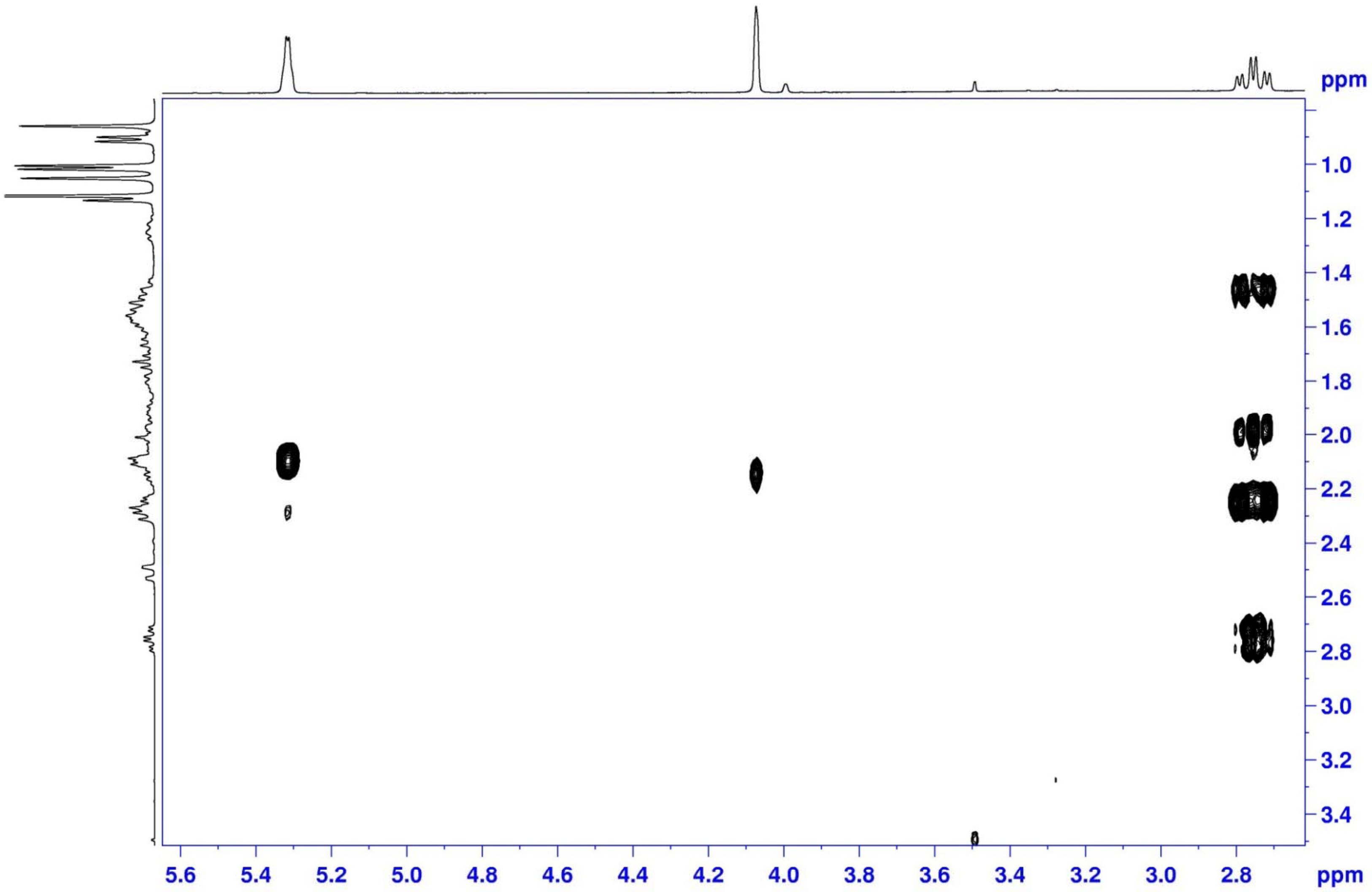

^1H-^1H COSY (400 MHz) spectrum of compound **3** in $CDCl_3$

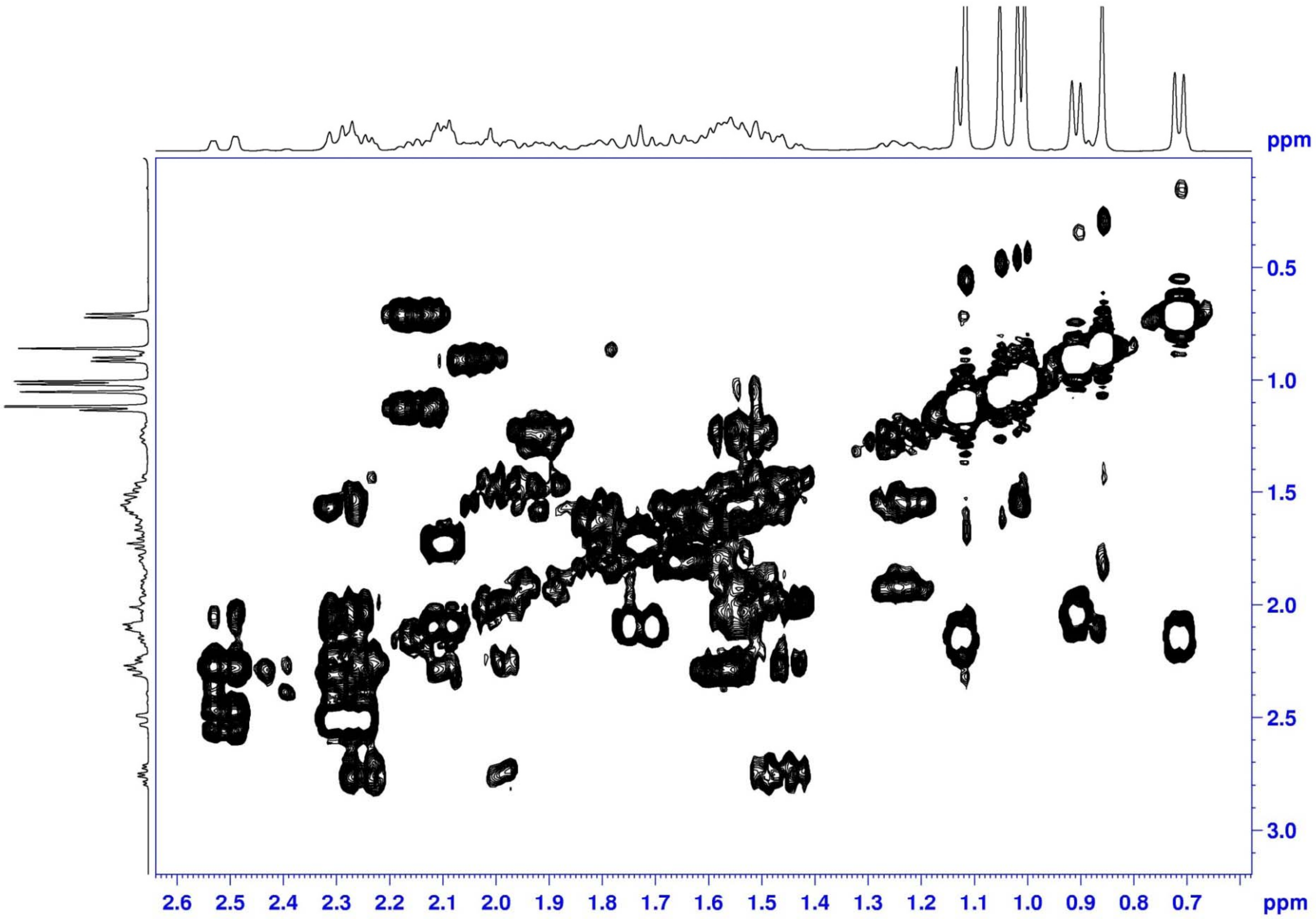

HSQC (400 MHz) spectrum of compound **3** in $CDCl_3$

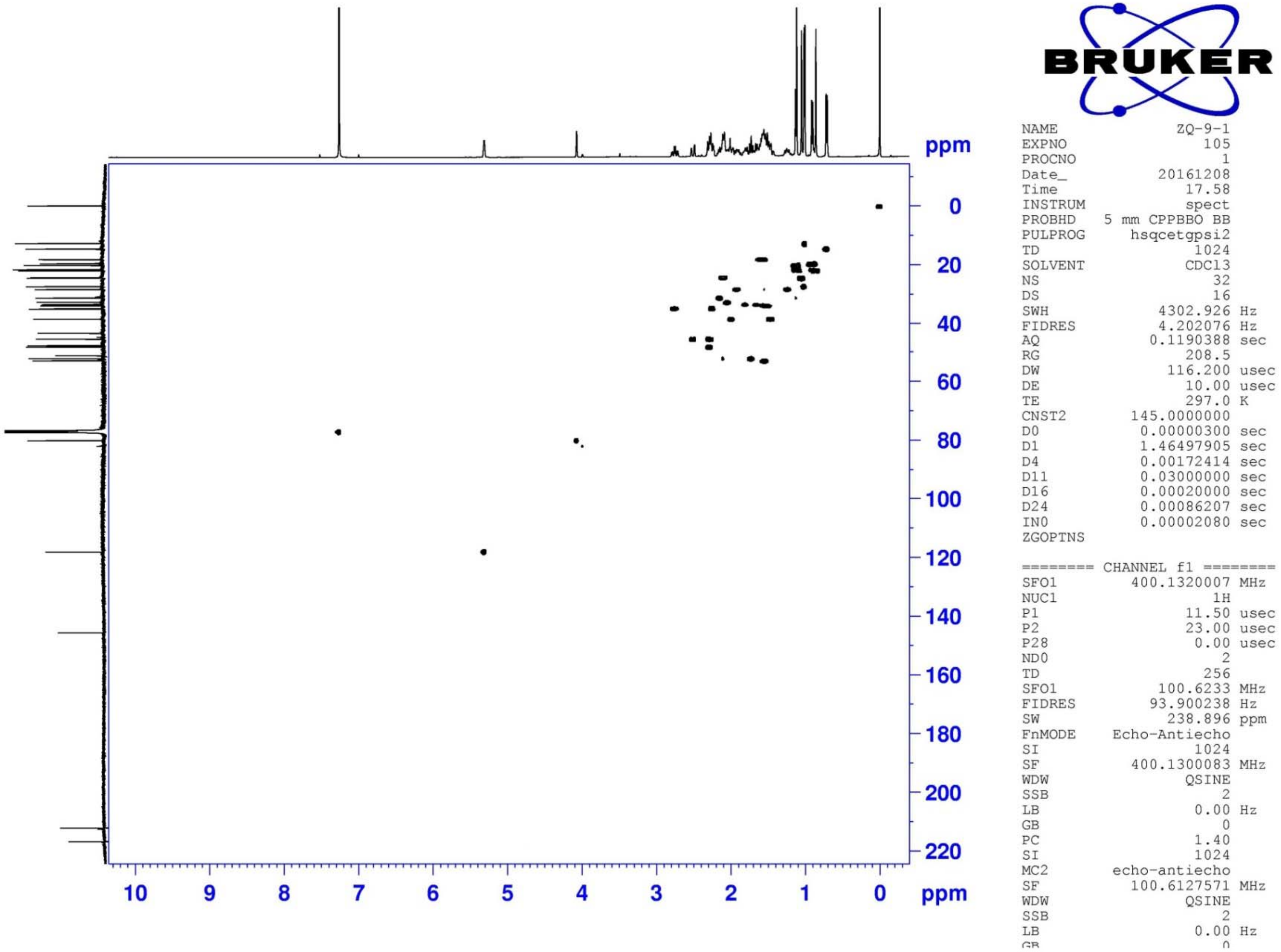

HSQC (400 MHz) spectrum of compound **3** in $CDCl_3$

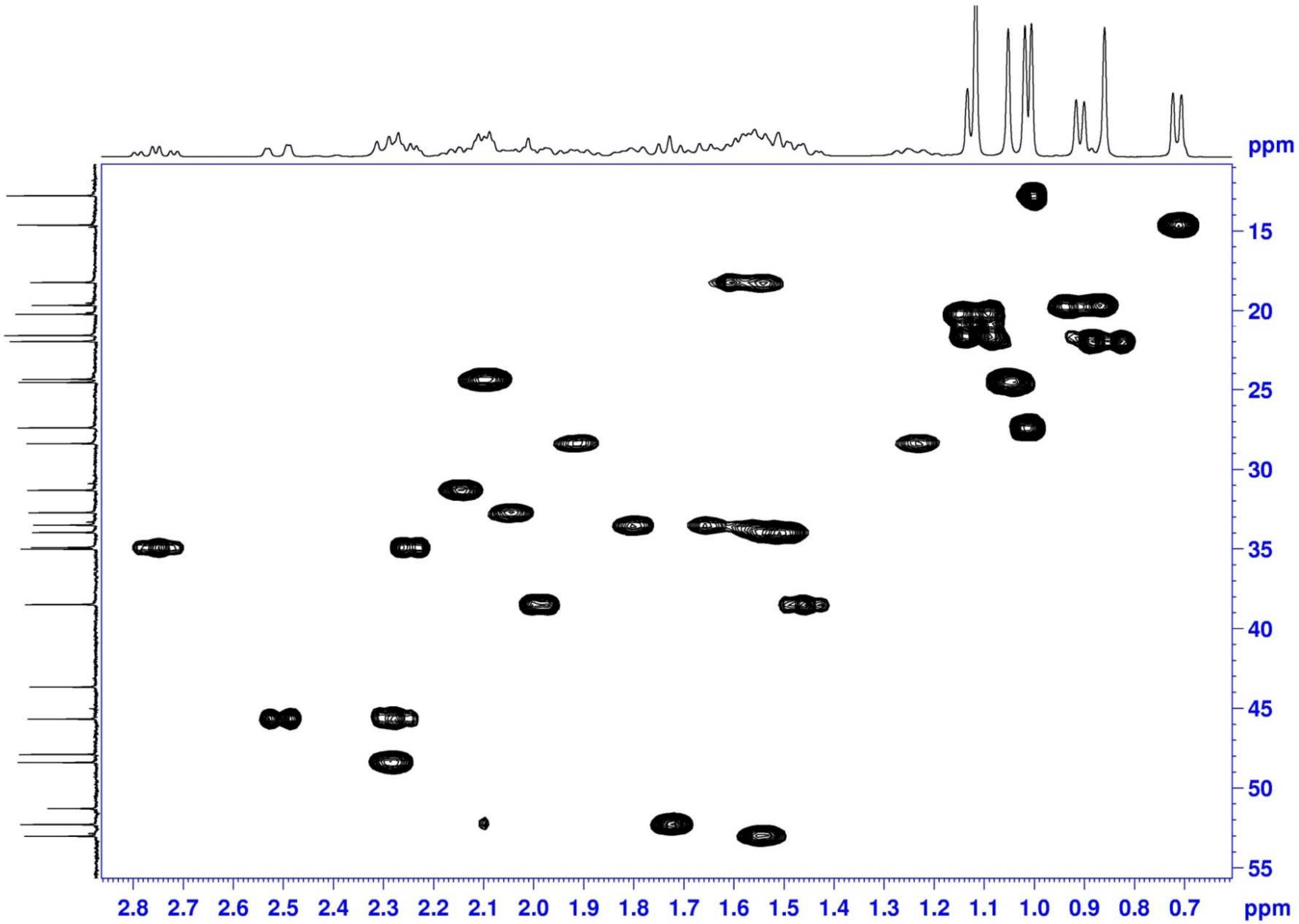

HMBC (400 MHz) spectrum of compound **3** in $CDCl_3$

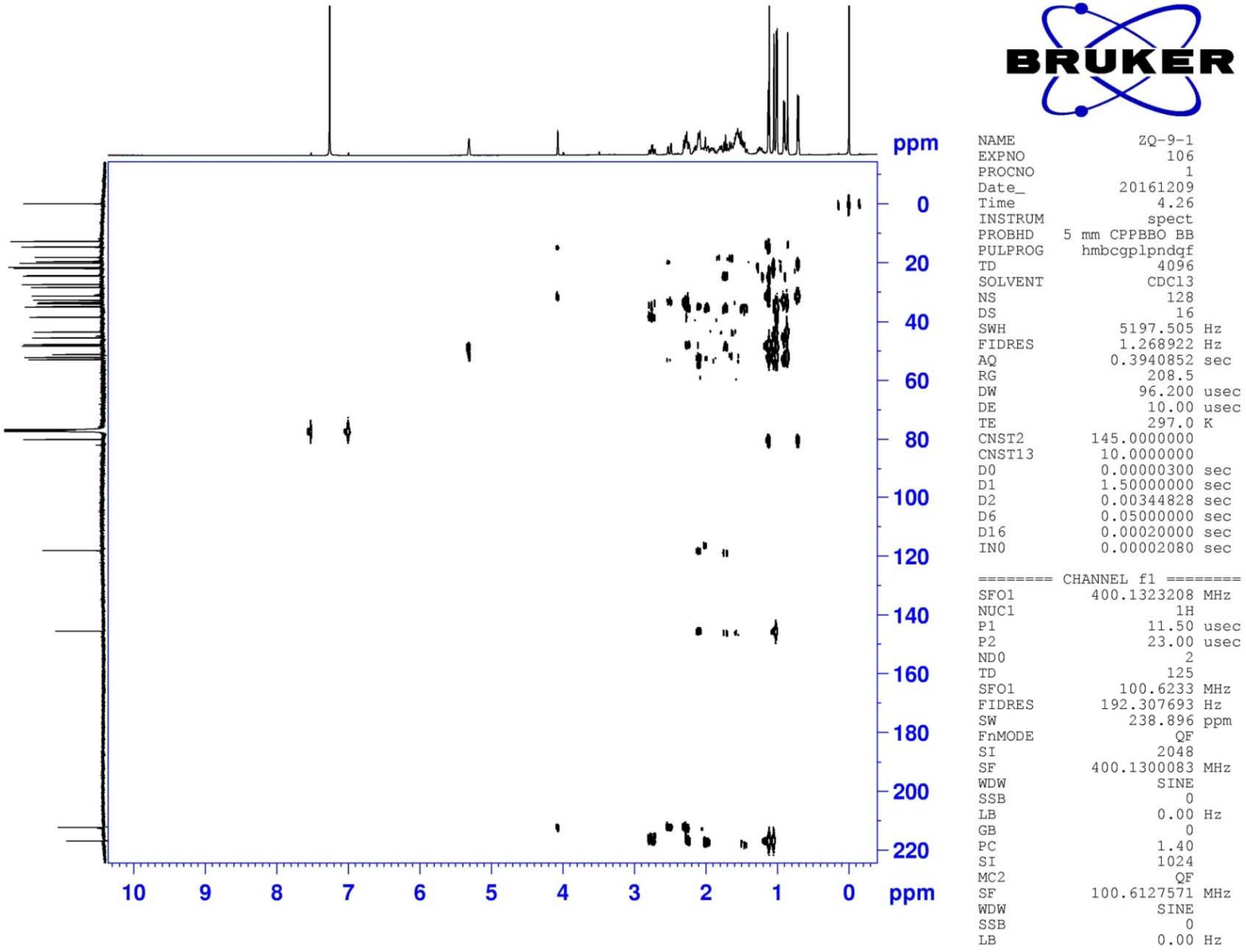

HMBC (400 MHz) spectrum of compound **3** in $CDCl_3$

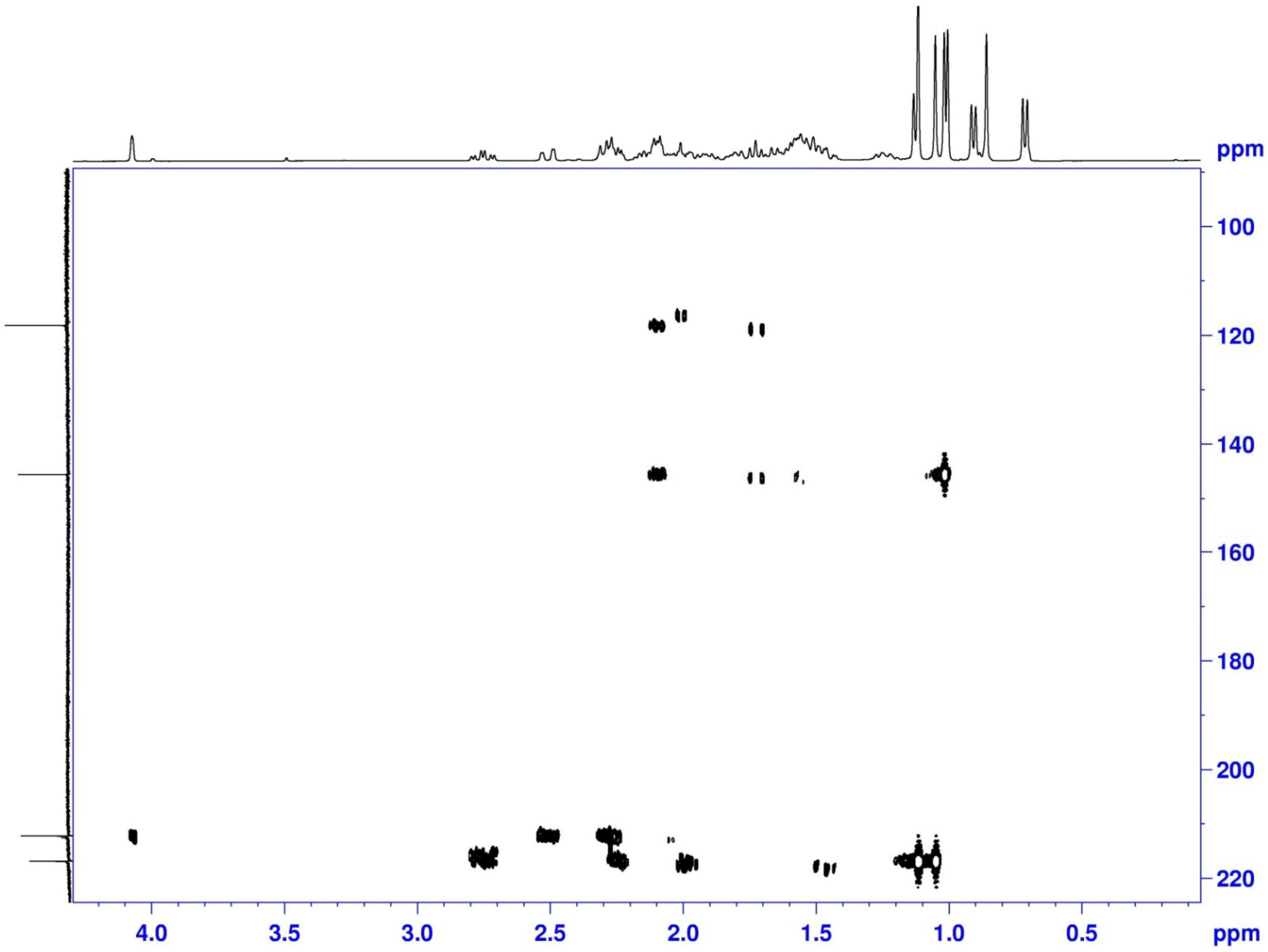

HMBC (400 MHz) spectrum of compound **3** in $CDCl_3$

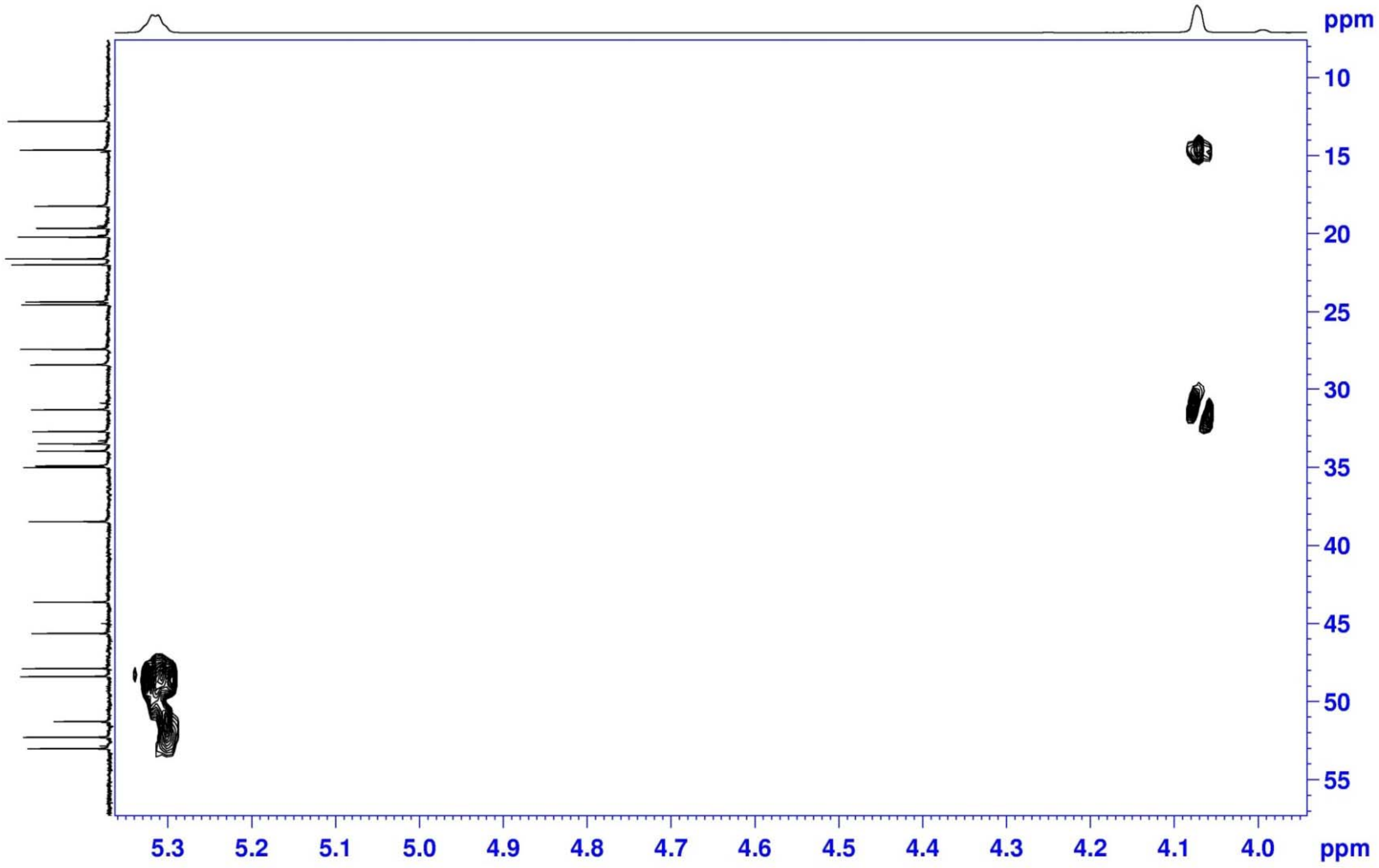

HMBC (400 MHz) spectrum of compound **3** in $CDCl_3$

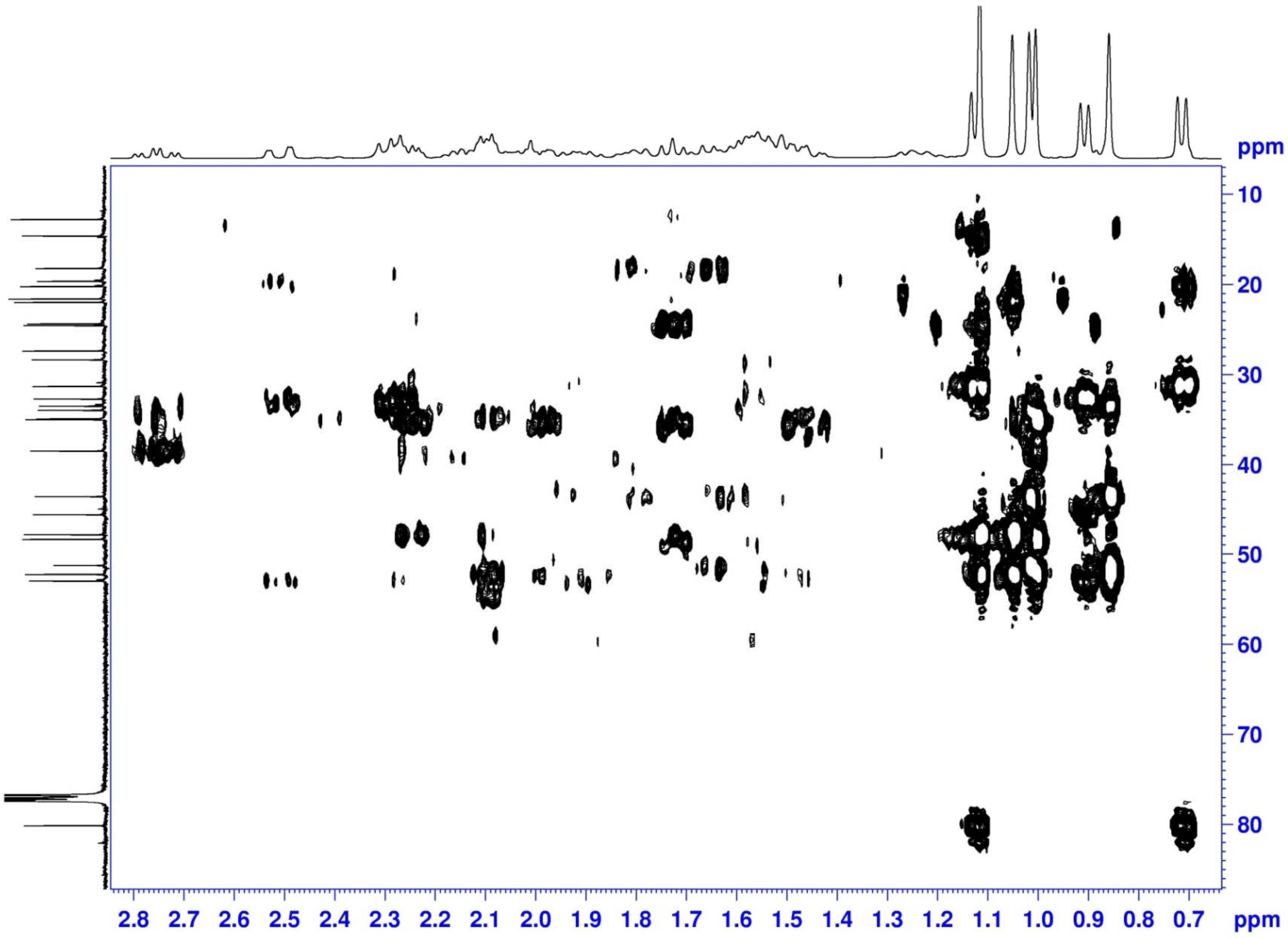

HMBC (400 MHz) spectrum of compound **3** in $CDCl_3$

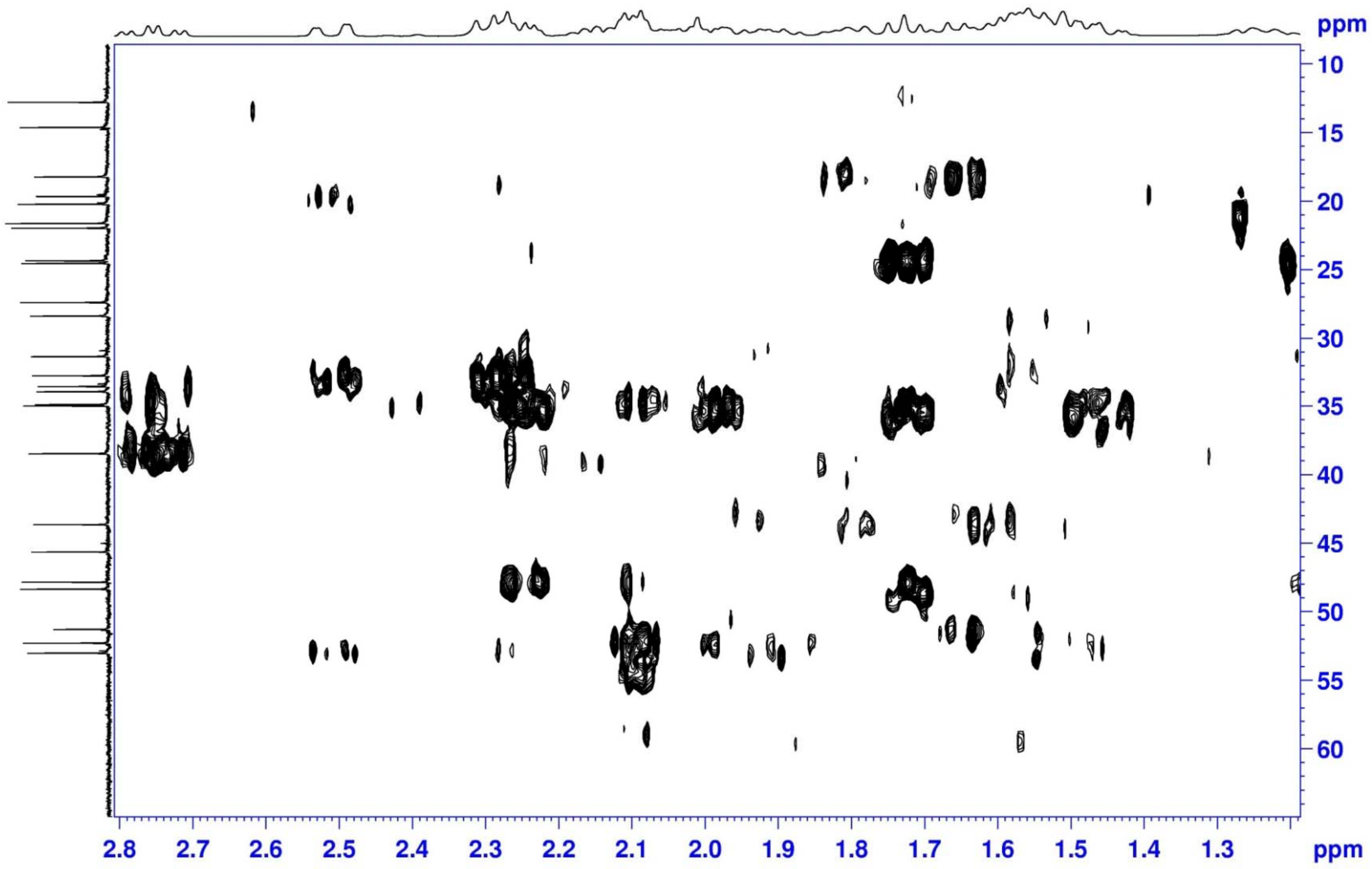

HMBC (400 MHz) spectrum of compound **3** in $CDCl_3$

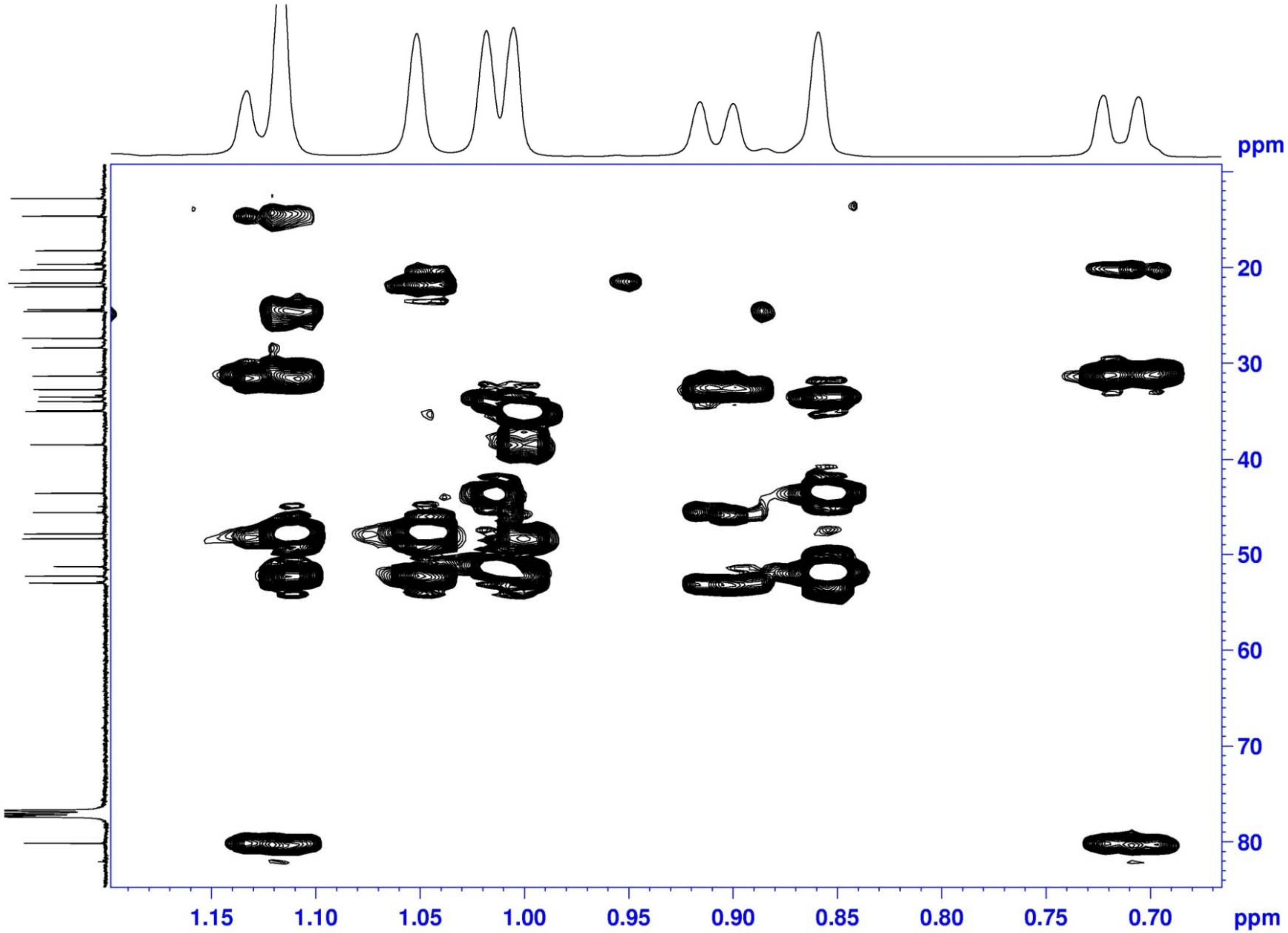

NOESY (400 MHz) spectrum of compound **3** in $CDCl_3$

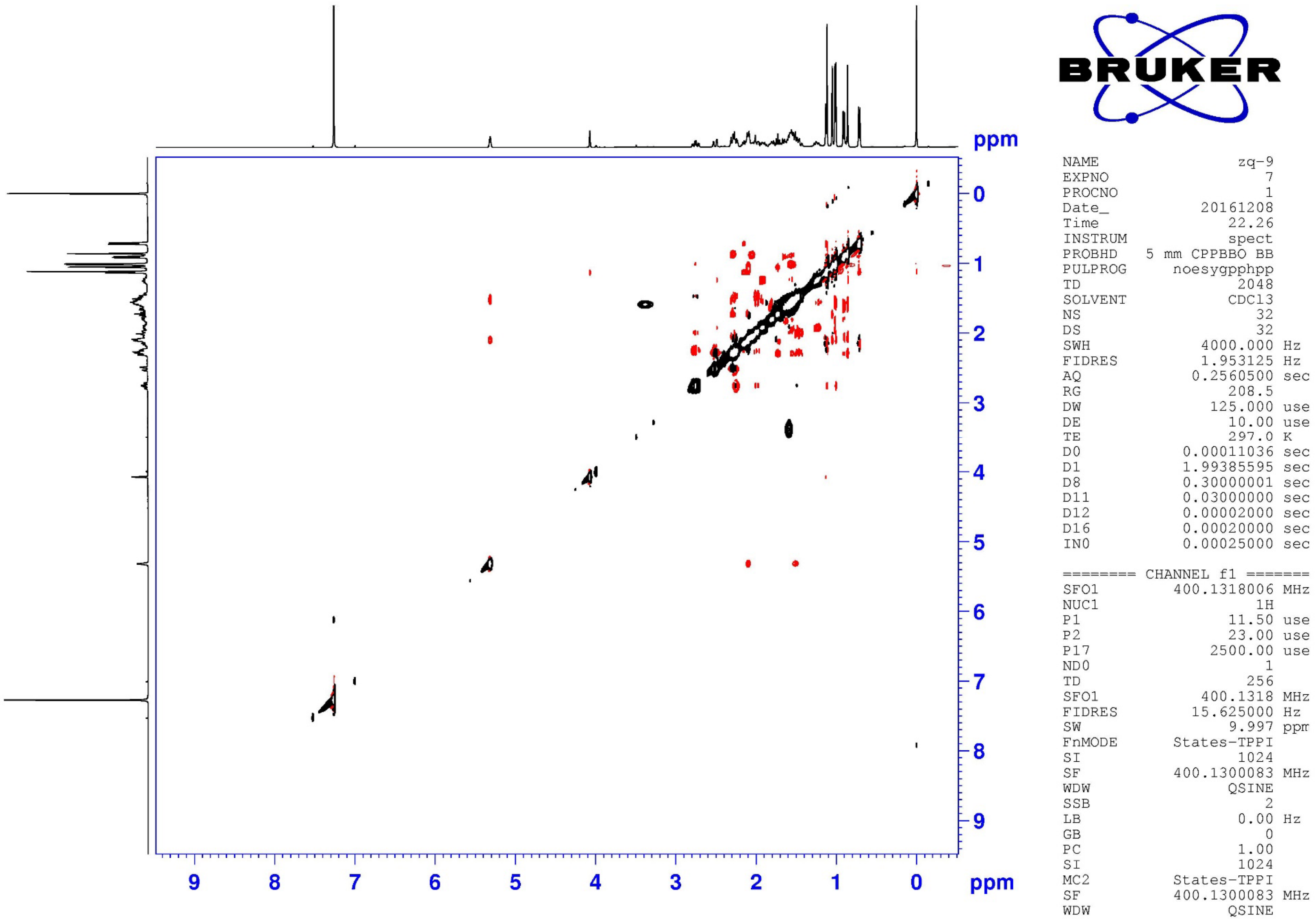

NOESY (400 MHz) spectrum of compound **3** in $CDCl_3$

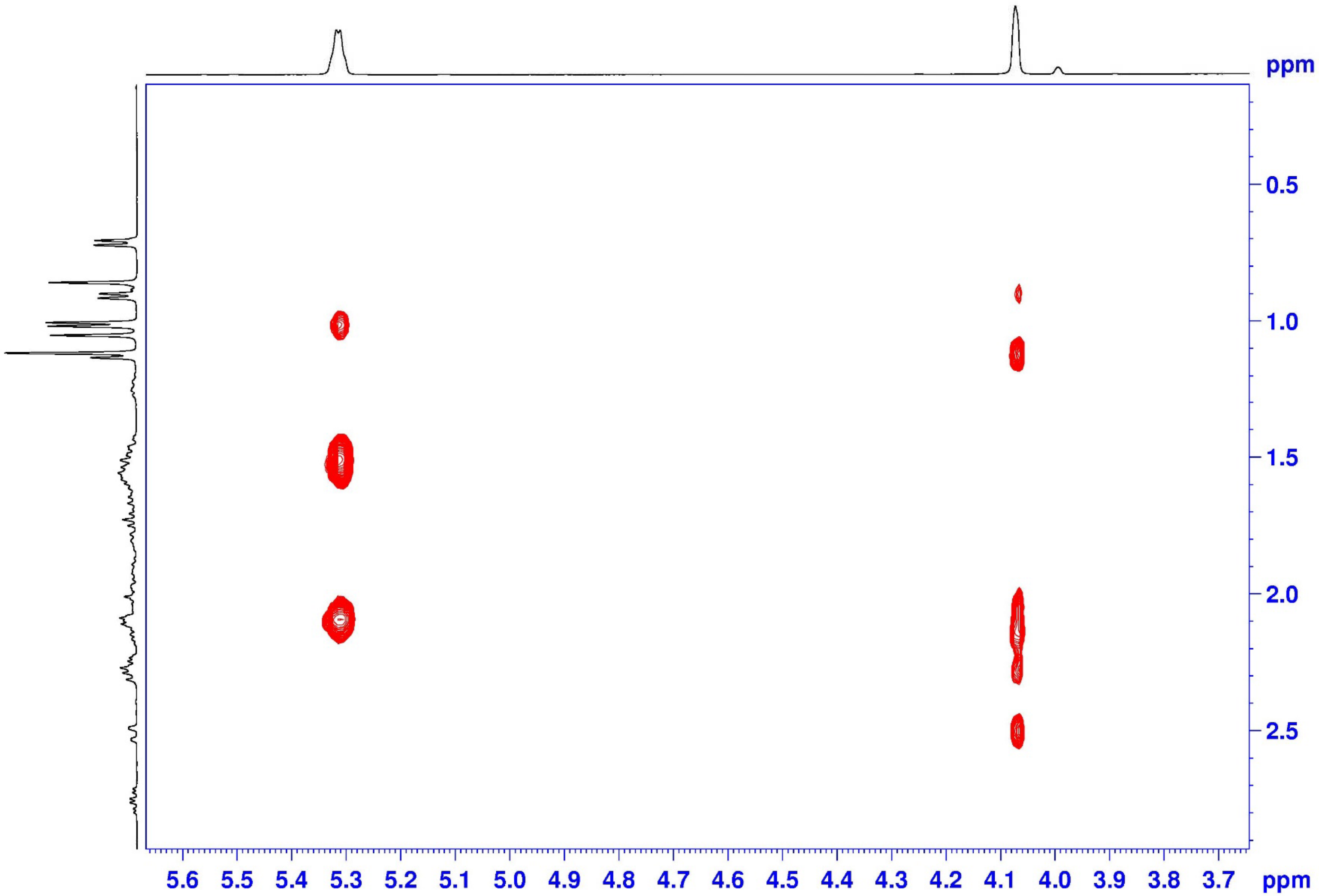

NOESY (400 MHz) spectrum of compound **3** in $CDCl_3$

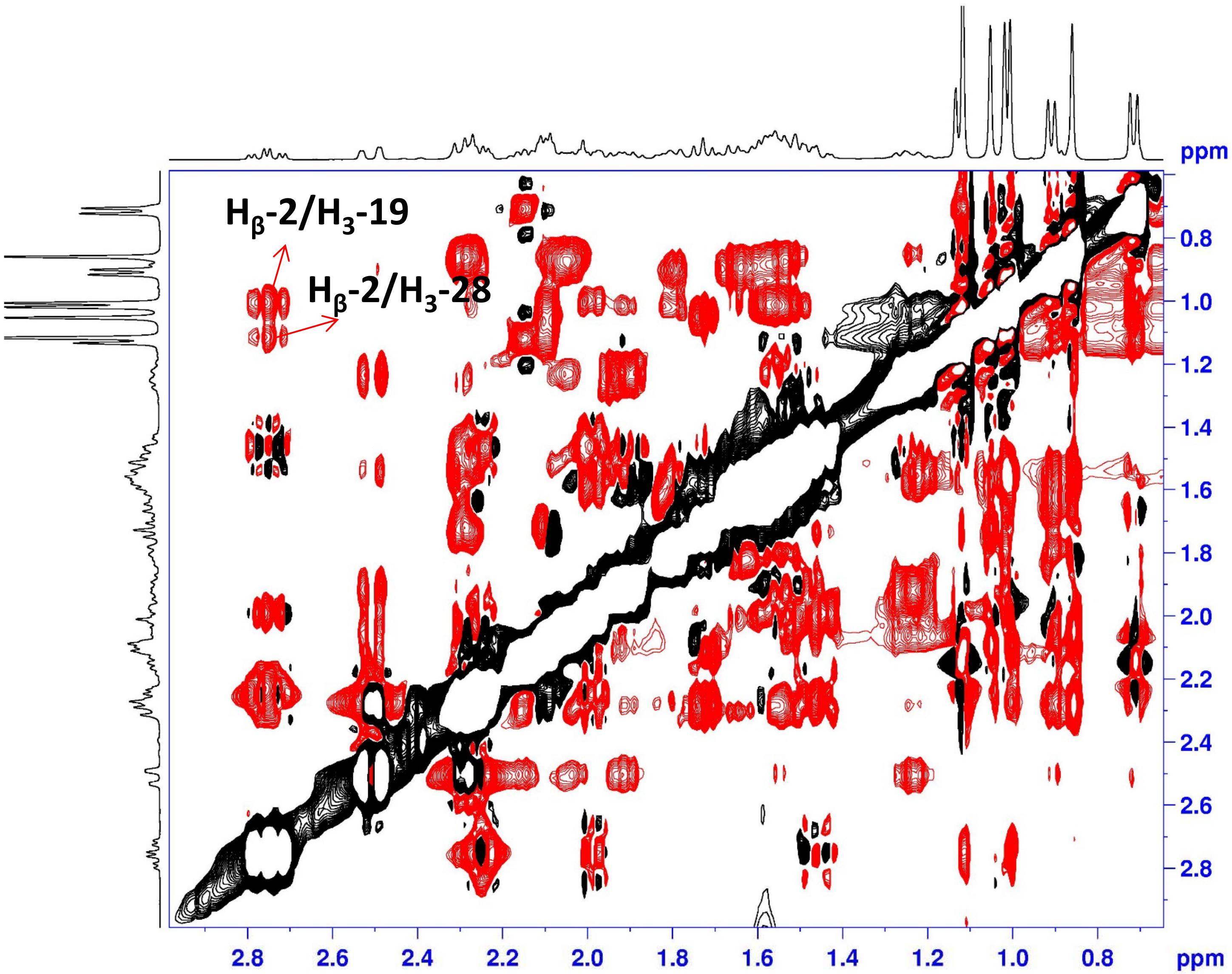

NOESY (400 MHz) spectrum of compound **3** in $CDCl_3$

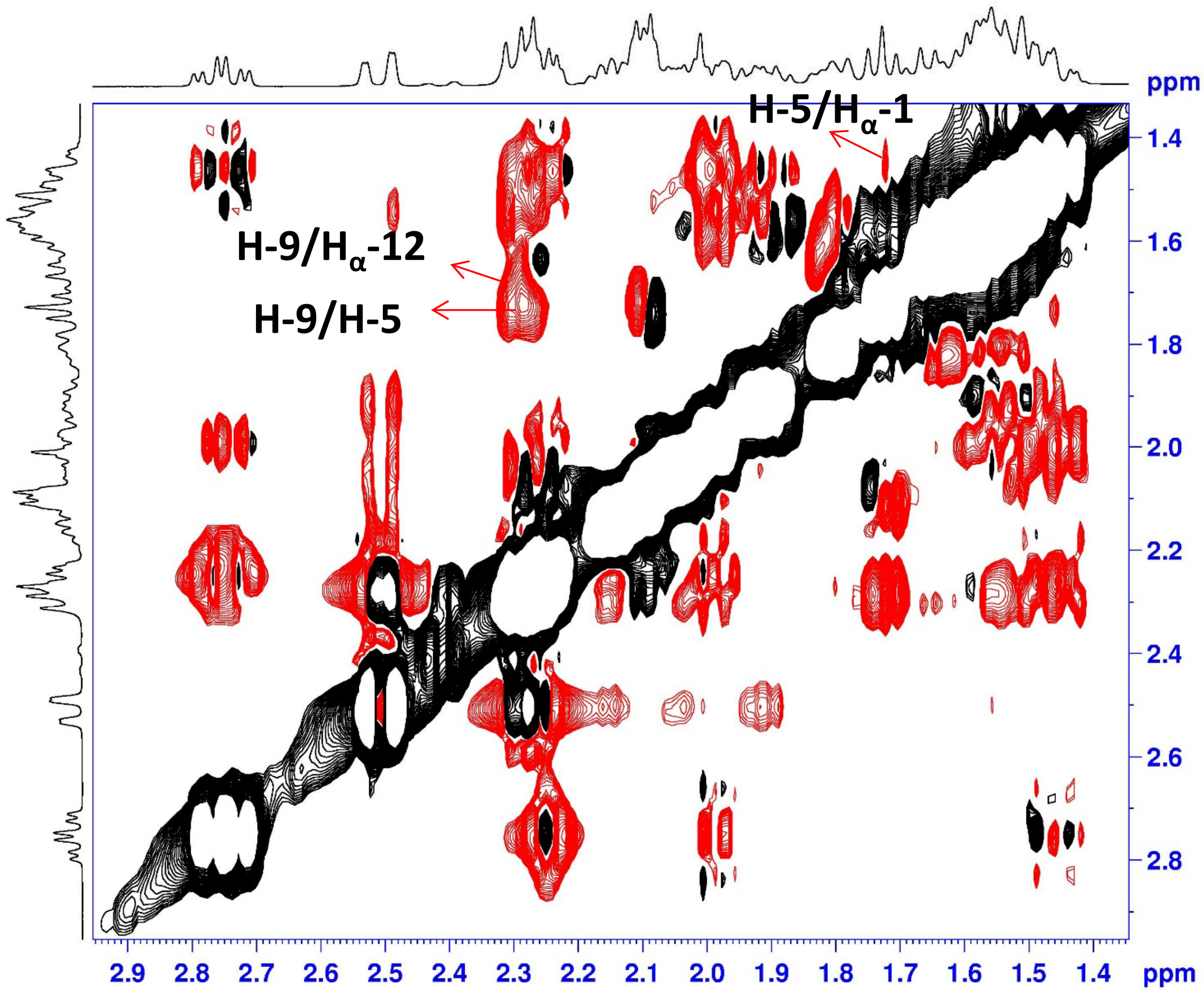

NOESY (400 MHz) spectrum of compound **3** in $CDCl_3$

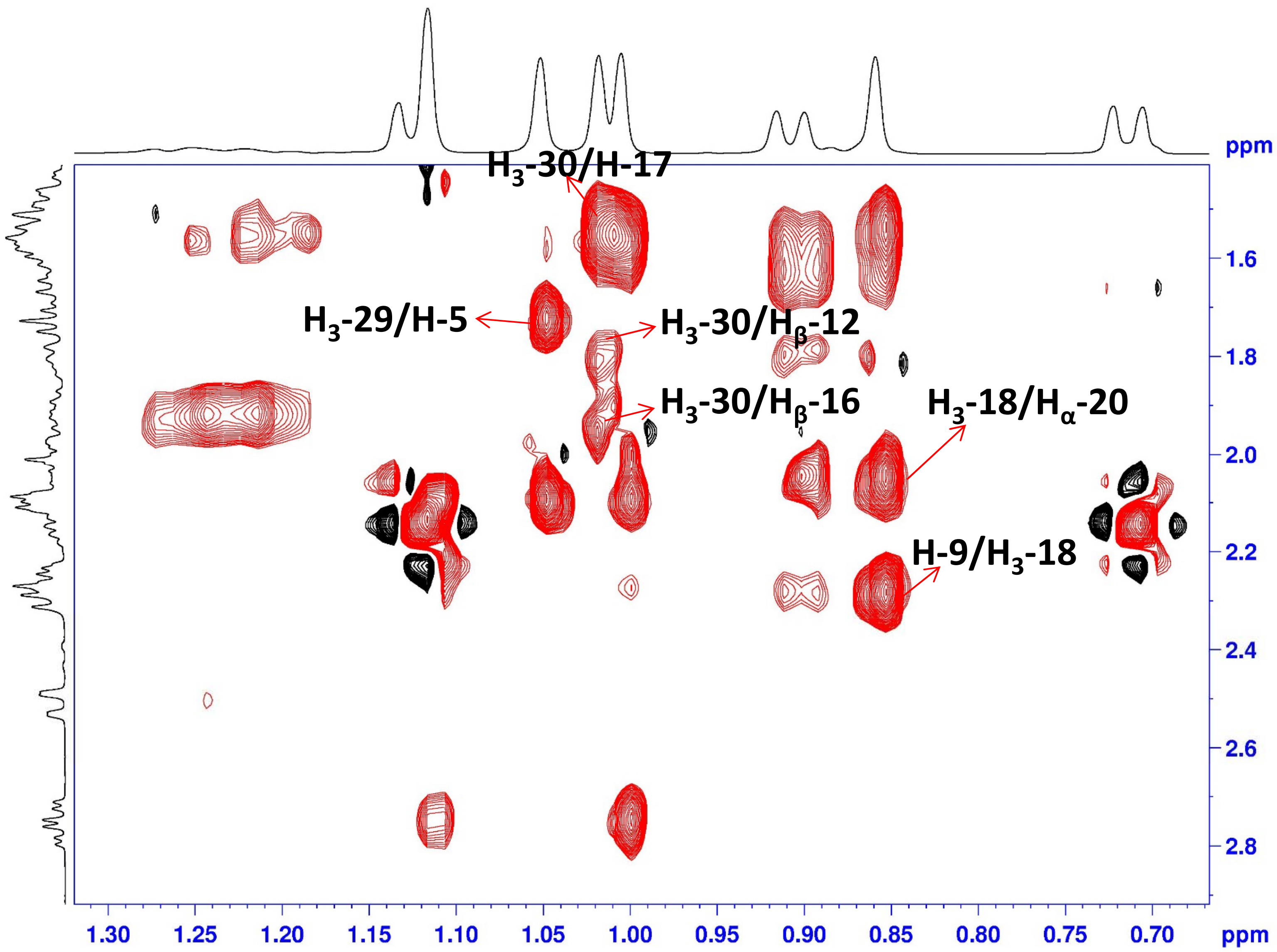

HR-ESIMS for compound **4**

Generic Display Report

Analysis Info

Analysis Name	D:\Data\MS\data\201802\liwanshan_XG-53-5_neg_55_01_4273.d	Acquisition Date	2/8/2018 5:09:54 PM
Method	LC_Direct Infusion_neg_100-1000mz.m	Operator	SCSIO
Sample Name	liwanshan_XG-53-5_neg	Instrument	maXis
Comment			

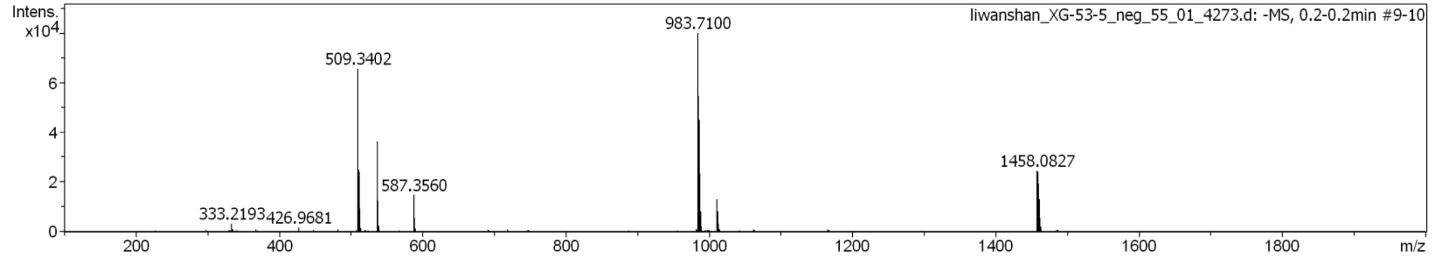

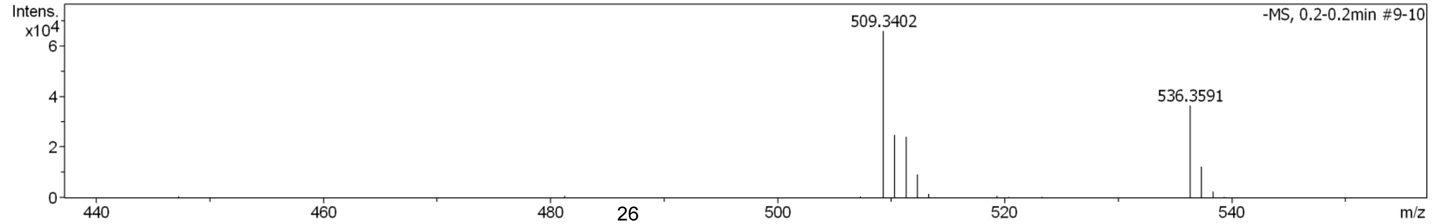

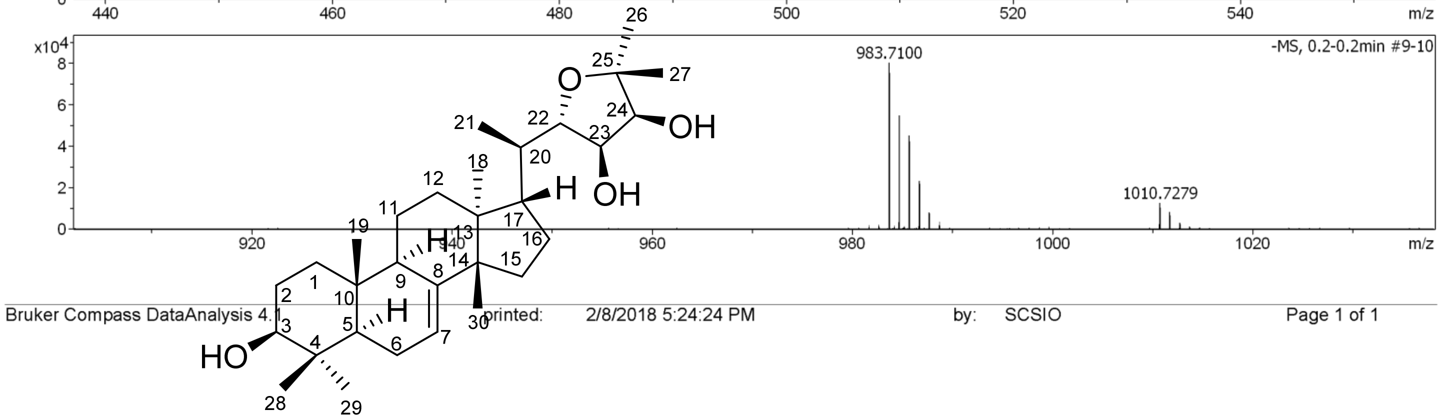

Bruker Compass DataAnalysis 4.1 printed: 2/8/2018 5:24:24 PM by: SCSIO Page 1 of 1

HR-ESIMS for compound **4**

Mass Spectrum SmartFormula Report

Analysis Info

Analysis Name	D:\Data\MS\data\201802\liwanshan_XG-53-5_neg_55_01_4273.d	Acquisition Date	2/8/2018 5:09:54 PM
Method	LC_Direct Infusion_neg_100-1000mz.m	Operator	SCSIO
Sample Name	liwanshan_XG-53-5_neg	Instrument	maXis 255552.00029
Comment			

Acquisition Parameter

Source Type	ESI	Ion Polarity	Negative	Set Nebulizer	0.4 Bar
Focus	Active	Set Capillary	4000 V	Set Dry Heater	180 °C
Scan Begin	100 m/z	Set End Plate Offset	-500 V	Set Dry Gas	4.0 l/min
Scan End	2000 m/z	Set Charging Voltage	0 V	Set Divert Valve	Waste
		Set Corona	0 nA	Set APCI Heater	0 °C

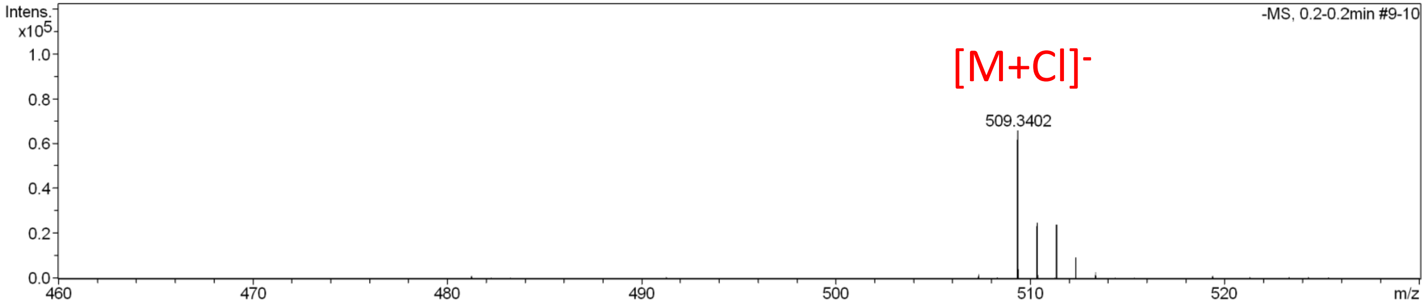

Meas. m/z	#	Ion Formula	Score	m/z	err [ppm]	err [mDa]	mSigma	rdb	e⁻ Conf	N-Rule
509.3402	1	C30H50ClO4	100.00	509.3403	-0.2	-0.1	25.2	5.5	even	ok
983.7100	1	C60H100ClO8	72.88	983.7112	-1.3	-1.2	12.6	10.5	even	ok

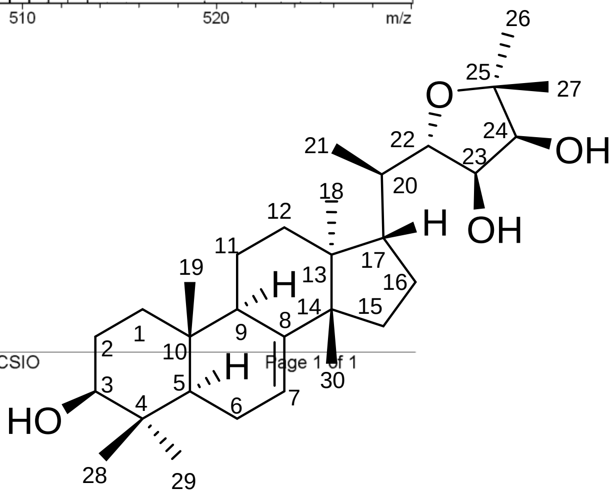

liwanshan_XG-53-5_neg_55_01_4273.d

Bruker Compass DataAnalysis 4.1 printed: 2/8/2018 5:23:39 PM by: SCSIO Page 1 of 1

^{1}H NMR (400 MHz) spectrum of compound **4** in $CDCl_3$

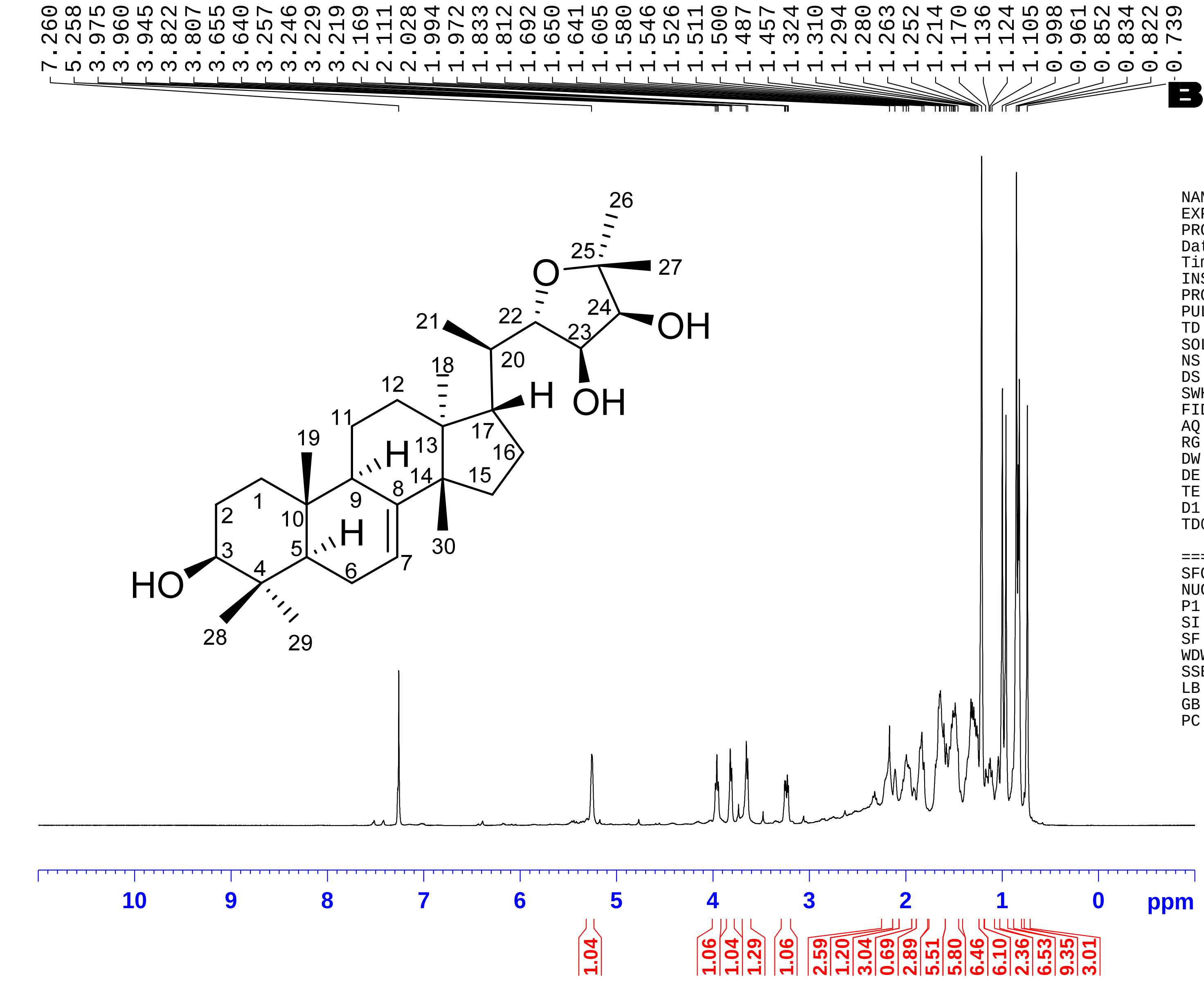

NAME LRX-XG-53-5
EXPNO 1
PROCNO 1
Date_ 20171226
Time 18.20
INSTRUM spect
PROBHD 5 mm CPPBBO BB
PULPROG zg30
TD 65536
SOLVENT CDCl3
NS 16
DS 2
SWH 8223.685 Hz
FIDRES 0.125483 Hz
AQ 3.9846387 sec
RG 85.34
DW 60.800 usec
DE 10.00 usec
TE 297.0 K
D1 1.00000000 sec
TD0 1

======== CHANNEL f1 ========
SFO1 400.1324710 MHz
NUC1 1H
P1 11.50 usec
SI 65536
SF 400.1300094 MHz
WDW EM
SSB 0
LB 0.30 Hz
GB 0
PC 1.00

^{1}H NMR (400 MHz) spectrum of compound **4** in $CDCl_3$

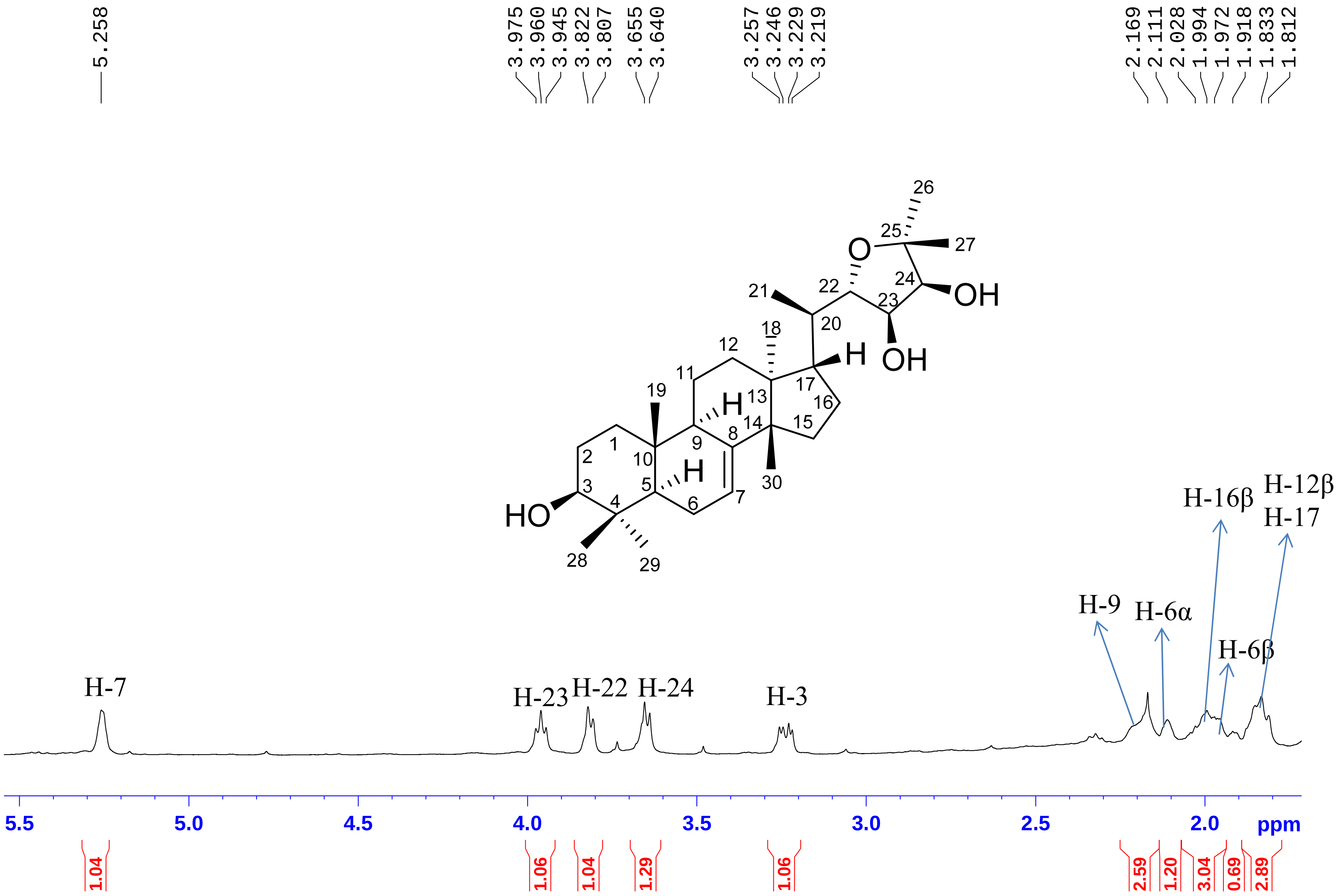

^{1}H NMR (400 MHz) spectrum of compound **4** in $CDCl_3$

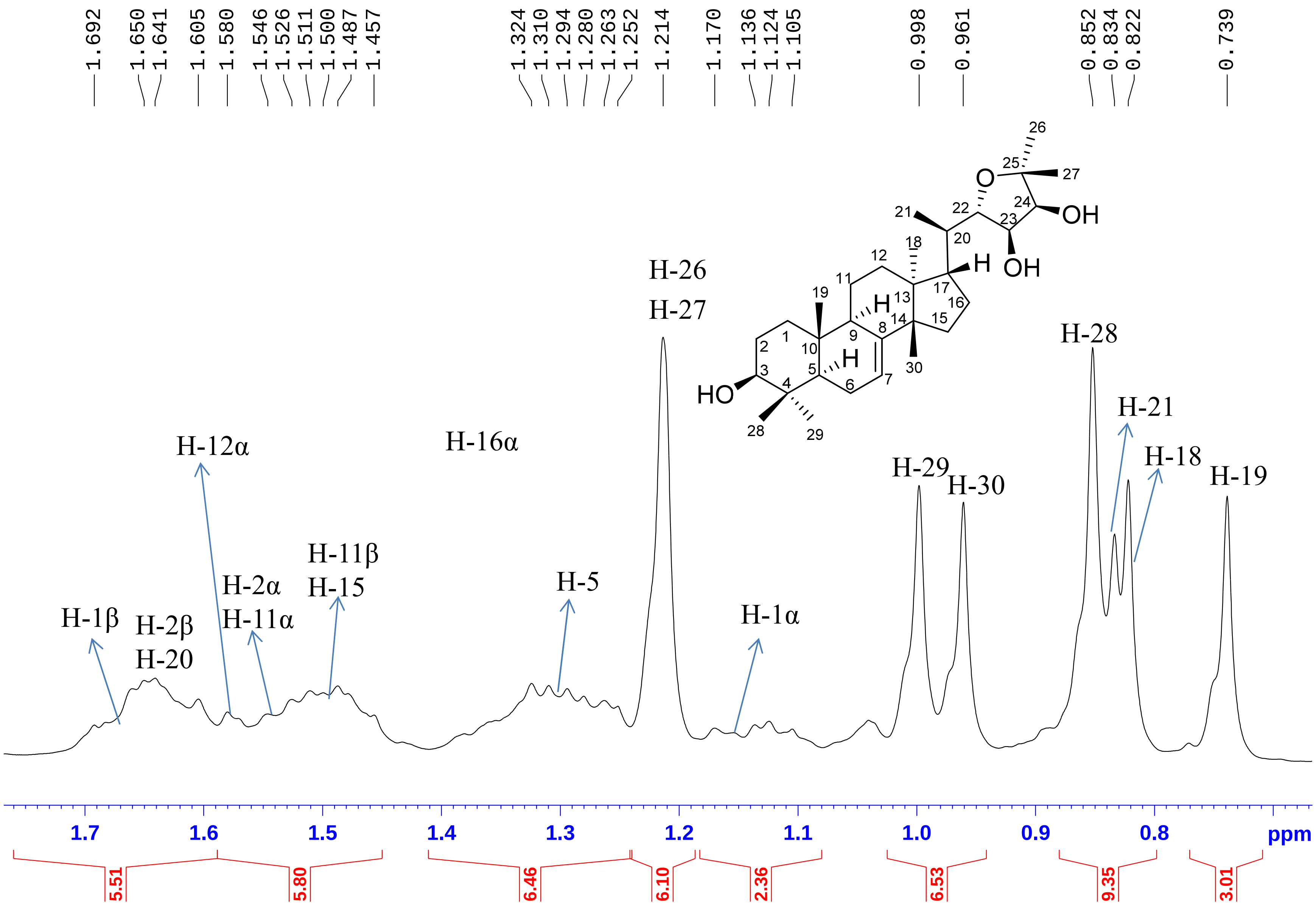

^{13}C NMR (100 MHz) spectrum of compound **4** in $CDCl_3$

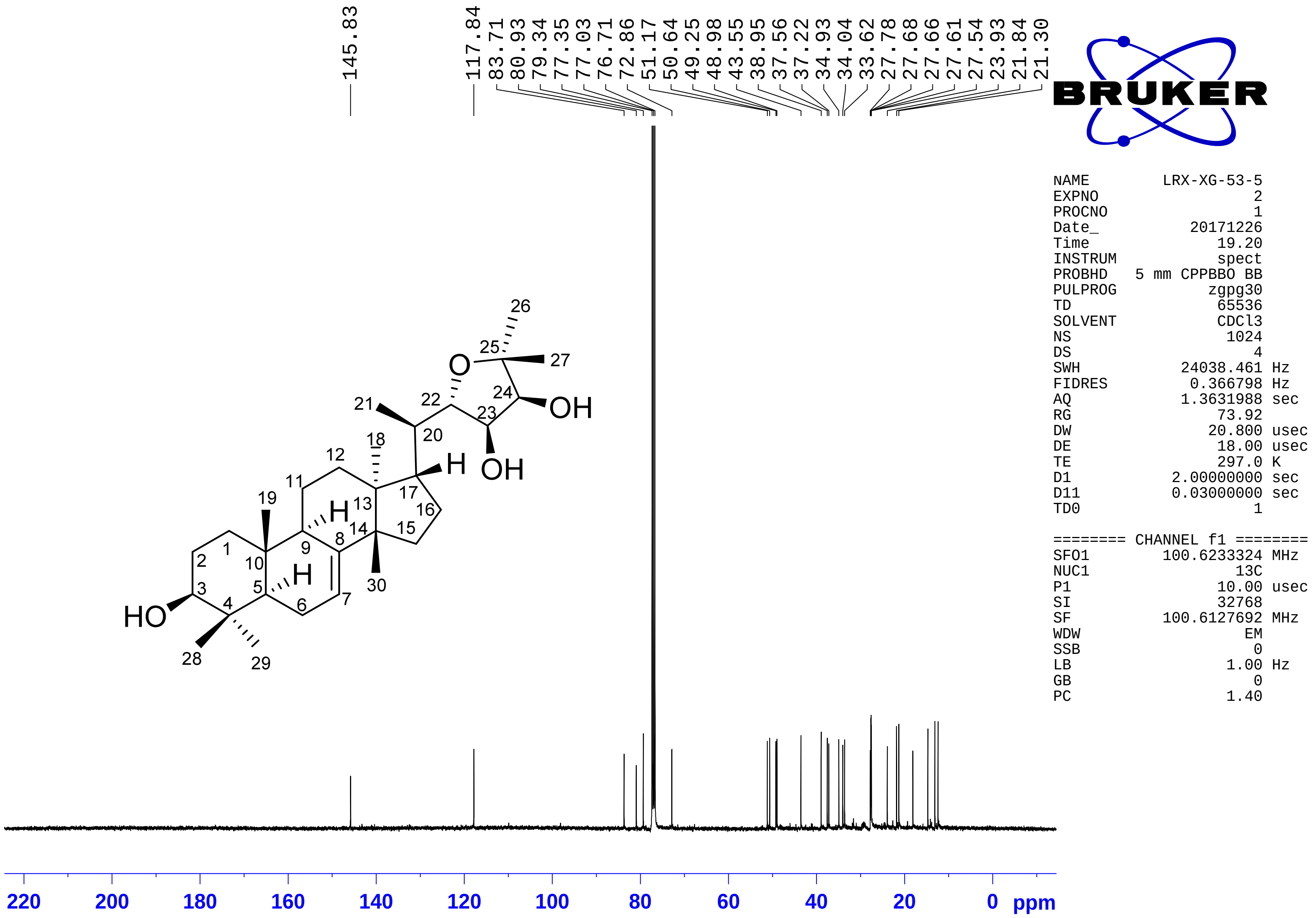

^{13}C NMR (100 MHz) spectrum of compound **4** in $CDCl_3$

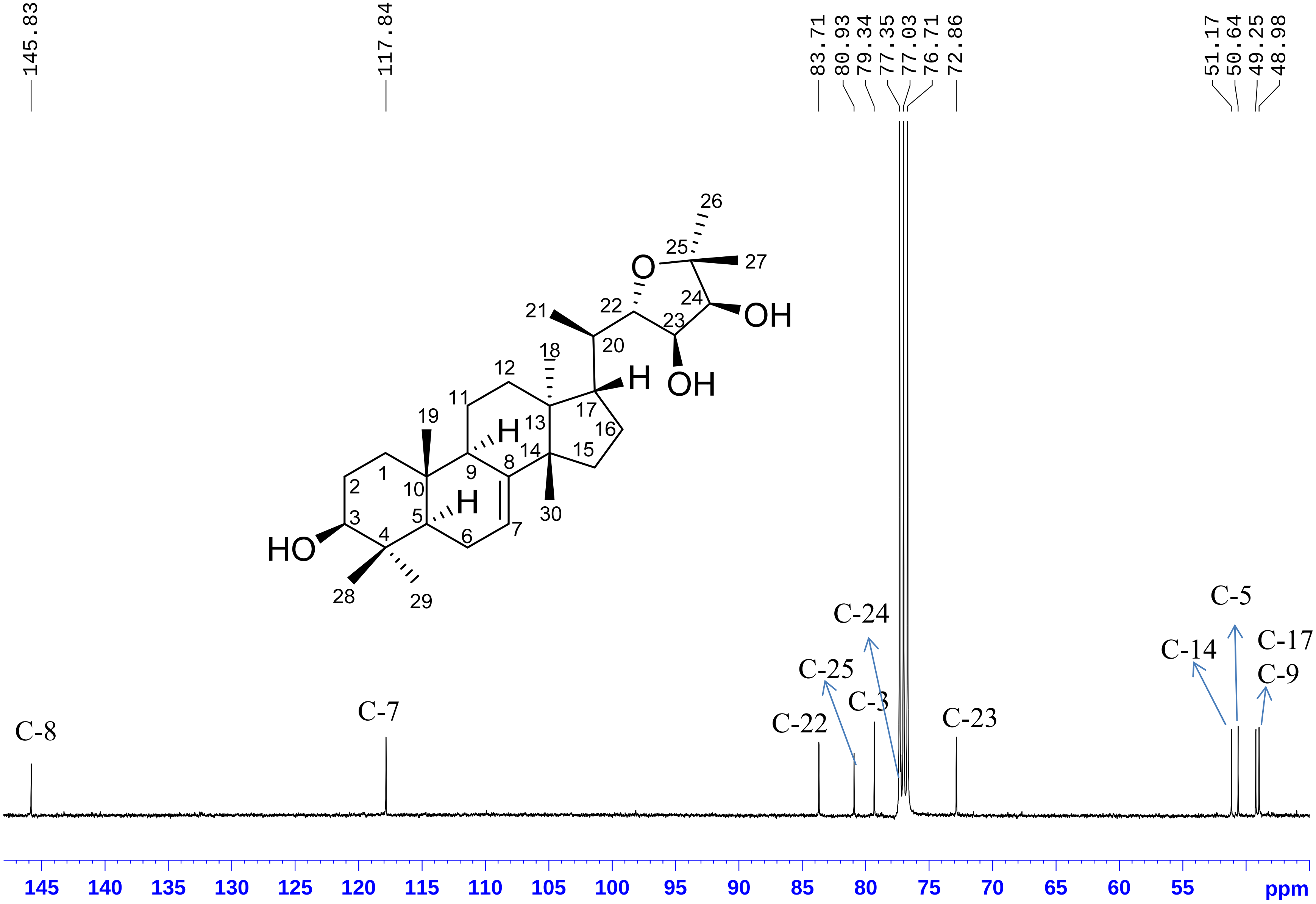

^{13}C NMR (100 MHz) spectrum of compound **4** in $CDCl_3$

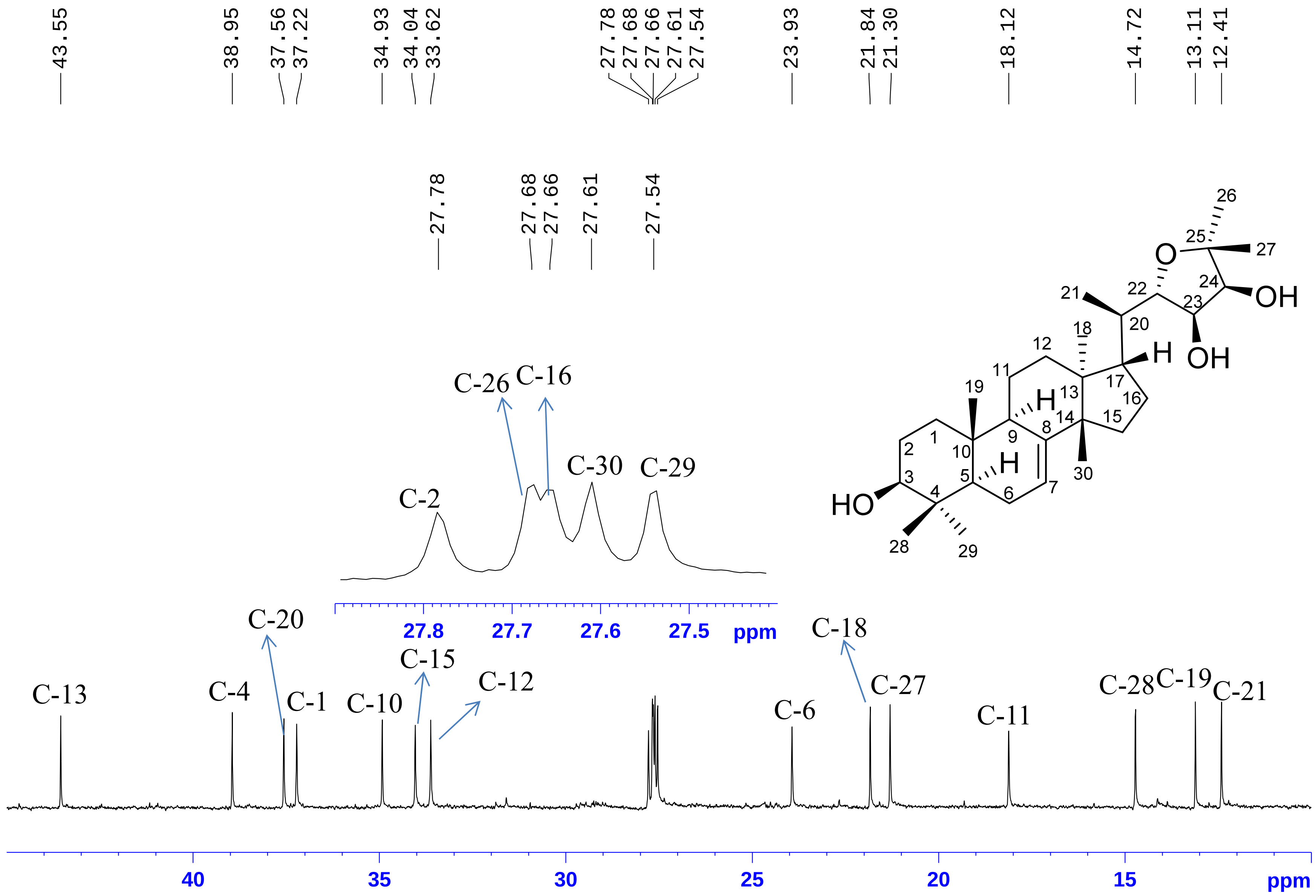

DEPT 135 spectrum of compound **4** in $CDCl_3$

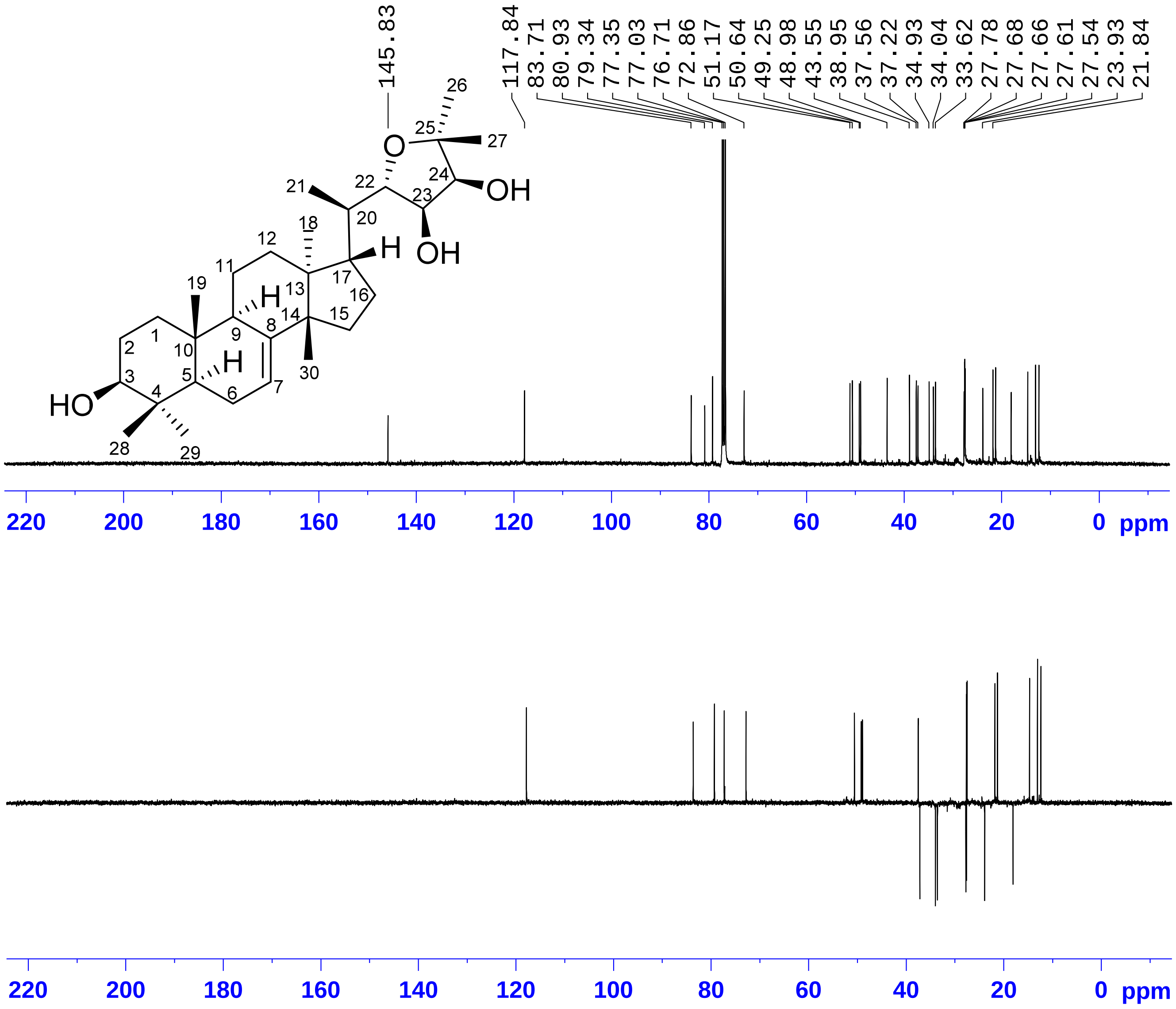


```
NAME          LRX-XG-53-5
EXPNO                   2
PROCNO                  1
Date_            20171226
Time                19.20
INSTRUM             spect
PROBHD   5 mm CPPBBO BB
PULPROG            zgpg30
TD                  65536
SOLVENT             CDCl3
NS                   1024
DS                      4
SWH             24038.461 Hz
FIDRES           0.366798 Hz
AQ              1.3631988 sec
RG                  73.92
DW                 20.800 usec
DE                  18.00 usec
TE                  297.0 K
D1             2.00000000 sec
D11            0.03000000 sec
TD0                     1

======== CHANNEL f1 ========
SFO1          100.6233324 MHz
NUC1                  13C
P1                  10.00 usec
SI                  32768
SF            100.6127692 MHz
WDW                    EM
SSB                     0
LB                   1.00 Hz
GB                      0
PC                   1.40
```

DEPT 135 spectrum of compound **4** in $CDCl_3$

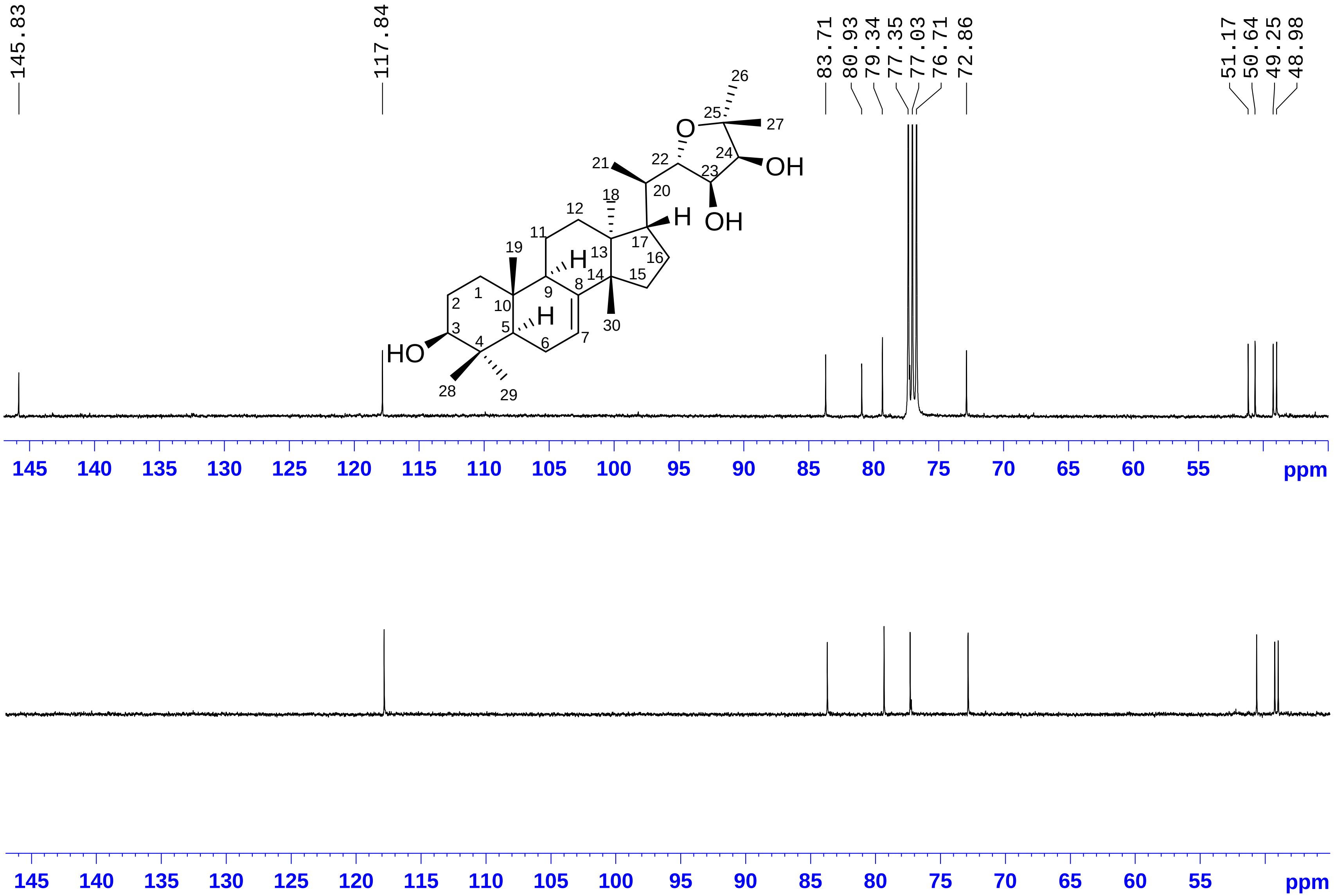

DEPT 135 spectrum of compound **4** in $CDCl_3$

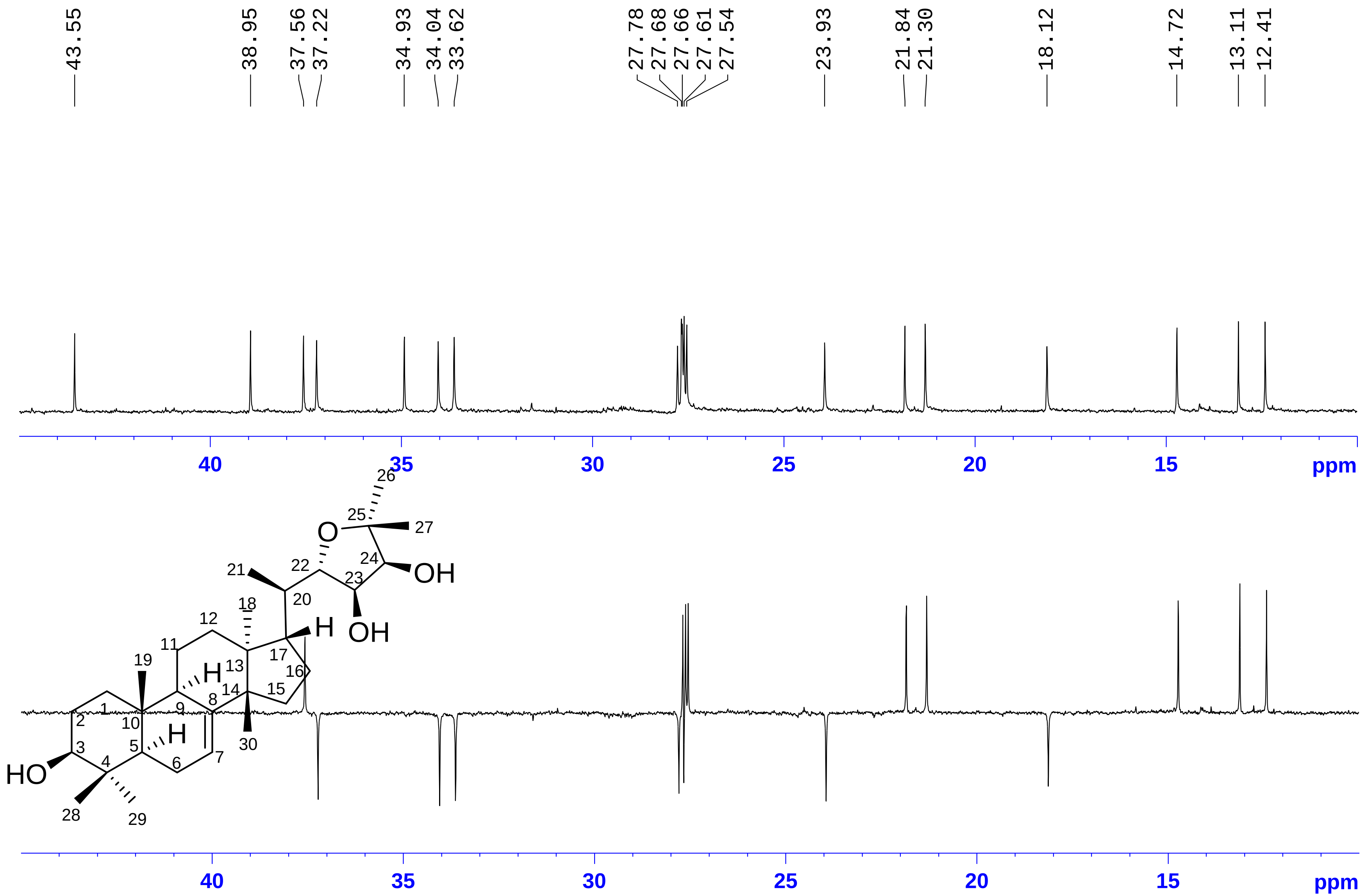

DEPT 135 spectrum of compound **4** in $CDCl_3$

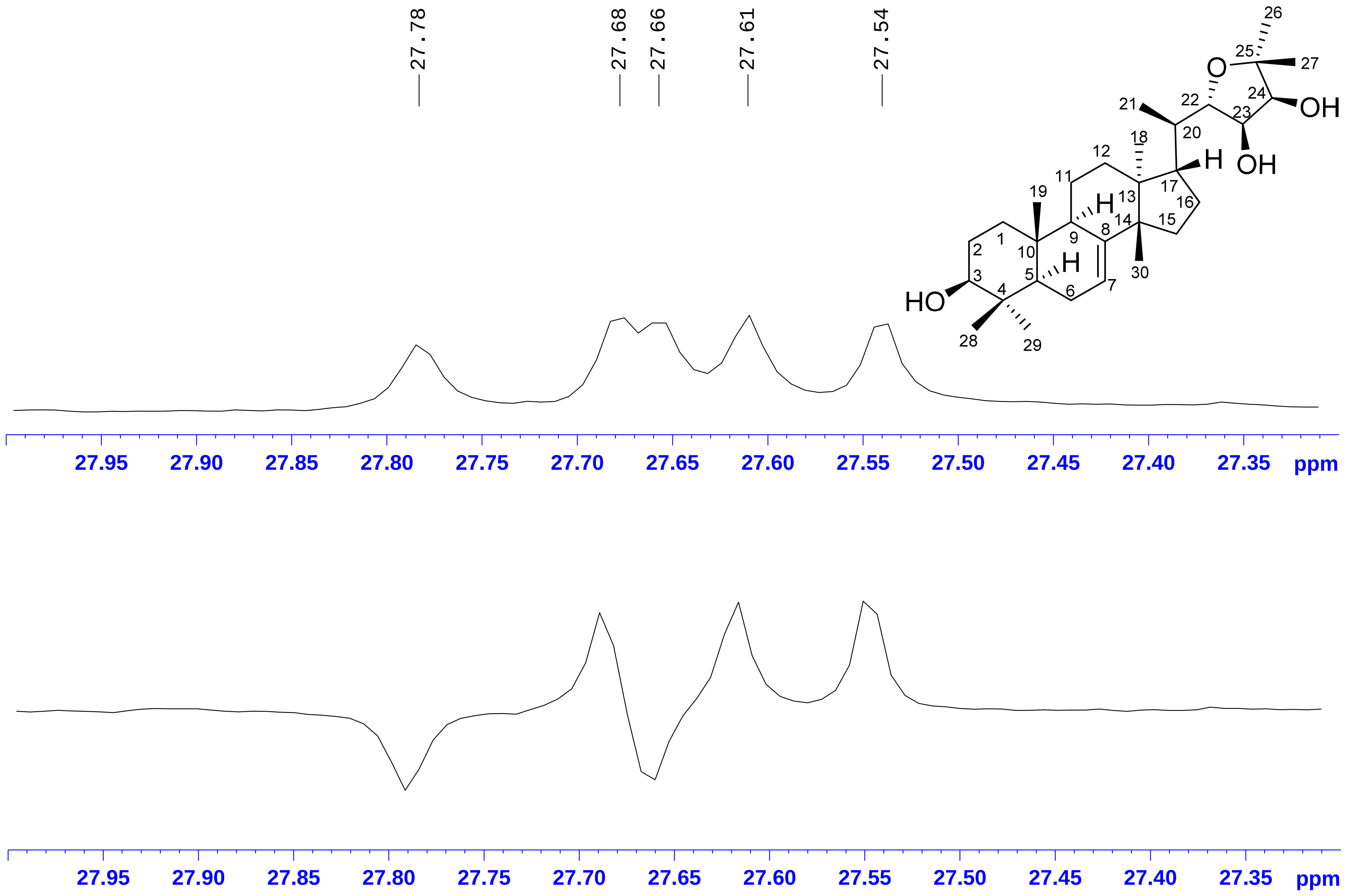

^{1}H-^{1}H COSY (400 MHz) spectrum of compound **4** in $CDCl_3$

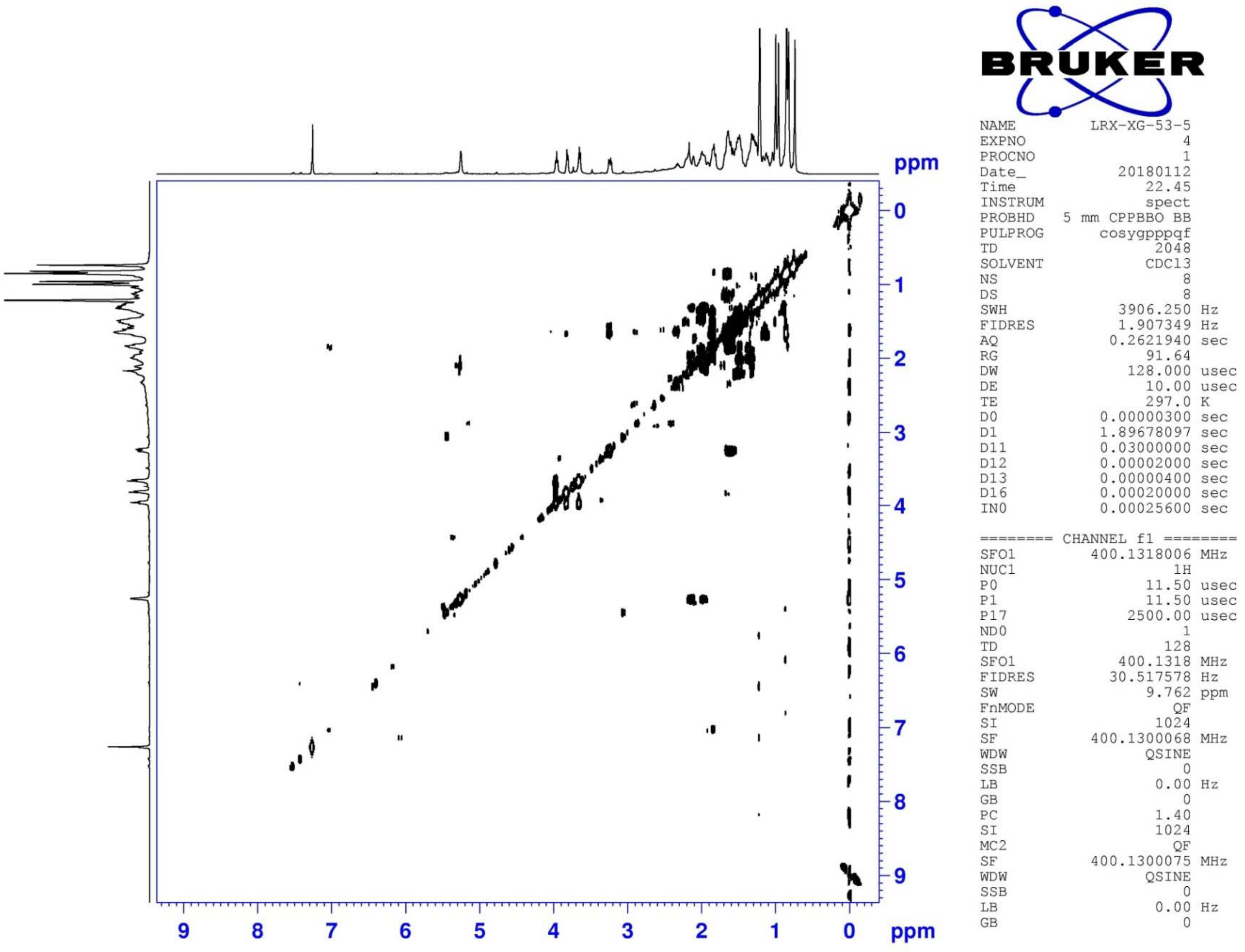

^{1}H-^{1}H COSY (400 MHz) spectrum of compound **4** in $CDCl_3$

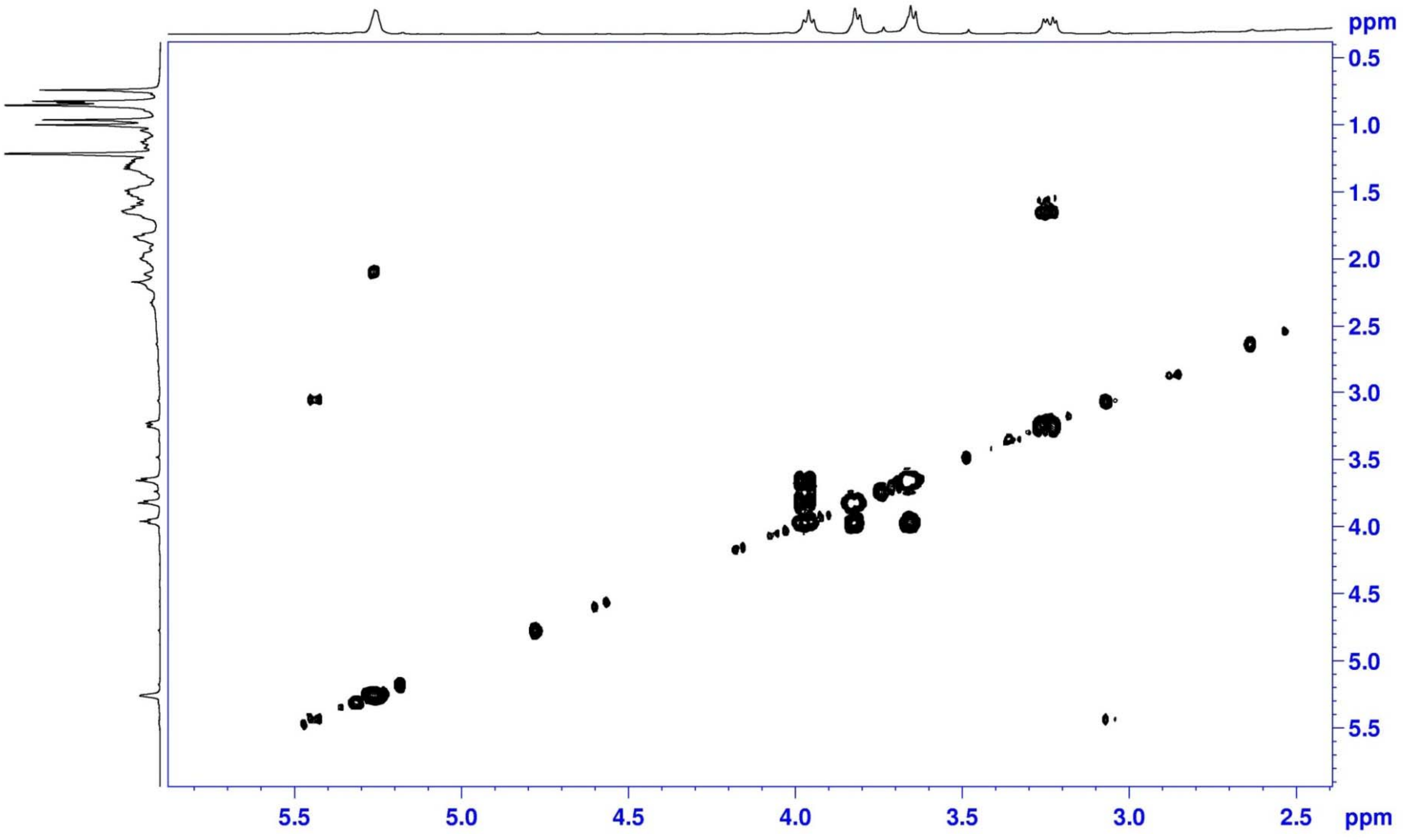

^1H-^1H COSY (400 MHz) spectrum of compound **4** in $CDCl_3$

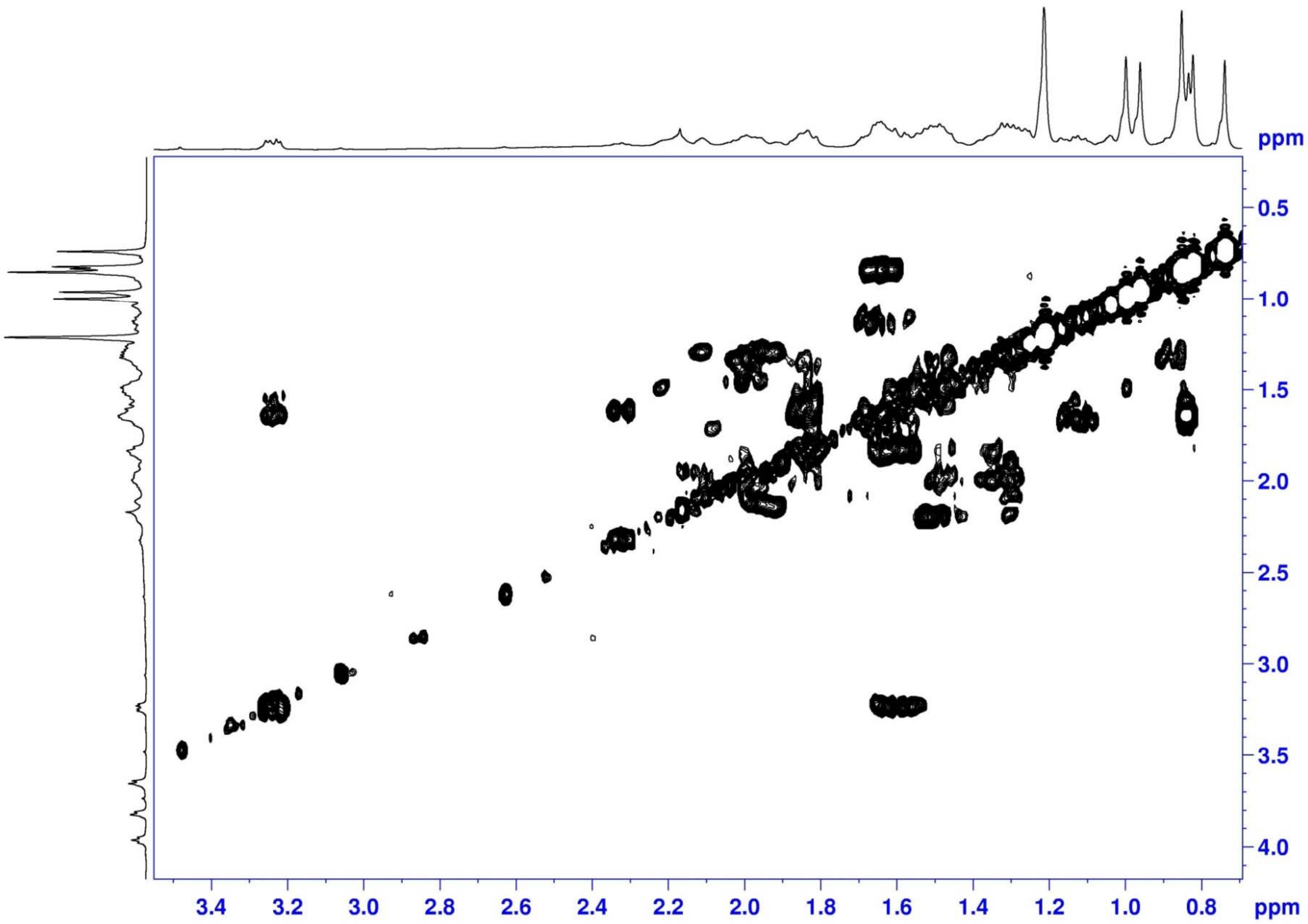

HSQC (400 MHz) spectrum of compound **4** in $CDCl_3$

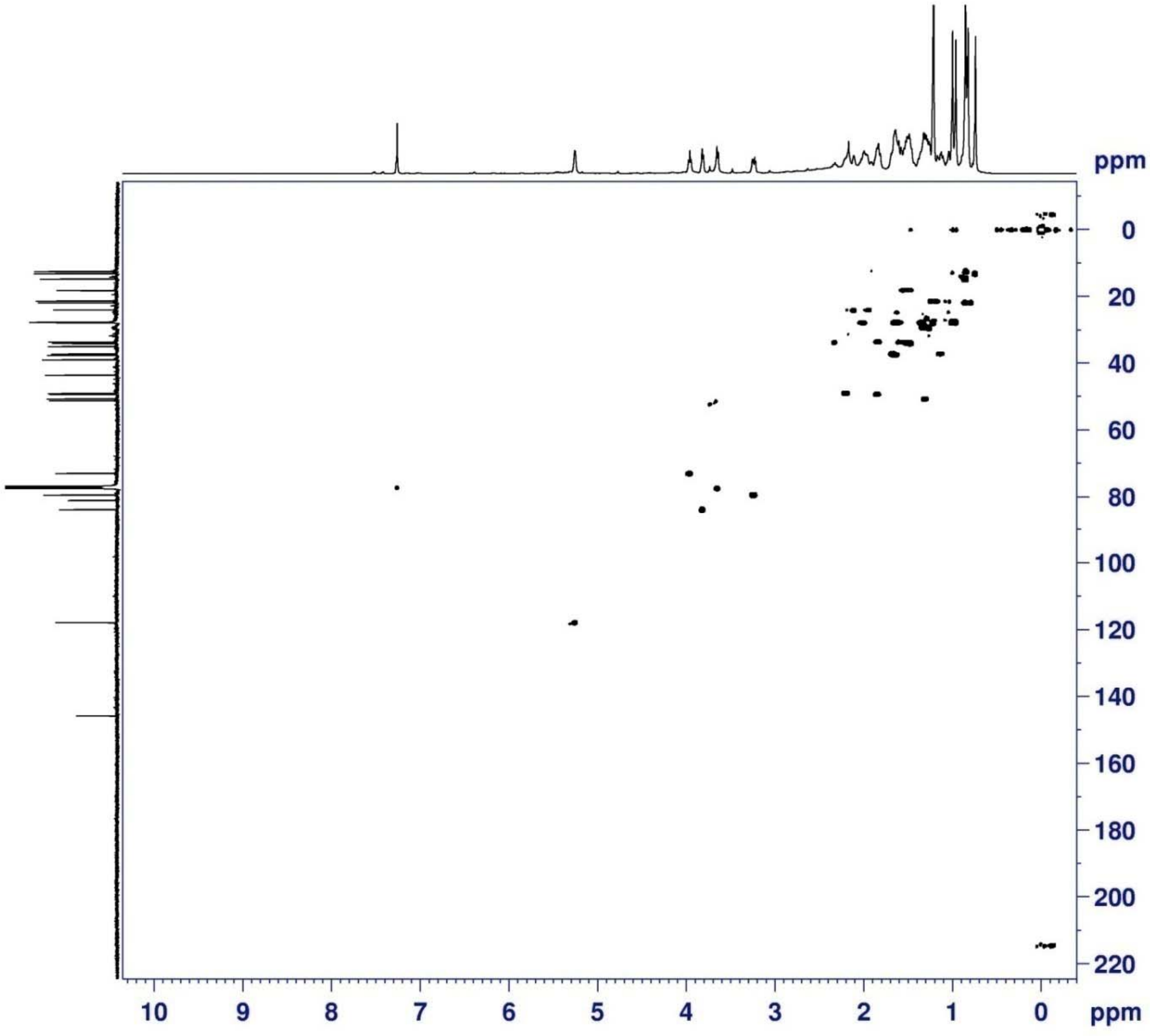


```
NAME          LRX-XG-53-5
EXPNO                   5
PROCNO                  1
Date_            20180112
Time                23.24
INSTRUM             spect
PROBHD   5 mm CPPBBO BB
PULPROG       hsqcetgpsi2
TD                   1024
SOLVENT             CDCl3
NS                     16
DS                     16
SWH              4302.926 Hz
FIDRES           4.202076 Hz
AQ              0.1190388 sec
RG                  208.5
DW                116.200 usec
DE                  10.00 usec
TE                  297.0 K
CNST2         145.0000000
D0             0.00000300 sec
D1             1.46497905 sec
D4             0.00172414 sec
D11            0.03000000 sec
D16            0.00020000 sec
D24            0.00086207 sec
IN0            0.00002080 sec
ZGOPTNS

======== CHANNEL f1 ========
SFO1          400.1320007 MHz
NUC1                   1H
P1                  11.50 usec
P2                  23.00 usec
P28                  0.00 usec
ND0                     2
TD                    256
SFO1             100.6233 MHz
FIDRES          93.900238 Hz
SW                238.896 ppm
FnMODE       Echo-Antiecho
SI                   1024
SF            400.1300095 MHz
WDW                 QSINE
SSB                     2
LB                   0.00 Hz
GB                      0
PC                   1.40
SI                   1024
MC2          echo-antiecho
SF            100.6127551 MHz
WDW                 QSINE
SSB                     2
```

HSQC (400 MHz) spectrum of compound **4** in $CDCl_3$

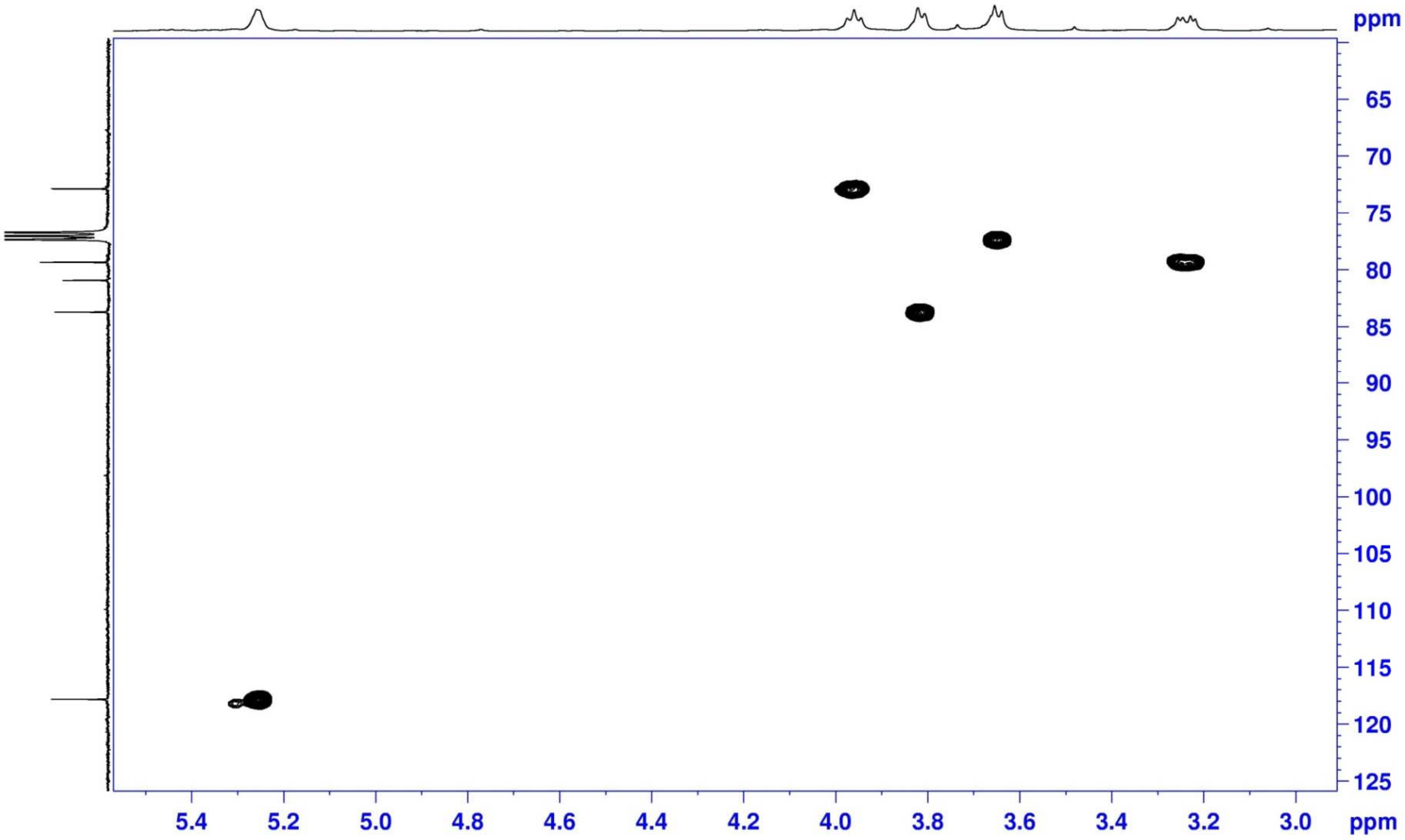

HSQC (400 MHz) spectrum of compound **4** in $CDCl_3$

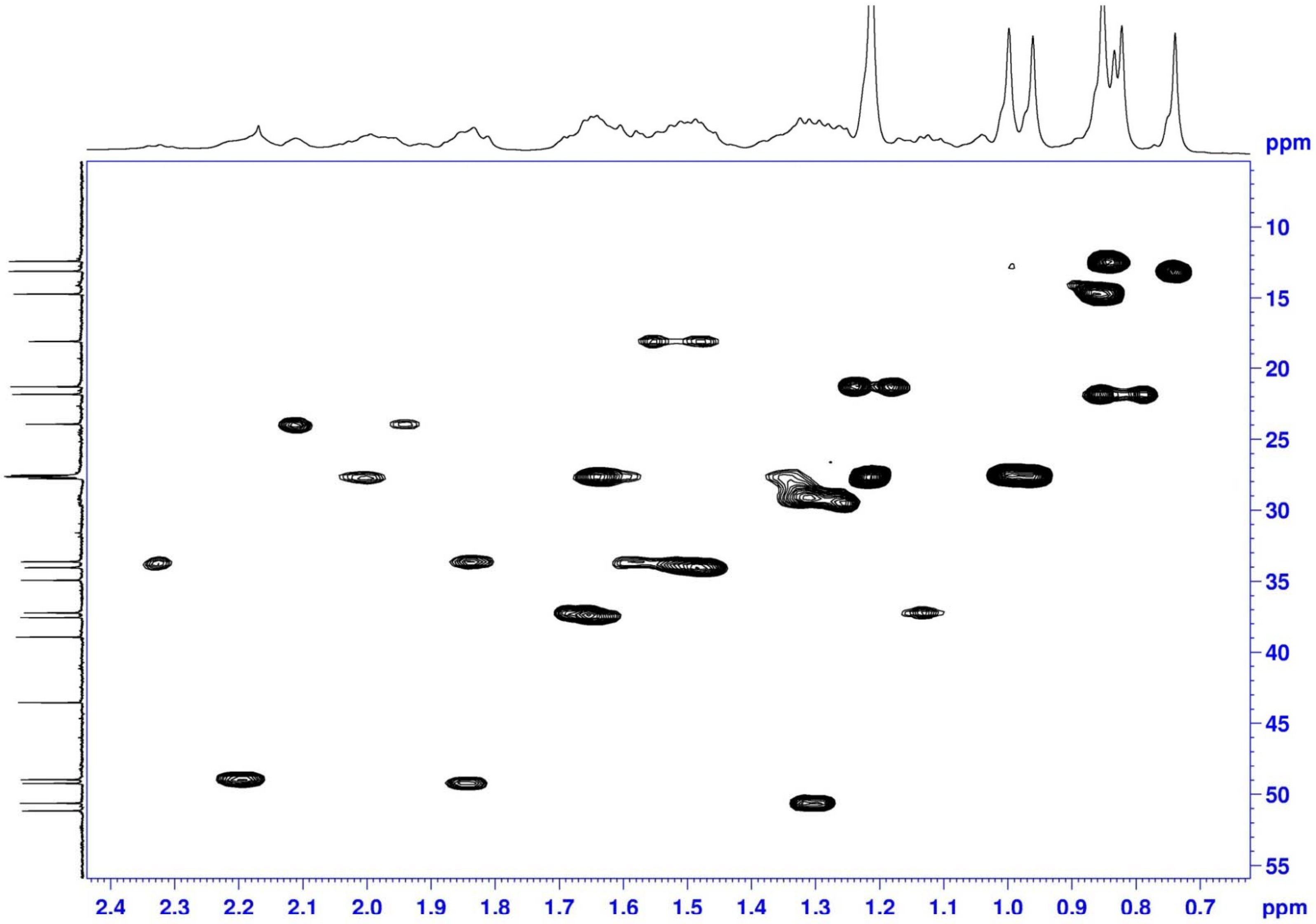

HMBC (400 MHz) spectrum of compound **4** in $CDCl_3$

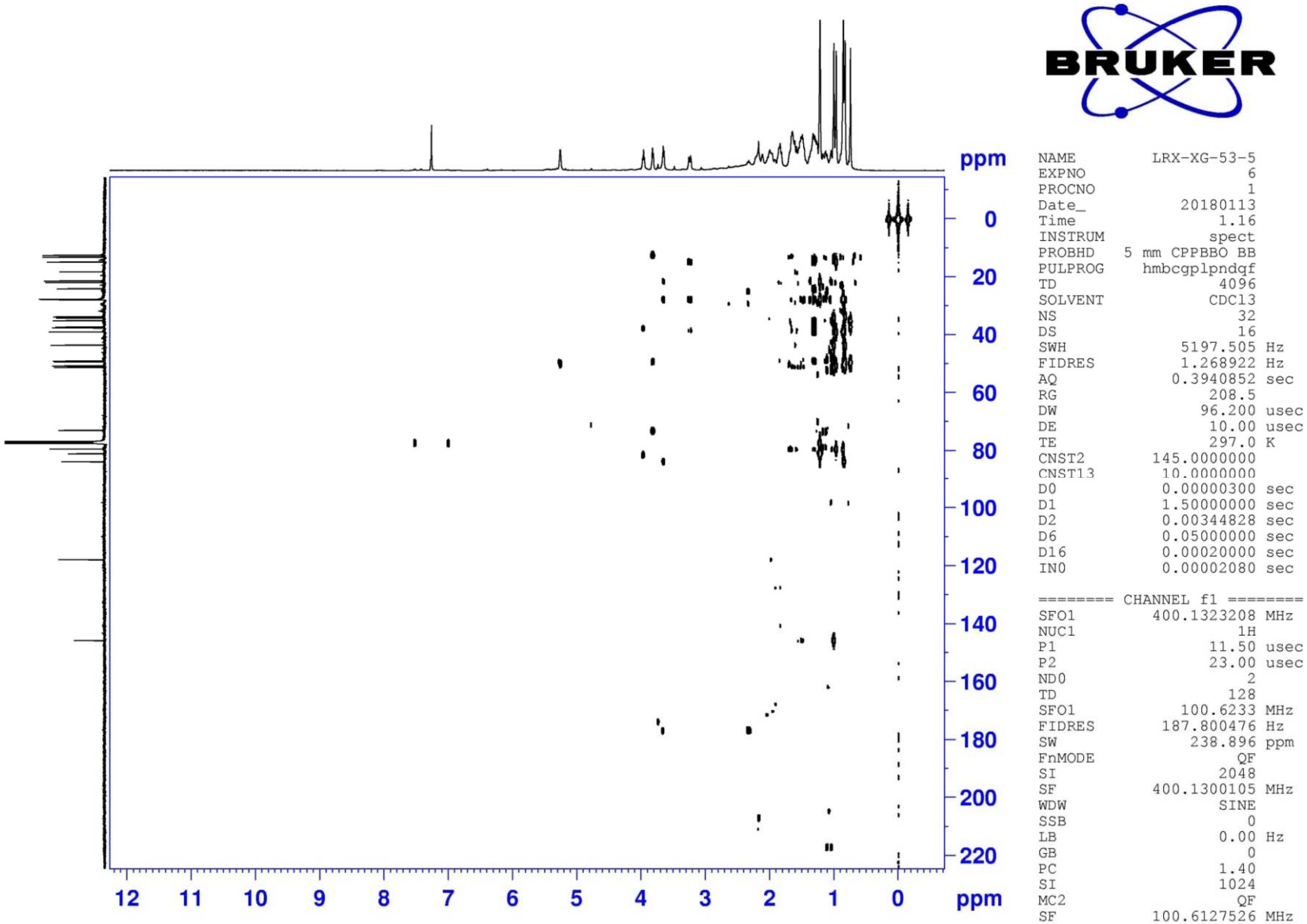


```
NAME          LRX-XG-53-5
EXPNO                   6
PROCNO                  1
Date_            20180113
Time                 1.16
INSTRUM             spect
PROBHD    5 mm CPPBBO BB
PULPROG     hmbcgplpndqf
TD                   4096
SOLVENT             CDCl3
NS                     32
DS                     16
SWH              5197.505 Hz
FIDRES           1.268922 Hz
AQ              0.3940852 sec
RG                  208.5
DW                 96.200 usec
DE                  10.00 usec
TE                  297.0 K
CNST2         145.0000000
CNST13         10.0000000
D0             0.00000300 sec
D1             1.50000000 sec
D2             0.00344828 sec
D6             0.05000000 sec
D16            0.00020000 sec
IN0            0.00002080 sec

======== CHANNEL f1 ========
SFO1          400.1323208 MHz
NUC1                   1H
P1                  11.50 usec
P2                  23.00 usec
ND0                     2
TD                    128
SFO1          100.6233 MHz
FIDRES         187.800476 Hz
SW                238.896 ppm
FnMODE                 QF
SI                   2048
SF            400.1300105 MHz
WDW                  SINE
SSB                     0
LB                   0.00 Hz
GB                      0
PC                   1.40
SI                   1024
MC2                    QF
SF            100.6127526 MHz
WDW                  SINE
SSB                     0
```

HMBC (400 MHz) spectrum of compound **4** in $CDCl_3$

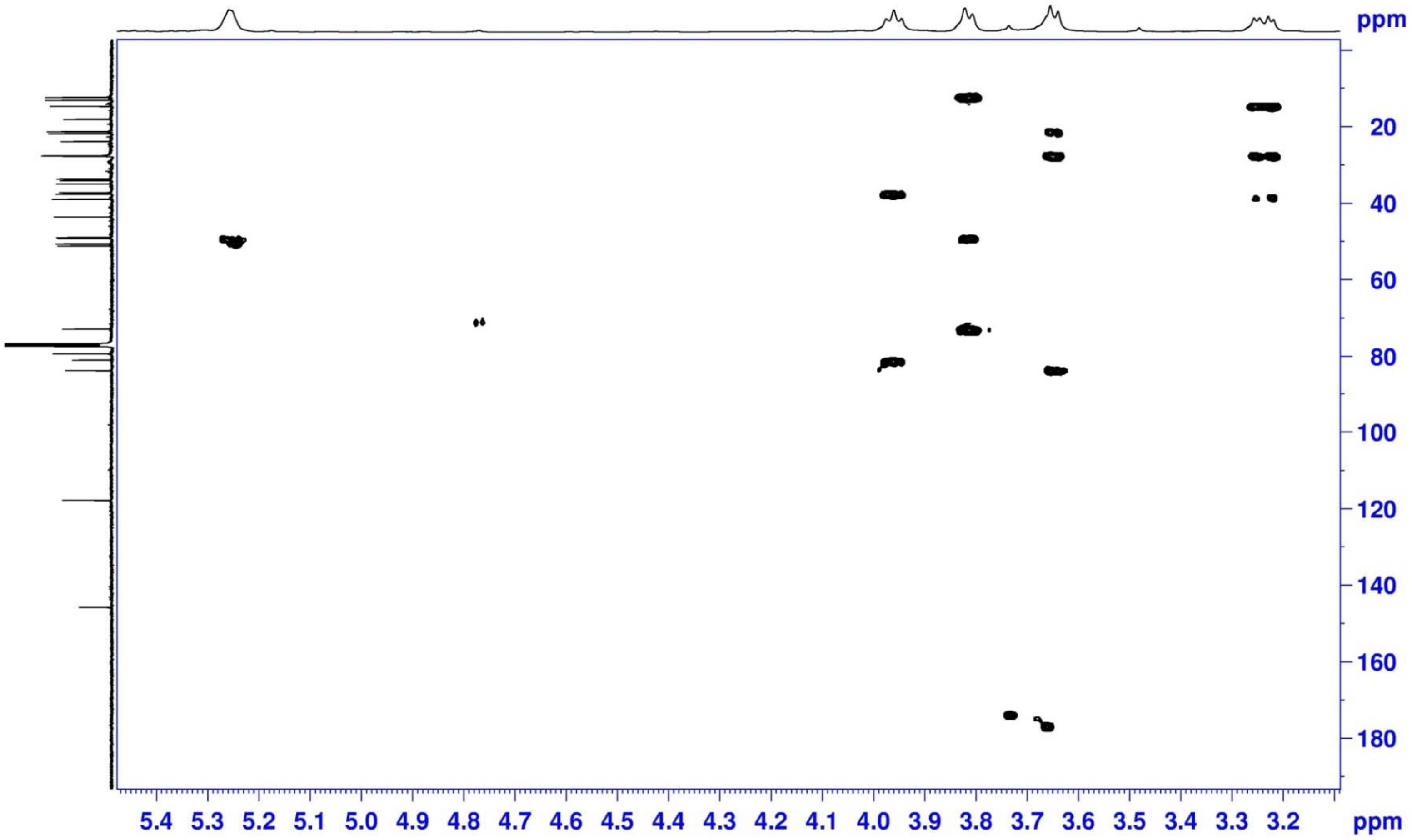

HMBC (400 MHz) spectrum of compound **4** in $CDCl_3$

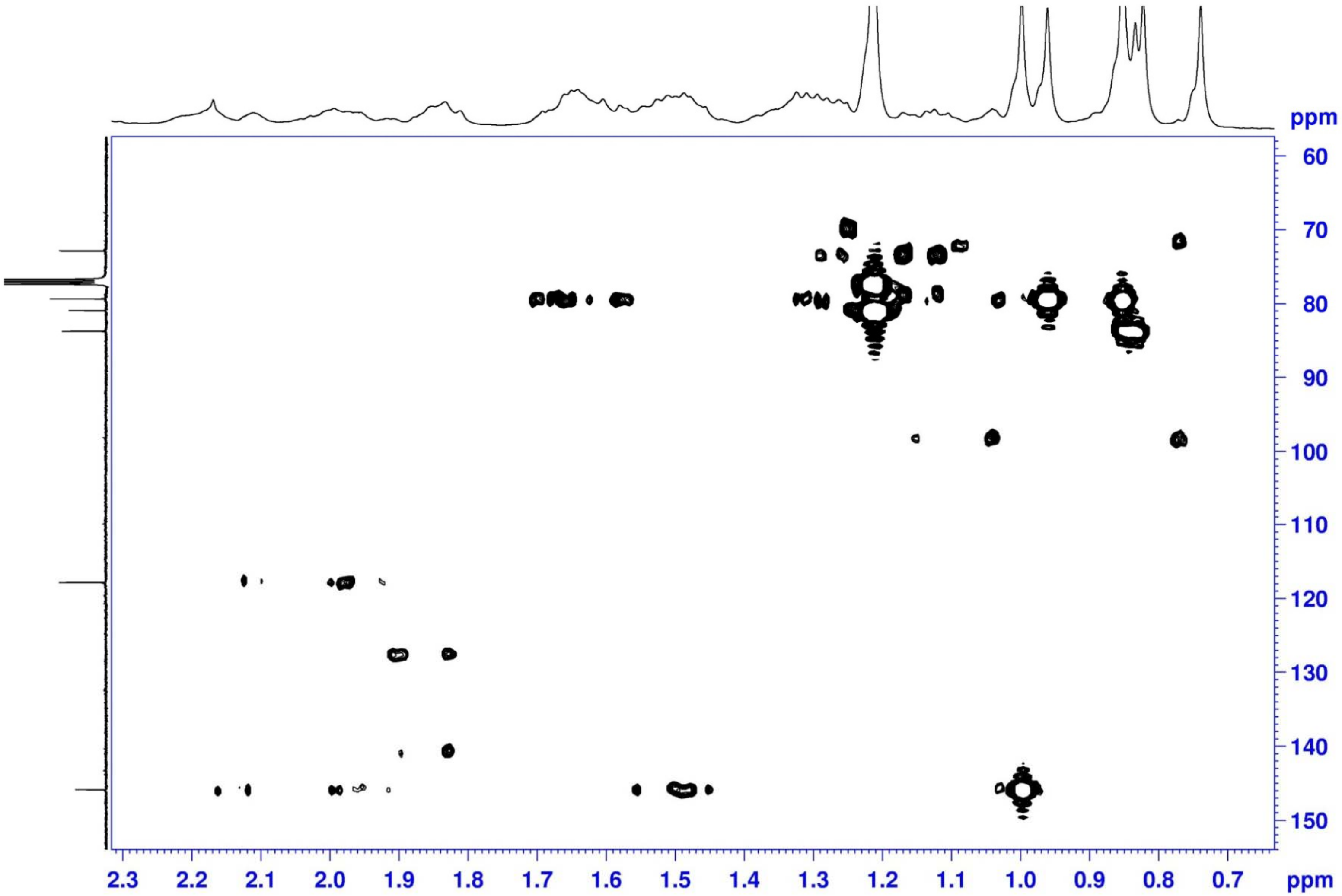

HMBC (400 MHz) spectrum of compound **4** in $CDCl_3$

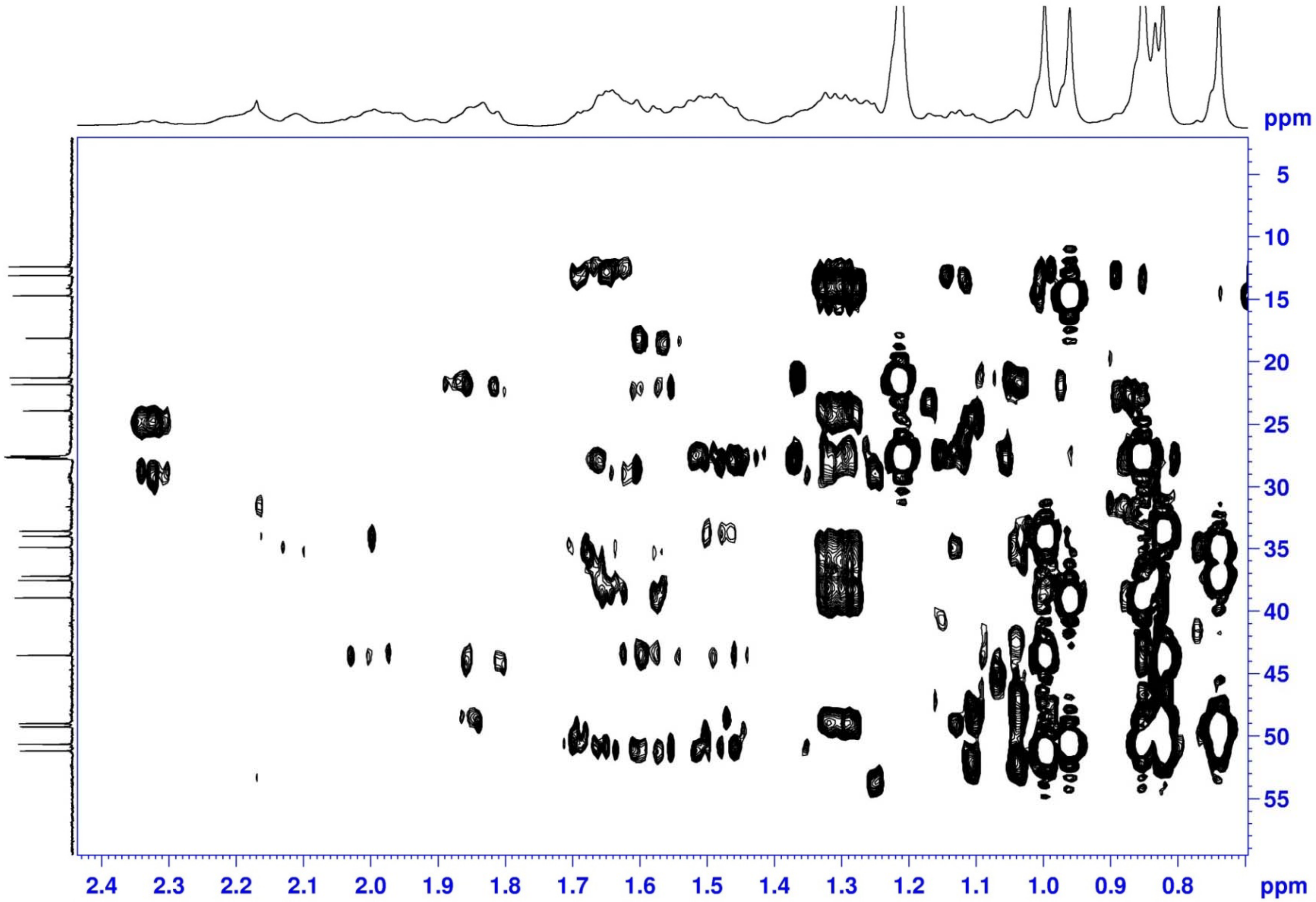

HMBC (400 MHz) spectrum of compound **4** in $CDCl_3$

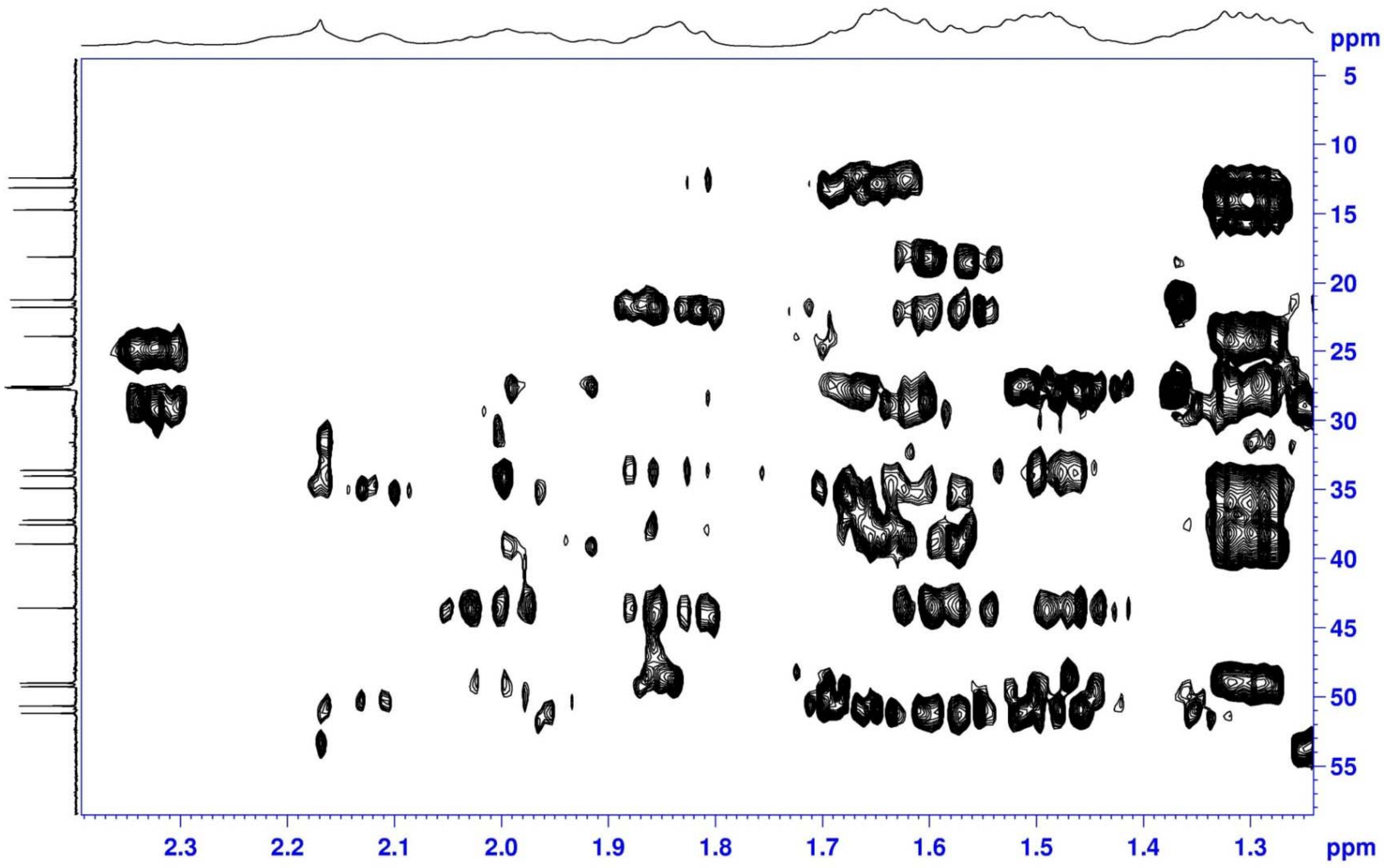

HMBC (400 MHz) spectrum of compound **4** in $CDCl_3$

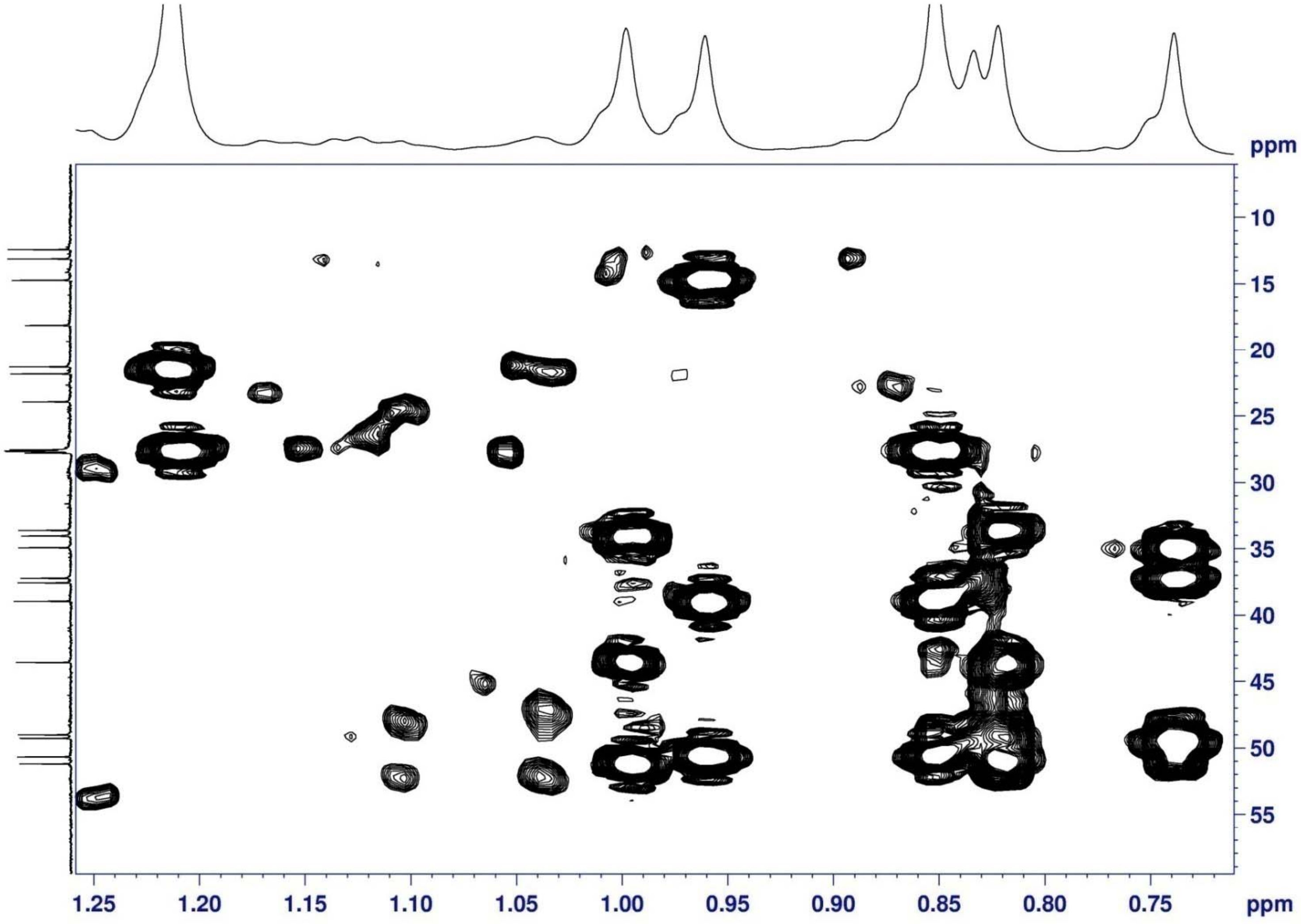

NOESY (400 MHz) spectrum of compound **4** in $CDCl_3$

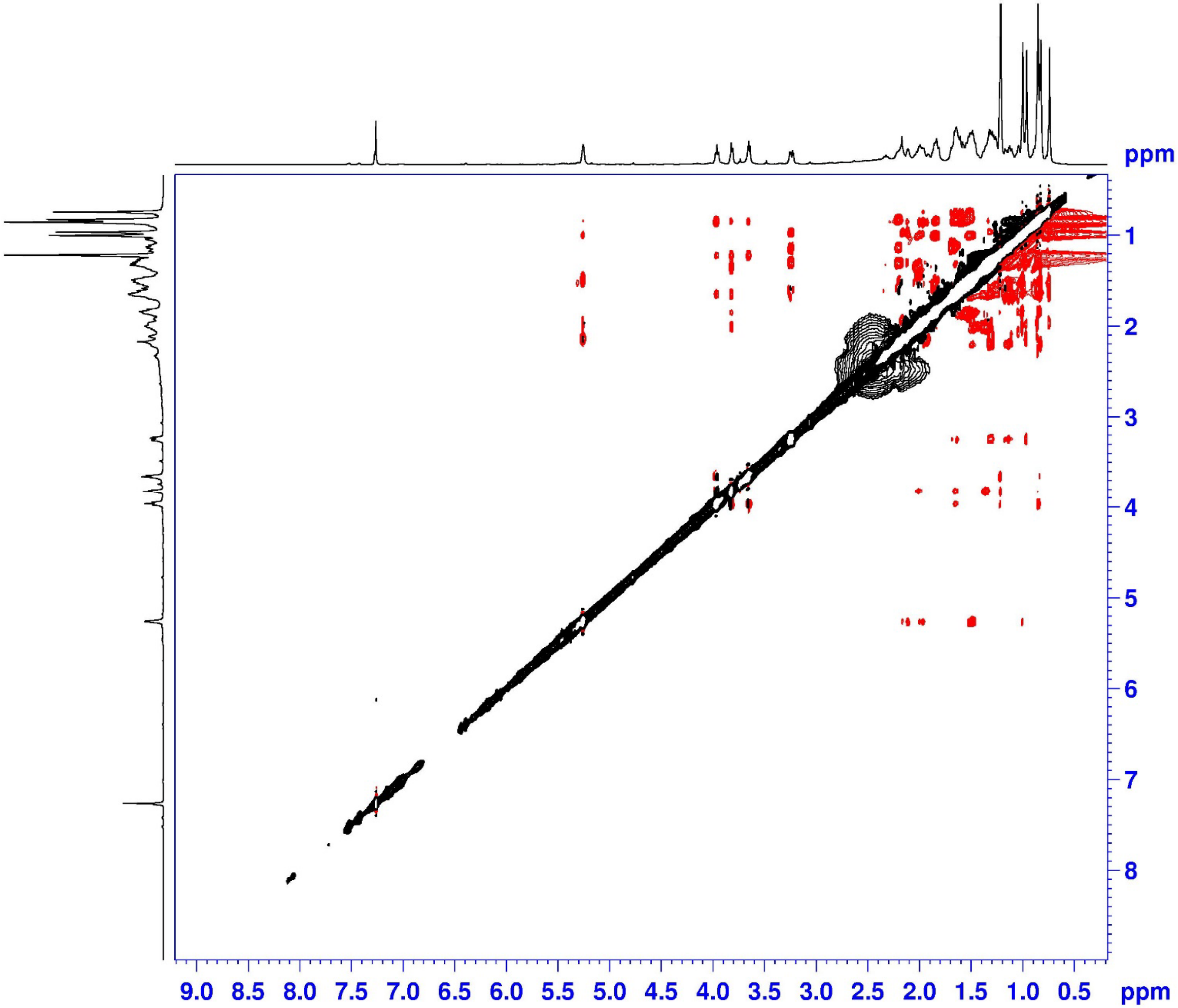

NAME	LRX-XG-53-5
EXPNO	7
PROCNO	1
Date_	20180113
Time	3.31
INSTRUM	spect
PROBHD	5 mm CPPBBO BB
PULPROG	noesygpphpp
TD	2048
SOLVENT	CDCl3
NS	16
DS	32
SWH	4000.000 Hz
FIDRES	1.953125 Hz
AQ	0.2560500 se
RG	91.64
DW	125.000 us
DE	10.00 us
TE	297.0 K
D0	0.00011036 se
D1	1.99385595 se
D8	0.30000001 se
D11	0.03000000 se
D12	0.00002000 se
D16	0.00020000 se
IN0	0.00025000 se

======== CHANNEL f1 ======

SFO1	400.1318006 MH
NUC1	1H
P1	11.50 us
P2	23.00 us
P17	2500.00 us
ND0	1
TD	256
SFO1	400.1318 MH
FIDRES	15.625000 Hz
SW	9.997 pp
FnMODE	States-TPPI
SI	1024
SF	400.1300098 MH
WDW	QSINE
SSB	2
LB	0.00 Hz
GB	0
PC	1.00
SI	1024
MC2	States-TPPI
SF	400.1300098 MH
WDW	QSINE
SSB	2

NOESY (400 MHz) spectrum of compound **4** in $CDCl_3$

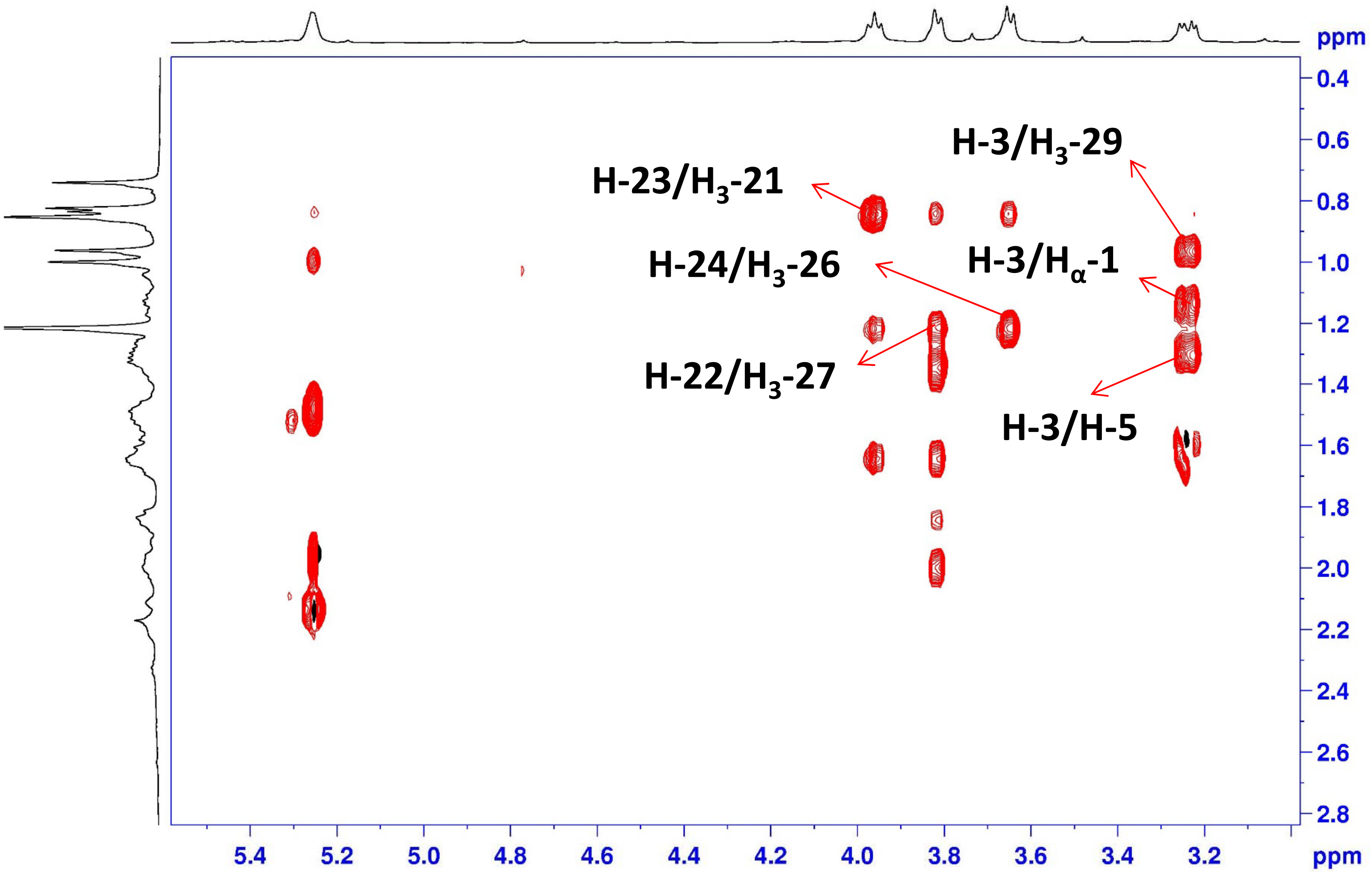

NOESY (400 MHz) spectrum of compound **4** in $CDCl_3$

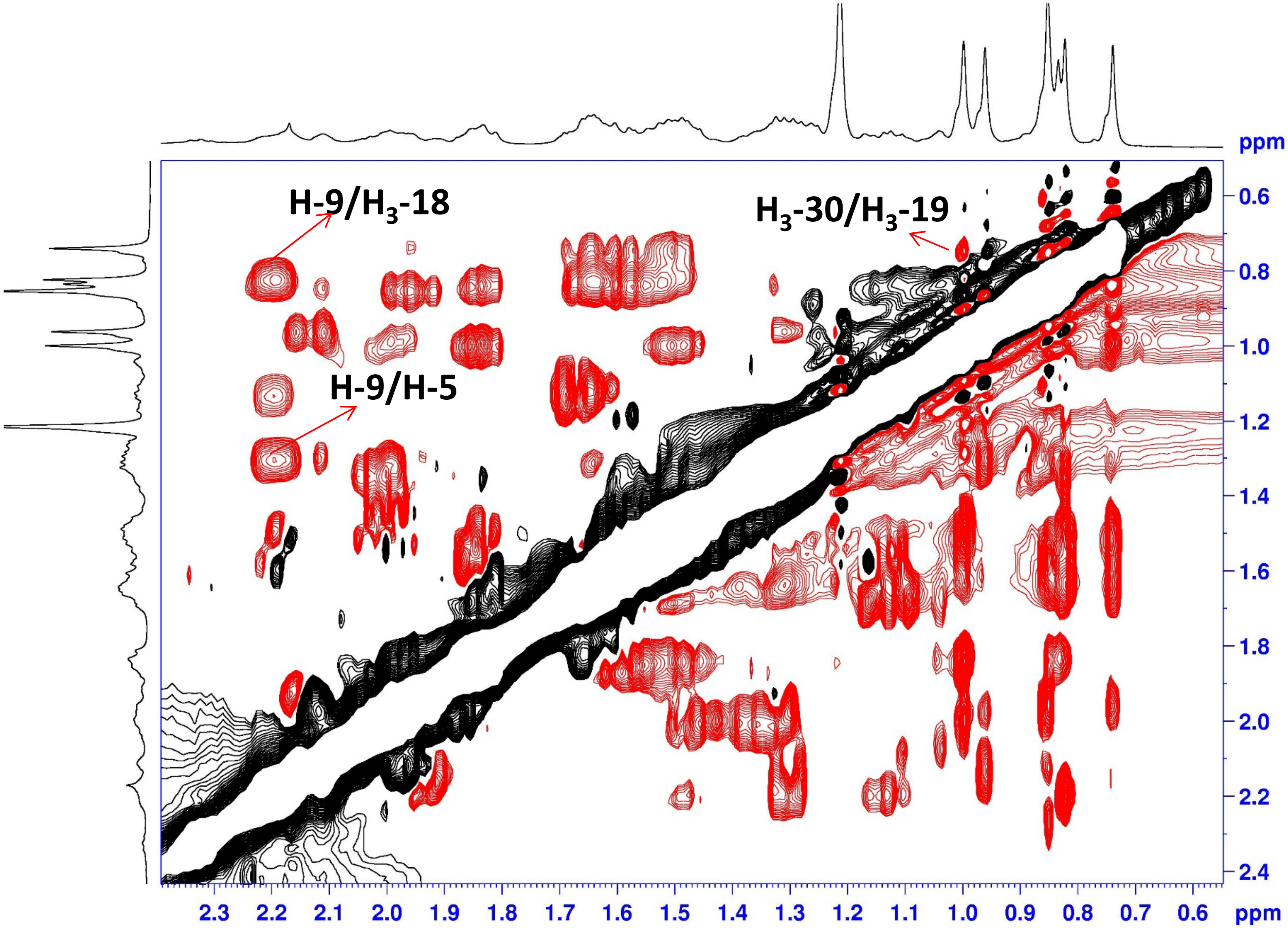

NOESY (400 MHz) spectrum of compound **4** in $CDCl_3$

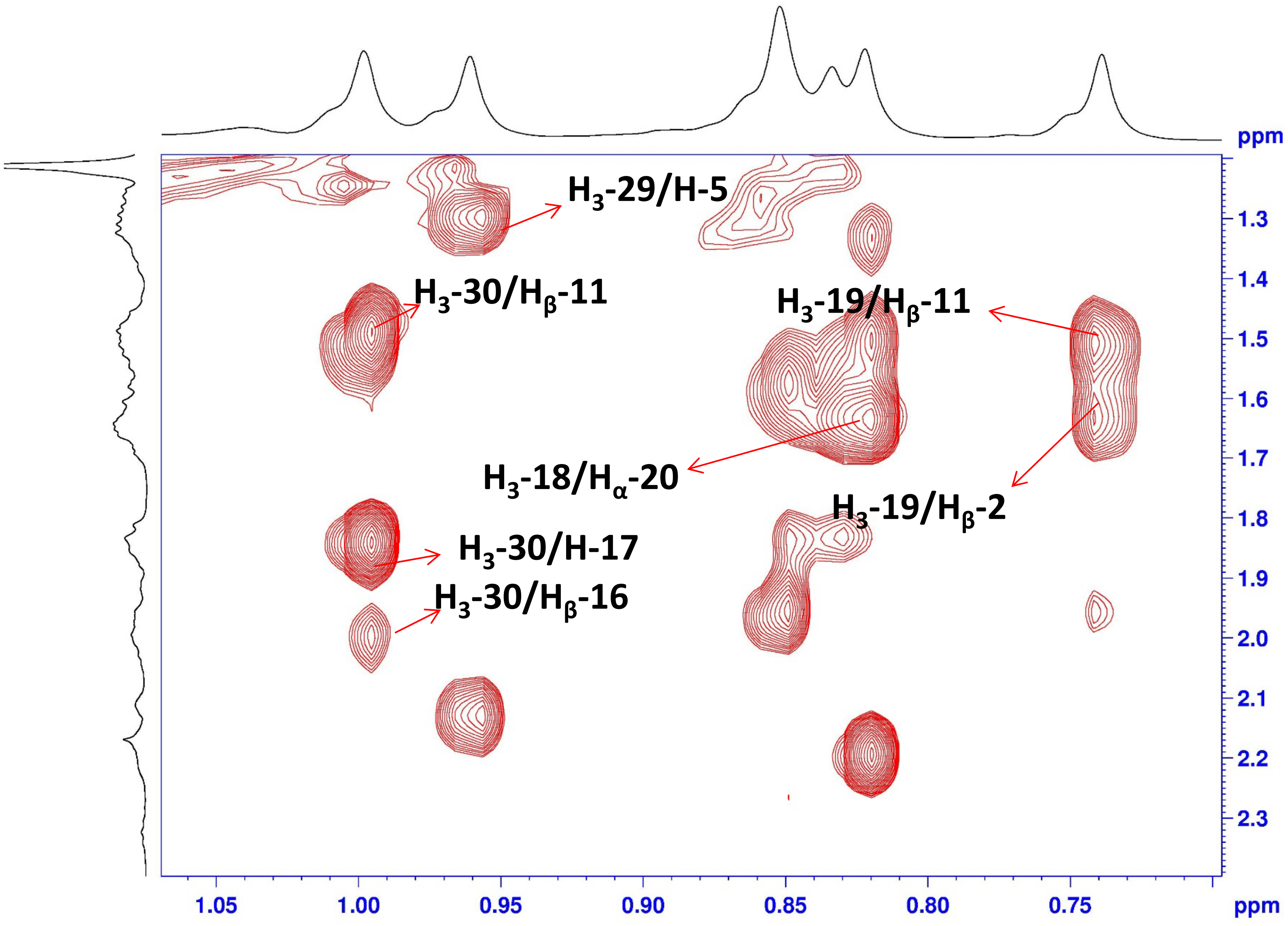

HR-ESIMS for compound **5**

Generic Display Report

**Analysis Info**		Acquisition Date	2/8/2018 5:06:23 PM
Analysis Name	D:\Data\MS\data\201802\liwanshan_XG-52-4_neg_54_01_4272.d		
Method	LC_Direct Infusion_neg_100-1000mz.m	Operator	SCSIO
Sample Name	liwanshan_XG-52-4_neg	Instrument	maXis
Comment			

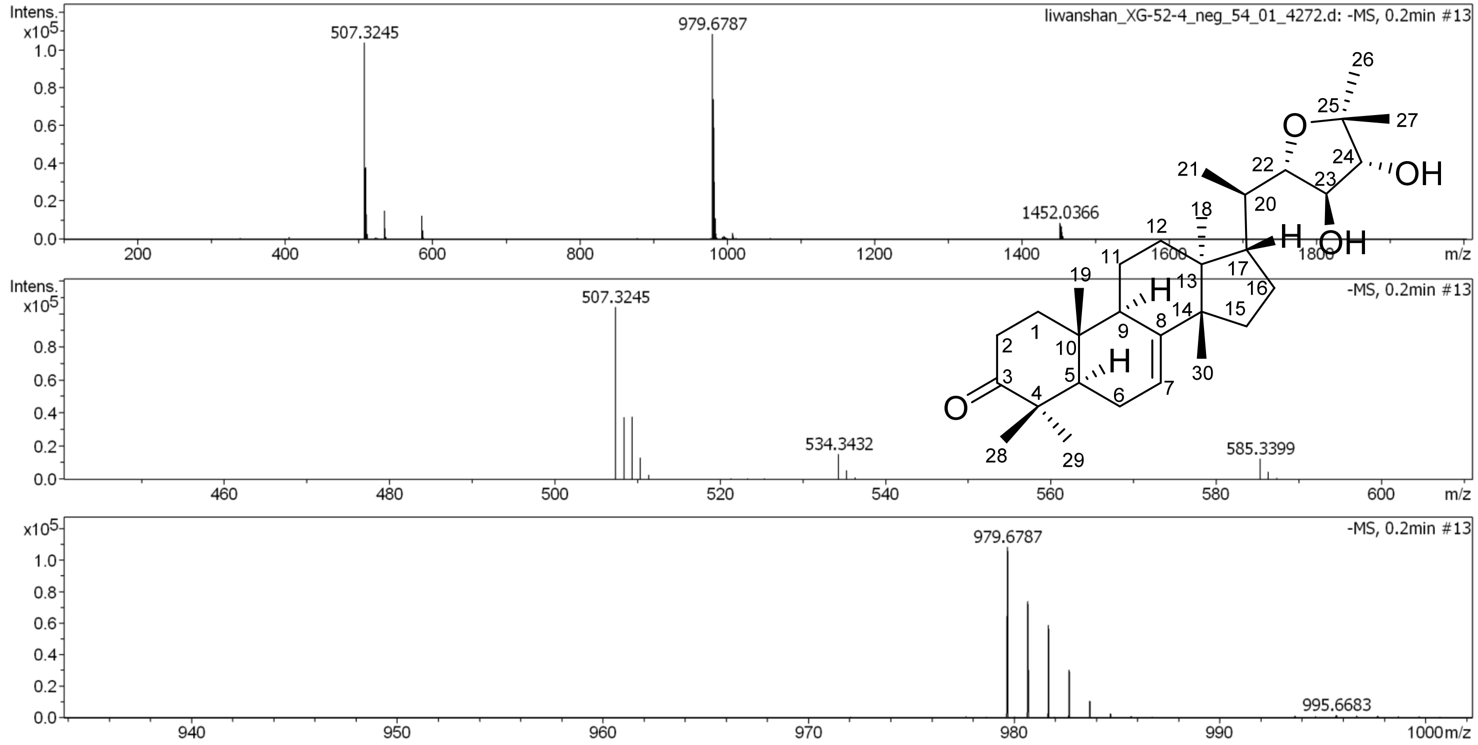

Bruker Compass DataAnalysis 4.1 printed: 2/8/2018 5:21:51 PM by: SCSIO Page 1 of 1

HR-ESIMS for compound **5**

Mass Spectrum SmartFormula Report

Analysis Info

Analysis Name	D:\Data\MS\data\201802\liwanshan_XG-52-4_neg_54_01_4272.d	Acquisition Date	2/8/2018 5:06:23 PM
Method	LC_Direct Infusion_neg_100-1000mz.m	Operator	SCSIO
Sample Name	liwanshan_XG-52-4_neg	Instrument	maXis 255552.00029
Comment			

Acquisition Parameter

Source Type	ESI	Ion Polarity	Negative	Set Nebulizer	0.4 Bar
Focus	Active	Set Capillary	4000 V	Set Dry Heater	180 °C
Scan Begin	100 m/z	Set End Plate Offset	-500 V	Set Dry Gas	4.0 l/min
Scan End	2000 m/z	Set Charging Voltage	0 V	Set Divert Valve	Waste
		Set Corona	0 nA	Set APCI Heater	0 °C

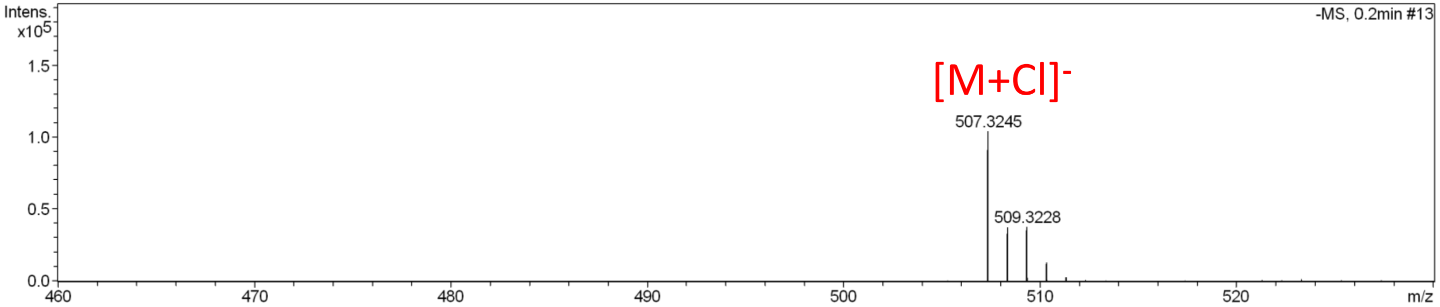

Meas. m/z	#	Ion Formula	Score	m/z	err [ppm]	err [mDa]	mSigma	rdb	e⁻ Conf	N-Rule
507.3245	1	C30H48ClO4	100.00	507.3247	-0.3	-0.2	15.8	6.5	even	ok
979.6787	1	C60H96ClO8	70.91	979.6799	-1.2	-1.2	10.7	12.5	even	ok

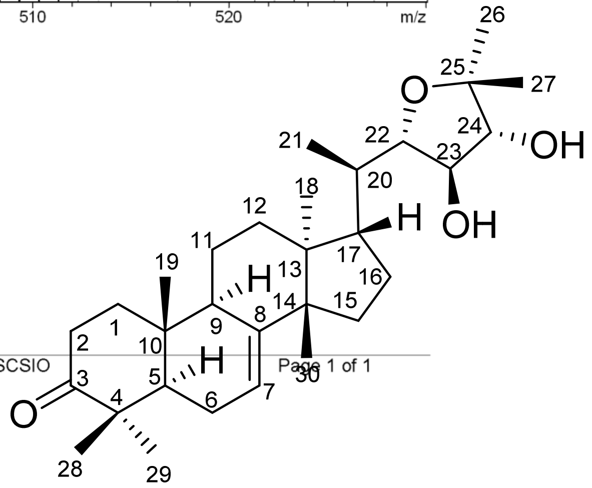

liwanshan_XG-52-4_neg_54_01_4272.d

Bruker Compass DataAnalysis 4.1 printed: 2/8/2018 5:21:18 PM by: SCSIO Page 1 of 1

^{1}H NMR (400 MHz) spectrum of compound **5** in $CDCl_3$

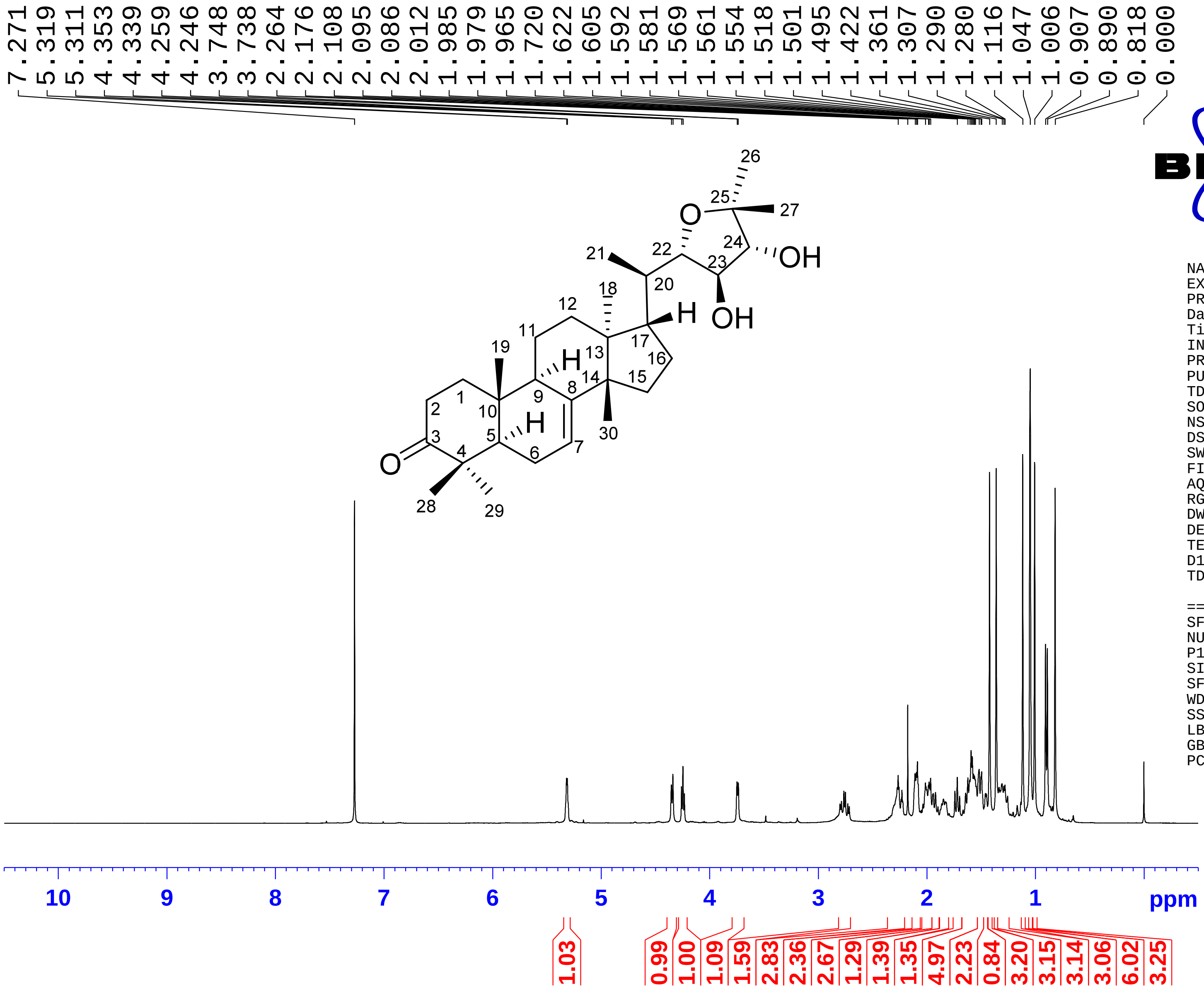


```
NAME           LRX-XG-52-4
EXPNO                    1
PROCNO                   1
Date_             20171221
Time                 20.41
INSTRUM              spect
PROBHD    5 mm CPPBBO BB
PULPROG               zg30
TD                   65536
SOLVENT              CDCl3
NS                      16
DS                       2
SWH               8223.685 Hz
FIDRES            0.125483 Hz
AQ               3.9846387 sec
RG                   41.06
DW                  60.800 usec
DE                   10.00 usec
TE                   297.0 K
D1              1.00000000 sec
TD0                      1

======== CHANNEL f1 ========
SFO1           400.1324710 MHz
NUC1                    1H
P1                   11.50 usec
SI                   65536
SF             400.1300051 MHz
WDW                     EM
SSB                      0
LB                    0.30 Hz
GB                       0
PC                    1.00
```

^{1}H NMR (400 MHz) spectrum of compound **5** in $CDCl_3$

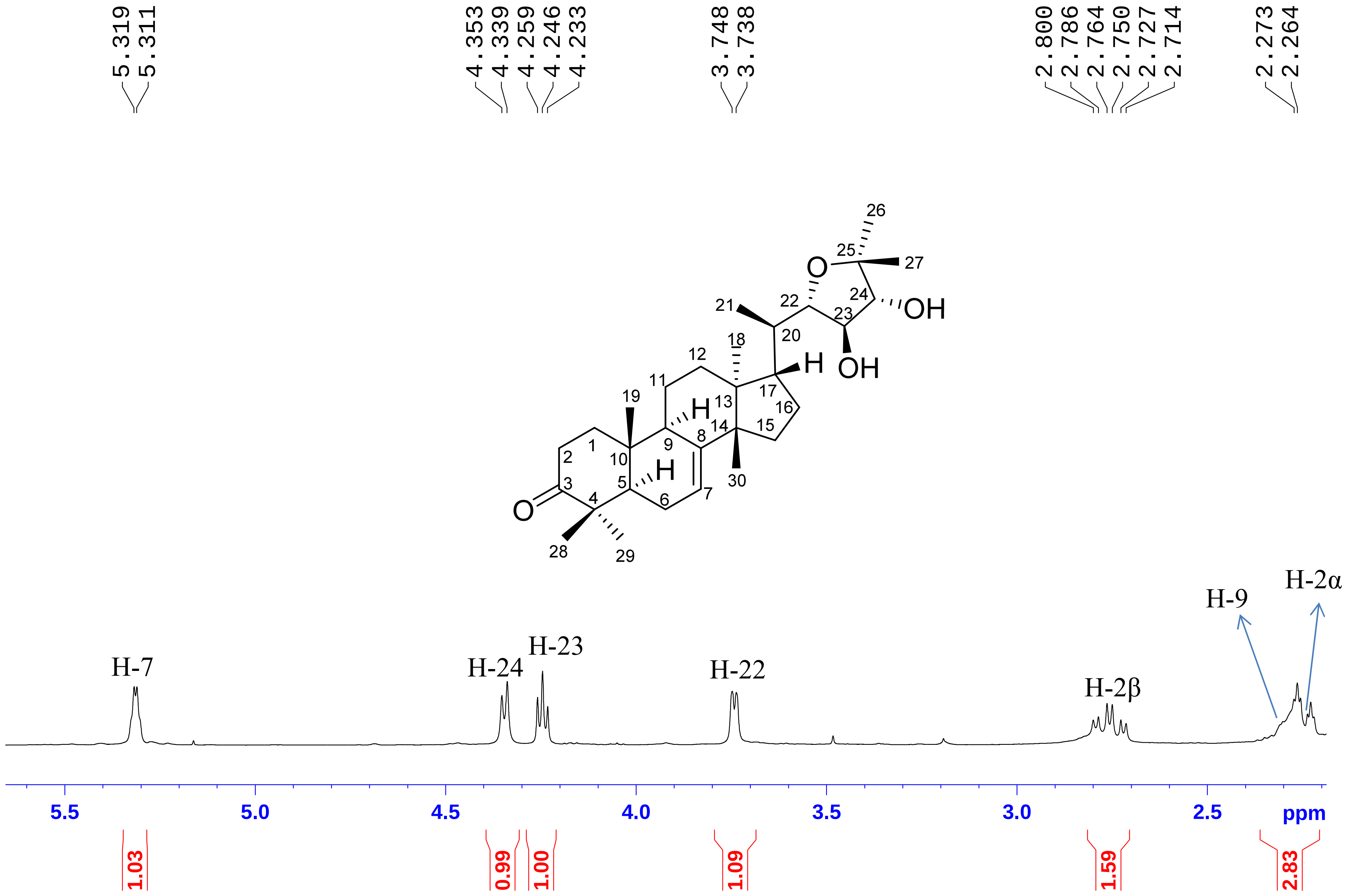

^{1}H NMR (400 MHz) spectrum of compound **5** in $CDCl_3$

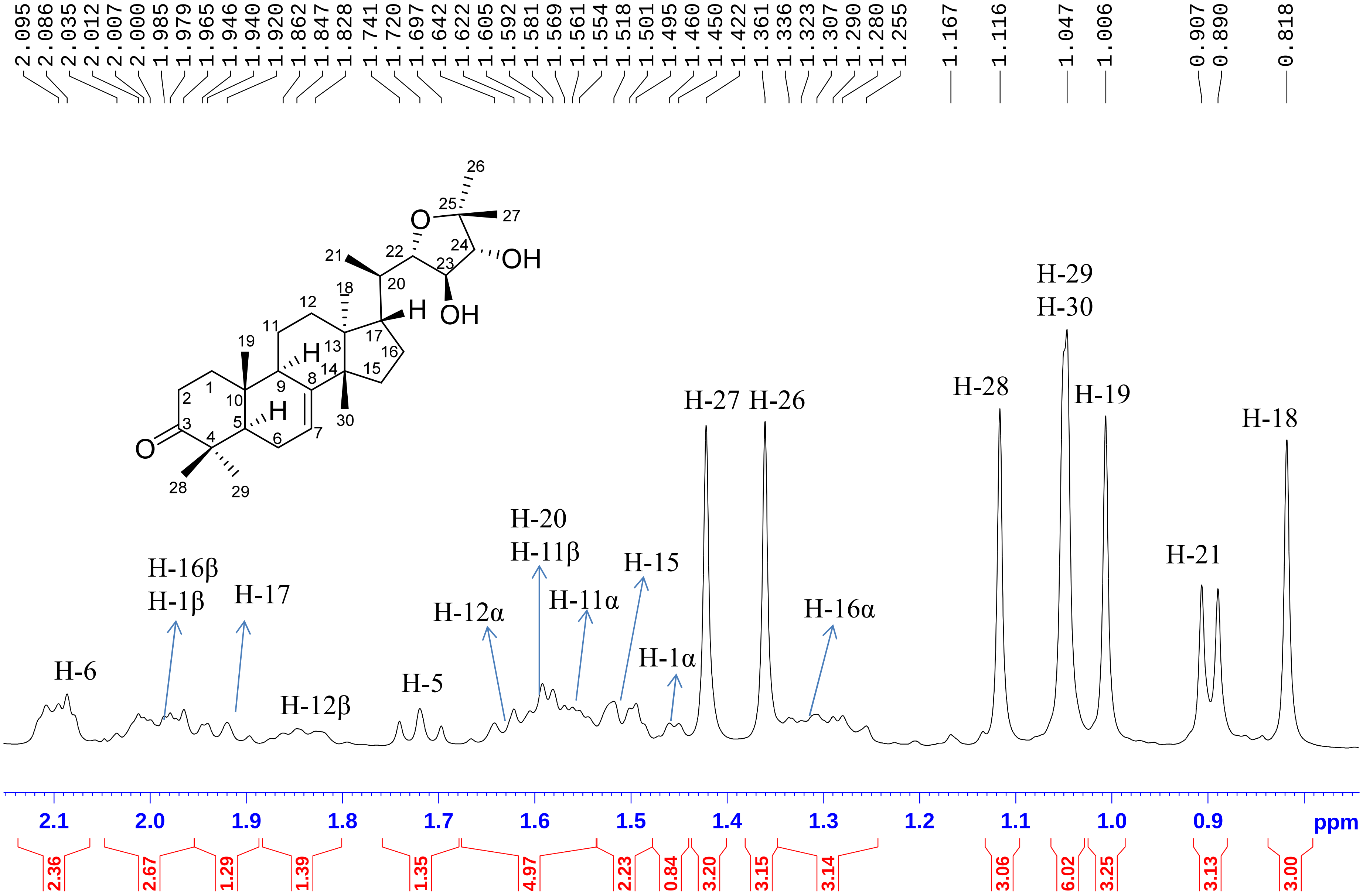

^{13}C NMR (100 MHz) spectrum of compound **5** in $CDCl_3$

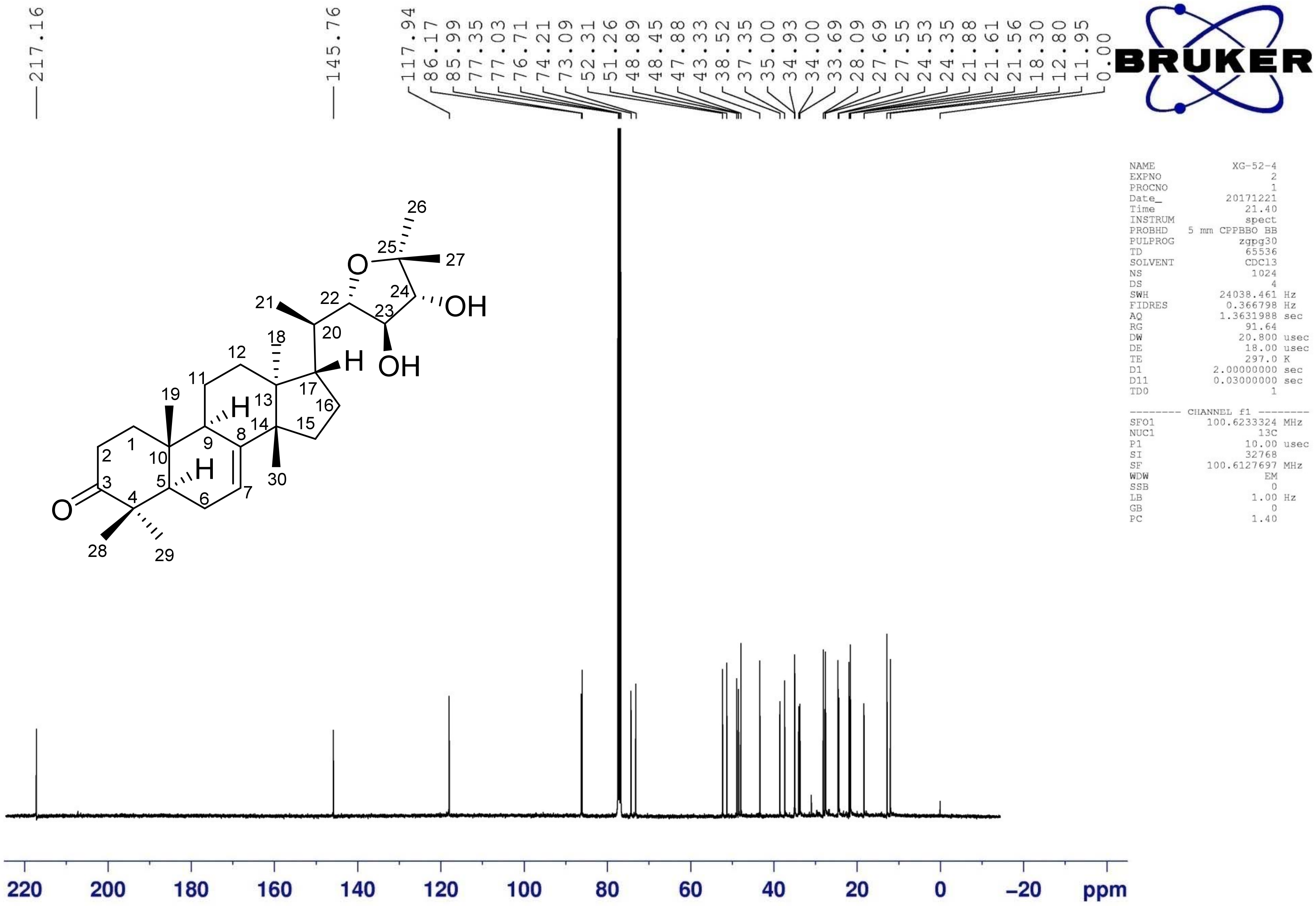

^{13}C NMR (100 MHz) spectrum of compound **5** in $CDCl_3$

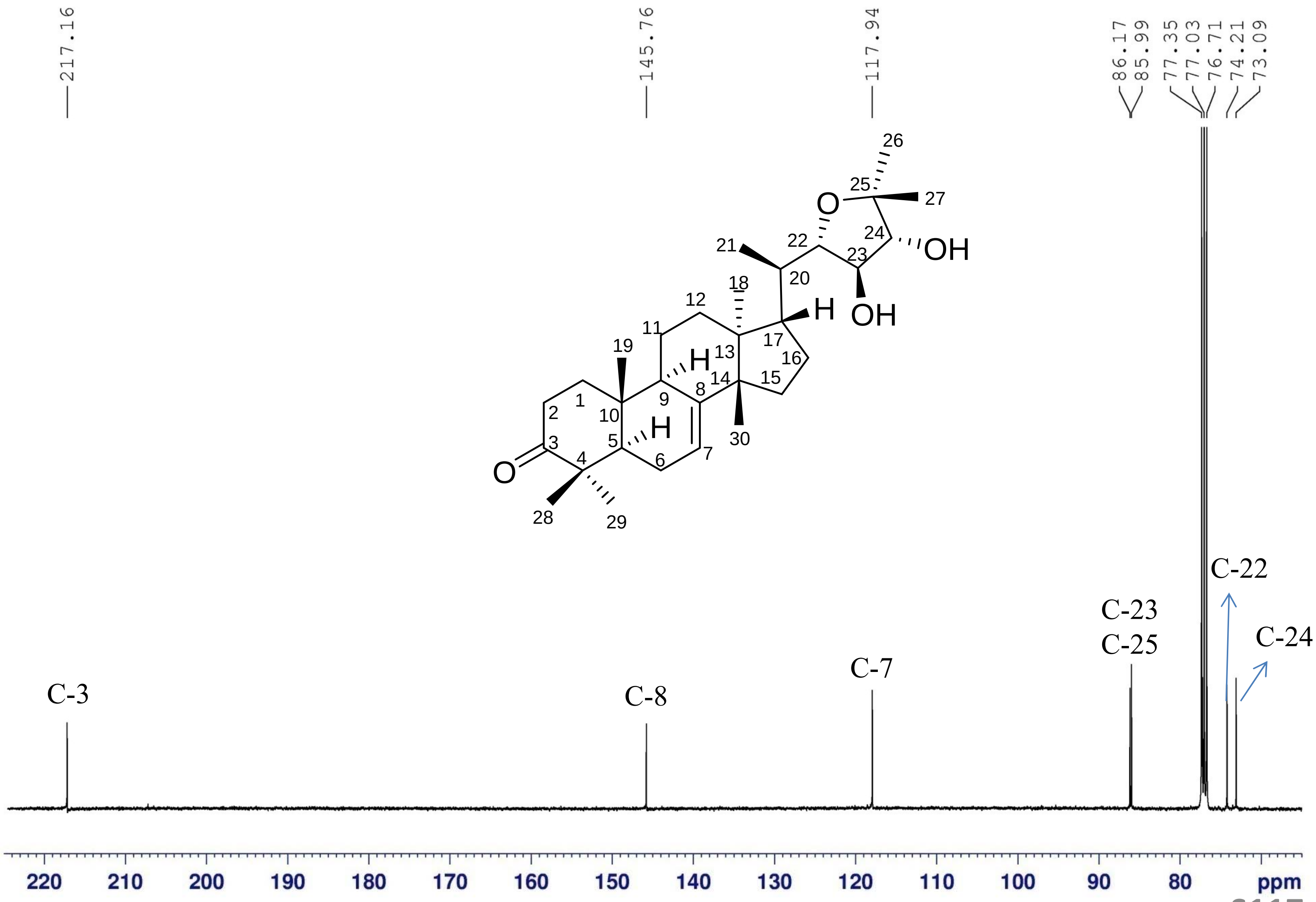

^{13}C NMR (100 MHz) spectrum of compound **5** in $CDCl_3$

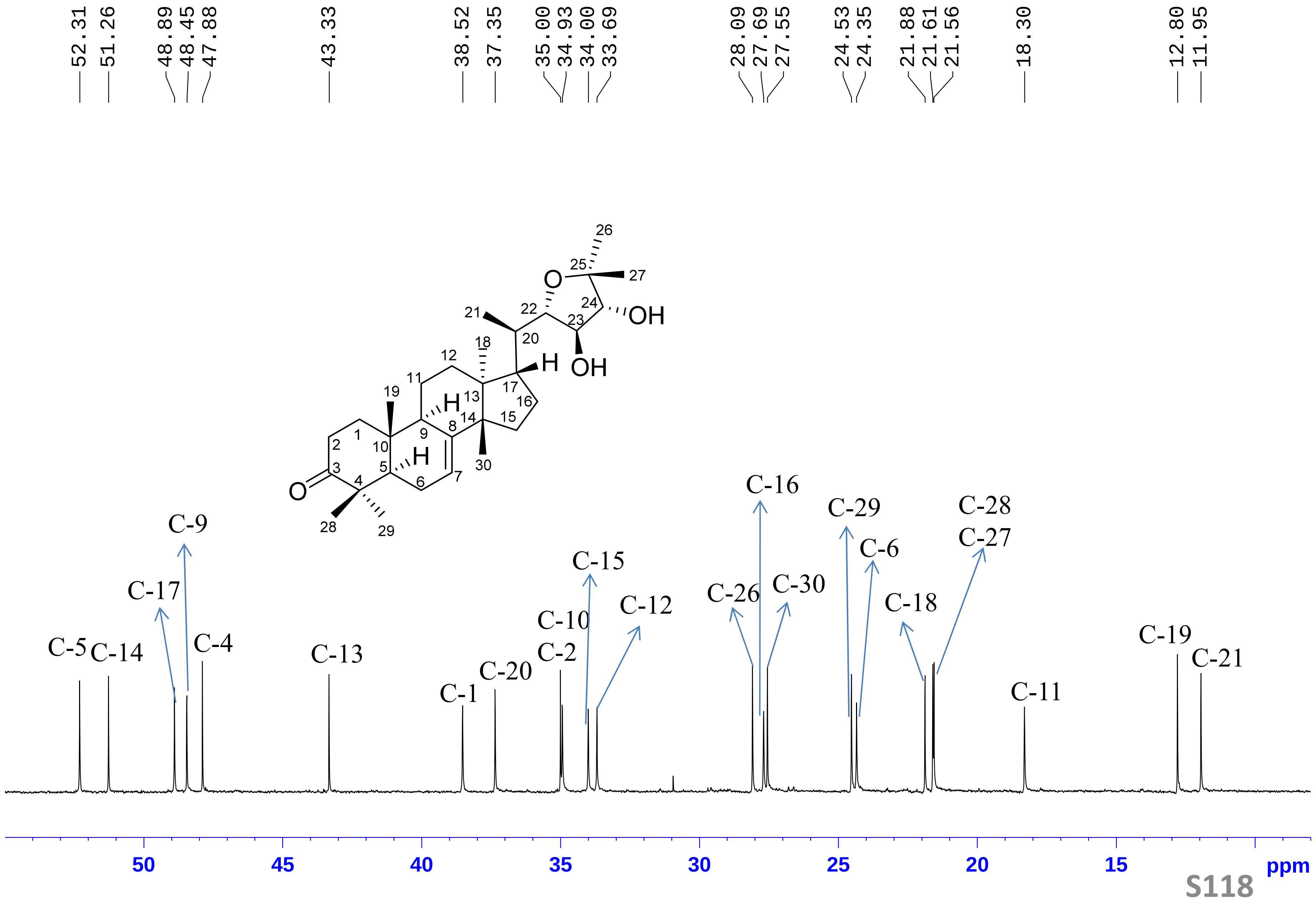

DEPT135 (100 MHz) spectrum of compound **5** in $CDCl_3$

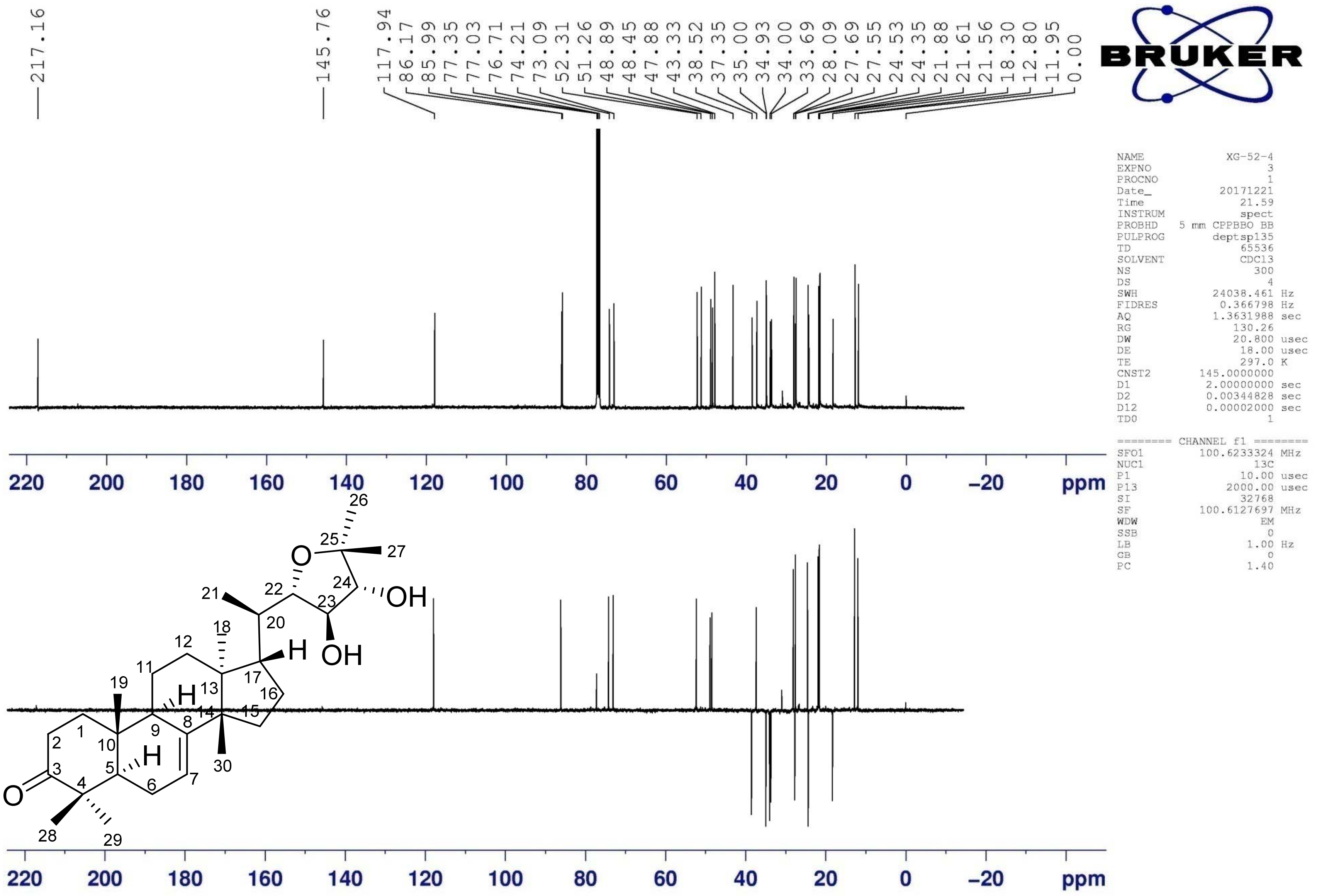

DEPT135 (100 MHz) spectrum of compound **5** in $CDCl_3$

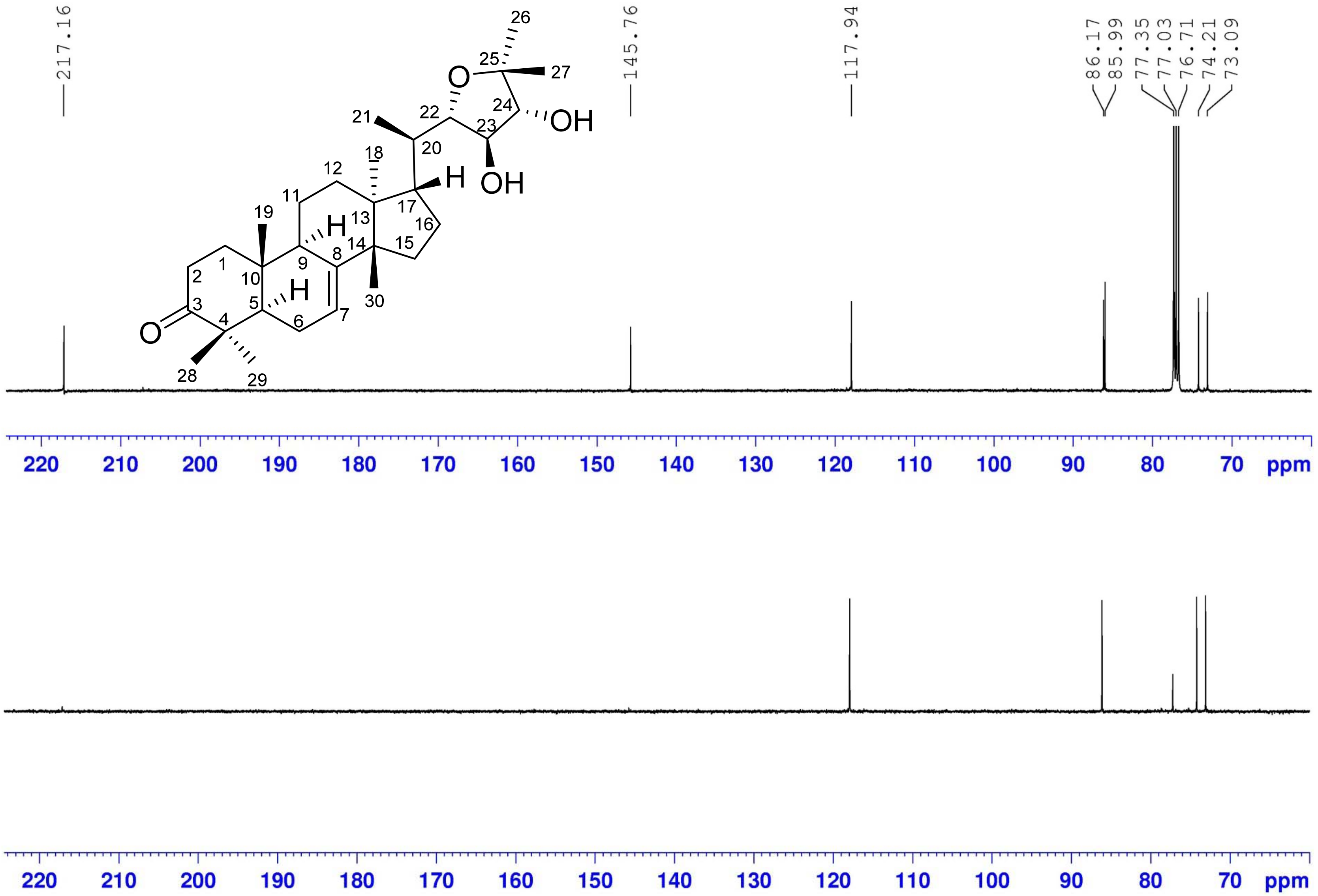

DEPT135 (100 MHz) spectrum of compound **5** in $CDCl_3$

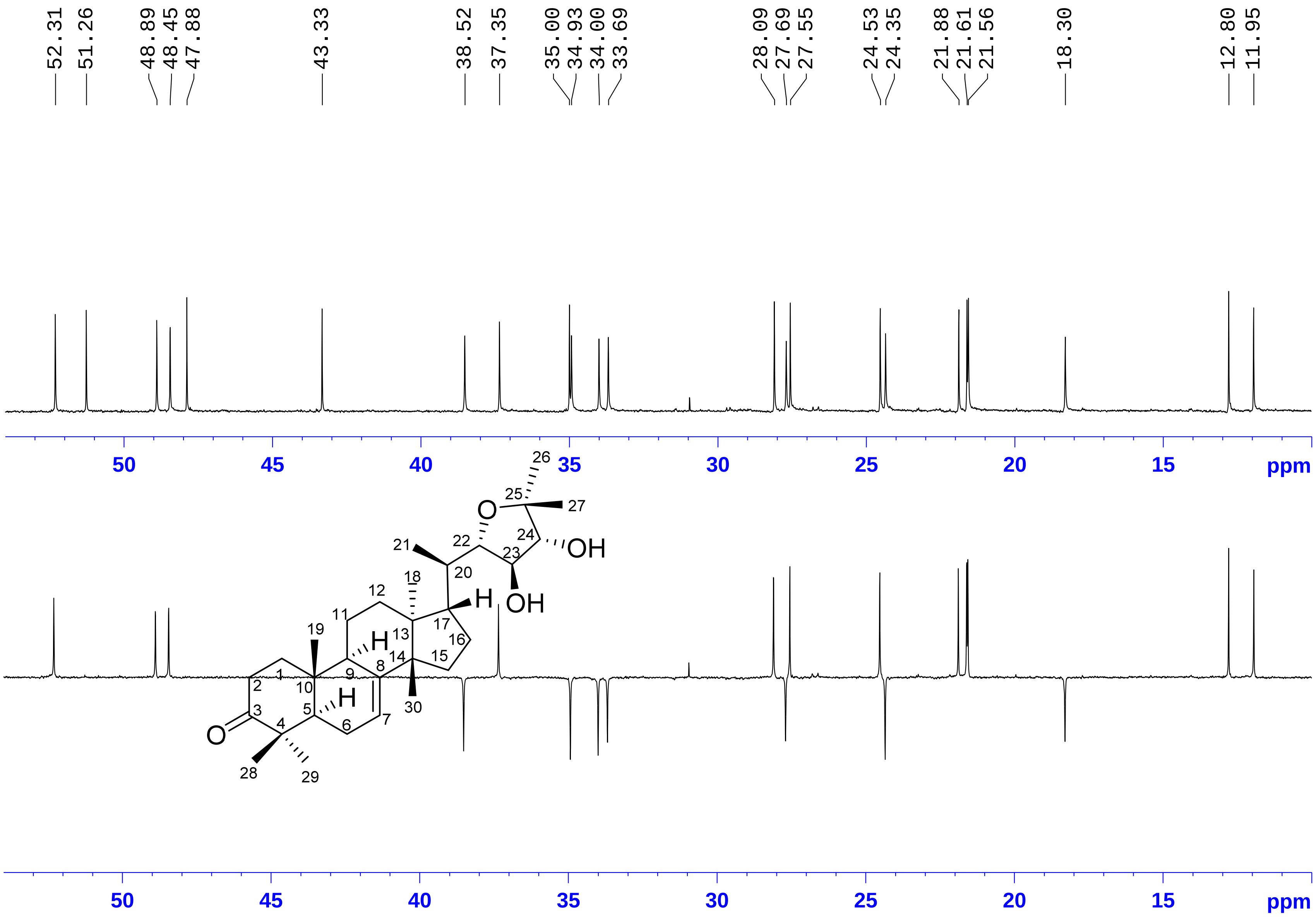

^{1}H-^{1}H COSY (400 MHz) spectrum of compound **5** in $CDCl_3$

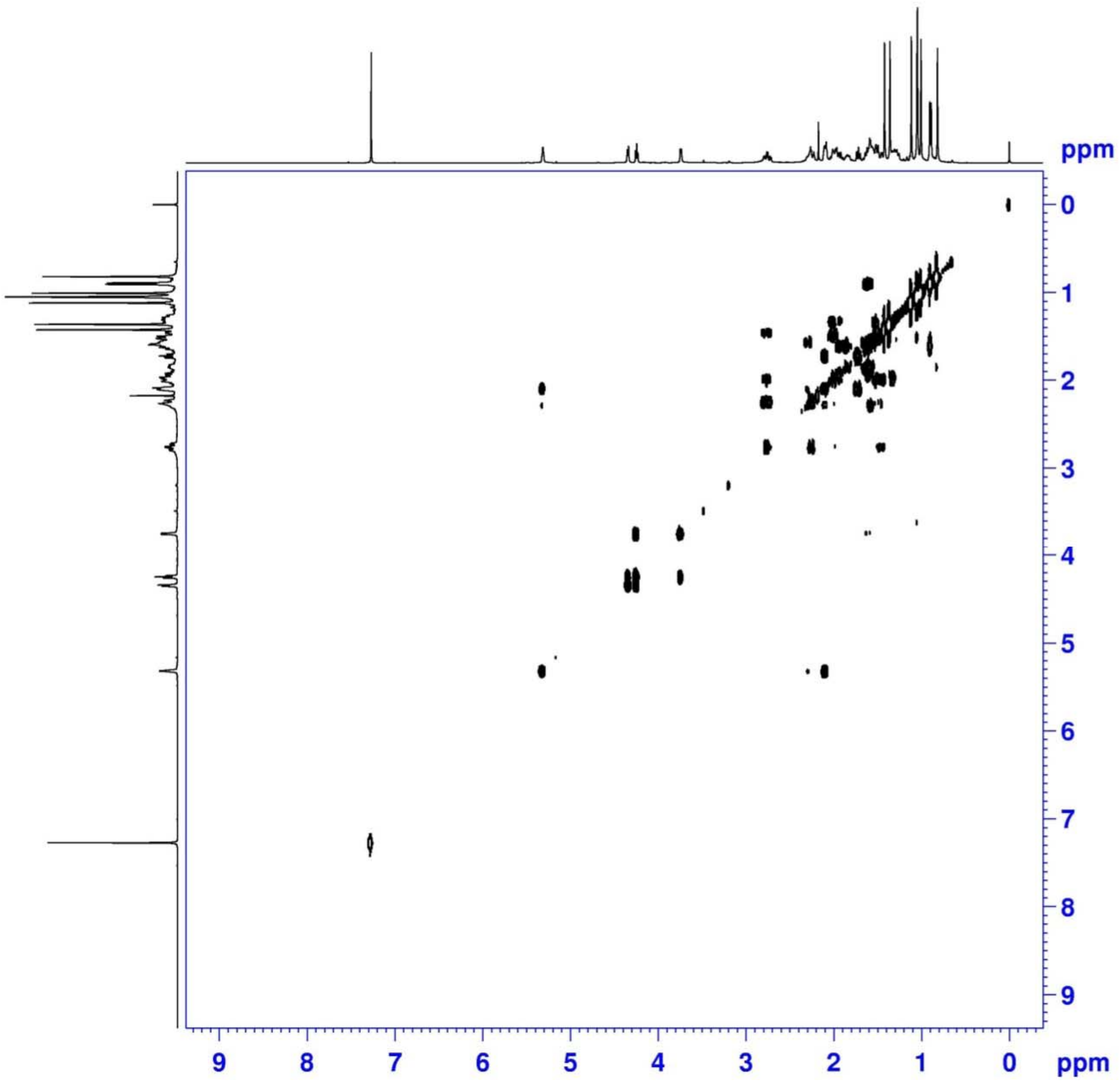

NAME	XG-52-4	
EXPNO	4	
PROCNO	1	
Date_	20171226	
Time	19.41	
INSTRUM	spect	
PROBHD	5 mm CPPBBO BB	
PULPROG	cosygpppqf	
TD	2048	
SOLVENT	CDCl3	
NS	8	
DS	8	
SWH	3906.250	Hz
FIDRES	1.907349	Hz
AQ	0.2621940	sec
RG	65.23	
DW	128.000	usec
DE	10.00	usec
TE	297.0	K
D0	0.00000300	sec
D1	1.89678097	sec
D11	0.03000000	sec
D12	0.00002000	sec
D13	0.00000400	sec
D16	0.00020000	sec
IN0	0.00025600	sec
======== CHANNEL f1 ========		
SFO1	400.1318006	MHz
NUC1	1H	
P0	11.50	usec
P1	11.50	usec
P17	2500.00	usec
ND0	1	
TD	128	
SFO1	400.1318	MHz
FIDRES	30.517578	Hz
SW	9.762	ppm
FnMODE	QF	
SI	1024	
SF	400.1300009	MHz
WDW	QSINE	
SSB	0	
LB	0.00	Hz
GB	0	
PC	1.40	
SI	1024	
MC2	QF	
SF	400.1300009	MHz
WDW	QSINE	
SSB	0	
LB	0.00	Hz
GB	0	

^{1}H-^{1}H COSY (400 MHz) spectrum of compound **5** in $CDCl_3$

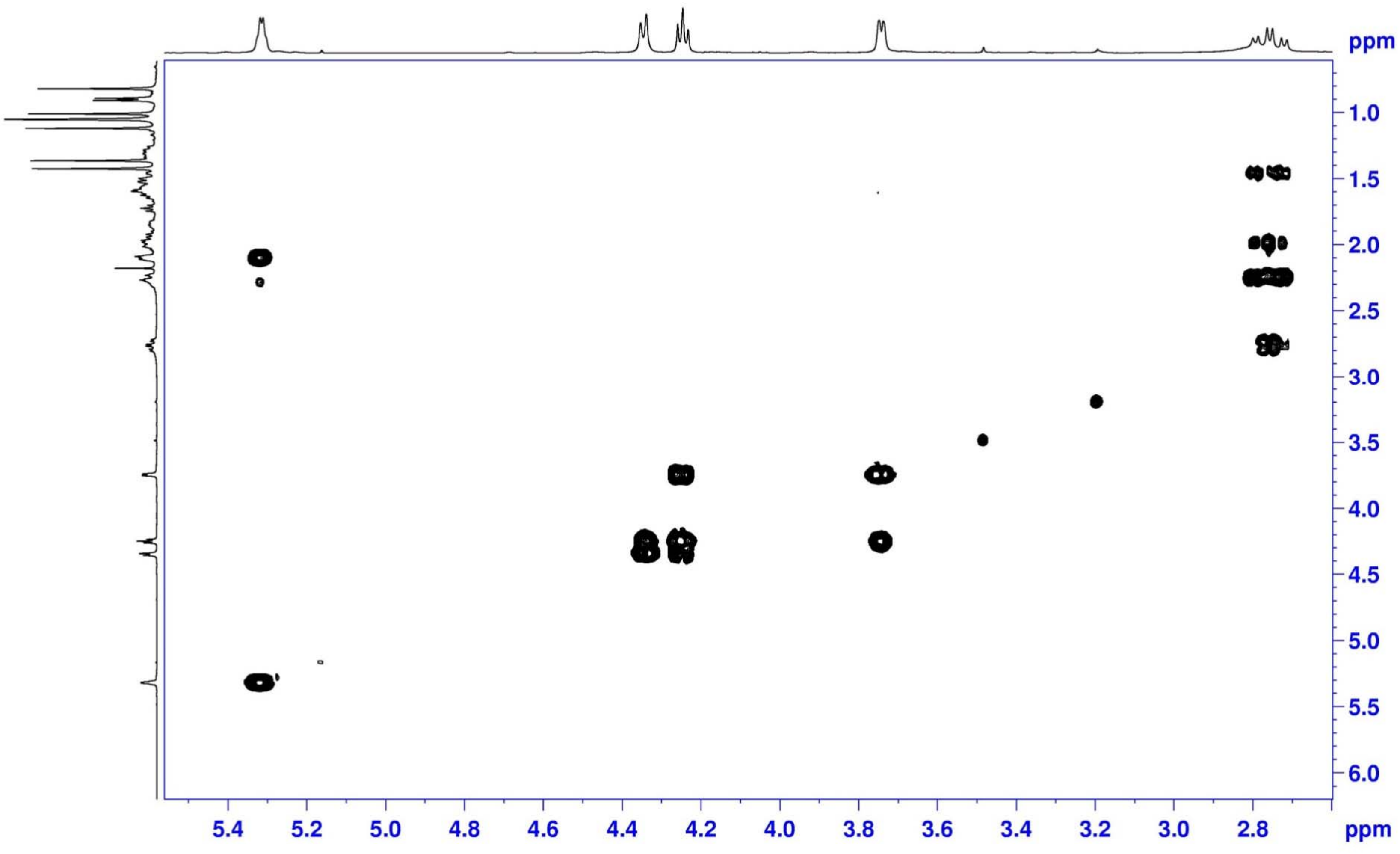

^{1}H-^{1}H COSY (400 MHz) spectrum of compound **5** in $CDCl_3$

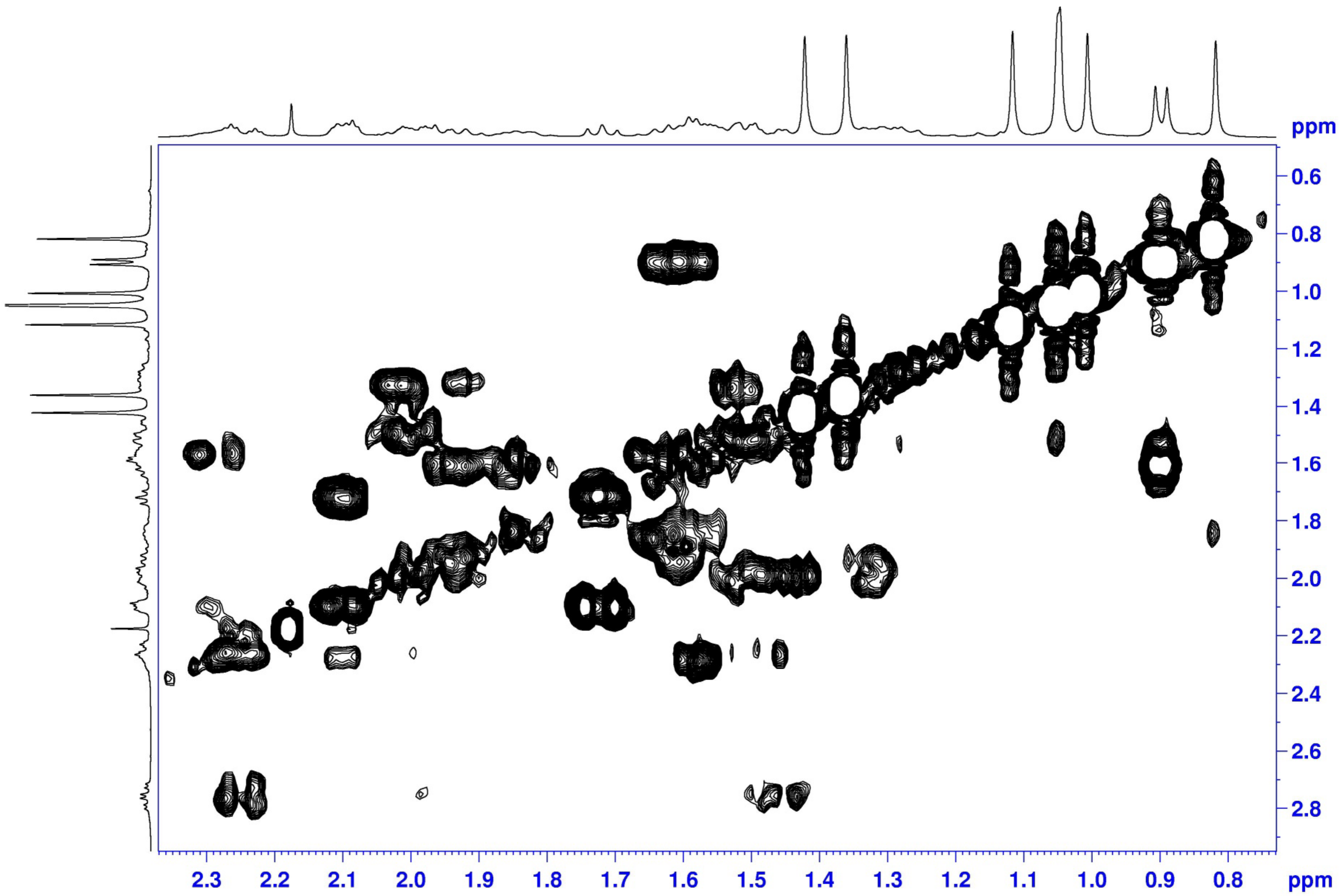

HSQC (400 MHz) spectrum of compound **5** in $CDCl_3$

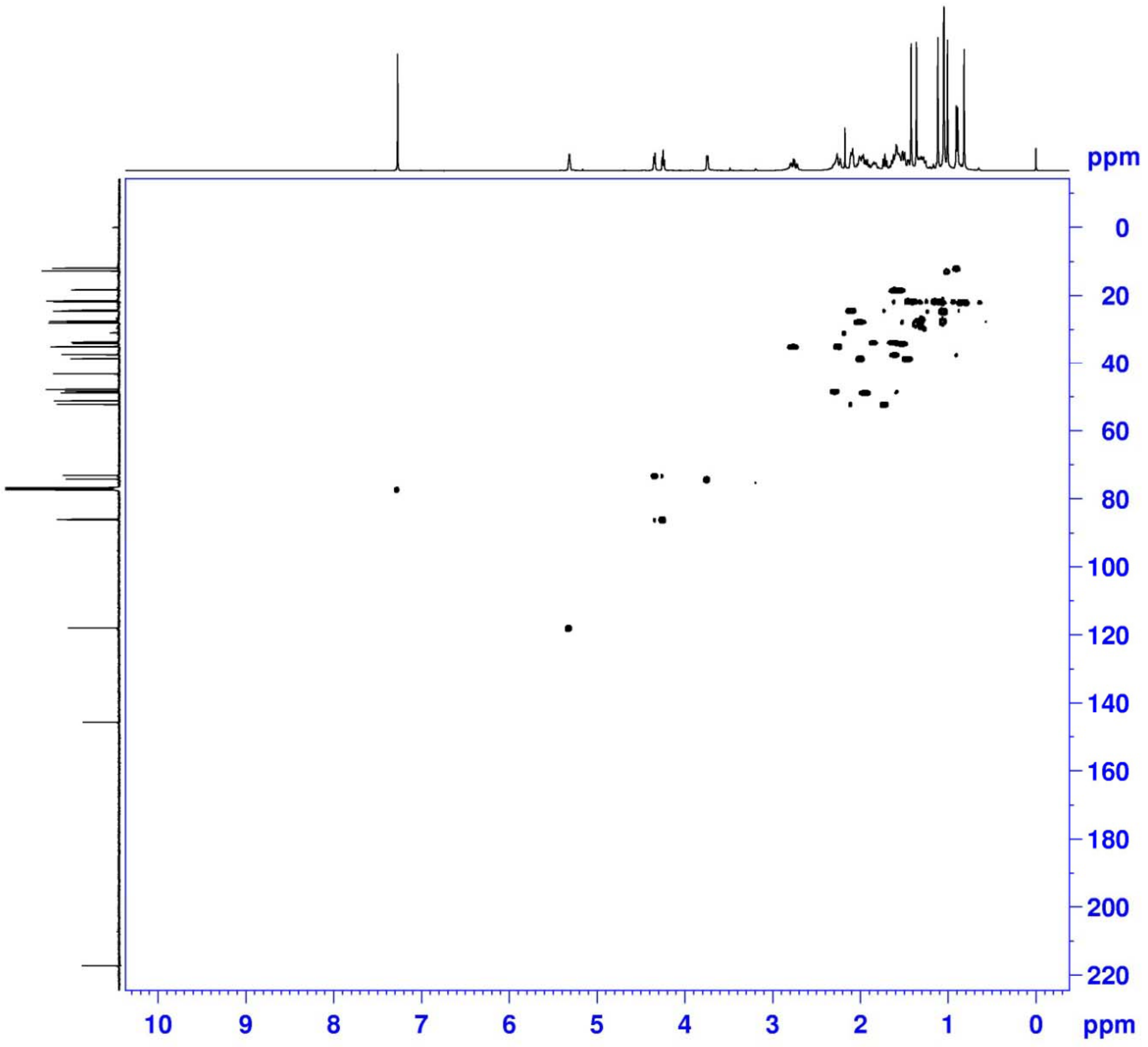


```
NAME            XG-52-4
EXPNO                 5
PROCNO                1
Date_          20171226
Time              20.21
INSTRUM           spect
PROBHD   5 mm CPPBBO BB
PULPROG      hsqcetgpsi2
TD                 1024
SOLVENT           CDCl3
NS                   16
DS                   16
SWH            4302.926 Hz
FIDRES         4.202076 Hz
AQ            0.1190388 sec
RG                208.5
DW              116.200 usec
DE                10.00 usec
TE                297.0 K
CNST2       145.0000000
D0           0.00000300 sec
D1           1.46497905 sec
D4           0.00172414 sec
D11          0.03000000 sec
D16          0.00020000 sec
D24          0.00086207 sec
IN0          0.00002080 sec
ZGOPTNS

======== CHANNEL f1 ========
SFO1        400.1320007 MHz
NUC1                 1H
P1                11.50 usec
P2                23.00 usec
P28                0.00 usec
ND0                   2
TD                  256
SFO1           100.6233 MHz
FIDRES        93.900238 Hz
SW              238.896 ppm
FnMODE     Echo-Antiecho
SI                 1024
SF          400.1300014 MHz
WDW               QSINE
SSB                   2
LB                 0.00 Hz
GB                    0
PC                 1.40
SI                 1024
MC2        echo-antiecho
SF          100.6127577 MHz
WDW               QSINE
SSB                   2
LB                 0.00 Hz
GB                    0
```

HSQC (400 MHz) spectrum of compound **5** in $CDCl_3$

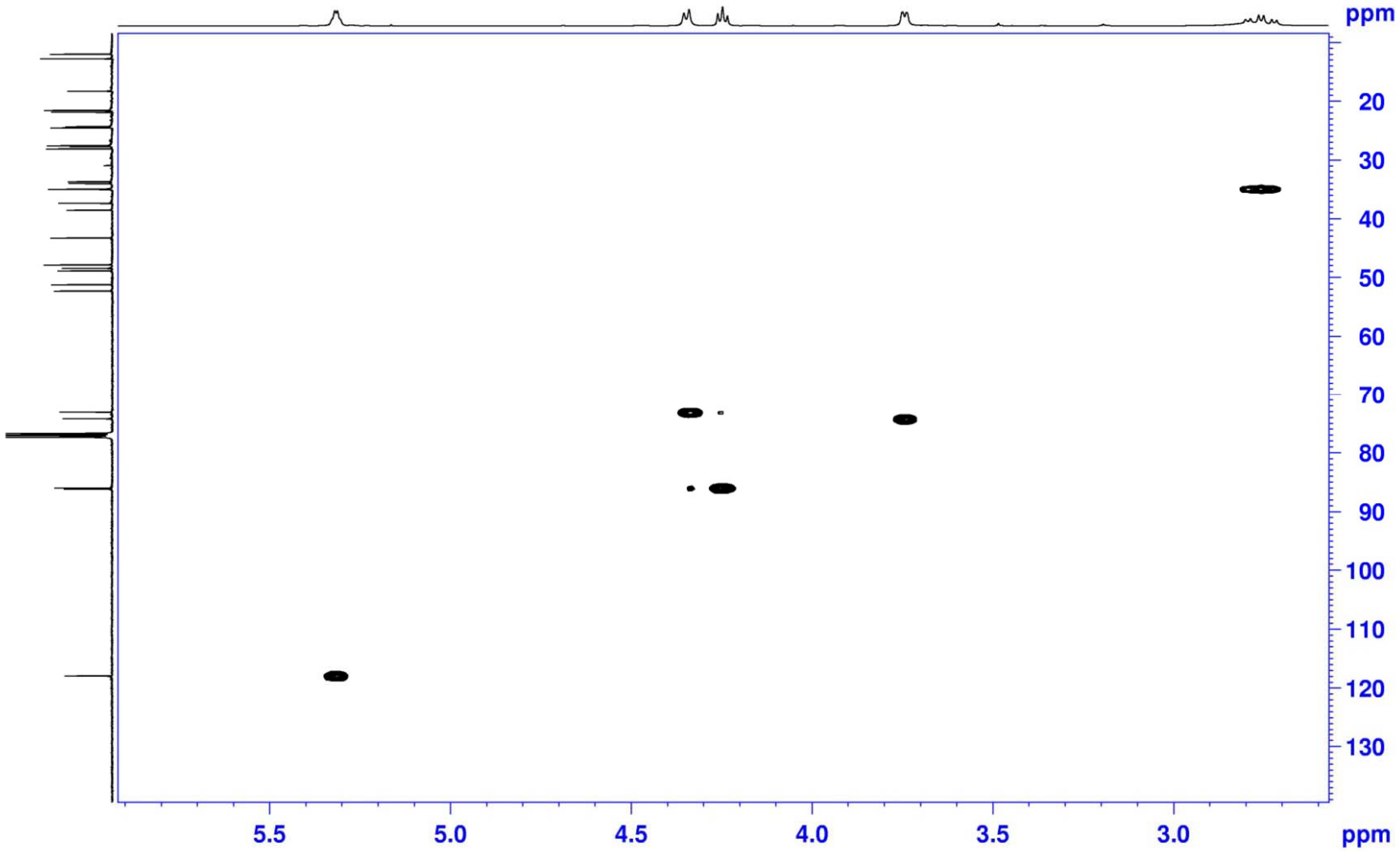

HSQC (400 MHz) spectrum of compound **5** in $CDCl_3$

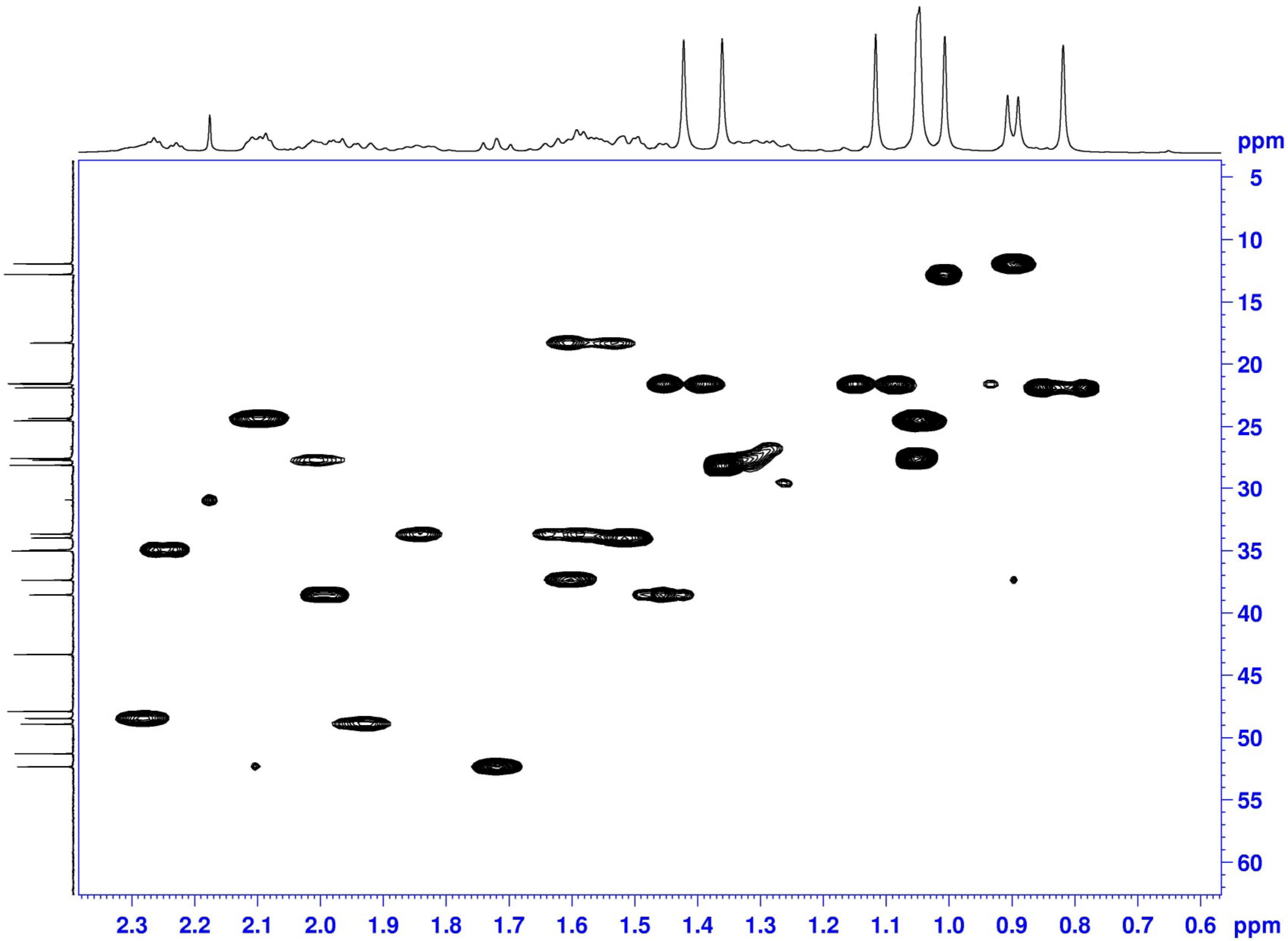

HMBC (400 MHz) spectrum of compound **5** in $CDCl_3$

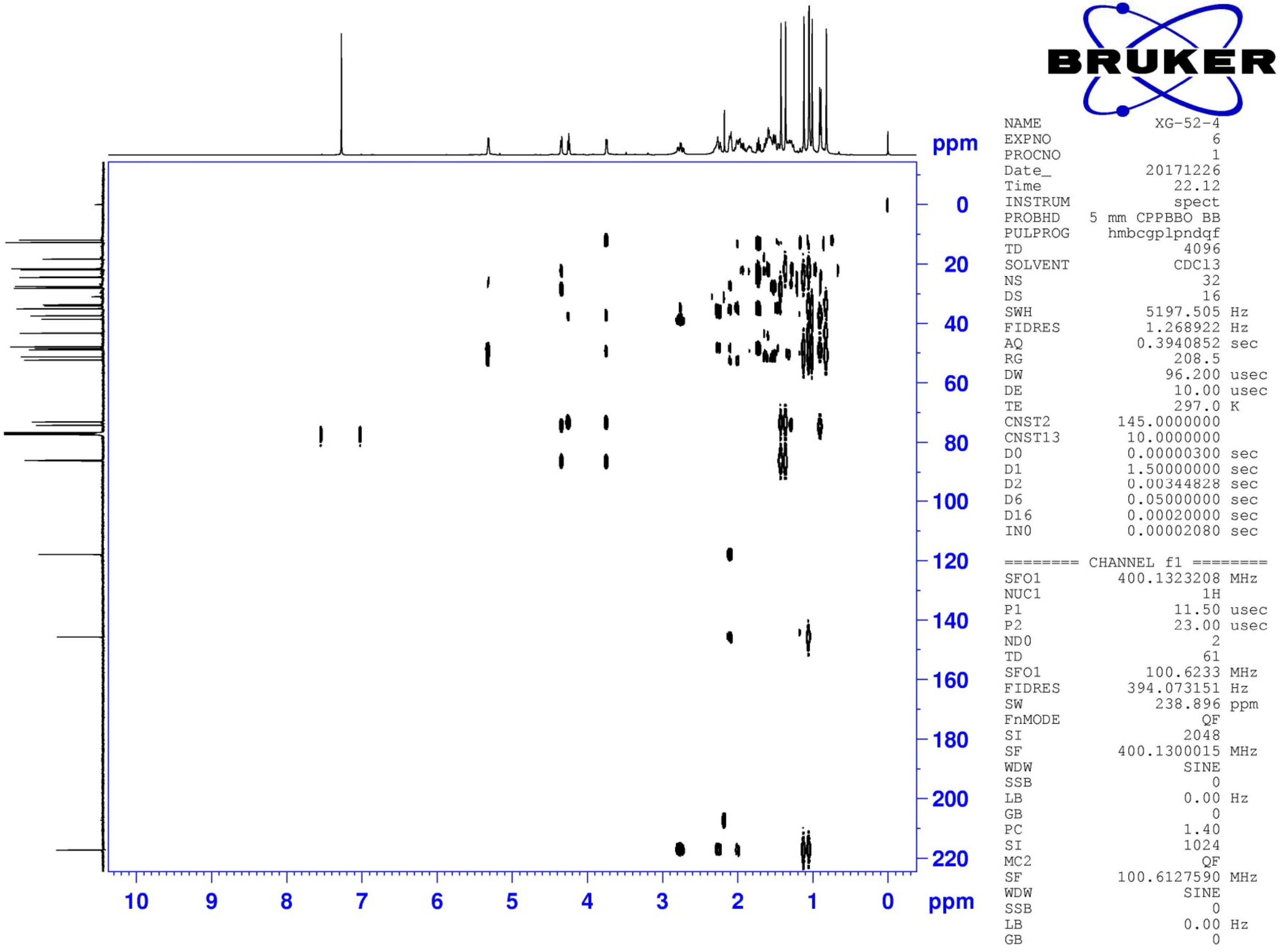

HMBC (400 MHz) spectrum of compound **5** in $CDCl_3$

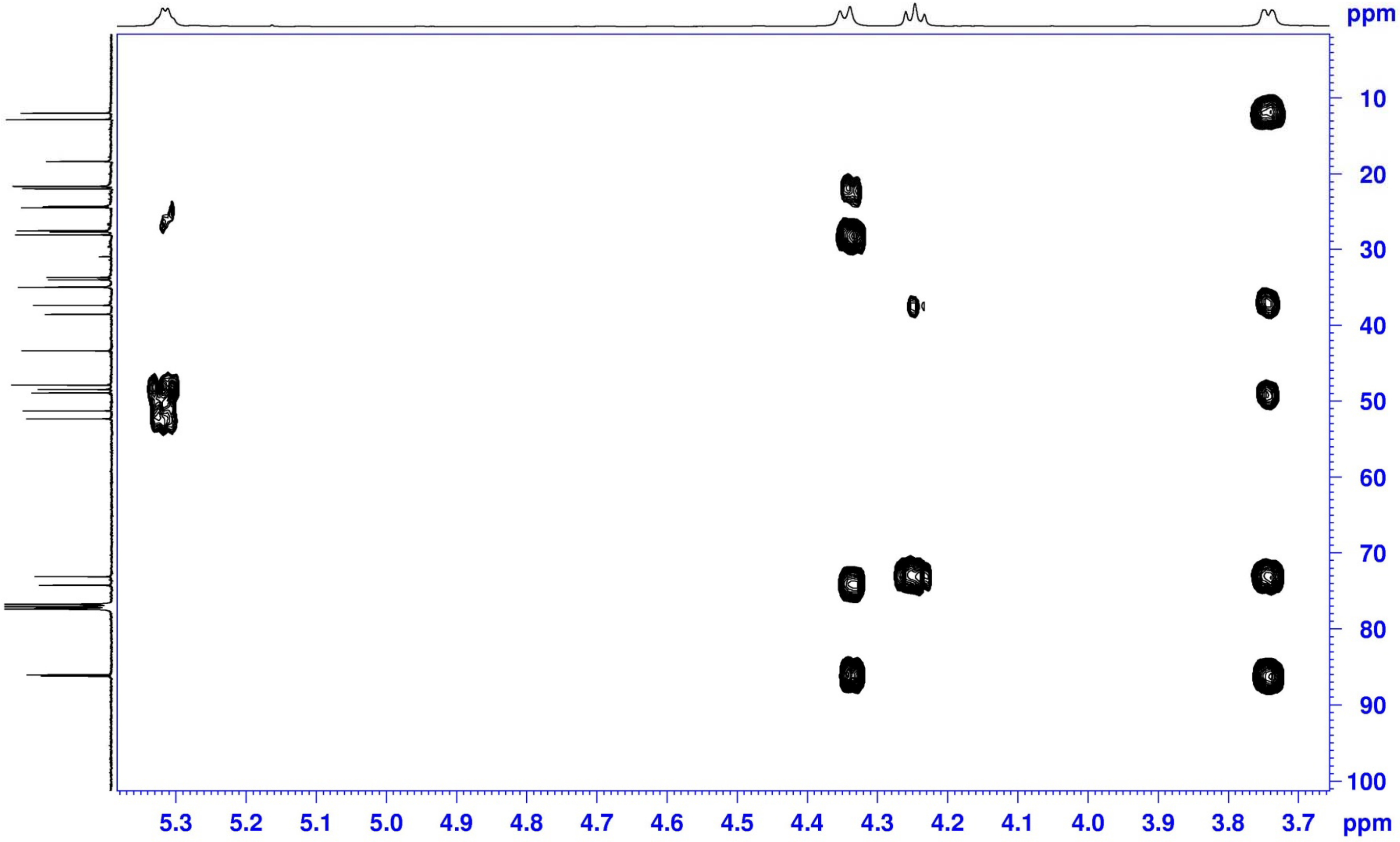

HMBC (400 MHz) spectrum of compound **5** in $CDCl_3$

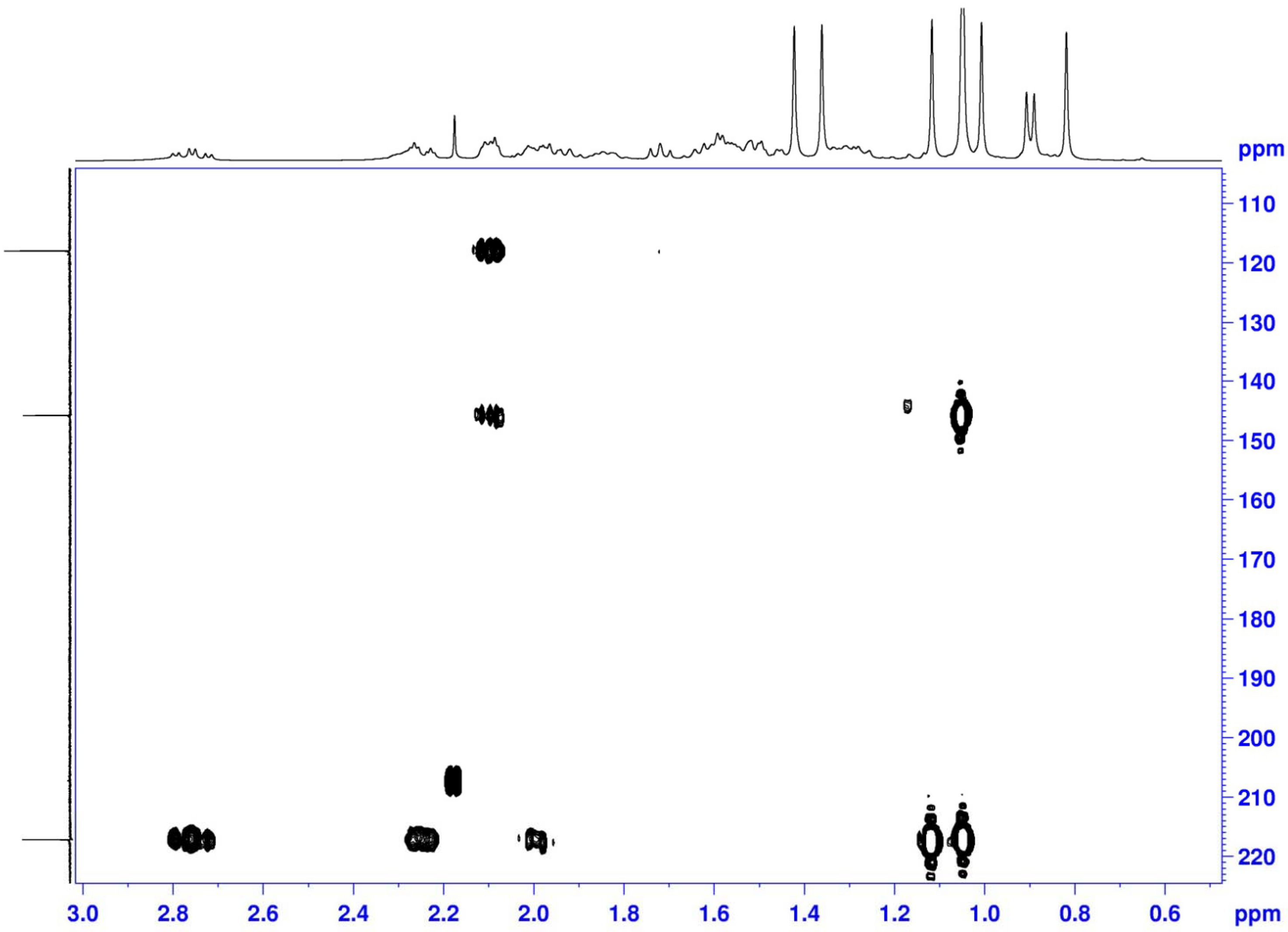

HMBC (400 MHz) spectrum of compound **5** in $CDCl_3$

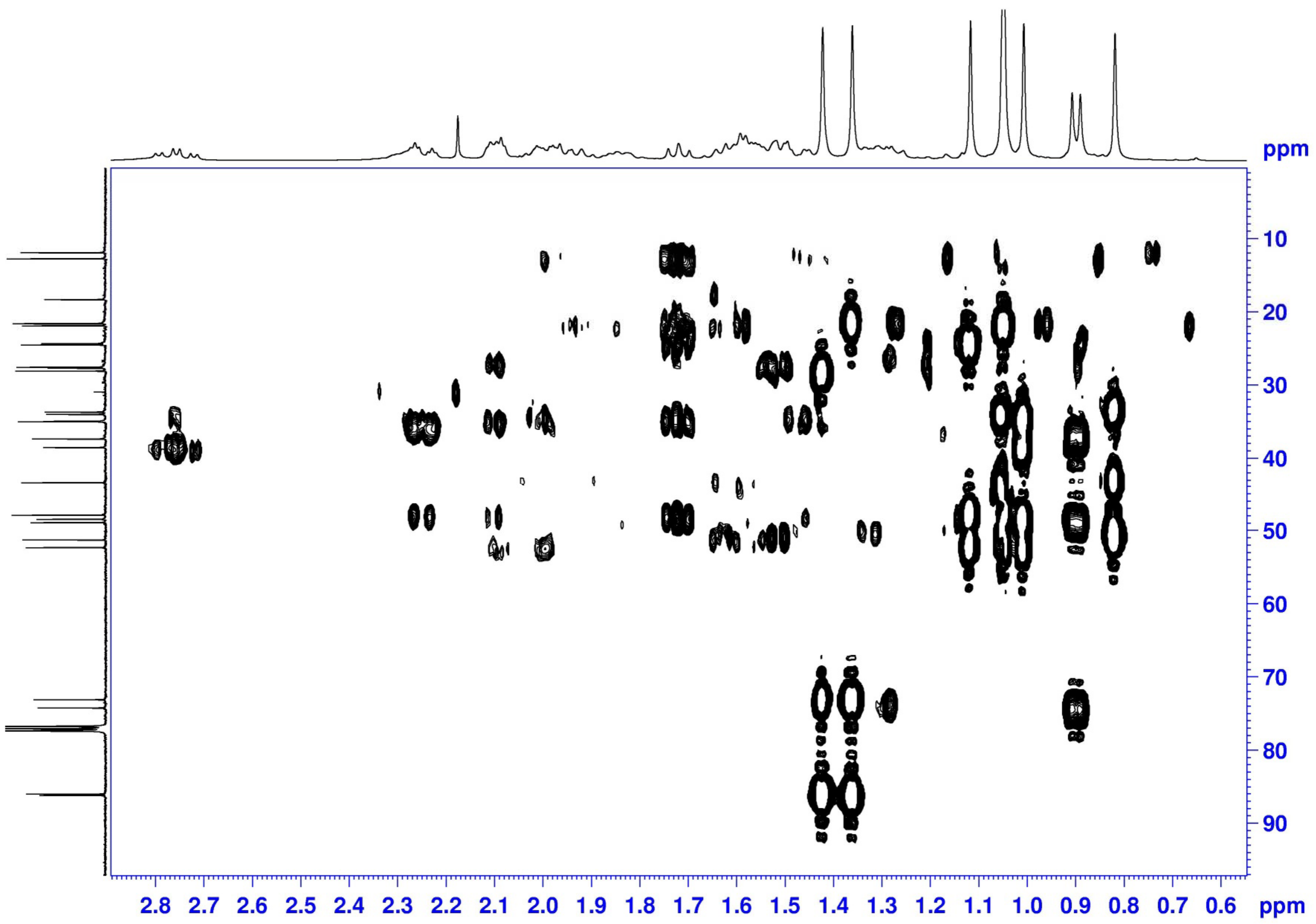

HMBC (400 MHz) spectrum of compound **5** in $CDCl_3$

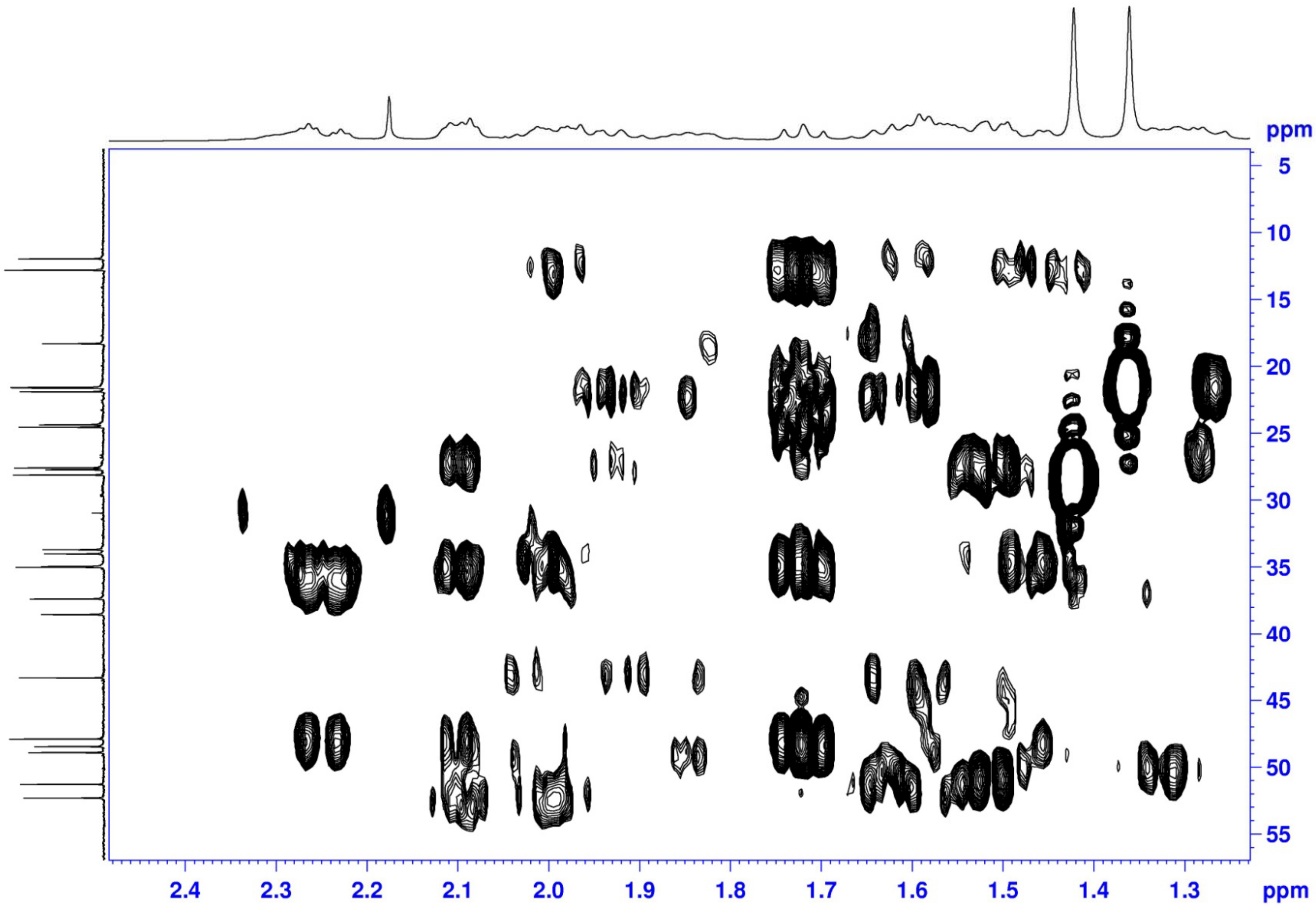

HMBC (400 MHz) spectrum of compound **5** in $CDCl_3$

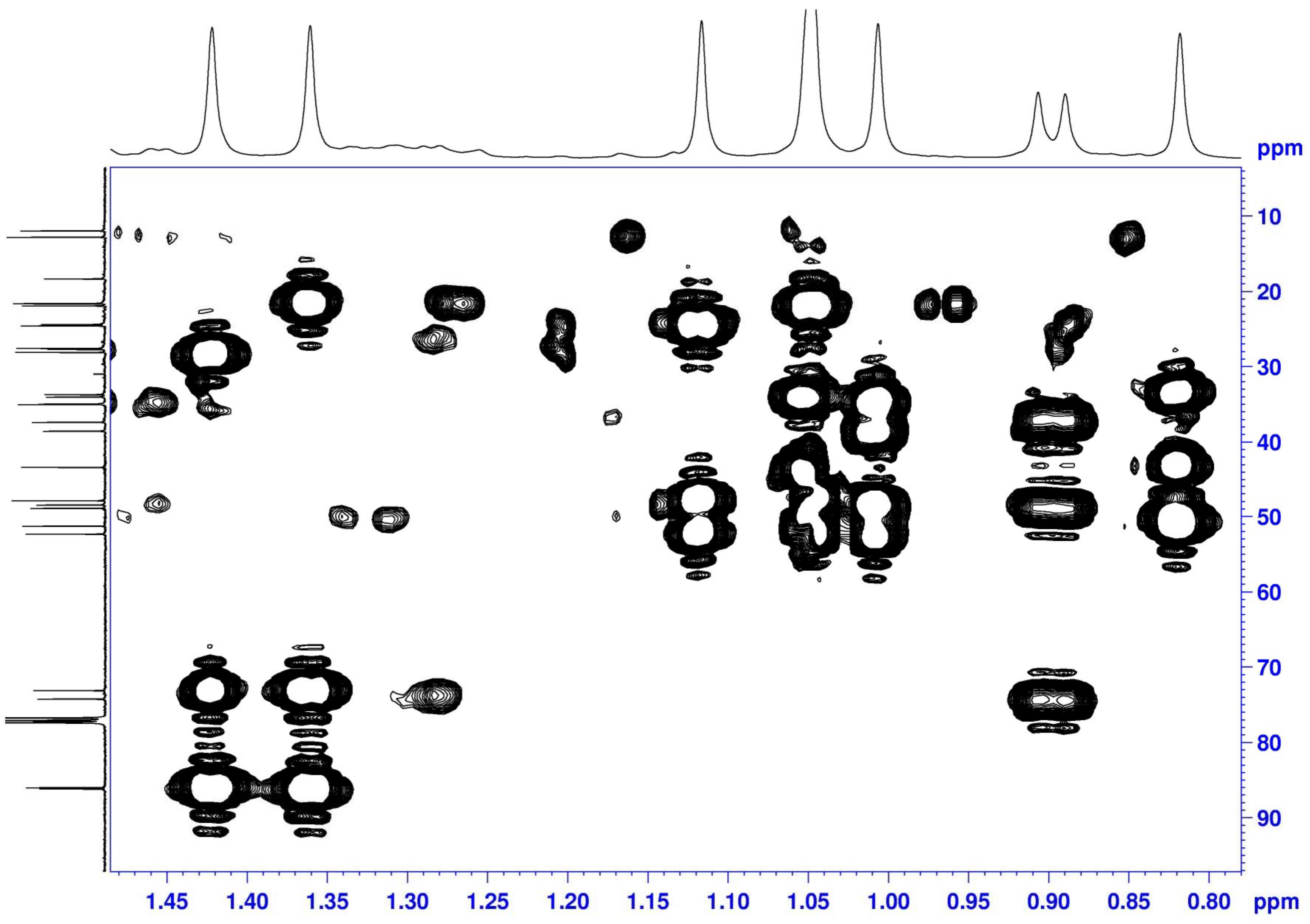

NOESY (400 MHz) spectrum of compound **5** in $CDCl_3$

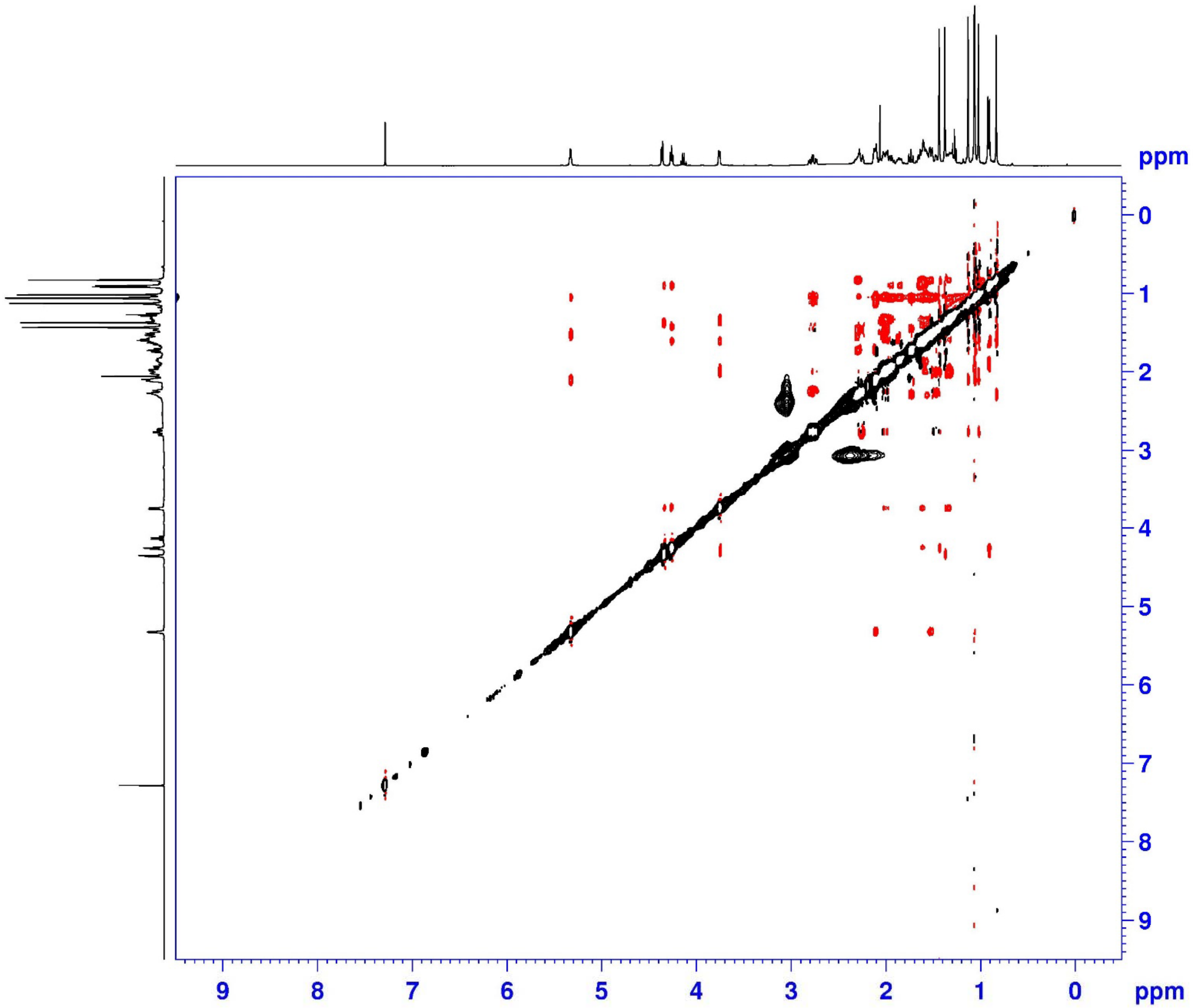

NAME LRX-52-4
EXPNO 7
PROCNO 1
Date_ 20171228
Time 5.30
INSTRUM spect
PROBHD 5 mm CPPBBO BB
PULPROG noesygpphpp
TD 2048
SOLVENT CDCl3
NS 16
DS 32
SWH 4000.000 Hz
FIDRES 1.953125 Hz
AQ 0.2560500 sec
RG 65.23
DW 125.000 use
DE 10.00 use
TE 297.0 K
D0 0.00011036 sec
D1 1.99385595 sec
D8 0.30000001 sec
D11 0.03000000 sec
D12 0.00002000 sec
D16 0.00020000 sec
IN0 0.00025000 sec

======== CHANNEL f1 =======
SFO1 400.1318006 MHz
NUC1 1H
P1 11.50 use
P2 23.00 use
P17 2500.00 use
ND0 1
TD 256
SFO1 400.1318 MHz
FIDRES 15.625000 Hz
SW 9.997 ppm
FnMODE States-TPPI
SI 1024
SF 400.1300000 MHz
WDW QSINE
SSB 2
LB 0.00 Hz
GB 0
PC 1.00
SI 1024
MC2 States-TPPI
SF 400.1300000 MHz
WDW QSINE
SSB 2

NOESY (400 MHz) spectrum of compound **5** in $CDCl_3$

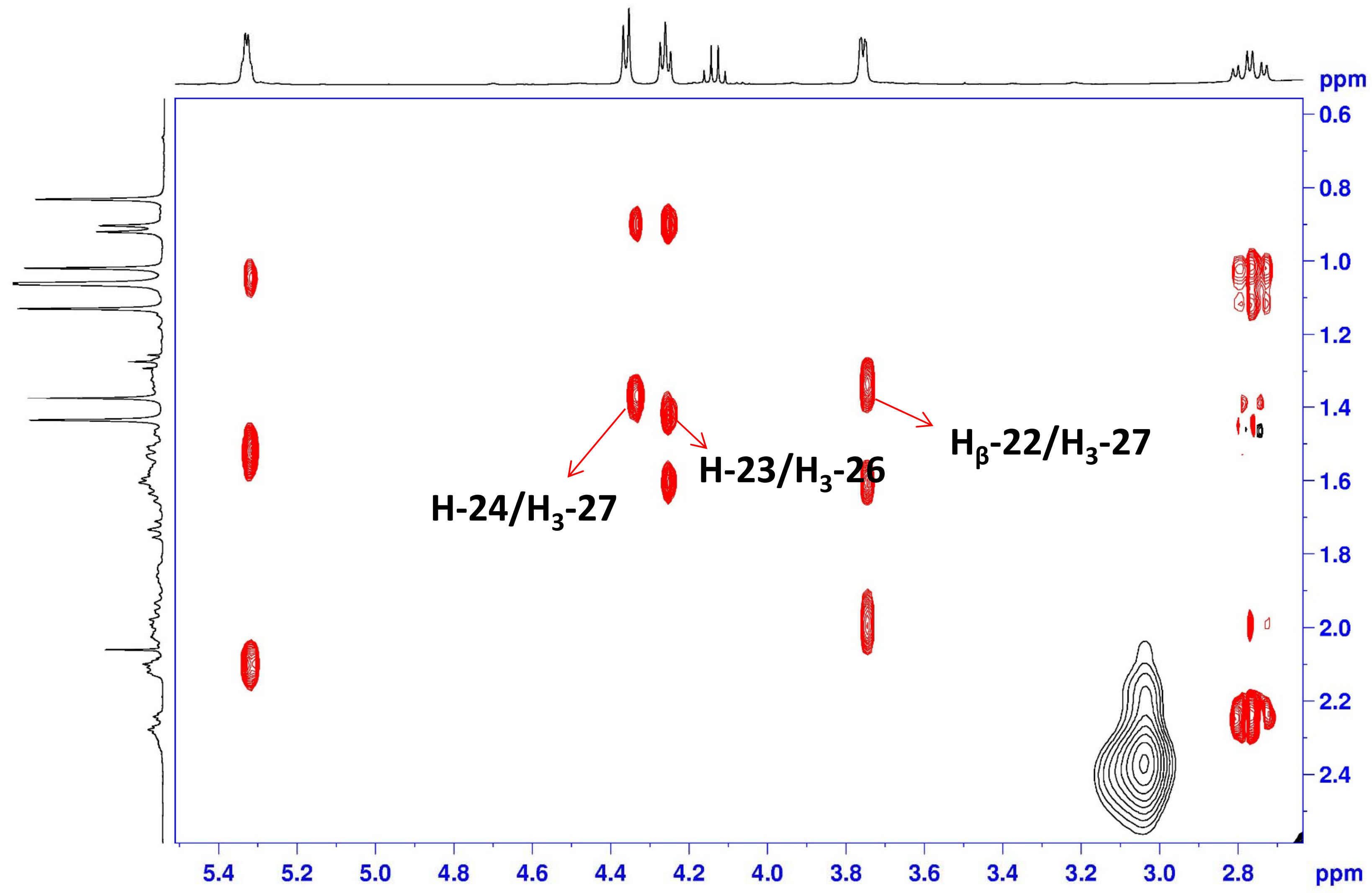

NOESY (400 MHz) spectrum of compound **5** in $CDCl_3$

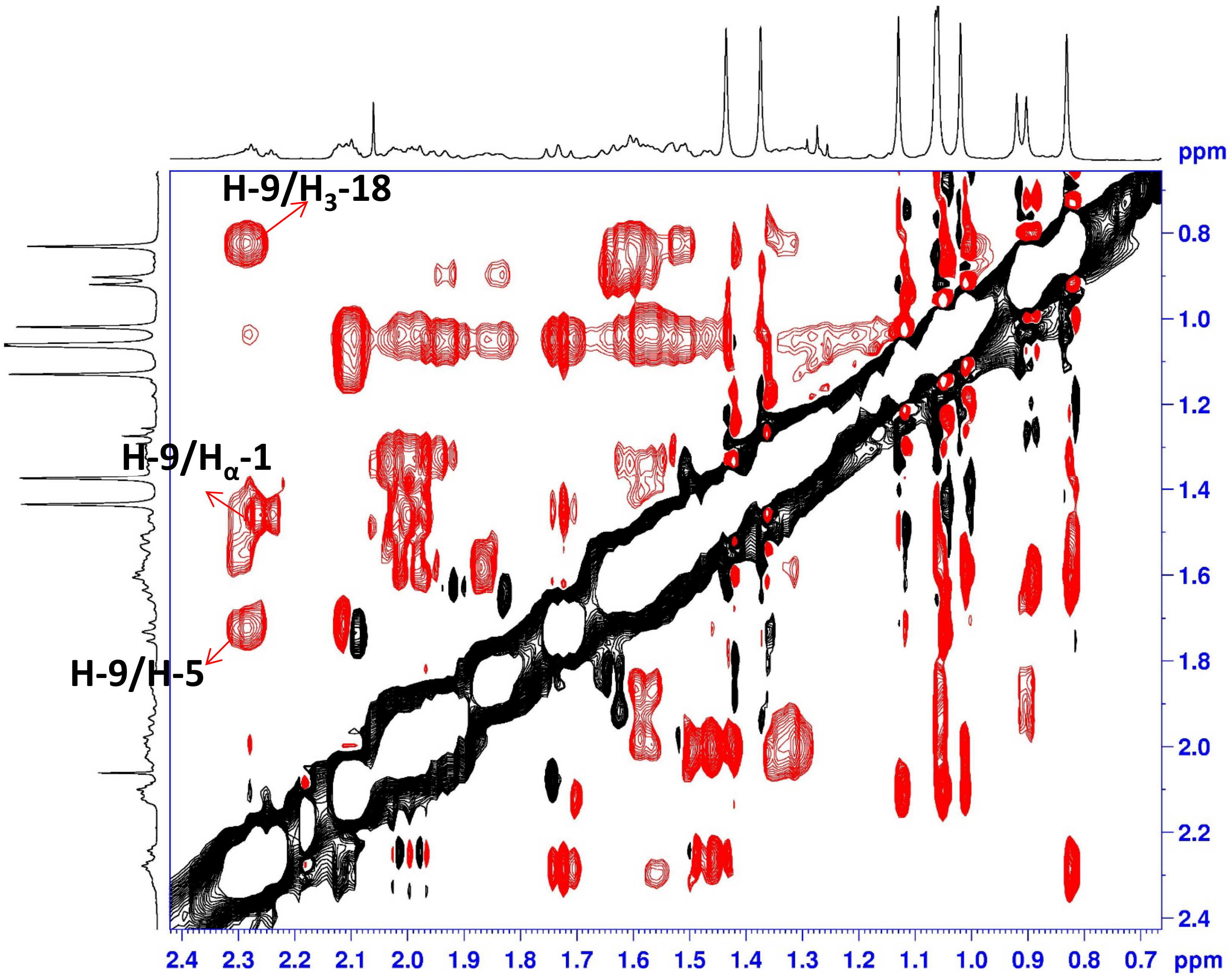

NOESY (400 MHz) spectrum of compound **5** in $CDCl_3$

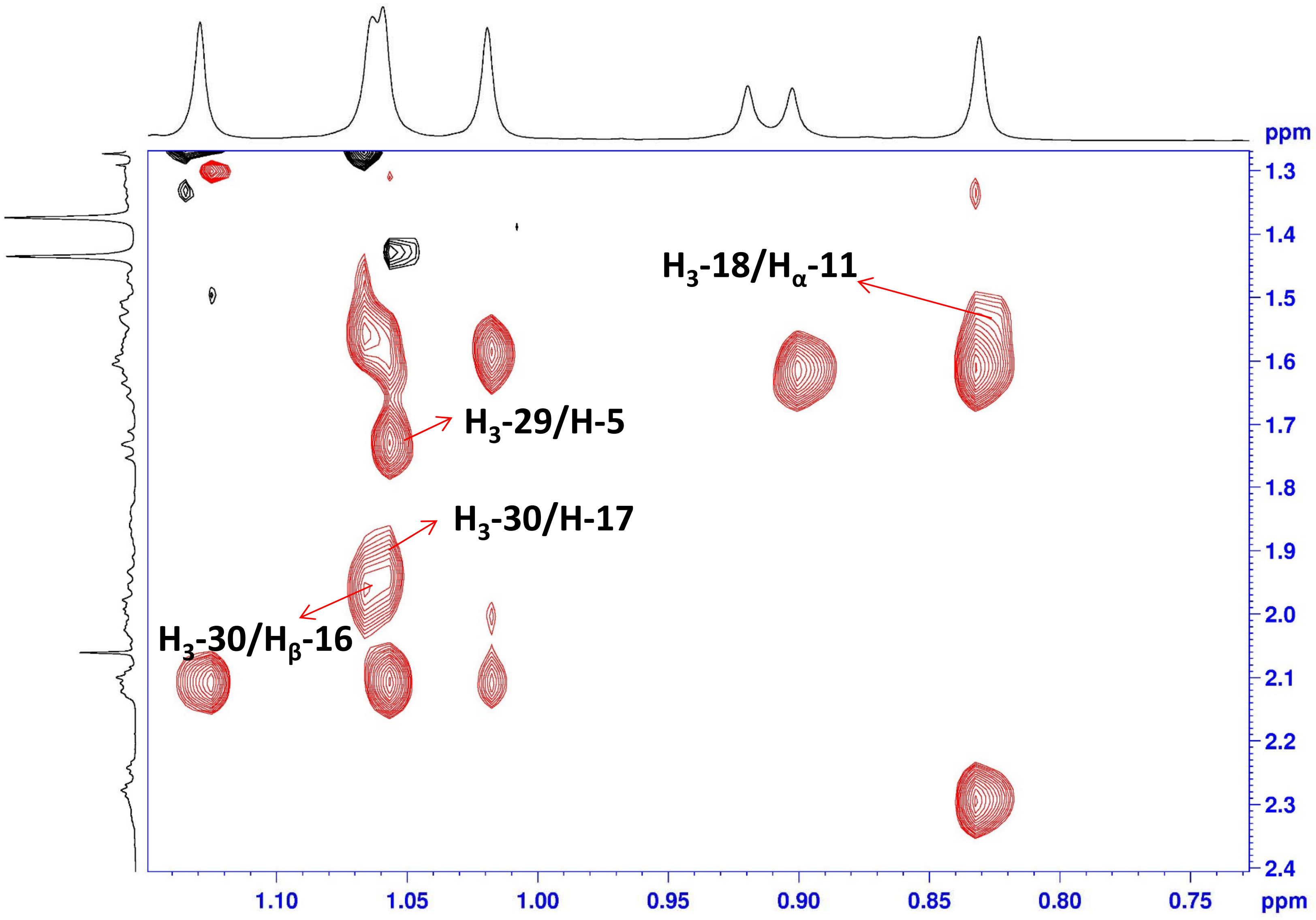

HR-ESIMS for compound **6**

Generic Display Report

Analysis Info		Acquisition Date	12/21/2016 4:51:43 PM
Analysis Name	D:\Data\MS\data\201612\liwanshan_EA-16_pos_25_01_2825.d		
Method	LC_Direct Infusion_pos_70-500mz.m	Operator	SCSIO
Sample Name	liwanshan_EA-16_pos	Instrument	maXis
Comment			

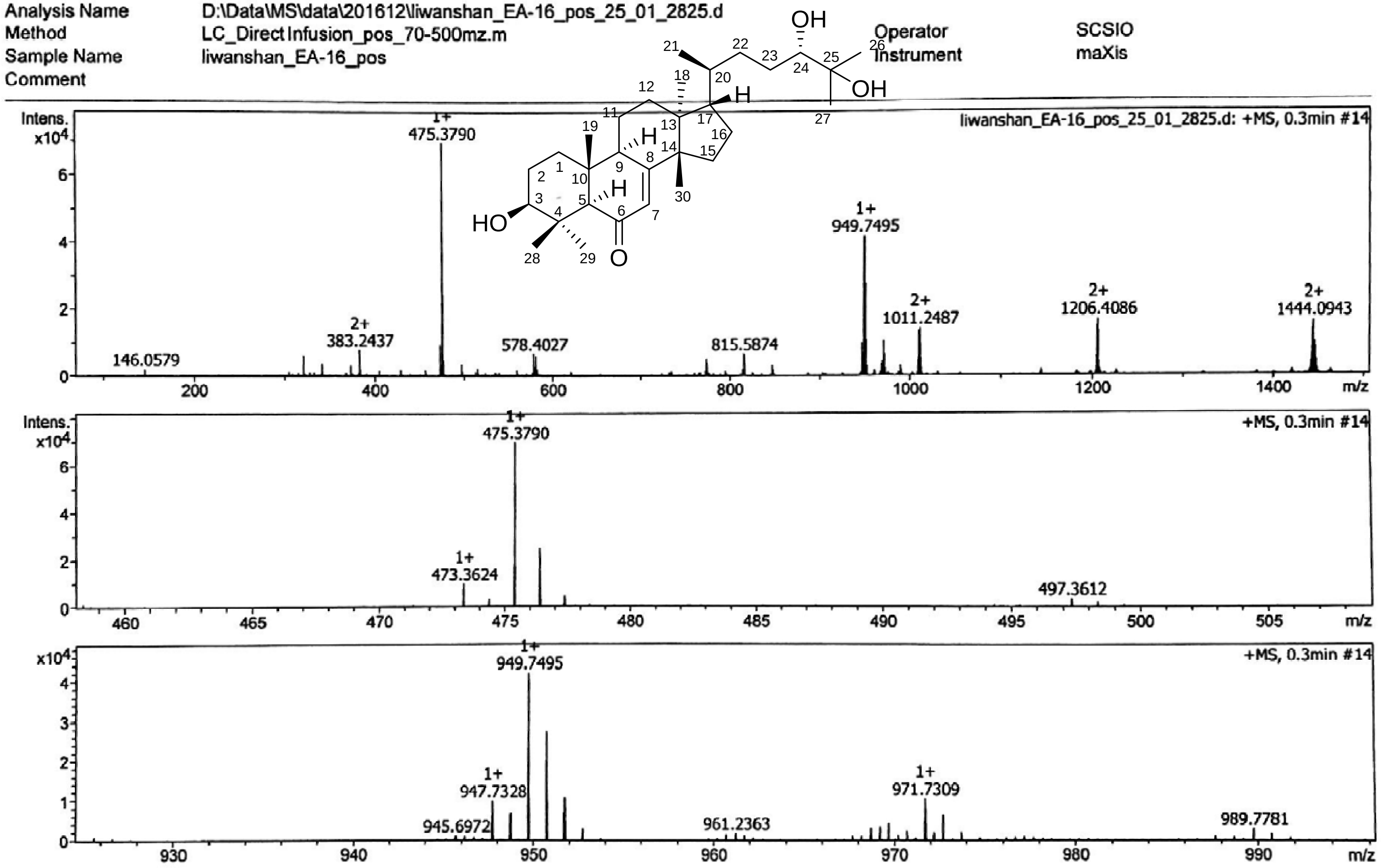

Bruker Compass DataAnalysis 4.1 printed: 12/21/2016 4:58:13 PM by: SCSIO Page 1 of 1

HR-ESIMS for compound **6**

Mass Spectrum SmartFormula Report

Analysis Info

Analysis Name: D:\Data\MS\data\201612\liwanshan_EA-16_pos_25_01_2825.d
Method: LC_Direct Infusion_pos_70-500mz.m
Sample Name: liwanshan_EA-16_pos
Comment:

Acquisition Date: 12/21/2016 4:51:43 PM
Operator: SCSIO
Instrument: maXis 255552.00029

Acquisition Parameter

Source Type	ESI	Ion Polarity	Positive	Set Nebulizer	0.4 Bar
Focus	Active	Set Capillary	4500 V	Set Dry Heater	180 °C
Scan Begin	70 m/z	Set End Plate Offset	-500 V	Set Dry Gas	4.0 l/min
Scan End	1500 m/z	Set Charging Voltage	0 V	Set Divert Valve	Waste
		Set Corona	0 nA	Set APCI Heater	0 °C

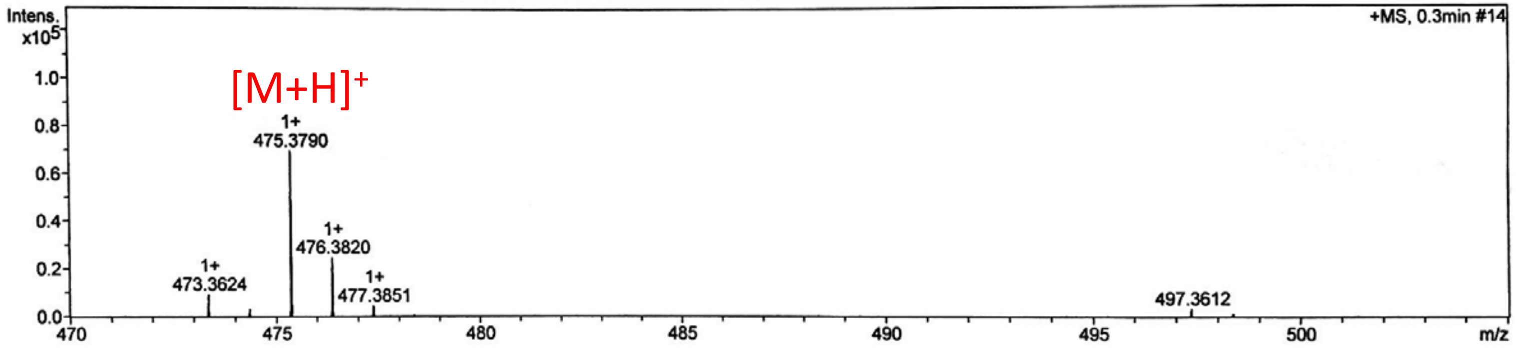

Meas. m/z	#	Ion Formula	Score	m/z	err [ppm]	err [mDa]	mSigma	rdb	e⁻ Conf	N-Rule
475.379013	1	C30H51O4	100.00	475.378187	-1.7	-0.8	10.7	5.5	even	ok
497.361200	1	C30H50NaO4	100.00	497.360131	2.2	1.1	29.1	5.5	even	ok
949.749455	1	C60H101O8	100.00	949.749097	-0.4	-0.4	12.2	10.5	even	ok
971.730887	1	C60H100NaO8	100.00	971.731041	-0.2	-0.2	30.2	10.5	even	ok

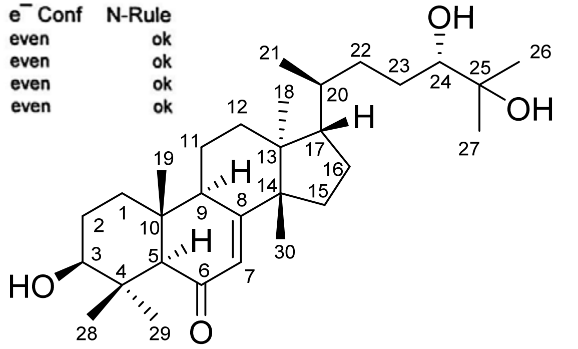

liwanshan_EA-16_pos_25_01_2825.d
Bruker Compass DataAnalysis 4.1 printed: 12/21/2016 4:57:45 PM by: SCSIO Page 1 of 1

^{1}H NMR (400 MHz) spectrum of compound **6** in $CDCl_3$

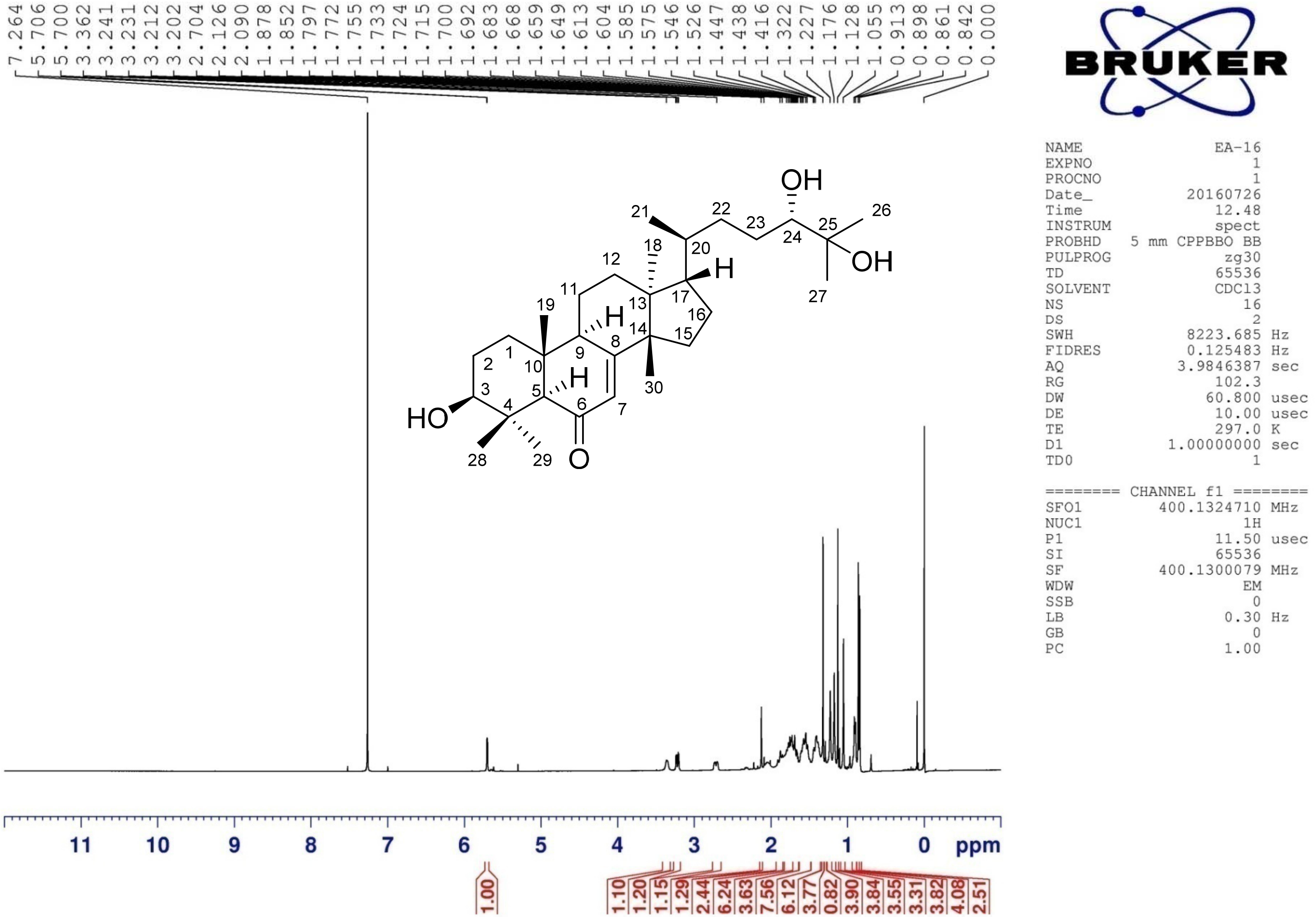

^{1}H NMR (400 MHz) spectrum of compound **6** in $CDCl_3$

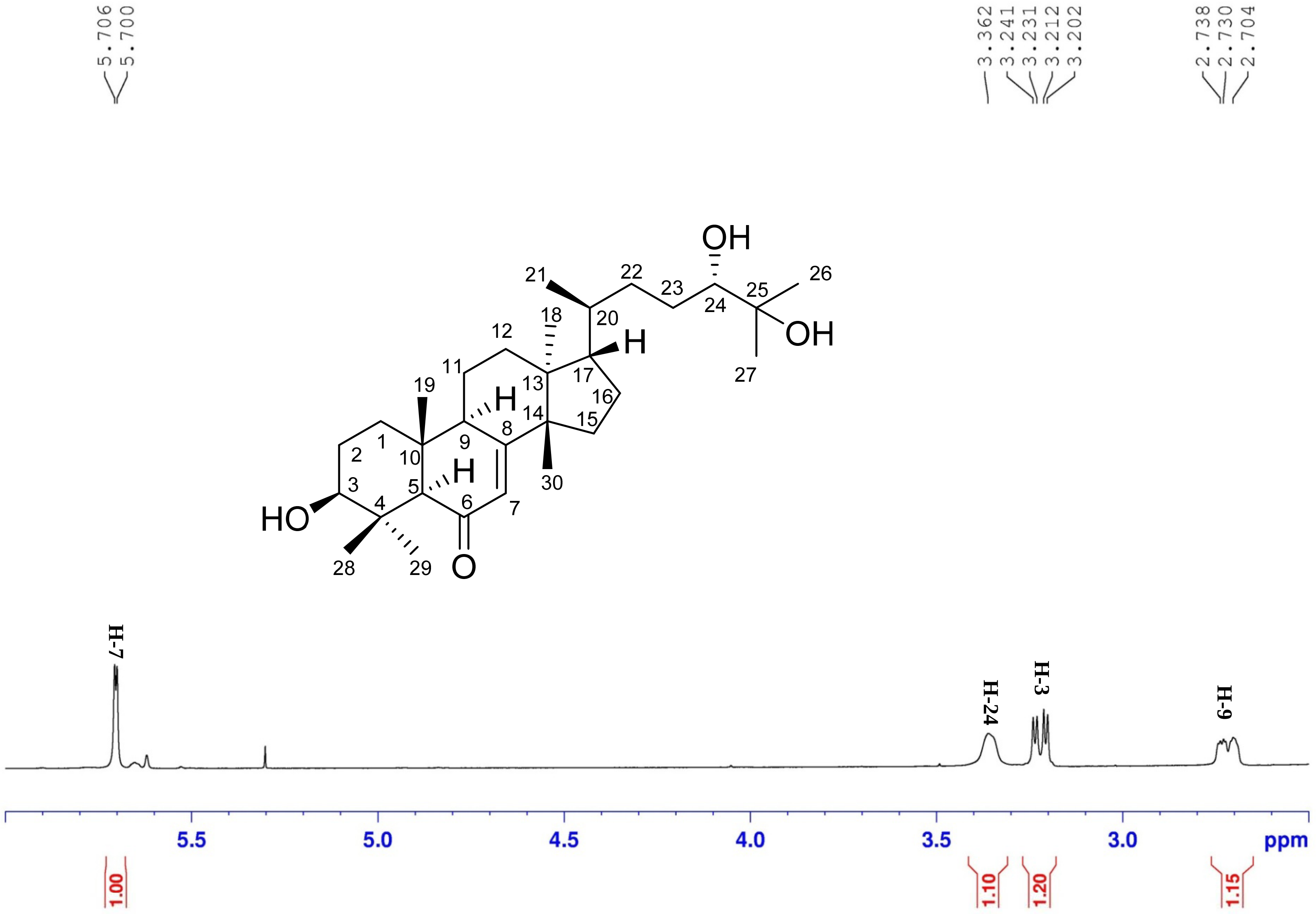

^{1}H NMR (400 MHz) spectrum of compound **6** in $CDCl_3$

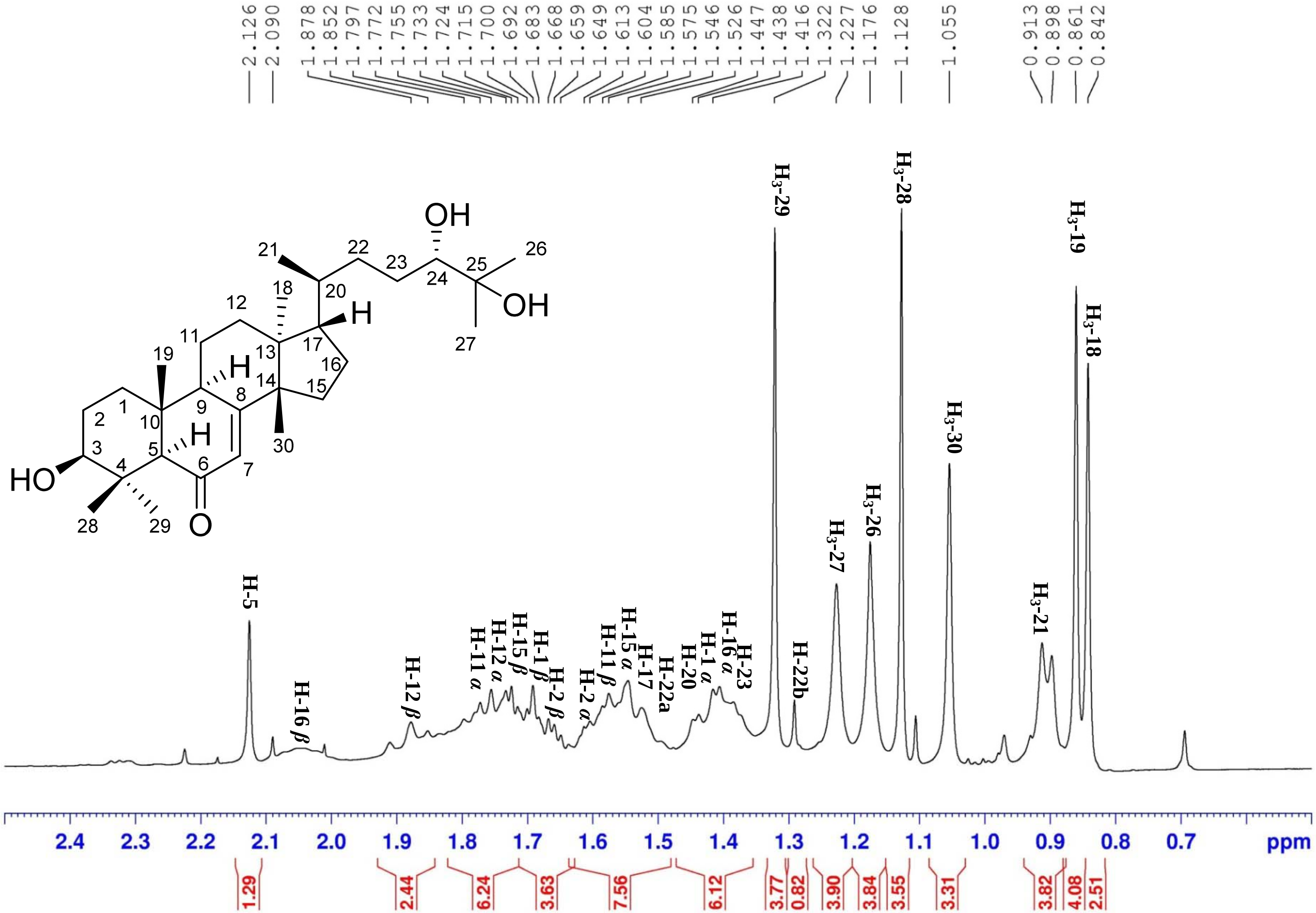

^{13}C NMR (100 MHz) spectrum of compound **6** in $CDCl_3$

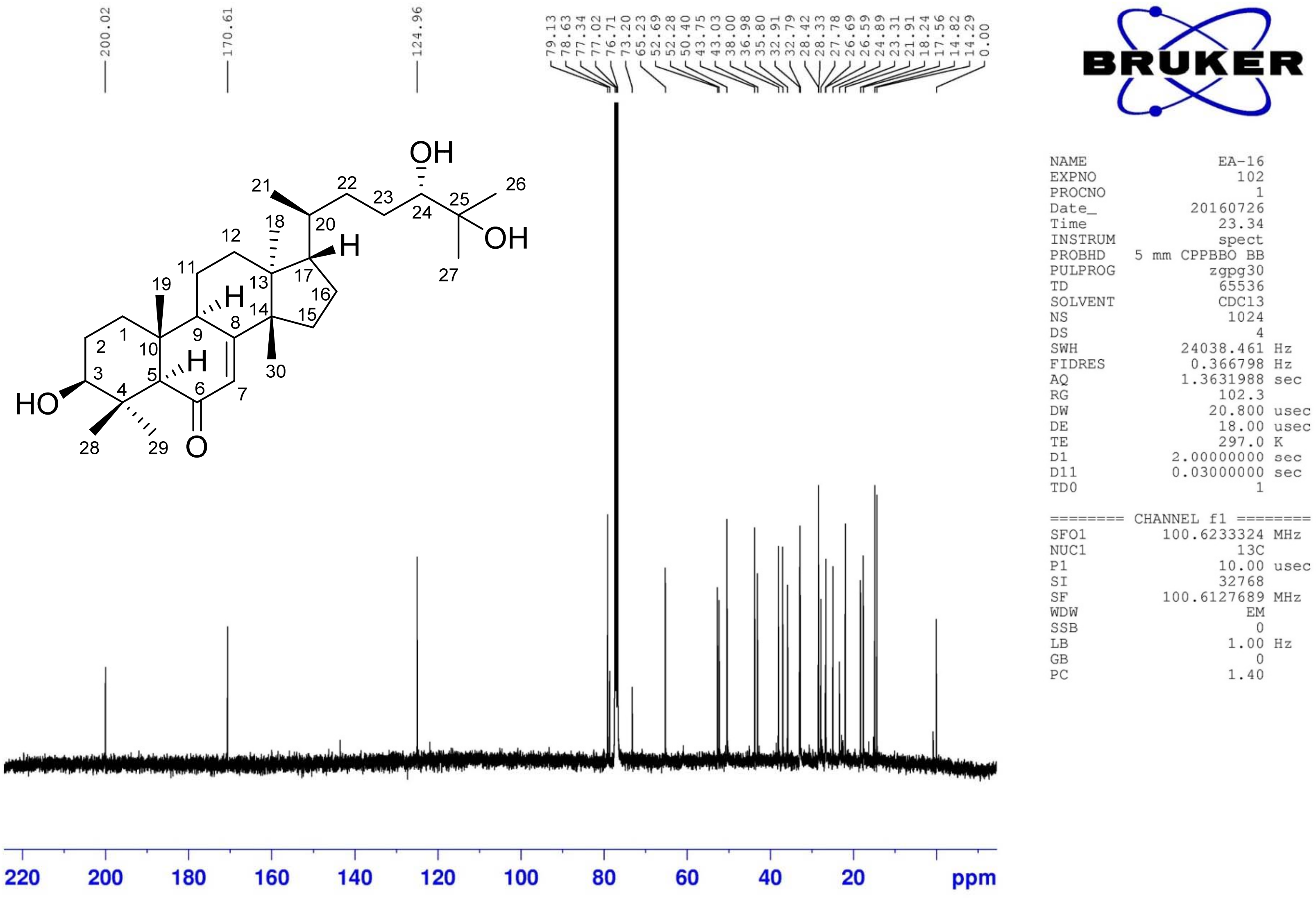

^{13}C NMR (100 MHz) spectrum of compound **6** in $CDCl_3$

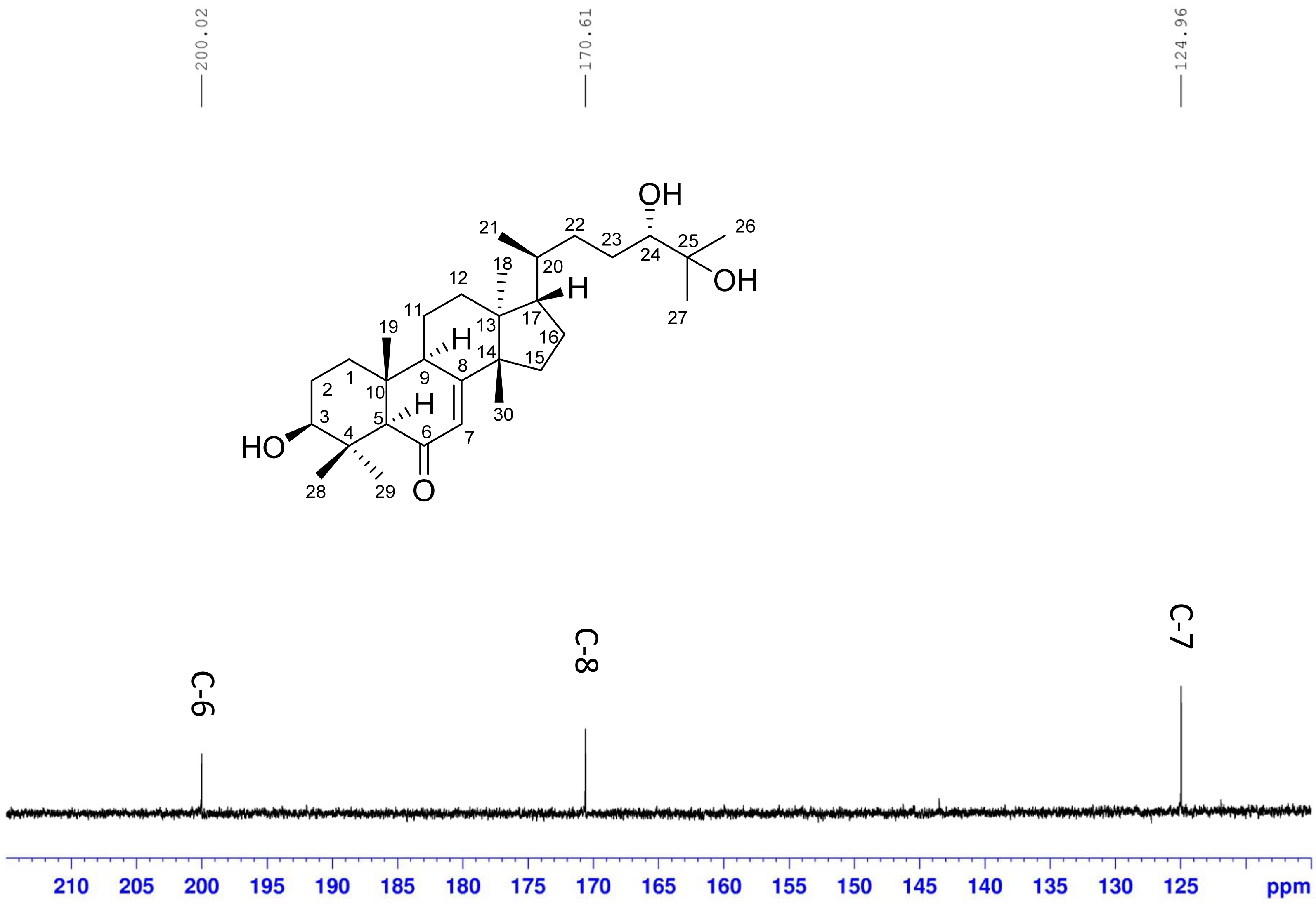

^{13}C NMR (100 MHz) spectrum of compound **6** in $CDCl_3$

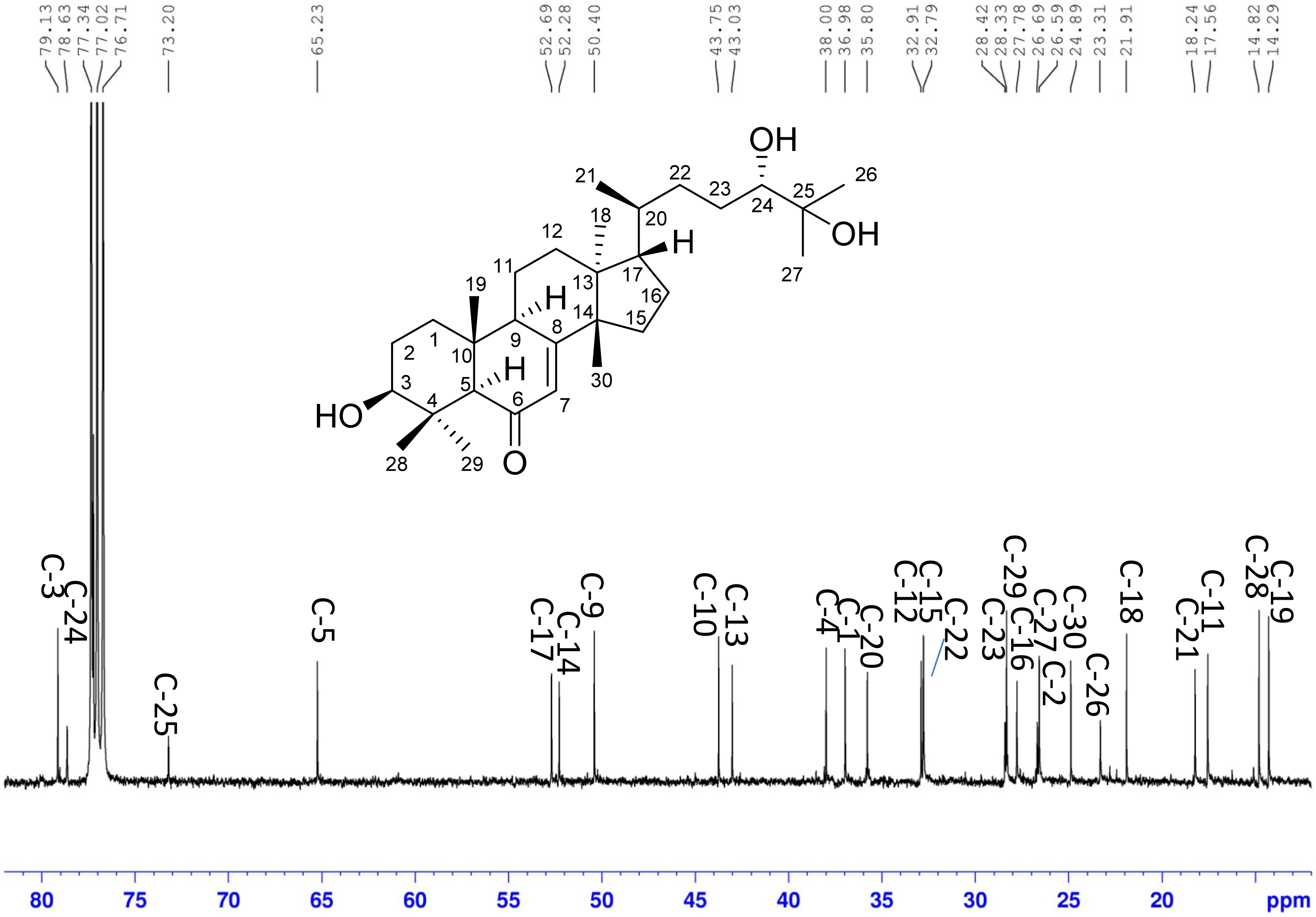

^{13}C NMR (100 MHz) spectrum of compound **6** in $CDCl_3$

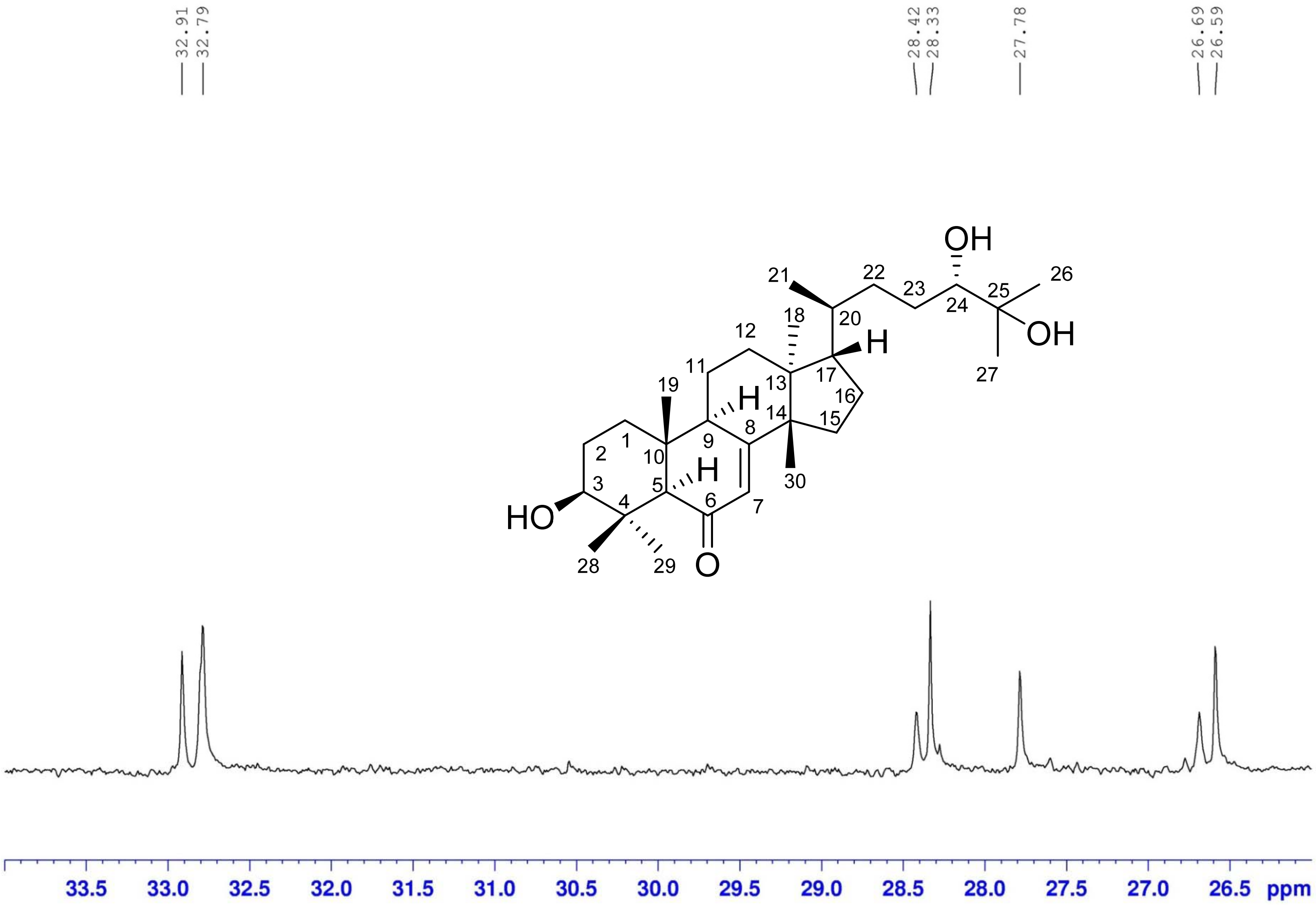

DEPT135 (100 MHz) spectrum of compound **6** in $CDCl_3$

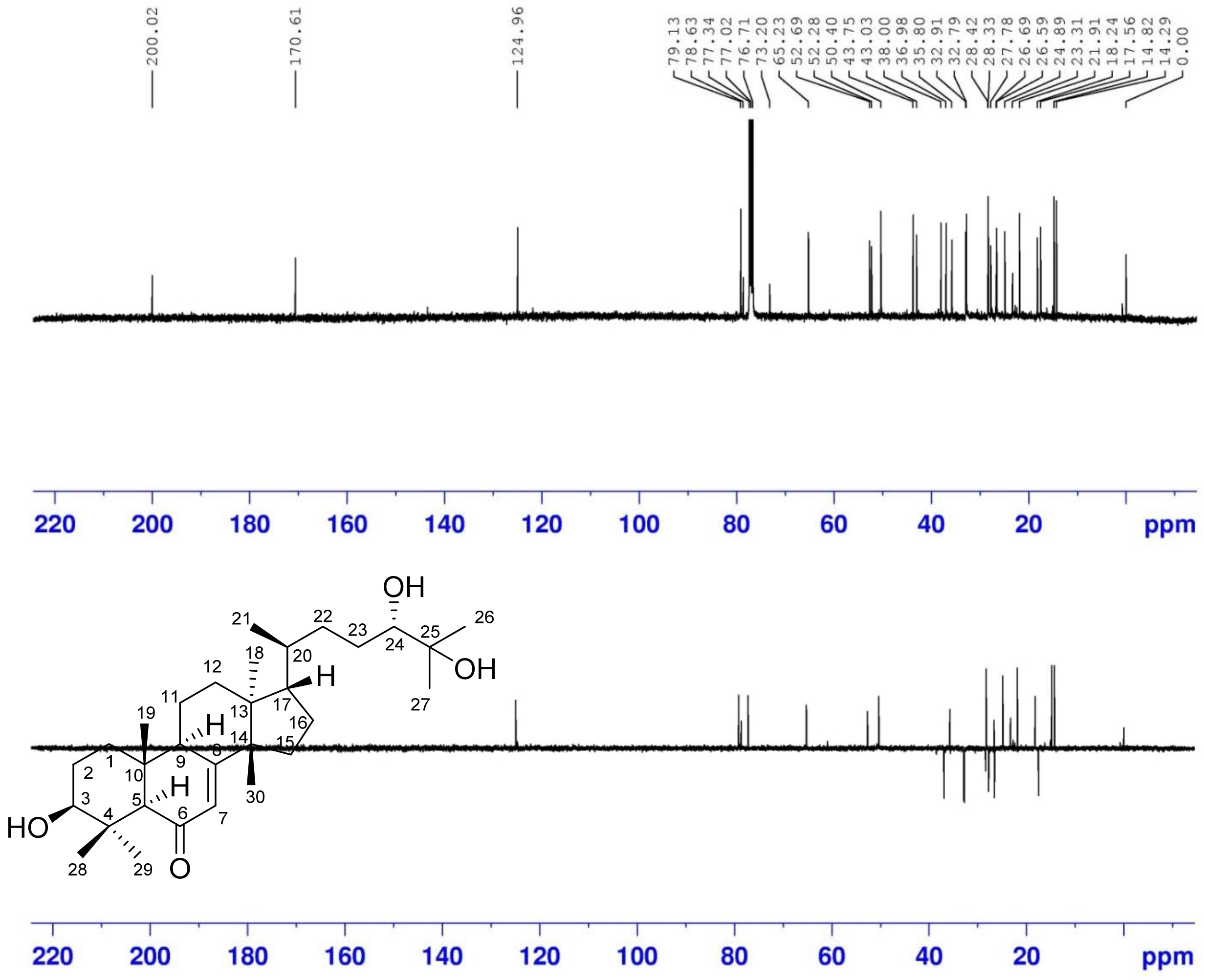


```
NAME              EA-16
EXPNO               103
PROCNO                1
Date_          20160726
Time              23.52
INSTRUM           spect
PROBHD   5 mm CPPBBO BB
PULPROG      deptsp135
TD                65536
SOLVENT           CDCl3
NS                  300
DS                    4
SWH           24038.461 Hz
FIDRES         0.366798 Hz
AQ            1.3631988 sec
RG               130.26
DW               20.800 usec
DE                18.00 usec
TE                297.0 K
CNST2       145.0000000
D1           2.00000000 sec
D2           0.00344828 sec
D12          0.00002000 sec
TD0                   1

======== CHANNEL f1 ========
SFO1        100.6233324 MHz
NUC1                13C
P1                10.00 usec
P13             2000.00 usec
SI                32768
SF          100.6127689 MHz
WDW                  EM
SSB                   0
LB                 1.00 Hz
GB                    0
PC                 1.40
```

DEPT135 (100 MHz) spectrum of compound **6** in $CDCl_3$

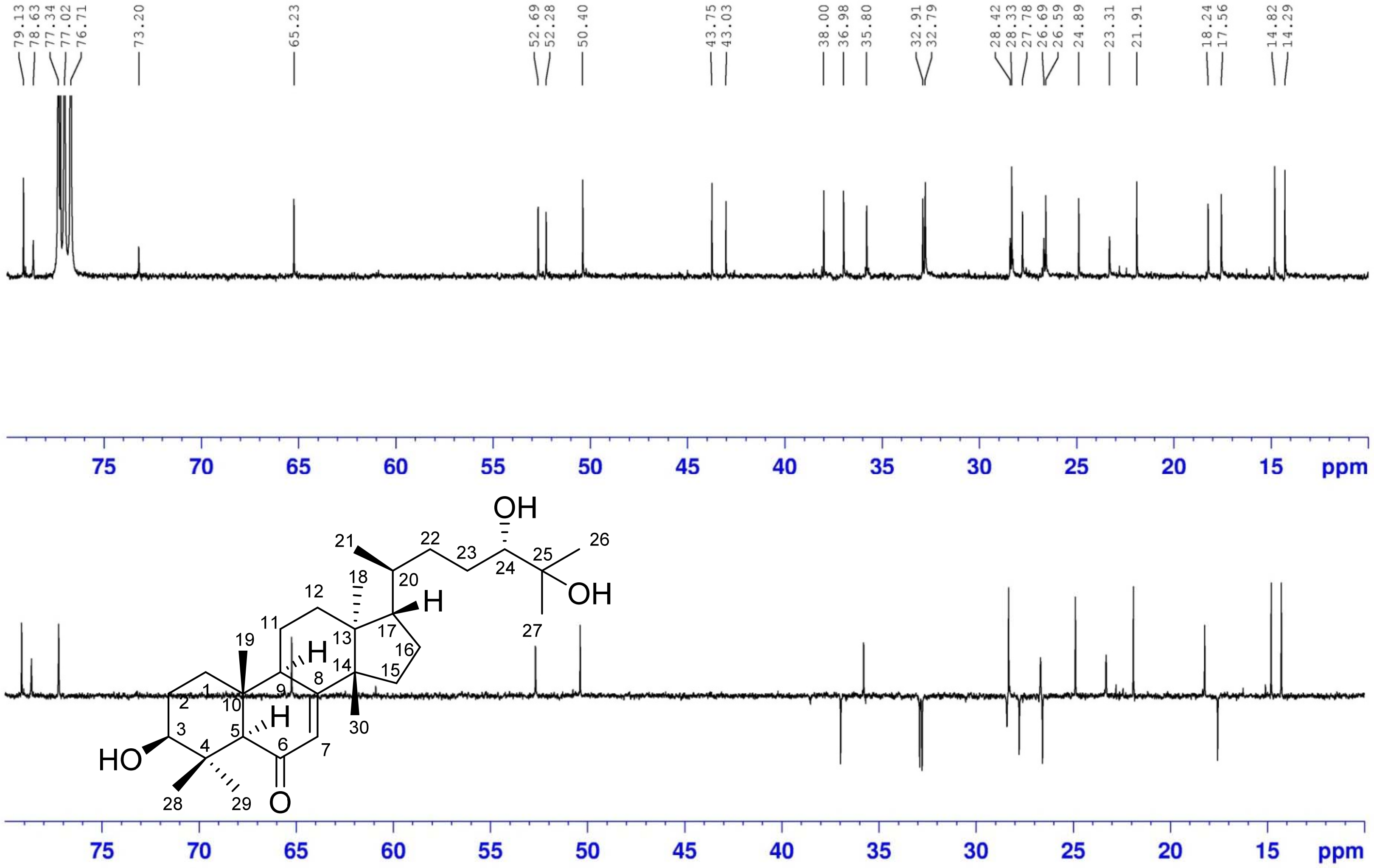

^{1}H-^{1}H COSY (400 MHz) spectrum of compound **6** in $CDCl_3$

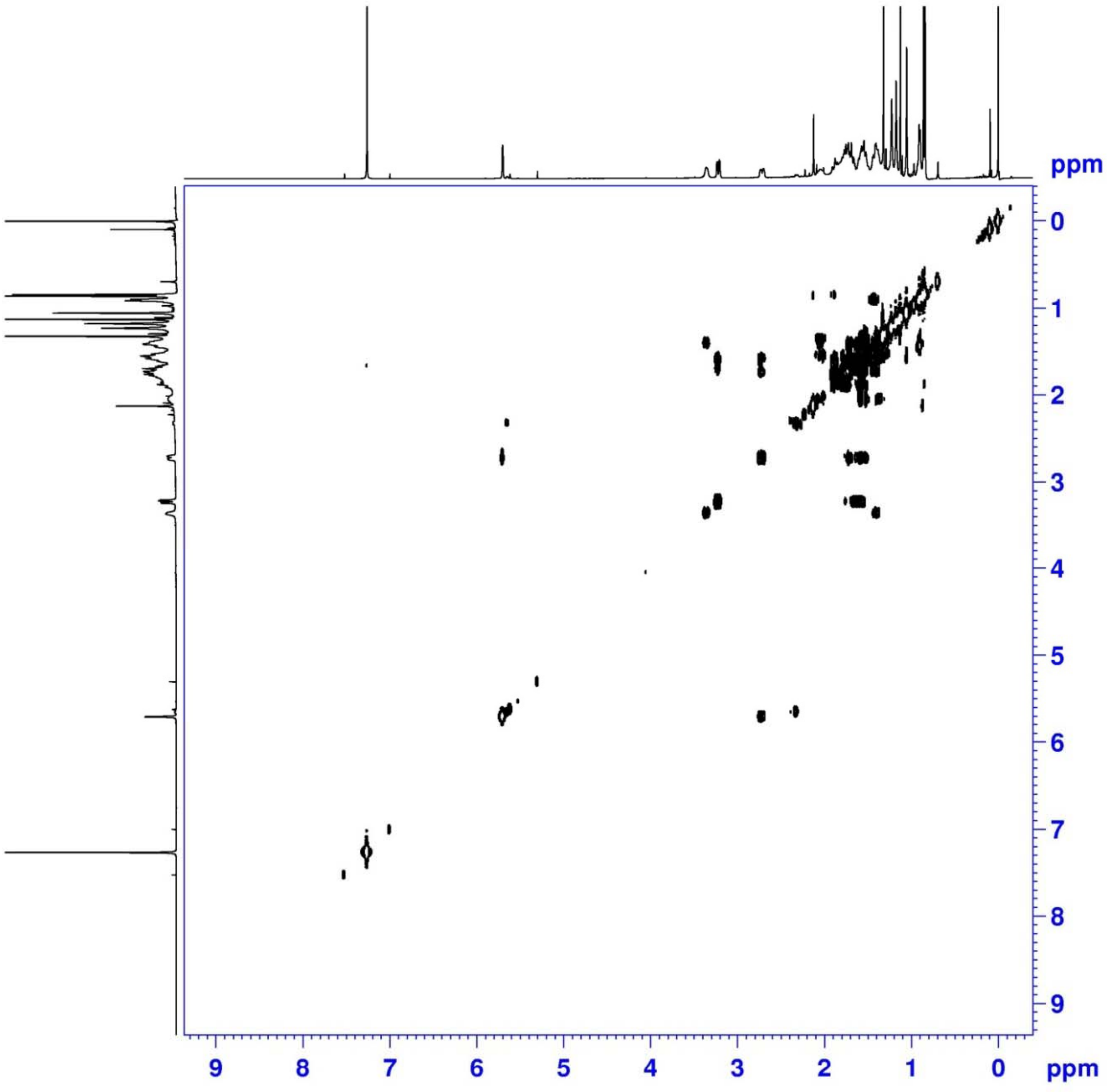

NAME	EA-16
EXPNO	4
PROCNO	1
Date_	20160806
Time	5.58
INSTRUM	spect
PROBHD	5 mm CPPBBO BB
PULPROG	cosygpppqf
TD	2048
SOLVENT	CDCl3
NS	8
DS	8
SWH	3906.250 Hz
FIDRES	1.907349 Hz
AQ	0.2621940 sec
RG	208.5
DW	128.000 usec
DE	10.00 usec
TE	297.0 K
D0	0.00000300 sec
D1	1.89678097 sec
D11	0.03000000 sec
D12	0.00002000 sec
D13	0.00000400 sec
D16	0.00020000 sec
IN0	0.00025600 sec
======== CHANNEL f1 ========	
SFO1	400.1318006 MHz
NUC1	1H
P0	11.50 usec
P1	11.50 usec
P17	2500.00 usec
ND0	1
TD	128
SFO1	400.1318 MHz
FIDRES	30.517578 Hz
SW	9.762 ppm
FnMODE	QF
SI	1024
SF	400.1300062 MHz
WDW	QSINE
SSB	0
LB	0.00 Hz
GB	0
PC	1.40
SI	1024
MC2	QF
SF	400.1300086 MHz
WDW	QSINE
SSB	0

^{1}H-^{1}H COSY (400 MHz) spectrum of compound **6** in $CDCl_3$

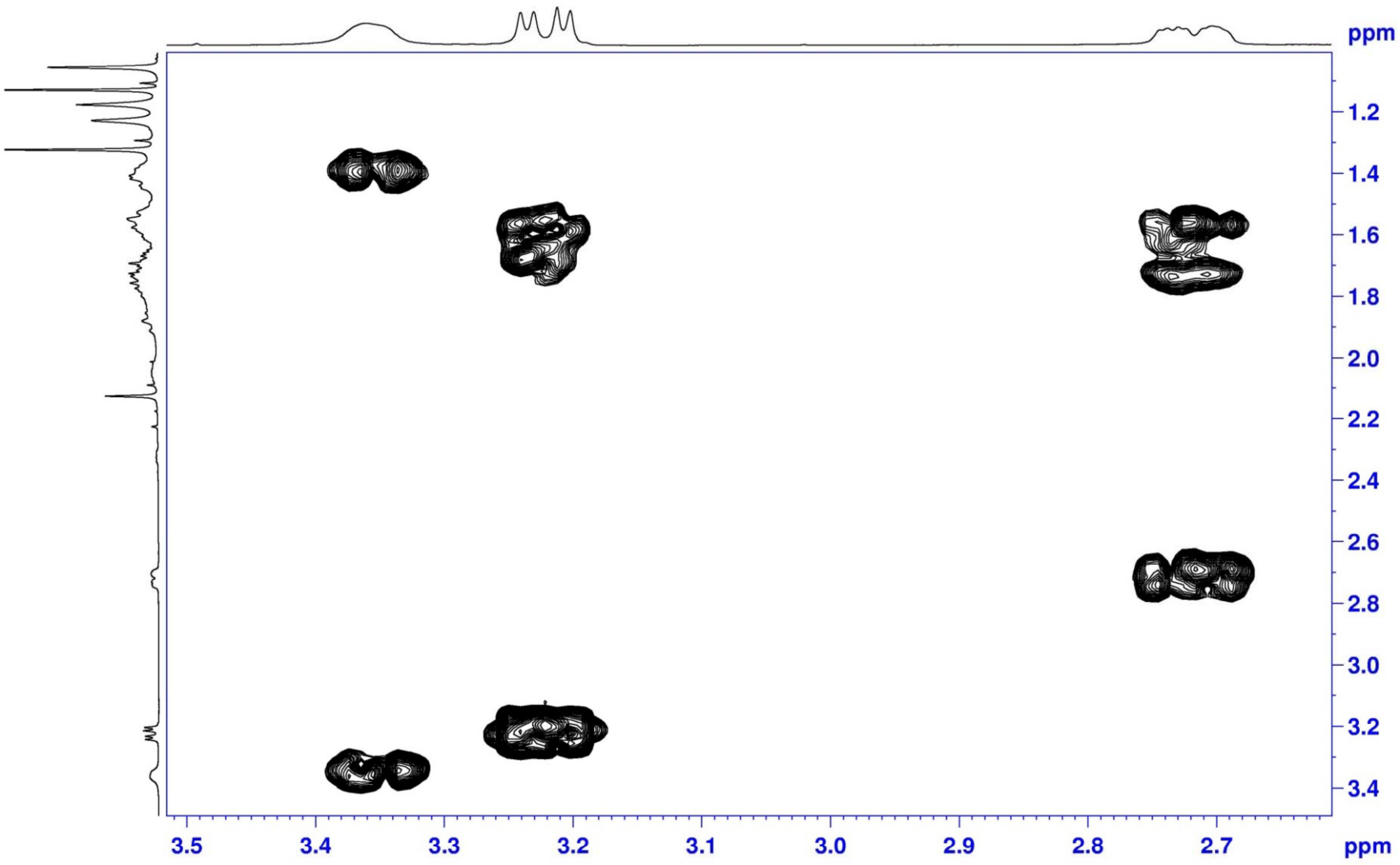

^{1}H-^{1}H COSY (400 MHz) spectrum of compound **6** in $CDCl_3$

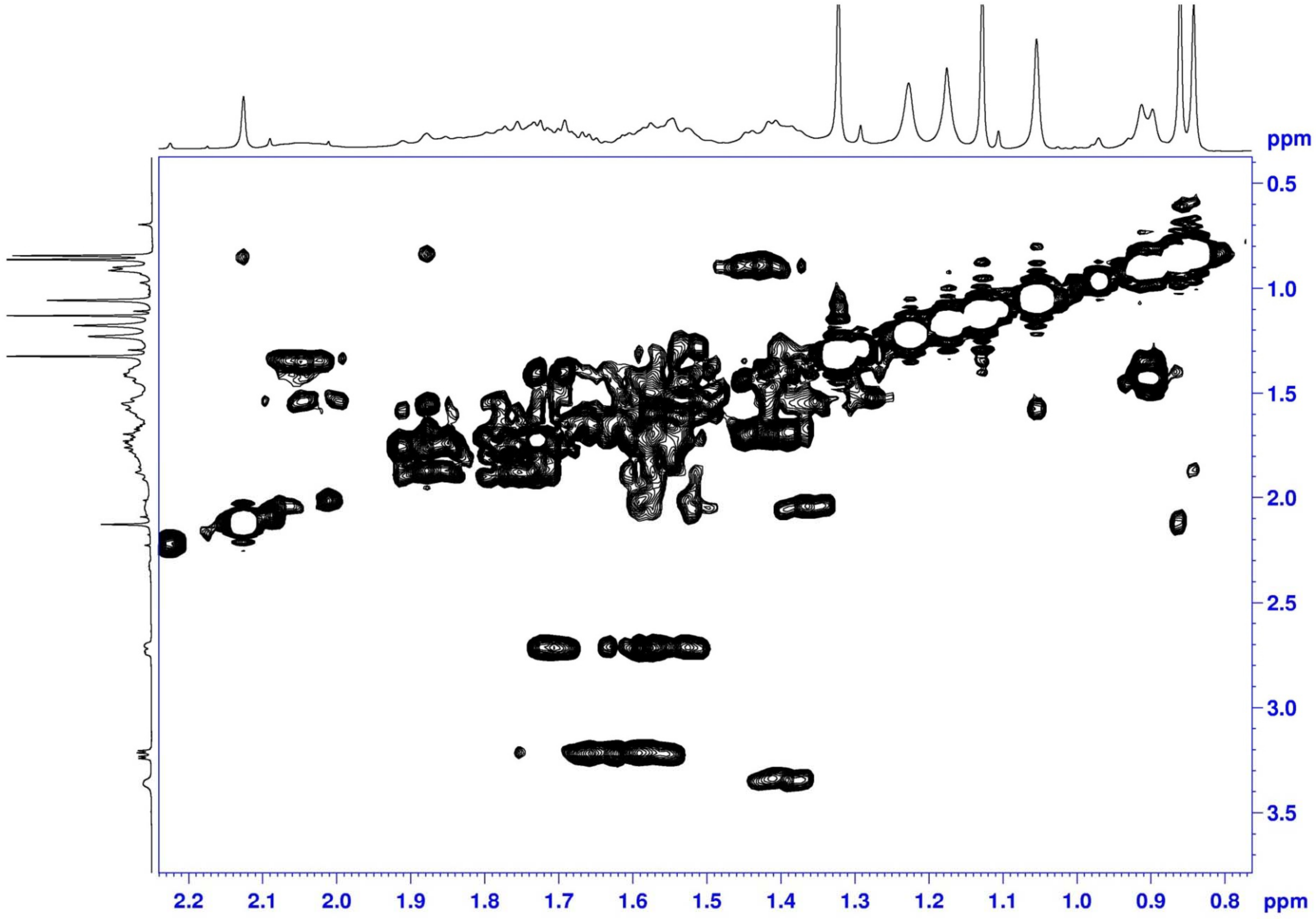

^{1}H-^{1}H COSY (400 MHz) spectrum of compound **6** in $CDCl_3$

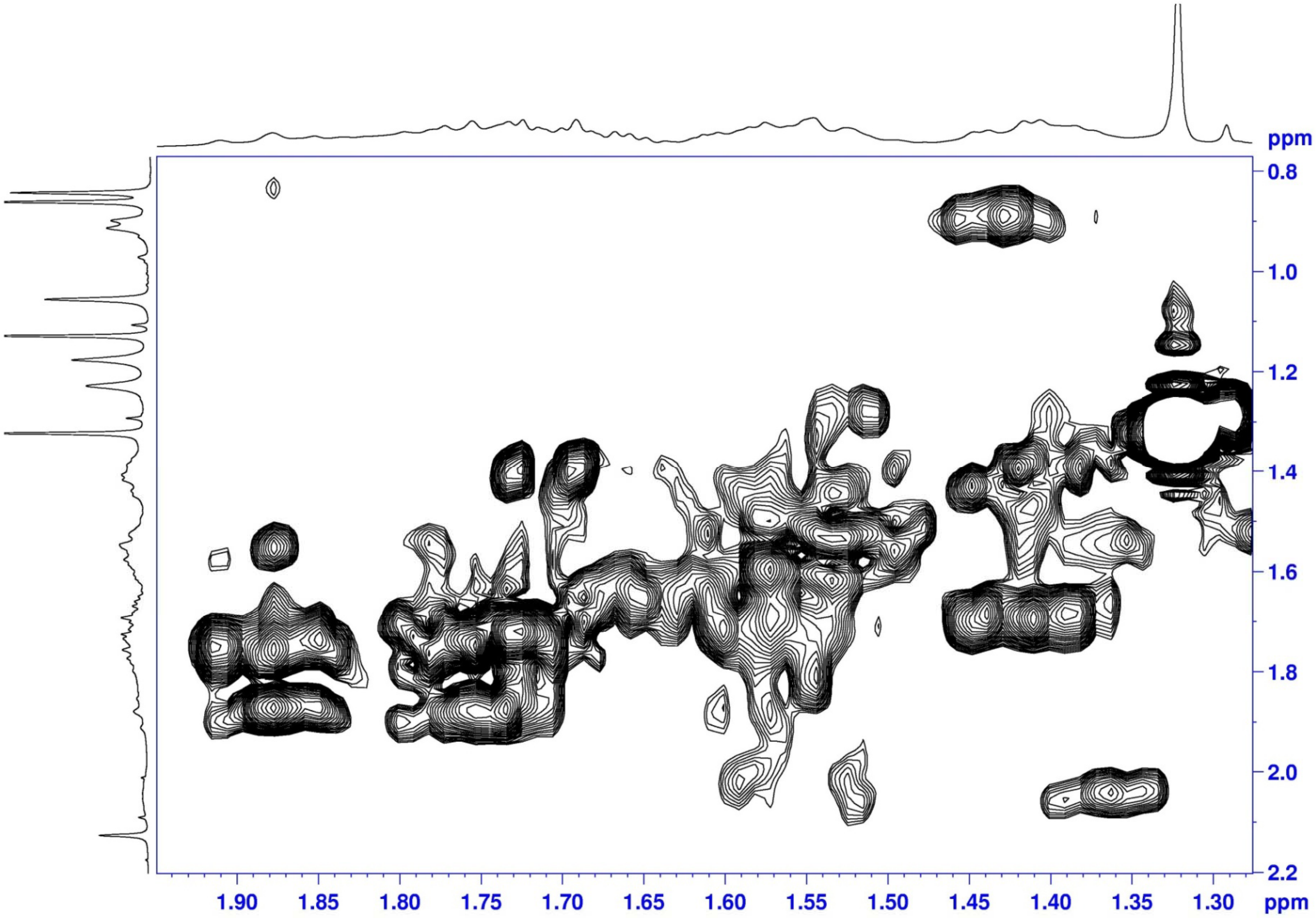

HSQC (400 MHz) spectrum of compound **6** in $CDCl_3$

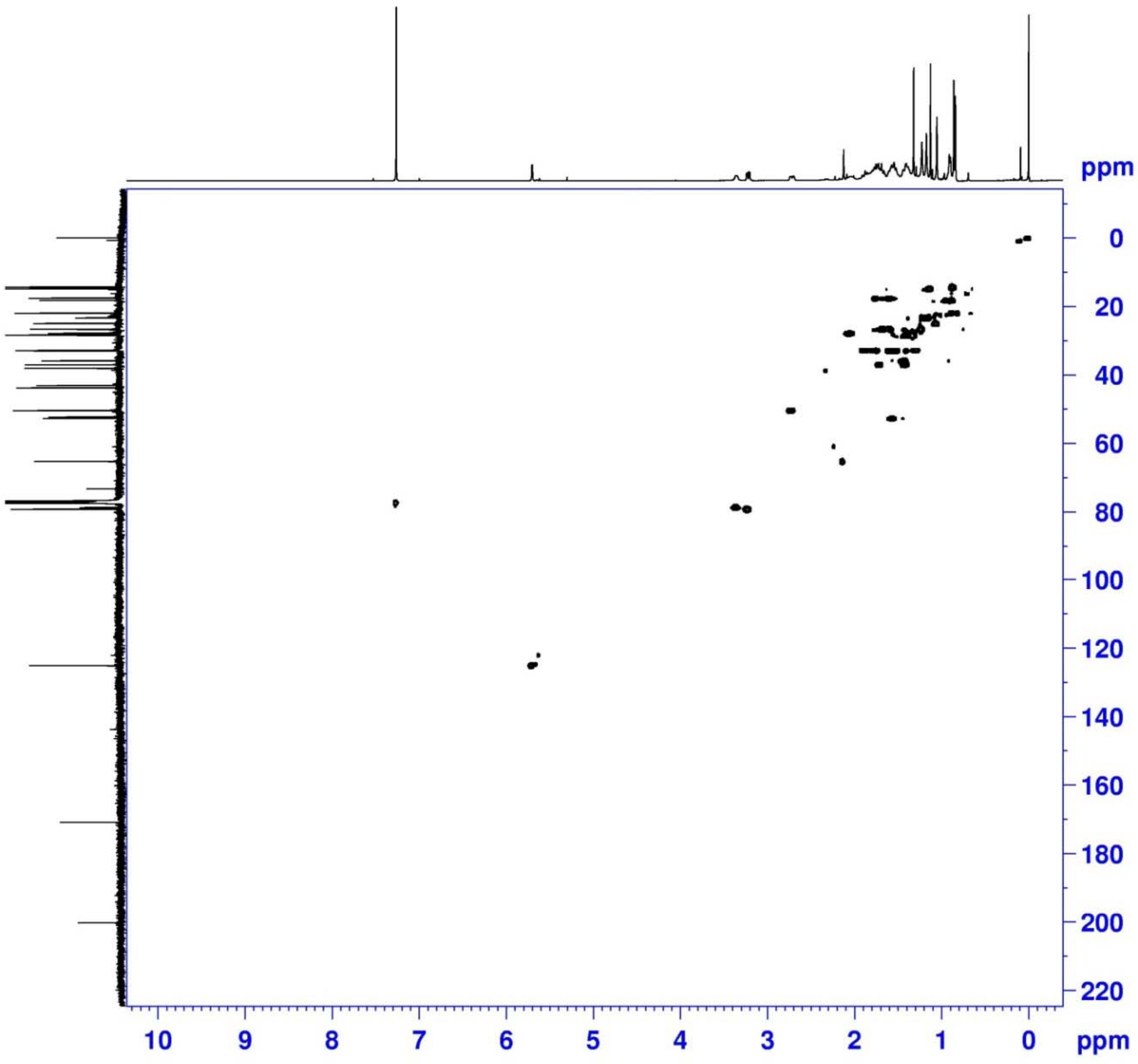

NAME	EA-16	
EXPNO	5	
PROCNO	1	
Date_	20160806	
Time	6.37	
INSTRUM	spect	
PROBHD	5 mm CPPBBO BB	
PULPROG	hsqcetgpsi2	
TD	1024	
SOLVENT	CDCl3	
NS	16	
DS	16	
SWH	4302.926	Hz
FIDRES	4.202076	Hz
AQ	0.1190388	sec
RG	208.5	
DW	116.200	usec
DE	10.00	usec
TE	297.0	K
CNST2	145.0000000	
D0	0.00000300	sec
D1	1.46497905	sec
D4	0.00172414	sec
D11	0.03000000	sec
D16	0.00020000	sec
D24	0.00086207	sec
IN0	0.00002080	sec
ZGOPTNS		
======== CHANNEL f1 ========		
SFO1	400.1320007	MHz
NUC1	1H	
P1	11.50	usec
P2	23.00	usec
P28	0.00	usec
ND0	2	
TD	256	
SFO1	100.6233	MHz
FIDRES	93.900238	Hz
SW	238.896	ppm
FnMODE	Echo-Antiecho	
SI	1024	
SF	400.1300059	MHz
WDW	QSINE	
SSB	2	
LB	0.00	Hz
GB	0	
PC	1.40	
SI	1024	
MC2	echo-antiecho	
SF	100.6127568	MHz
WDW	QSINE	
SSB	2	

HSQC (400 MHz) spectrum of compound **6** in $CDCl_3$

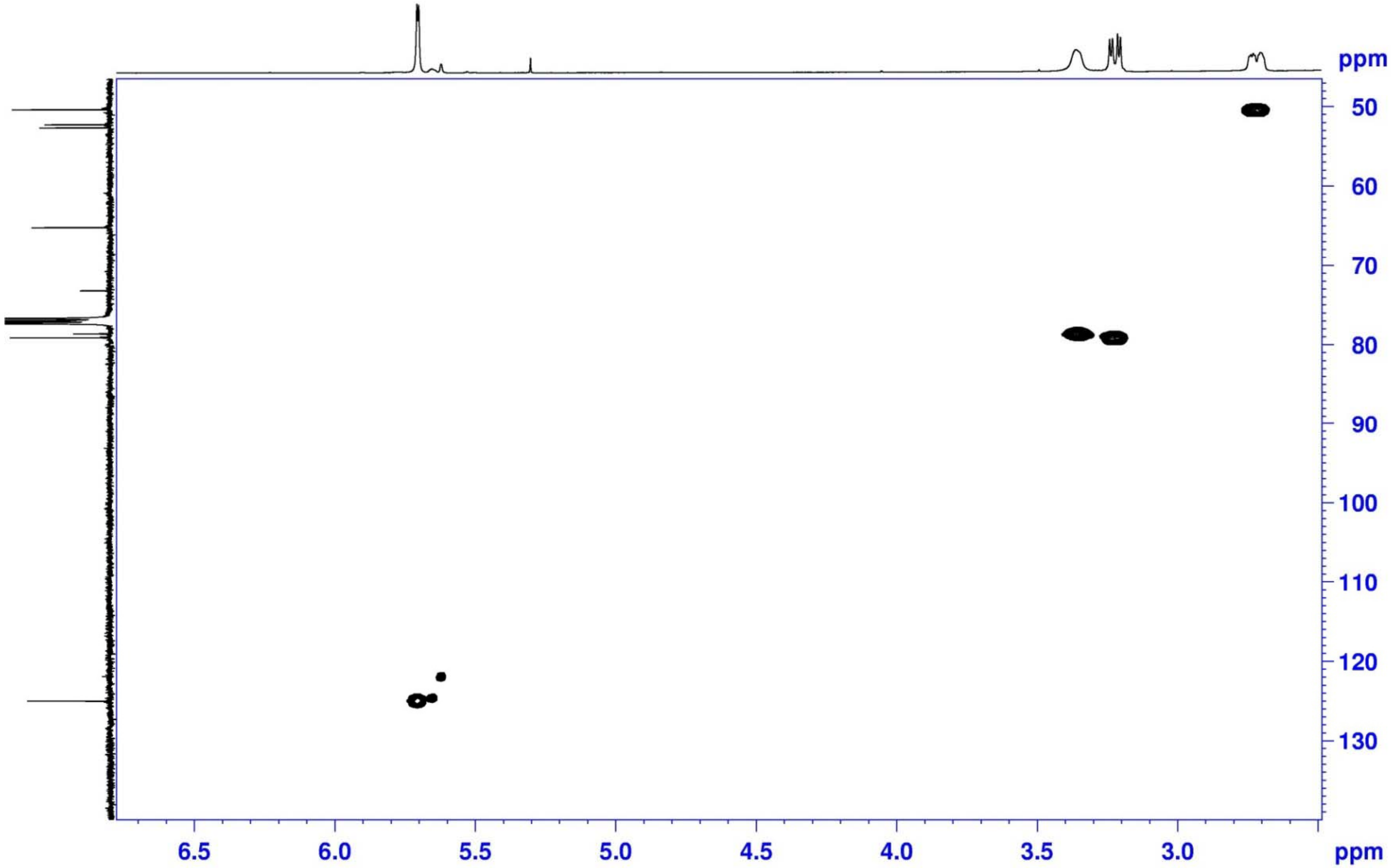

HSQC (400 MHz) spectrum of compound **6** in $CDCl_3$

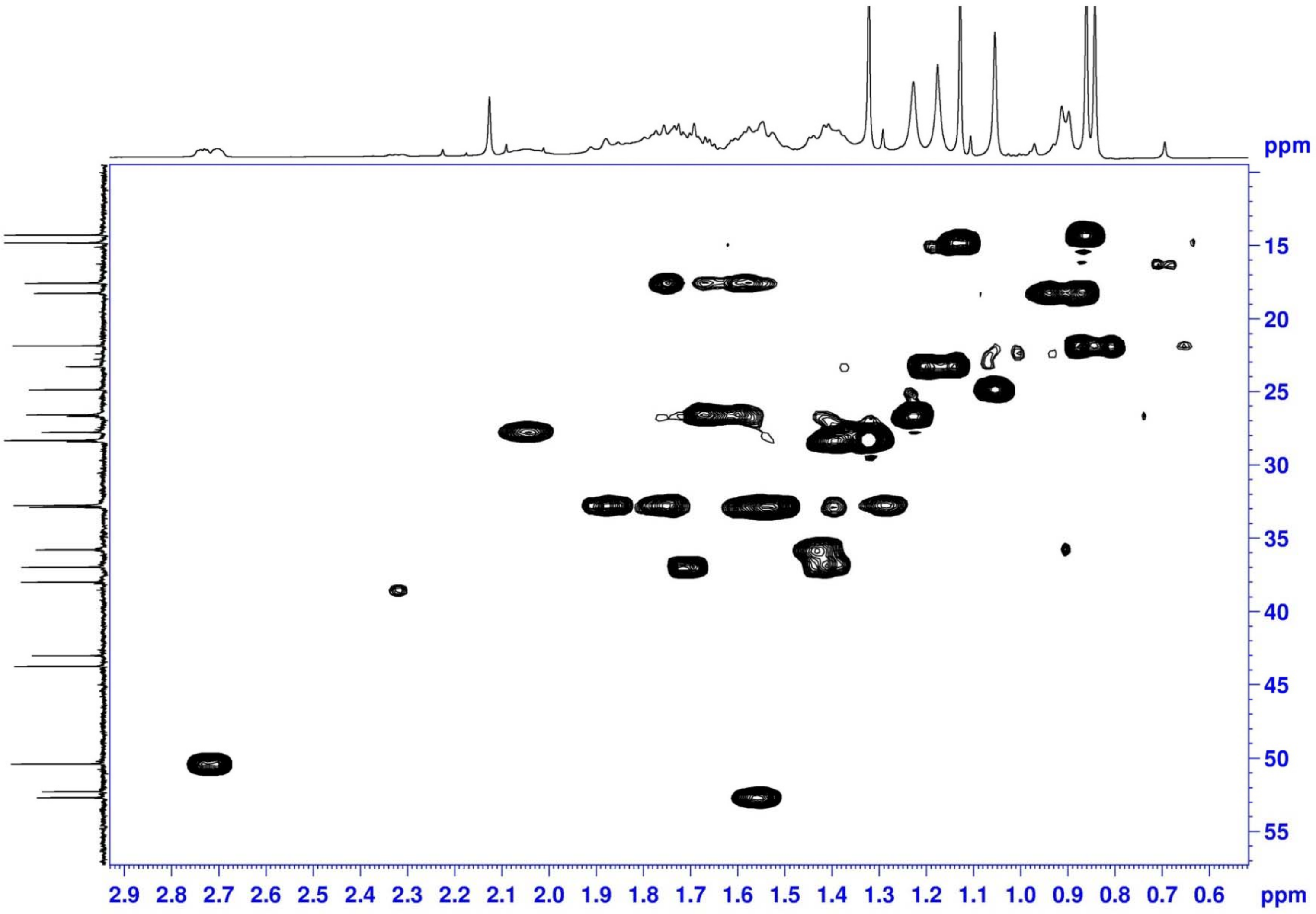

HSQC (400 MHz) spectrum of compound **6** in $CDCl_3$

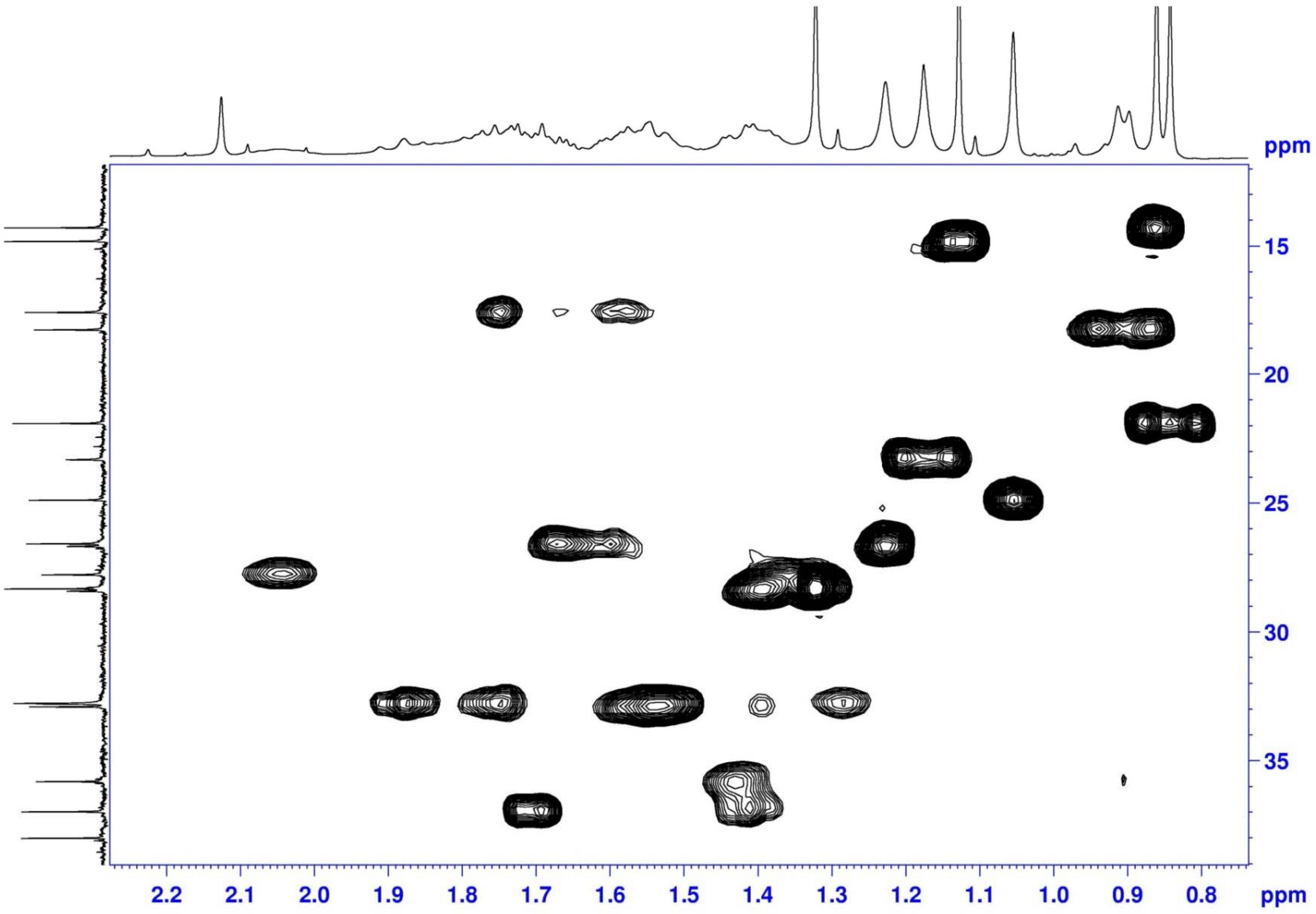

HMBC (400 MHz) spectrum of compound **6** in $CDCl_3$

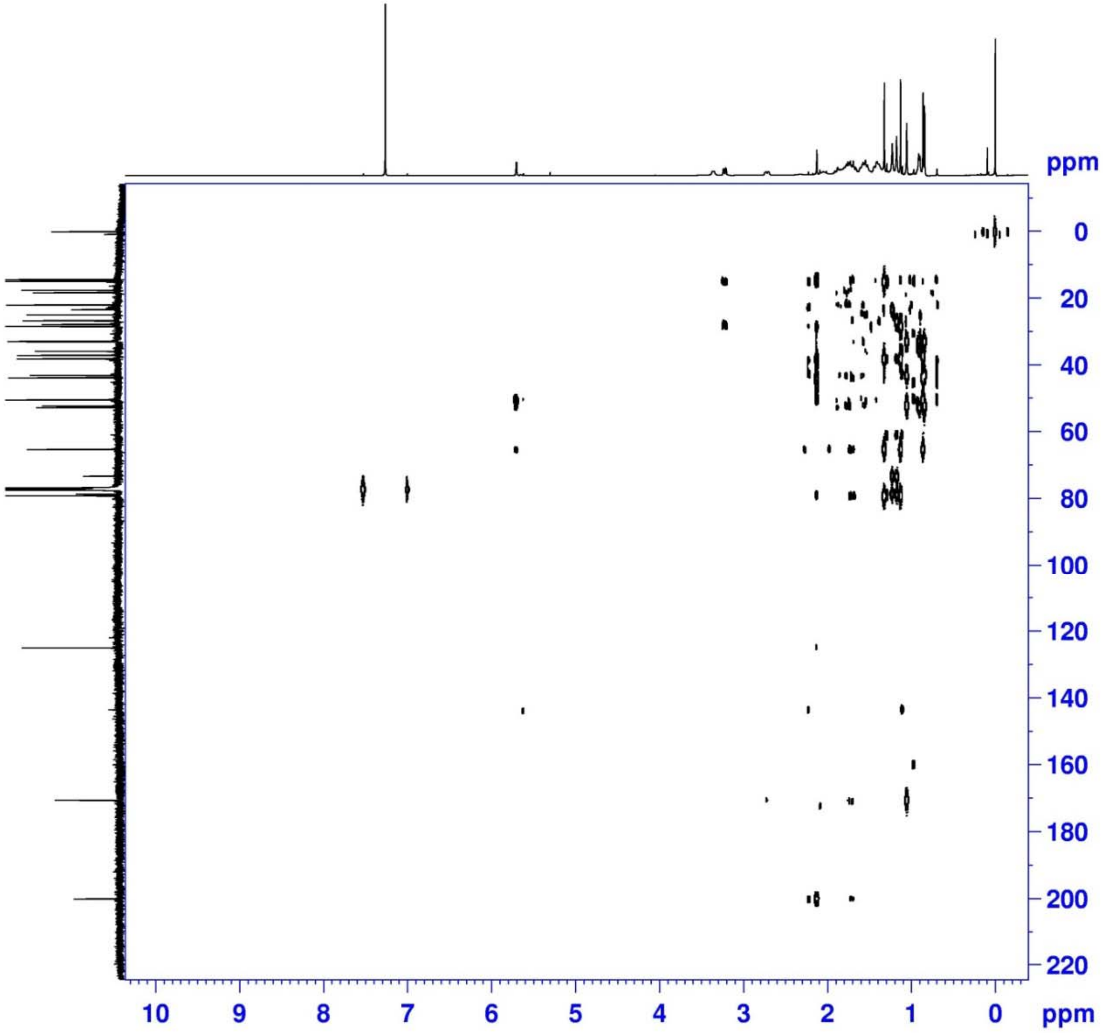


```
N[illegible]
EXPNO              6
PROCNO             1
Date_       20160806
Time            8.28
INSTRUM        spect
PROBHD   5 mm CPPBBO BB
PULPROG   hmbcgplpndqf
TD              4096
SOLVENT        CDCl3
NS                32
DS                16
SWH         5197.505 Hz
FIDRES      1.268922 Hz
AQ         0.3940852 sec
RG             208.5
DW            96.200 usec
DE             10.00 usec
TE             297.0 K
CNST2    145.0000000
CNST13    10.0000000
D0        0.00000300 sec
D1        1.50000000 sec
D2        0.00344828 sec
D6        0.05000000 sec
D16       0.00020000 sec
IN0       0.00002080 sec

======== CHANNEL f1 ========
SFO1     400.1323208 MHz
NUC1              1H
P1             11.50 usec
P2             23.00 usec
ND0                2
TD               128
SFO1     100.6233 MHz
FIDRES    187.800476 Hz
SW           238.896 ppm
FnMODE            QF
SI              2048
SF       400.1300066 MHz
WDW             SINE
SSB                0
LB              0.00 Hz
GB                 0
PC              1.40
SI              1024
MC2               QF
SF       100.6127622 MHz
WDW             SINE
SSB                0
LB              0.00 Hz
GB                 0
```

HMBC (400 MHz) spectrum of compound **6** in $CDCl_3$

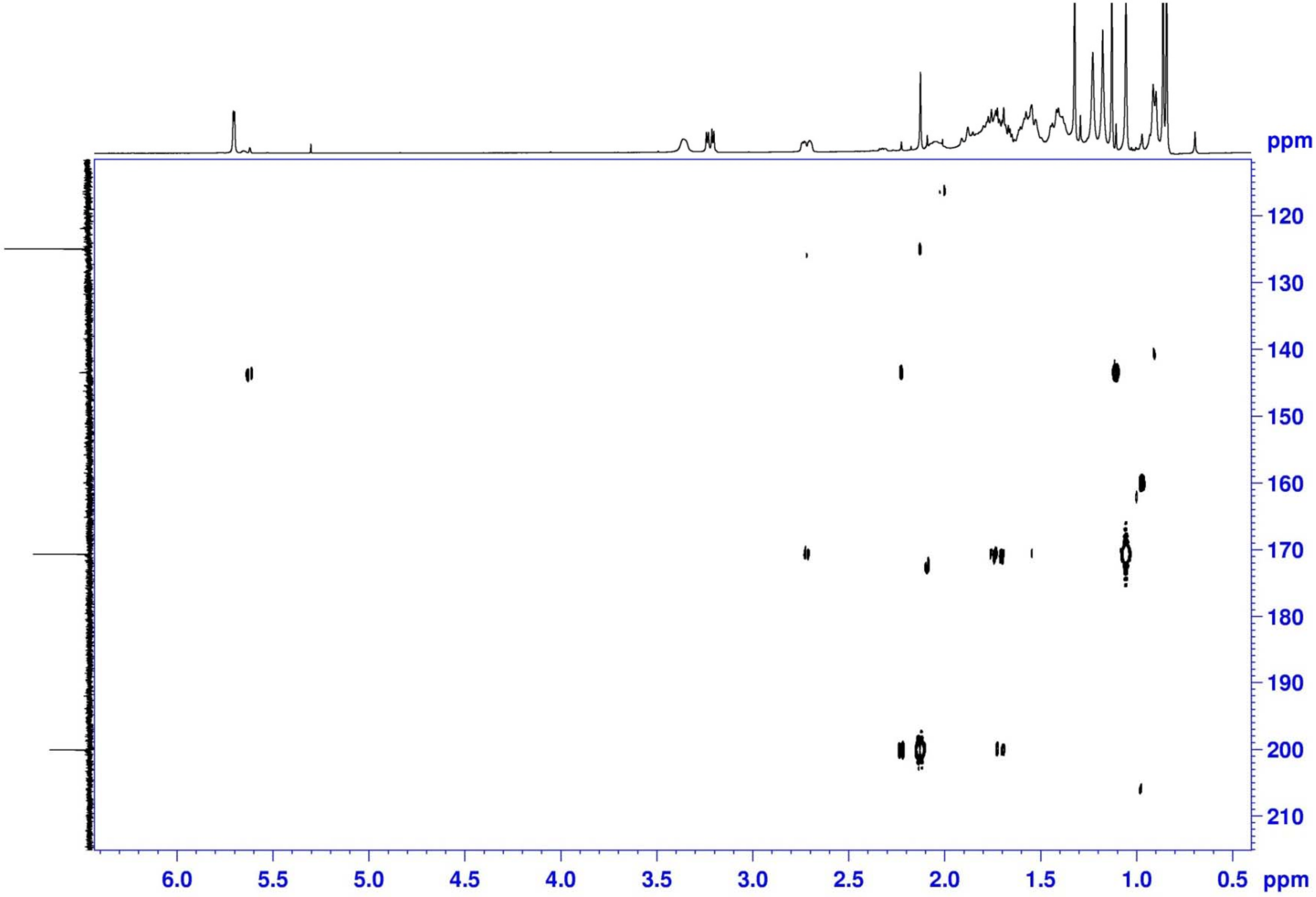

HMBC (400 MHz) spectrum of compound **6** in $CDCl_3$

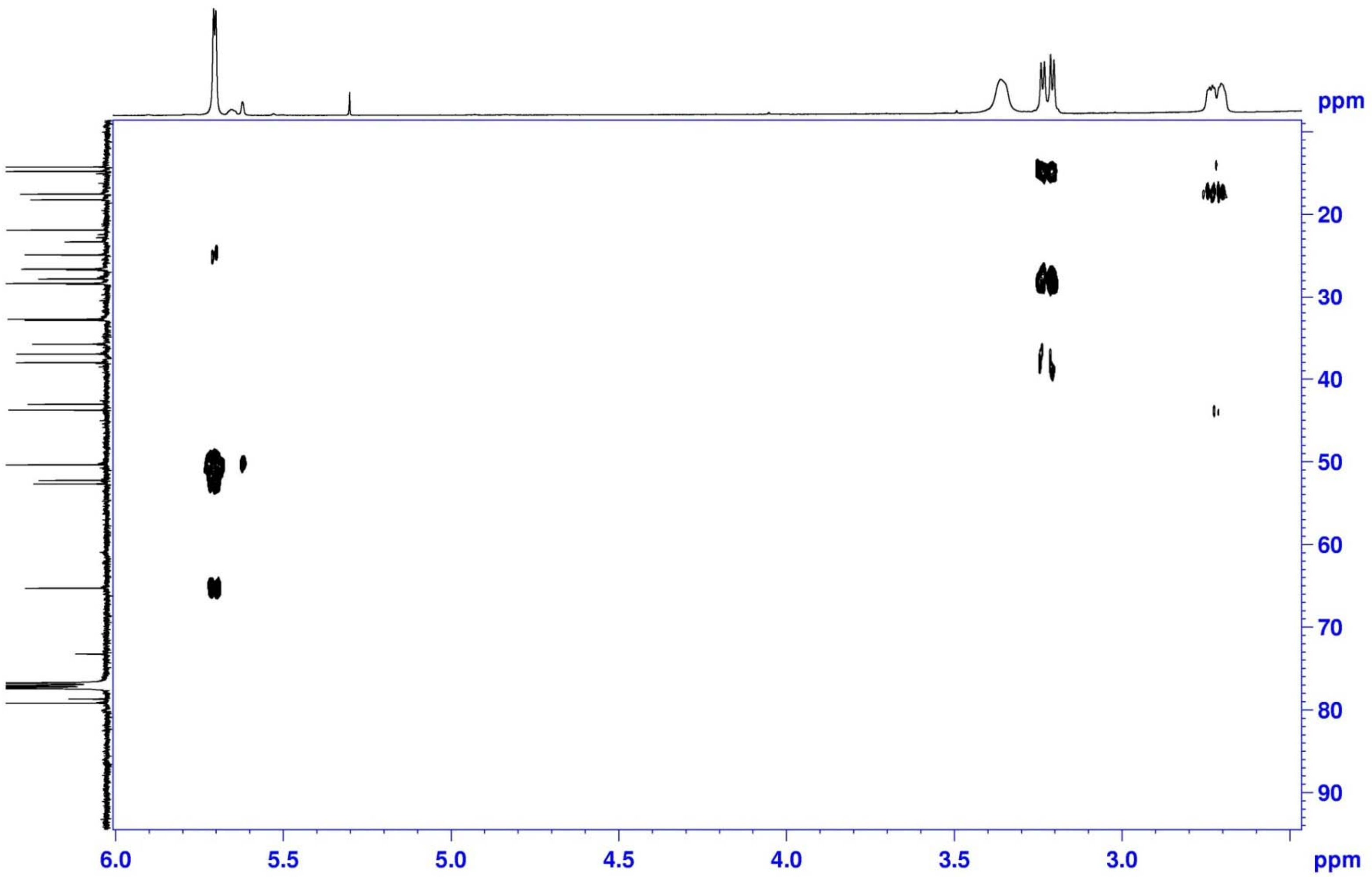

HMBC (400 MHz) spectrum of compound **6** in $CDCl_3$

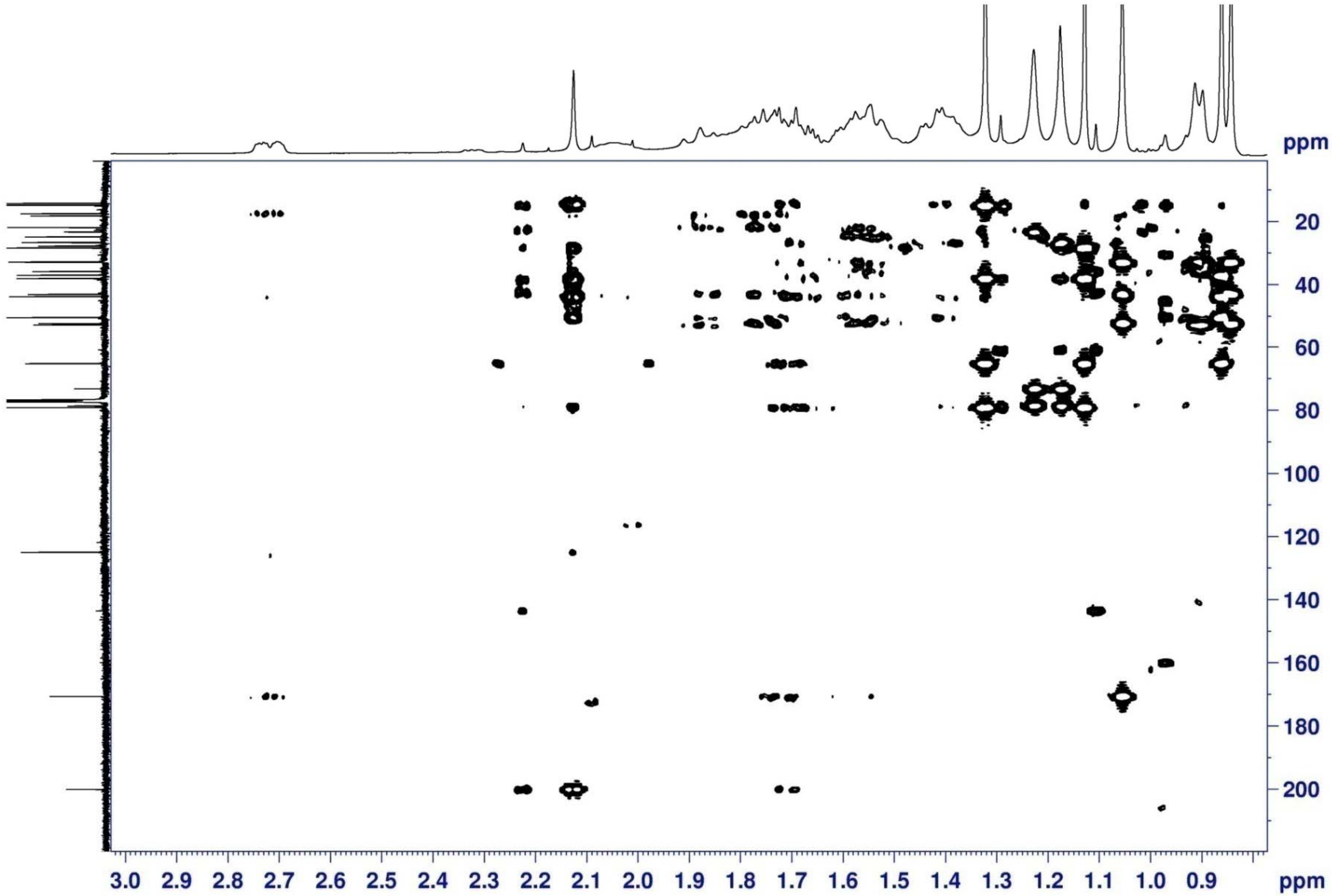

HMBC (400 MHz) spectrum of compound **6** in $CDCl_3$

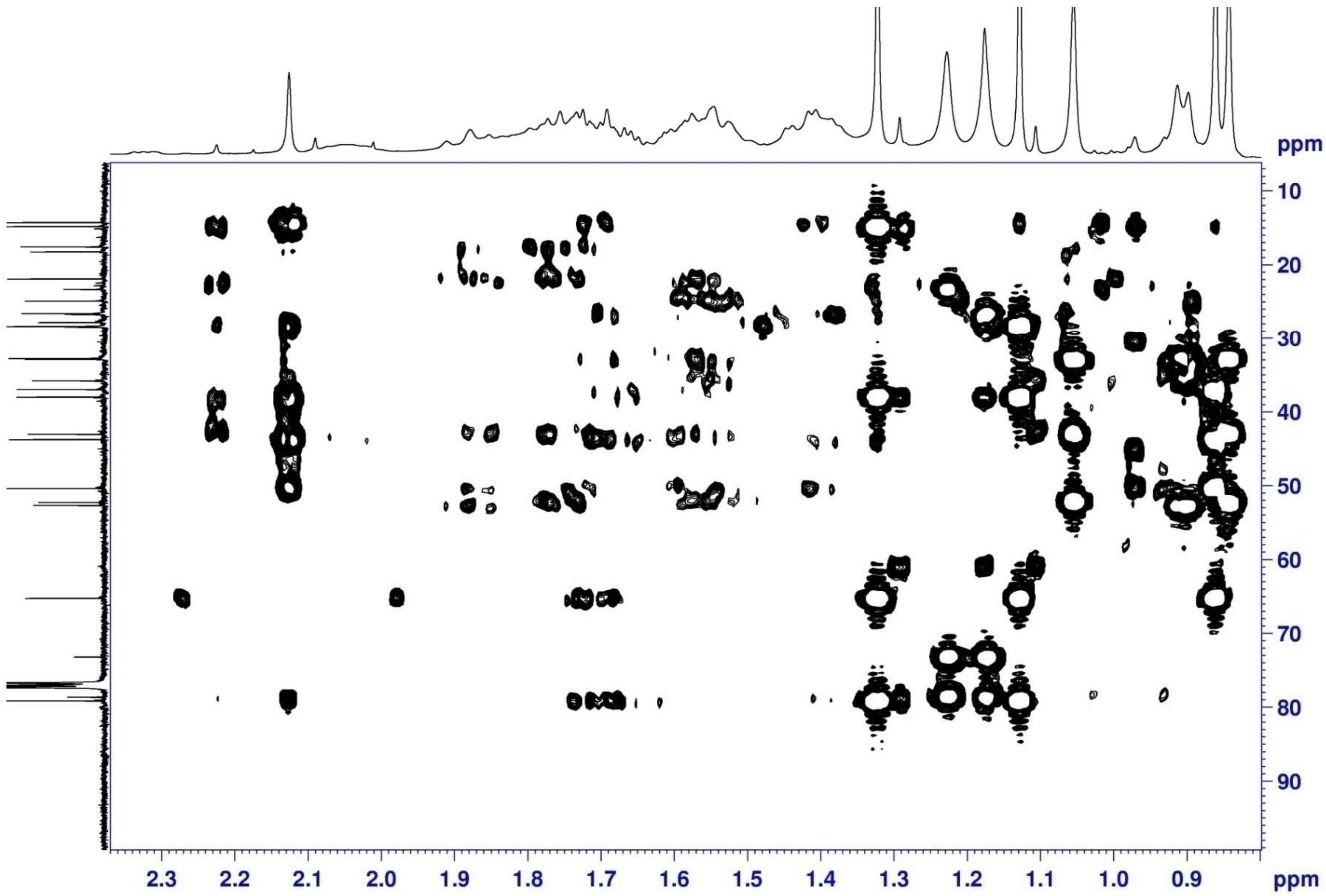

HMBC (400 MHz) spectrum of compound **6** in $CDCl_3$

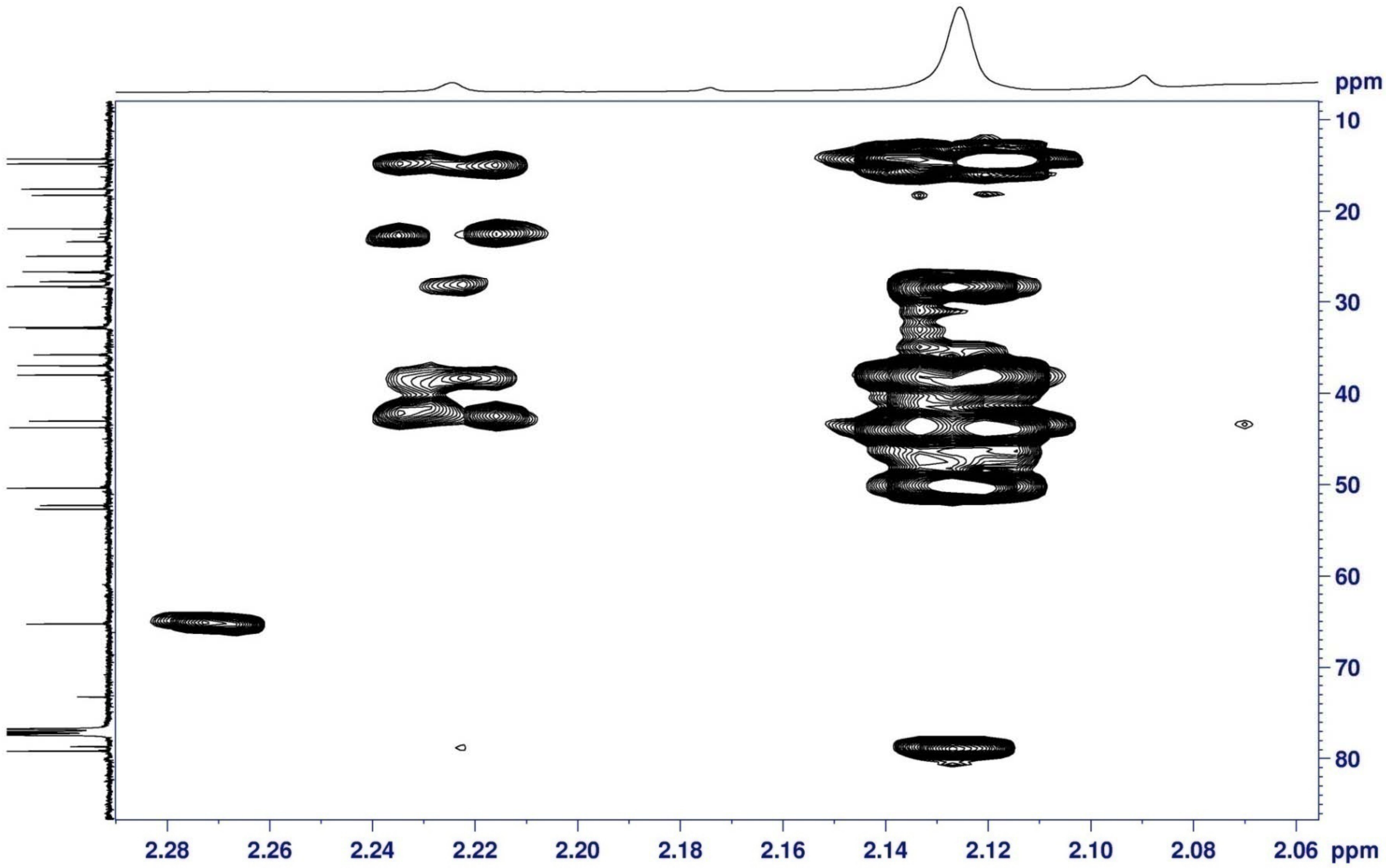

HMBC (400 MHz) spectrum of compound **6** in $CDCl_3$

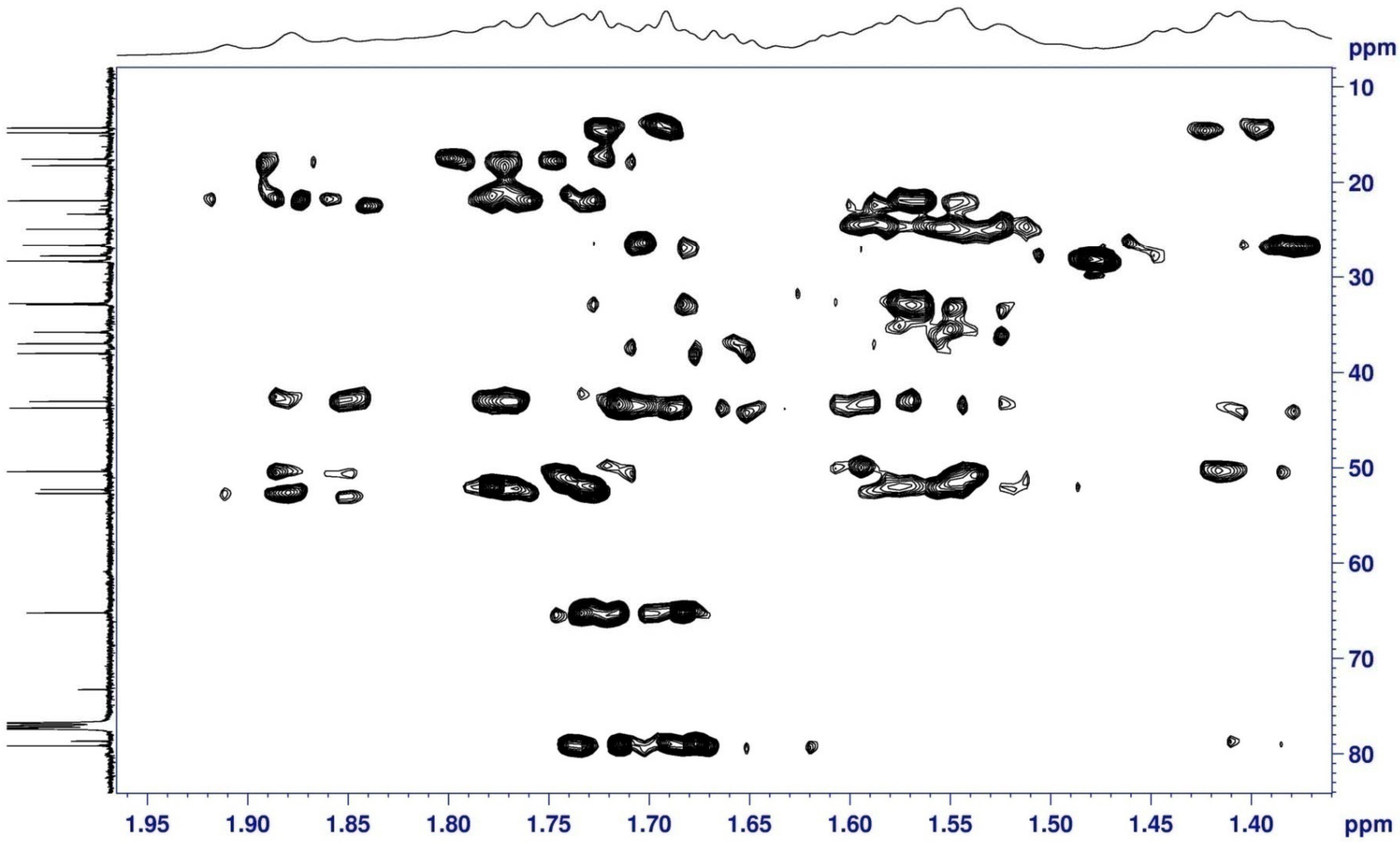

HMBC (400 MHz) spectrum of compound **6** in $CDCl_3$

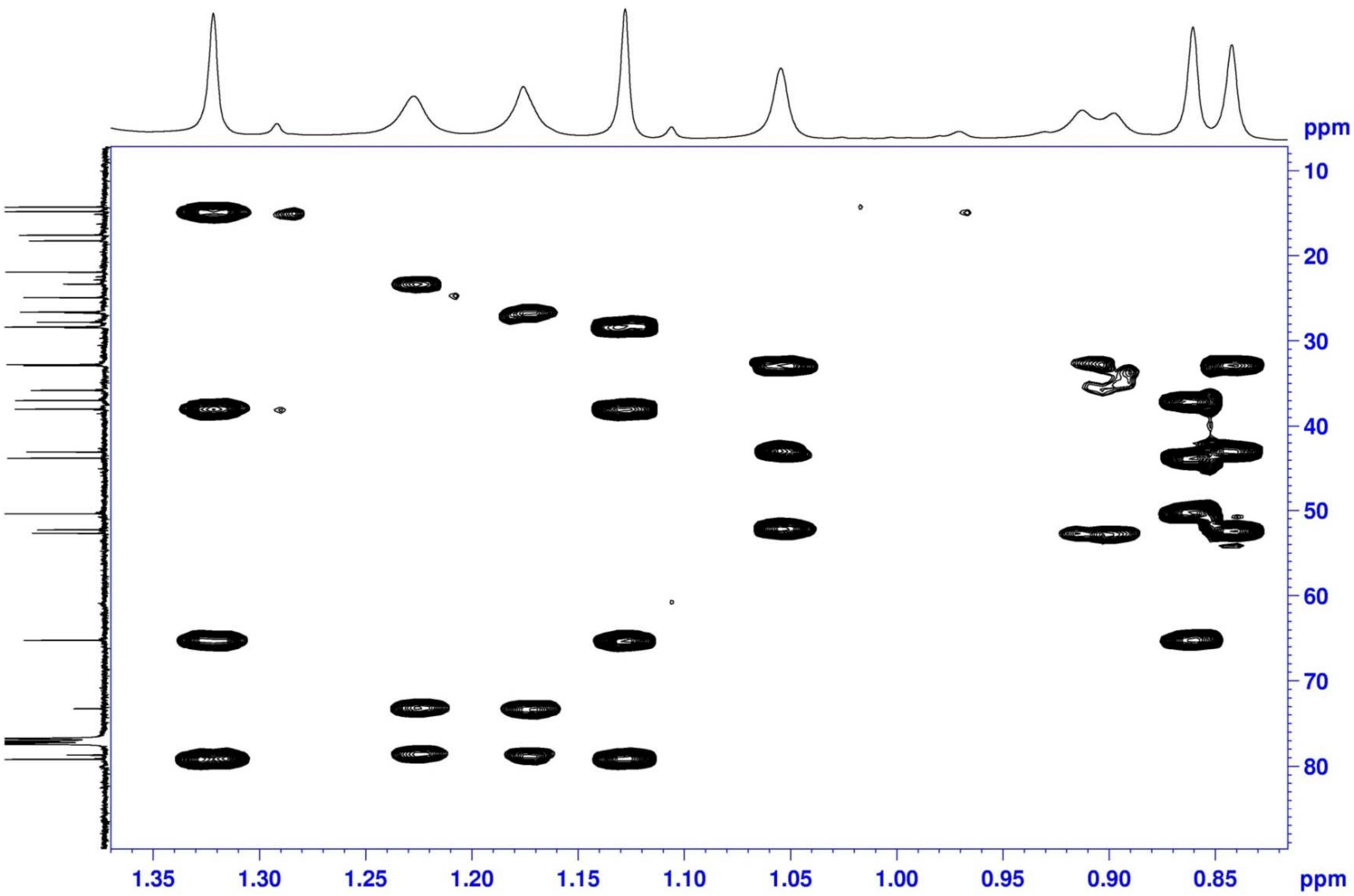

NOESY (400 MHz) spectrum of compound **6** in $CDCl_3$

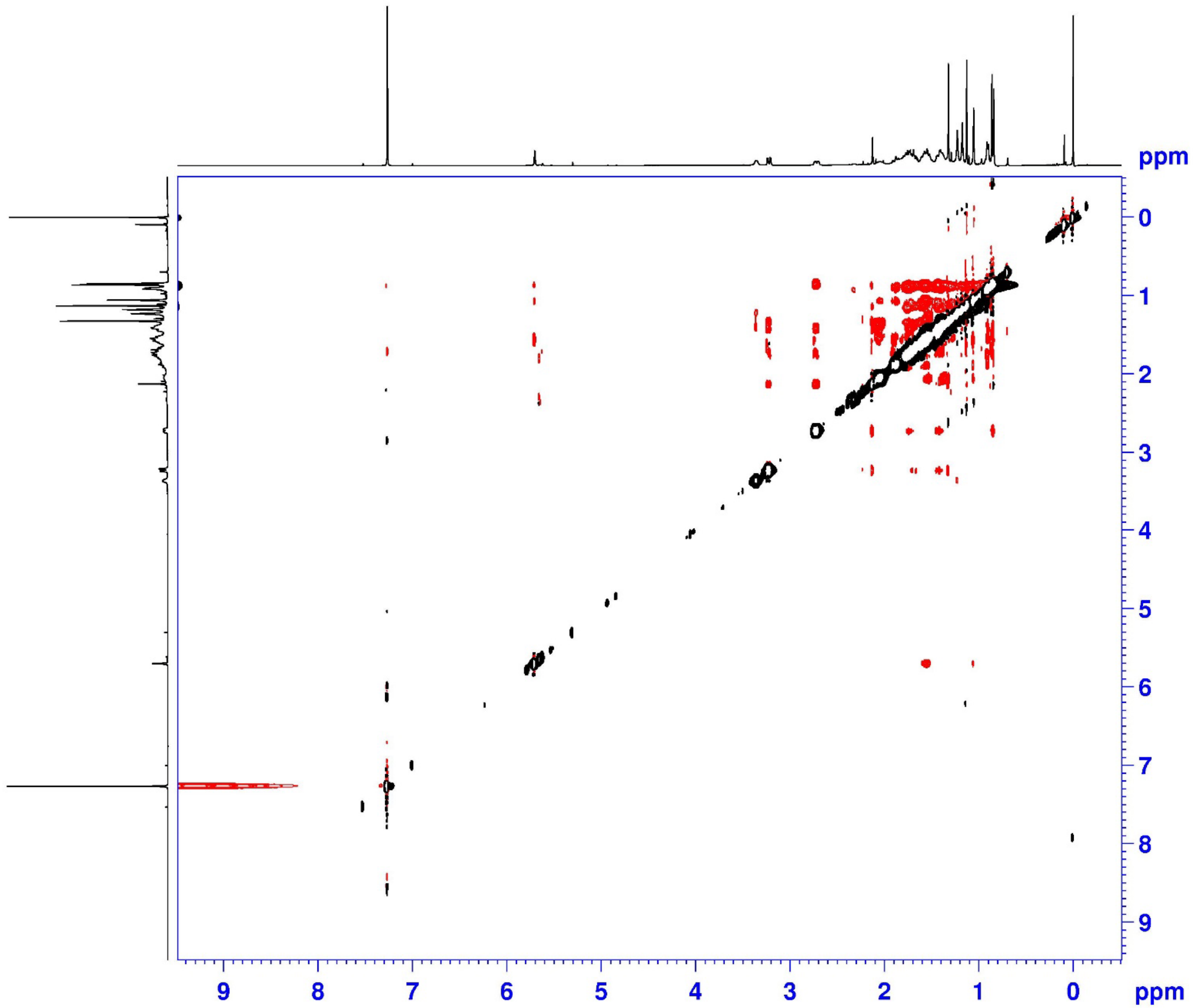

NAME EA-16
EXPNO 7
PROCNO 1
Date_ 20160806
Time 10.44
INSTRUM spect
PROBHD 5 mm CPPBBO BB
PULPROG noesygpphpp
TD 2048
SOLVENT CDCl3
NS 16
DS 32
SWH 4000.000 Hz
FIDRES 1.953125 Hz
AQ 0.2560500 se
RG 208.5
DW 125.000 us
DE 10.00 us
TE 297.0 K
D0 0.00011036 se
D1 1.99385595 se
D8 0.30000001 se
D11 0.03000000 se
D12 0.00002000 se
D16 0.00020000 se
IN0 0.00025000 se

======== CHANNEL f1 ======
SFO1 400.1318006 MH
NUC1 1H
P1 11.50 us
P2 23.00 us
P17 2500.00 us
ND0 1
TD 256
SFO1 400.1318 MH
FIDRES 15.625000 Hz
SW 9.997 pp
FnMODE States-TPPI
SI 1024
SF 400.1300058 MH
WDW QSINE
SSB 2
LB 0.00 Hz
GB 0
PC 1.00
SI 1024
MC2 States-TPPI
SF 400.1300069 MH
WDW OSINE

NOESY (400 MHz) spectrum of compound **6** in $CDCl_3$

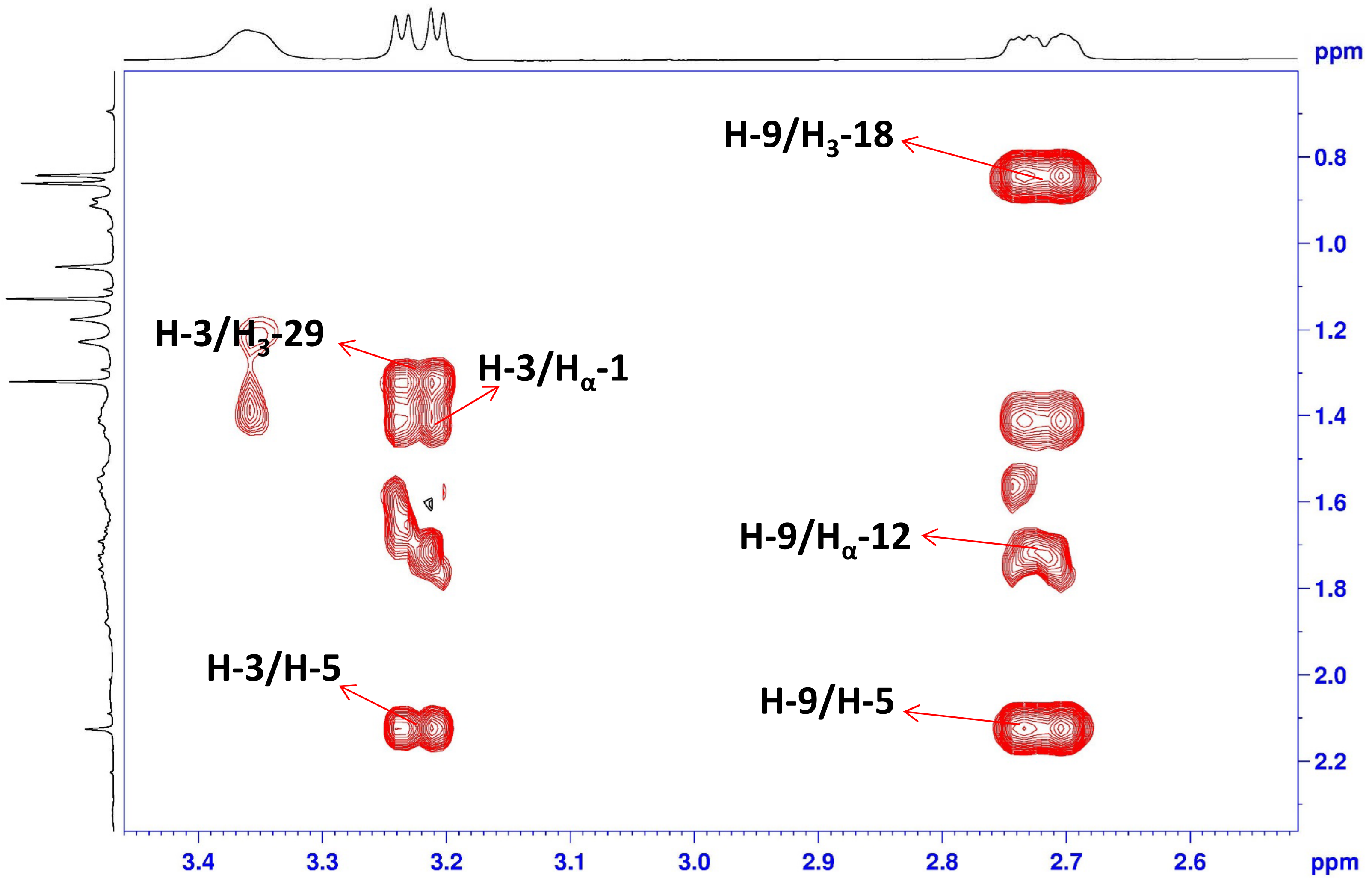

NOESY (400 MHz) spectrum of compound **6** in $CDCl_3$

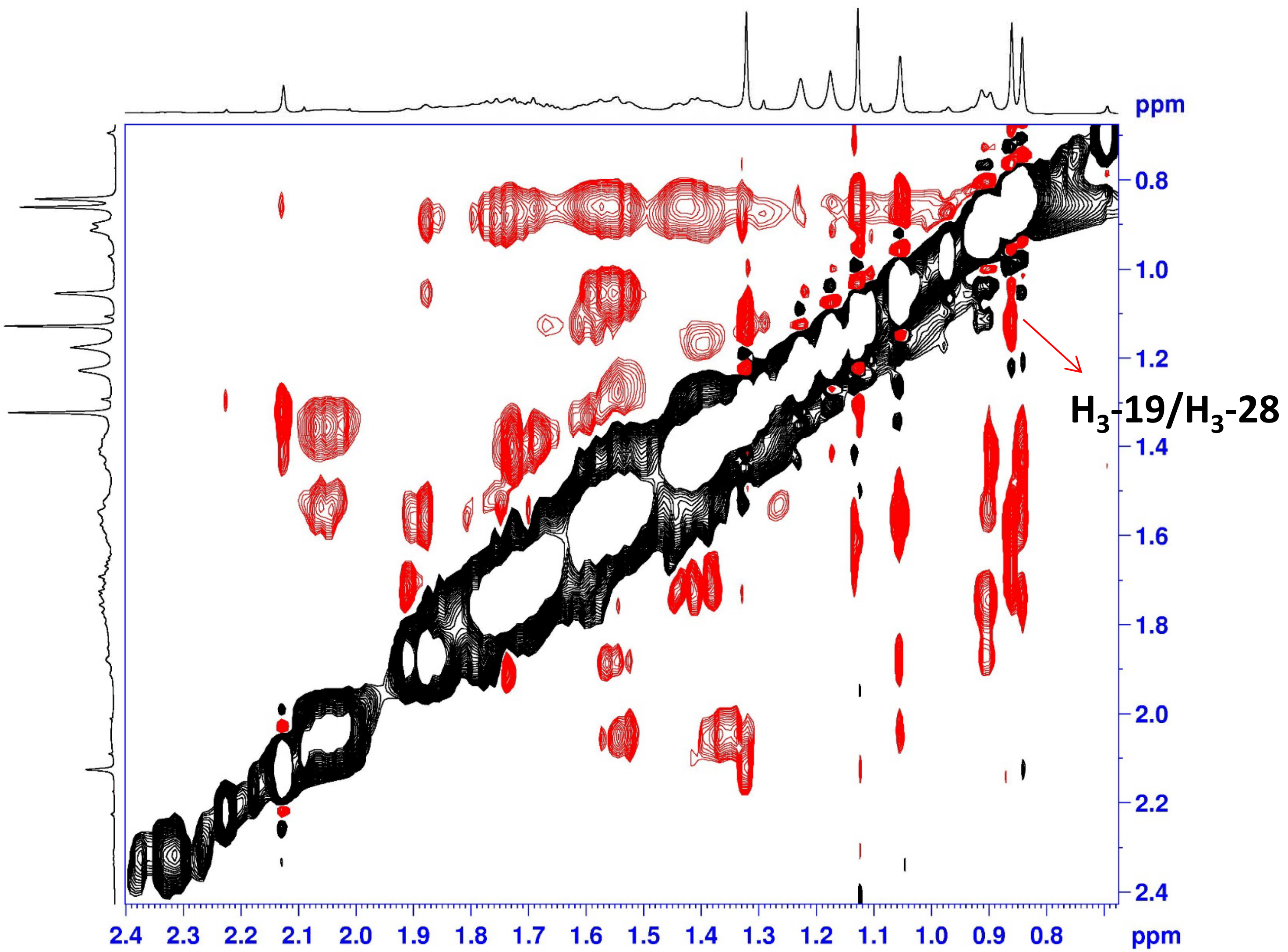

NOESY (400 MHz) spectrum of compound **6** in $CDCl_3$

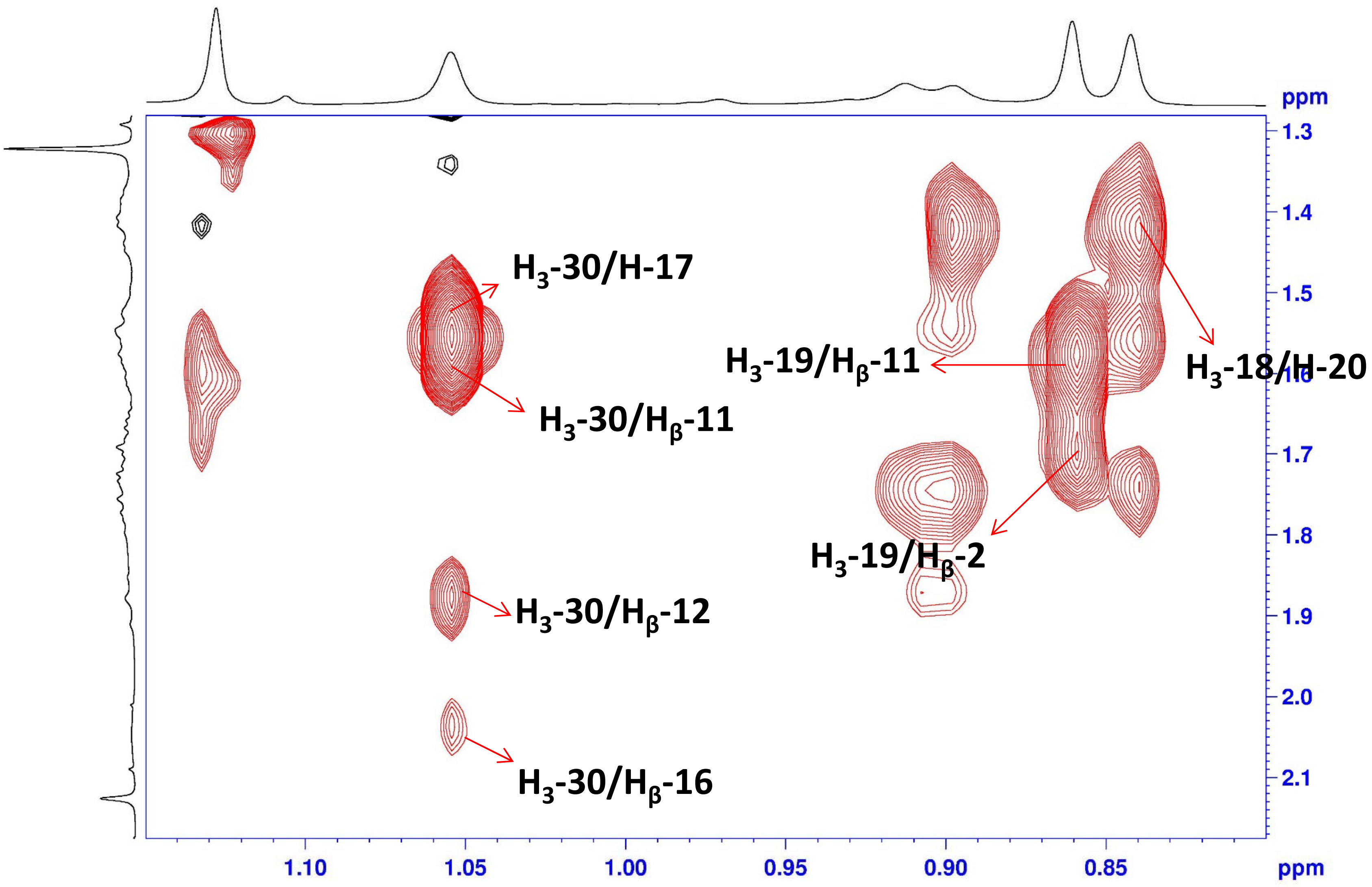

HR-ESIMS for compound **7**

Generic Display Report

Analysis Info		Acquisition Date	12/21/2016 4:55:09 PM
Analysis Name	D:\Data\MS\data\201612\liwanshan_EA-17_pos_26_01_2826.d		
Method	LC_Direct Infusion_pos_70-500mz.m	Operator	SCSIO
Sample Name	liwanshan_EA-17_pos	Instrument	maXis
Comment			

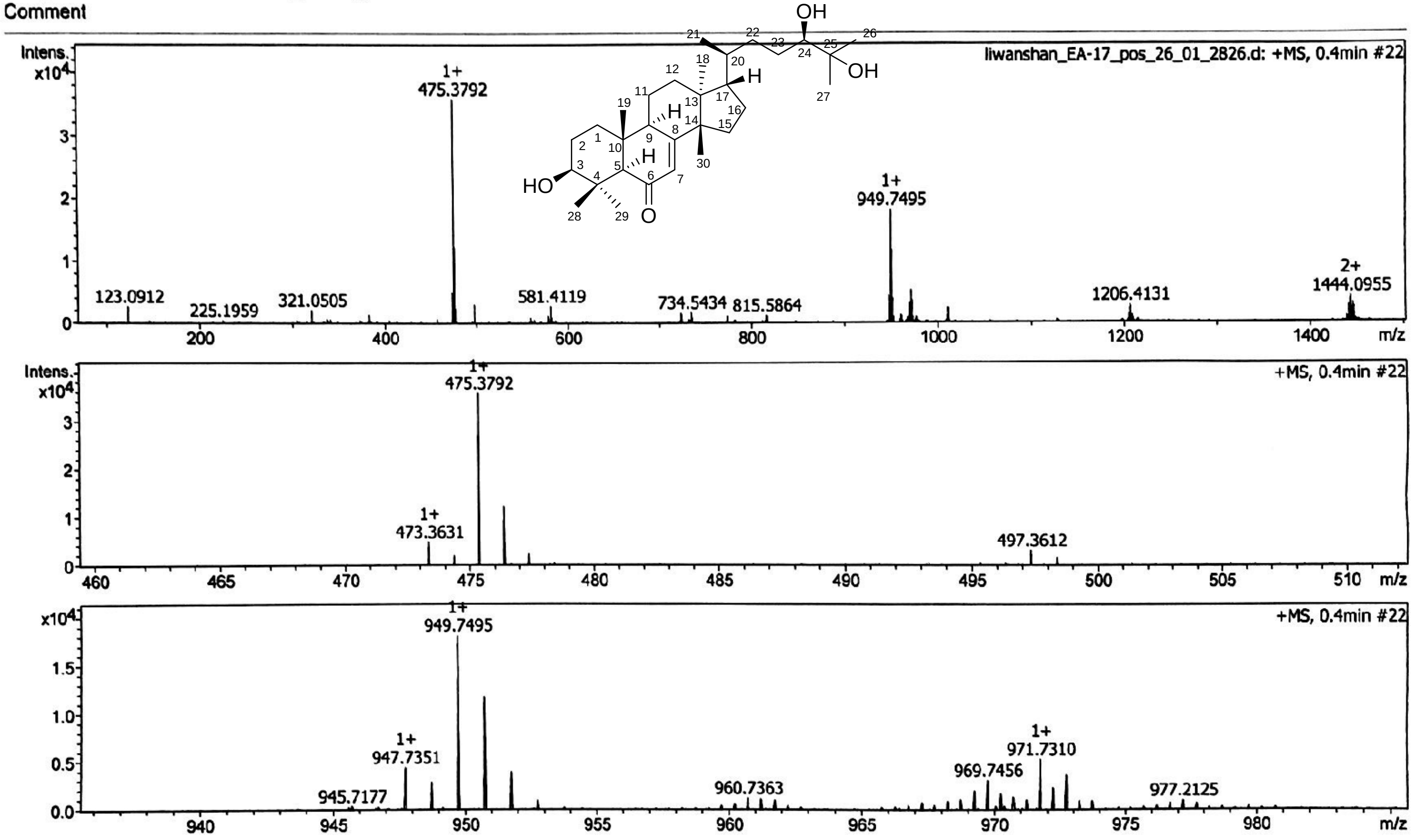

Bruker Compass DataAnalysis 4.1 printed: 12/21/2016 4:59:50 PM by: SCSIO Page 1 of 1

HR-ESIMS for compound **7**

Mass Spectrum SmartFormula Report

Analysis Info

Analysis Name	D:\Data\MS\data\201612\liwanshan_EA-17_pos_26_01_2826.d	Acquisition Date	12/21/2016 4:55:09 PM
Method	LC_Direct Infusion_pos_70-500mz.m	Operator	SCSIO
Sample Name	liwanshan_EA-17_pos	Instrument	maXis 255552.00029
Comment			

Acquisition Parameter

Source Type	ESI	Ion Polarity	Positive	Set Nebulizer	0.4 Bar
Focus	Active	Set Capillary	4500 V	Set Dry Heater	180 °C
Scan Begin	70 m/z	Set End Plate Offset	-500 V	Set Dry Gas	4.0 l/min
Scan End	1500 m/z	Set Charging Voltage	0 V	Set Divert Valve	Waste
		Set Corona	0 nA	Set APCI Heater	0 °C

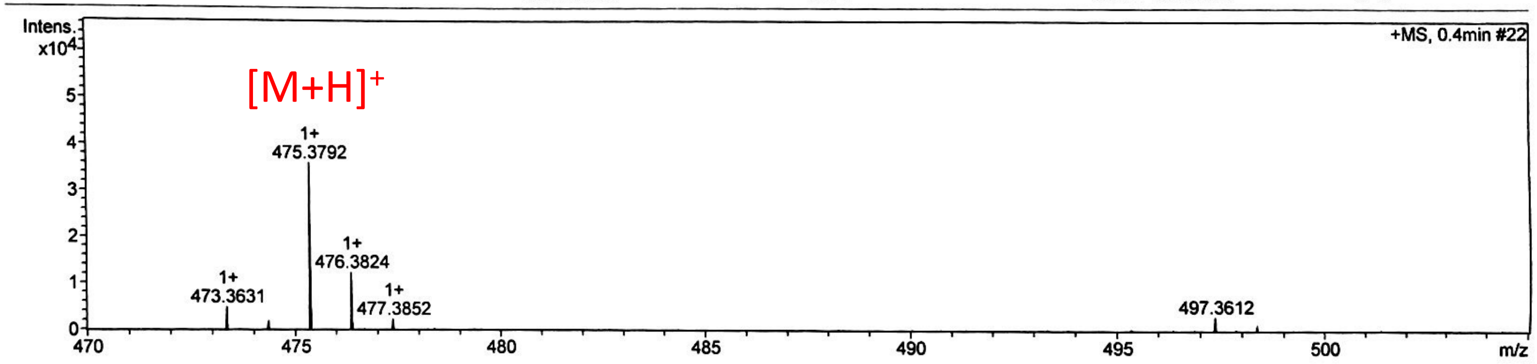

Meas. m/z	#	Ion Formula	Score	m/z	err [ppm]	err [mDa]	mSigma	rdb	e⁻ Conf	N-Rule
475.379226	1	C30H51O4	100.00	475.378187	2.2	1.0	6.3	5.5	even	ok
497.361232	1	C30H50NaO4	100.00	497.360131	-2.2	-1.1	20.8	5.5	even	ok
949.749486	1	C60H101O8	100.00	949.749097	0.4	0.4	9.2	10.5	even	ok
971.730951	1	C60H100NaO8	100.00	971.731041	-0.1	-0.1	25.7	10.5	even	ok

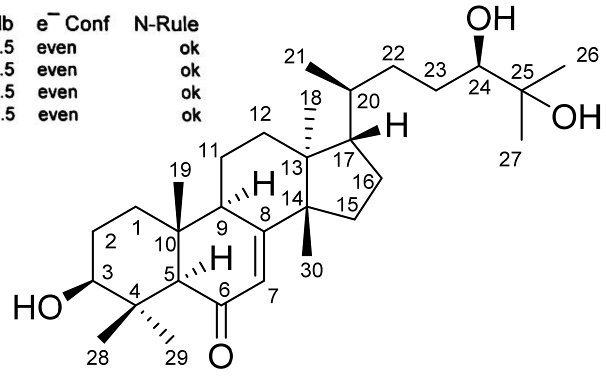

^{1}H NMR (400 MHz) spectrum of compound **7** in $CDCl_3$

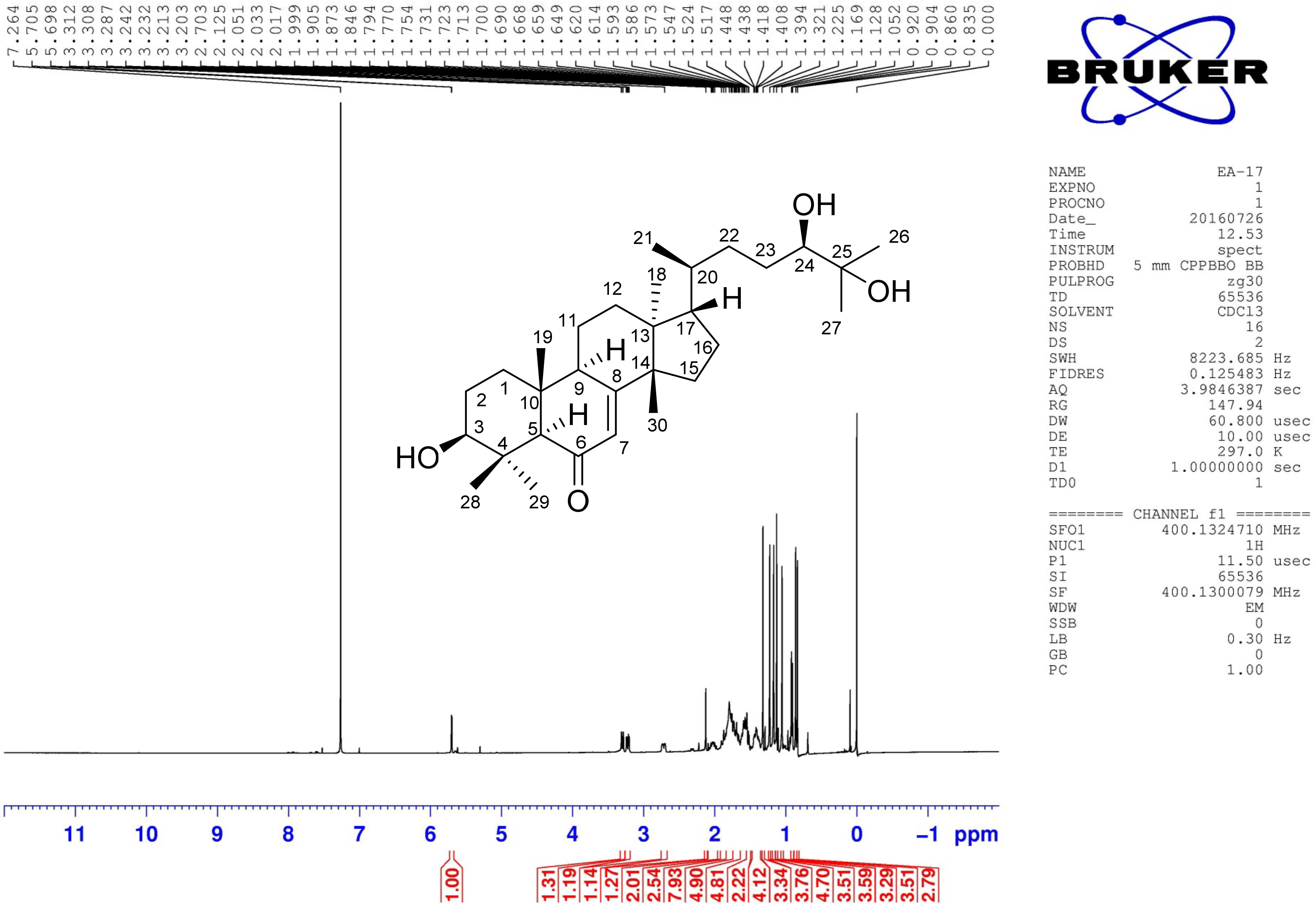

^{1}H NMR (400 MHz) spectrum of compound **7** in $CDCl_3$

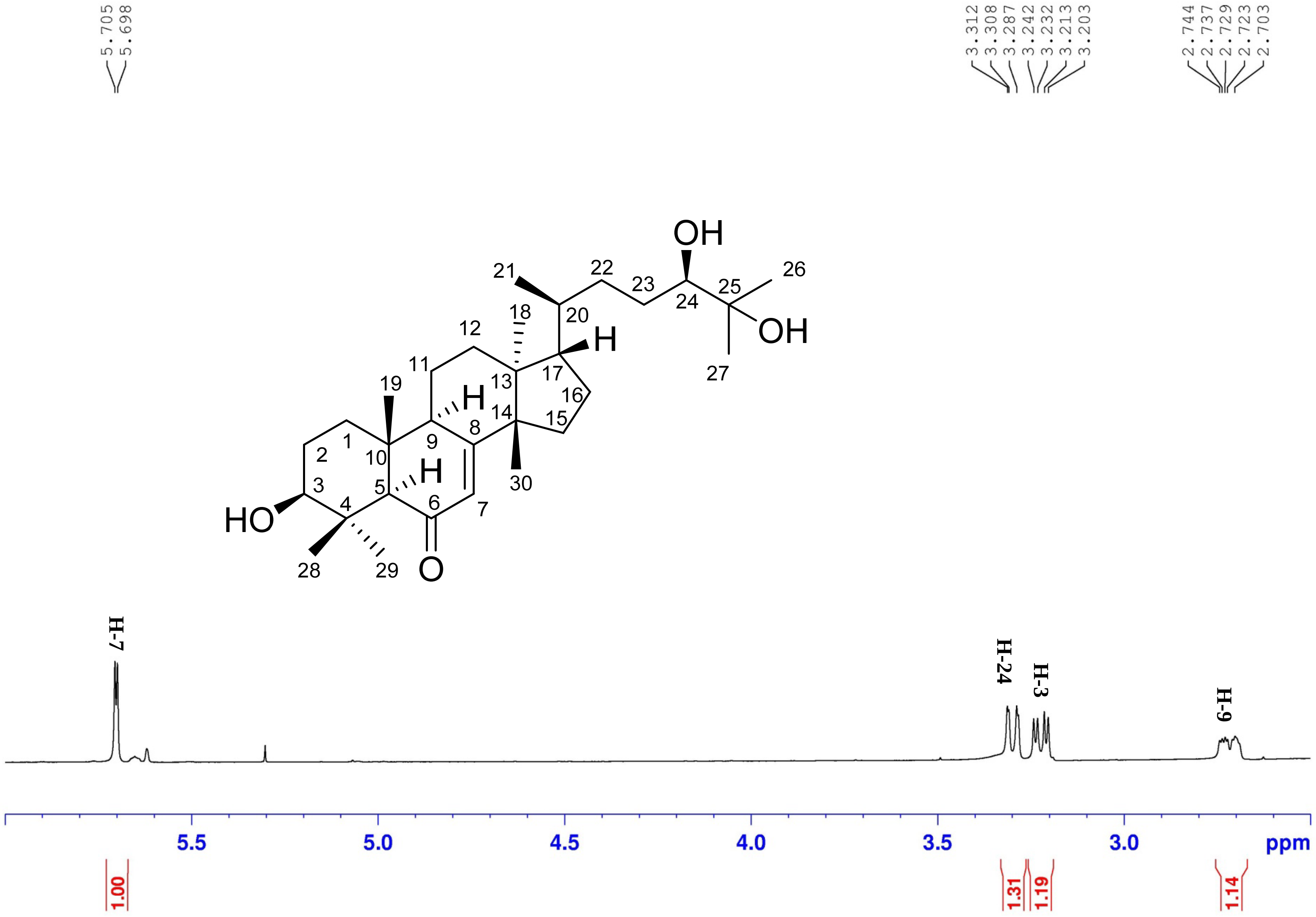

^{1}H NMR (400 MHz) spectrum of compound **7** in $CDCl_3$

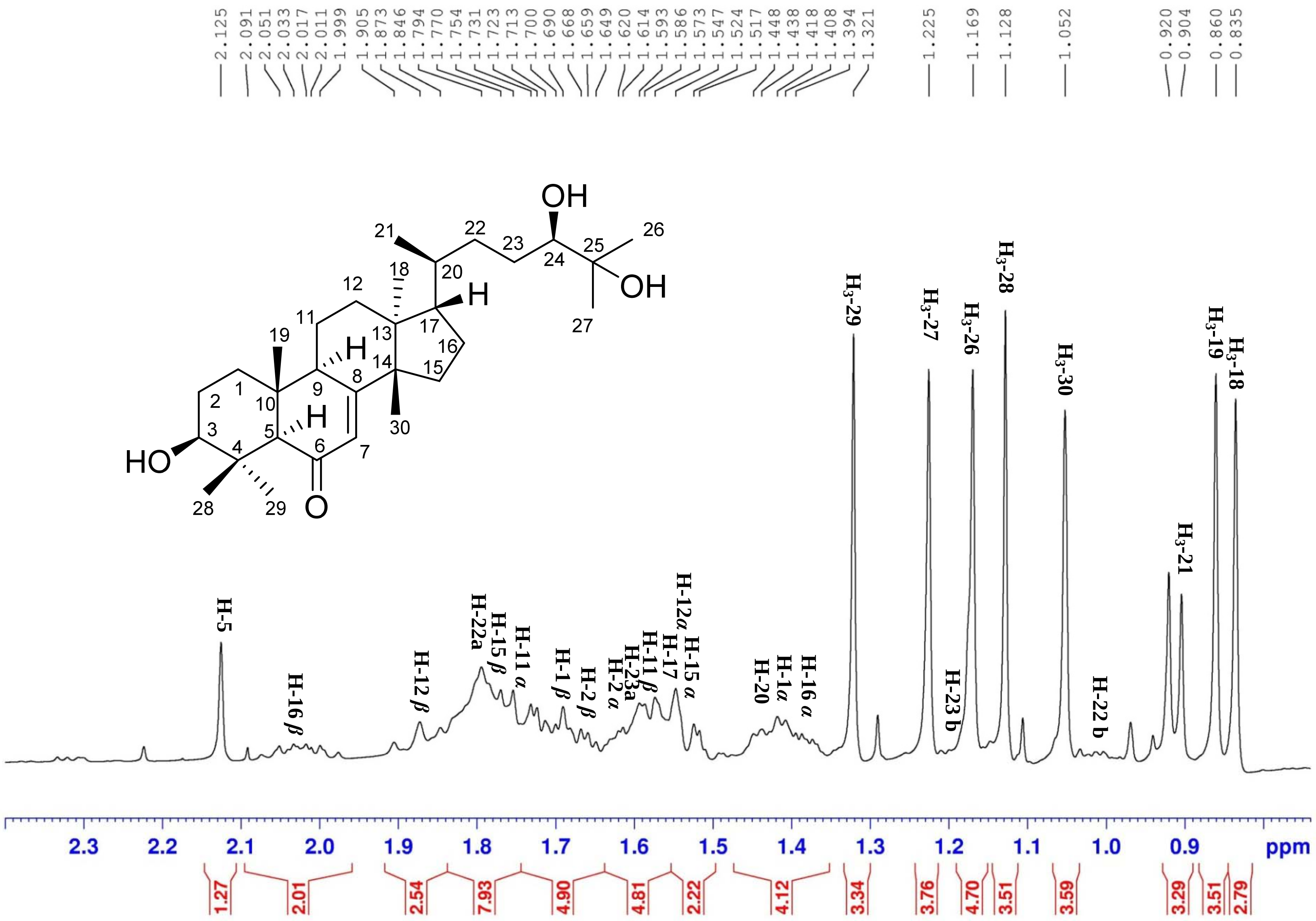

^{13}C NMR (100 MHz) spectrum of compound **7** in $CDCl_3$

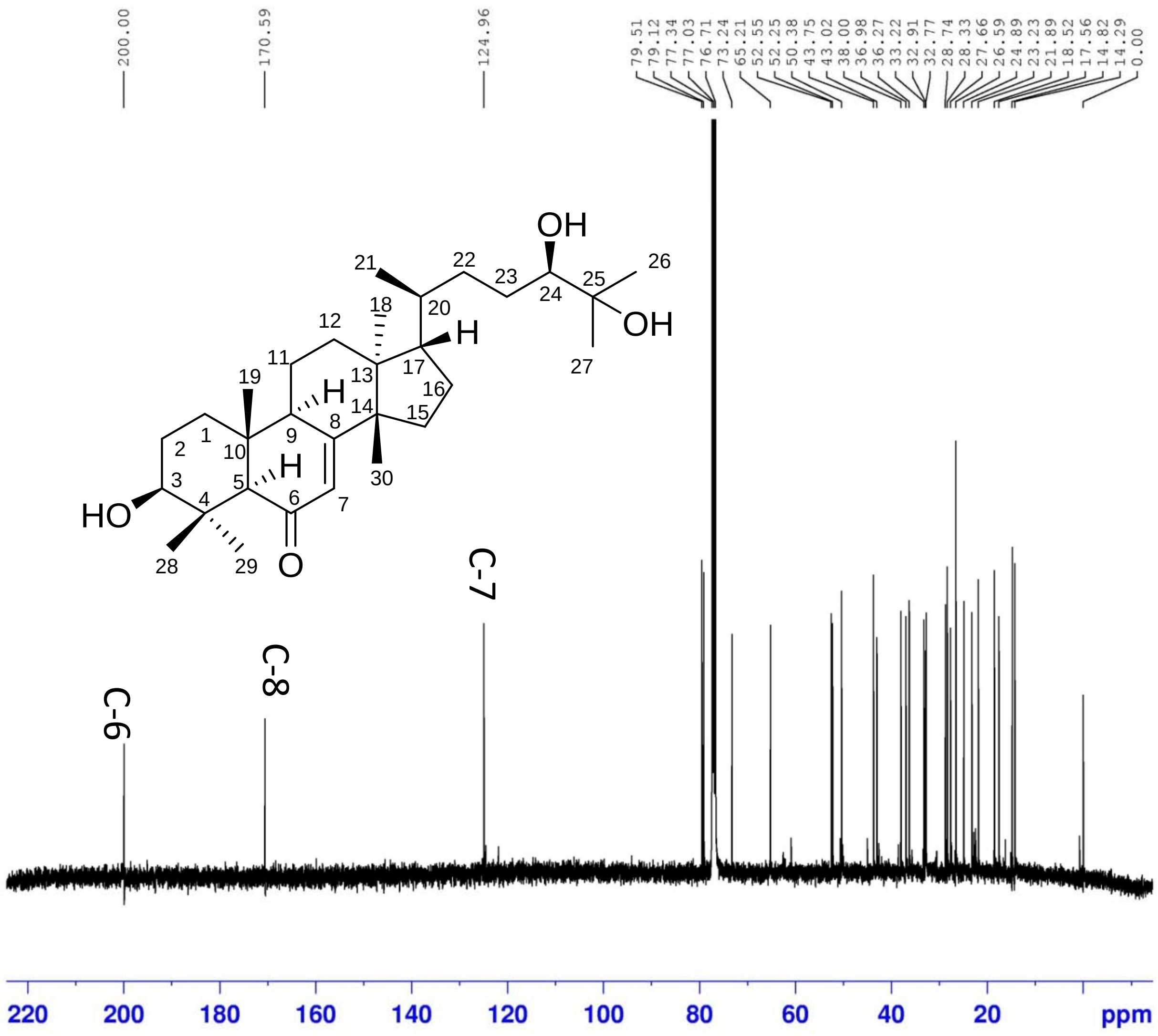

NAME	EA-17
EXPNO	102
PROCNO	1
Date_	20160727
Time	0.54
INSTRUM	spect
PROBHD	5 mm CPPBBO BB
PULPROG	zgpg30
TD	65536
SOLVENT	CDCl3
NS	1024
DS	4
SWH	24038.461 Hz
FIDRES	0.366798 Hz
AQ	1.3631988 sec
RG	102.3
DW	20.800 usec
DE	18.00 usec
TE	297.0 K
D1	2.00000000 sec
D11	0.03000000 sec
TD0	1
======== CHANNEL f1 ========	
SFO1	100.6233324 MHz
NUC1	13C
P1	10.00 usec
SI	32768
SF	100.6127690 MHz
WDW	EM
SSB	0
LB	1.00 Hz
GB	0
PC	1.40

^{13}C NMR (100 MHz) spectrum of compound **7** in $CDCl_3$

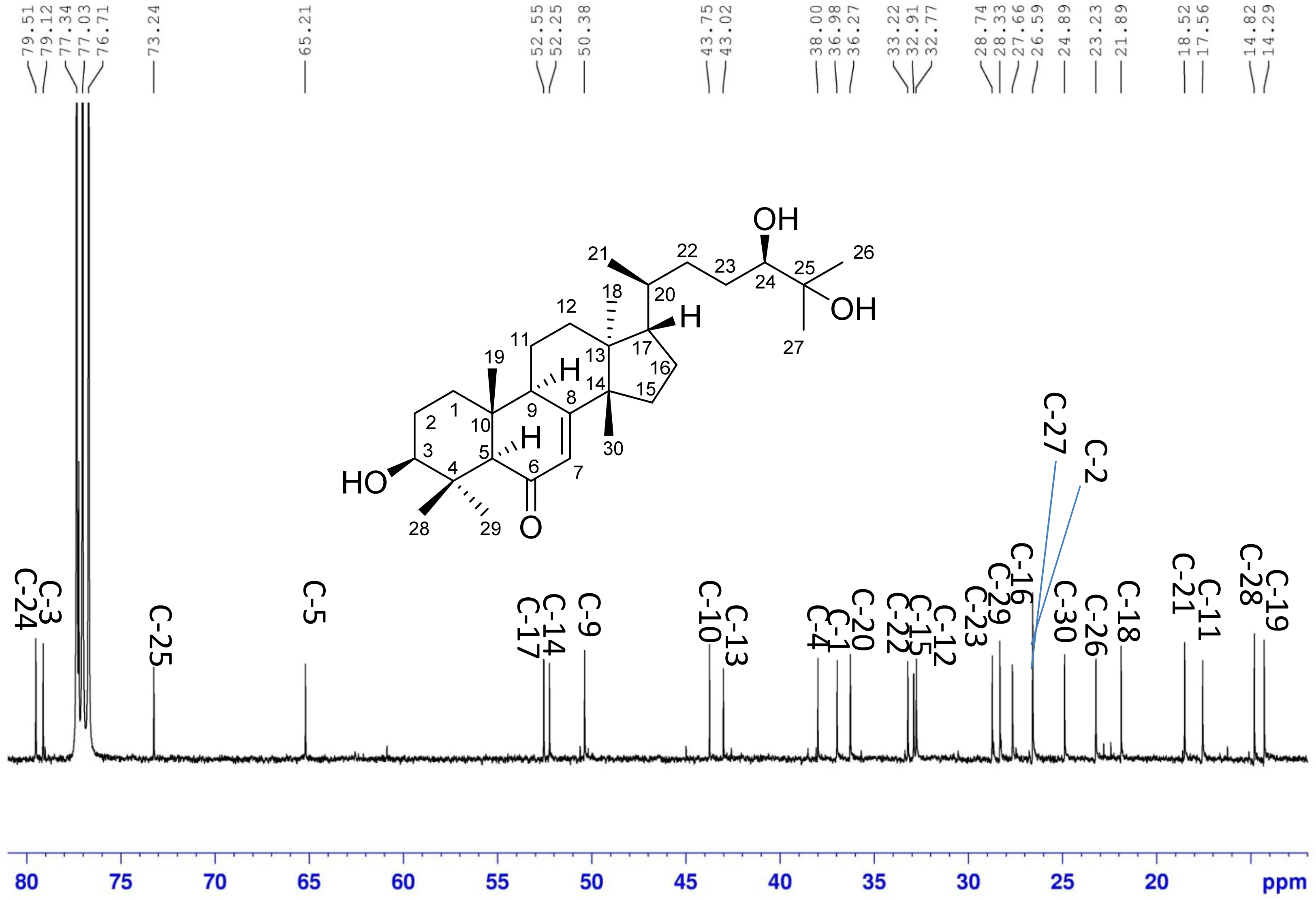

DEPT135 (100 MHz) spectrum of compound **7** in $CDCl_3$

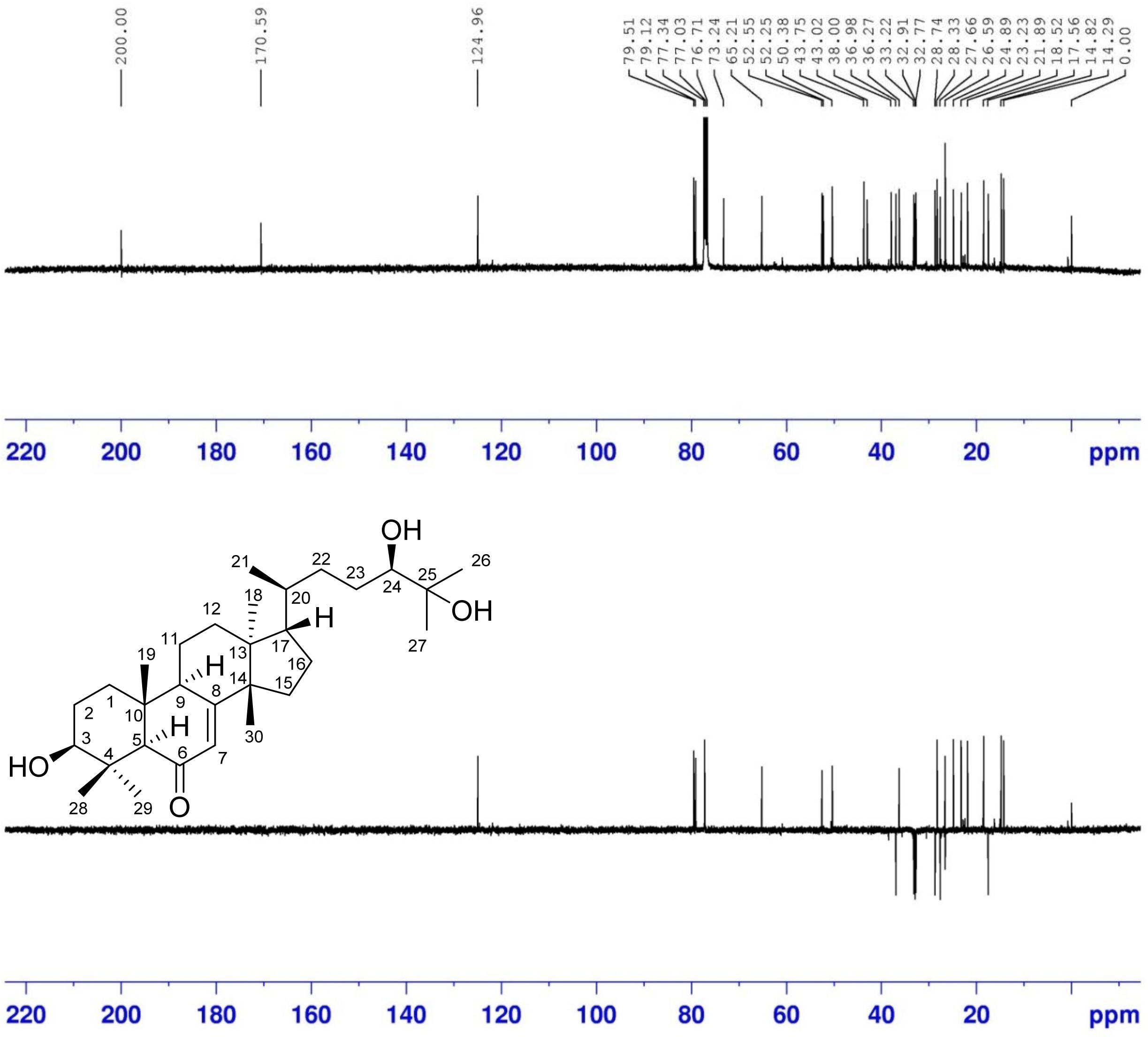

NAME	EA-17	
EXPNO	103	
PROCNO	1	
Date_	20160727	
Time	1.12	
INSTRUM	spect	
PROBHD	5 mm CPPBBO BB	
PULPROG	deptsp135	
TD	65536	
SOLVENT	CDCl3	
NS	300	
DS	4	
SWH	24038.461	Hz
FIDRES	0.366798	Hz
AQ	1.3631988	sec
RG	130.26	
DW	20.800	usec
DE	18.00	usec
TE	297.0	K
CNST2	145.0000000	
D1	2.00000000	sec
D2	0.00344828	sec
D12	0.00002000	sec
TD0	1	
======== CHANNEL f1 ========		
SFO1	100.6233324	MHz
NUC1	13C	
P1	10.00	usec
P13	2000.00	usec
SI	32768	
SF	100.6127690	MHz
WDW	EM	
SSB	0	
LB	1.00	Hz
GB	0	
PC	1.40	

DEPT135 (100 MHz) spectrum of compound **7** in $CDCl_3$

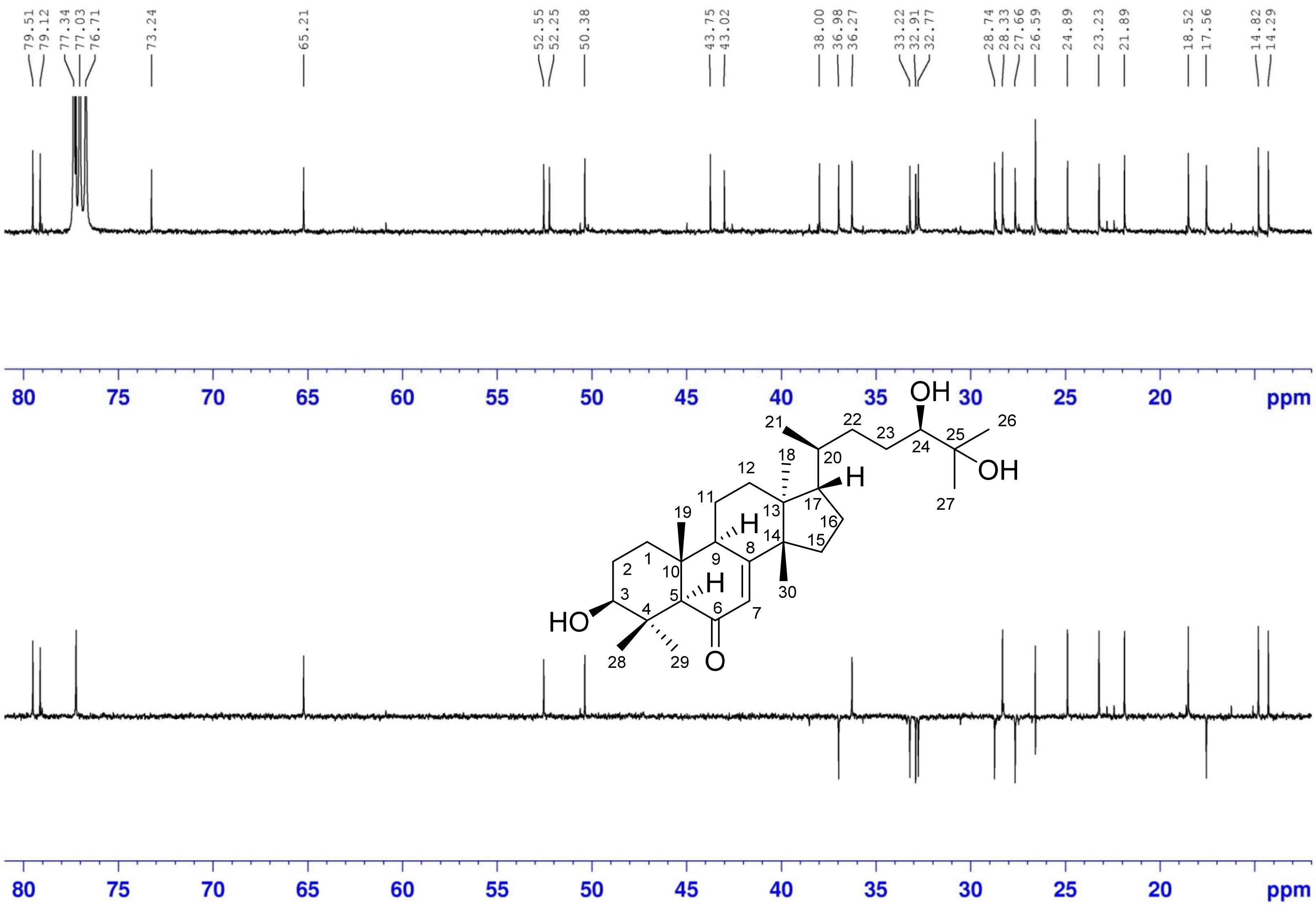

^{1}H-^{1}H COSY (400 MHz) spectrum of compound **7** in $CDCl_3$

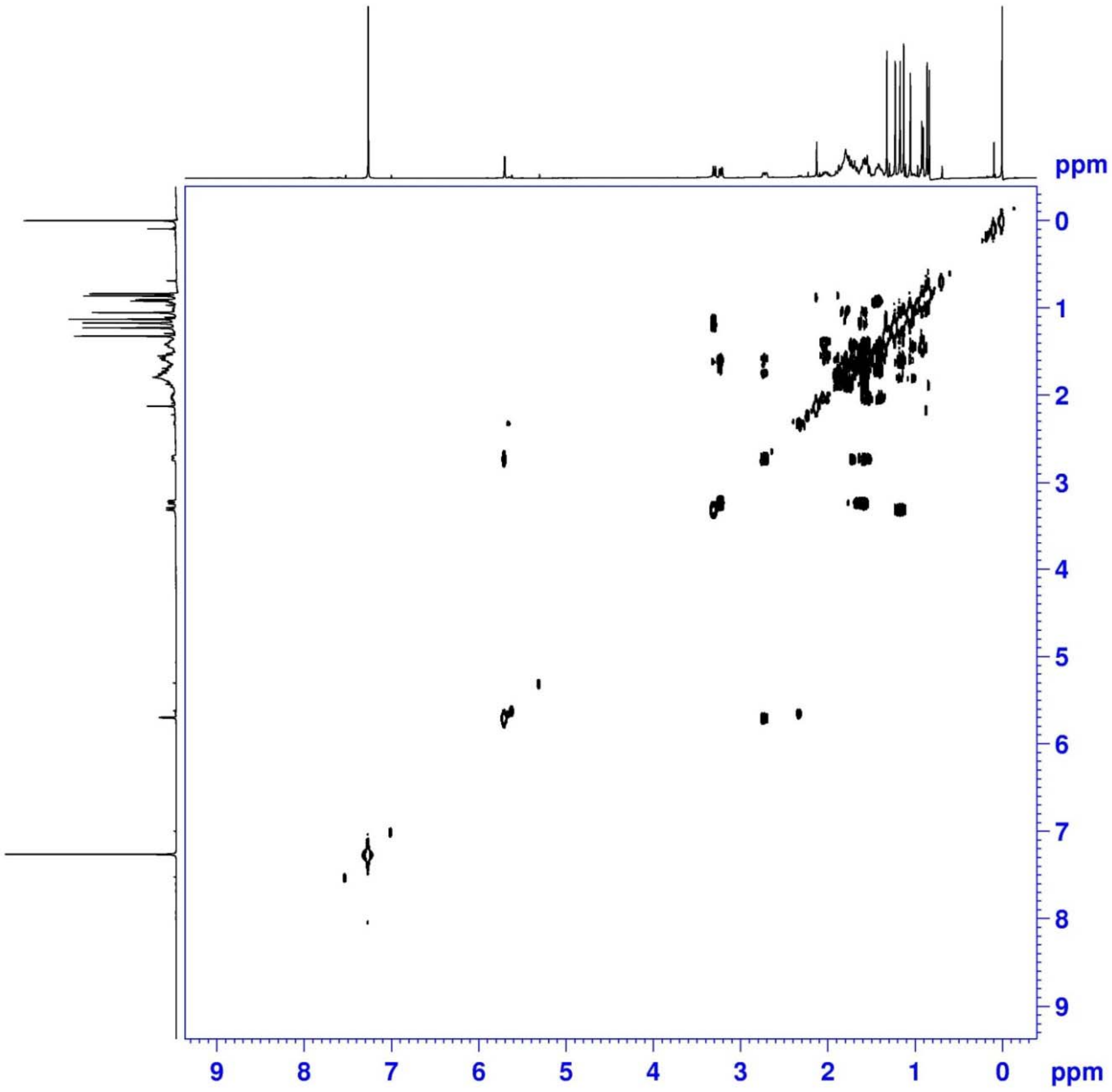


```
NAME               EA-17
EXPNO                104
PROCNO                 1
Date_           20160805
Time               22.10
INSTRUM            spect
PROBHD   5 mm CPPBBO BB
PULPROG       cosygpppqf
TD                  2048
SOLVENT            CDCl3
NS                     8
DS                     8
SWH             3906.250 Hz
FIDRES          1.907349 Hz
AQ             0.2621940 sec
RG                 208.5
DW               128.000 usec
DE                 10.00 usec
TE                 297.0 K
D0            0.00000300 sec
D1            1.89678097 sec
D11           0.03000000 sec
D12           0.00002000 sec
D13           0.00000400 sec
D16           0.00020000 sec
IN0           0.00025600 sec

======== CHANNEL f1 ========
SFO1         400.1318006 MHz
NUC1                  1H
P0                 11.50 usec
P1                 11.50 usec
P17              2500.00 usec
ND0                    1
TD                   128
SFO1            400.1318 MHz
FIDRES         30.517578 Hz
SW                 9.762 ppm
FnMODE                QF
SI                  1024
SF           400.1300058 MHz
WDW                QSINE
SSB                    0
LB                  0.00 Hz
GB                     0
PC                  1.40
SI                  1024
MC2                   QF
SF           400.1300047 MHz
WDW                QSINE
SSB                    0
[illegible]
```

^{1}H-^{1}H COSY (400 MHz) spectrum of compound **7** in $CDCl_3$

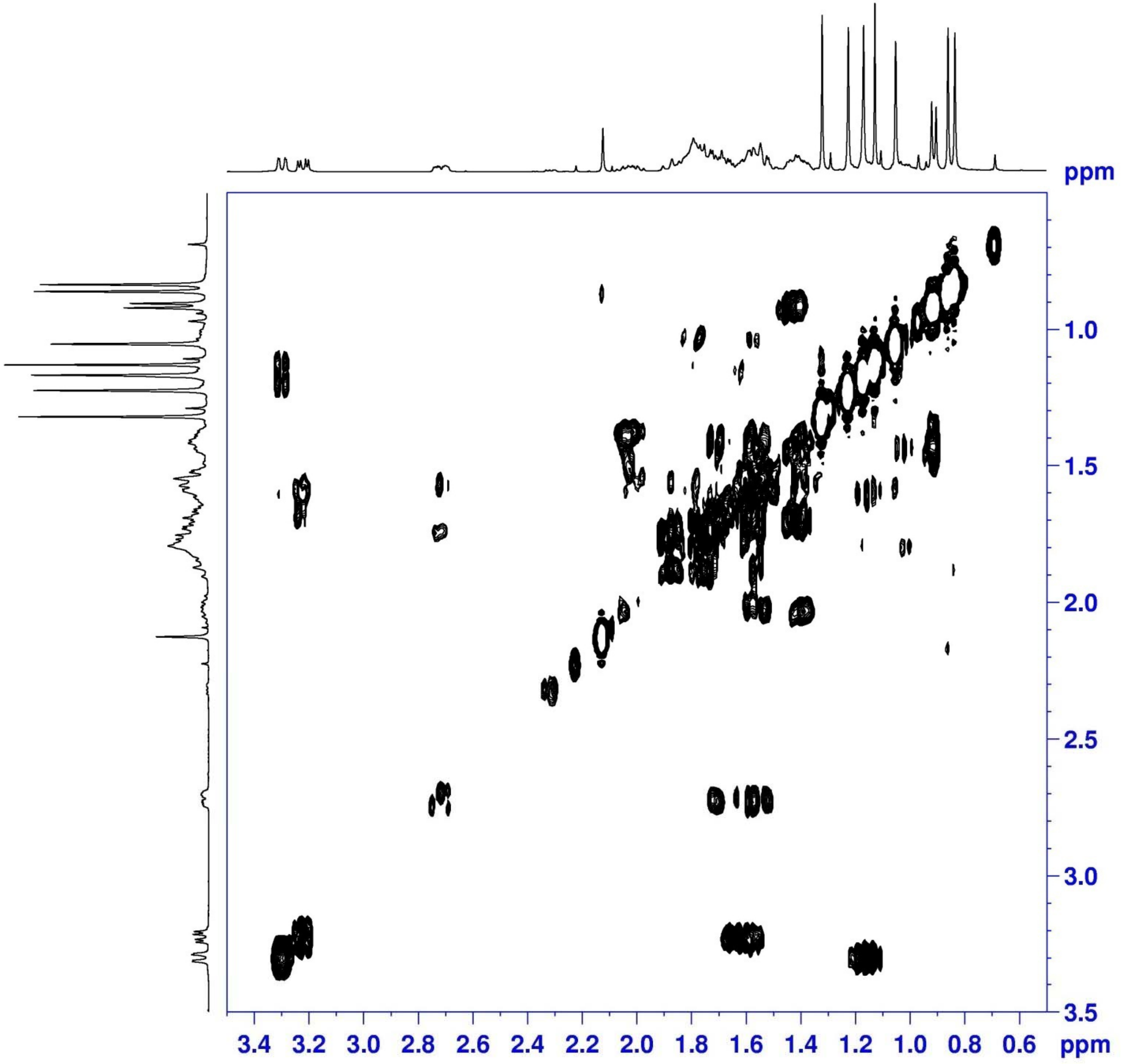

^1H-^1H COSY (400 MHz) spectrum of compound **7** in $CDCl_3$

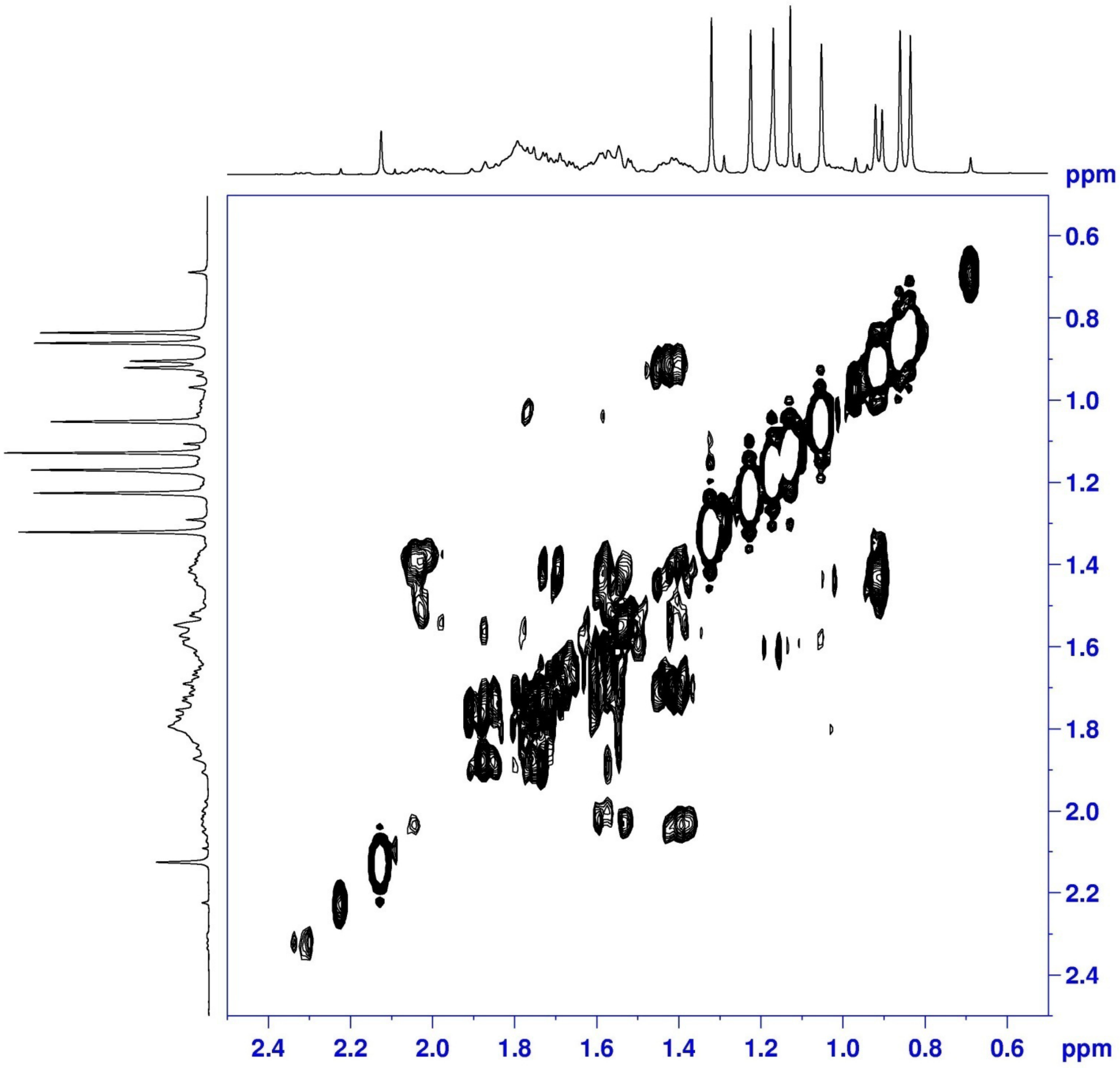

HSQC (400 MHz) spectrum of compound **7** in $CDCl_3$

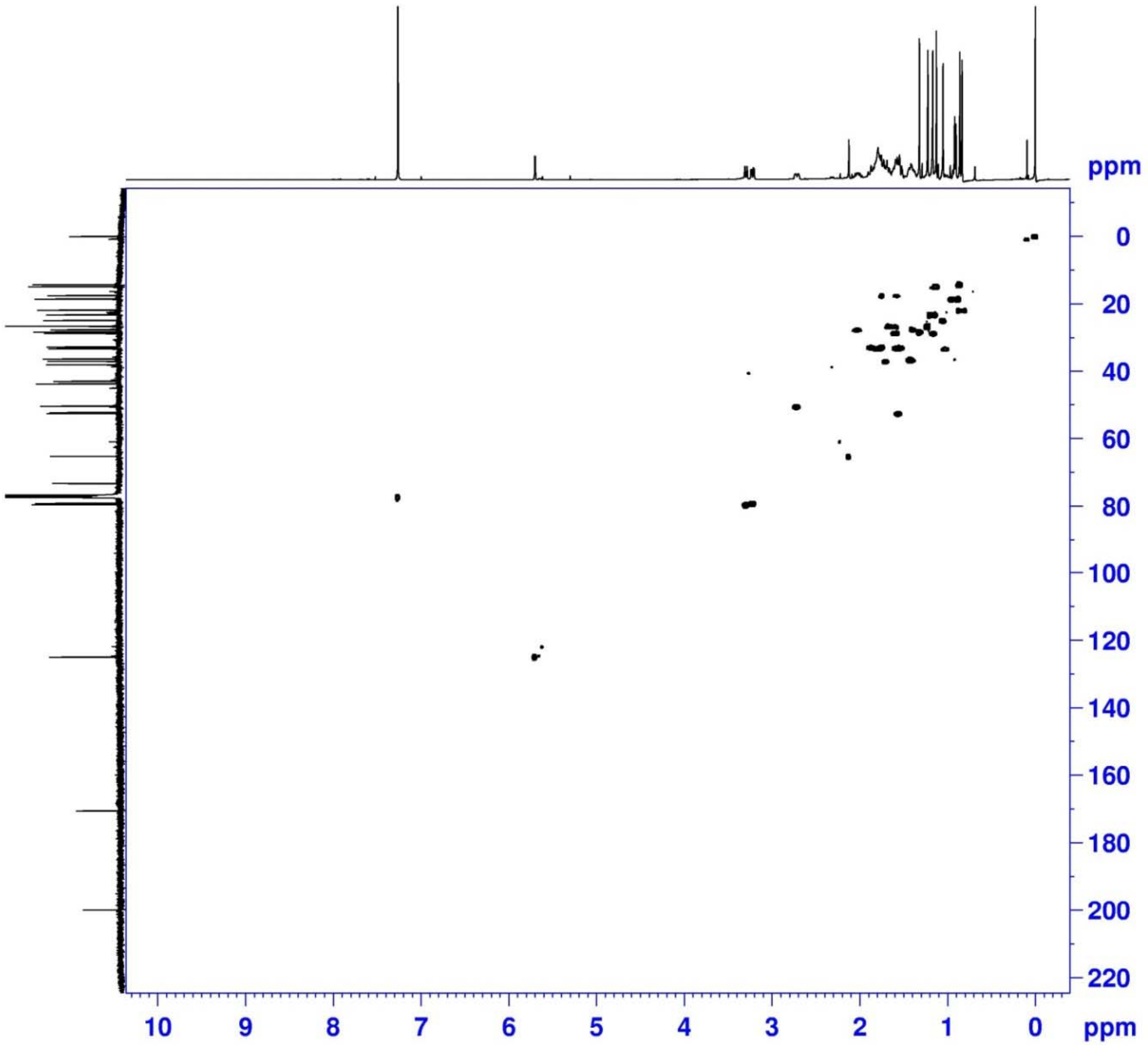


```
NAME                EA-17
EXPNO                 105
PROCNO                  1
Date_            20160805
Time                22.50
INSTRUM             spect
PROBHD   5 mm CPPBBO BB
PULPROG      hsqcetgpsi2
TD                   1024
SOLVENT             CDCl3
NS                     16
DS                     16
SWH              4302.926 Hz
FIDRES           4.202076 Hz
AQ              0.1190388 sec
RG                  208.5
DW                116.200 usec
DE                  10.00 usec
TE                  297.0 K
CNST2         145.0000000
D0             0.00000300 sec
D1             1.46497905 sec
D4             0.00172414 sec
D11            0.03000000 sec
D16            0.00020000 sec
D24            0.00086207 sec
IN0            0.00002080 sec
ZGOPTNS

======== CHANNEL f1 ========
SFO1         400.1320007 MHz
NUC1                  1H
P1                 11.50 usec
P2                 23.00 usec
P28                 0.00 usec
ND0                    2
TD                   256
SFO1            100.6233 MHz
FIDRES         93.900238 Hz
SW               238.896 ppm
FnMODE      Echo-Antiecho
SI                  1024
SF           400.1300061 MHz
WDW                QSINE
SSB                    2
LB                  0.00 Hz
GB                     0
PC                  1.40
SI                  1024
MC2         echo-antiecho
SF           100.6127554 MHz
WDW                QSINE
SSB                    2
LB                  0.00 Hz
```

HSQC (400 MHz) spectrum of compound **7** in $CDCl_3$

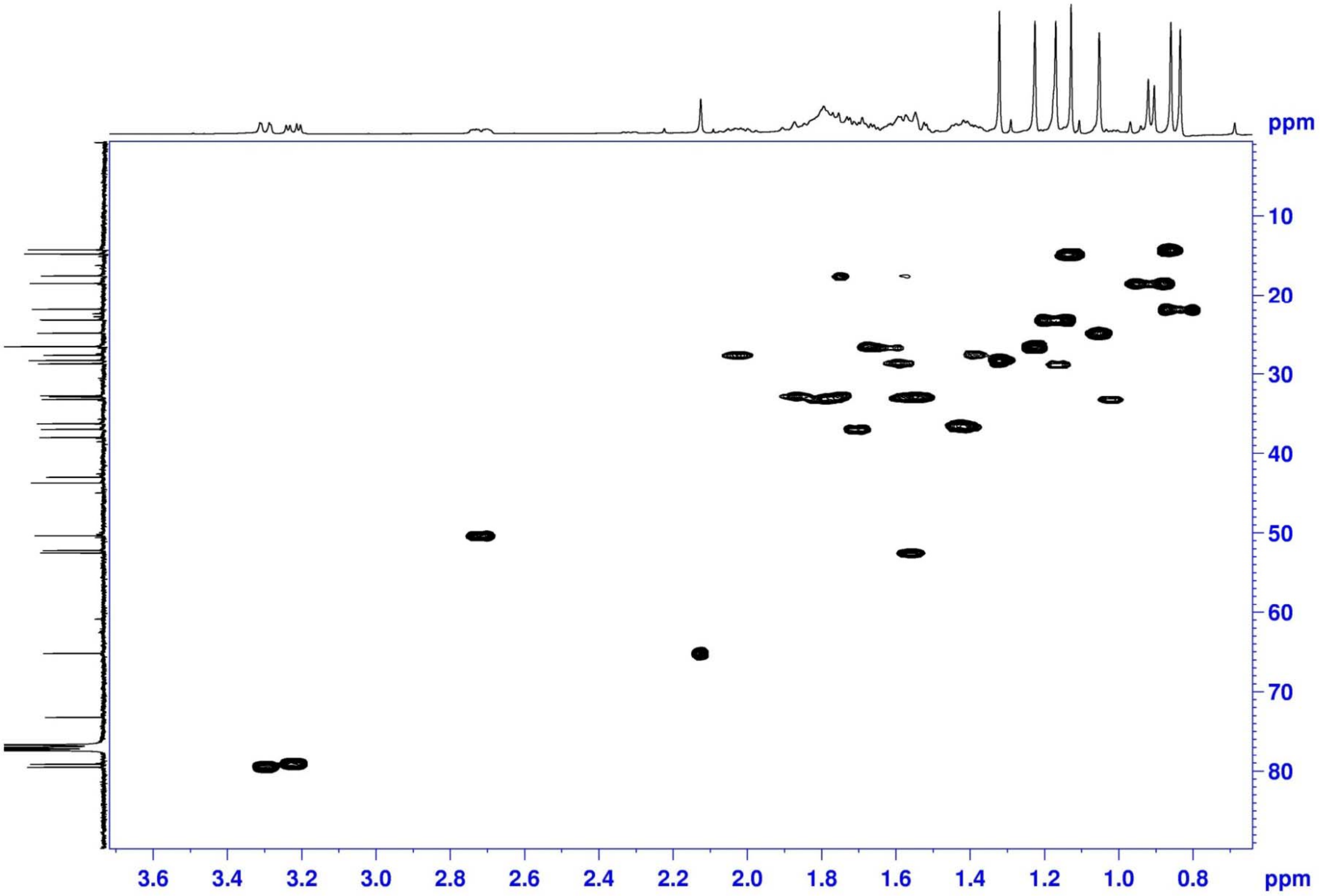

HSQC (400 MHz) spectrum of compound **7** in $CDCl_3$

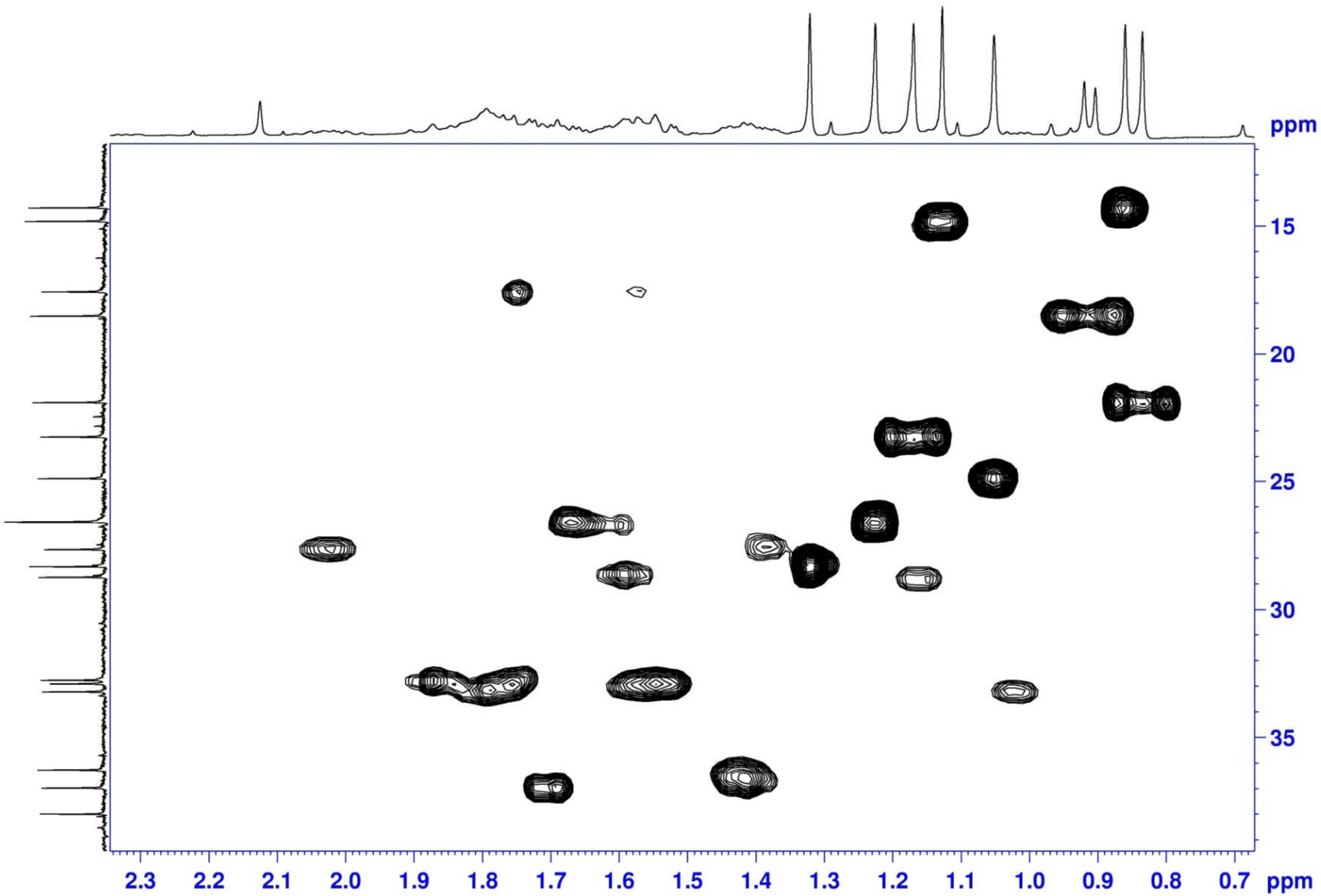

HMBC (400 MHz) spectrum of compound **7** in $CDCl_3$

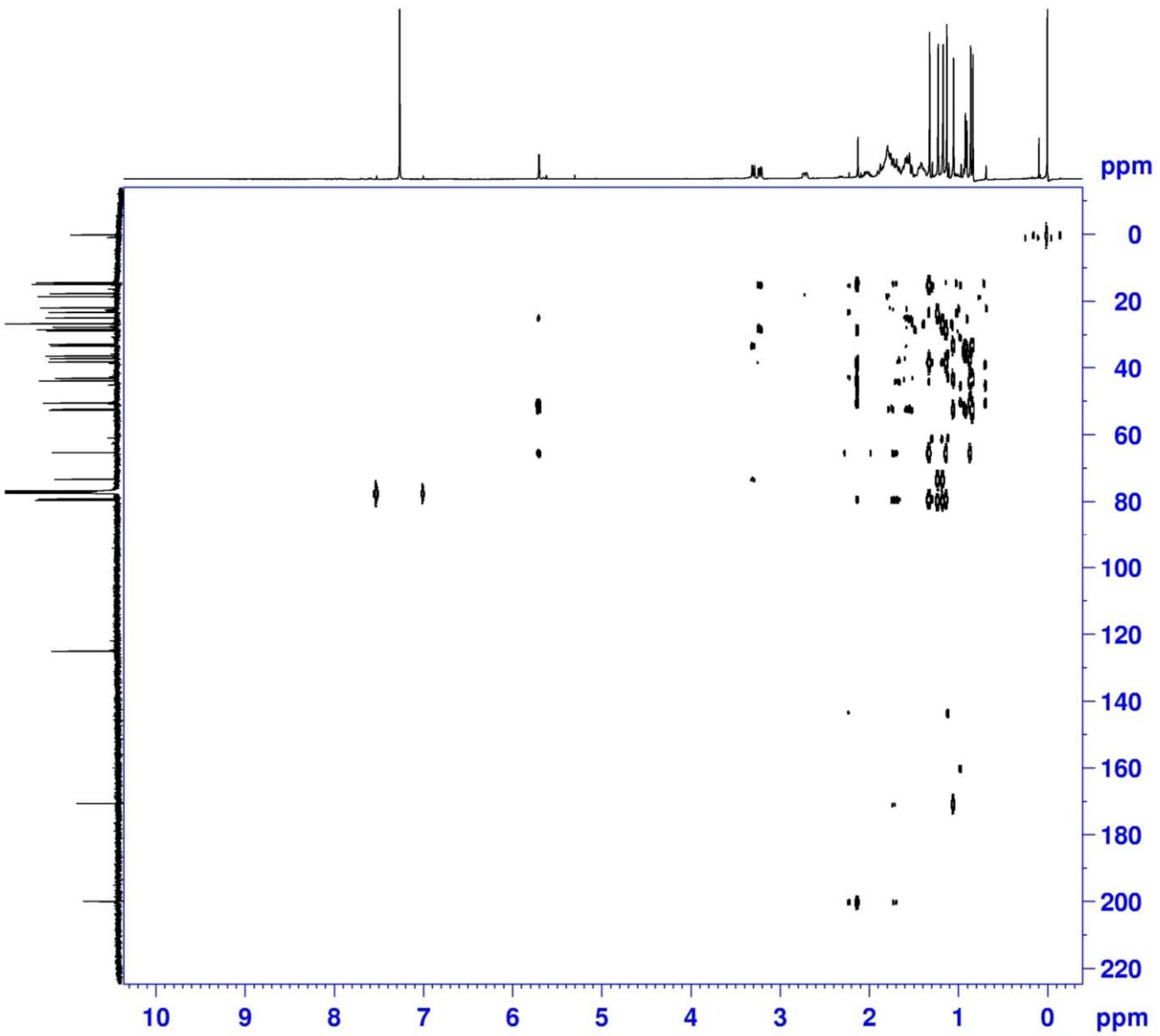


```
NAME                EA-17
EXPNO                 106
PROCNO                  1
Date_            20160806
Time                 0.41
INSTRUM              spect
PROBHD     5 mm CPPBBO BB
PULPROG      hmbcgplpndqf
TD                   4096
SOLVENT             CDCl3
NS                     32
DS                     16
SWH              5197.505 Hz
FIDRES           1.268922 Hz
AQ              0.3940852 sec
RG                  208.5
DW                 96.200 usec
DE                  10.00 usec
TE                  297.0 K
CNST2         145.0000000
CNST13         10.0000000
D0             0.00000300 sec
D1             1.50000000 sec
D2             0.00344828 sec
D6             0.05000000 sec
D16            0.00020000 sec
IN0            0.00002080 sec

======== CHANNEL f1 ========
SFO1          400.1323208 MHz
NUC1                   1H
P1                  11.50 usec
P2                  23.00 usec
ND0                     2
TD                    128
SFO1             100.6233 MHz
FIDRES         187.800476 Hz
SW                238.896 ppm
FnMODE                 QF
SI                   2048
SF            400.1300075 MHz
WDW                  SINE
SSB                     0
LB                   0.00 Hz
GB                      0
PC                   1.40
SI                   1024
MC2                    QF
SF            100.6127394 MHz
WDW                  SINE
SSB                     0
LB                   0.00 Hz
```

HMBC (400 MHz) spectrum of compound **7** in $CDCl_3$

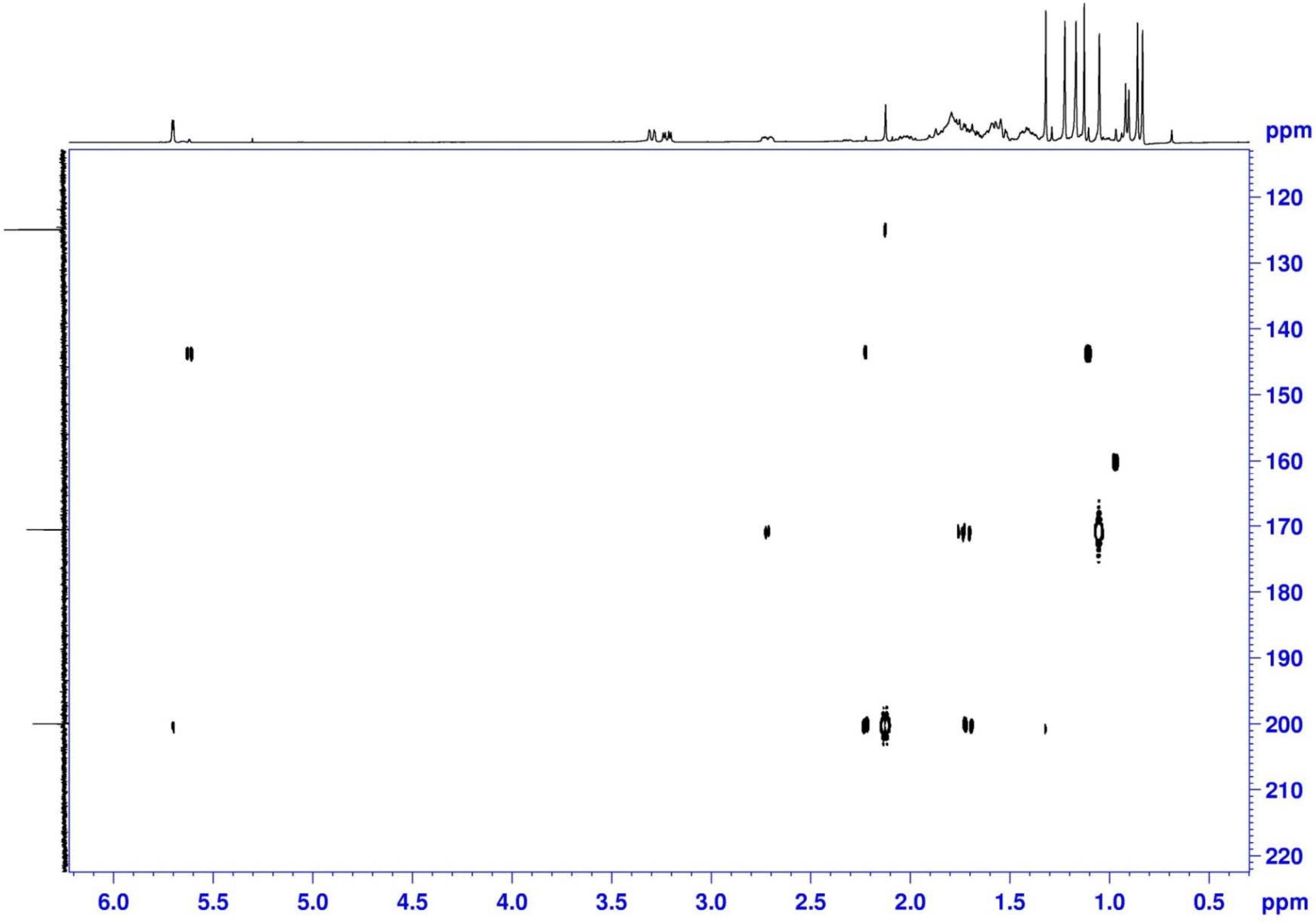

HMBC (400 MHz) spectrum of compound **7** in $CDCl_3$

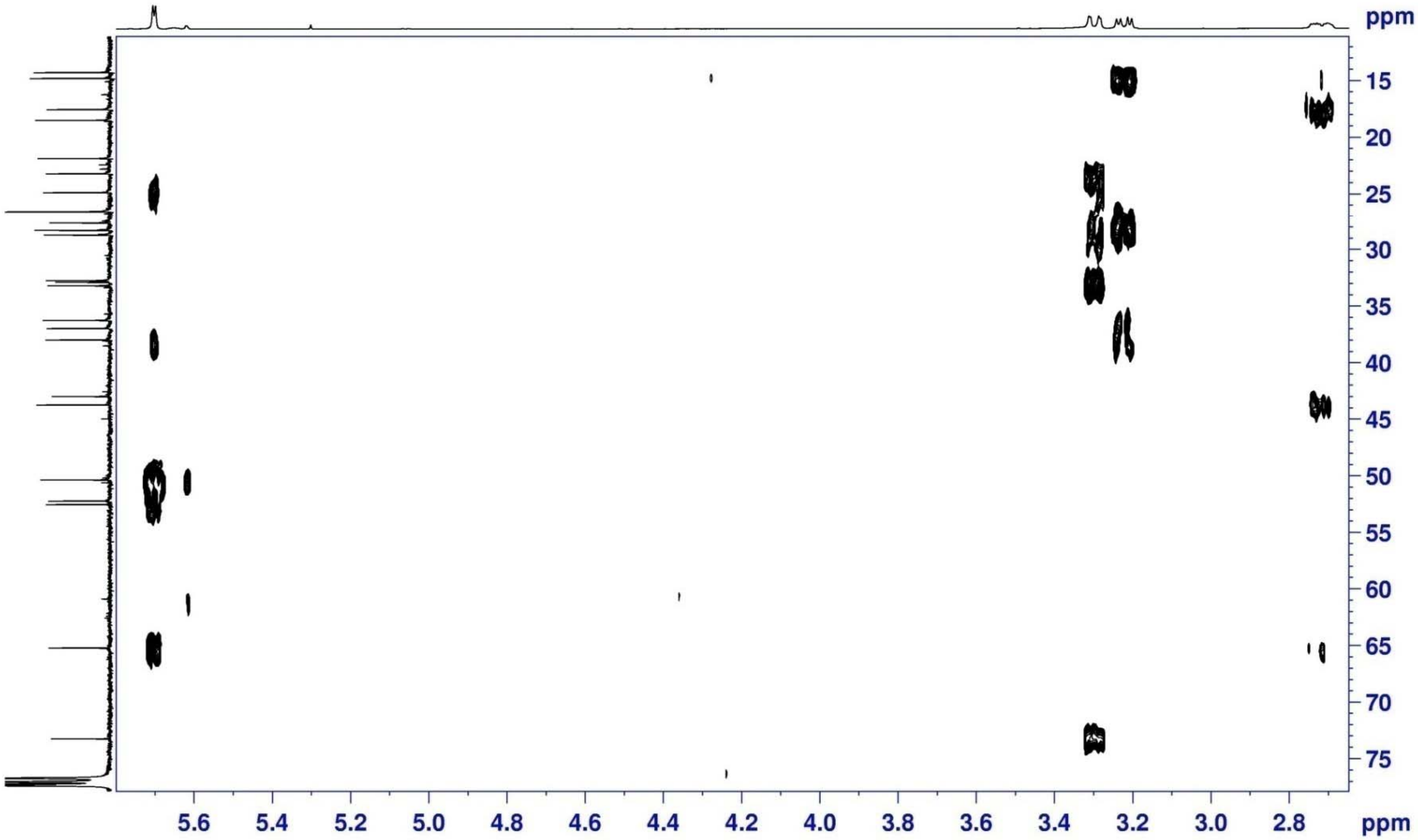

HMBC (400 MHz) spectrum of compound **7** in $CDCl_3$

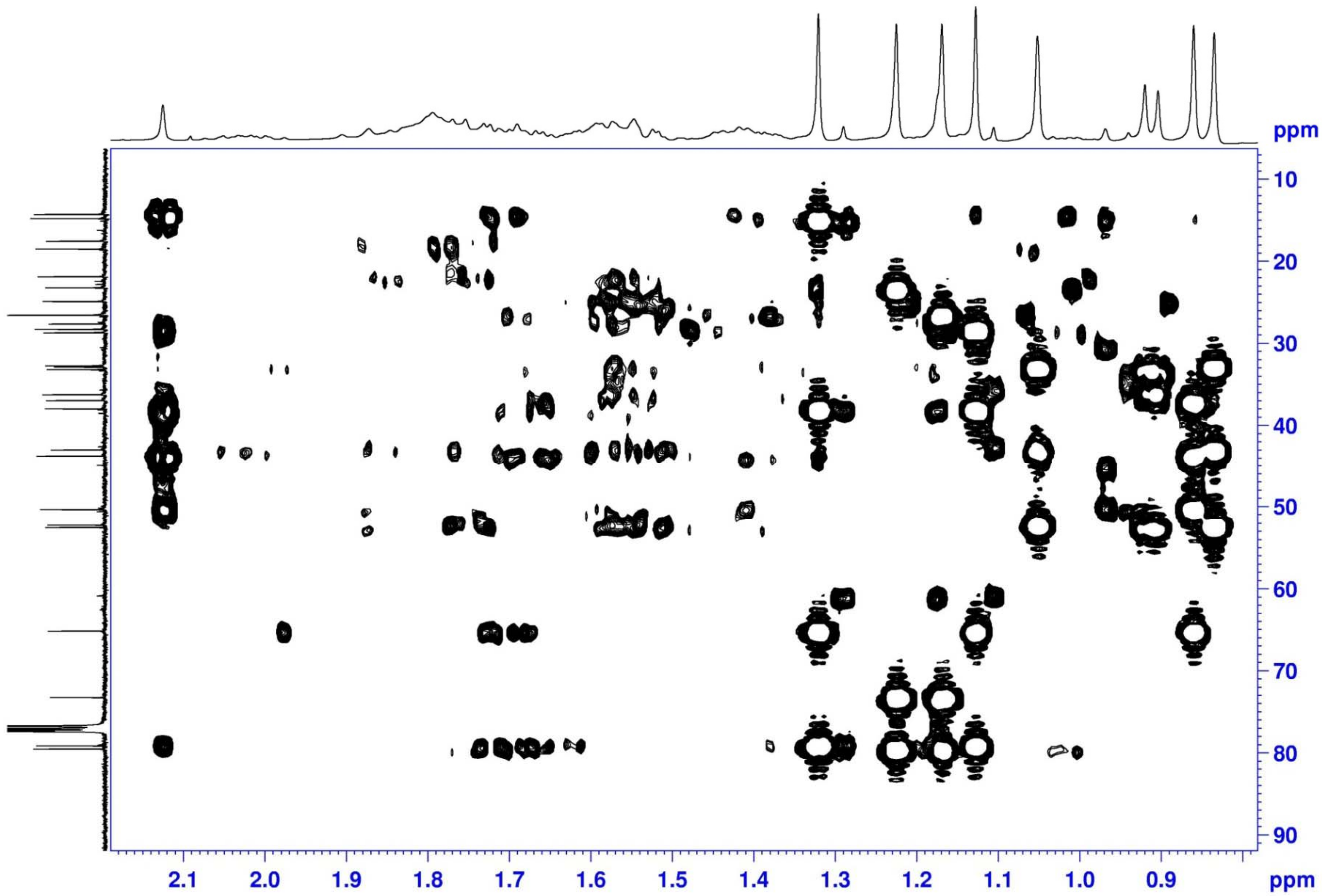

HMBC (400 MHz) spectrum of compound **7** in $CDCl_3$

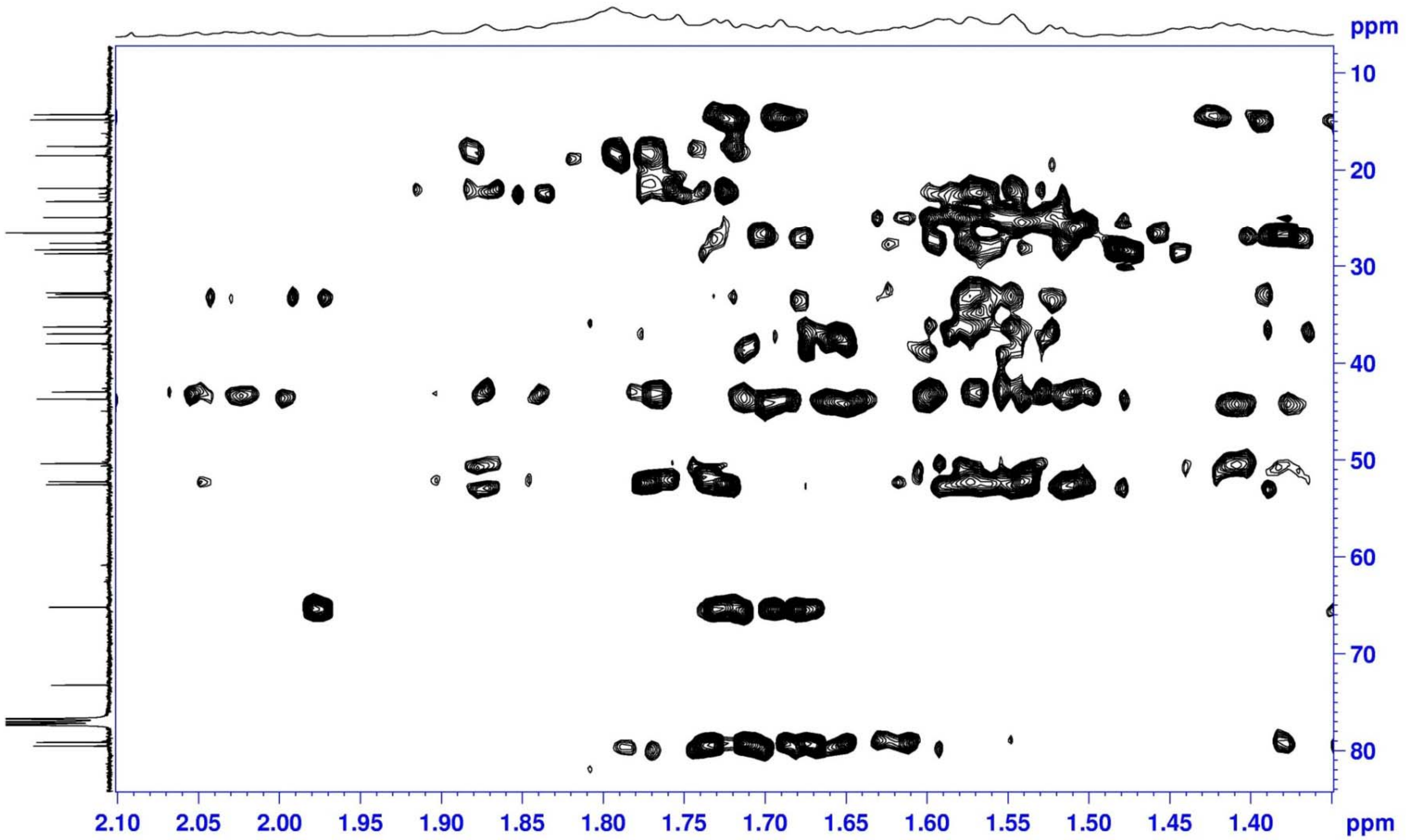

HMBC (400 MHz) spectrum of compound **7** in $CDCl_3$

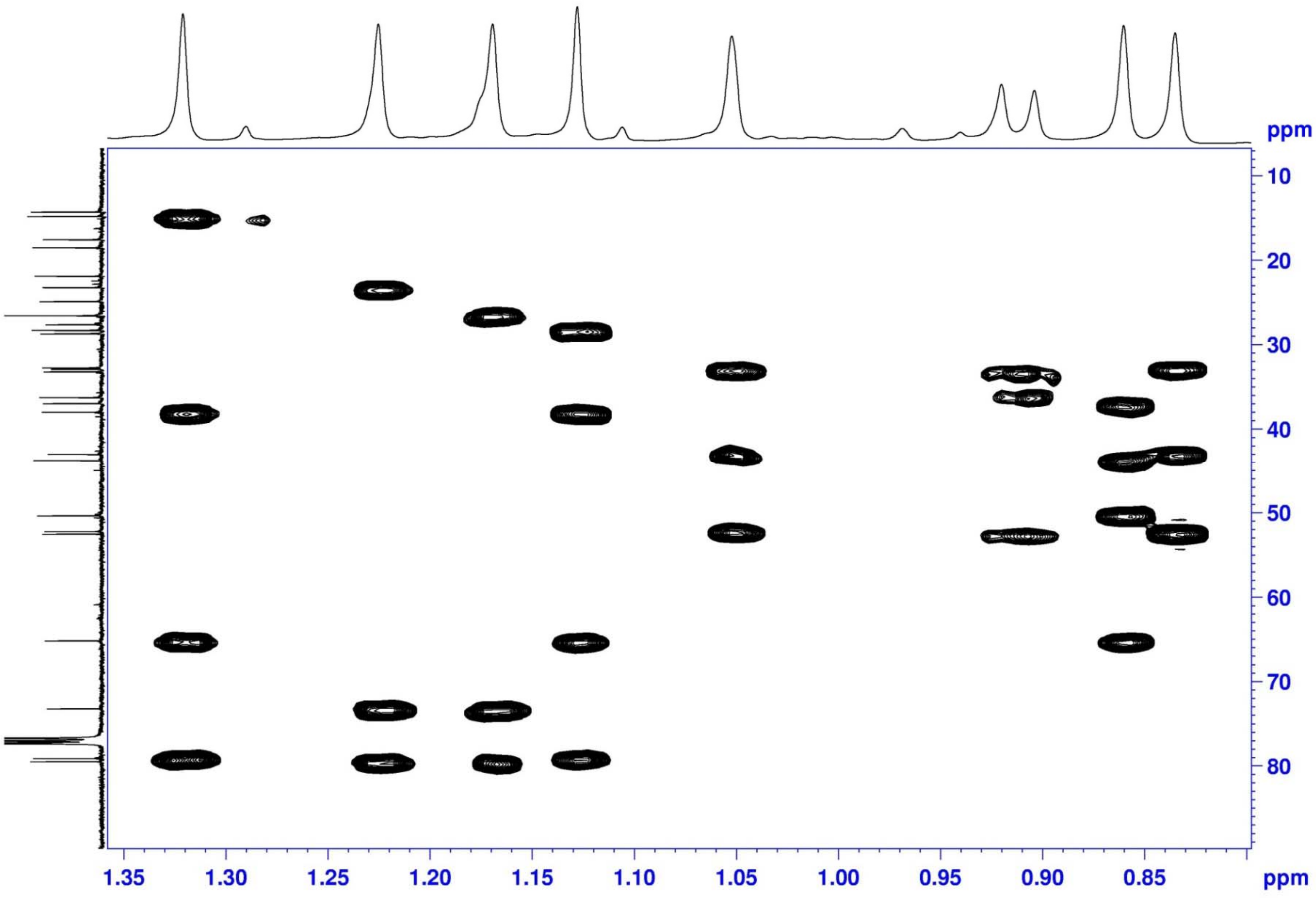

NOESY (400 MHz) spectrum of compound **7** in $CDCl_3$

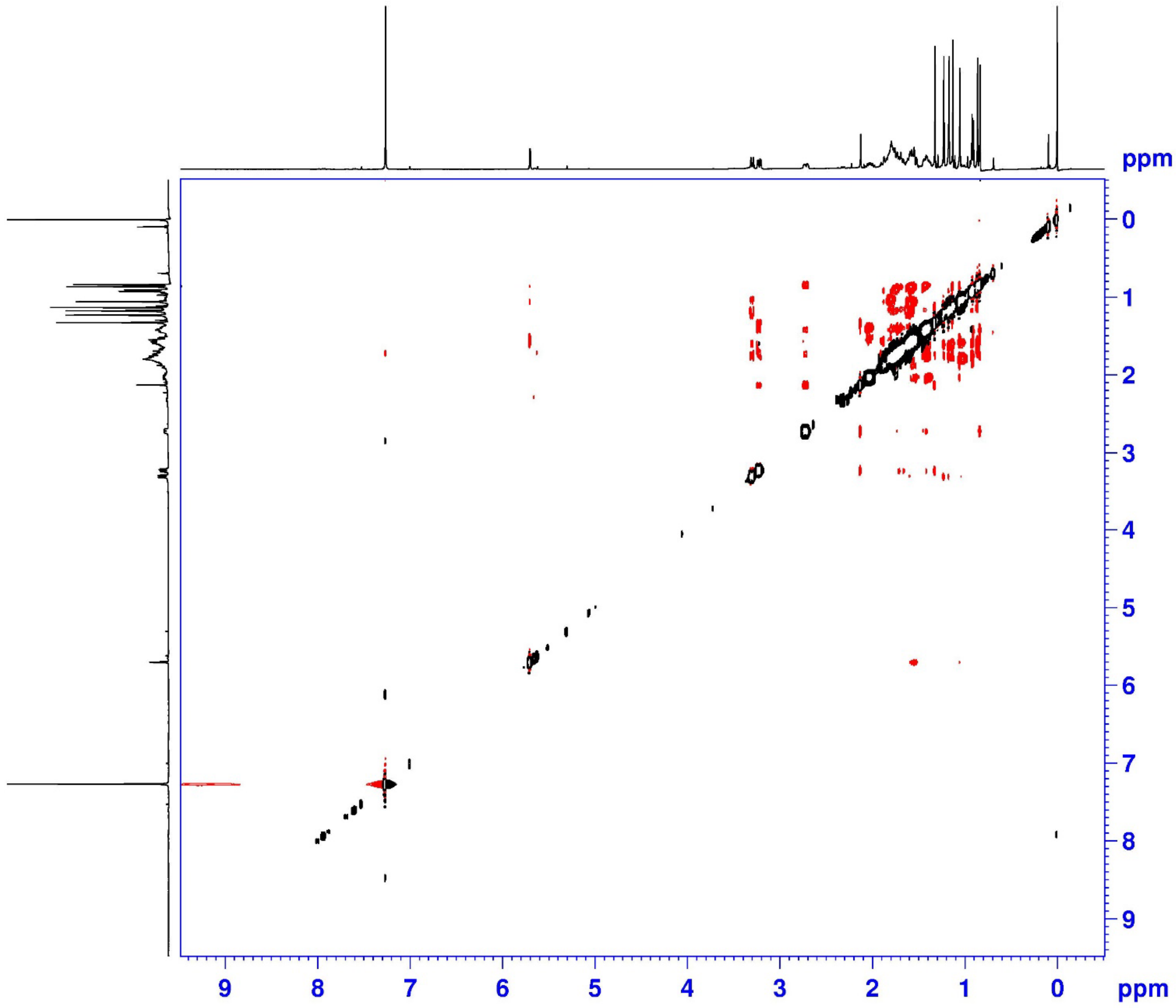

NAME	EA-17	
EXPNO	107	
PROCNO	1	
Date_	20160806	
Time	2.56	
INSTRUM	spect	
PROBHD	5 mm CPPBBO BB	
PULPROG	noesygpphpp	
TD	2048	
SOLVENT	CDCl3	
NS	16	
DS	32	
SWH	4000.000	Hz
FIDRES	1.953125	Hz
AQ	0.2560500	sec
RG	208.5	
DW	125.000	usec
DE	10.00	usec
TE	297.0	K
D0	0.00011036	sec
D1	1.99385595	sec
D8	0.30000001	sec
D11	0.03000000	sec
D12	0.00002000	sec
D16	0.00020000	sec
IN0	0.00025000	sec
======== CHANNEL f1 ========		
SFO1	400.1318006	MHz
NUC1	1H	
P1	11.50	usec
P2	23.00	usec
P17	2500.00	usec
ND0	1	
TD	256	
SFO1	400.1318	MHz
FIDRES	15.625000	Hz
SW	9.997	ppm
FnMODE	States-TPPI	
SI	1024	
SF	400.1300055	MHz
WDW	QSINE	
SSB	2	
LB	0.00	Hz
GB	0	
PC	1.00	
SI	1024	
MC2	States-TPPI	
SF	400.1300064	MHz
WDW	QSINE	
SSB	2	

NOESY (400 MHz) spectrum of compound **7** in $CDCl_3$

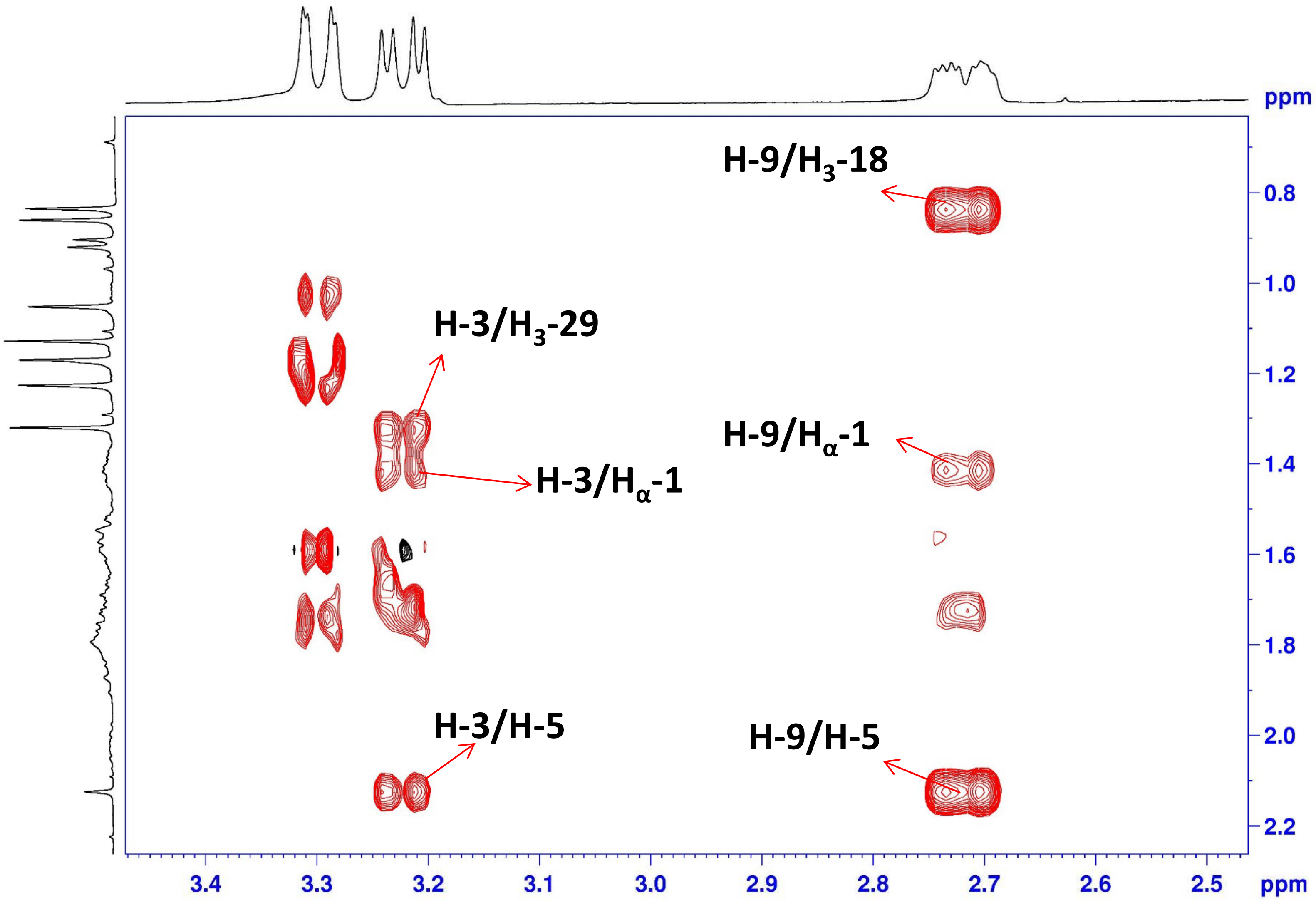

NOESY (400 MHz) spectrum of compound **7** in $CDCl_3$

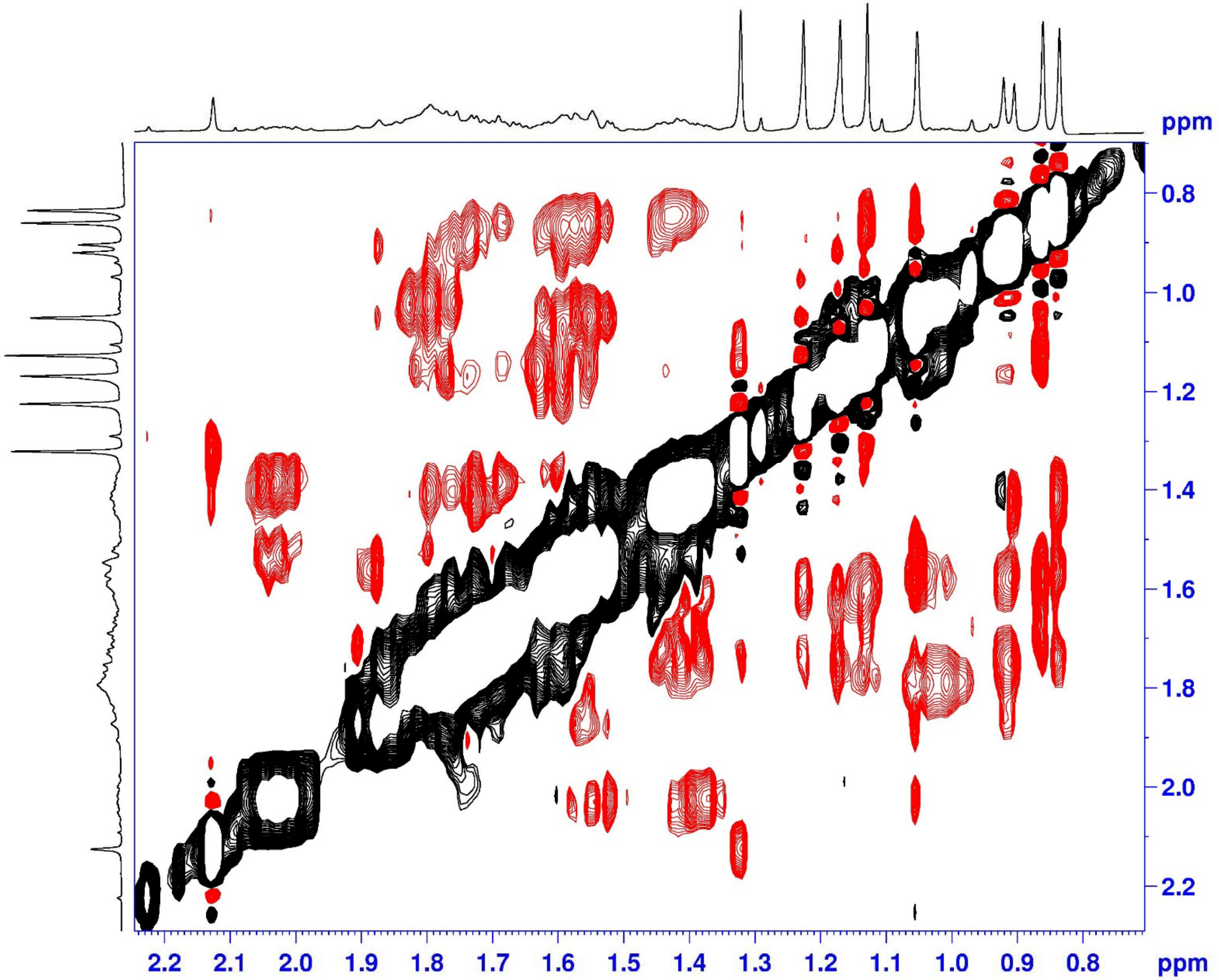

NOESY (400 MHz) spectrum of compound **7** in $CDCl_3$

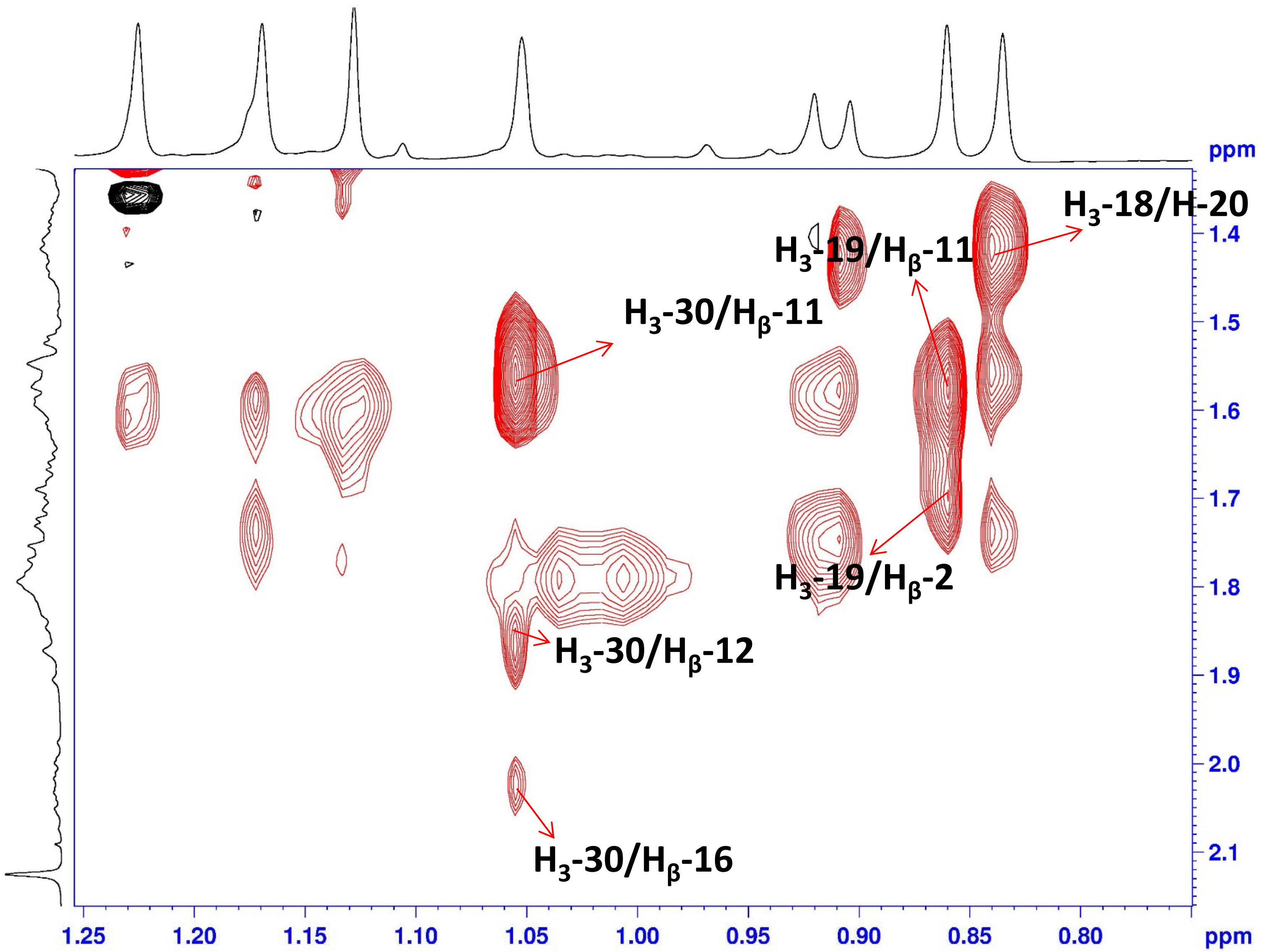

HR-ESIMS for compound **8**

Elemental Composition Report **Page 1**

Single Mass Analysis
Tolerance = 5.0 PPM / DBE: min = -1.5, max = 50.0
Element prediction: Off
Number of isotope peaks used for i-FIT = 3

Monoisotopic Mass, Even Electron Ions
360 formula(e) evaluated with 2 results within limits (up to 50 closest results for each mass)
Elements Used:
C: 0-50 H: 0-100 N: 0-1 O: 0-20 Na: 0-1
EA3-44-3-1
201807308 258 (2.075)

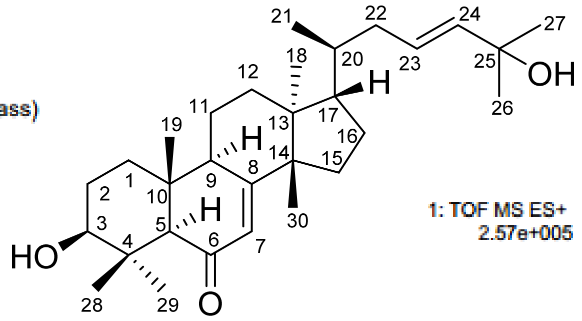

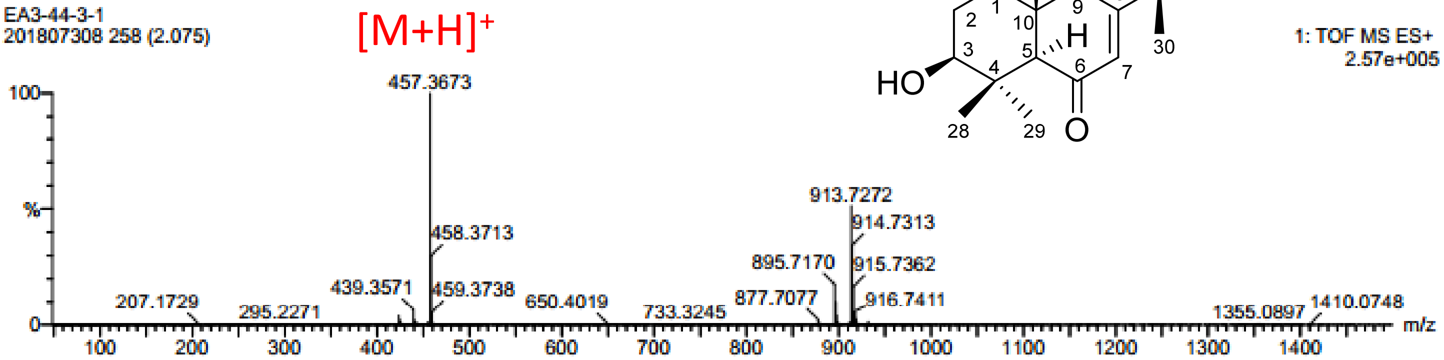

Minimum:				-1.5				
Maximum:		5.0	5.0	50.0				

Mass	Calc. Mass	mDa	PPM	DBE	i-FIT	Norm	Conf(%)	Formula
457.3673	457.3682	-0.9	-2.0	6.5	415.9	2.086	12.41	C30 H49 O3
	457.3658	1.5	3.3	3.5	414.0	0.133	87.59	C28 H50 O3 Na

^{1}H NMR (400 MHz) spectrum of compound **8** in $CDCl_3$

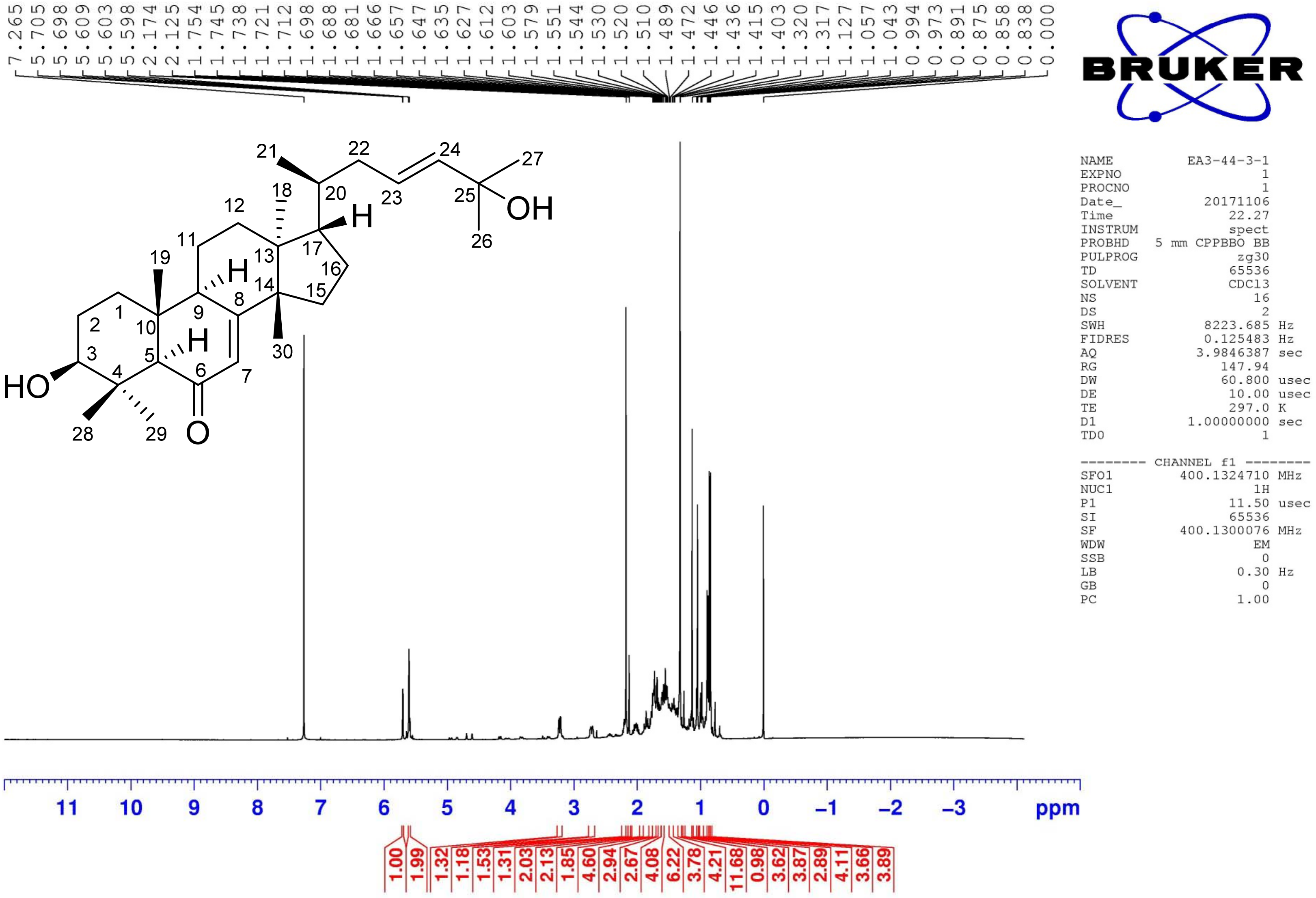

^{1}H NMR (400 MHz) spectrum of compound **8** in $CDCl_3$

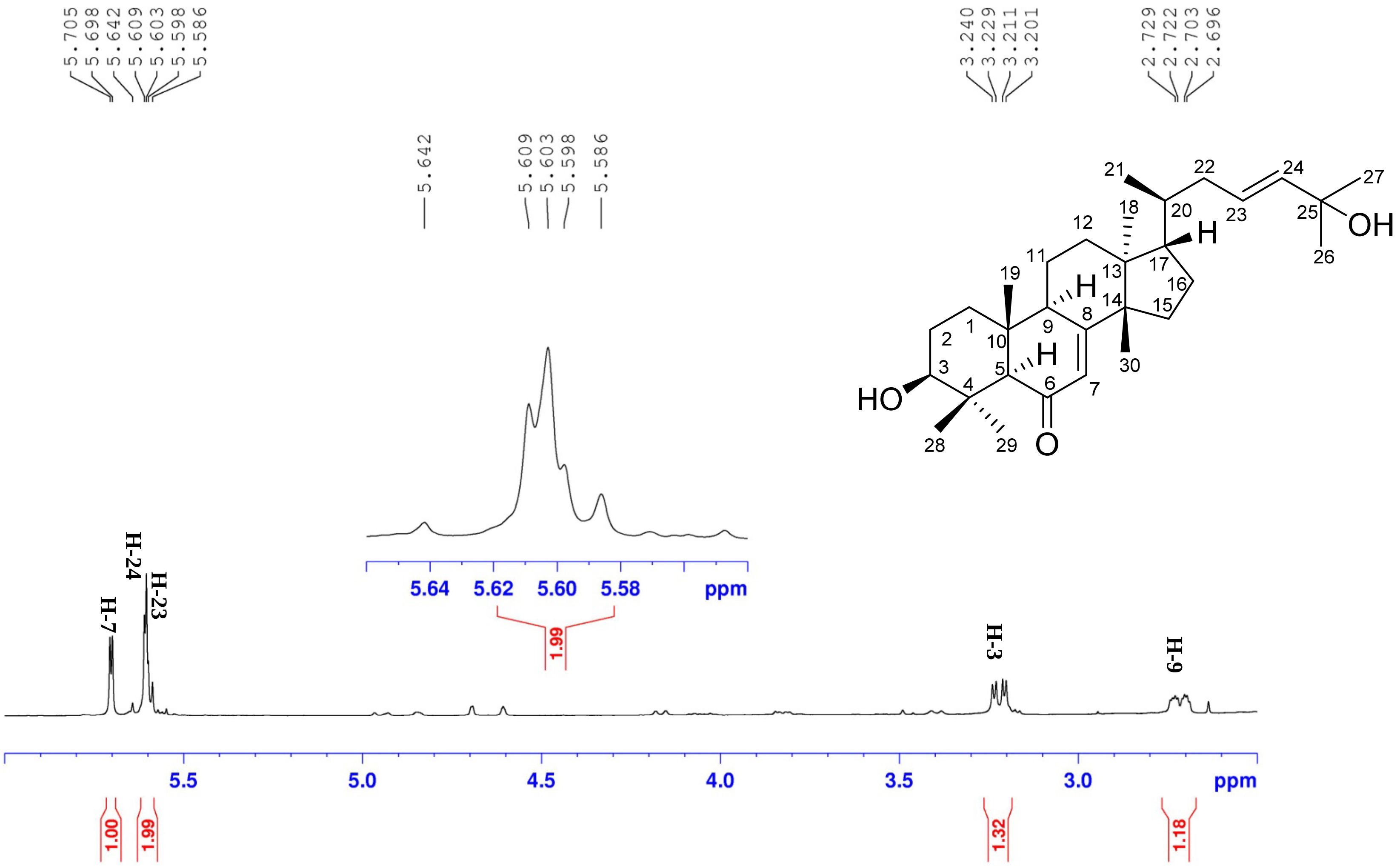

^{1}H NMR (400 MHz) spectrum of compound **8** in $CDCl_3$

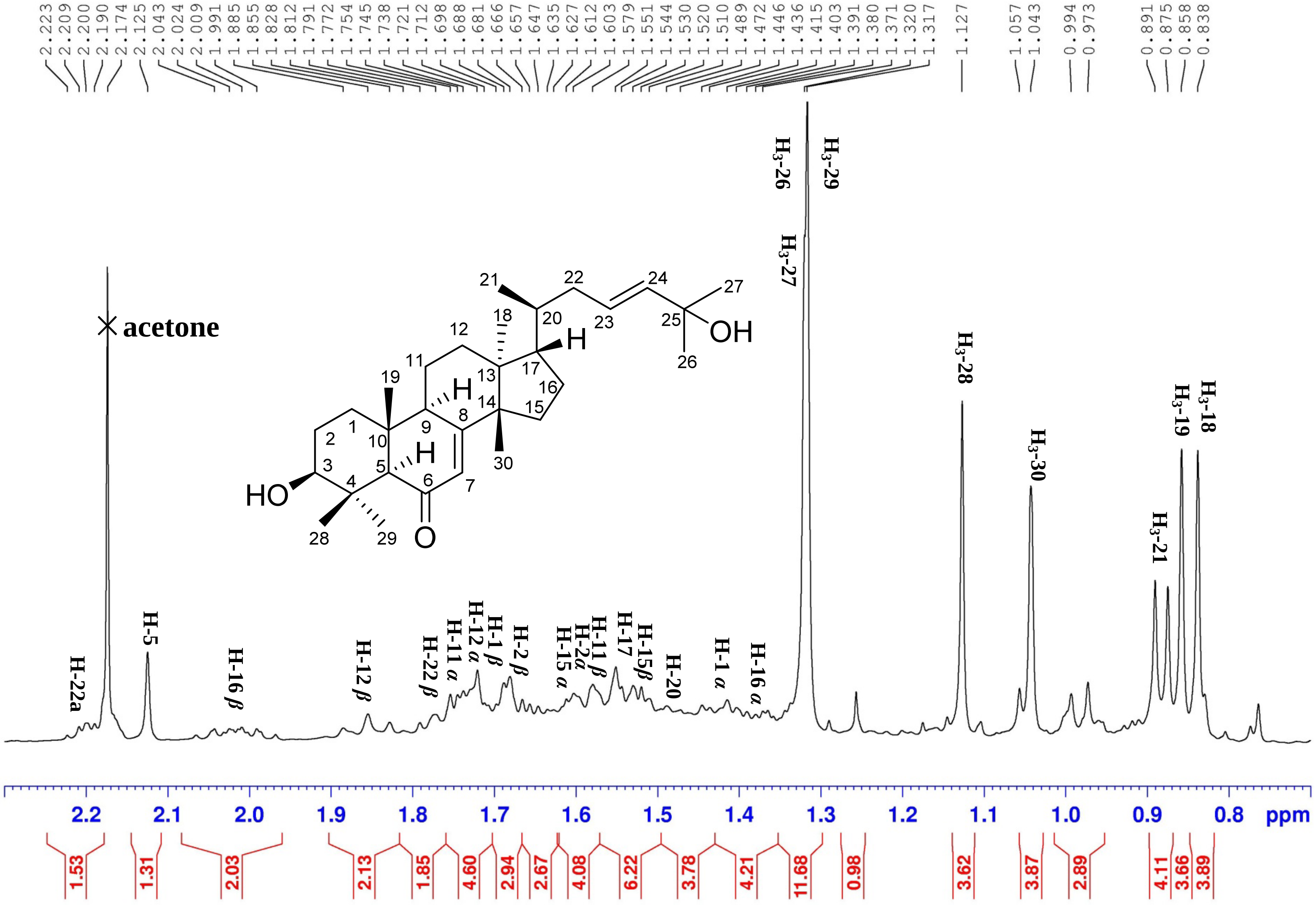

^{13}C NMR (100 MHz) spectrum of compound **8** in $CDCl_3$

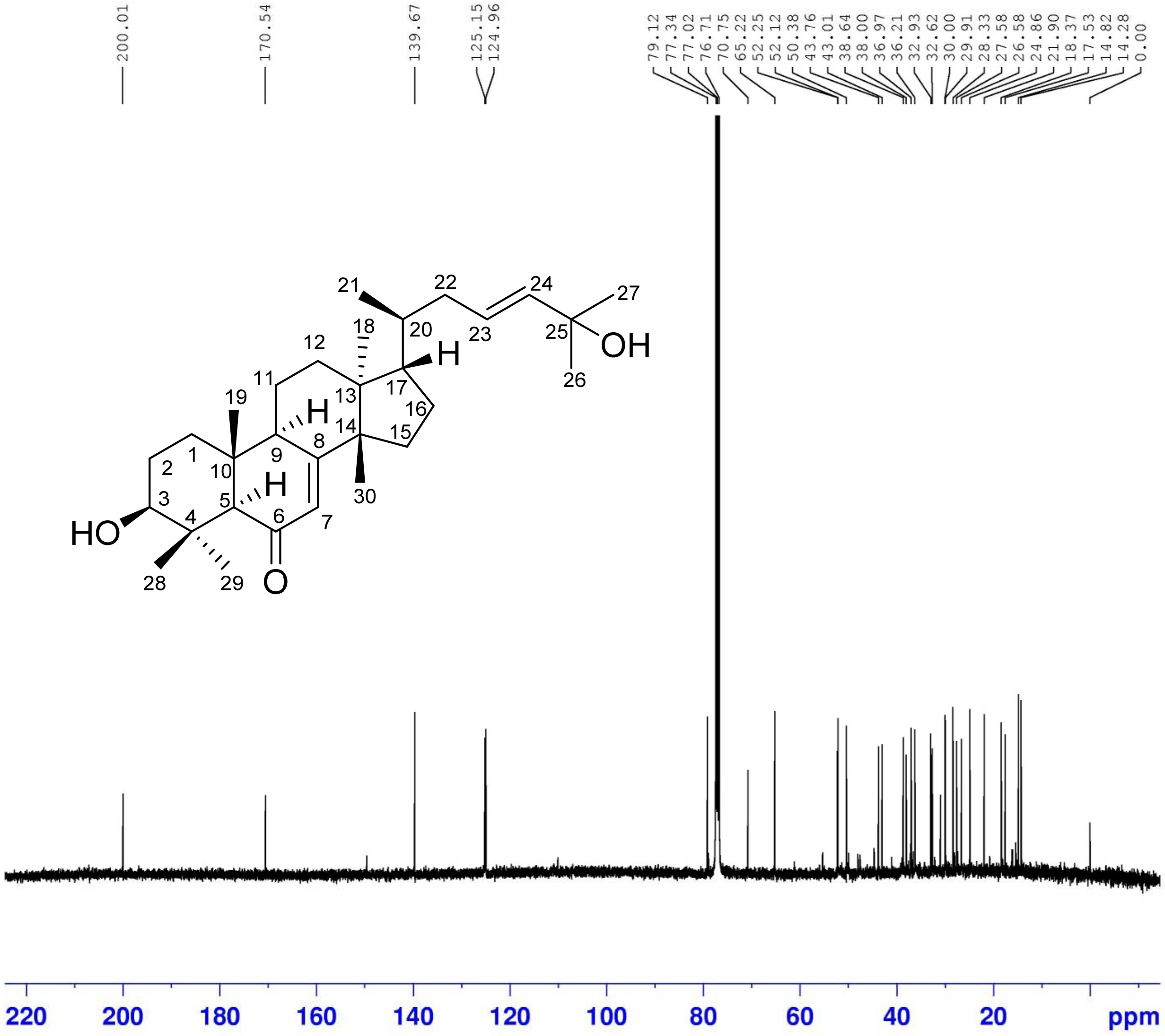


```
NAME          EA3-44-3-1
EXPNO                  2
PROCNO                 1
Date_           20171107
Time                2.49
INSTRUM            spect
PROBHD   5 mm CPPBBO BB
PULPROG          zgpg30
TD                 65536
SOLVENT            CDCl3
NS                  1024
DS                     4
SWH            24038.461 Hz
FIDRES          0.366798 Hz
AQ             1.3631988 sec
RG                117.37
DW                20.800 usec
DE                 18.00 usec
TE                 297.0 K
D1            2.00000000 sec
D11           0.03000000 sec
TD0                    1

======== CHANNEL f1 ========
SFO1         100.6233324 MHz
NUC1                 13C
P1                 10.00 usec
SI                 32768
SF           100.6127689 MHz
WDW                   EM
SSB                    0
LB                  1.00 Hz
GB                     0
PC                  1.40
```

^{13}C NMR (100 MHz) spectrum of compound **8** in $CDCl_3$

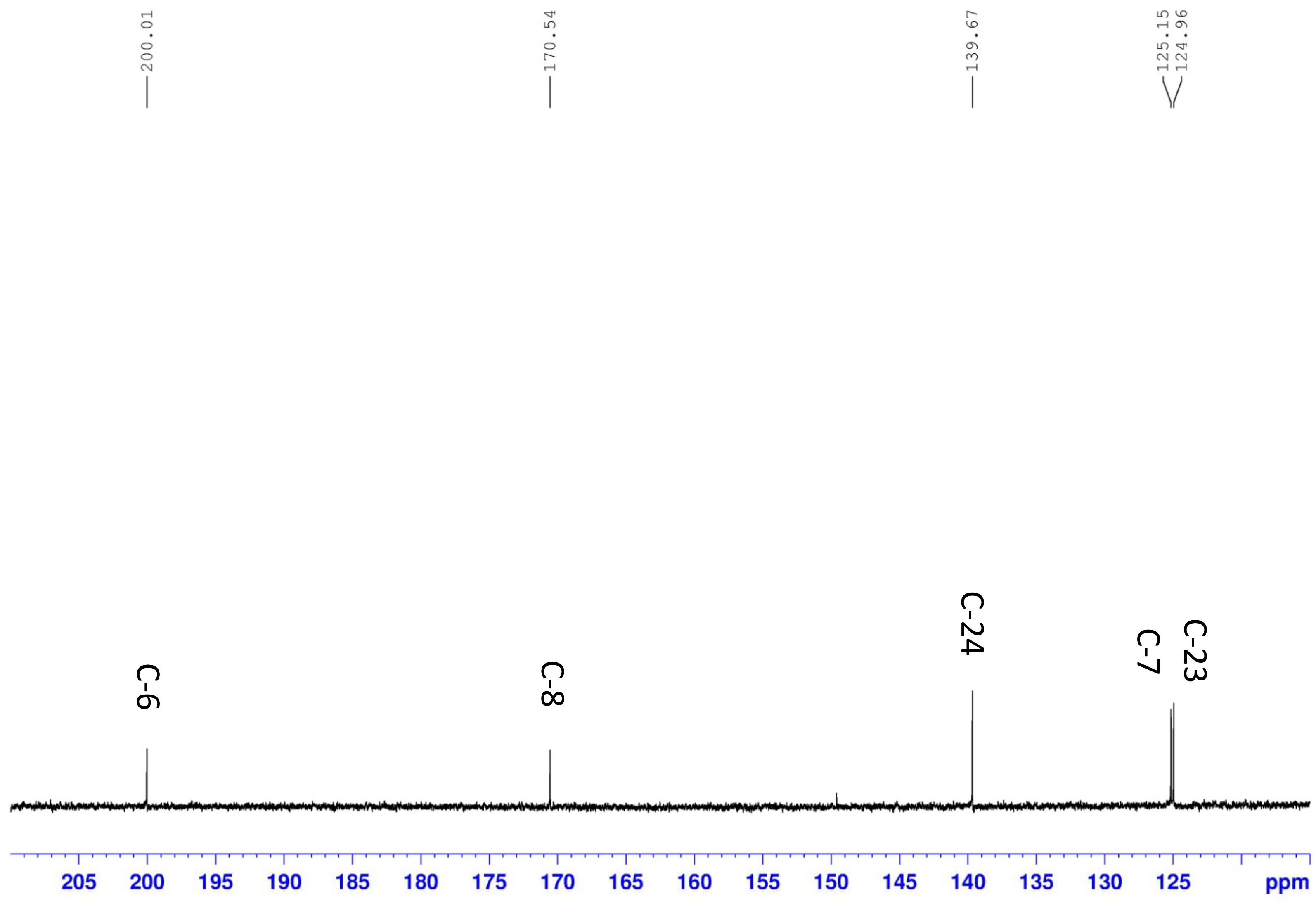

^{13}C NMR (100 MHz) spectrum of compound **8** in $CDCl_3$

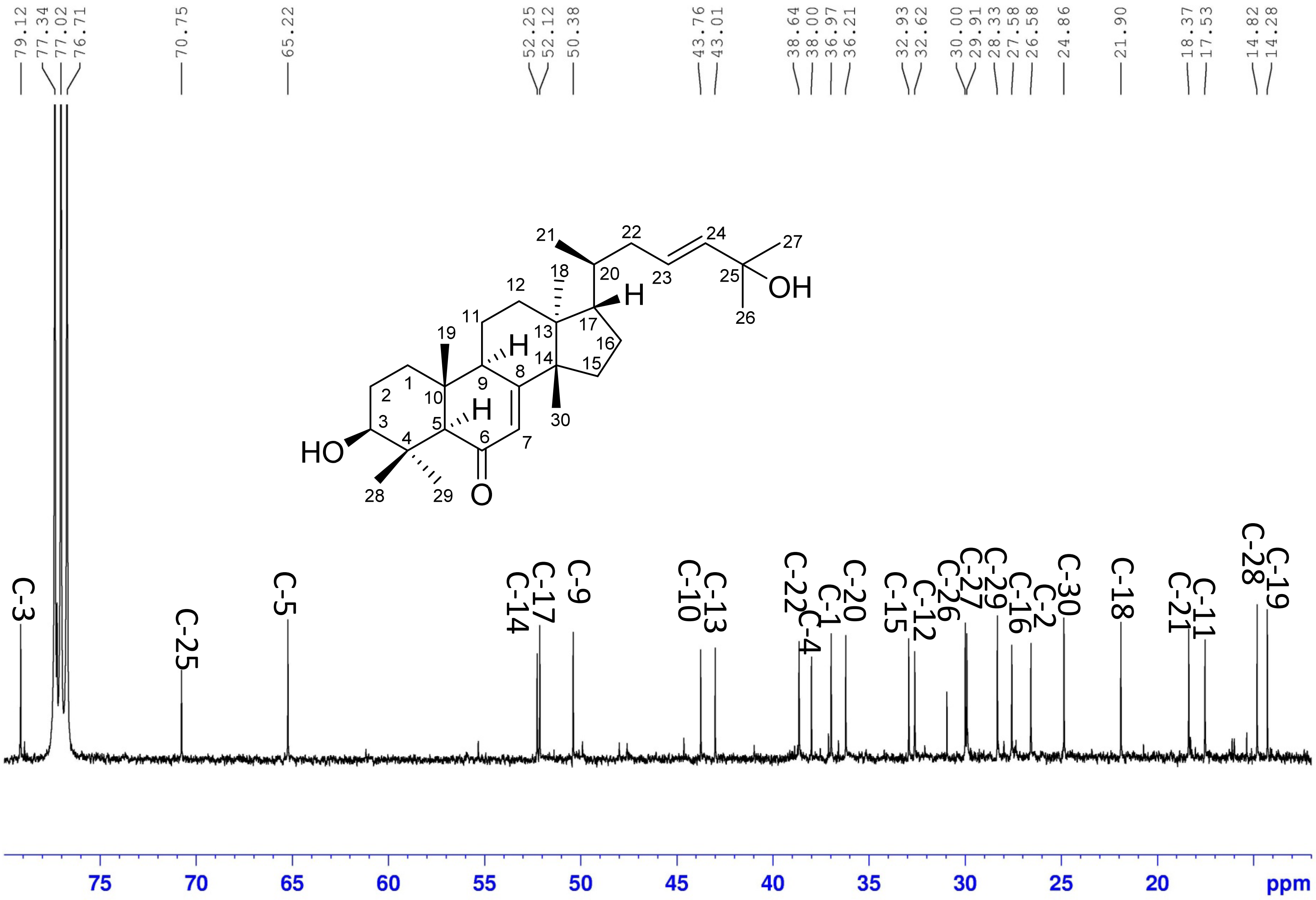

DEPT135 (100 MHz) spectrum of compound **8** in $CDCl_3$

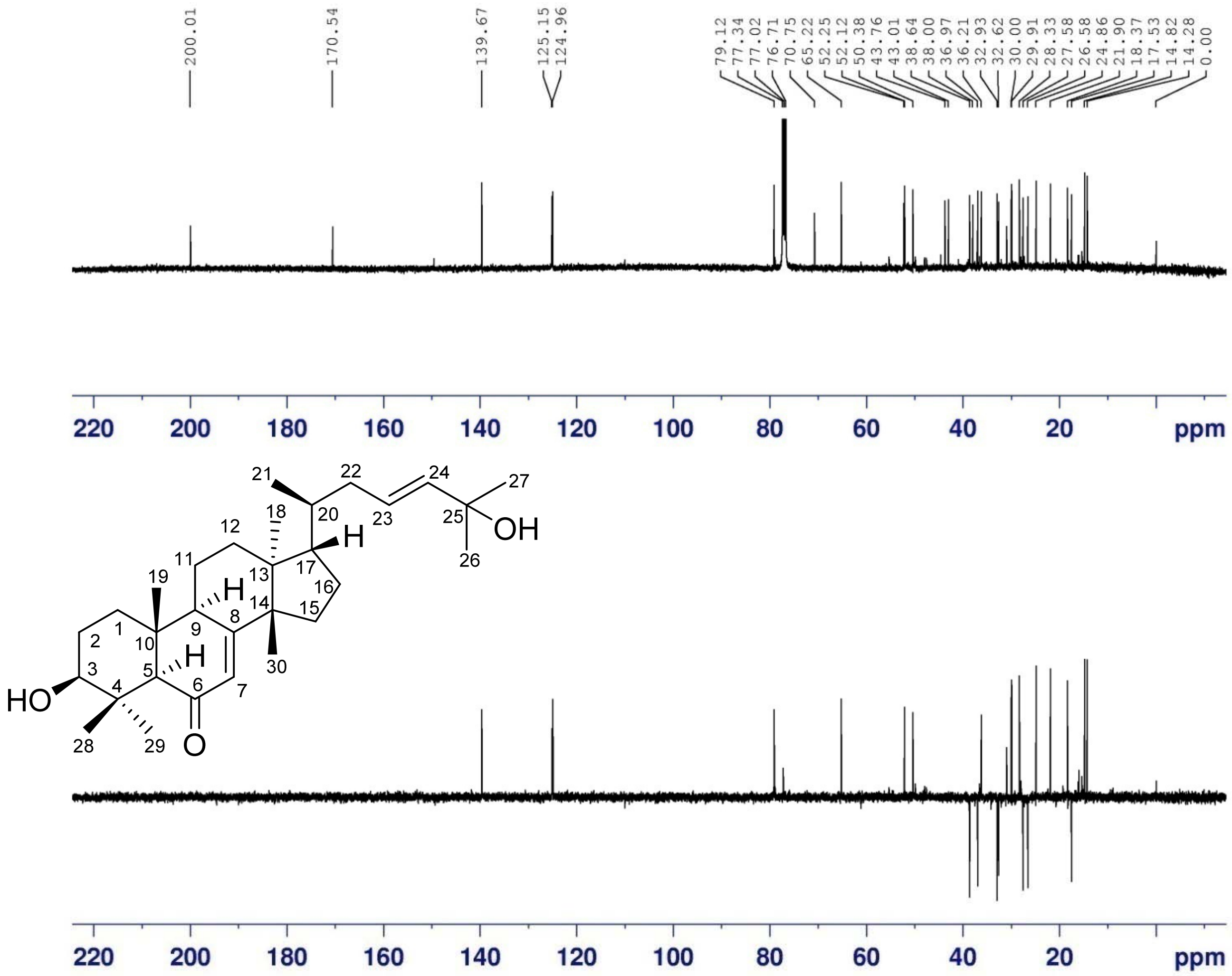

NAME	EA3-44-3-1
EXPNO	3
PROCNO	1
Date_	20171107
Time	3.07
INSTRUM	spect
PROBHD	5 mm CPPBBO BB
PULPROG	deptsp135
TD	65536
SOLVENT	CDCl3
NS	300
DS	4
SWH	24038.461 Hz
FIDRES	0.366798 Hz
AQ	1.3631988 sec
RG	130.26
DW	20.800 usec
DE	18.00 usec
TE	297.0 K
CNST2	145.0000000
D1	2.00000000 sec
D2	0.00344828 sec
D12	0.00002000 sec
TD0	1
======== CHANNEL f1 ========	
SFO1	100.6233324 MHz
NUC1	13C
P1	10.00 usec
P13	2000.00 usec
SI	32768
SF	100.6127689 MHz
WDW	EM
SSB	0
LB	1.00 Hz
GB	0
PC	1.40

DEPT135 (100 MHz) spectrum of compound **8** in $CDCl_3$

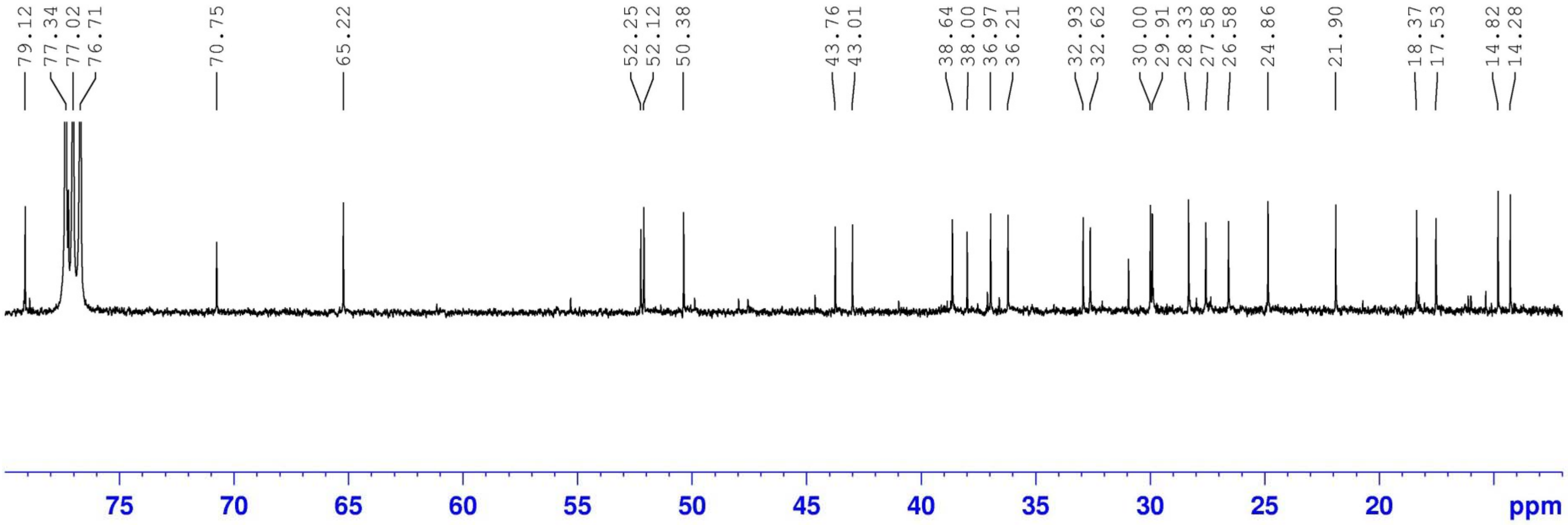

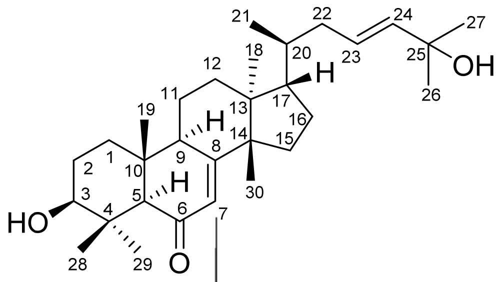

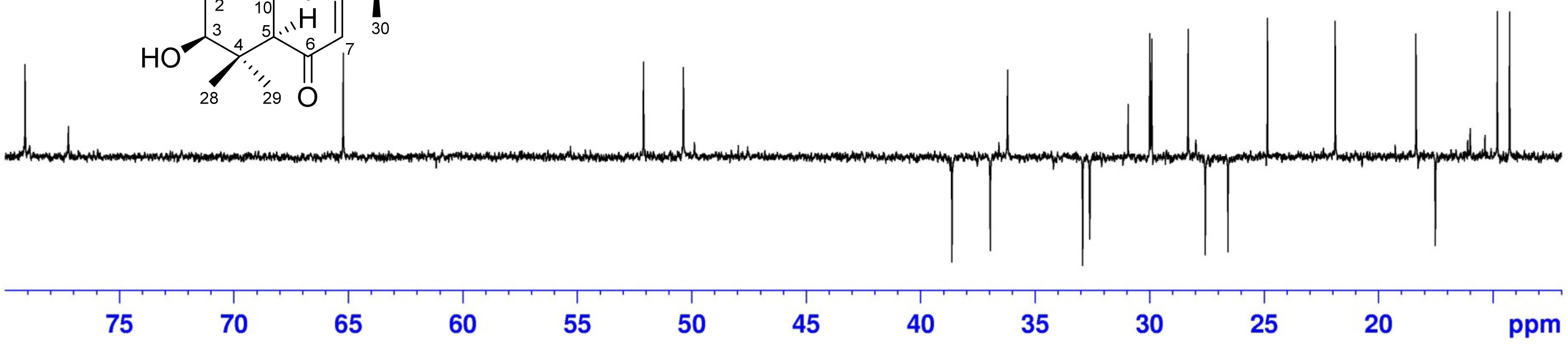

^{1}H-^{1}H COSY (400 MHz) spectrum of compound **8** in $CDCl_3$

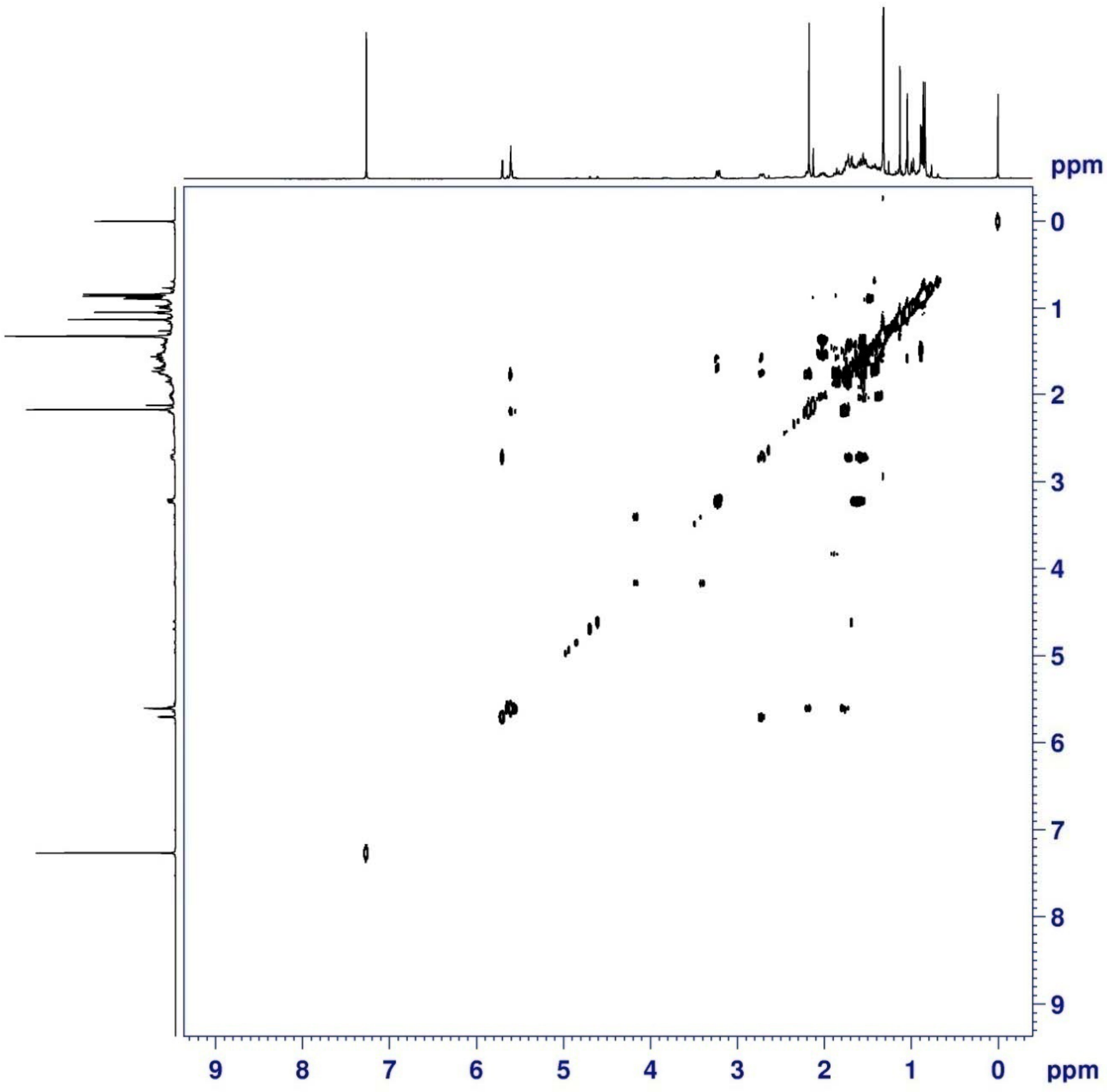


```
NAME            EA3-44-3-1
EXPNO                    4
PROCNO                   1
Date_             20171109
Time                 16.41
INSTRUM              spect
PROBHD   5 mm CPPBBO BB
PULPROG         cosygpppqf
TD                    2048
SOLVENT              CDCl3
NS                       8
DS                       8
SWH               3906.250 Hz
FIDRES            1.907349 Hz
AQ               0.2621940 sec
RG                   208.5
DW                 128.000 usec
DE                   10.00 usec
TE                   297.0 K
D0              0.00000300 sec
D1              1.89678097 sec
D11             0.03000000 sec
D12             0.00002000 sec
D13             0.00000400 sec
D16             0.00020000 sec
IN0             0.00025600 sec

======== CHANNEL f1 ========
SFO1           400.1318006 MHz
NUC1                    1H
P0                   11.50 usec
P1                   11.50 usec
P17                2500.00 usec
ND0                      1
TD                     128
SFO1              400.1318 MHz
FIDRES           30.517578 Hz
SW                   9.762 ppm
FnMODE                  QF
SI                    1024
SF             400.1300062 MHz
WDW                  QSINE
SSB                      0
LB                    0.00 Hz
GB                       0
PC                    1.40
SI                    1024
MC2                     QF
SF             400.1300068 MHz
WDW                  QSINE
SSB                      0
LB                    0.00 Hz
```

^{1}H-^{1}H COSY (400 MHz) spectrum of compound **8** in $CDCl_3$

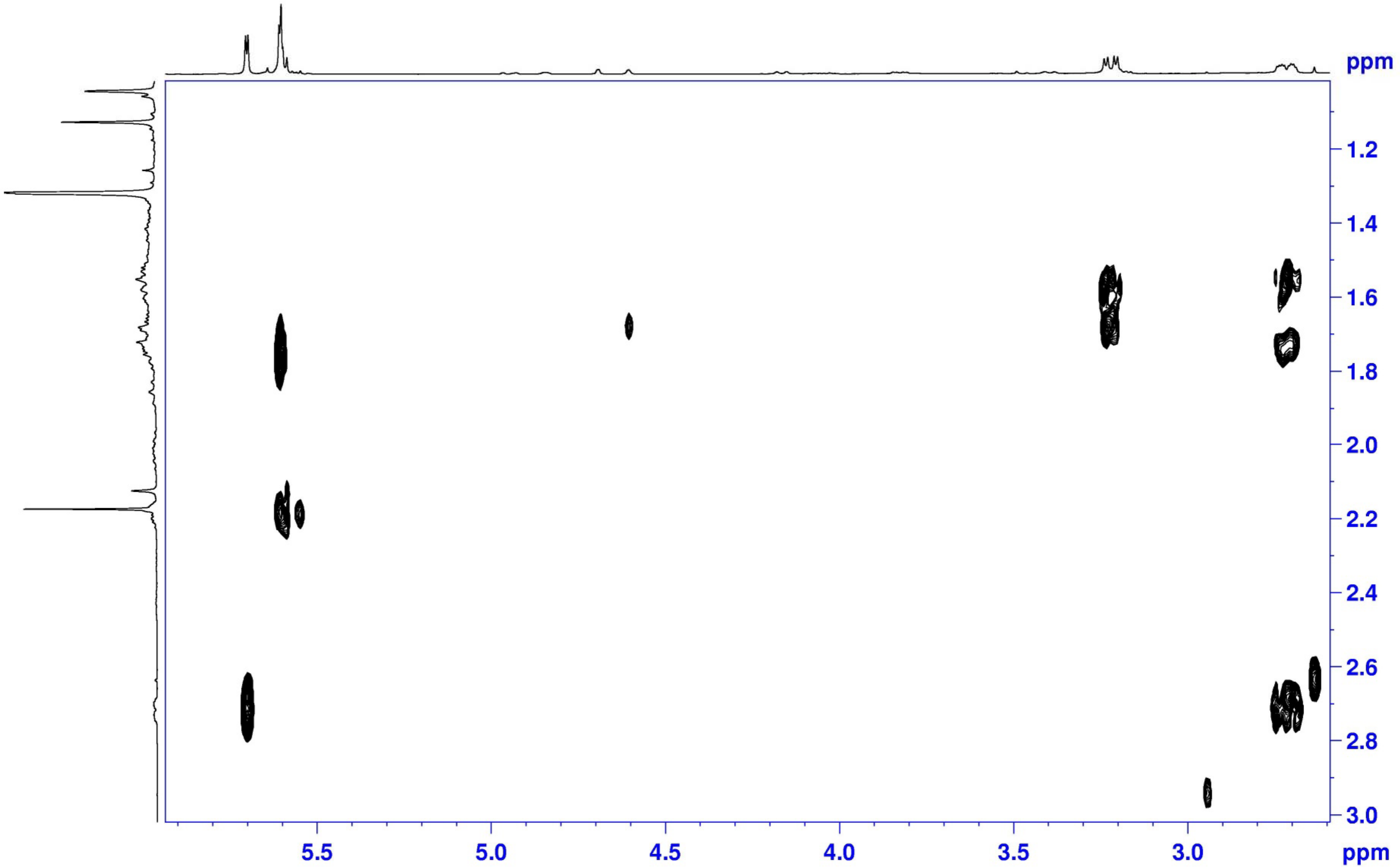

^{1}H-^{1}H COSY (400 MHz) spectrum of compound **8** in $CDCl_3$

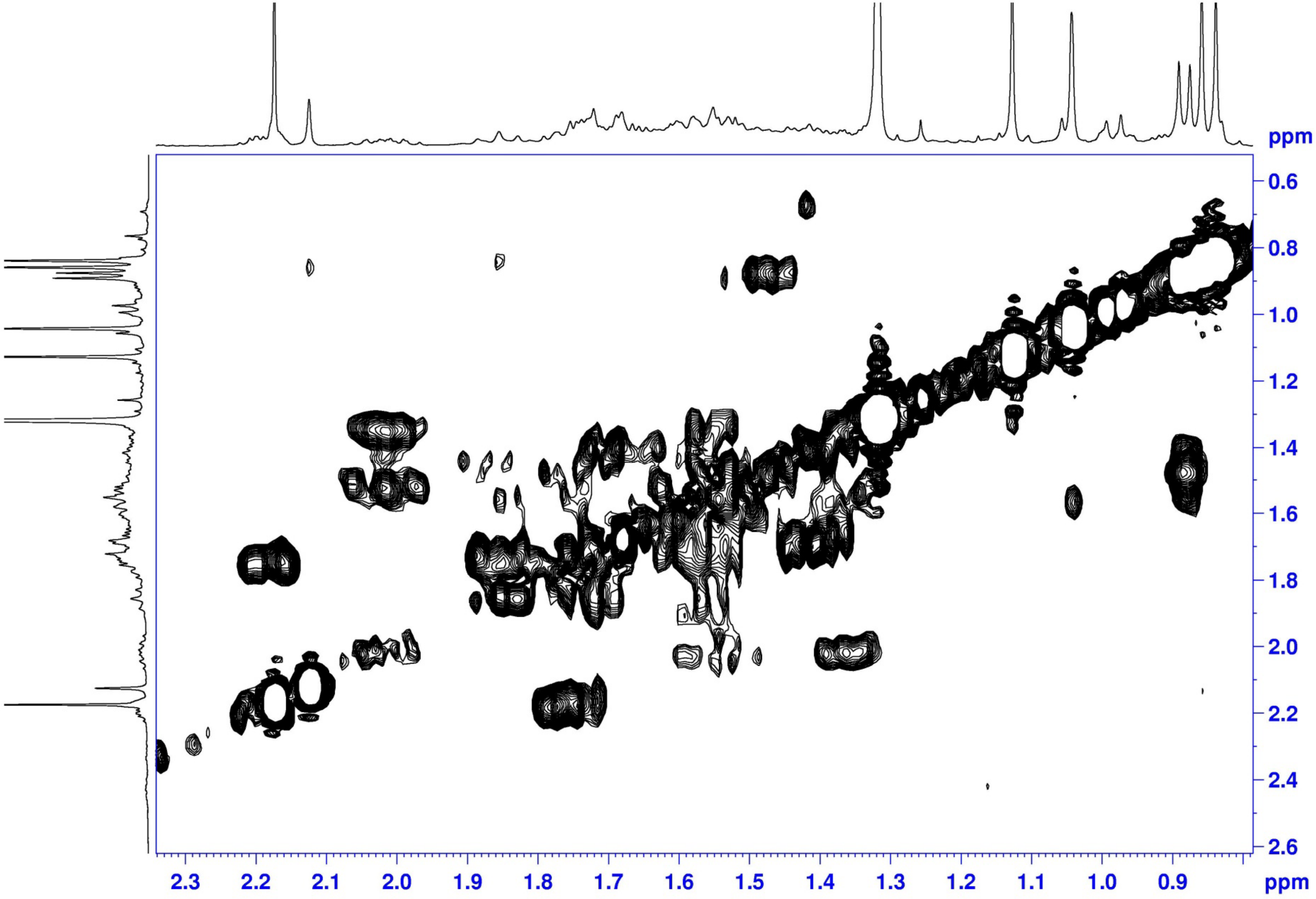

^{1}H-^{1}H COSY (400 MHz) spectrum of compound **8** in $CDCl_3$

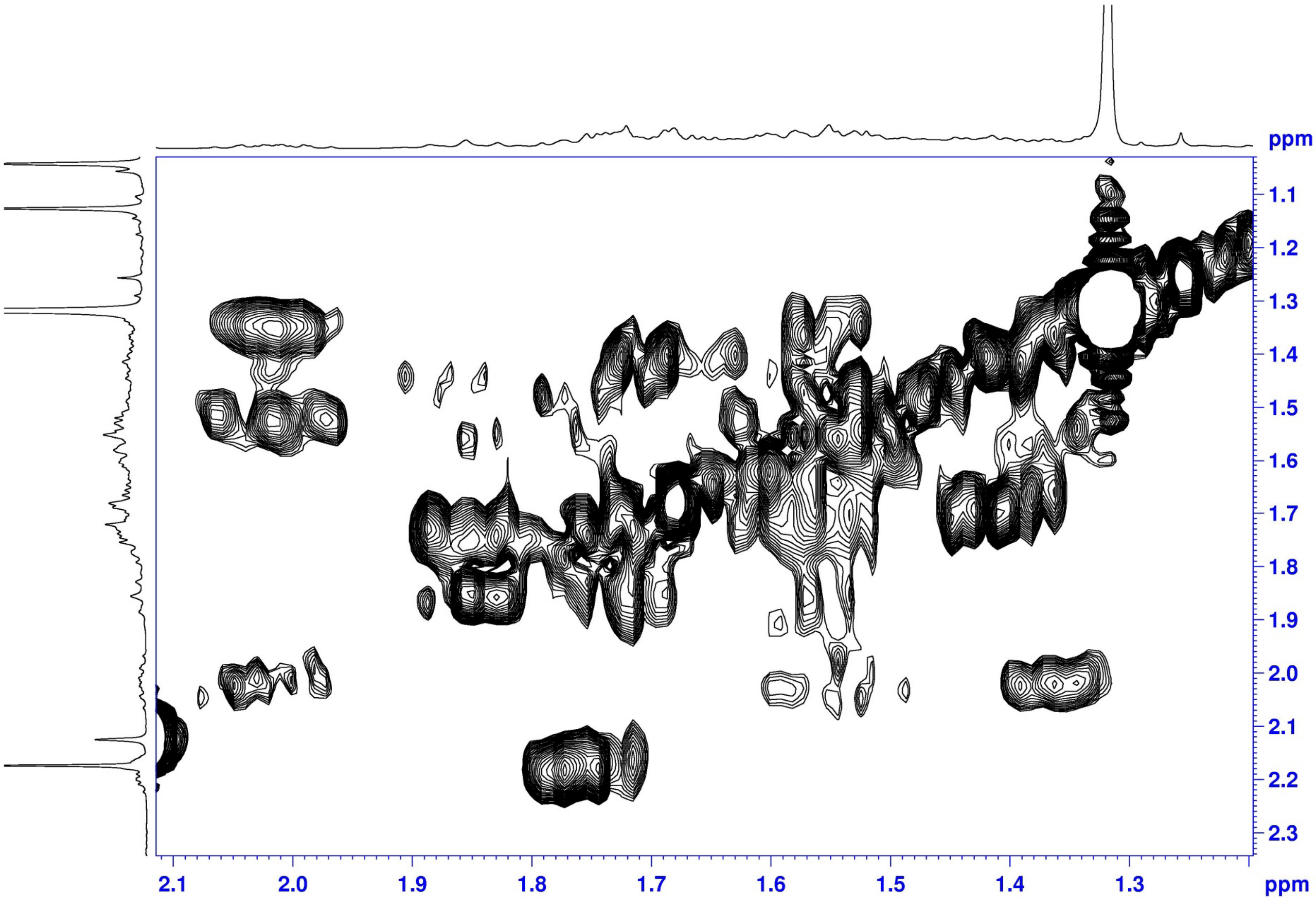

HSQC (400 MHz) spectrum of compound **8** in $CDCl_3$

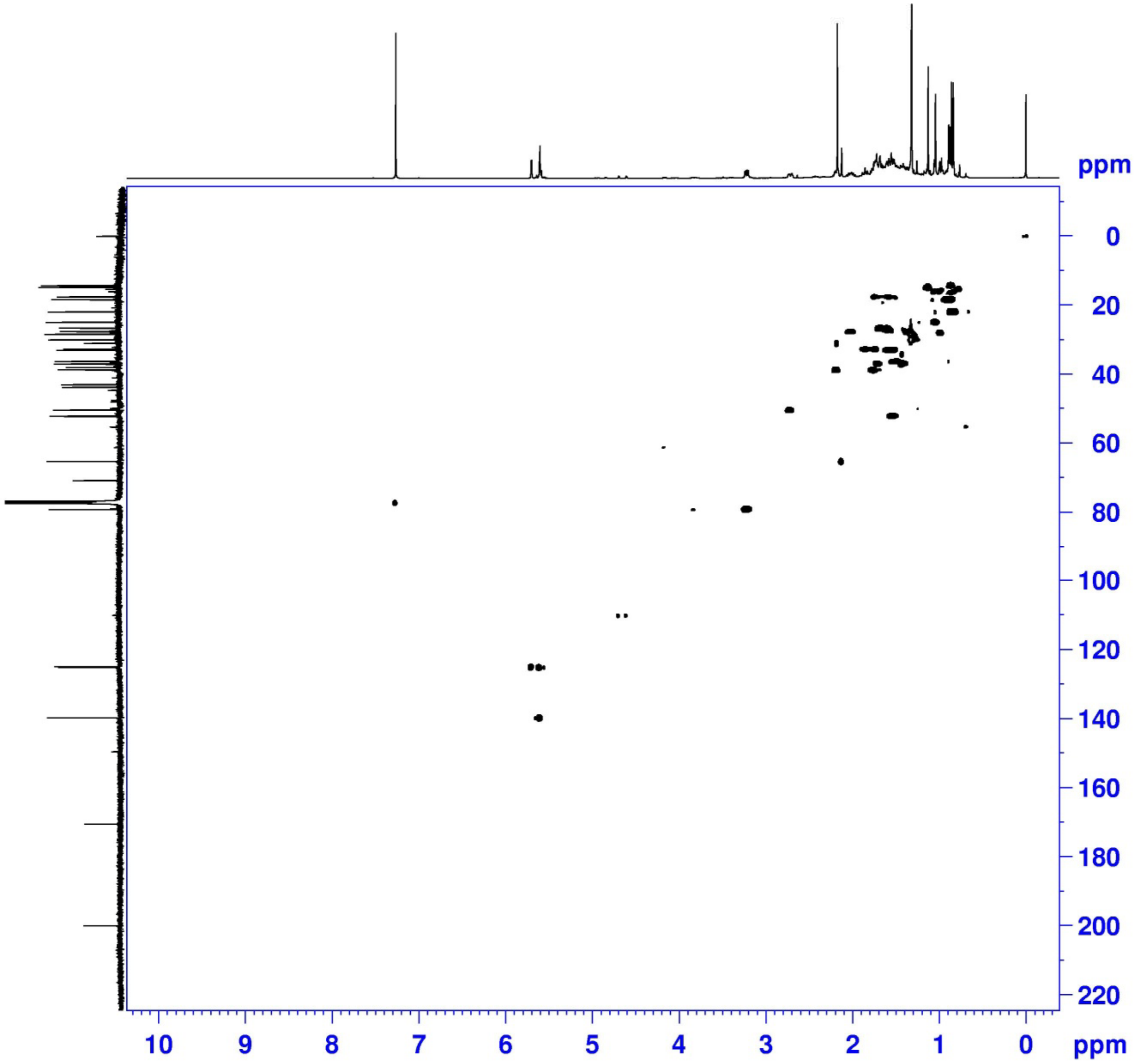


```
NAME           EA3-44-3-1
EXPNO                   5
PROCNO                  1
Date_            20171109
Time                17.20
INSTRUM             spect
PROBHD   5 mm CPPBBO BB
PULPROG       hsqcetgpsi2
TD                   1024
SOLVENT             CDCl3
NS                     16
DS                     16
SWH              4302.926 Hz
FIDRES           4.202076 Hz
AQ              0.1190388 sec
RG                  208.5
DW                116.200 usec
DE                  10.00 usec
TE                  297.0 K
CNST2         145.0000000
D0             0.00000300 sec
D1             1.46497905 sec
D4             0.00172414 sec
D11            0.03000000 sec
D16            0.00020000 sec
D24            0.00086207 sec
IN0            0.00002080 sec
ZGOPTNS

======== CHANNEL f1 ========
SFO1          400.1320007 MHz
NUC1                   1H
P1                  11.50 usec
P2                  23.00 usec
P28                  0.00 usec
ND0                     2
TD                    256
SFO1             100.6233 MHz
FIDRES          93.900238 Hz
SW                238.896 ppm
FnMODE       Echo-Antiecho
SI                   1024
SF            400.1300033 MHz
WDW                 QSINE
SSB                     2
LB                   0.00 Hz
GB                      0
PC                   1.40
SI                   1024
MC2         echo-antiecho
SF            100.6127549 MHz
WDW                 QSINE
```

HSQC (400 MHz) spectrum of compound **8** in $CDCl_3$

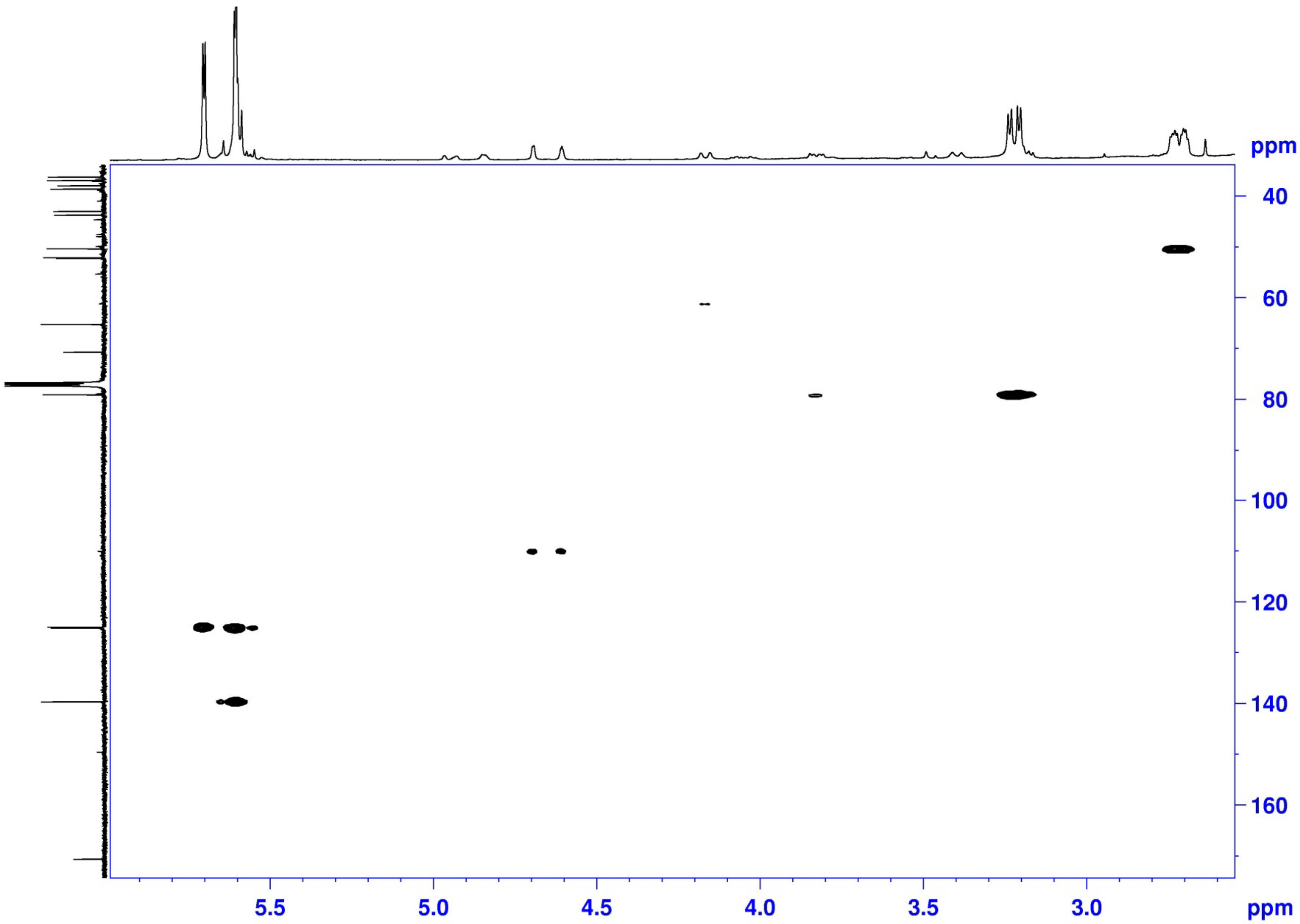

HSQC (400 MHz) spectrum of compound **8** in $CDCl_3$

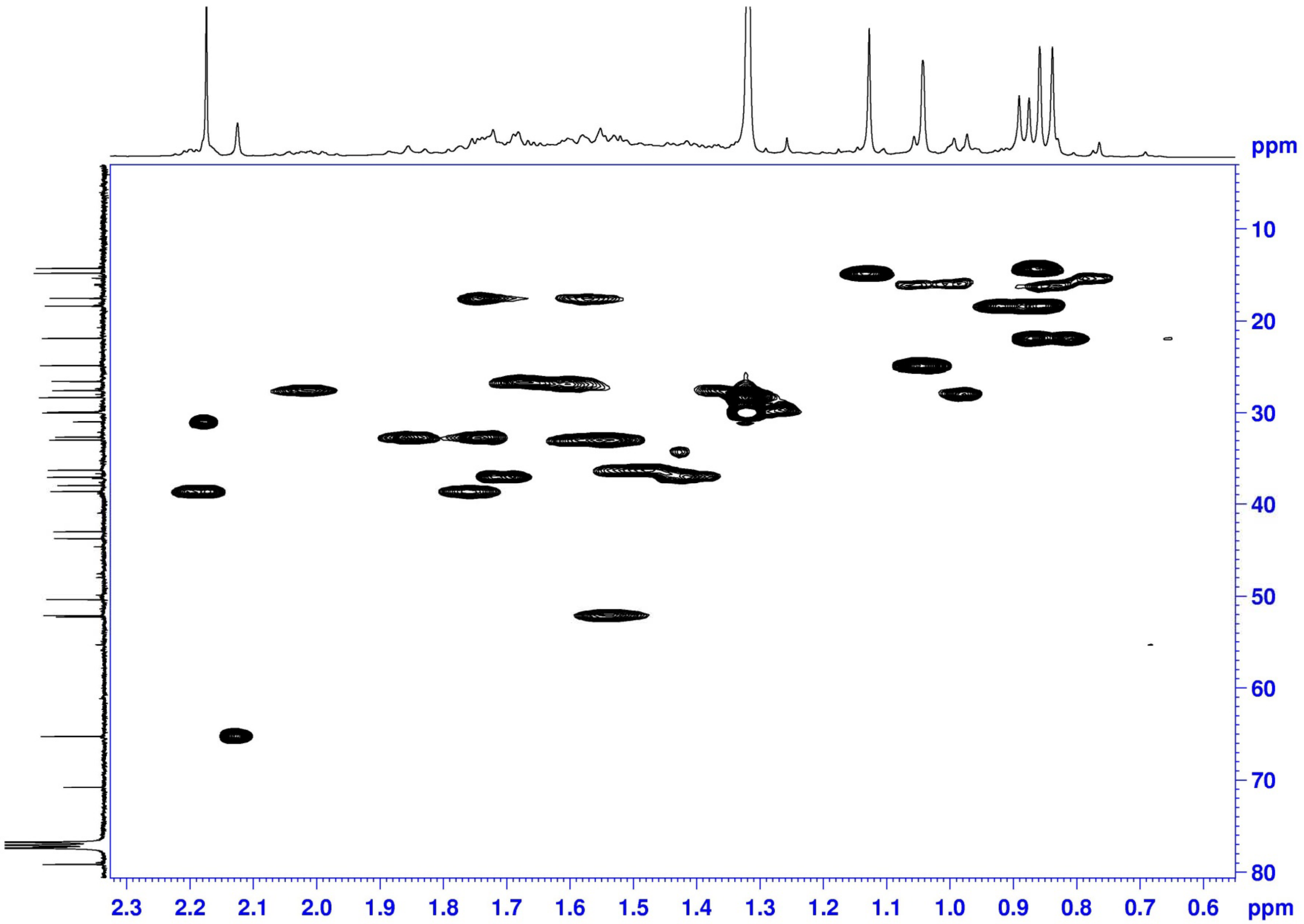

HSQC (400 MHz) spectrum of compound **8** in $CDCl_3$

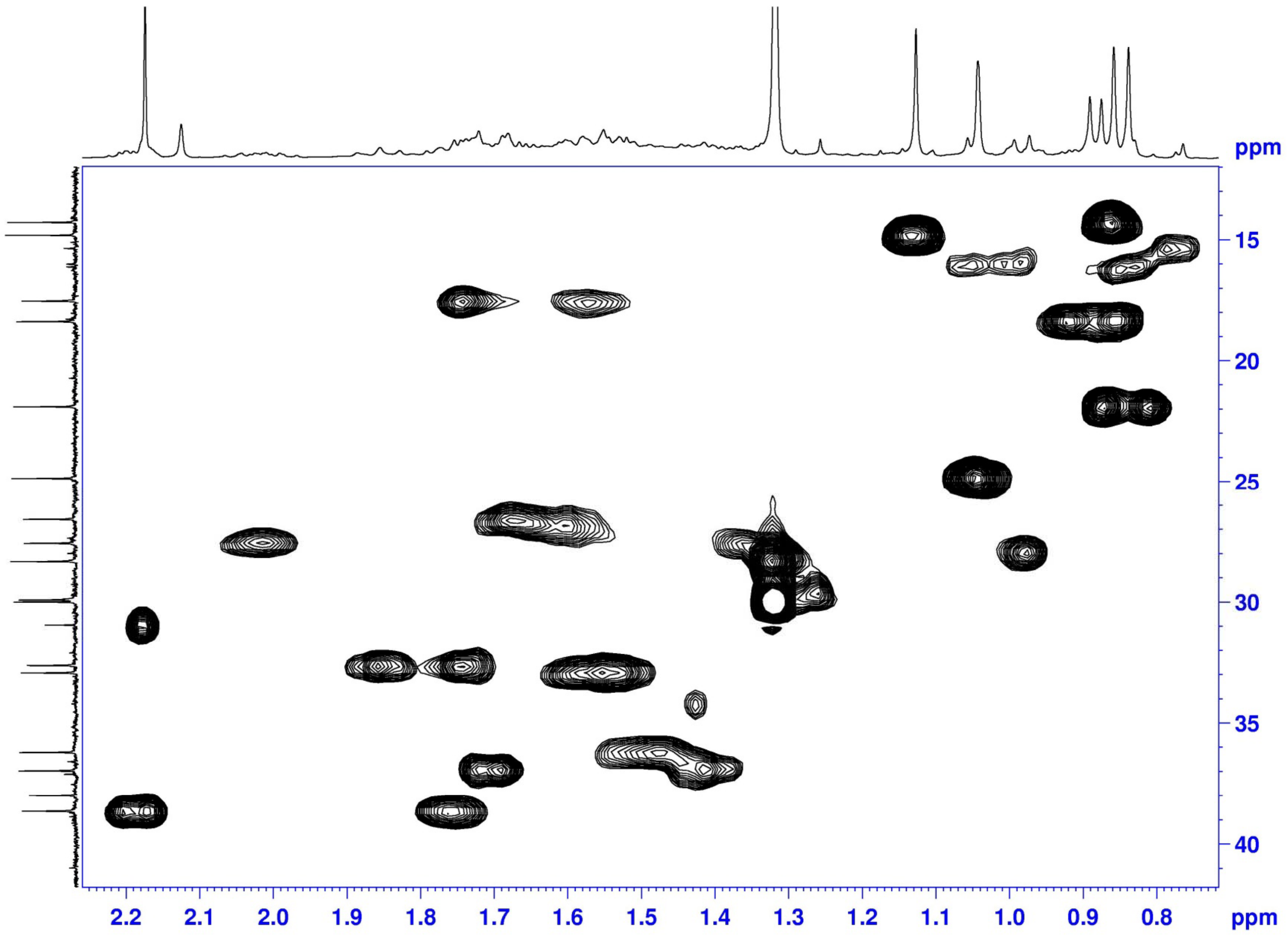

HMBC (400 MHz) spectrum of compound **8** in $CDCl_3$

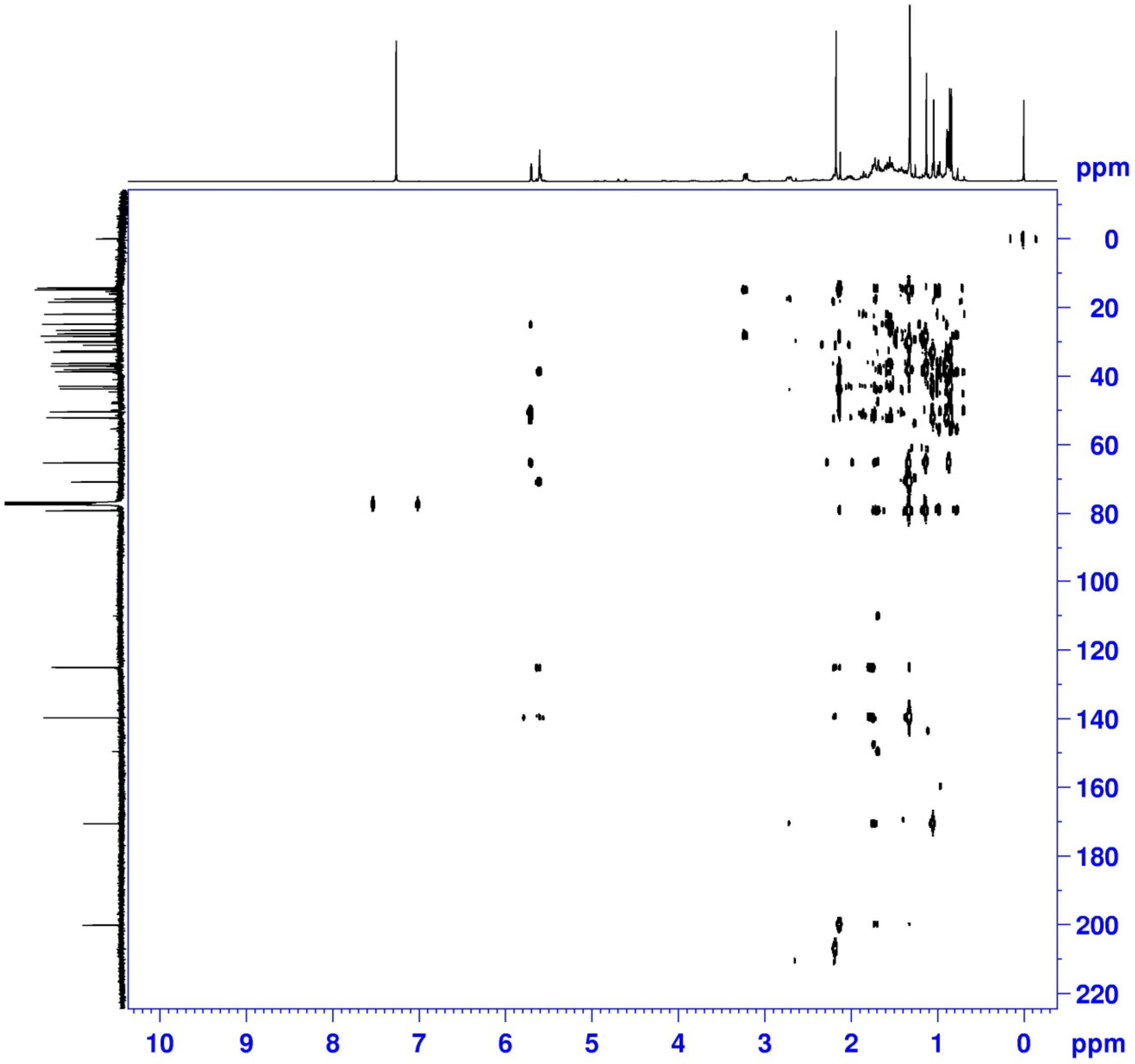


```
NAME            EA3-44-3-1
EXPNO                    6
PROCNO                   1
Date_             20171109
Time                 19.11
INSTRUM              spect
PROBHD   5 mm CPPBBO BB
PULPROG      hmbcgplpndqf
TD                    4096
SOLVENT              CDCl3
NS                      32
DS                      16
SWH              5197.505 Hz
FIDRES           1.268922 Hz
AQ              0.3940852 sec
RG                   208.5
DW                  96.200 usec
DE                   10.00 usec
TE                   297.0 K
CNST2          145.0000000
CNST13          10.0000000
D0              0.00000300 sec
D1              1.50000000 sec
D2              0.00344828 sec
D6              0.05000000 sec
D16             0.00020000 sec
IN0             0.00002080 sec

======== CHANNEL f1 ========
SFO1          400.1323208 MHz
NUC1                    1H
P1                   11.50 usec
P2                   23.00 usec
ND0                      2
TD                     128
SFO1           100.6233 MHz
FIDRES       187.800476 Hz
SW              238.896 ppm
FnMODE                  QF
SI                    2048
SF            400.1300051 MHz
WDW                   SINE
SSB                      0
LB                    0.00 Hz
GB                       0
PC                    1.40
SI                    1024
MC2                     QF
SF            100.6127717 MHz
WDW                   SINE
SSB                      0
LB                    0.00 Hz
[illegible]
```

HMBC (400 MHz) spectrum of compound **8** in $CDCl_3$

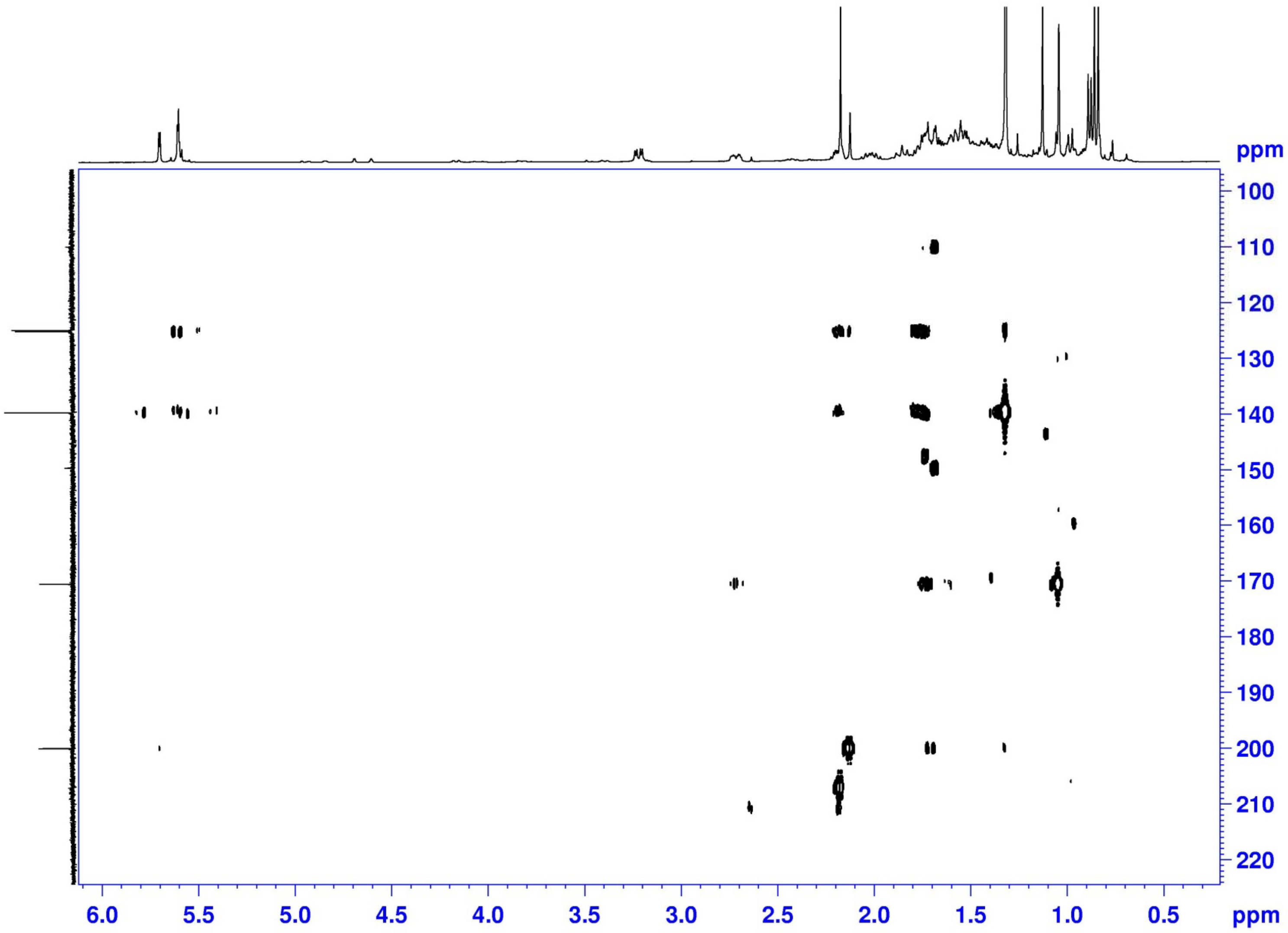

HMBC (400 MHz) spectrum of compound **8** in $CDCl_3$

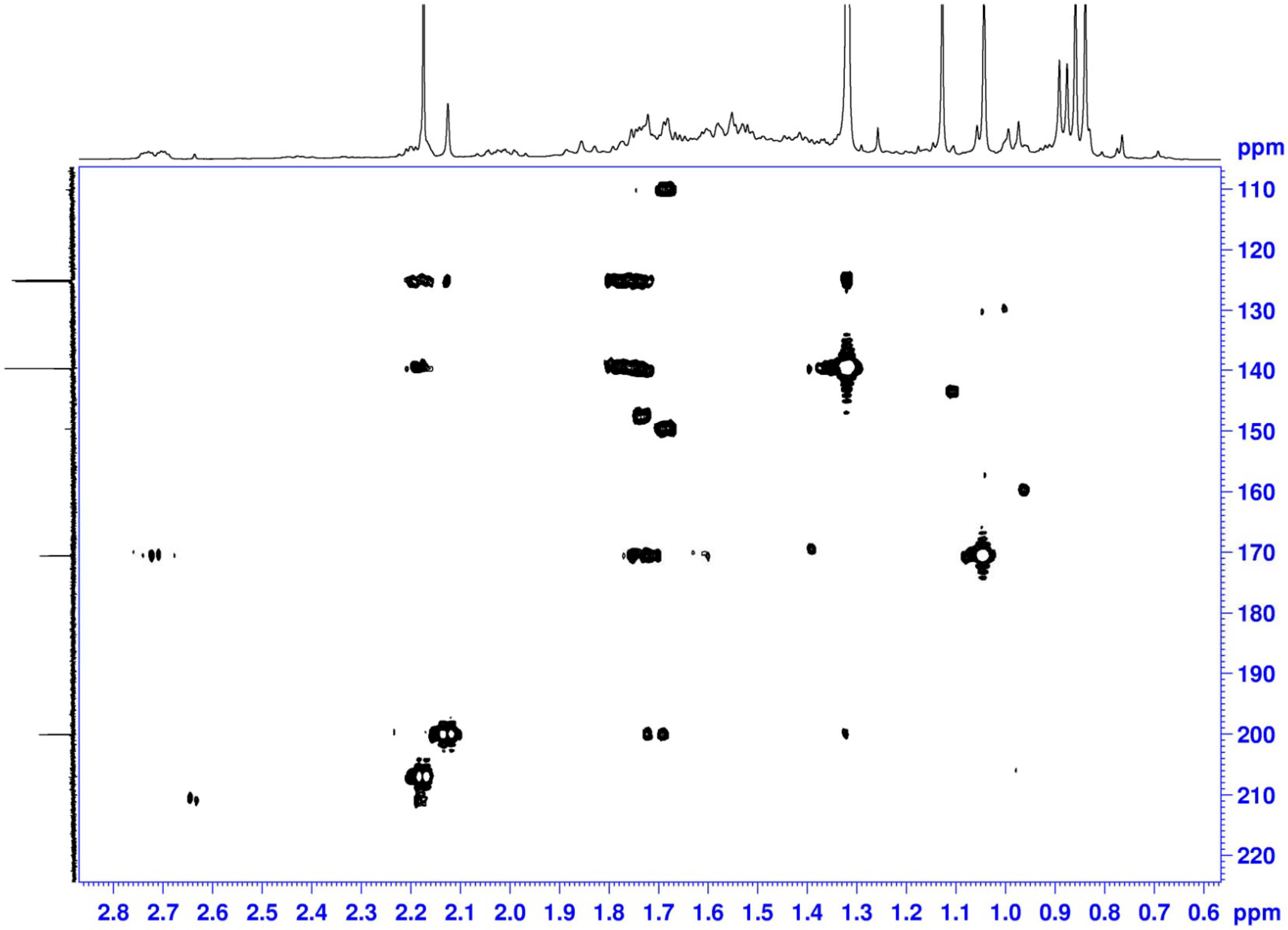

HMBC (400 MHz) spectrum of compound **8** in $CDCl_3$

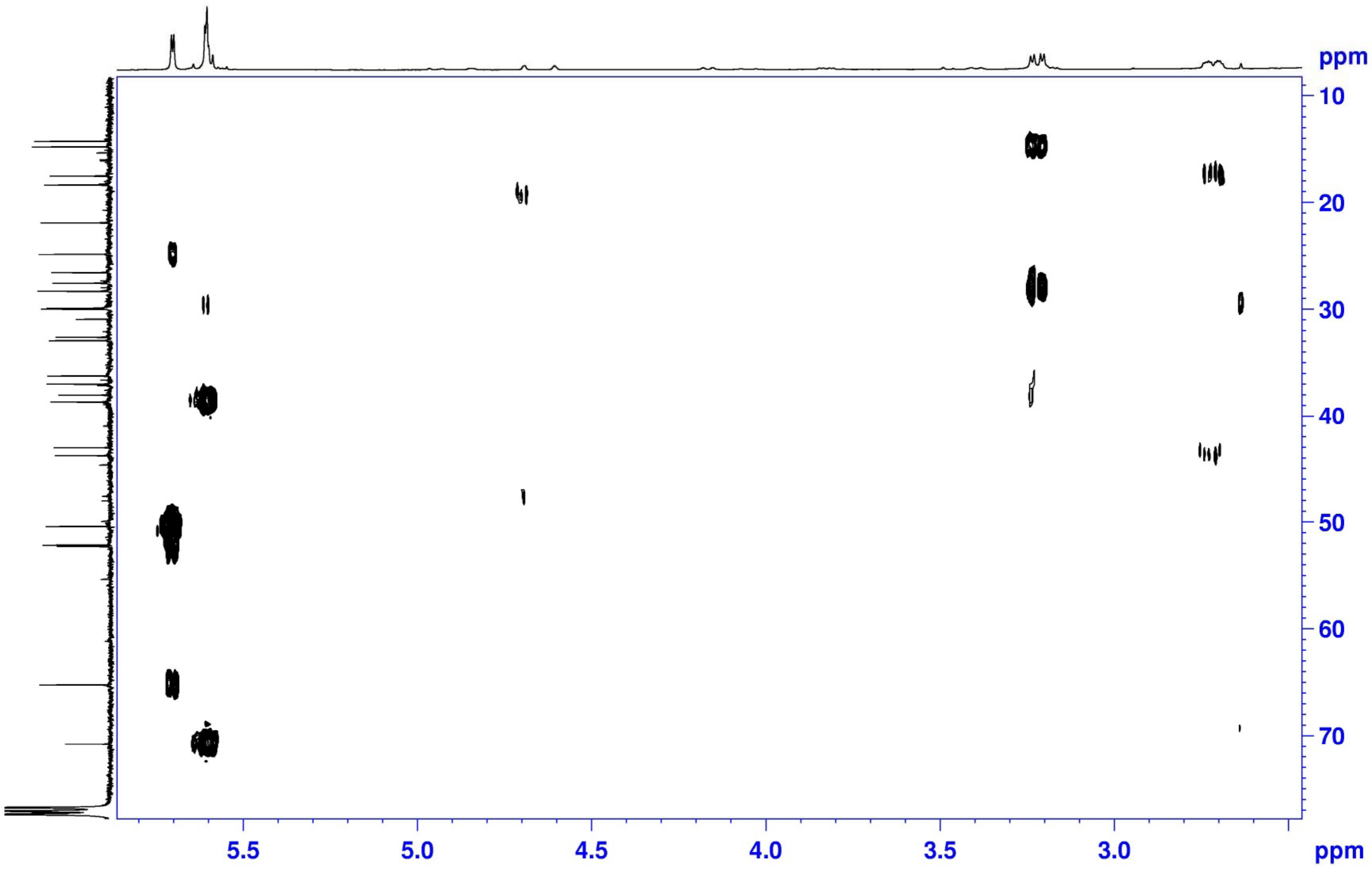

HMBC (400 MHz) spectrum of compound **8** in $CDCl_3$

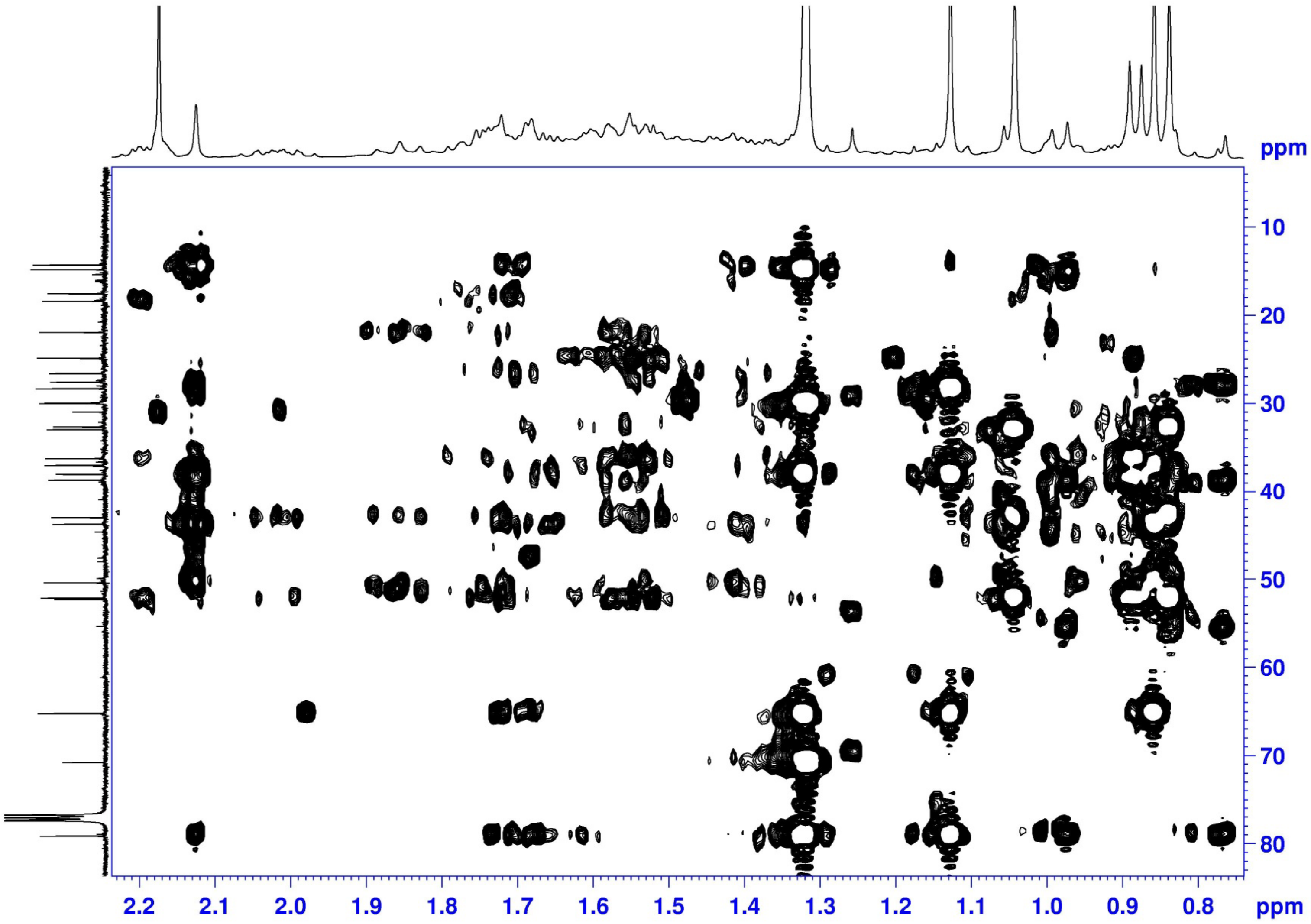

HMBC (400 MHz) spectrum of compound **8** in $CDCl_3$

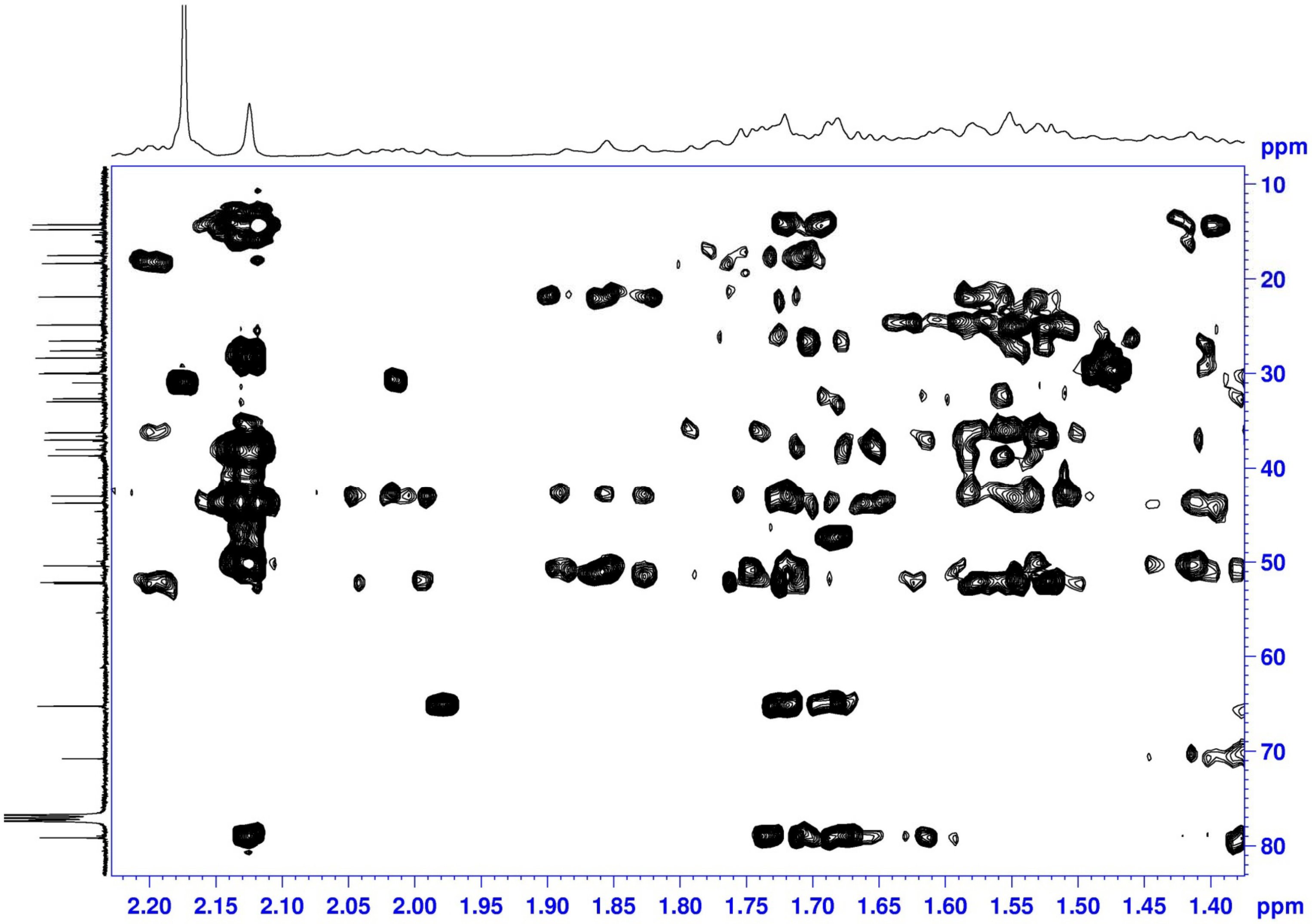

HMBC (400 MHz) spectrum of compound **8** in $CDCl_3$

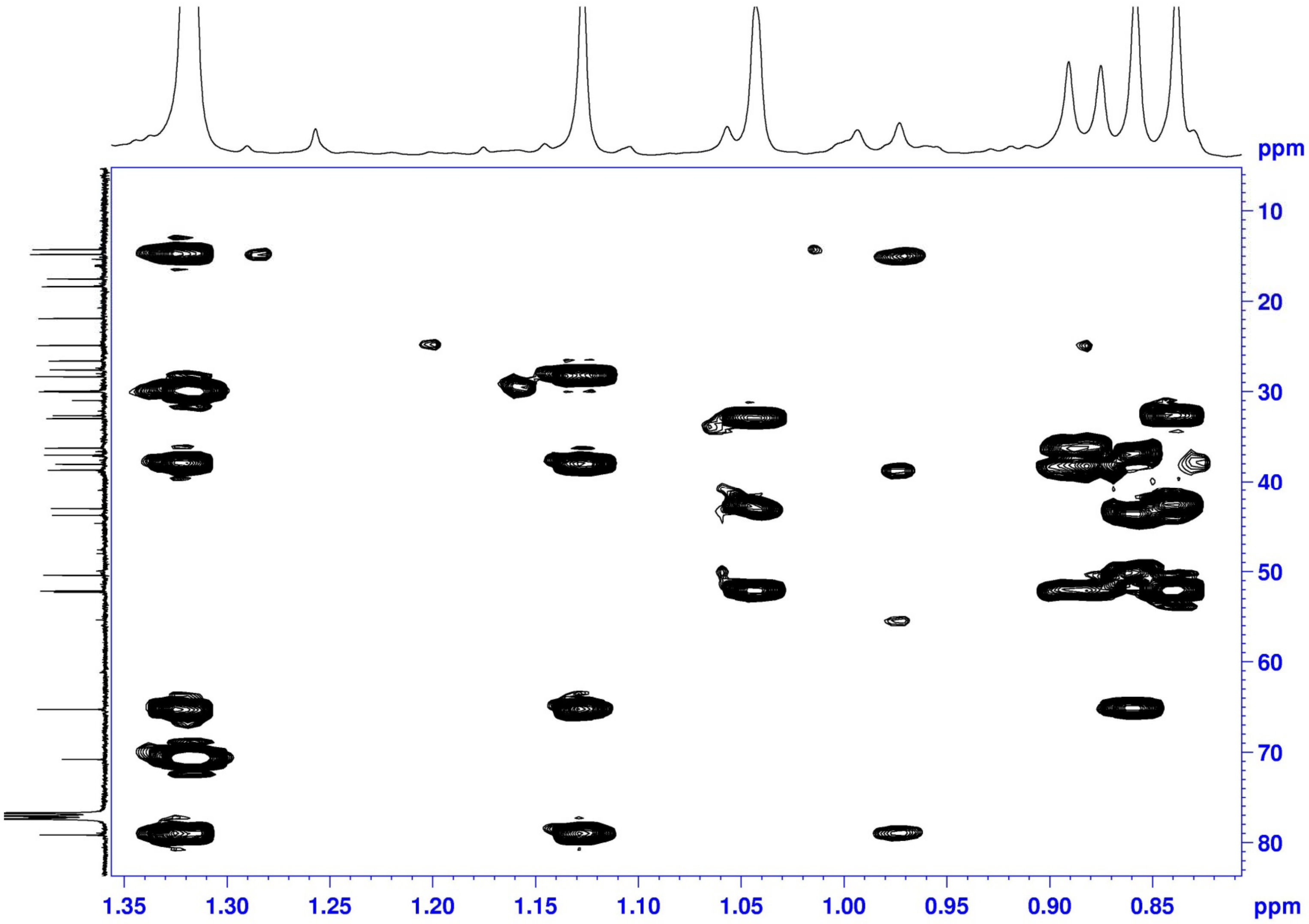

NOESY (400 MHz) spectrum of compound **8** in $CDCl_3$

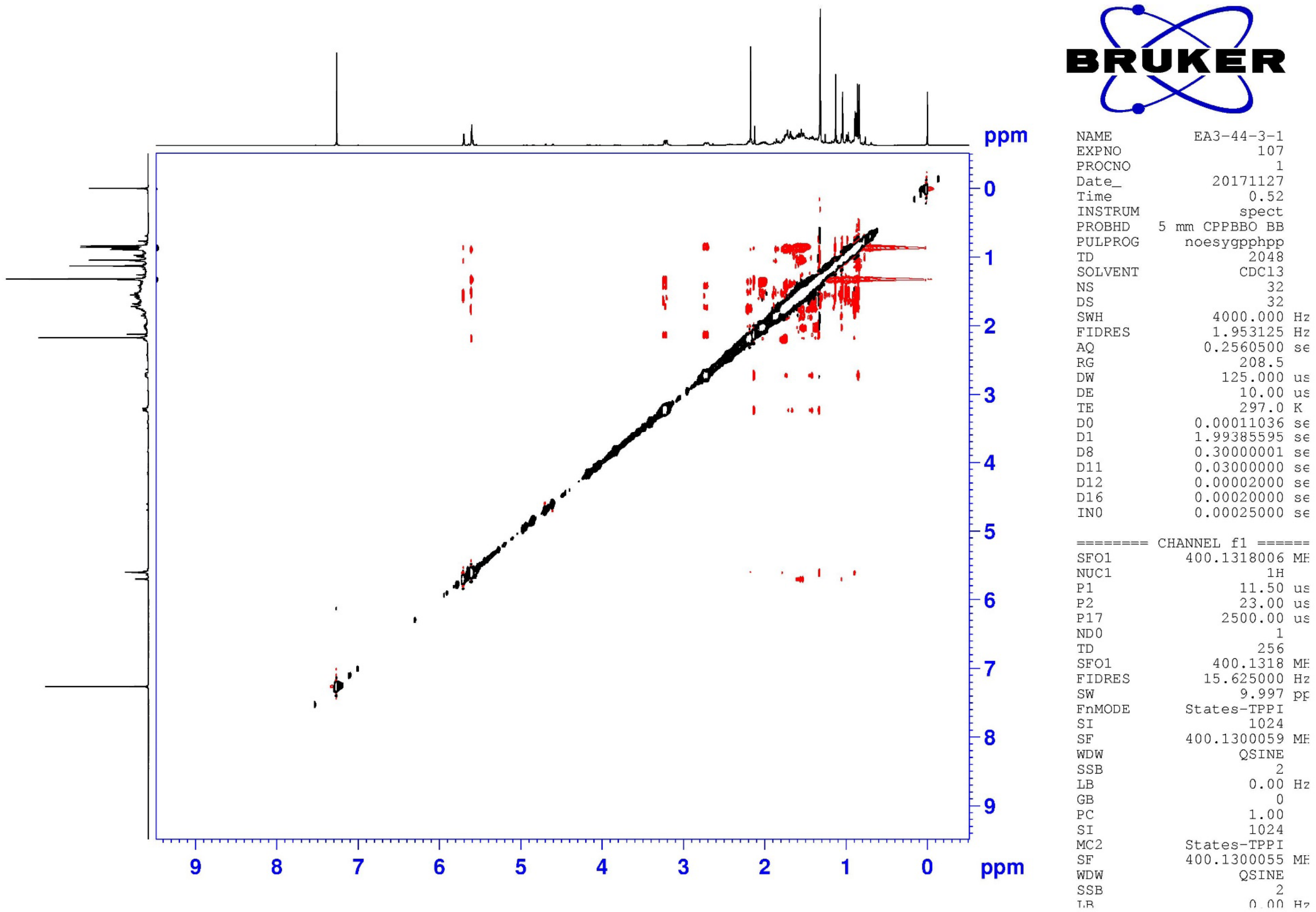

NOESY (400 MHz) spectrum of compound **8** in $CDCl_3$

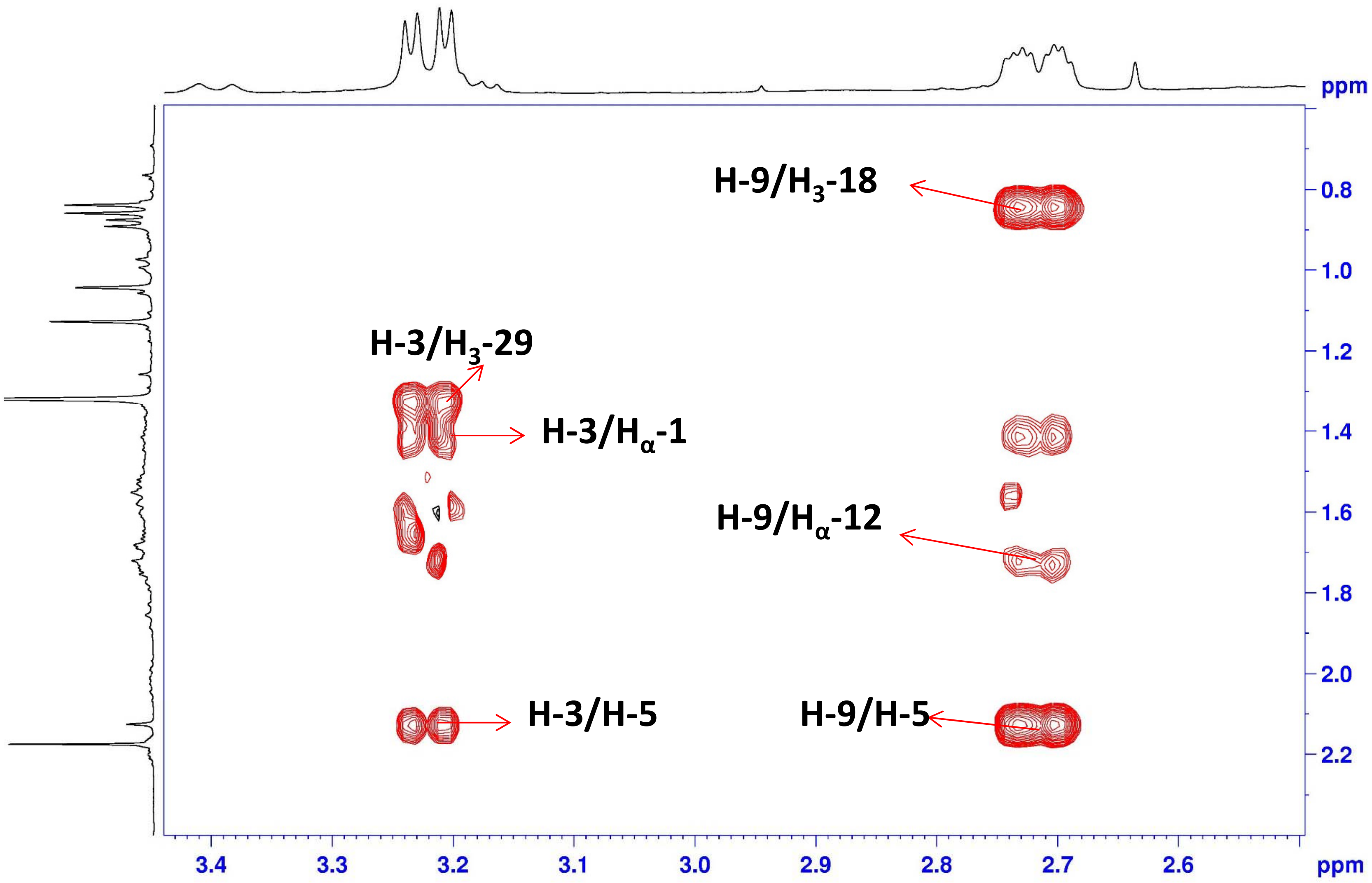

NOESY (400 MHz) spectrum of compound **8** in $CDCl_3$

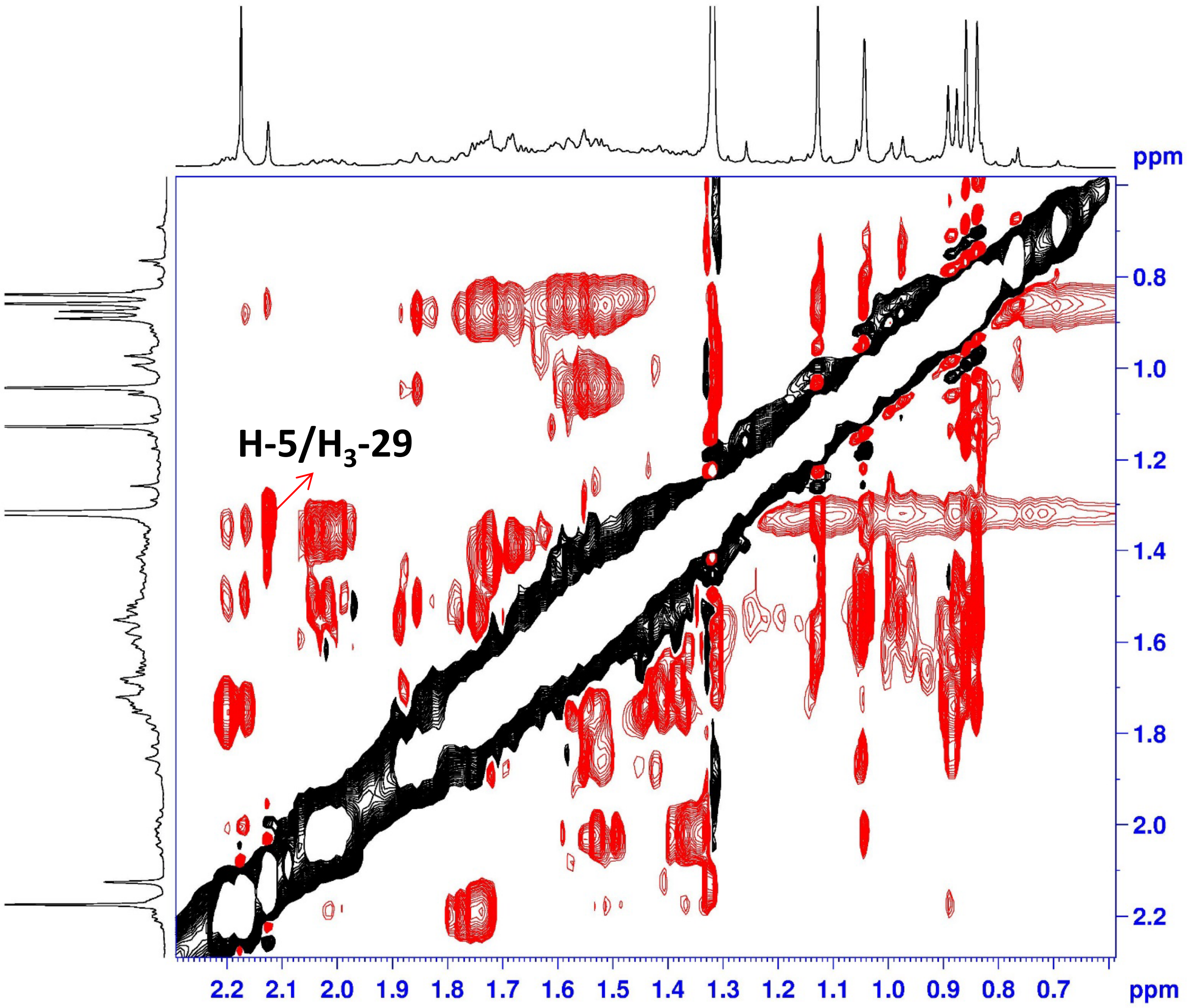

NOESY (400 MHz) spectrum of compound **8** in $CDCl_3$

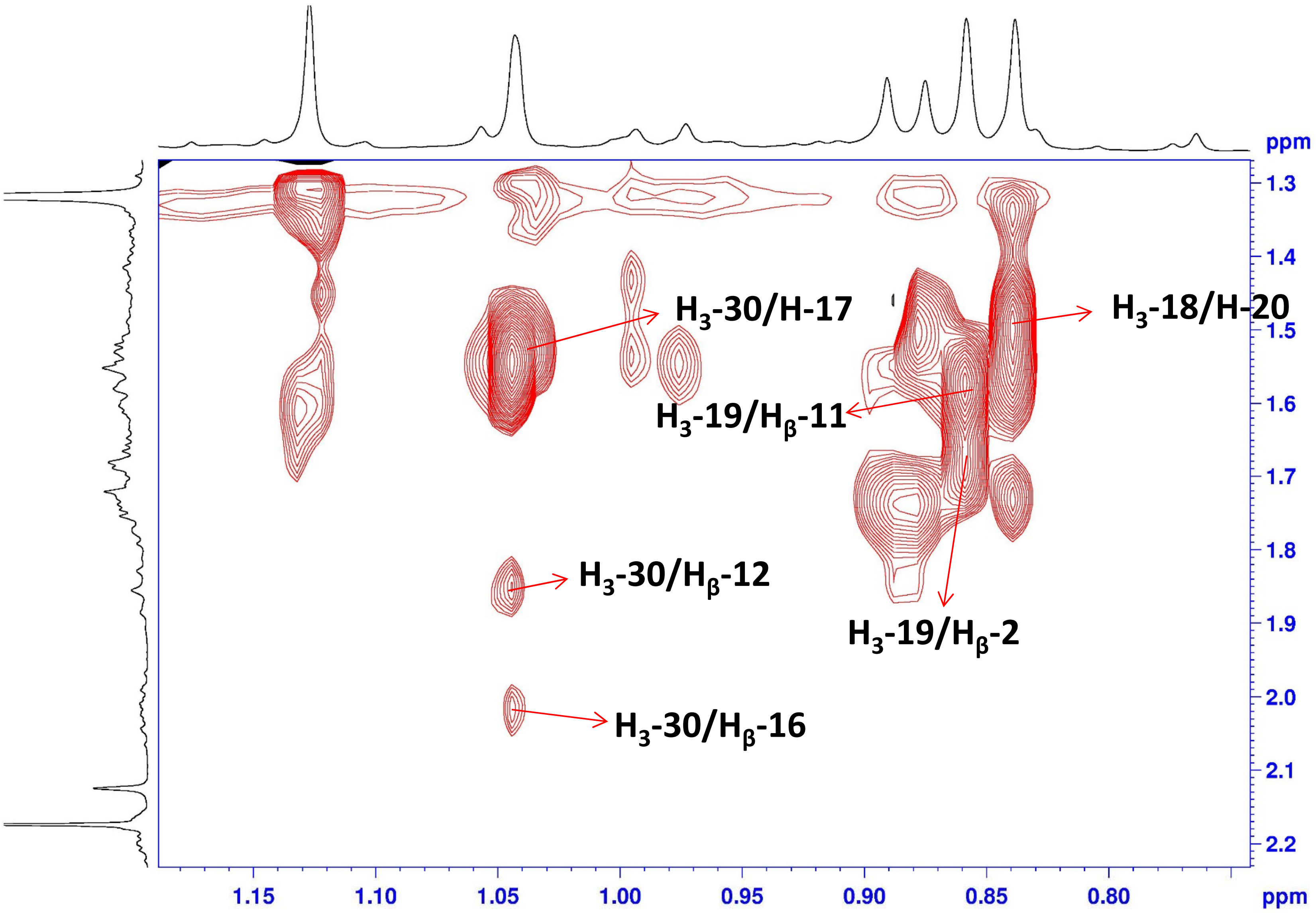

NOESY (400 MHz) spectrum of compound **8** in $CDCl_3$

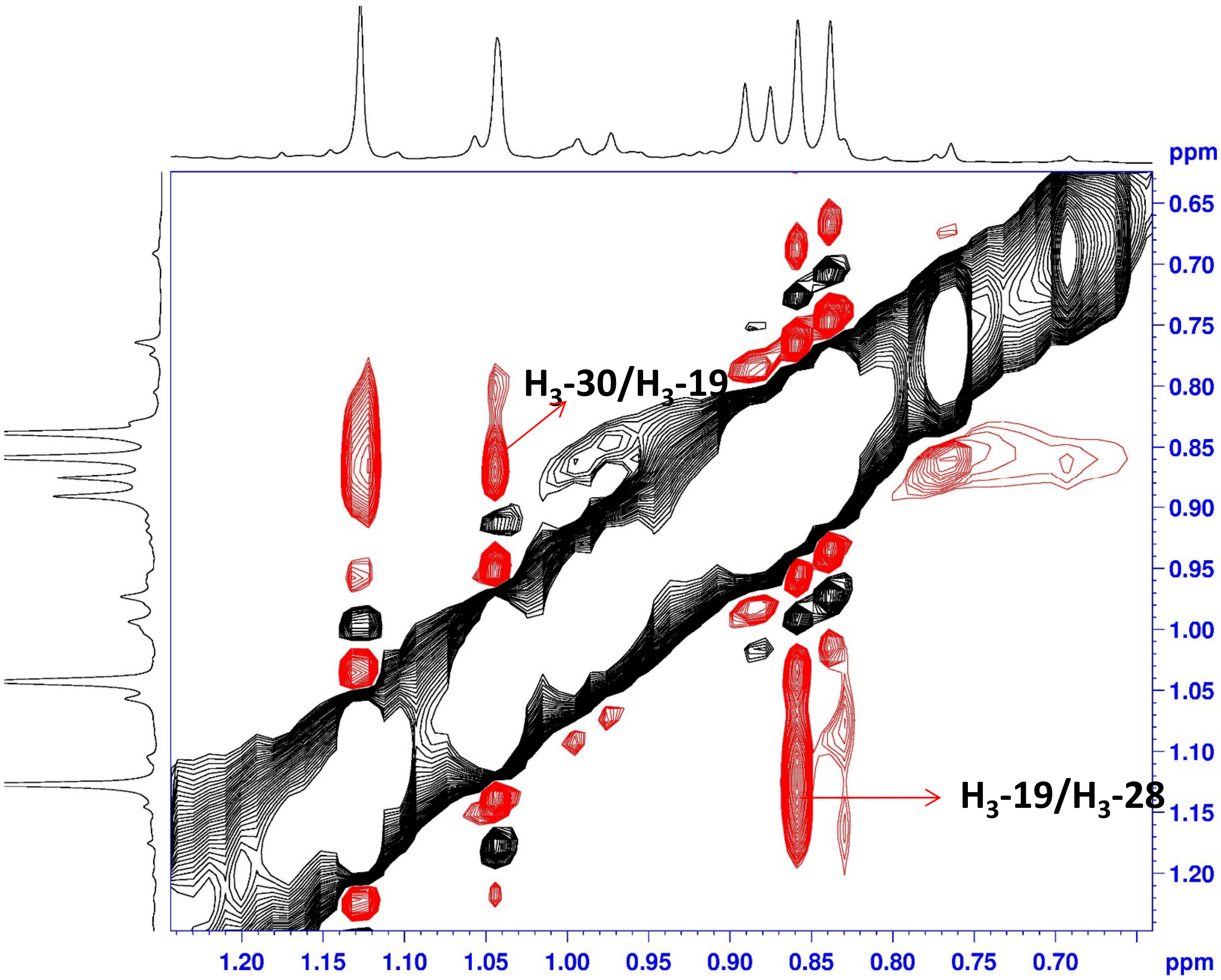

HR-ESIMS for compound **9**

Generic Display Report

**Analysis Info**		Acquisition Date	8/21/2017 4:14:53 PM
Analysis Name	D:\Data\MS\data\201708\jiangzhongping_EA2-27_neg_6_01_3384.d		
Method	LC_Direct Infusion_neg_100-1000mz.m	Operator	SCSIO
Sample Name	jiangzhongping_EA2-27_neg	Instrument	maXis
Comment			

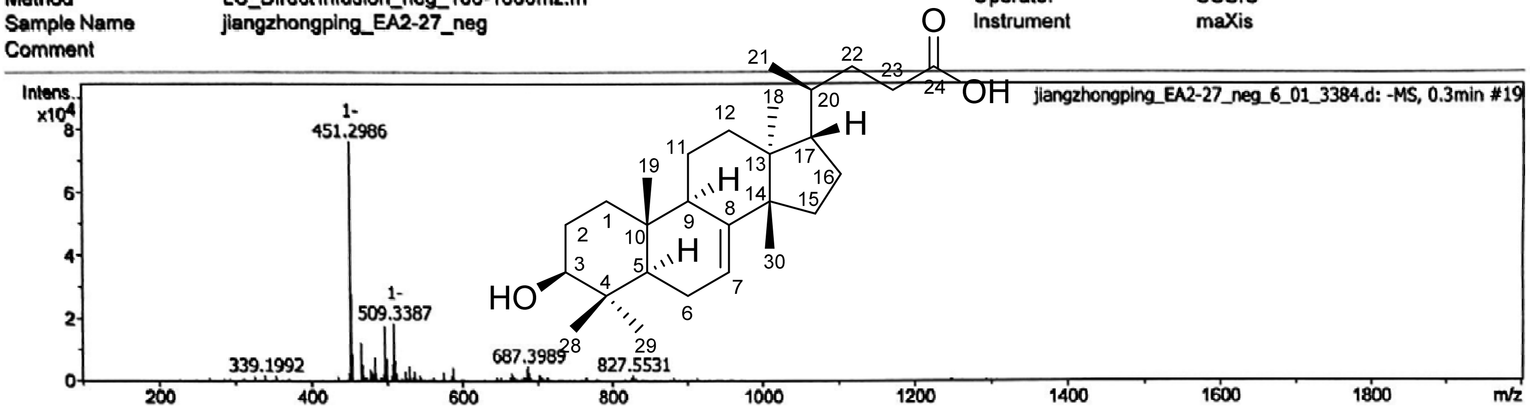

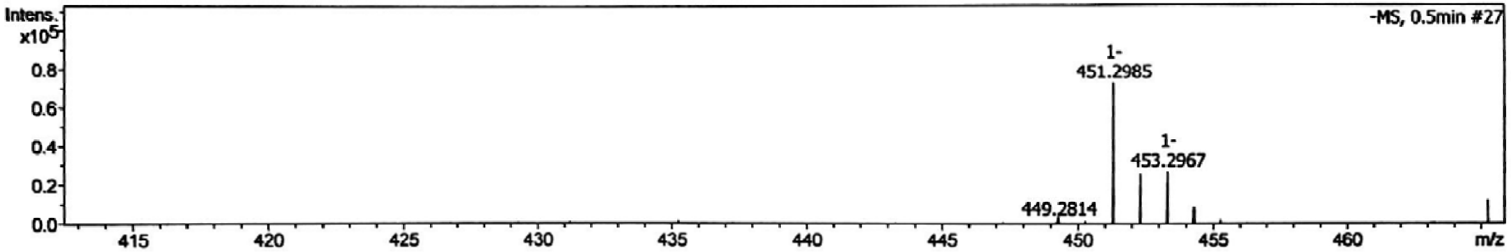

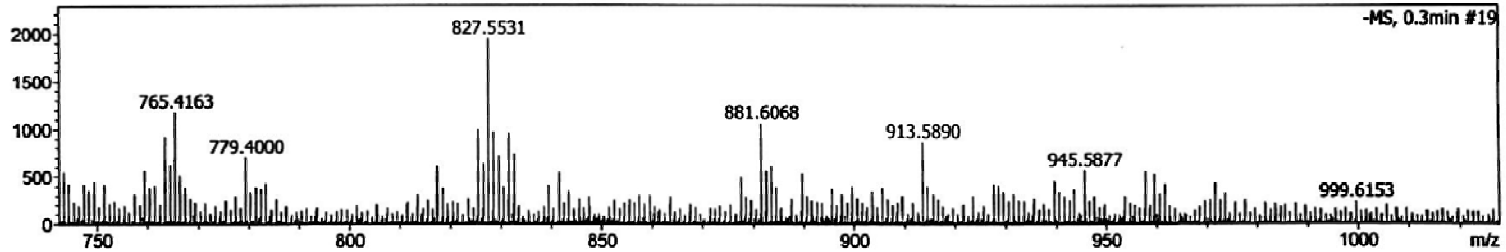

Bruker Compass DataAnalysis 4.1 printed: 8/21/2017 4:21:02 PM by: SCSIO Page 1 of 1

HR-ESIMS for compound **9**

Mass Spectrum SmartFormula Report

Analysis Info

Analysis Name	D:\Data\MS\data\201708\jiangzhongping_EA2-27_neg_6_01_3384.d	Acquisition Date	8/21/2017 4:14:53 PM
Method	LC_Direct Infusion_neg_100-1000mz.m	Operator	SCSIO
Sample Name	jiangzhongping_EA2-27_neg	Instrument	maXis 255552.00029
Comment			

Acquisition Parameter

Source Type	ESI	Ion Polarity	Negative	Set Nebulizer	0.4 Bar
Focus	Active	Set Capillary	4000 V	Set Dry Heater	180 °C
Scan Begin	100 m/z	Set End Plate Offset	-500 V	Set Dry Gas	4.0 l/min
Scan End	2000 m/z	Set Charging Voltage	0 V	Set Divert Valve	Waste
		Set Corona	0 nA	Set APCI Heater	0 °C

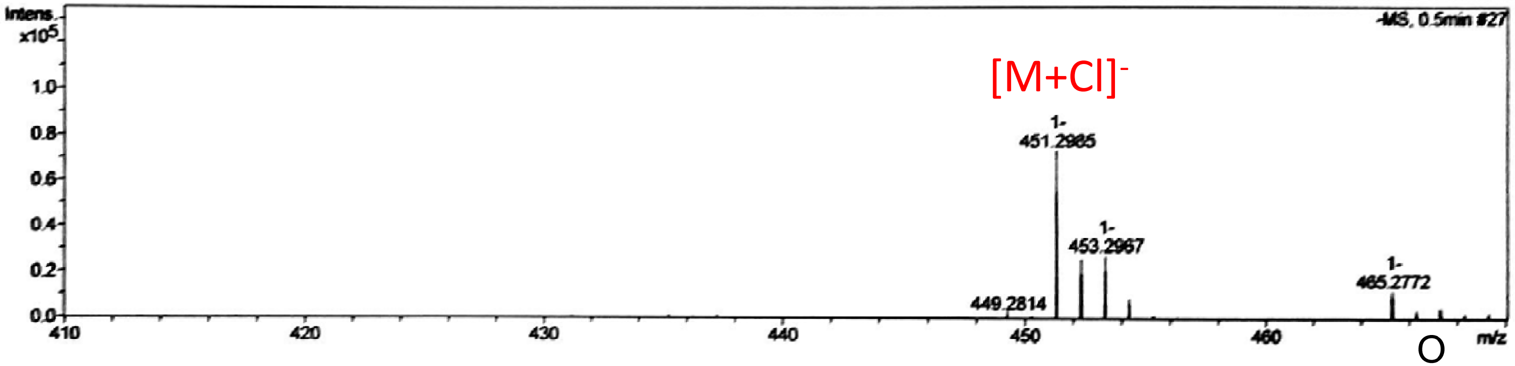

Meas. m/z	#	Ion Formula	Score	m/z	err [ppm]	err [mDa]	mSigma	rdb	e⁻ Conf	N-Rule
451.298467	1	C27H44ClO3	100.00	451.298447	-0.0	-0.0	25.6	5.5	even	ok

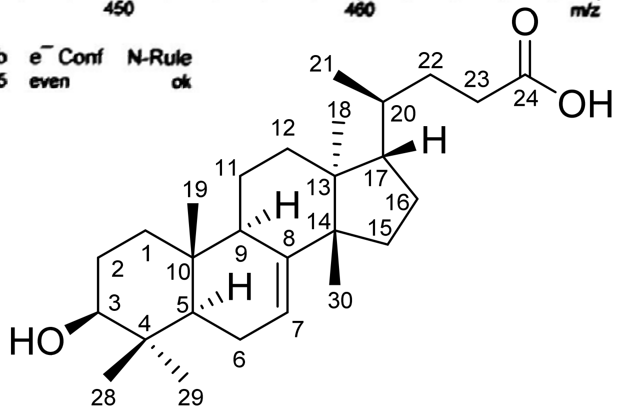

^{1}H NMR (400 MHz) spectrum of compound **9** in $CDCl_3$

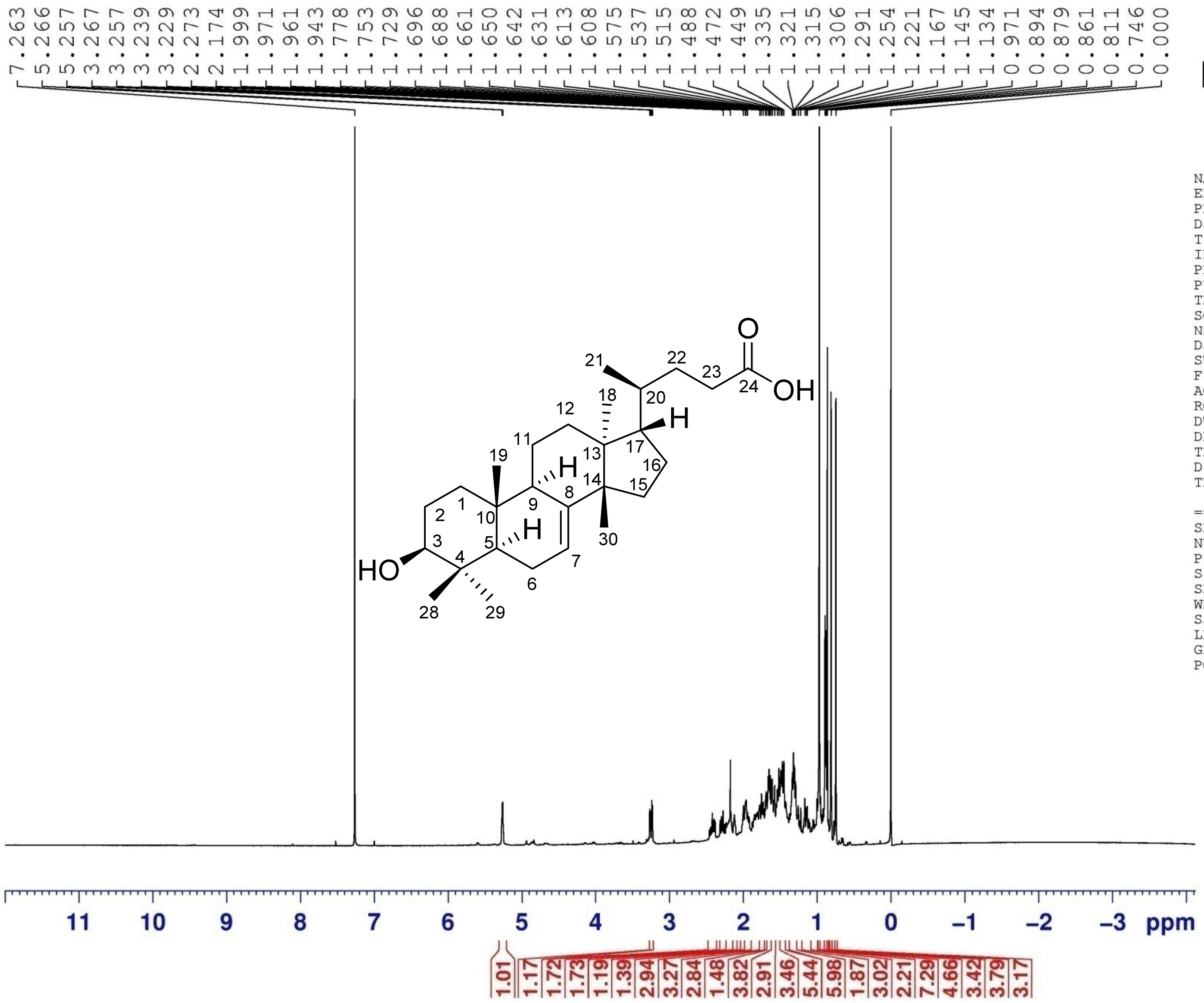

NAME	EA2-27(EA2-33-3)
EXPNO	1
PROCNO	1
Date_	20170613
Time	4.00
INSTRUM	spect
PROBHD	5 mm CPPBBO BB
PULPROG	zg30
TD	65536
SOLVENT	CDCl3
NS	16
DS	2
SWH	8223.685 Hz
FIDRES	0.125483 Hz
AQ	3.9846387 sec
RG	208.5
DW	60.800 usec
DE	10.00 usec
TE	297.0 K
D1	1.00000000 sec
TD0	1
======== CHANNEL f1 ========	
SFO1	400.1324710 MHz
NUC1	1H
P1	11.50 usec
SI	65536
SF	400.1300088 MHz
WDW	EM
SSB	0
LB	0.30 Hz
GB	0
PC	1.00

^{1}H NMR (400 MHz) spectrum of compound **9** in $CDCl_3$

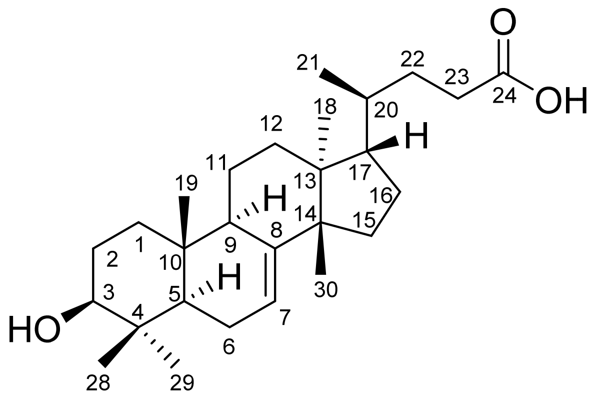

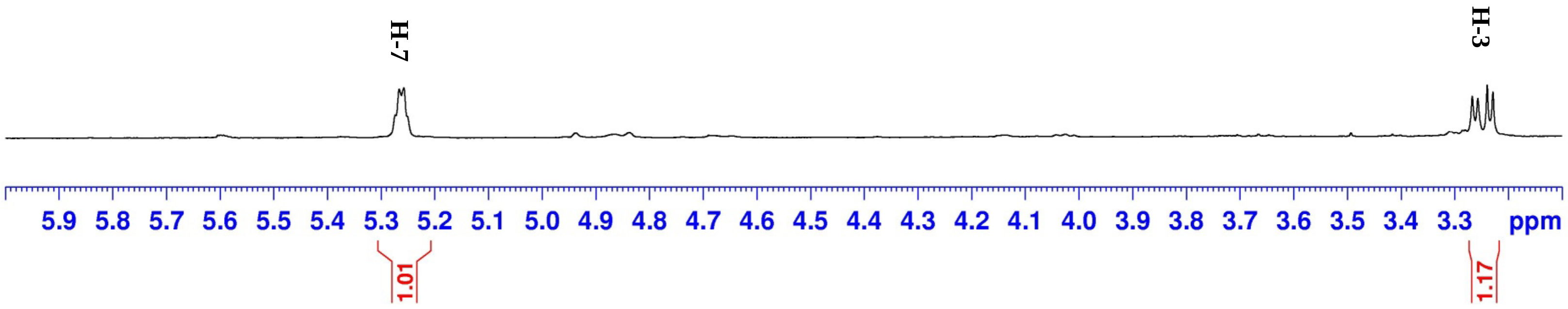

^{1}H NMR (400 MHz) spectrum of compound **9** in $CDCl_3$

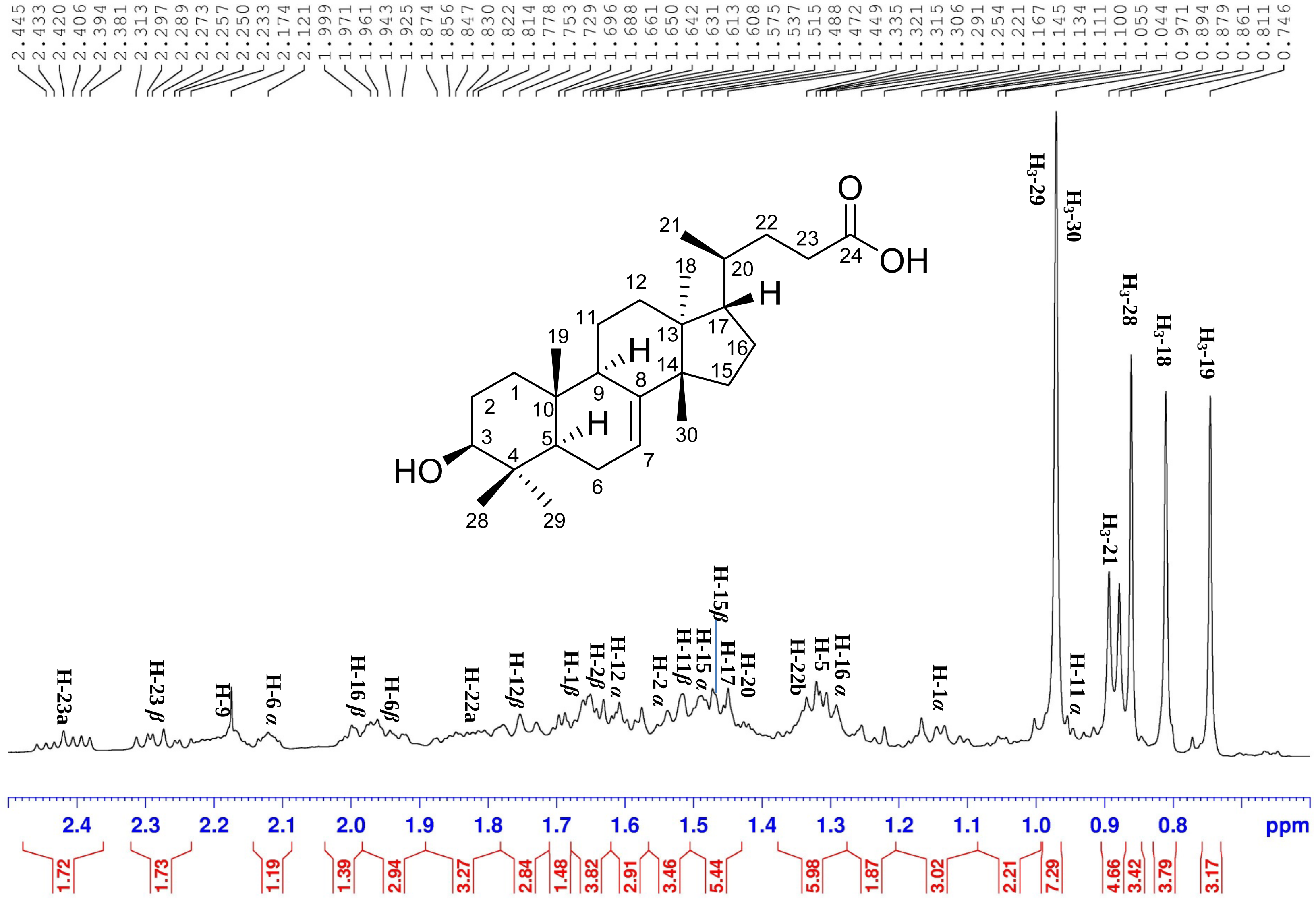

^{13}C NMR (100 MHz) spectrum of compound **9** in $CDCl_3$

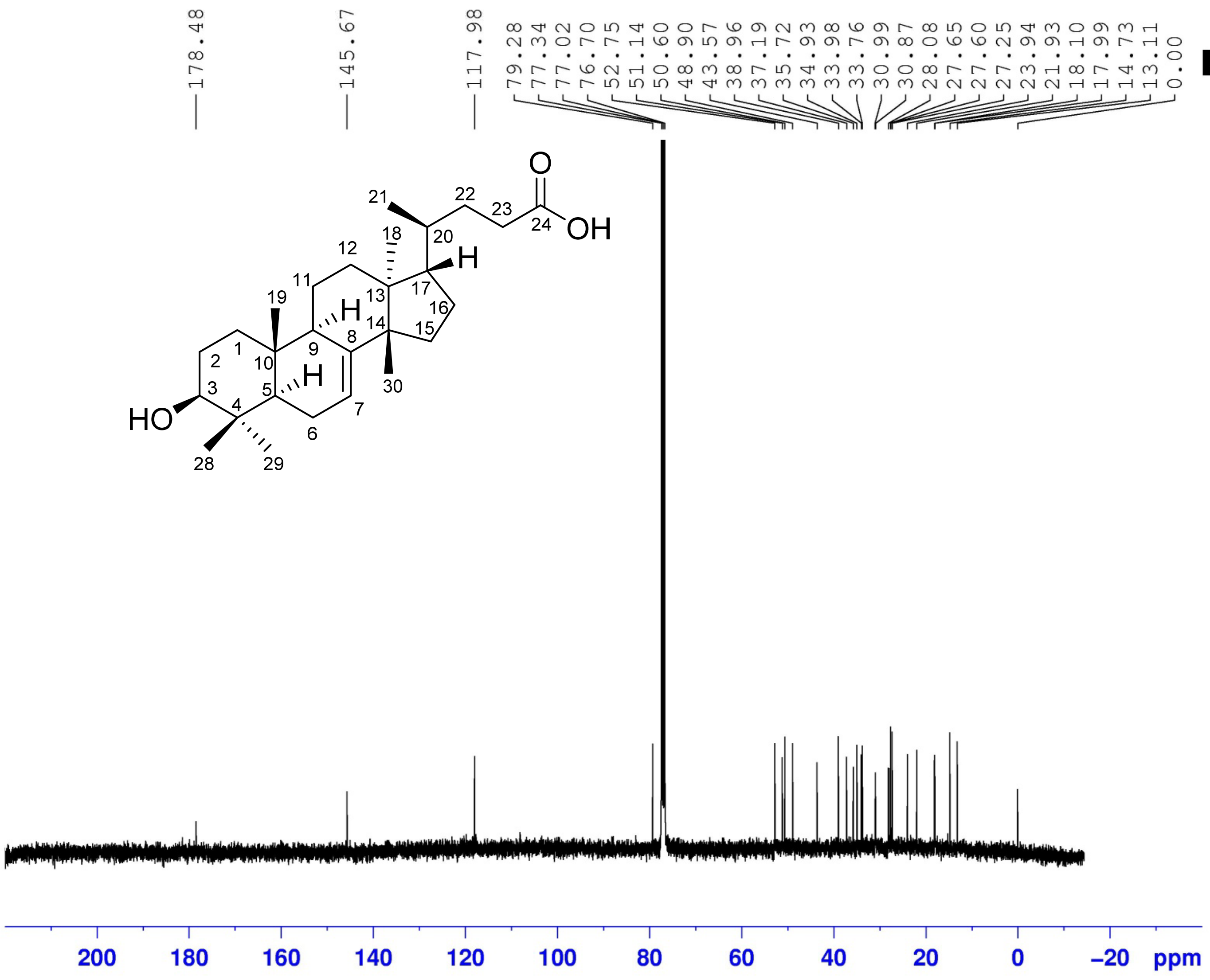

NAME	EA2-27(EA2-33-3)
EXPNO	2
PROCNO	1
Date_	20170613
Time	4.30
INSTRUM	spect
PROBHD	5 mm CPPBBO BB
PULPROG	zgpg30
TD	65536
SOLVENT	CDCl3
NS	500
DS	4
SWH	24038.461 Hz
FIDRES	0.366798 Hz
AQ	1.3631988 sec
RG	85.34
DW	20.800 usec
DE	18.00 usec
TE	297.0 K
D1	2.00000000 sec
D11	0.03000000 sec
TD0	1
======== CHANNEL f1 ========	
SFO1	100.6233324 MHz
NUC1	13C
P1	10.00 usec
SI	32768
SF	100.6127690 MHz
WDW	EM
SSB	0
LB	1.00 Hz
GB	0
PC	1.40

^{13}C NMR (100 MHz) spectrum of compound **9** in $CDCl_3$

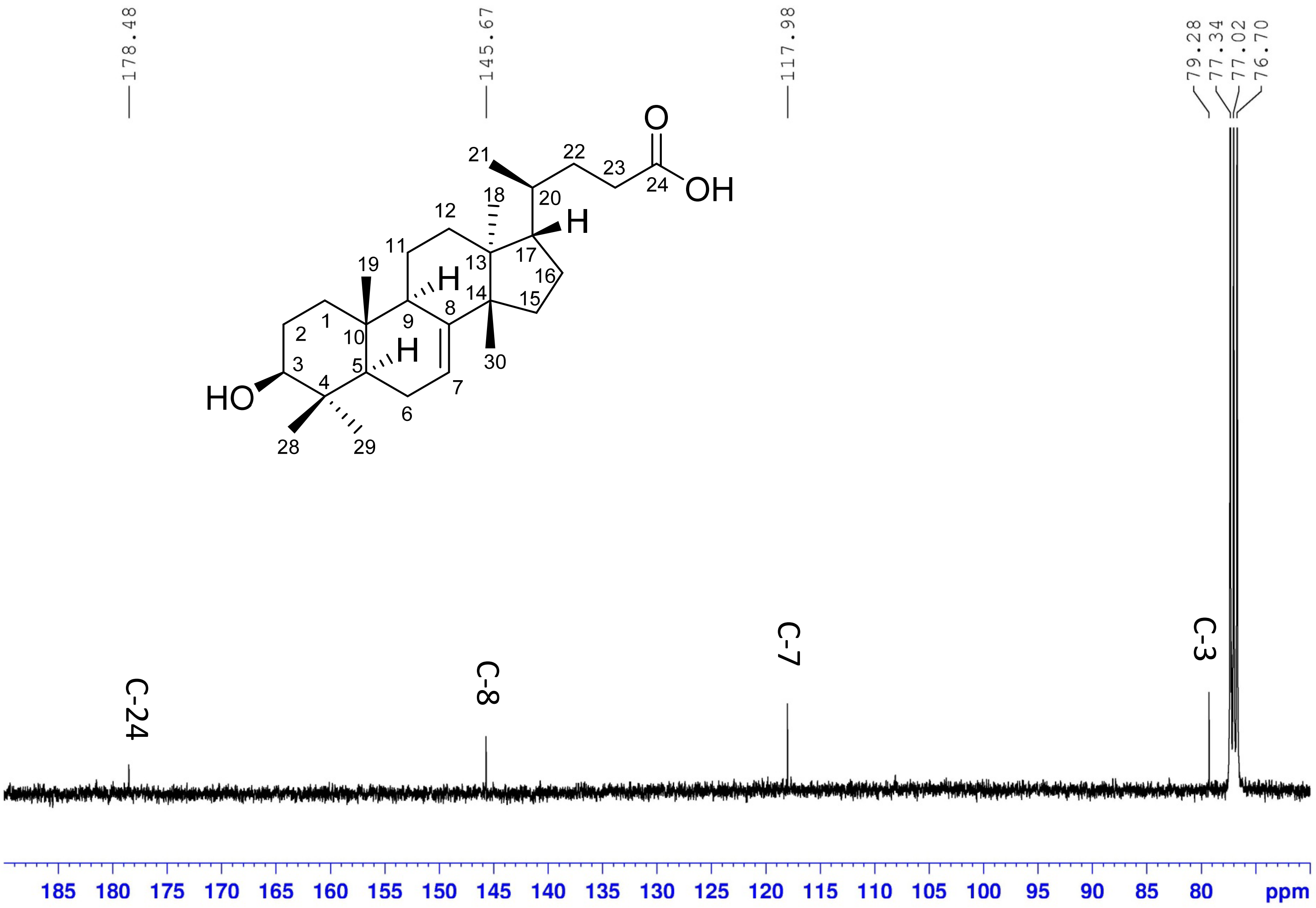

^{13}C NMR (100 MHz) spectrum of compound **9** in $CDCl_3$

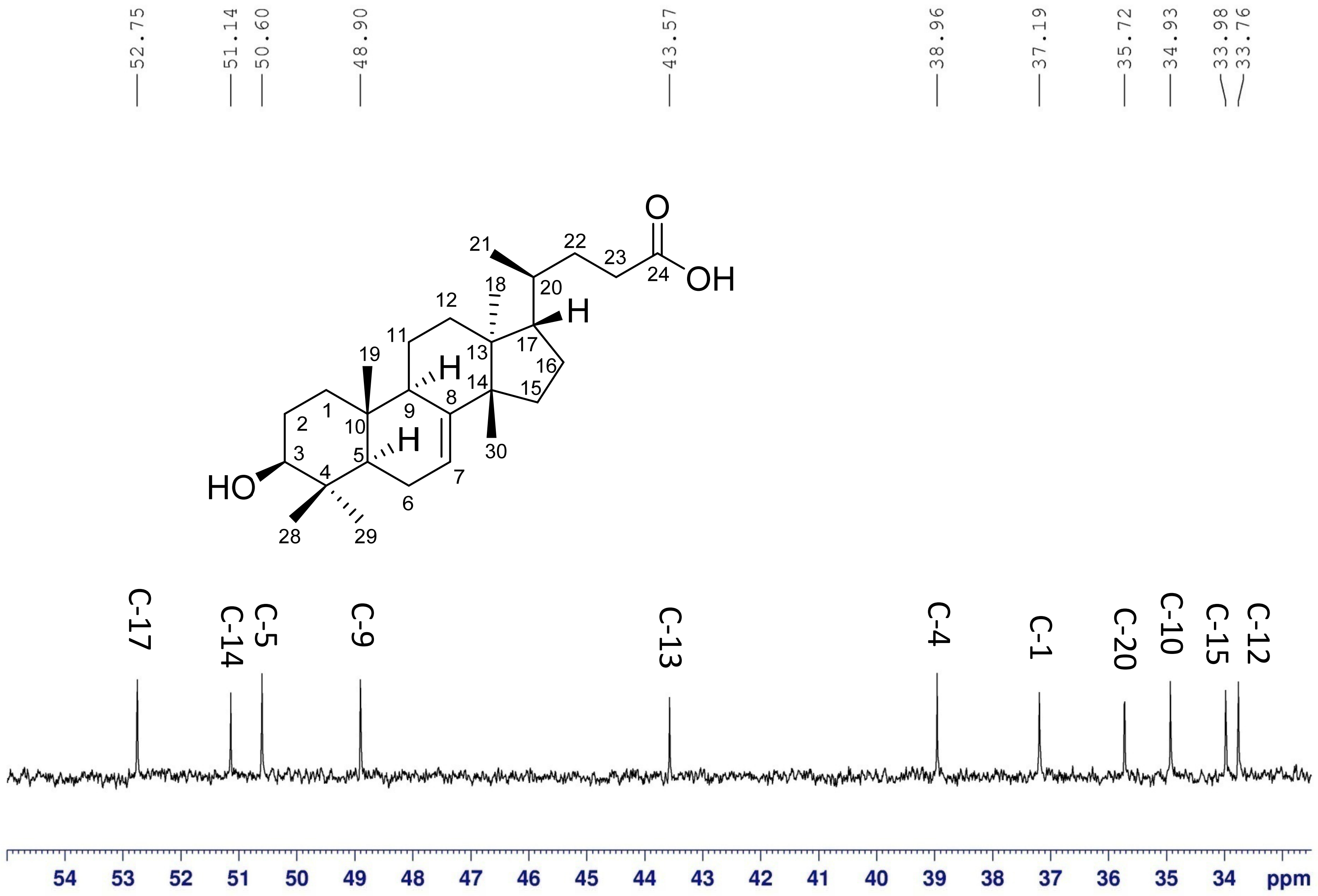

^{13}C NMR (100 MHz) spectrum of compound **9** in $CDCl_3$

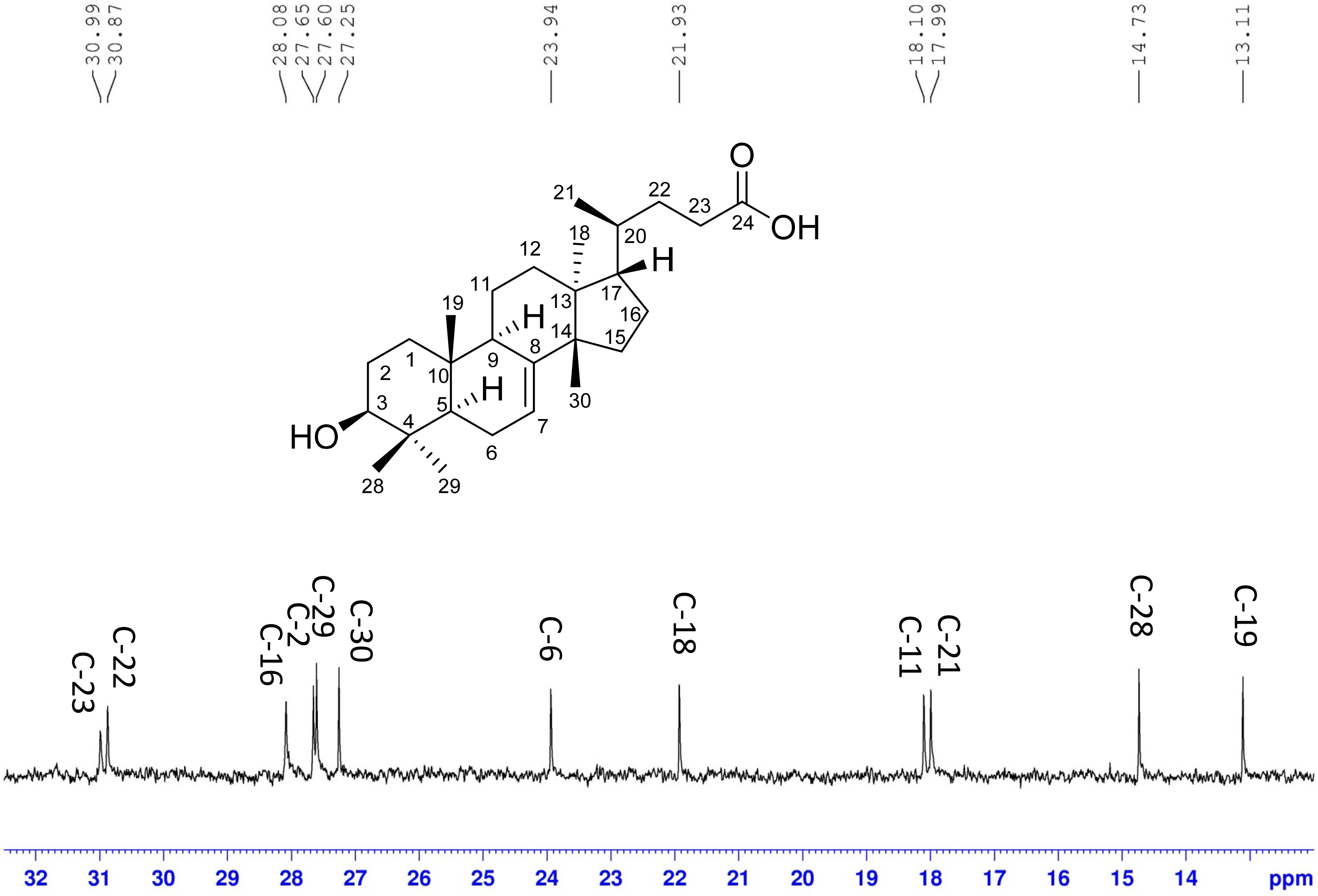

DEPT135 (100 MHz) spectrum of compound **9** in $CDCl_3$

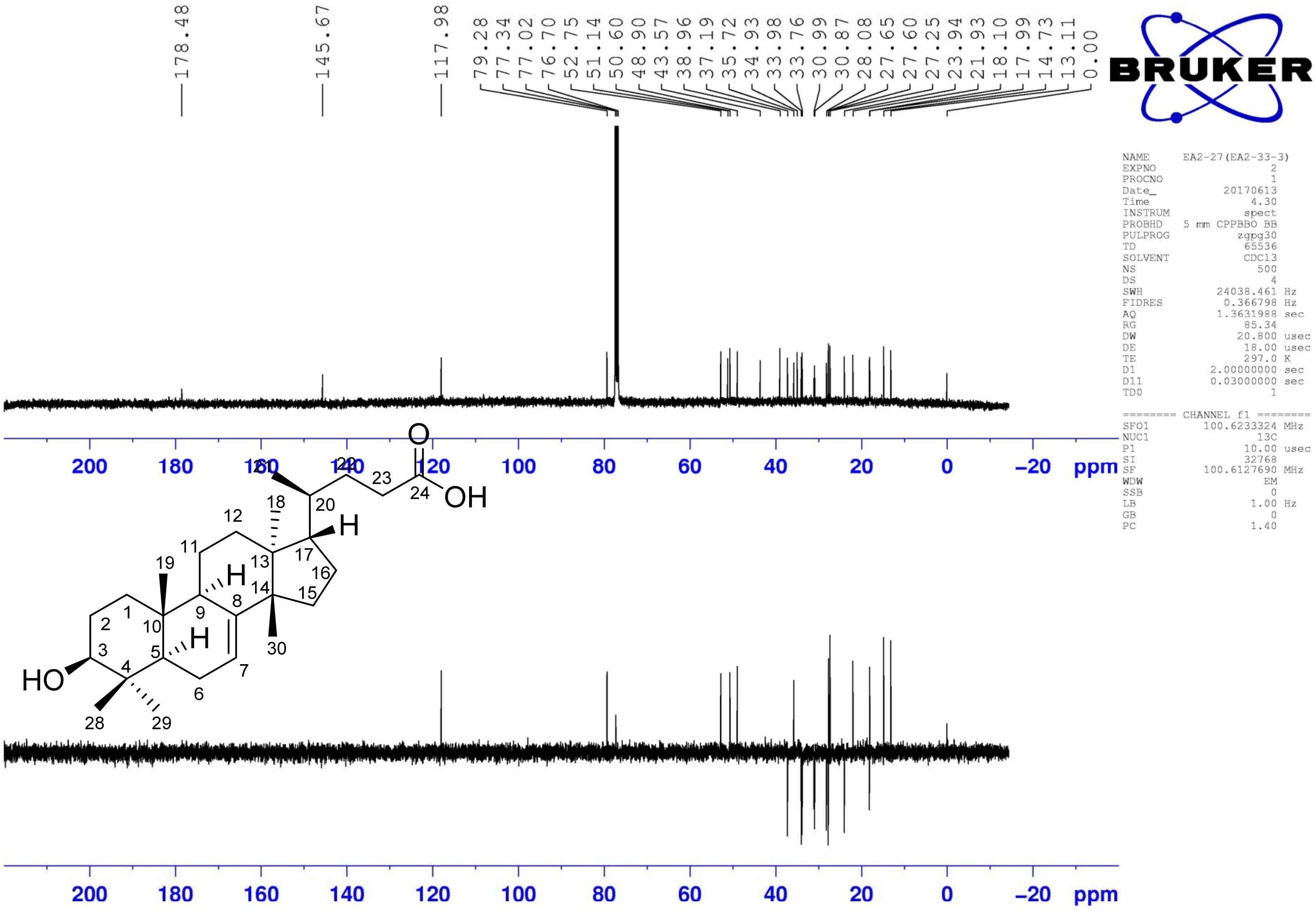

DEPT135 (100 MHz) spectrum of compound **9** in $CDCl_3$

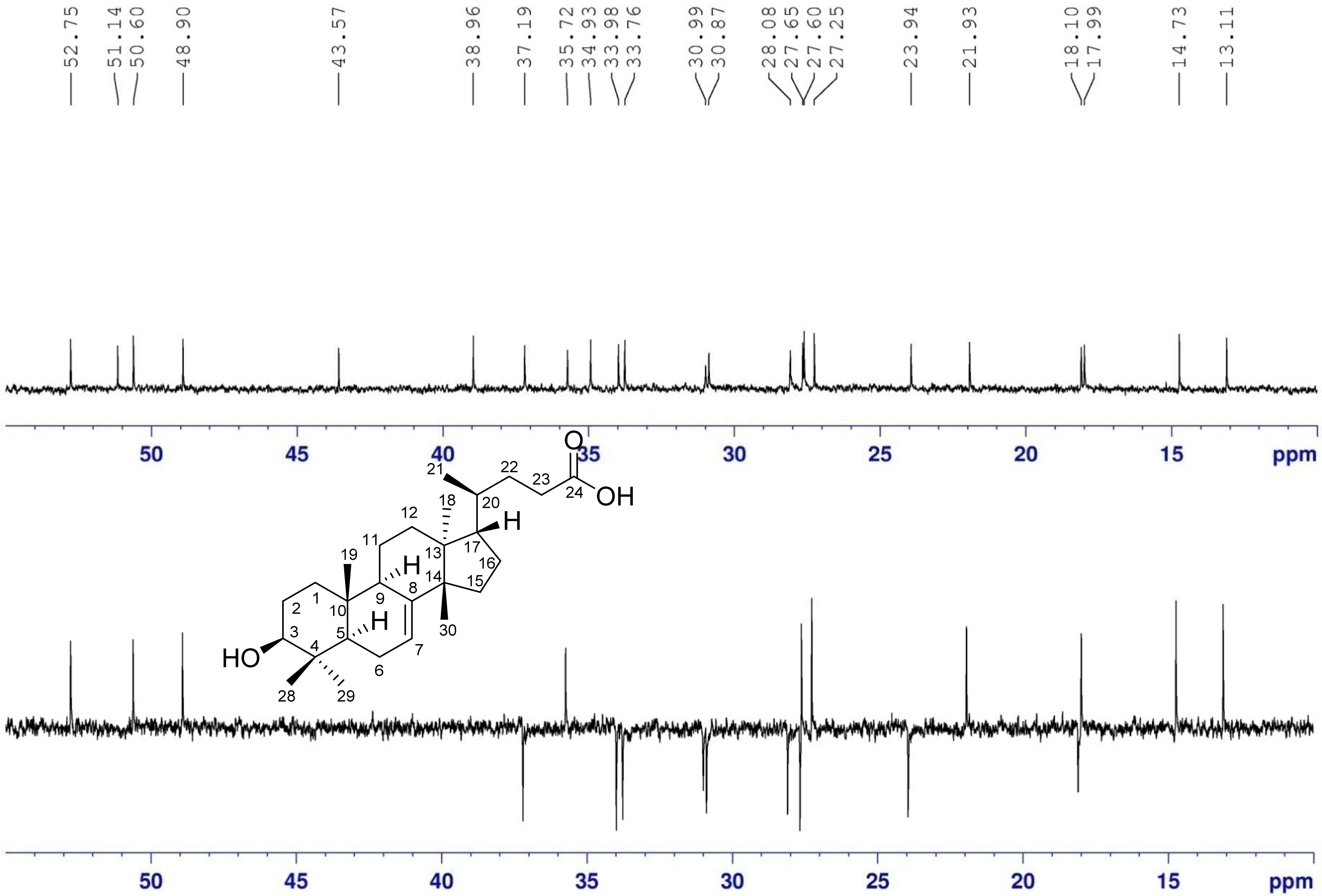

^{1}H-^{1}H COSY (400 MHz) spectrum of compound **9** in $CDCl_3$

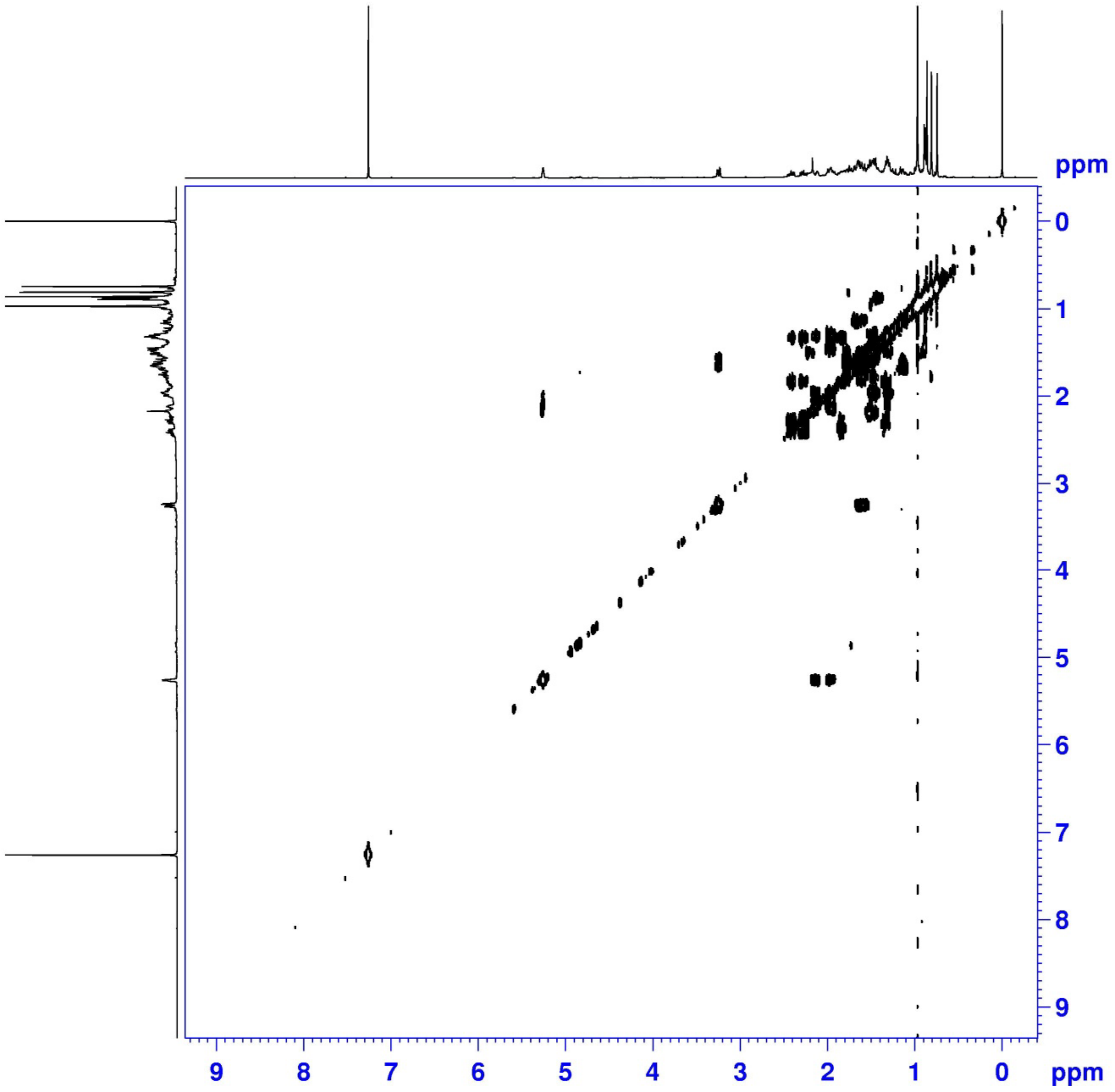

NAME	EA2-27(EA2-33-3)
EXPNO	103
PROCNO	1
Date_	20170620
Time	12.26
INSTRUM	spect
PROBHD	5 mm CPPBBO BB
PULPROG	cosygpppqf
TD	2048
SOLVENT	CDCl3
NS	8
DS	8
SWH	3906.250 Hz
FIDRES	1.907349 Hz
AQ	0.2621940 sec
RG	208.5
DW	128.000 usec
DE	10.00 usec
TE	297.0 K
D0	0.00000300 sec
D1	1.89678097 sec
D11	0.03000000 sec
D12	0.00002000 sec
D13	0.00000400 sec
D16	0.00020000 sec
IN0	0.00025600 sec
======== CHANNEL f1 ========	
SFO1	400.1318006 MHz
NUC1	1H
P0	11.50 usec
P1	11.50 usec
P17	2500.00 usec
ND0	1
TD	128
SFO1	400.1318 MHz
FIDRES	30.517578 Hz
SW	9.762 ppm
FnMODE	QF
SI	1024
SF	400.1300098 MHz
WDW	QSINE
SSB	0
LB	0.00 Hz
GB	0
PC	1.40
SI	1024
MC2	QF
SF	400.1300098 MHz
WDW	QSINE
SSB	0
LB	0.00 Hz
GB	0

^{1}H-^{1}H COSY (400 MHz) spectrum of compound **9** in $CDCl_3$

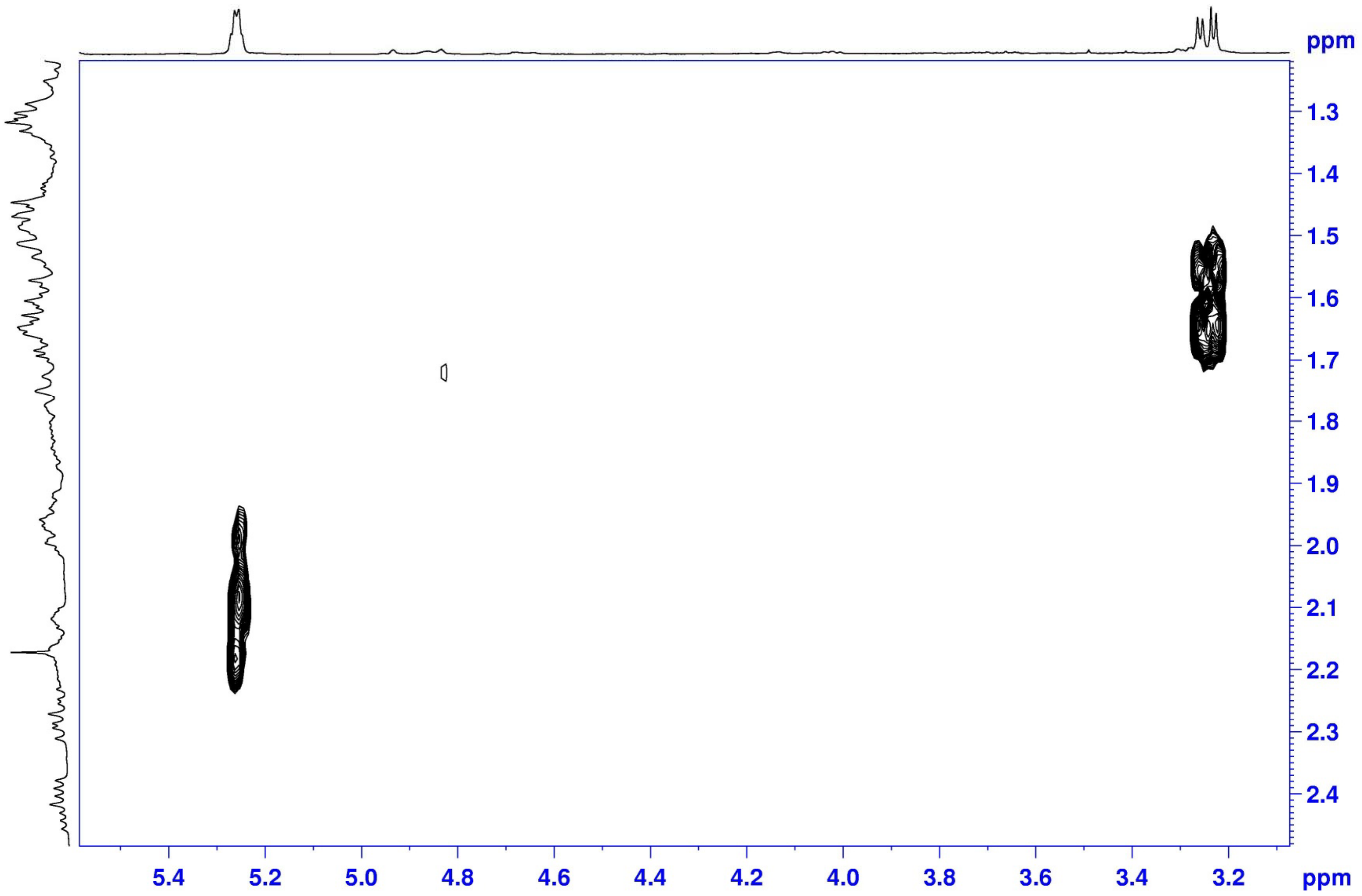

^{1}H-^{1}H COSY (400 MHz) spectrum of compound **9** in $CDCl_3$

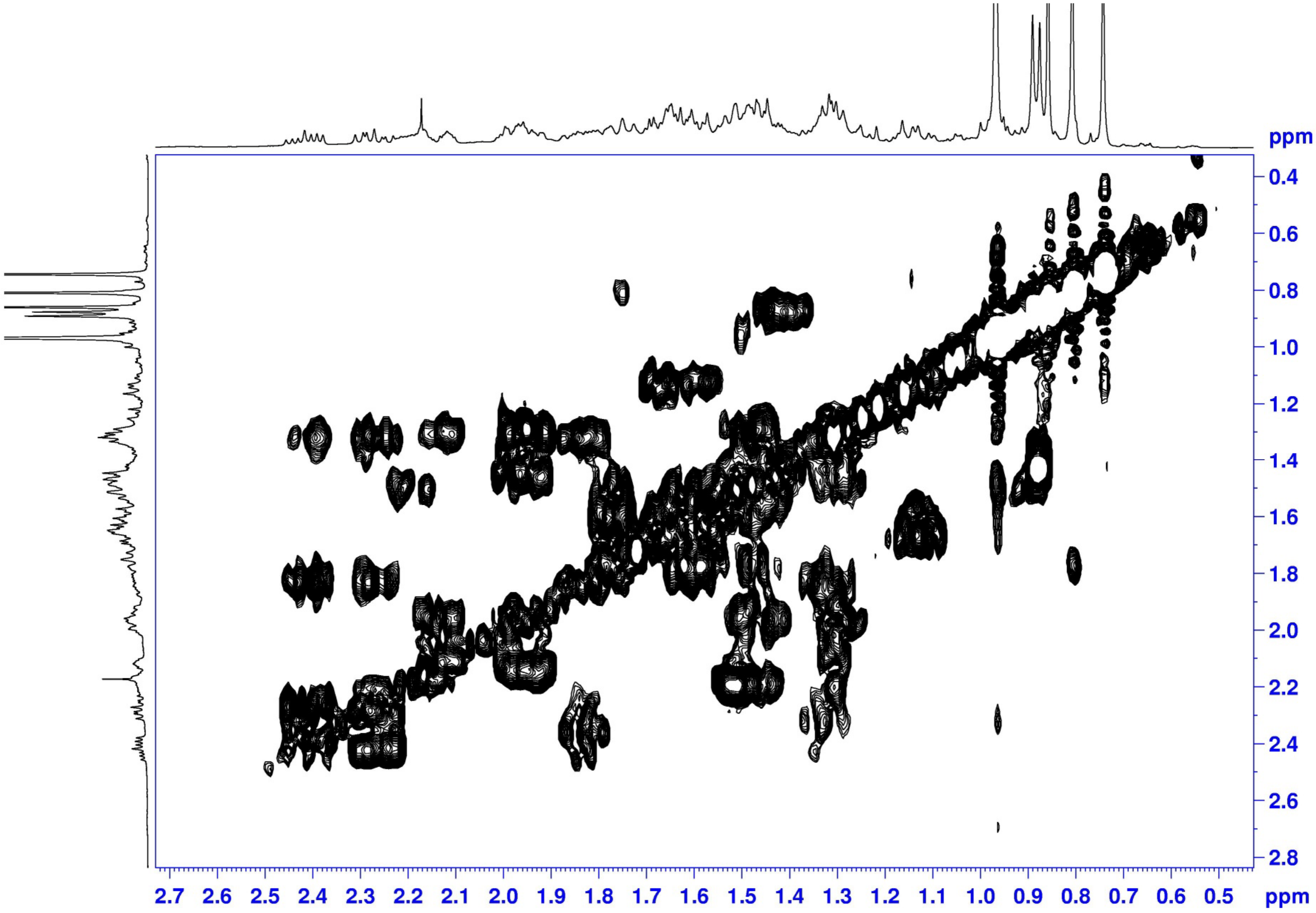

^{1}H-^{1}H COSY (400 MHz) spectrum of compound **9** in $CDCl_3$

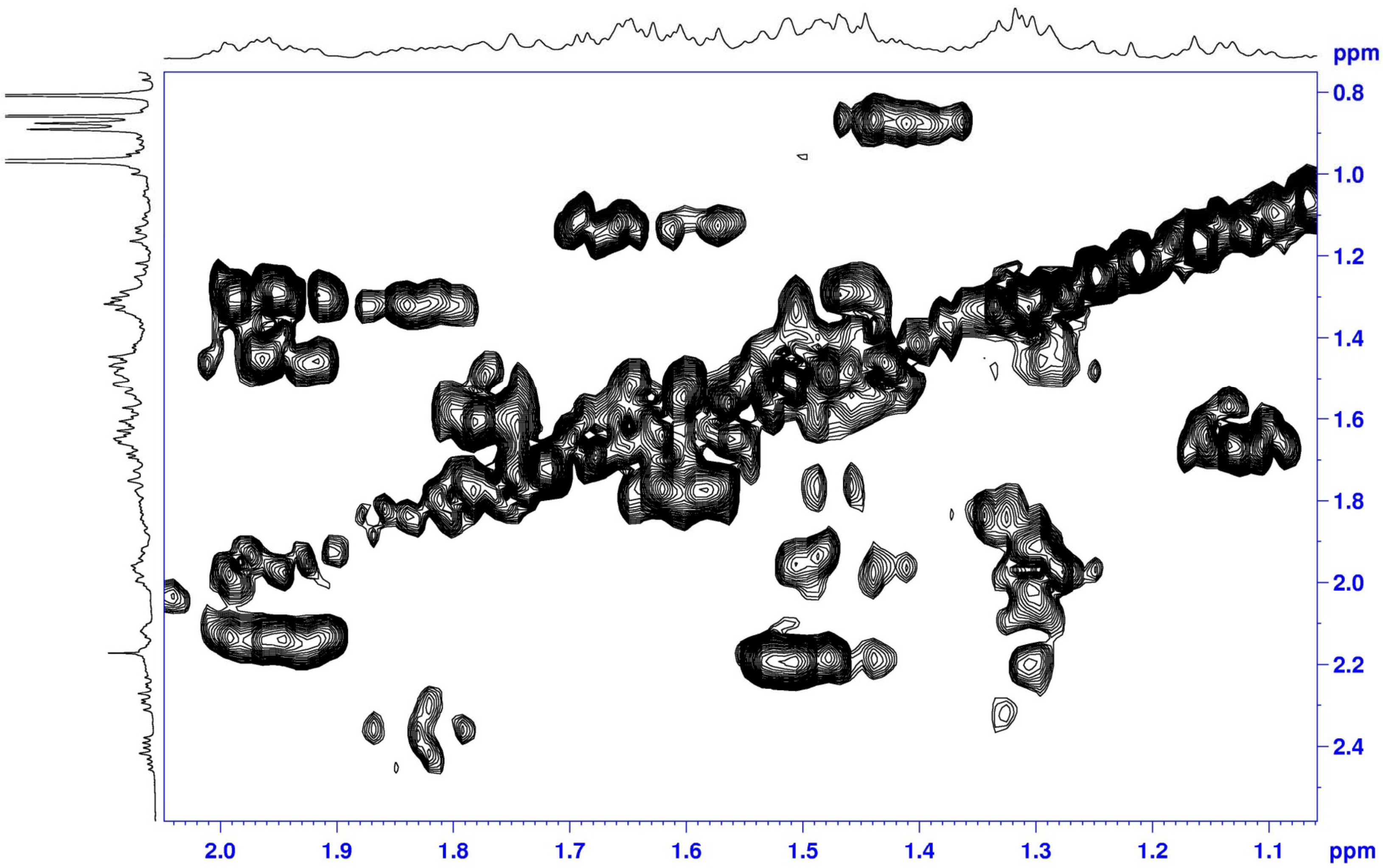

HSQC (400 MHz) spectrum of compound **9** in $CDCl_3$

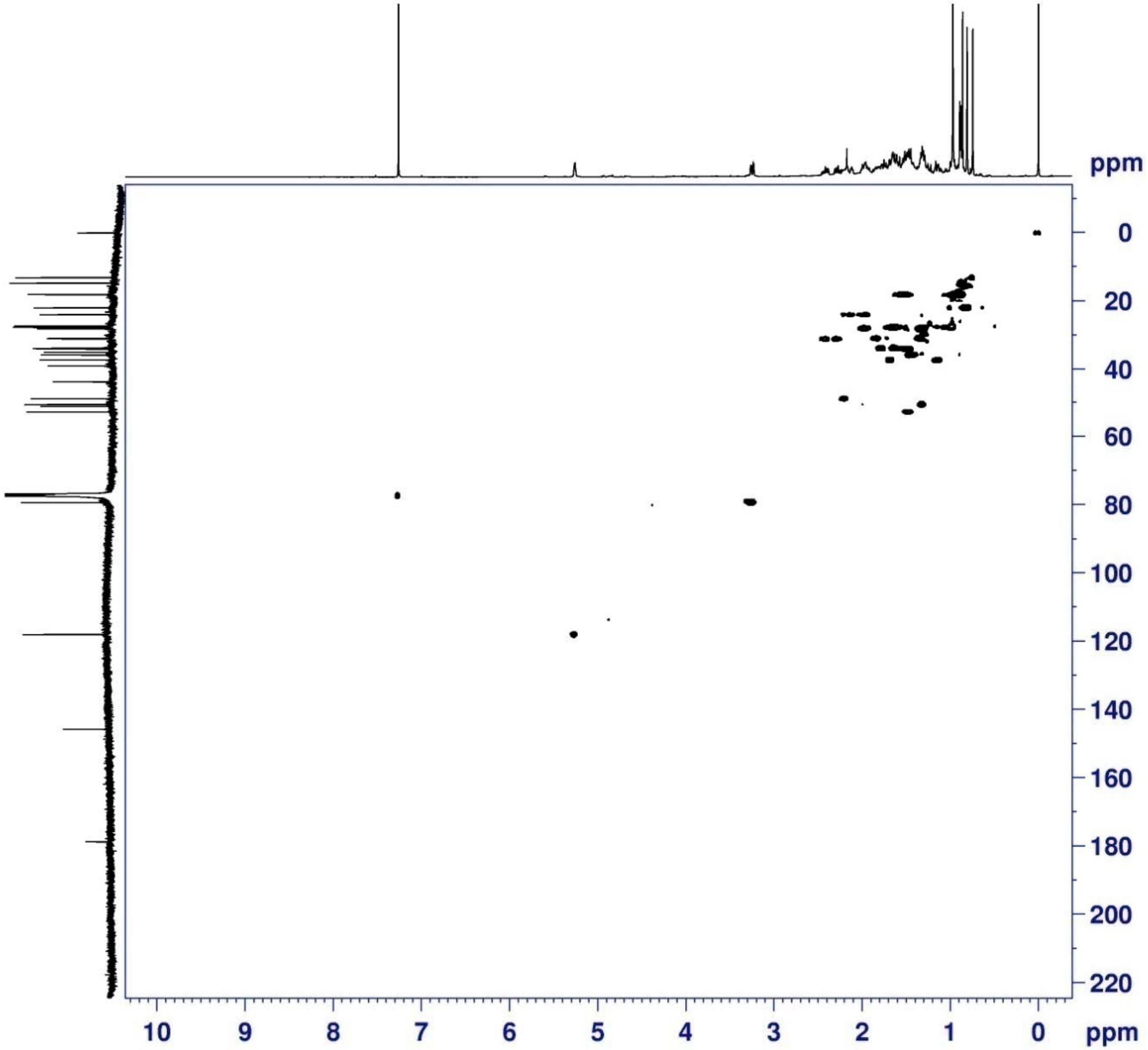


```
NAME       EA2-27(EA2-33-3)
EXPNO                   104
PROCNO                    1
Date_              20170620
Time                  13.06
INSTRUM               spect
PROBHD     5 mm CPPBBO BB
PULPROG         hsqcetgpsi2
TD                     1024
SOLVENT               CDCl3
NS                       16
DS                       16
SWH                4302.926 Hz
FIDRES             4.202076 Hz
AQ                0.1190388 sec
RG                    208.5
DW                  116.200 usec
DE                    10.00 usec
TE                    297.0 K
CNST2           145.0000000
D0               0.00000300 sec
D1               1.46497905 sec
D4               0.00172414 sec
D11              0.03000000 sec
D16              0.00020000 sec
D24              0.00086207 sec
IN0              0.00002080 sec
ZGOPTNS

======== CHANNEL f1 ========
SFO1            400.1320007 MHz
NUC1                     1H
P1                    11.50 usec
P2                    23.00 usec
P28                    0.00 usec
ND0                       2
TD                      256
SFO1               100.6233 MHz
FIDRES            93.900238 Hz
SW                  238.896 ppm
FnMODE         Echo-Antiecho
SI                     1024
SF              400.1300068 MHz
WDW                   QSINE
SSB                       2
LB                     0.00 Hz
GB                        0
PC                     1.40
SI                     1024
MC2            echo-antiecho
SF              100.6127543 MHz
WDW                   QSINE
SSB                       2
LB                     0.00 Hz
[illegible]
```

HSQC (400 MHz) spectrum of compound **9** in $CDCl_3$

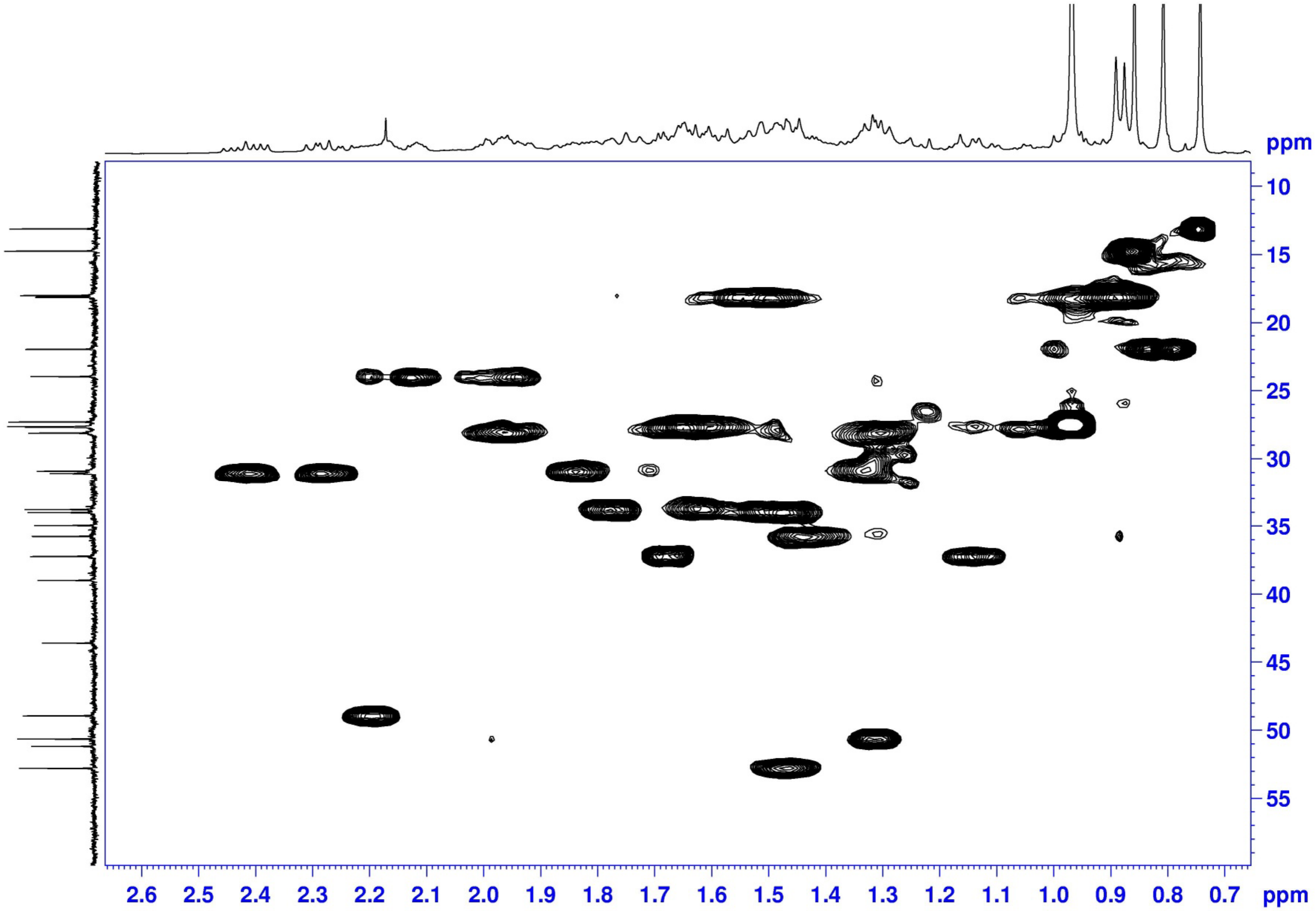

HSQC (400 MHz) spectrum of compound **9** in $CDCl_3$

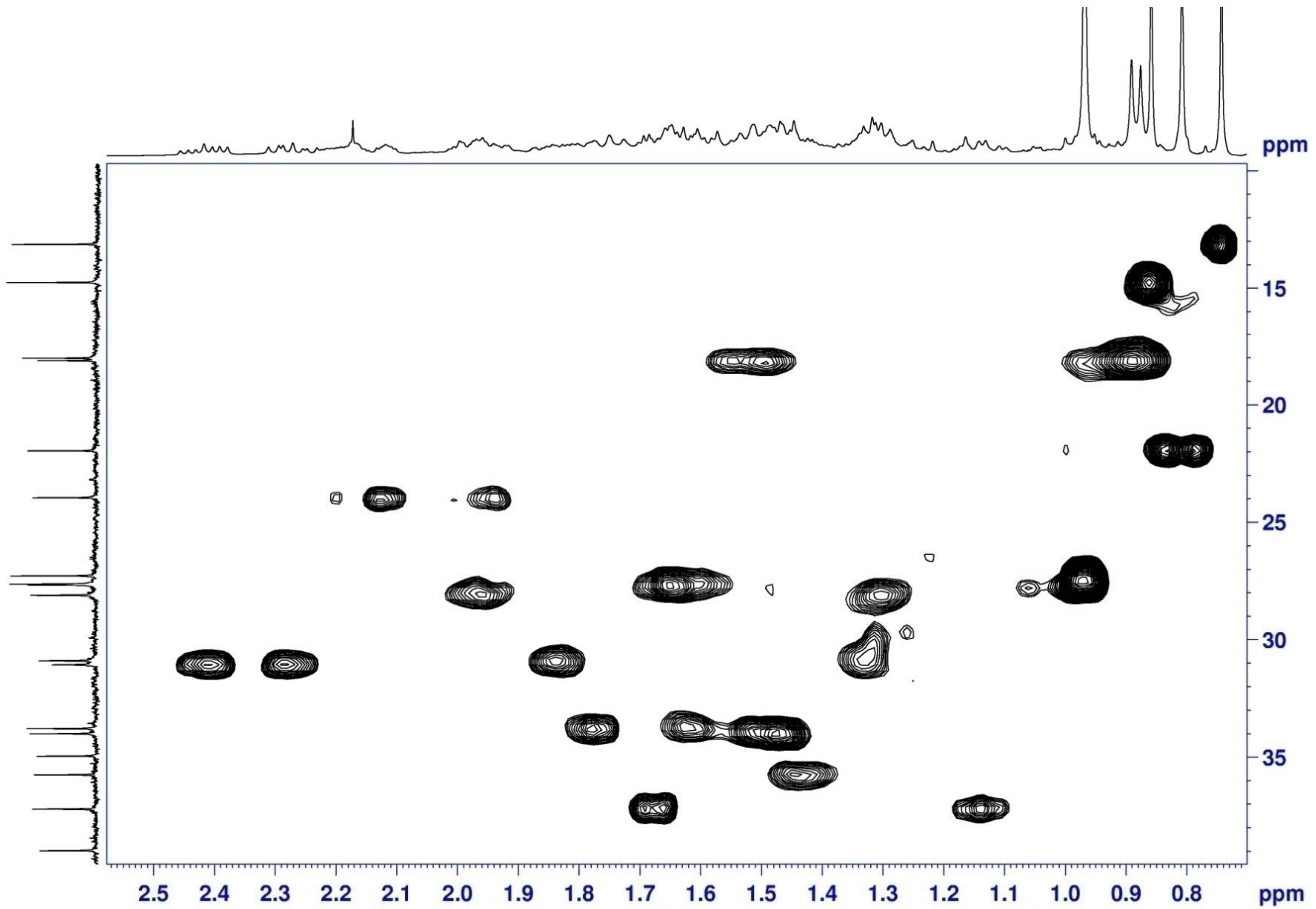

HMBC (400 MHz) spectrum of compound **9** in $CDCl_3$

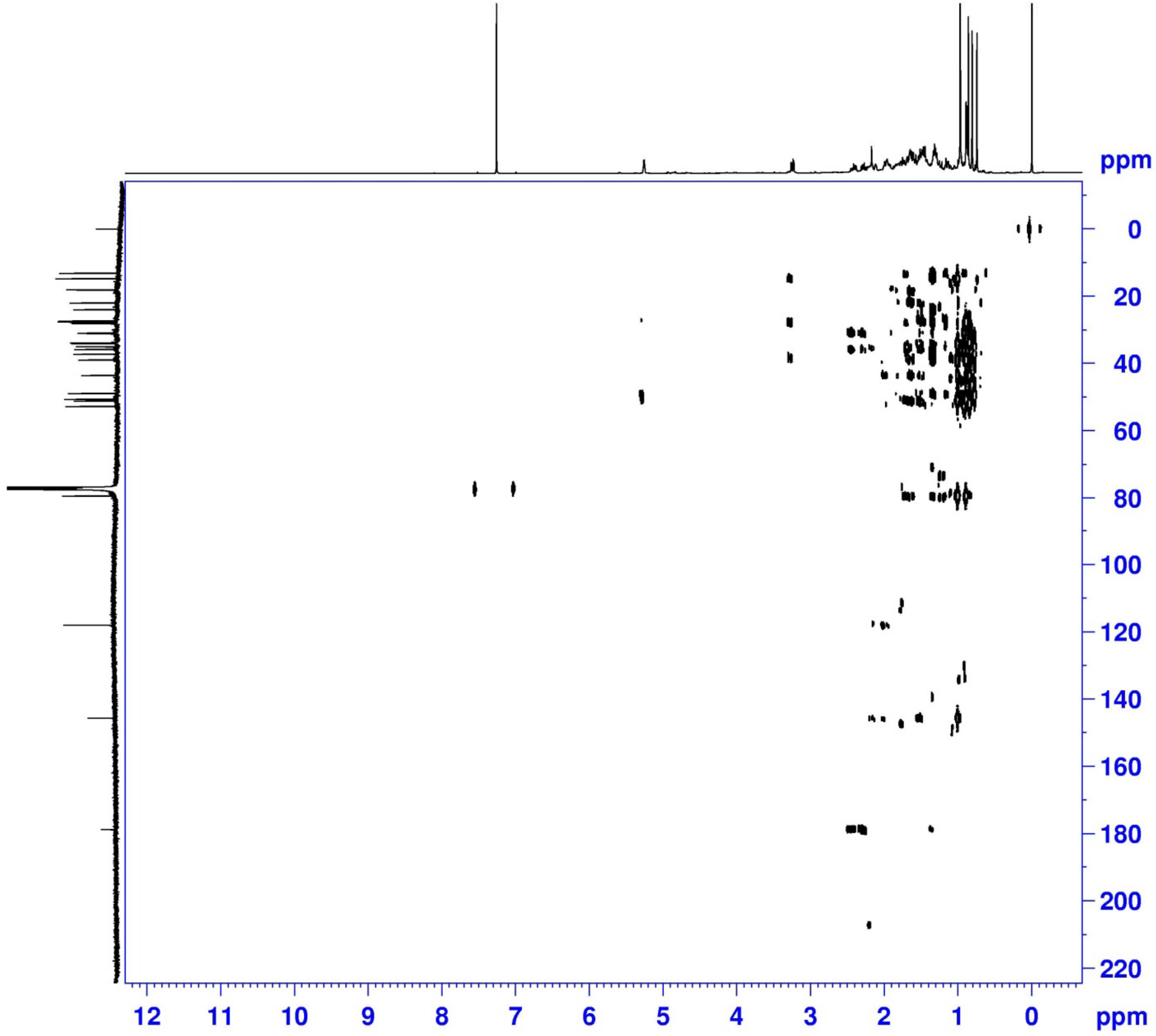

NAME	EA2-27(EA2-33-3)	
EXPNO	1006	
PROCNO	1	
Date_	20170620	
Time	23.02	
INSTRUM	spect	
PROBHD	5 mm CPPBBO BB	
PULPROG	hmbcgplpndqf	
TD	4096	
SOLVENT	CDCl3	
NS	64	
DS	16	
SWH	5197.505	Hz
FIDRES	1.268922	Hz
AQ	0.3940852	sec
RG	208.5	
DW	96.200	usec
DE	10.00	usec
TE	297.0	K
CNST2	145.0000000	
CNST13	10.0000000	
D0	0.00000300	sec
D1	1.50000000	sec
D2	0.00344828	sec
D6	0.05000000	sec
D16	0.00020000	sec
IN0	0.00002080	sec
======== CHANNEL f1 ========		
SFO1	400.1323208	MHz
NUC1	1H	
P1	11.50	usec
P2	23.00	usec
ND0	2	
TD	128	
SFO1	100.6233	MHz
FIDRES	187.800476	Hz
SW	238.896	ppm
FnMODE	QF	
SI	2048	
SF	400.1300000	MHz
WDW	SINE	
SSB	0	
LB	0.00	Hz
GB	0	
PC	1.40	
SI	1024	
MC2	QF	
SF	100.6127685	MHz
WDW	SINE	
SSB	0	
[illegible]	[illegible]	[illegible]

HMBC (400 MHz) spectrum of compound **9** in $CDCl_3$

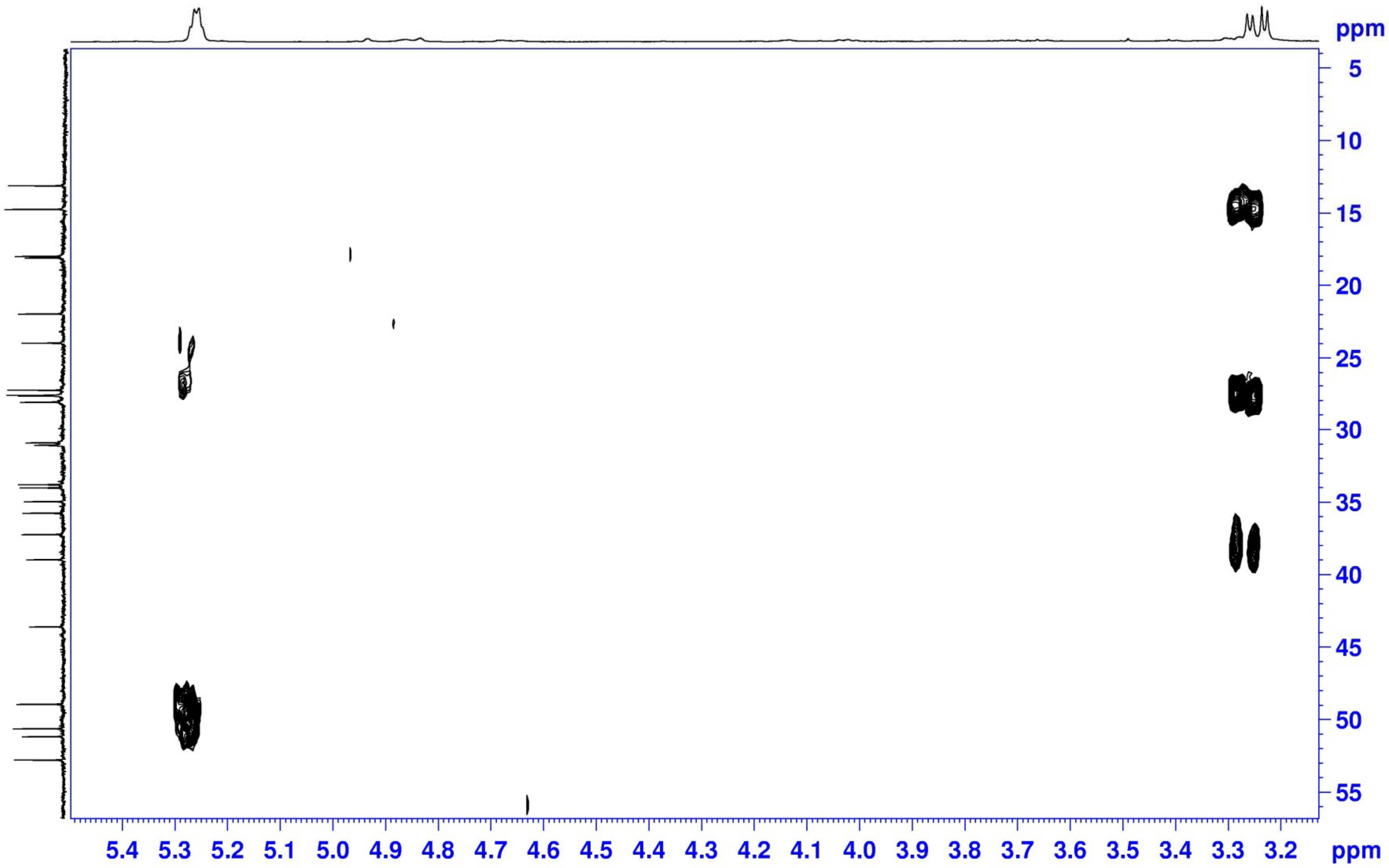

HMBC (400 MHz) spectrum of compound **9** in $CDCl_3$

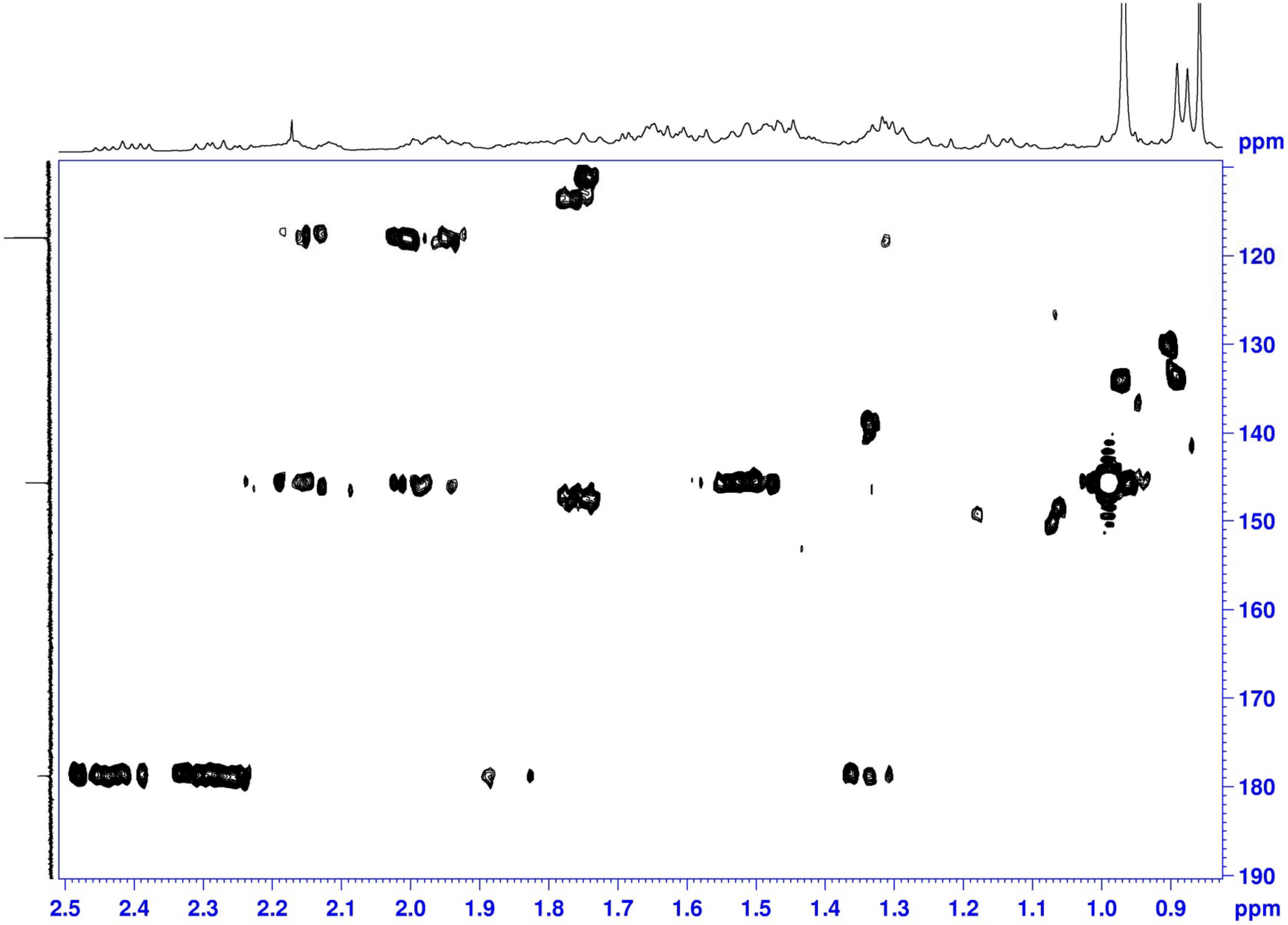

HMBC (400 MHz) spectrum of compound **9** in $CDCl_3$

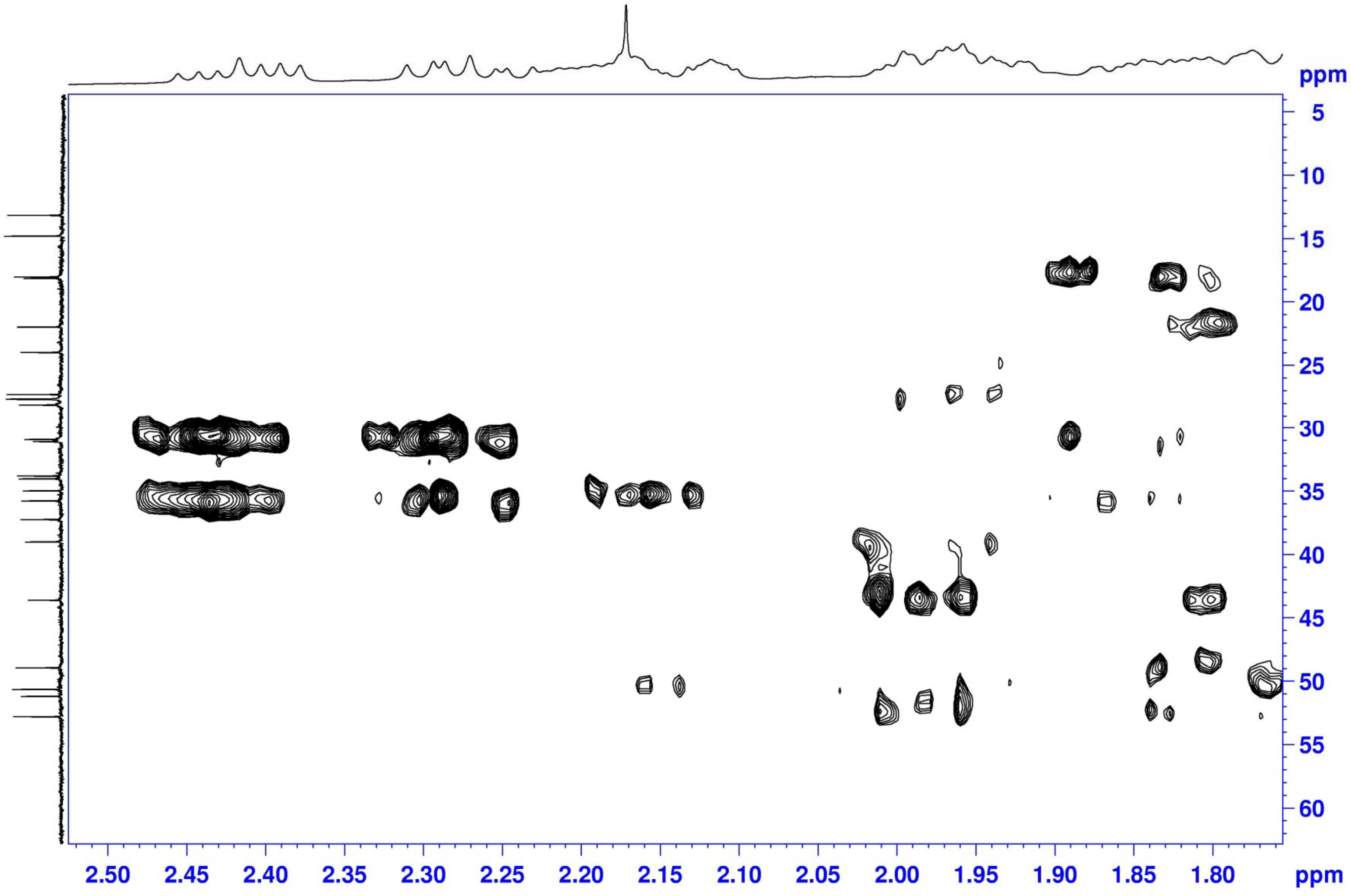

HMBC (400 MHz) spectrum of compound **9** in $CDCl_3$

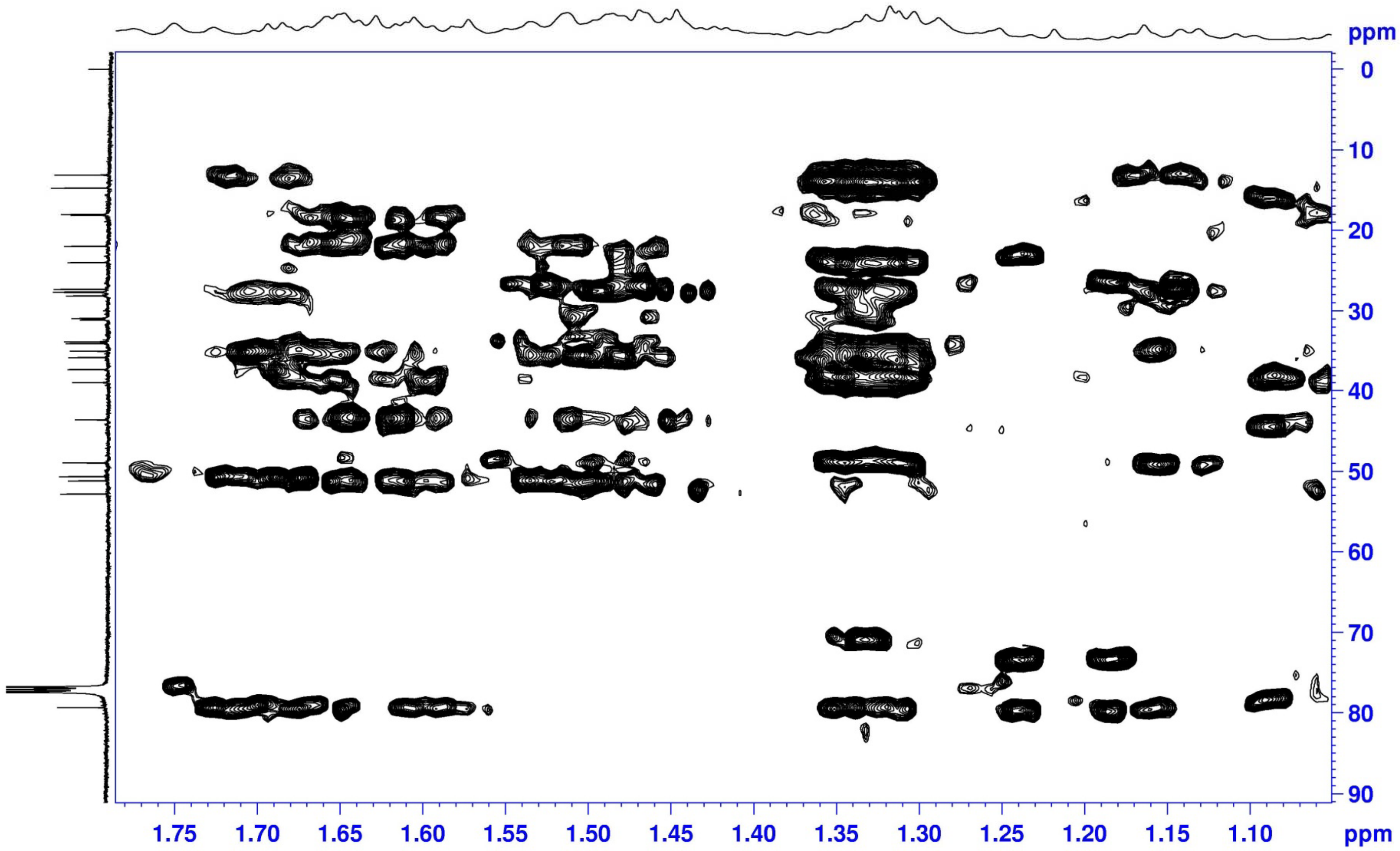

HMBC (400 MHz) spectrum of compound **9** in $CDCl_3$

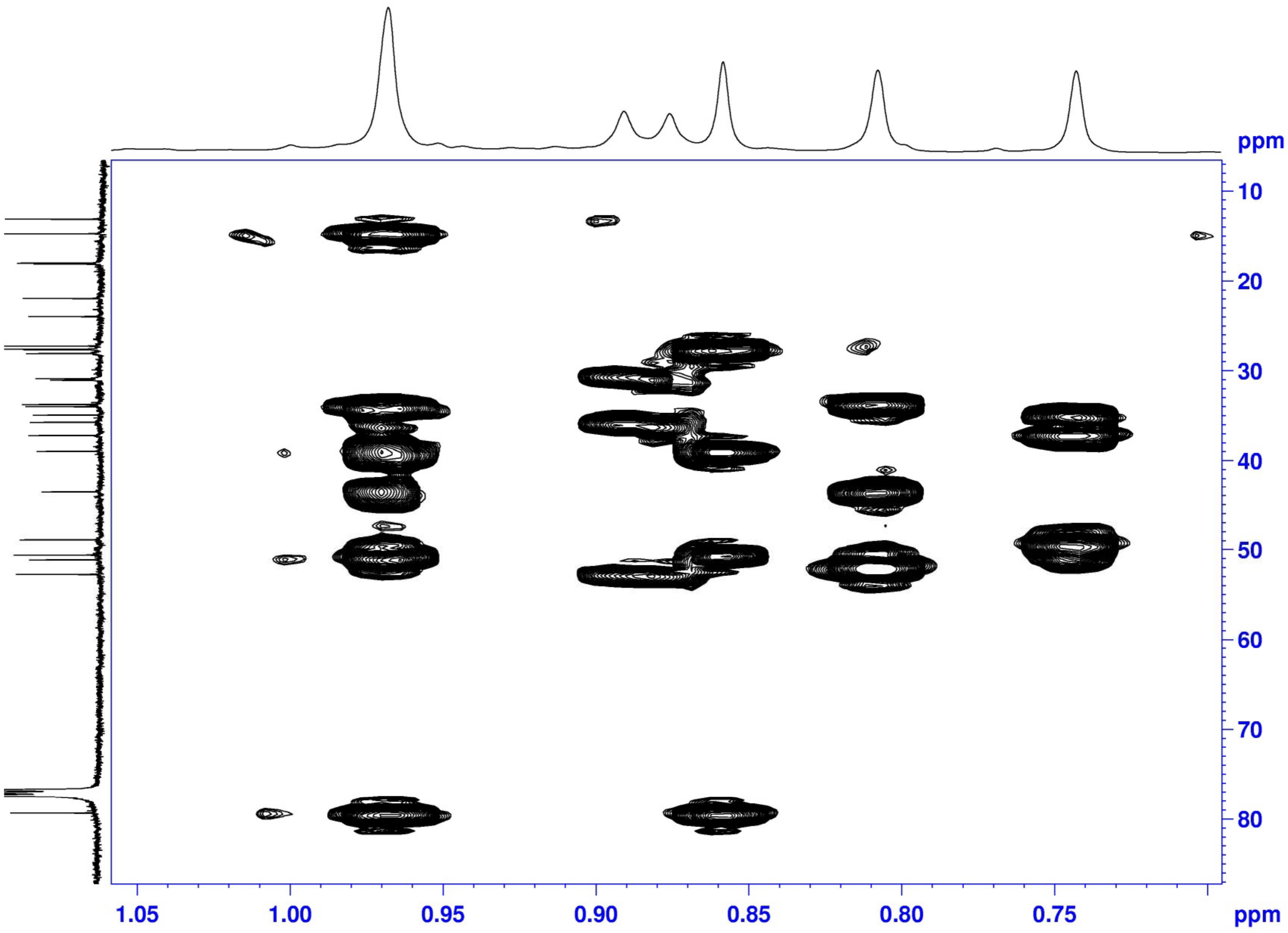

NOESY (400 MHz) spectrum of compound **9** in $CDCl_3$

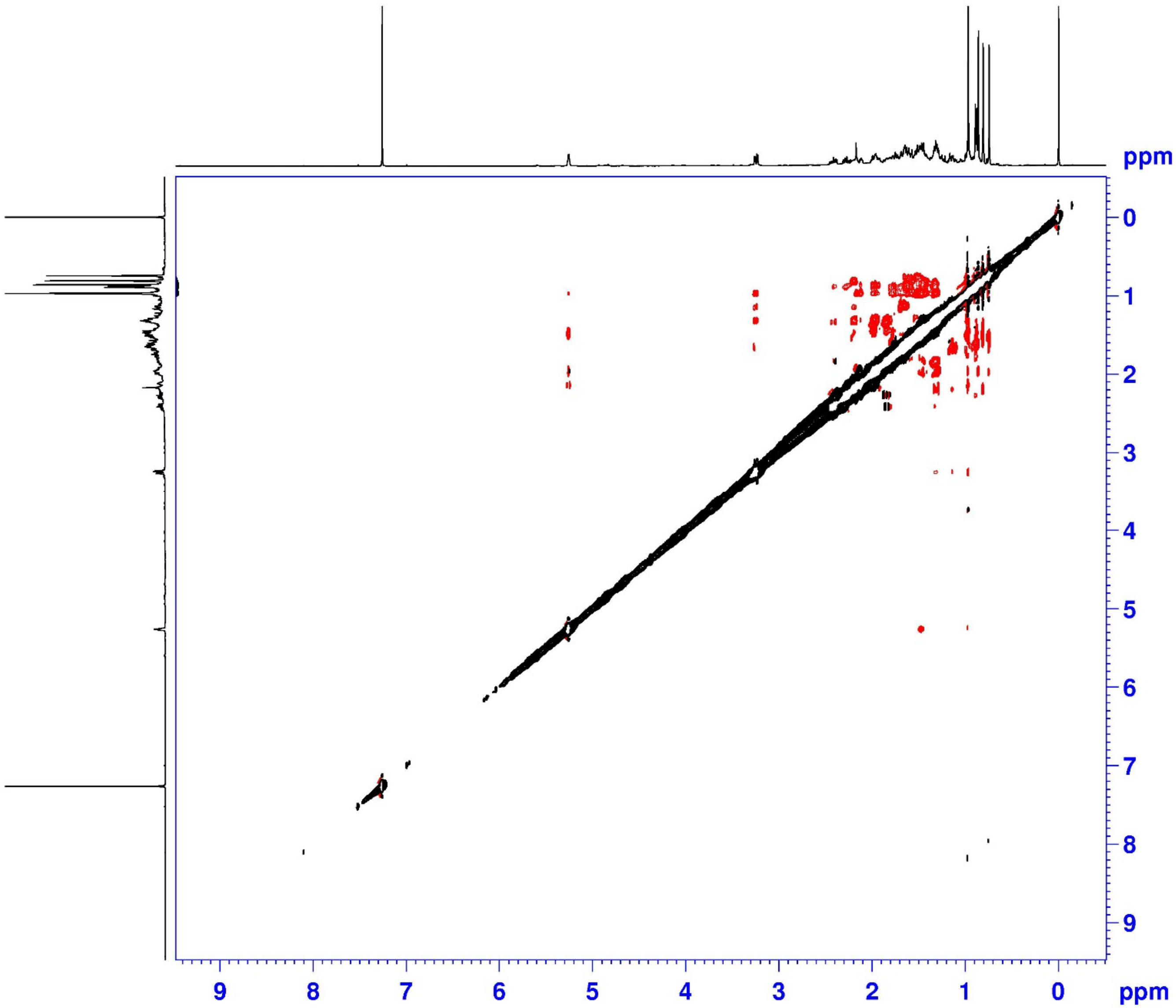


```
NAME        EA2-27(EA2-33-3)
EXPNO                 1007
PROCNO                   1
Date_             20170621
Time                  3.31
INSTRUM              spect
PROBHD      5 mm CPPBBO BB
PULPROG        noesygpphpp
TD                    2048
SOLVENT              CDCl3
NS                      32
DS                      32
SWH               4000.000 Hz
FIDRES            1.953125 Hz
AQ               0.2560500 se
RG                   208.5
DW                 125.000 us
DE                   10.00 us
TE                   297.0 K
D0              0.00011036 se
D1              1.99385595 se
D8              0.30000001 se
D11             0.03000000 se
D12             0.00002000 se
D16             0.00020000 se
IN0             0.00025000 se

======== CHANNEL f1 ======
SFO1           400.1318006 MH
NUC1                    1H
P1                   11.50 us
P2                   23.00 us
P17                2500.00 us
ND0                      1
TD                     256
SFO1              400.1318 MH
FIDRES           15.625000 Hz
SW                   9.997 pp
FnMODE         States-TPPI
SI                    1024
SF             400.1300098 MH
WDW                  QSINE
SSB                      2
LB                    0.00 Hz
GB                       0
PC                    1.00
SI                    1024
MC2            States-TPPI
SF             400.1300098 MH
WDW                  QSINE
SSB                      2
```

NOESY (400 MHz) spectrum of compound **9** in $CDCl_3$

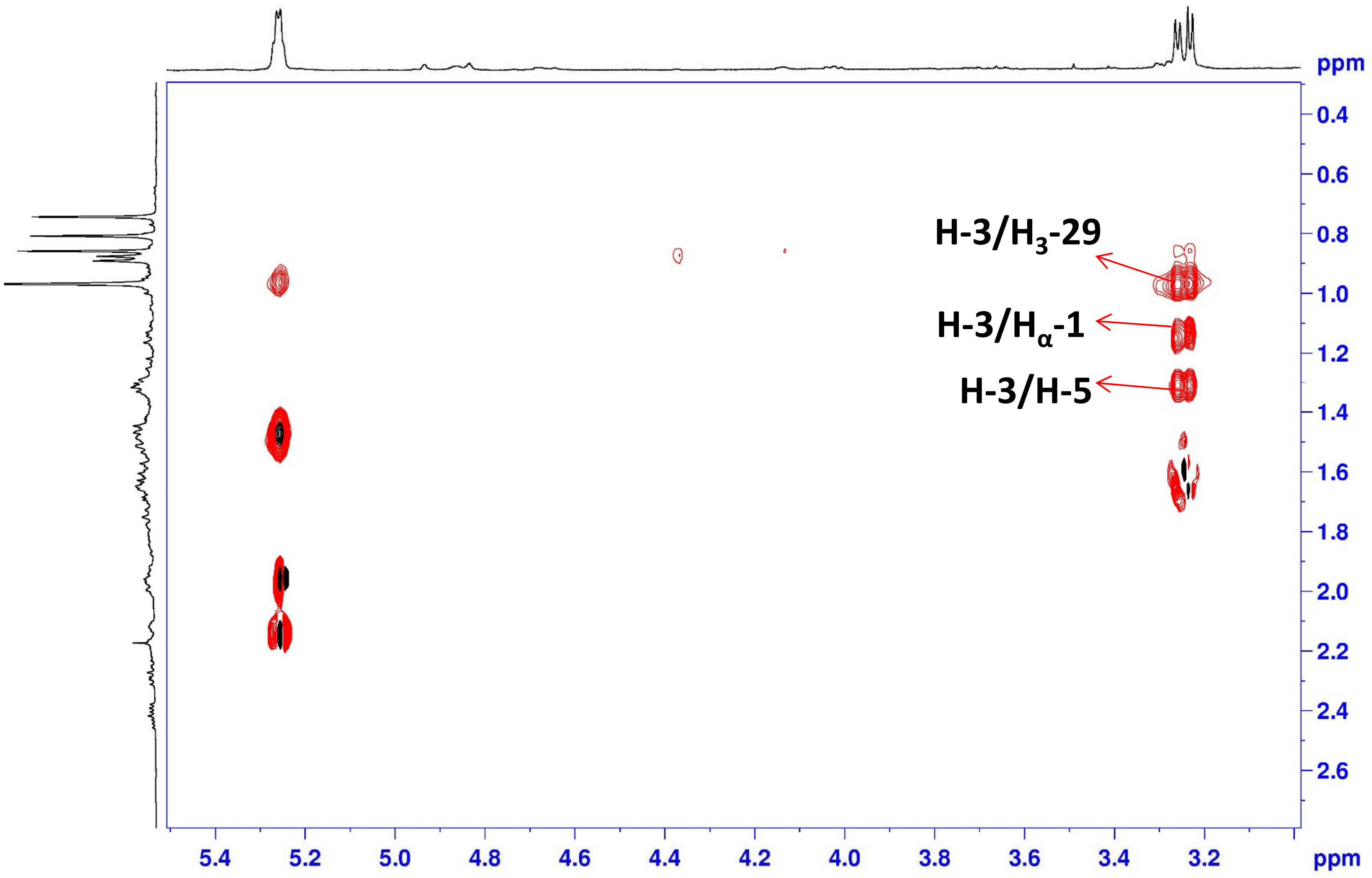

NOESY (400 MHz) spectrum of compound **9** in $CDCl_3$

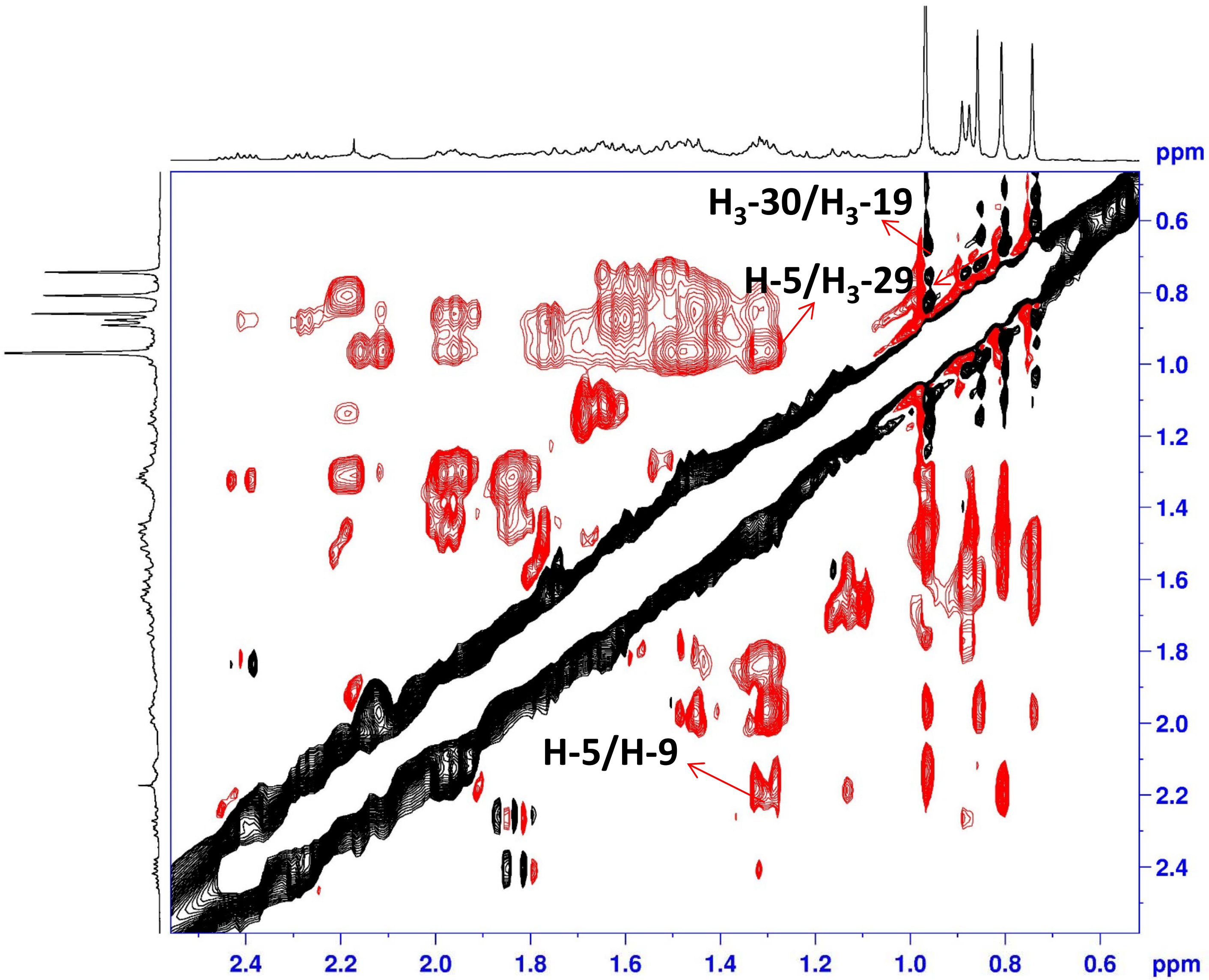

NOESY (400 MHz) spectrum of compound **9** in $CDCl_3$

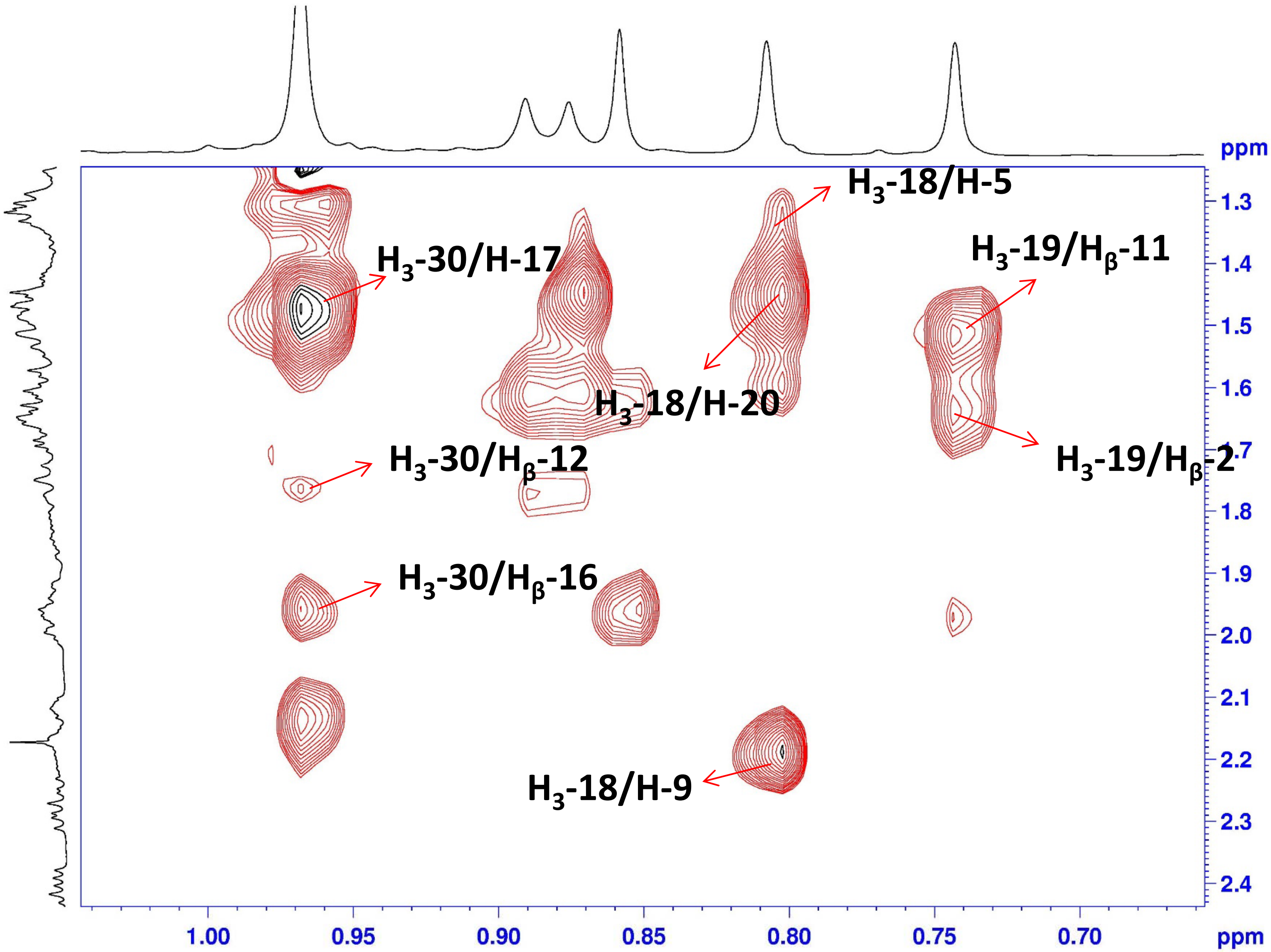

HR-ESIMS for compound **10**

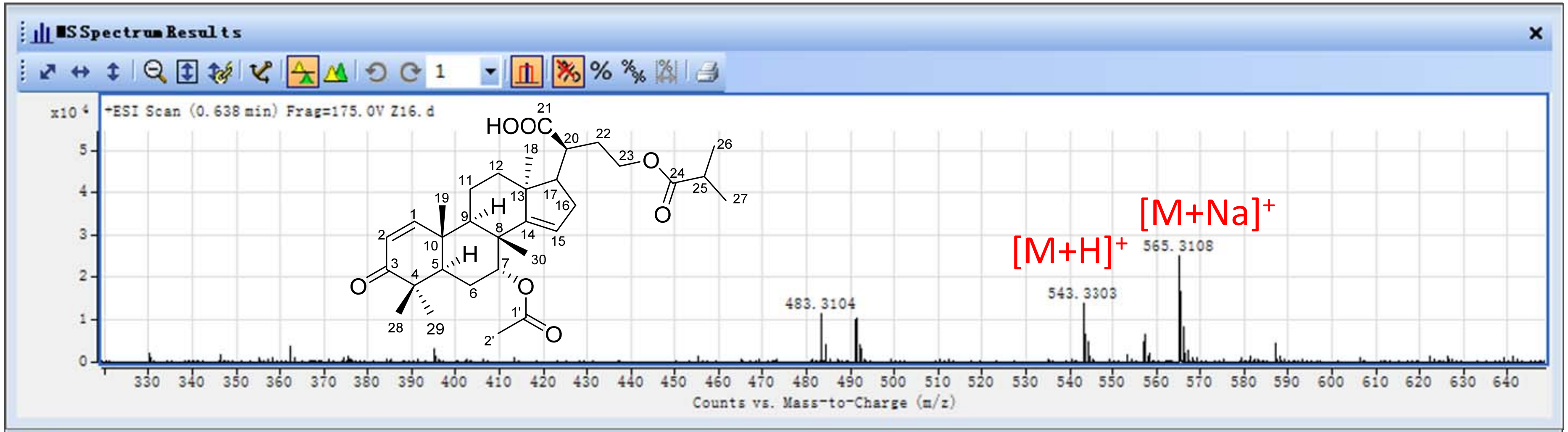

MS Formula Results: + Scan (0.638 min)

m/z	Ion	Formula	Abundance
543.3303	(M+H)+	C32 H47 O7	13701.3

Best	Formula (M)	Ion Formula	Score	Cross Score	Calc m/z	Diff (ppm)	Mass Match	Abund Match	Spacing Match
☑	C32 H46 O7	C32 H47 O7	94.53		543.3316	2.58	92.57	97.86	94.46

Isotope	Abund Sum%	Calc Abund Sum%	m/z	Calc m/z	Diff (ppm)
1	70.87	69.38	543.3303	543.3316	2.51
2	24.01	24.57	544.3327	544.335	4.31
3	4.26	5.22	545.3389	545.338	-1.66
4	0.86	0.82	546.3466	546.3408	-10.67

Best	Formula (M)	Ion Formula	Score	Cross Score	Calc m/z	Diff (ppm)	Mass Match	Abund Match	Spacing Match
☐	C39 H42 O2	C39 H43 O2	65.16		543.3258	-8.25	45.53	72.77	95.26
☐	C26 H51 Cl O9	C26 H52 Cl O9	62.58		543.3294	-1.46	97.56	0.14	67.56

^{1}H NMR (400 MHz) spectrum of compound **10** in $CDCl_3$

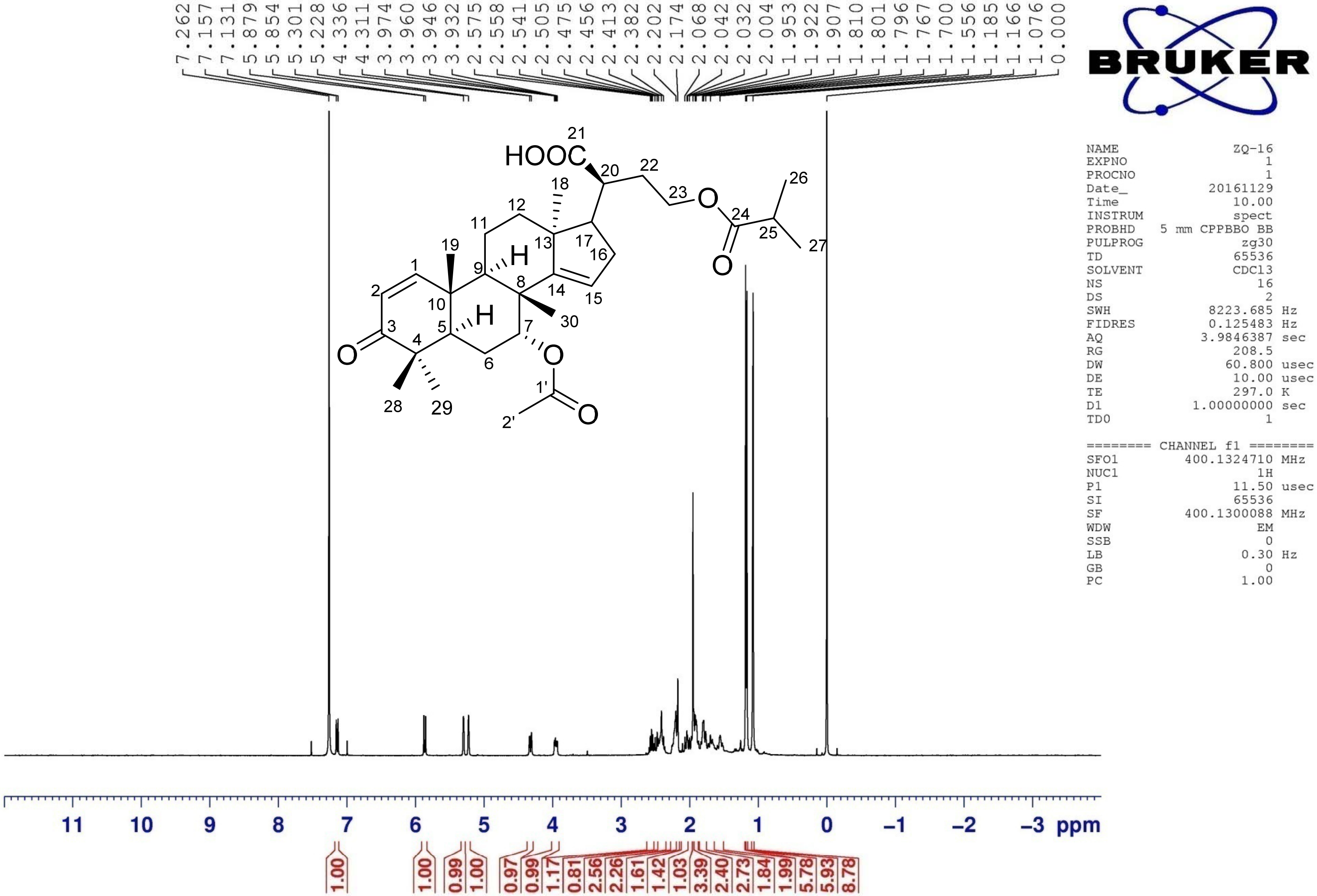

^{1}H NMR (400 MHz) spectrum of compound **10** in $CDCl_3$

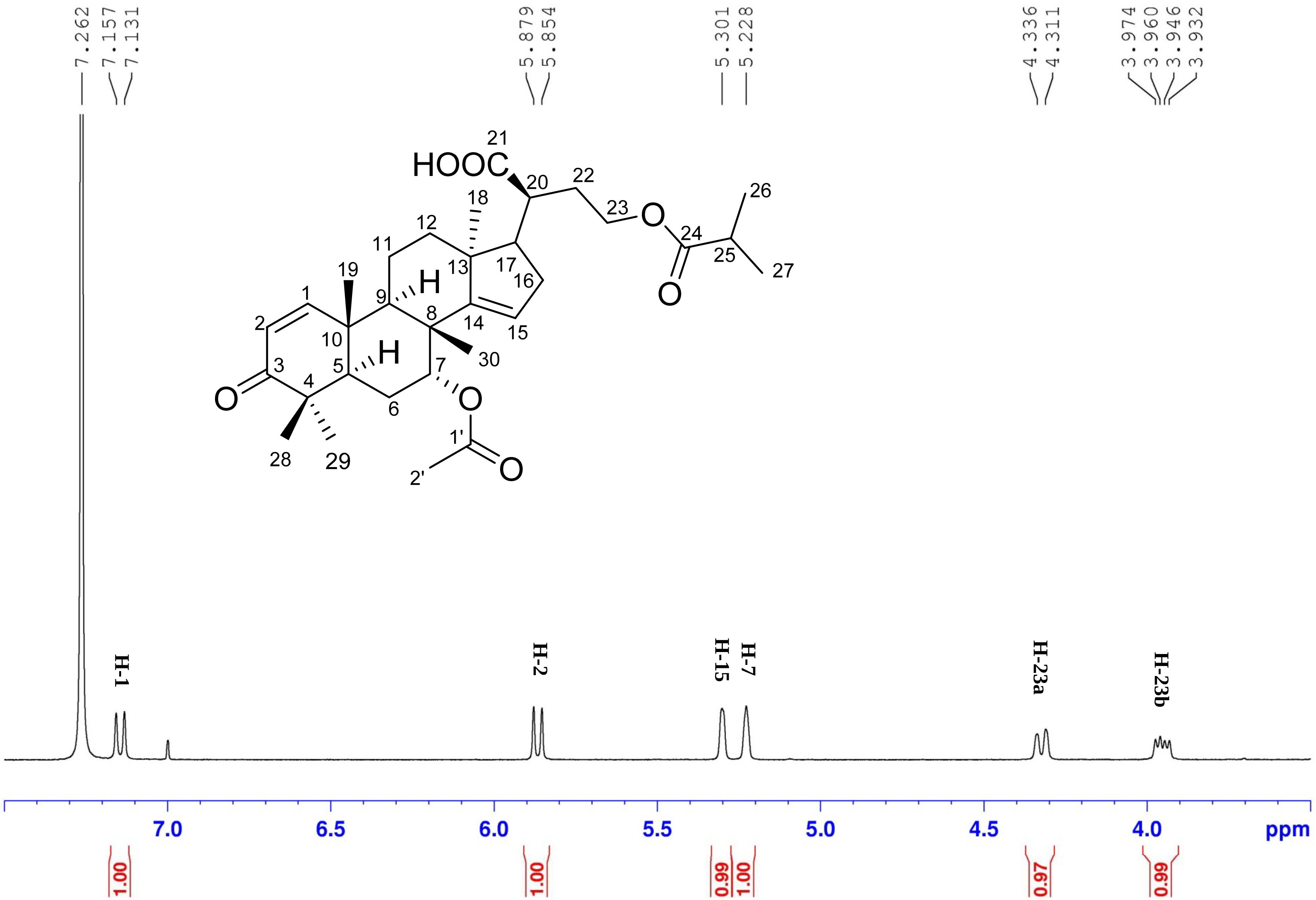

^{1}H NMR (400 MHz) spectrum of compound **10** in $CDCl_3$

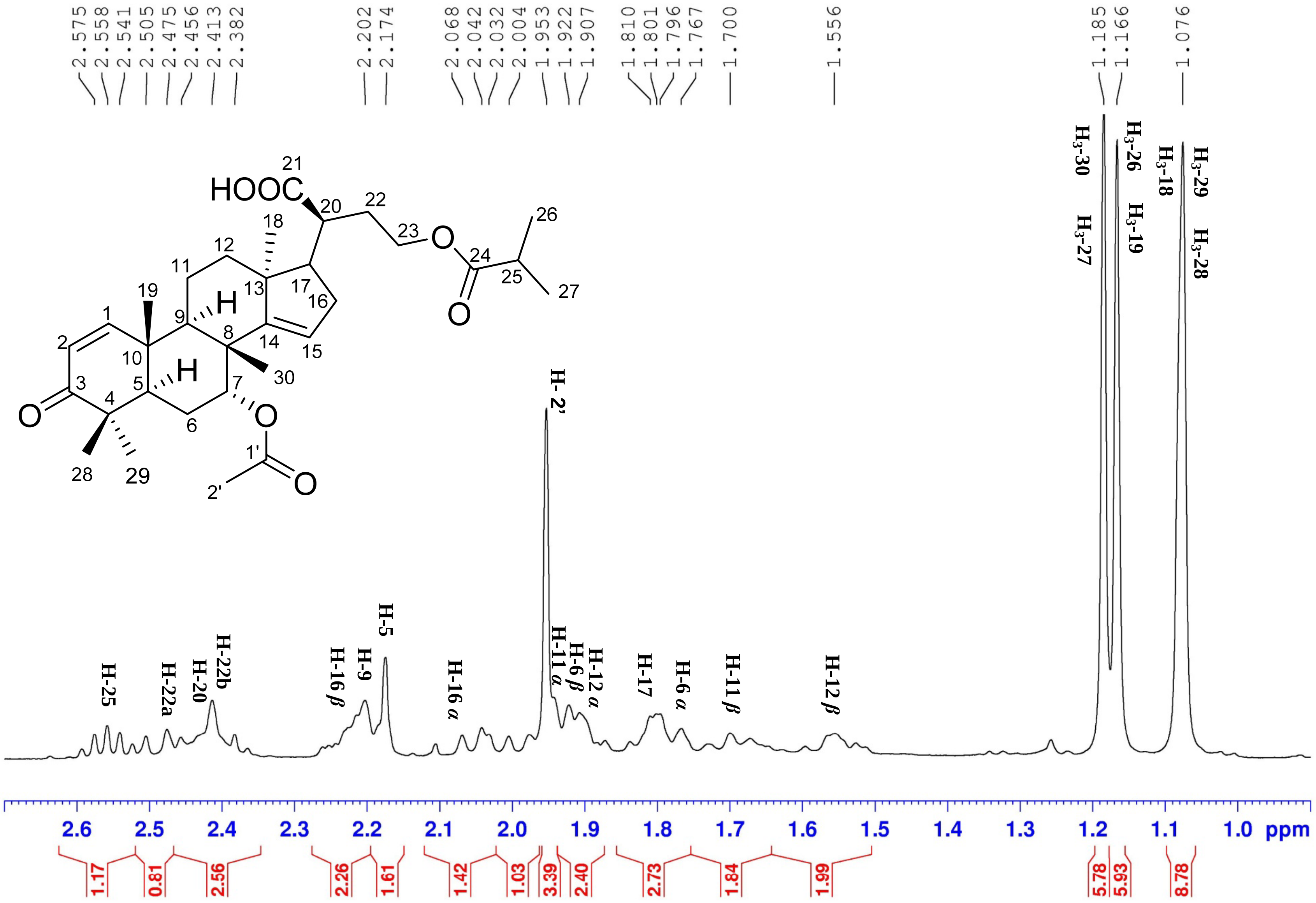

^{13}C NMR (100 MHz) spectrum of compound **10** in $CDCl_3$

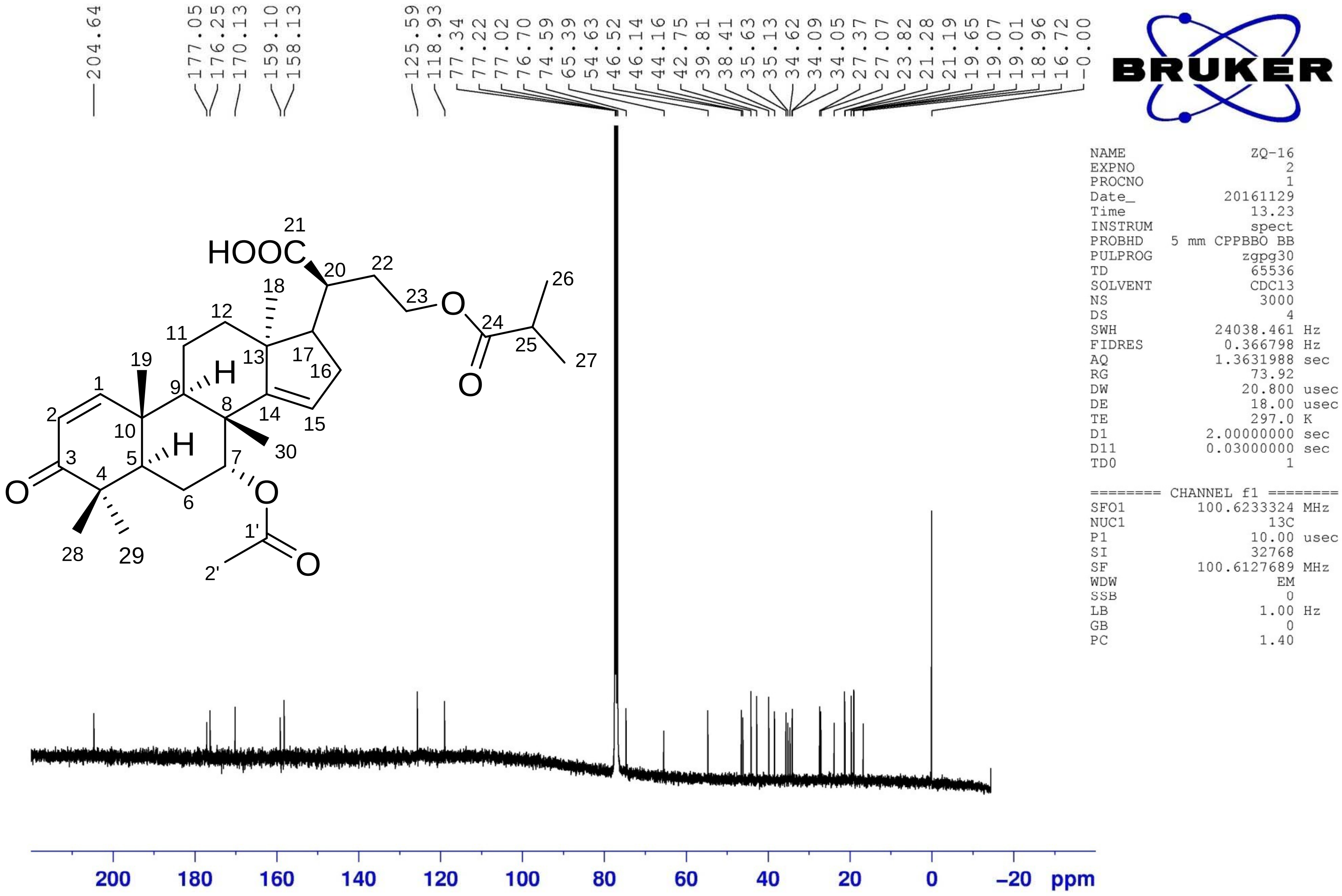

DEPT135 (100 MHz) spectrum of compound **10** in $CDCl_3$

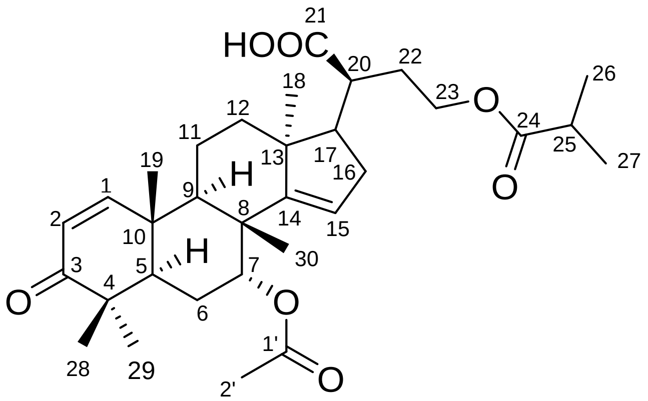

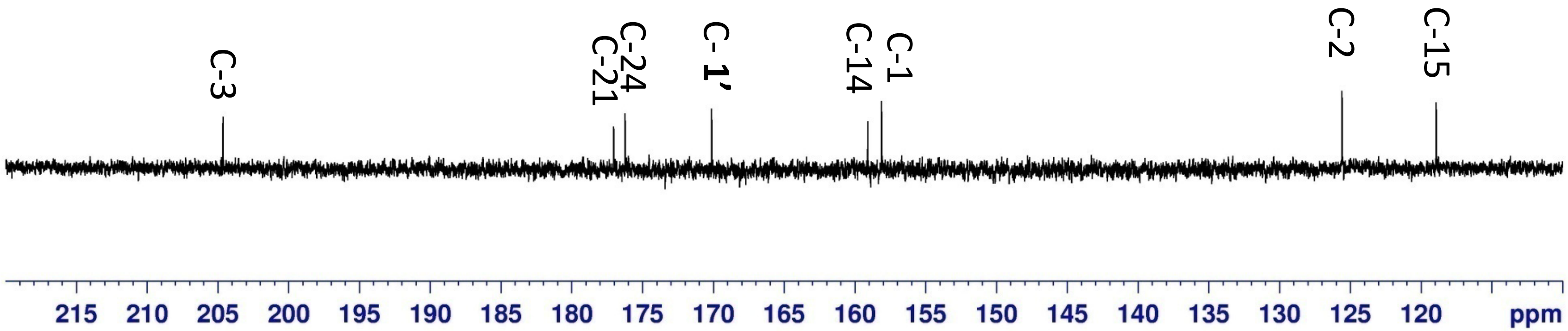

DEPT135 (100 MHz) spectrum of compound **10** in $CDCl_3$

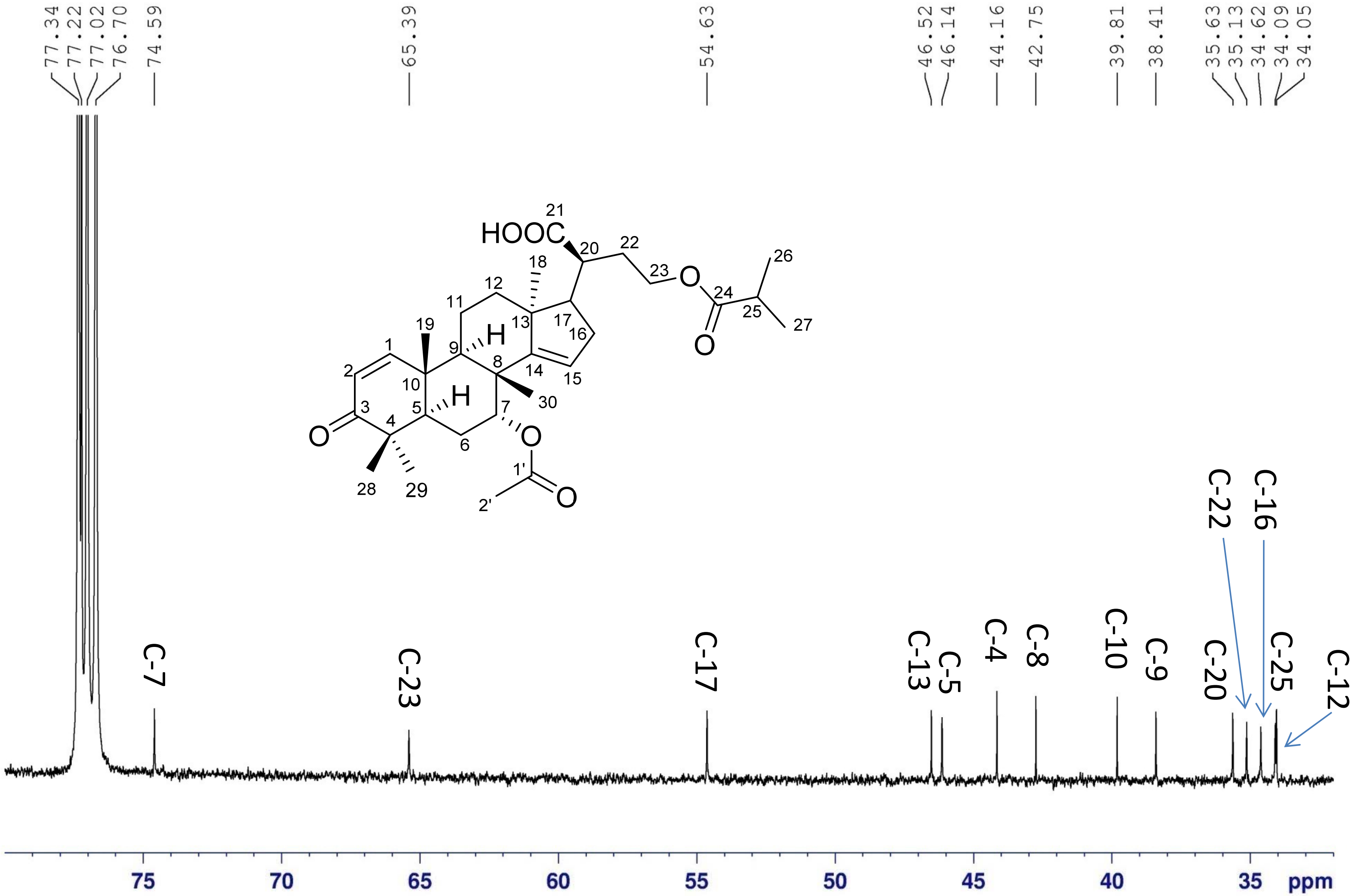

DEPT135 (100 MHz) spectrum of compound **10** in $CDCl_3$

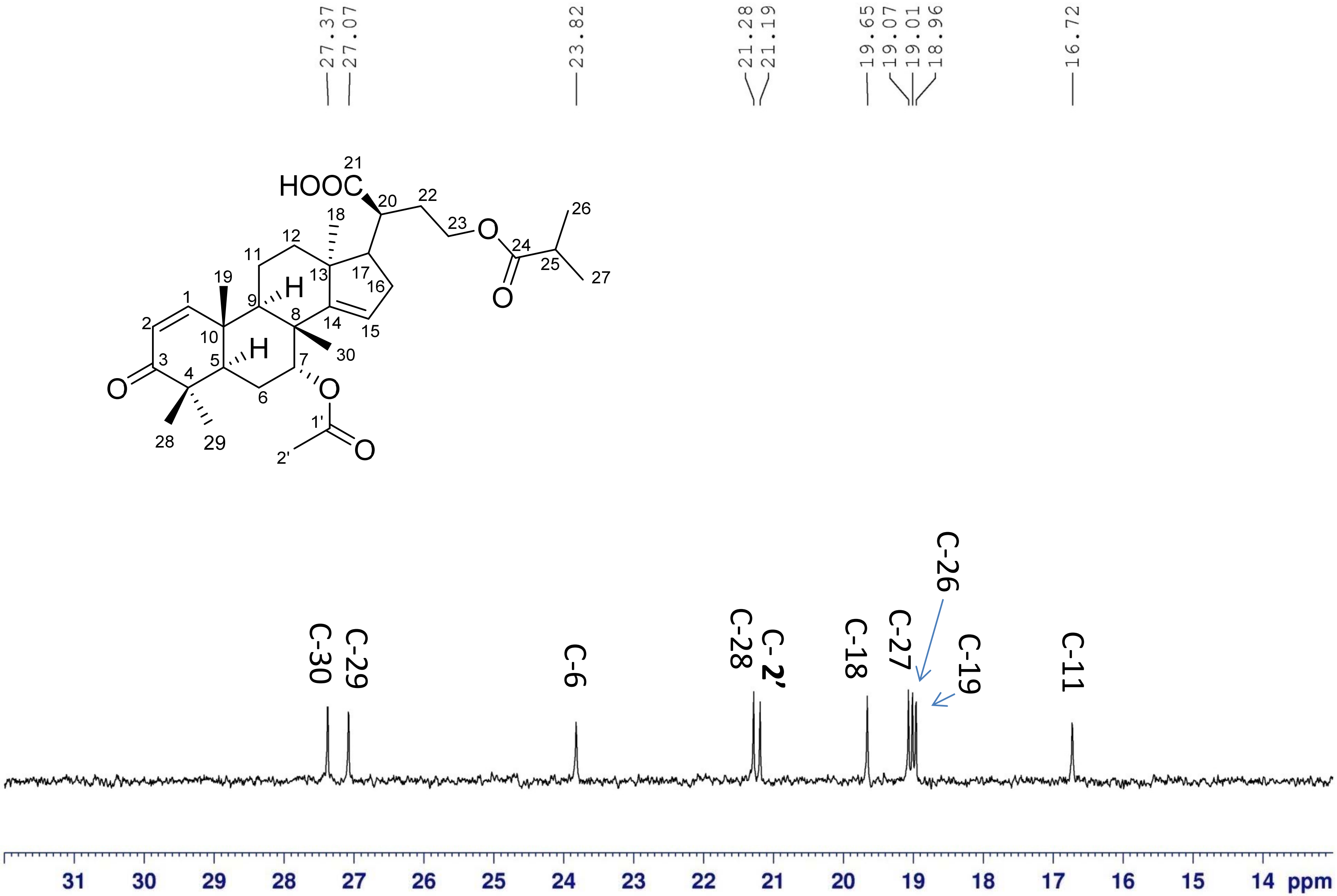

DEPT135 (100 MHz) spectrum of compound **10** in $CDCl_3$

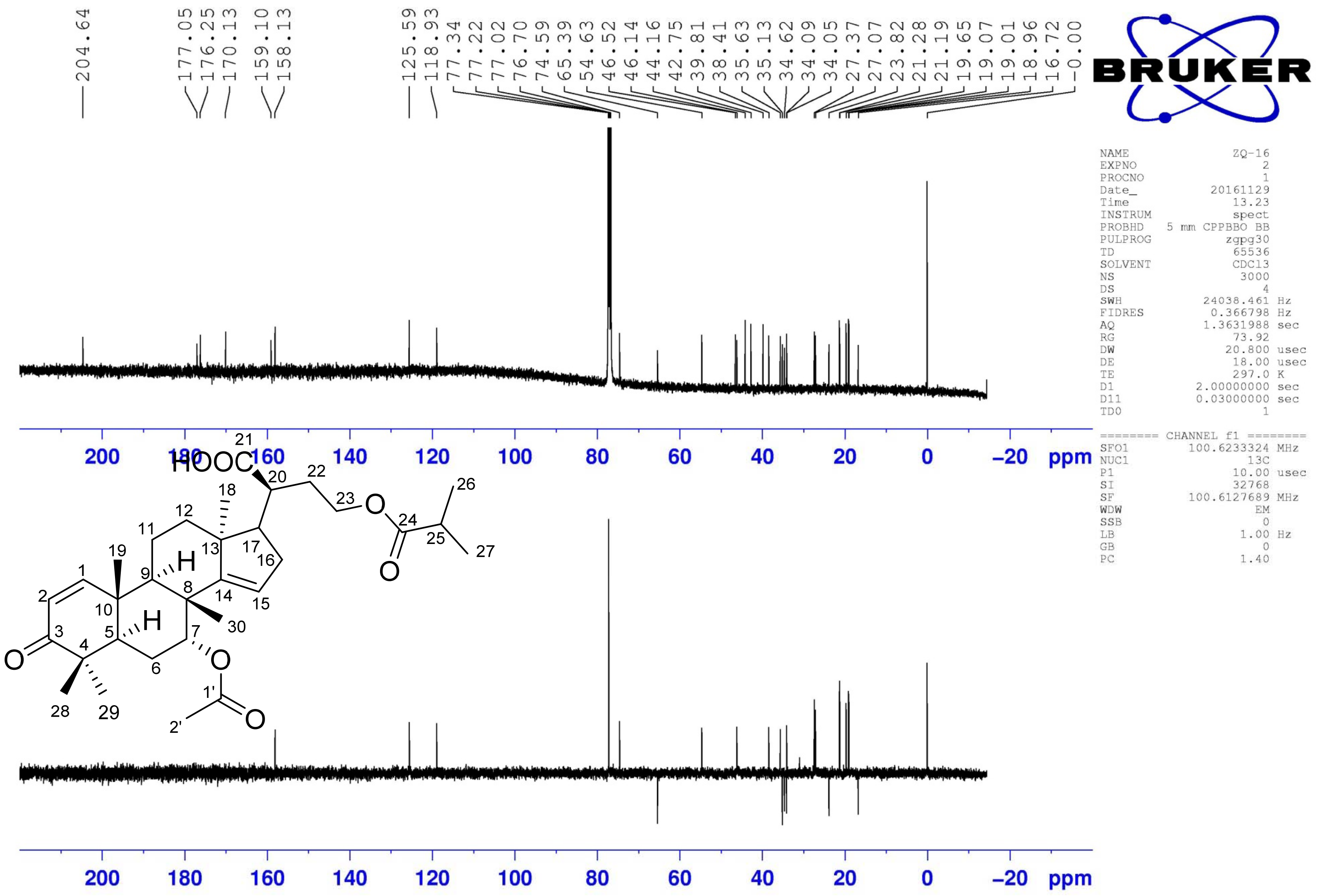

DEPT135 (100 MHz) spectrum of compound **10** in $CDCl_3$

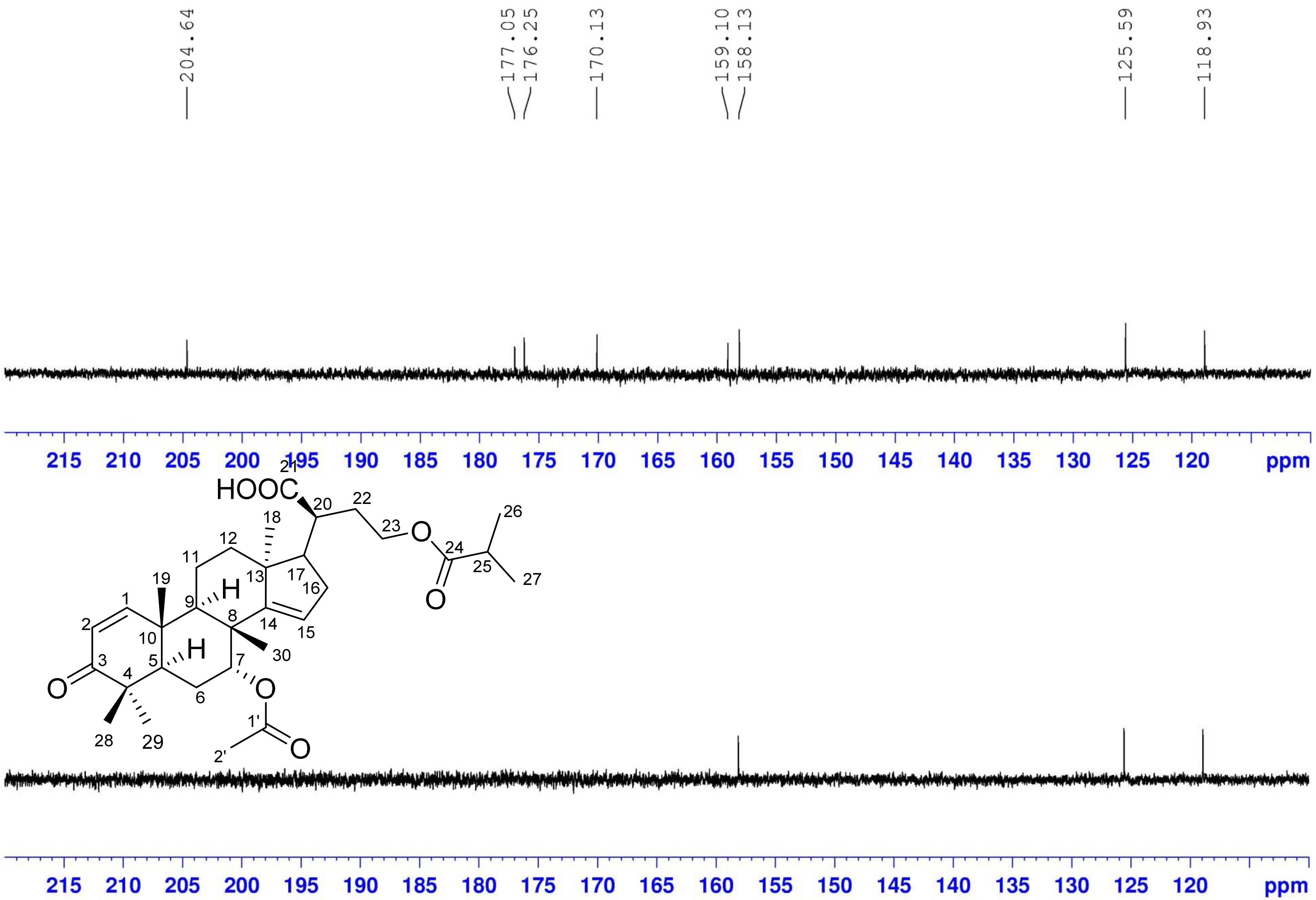

DEPT135 (100 MHz) spectrum of compound **10** in $CDCl_3$

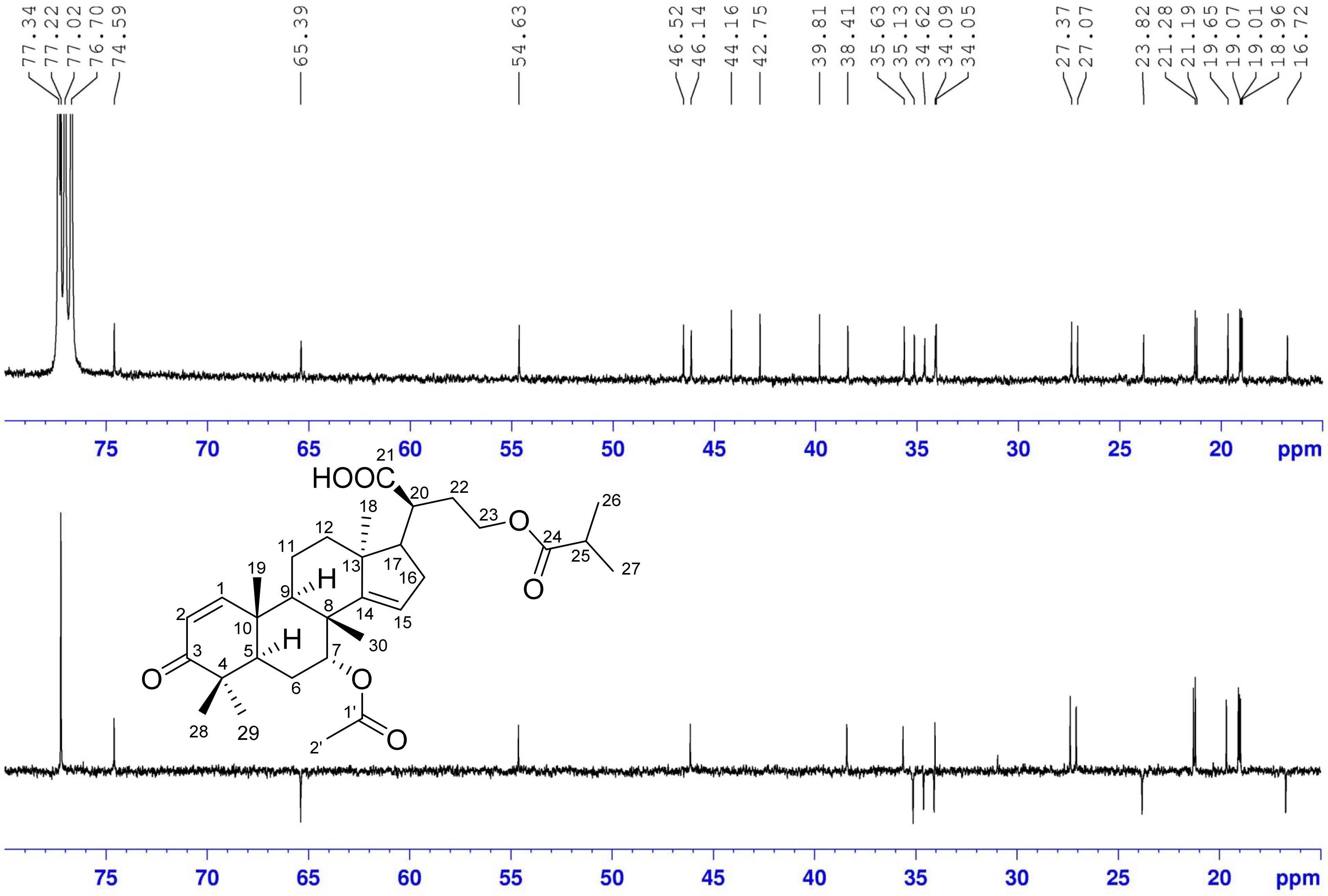

^{1}H-^{1}H COSY (400 MHz) spectrum of compound **10** in $CDCl_3$

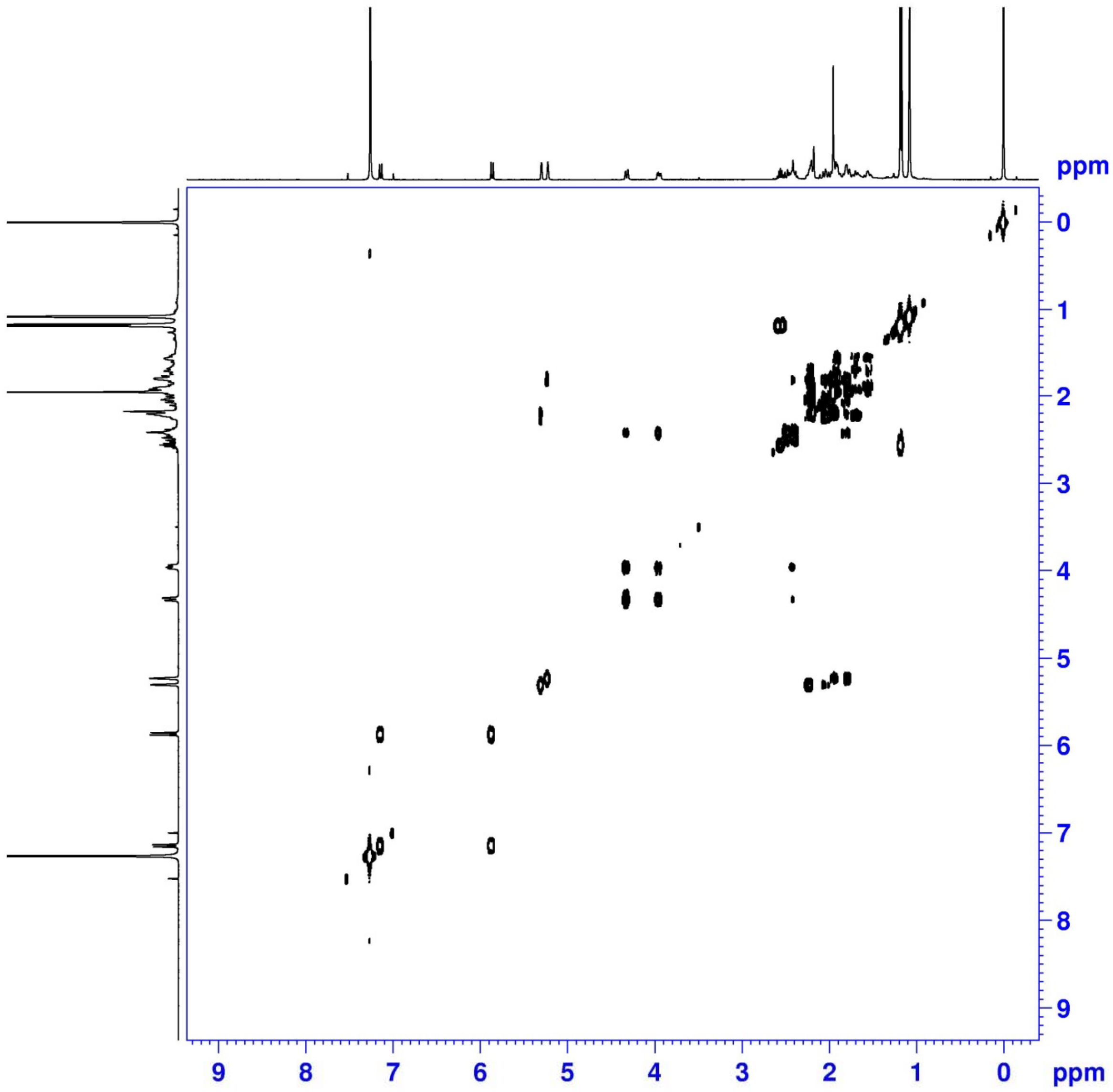


```
NAME                ZQ-16
EXPNO                   4
PROCNO                  1
Date_            20161203
Time                 8.34
INSTRUM             spect
PROBHD   5 mm CPPBBO BB
PULPROG        cosygpppqf
TD                   2048
SOLVENT             CDCl3
NS                      8
DS                      8
SWH              3906.250 Hz
FIDRES           1.907349 Hz
AQ              0.2621940 sec
RG                  208.5
DW                128.000 usec
DE                  10.00 usec
TE                  297.0 K
D0             0.00000300 sec
D1             1.89678097 sec
D11            0.03000000 sec
D12            0.00002000 sec
D13            0.00000400 sec
D16            0.00020000 sec
IN0            0.00025600 sec

======== CHANNEL f1 ========
SFO1          400.1318006 MHz
NUC1                   1H
P0                  11.50 usec
P1                  11.50 usec
P17               2500.00 usec
ND0                     1
TD                    128
SFO1             400.1318 MHz
FIDRES          30.517578 Hz
SW                  9.762 ppm
FnMODE                 QF
SI                   1024
SF            400.1300074 MHz
WDW                 QSINE
SSB                     0
LB                   0.00 Hz
GB                      0
PC                   1.40
SI                   1024
MC2                    QF
SF            400.1300054 MHz
WDW                 QSINE
SSB                     0
LB                   0.00 Hz
[illegible]
```

^{1}H-^{1}H COSY (400 MHz) spectrum of compound **10** in $CDCl_3$

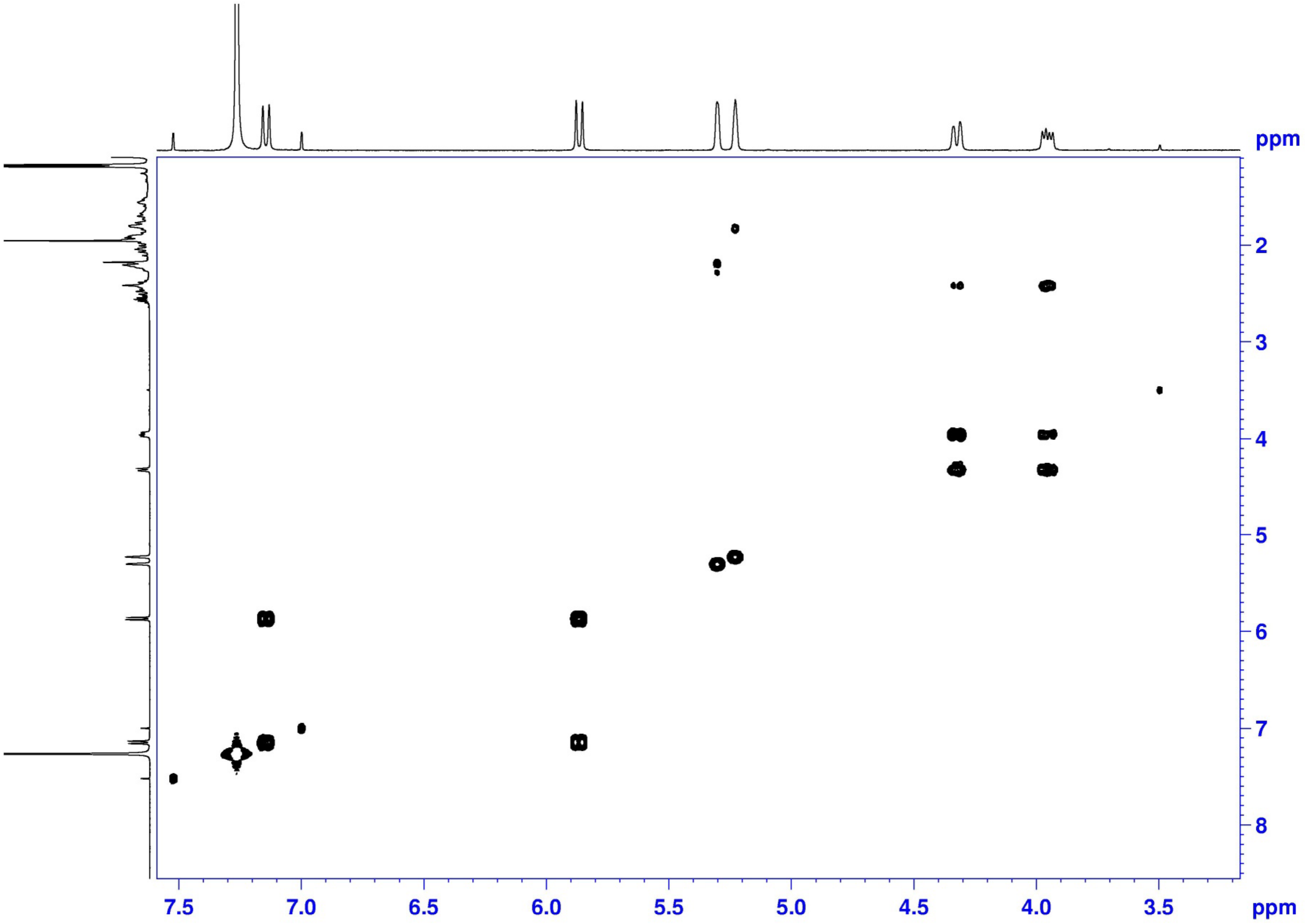

^{1}H-^{1}H COSY (400 MHz) spectrum of compound **10** in $CDCl_3$

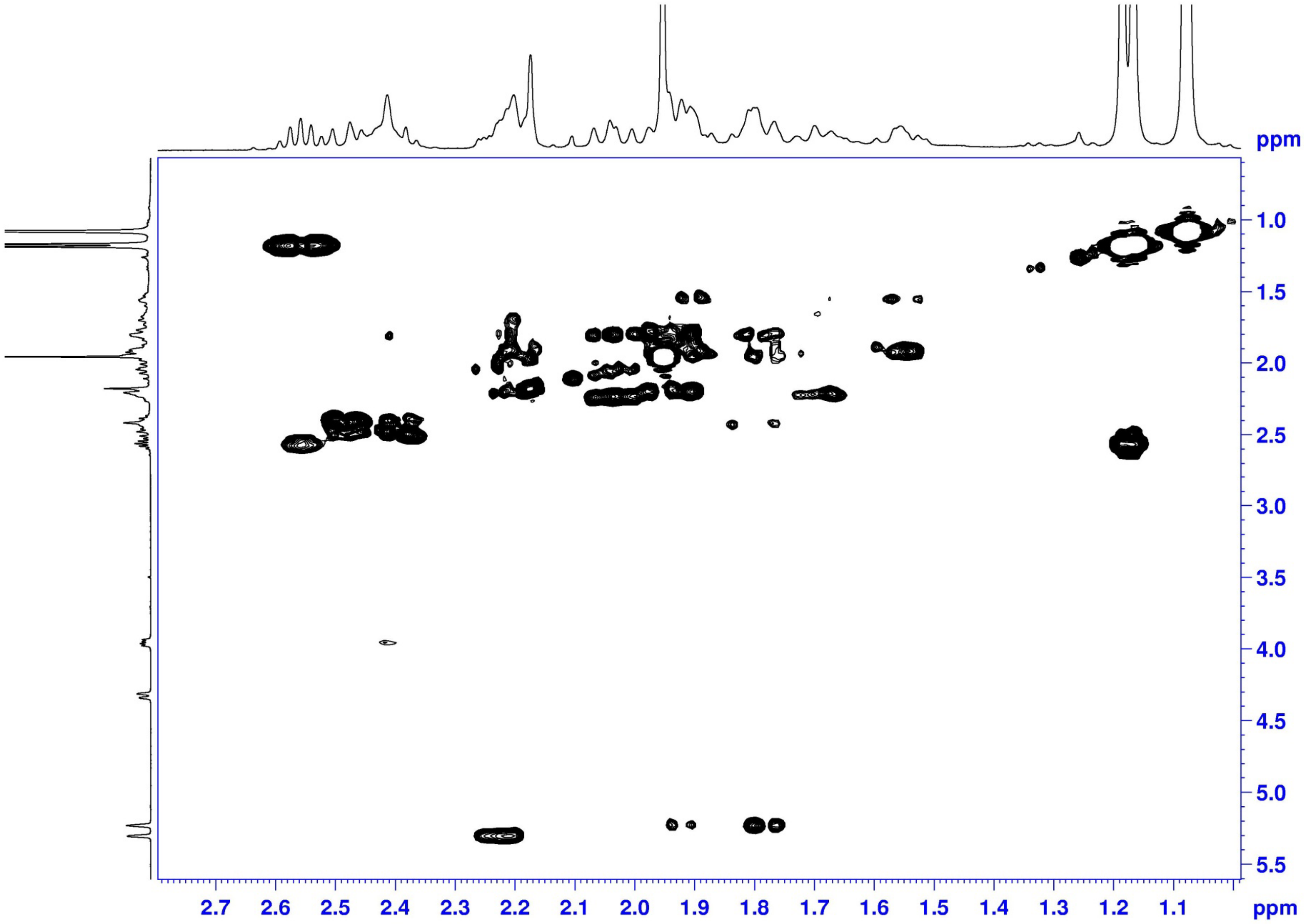

HSQC (400 MHz) spectrum of compound **10** in $CDCl_3$

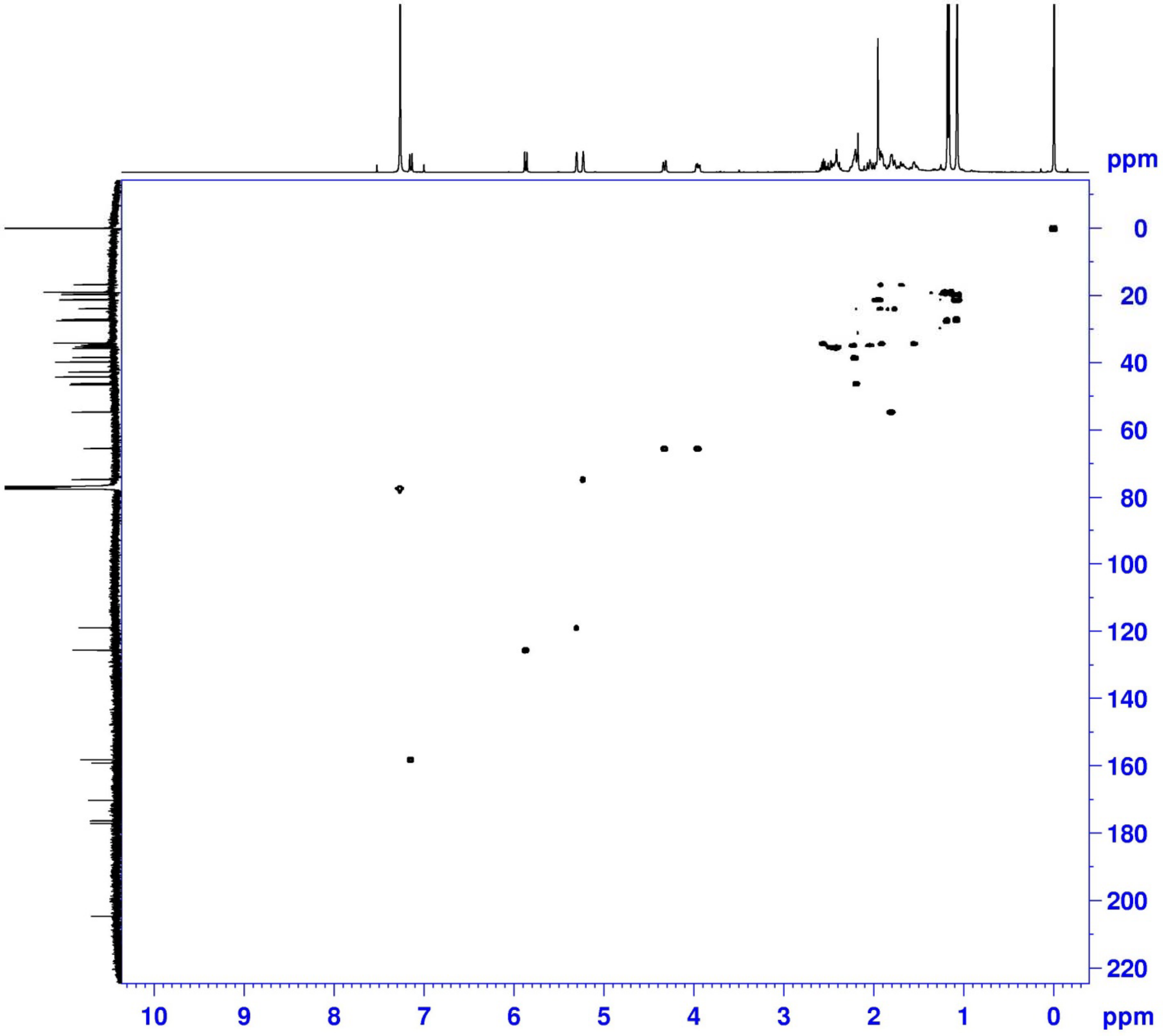

NAME	ZQ-16	
EXPNO	5	
PROCNO	1	
Date_	20161203	
Time	9.13	
INSTRUM	spect	
PROBHD	5 mm CPPBBO BB	
PULPROG	hsqcetgpsi2	
TD	1024	
SOLVENT	CDCl3	
NS	16	
DS	16	
SWH	4302.926	Hz
FIDRES	4.202076	Hz
AQ	0.1190388	sec
RG	208.5	
DW	116.200	usec
DE	10.00	usec
TE	297.0	K
CNST2	145.0000000	
D0	0.00000300	sec
D1	1.46497905	sec
D4	0.00172414	sec
D11	0.03000000	sec
D16	0.00020000	sec
D24	0.00086207	sec
IN0	0.00002080	sec
ZGOPTNS		
======== CHANNEL f1 ========		
SFO1	400.1320007	MHz
NUC1	1H	
P1	11.50	usec
P2	23.00	usec
P28	0.00	usec
ND0	2	
TD	256	
SFO1	100.6233	MHz
FIDRES	93.900238	Hz
SW	238.896	ppm
FnMODE	Echo-Antiecho	
SI	1024	
SF	400.1300066	MHz
WDW	QSINE	
SSB	2	
LB	0.00	Hz
GB	0	
PC	1.40	
SI	1024	
MC2	echo-antiecho	
SF	100.6127567	MHz
WDW	QSINE	

HSQC (400 MHz) spectrum of compound **10** in $CDCl_3$

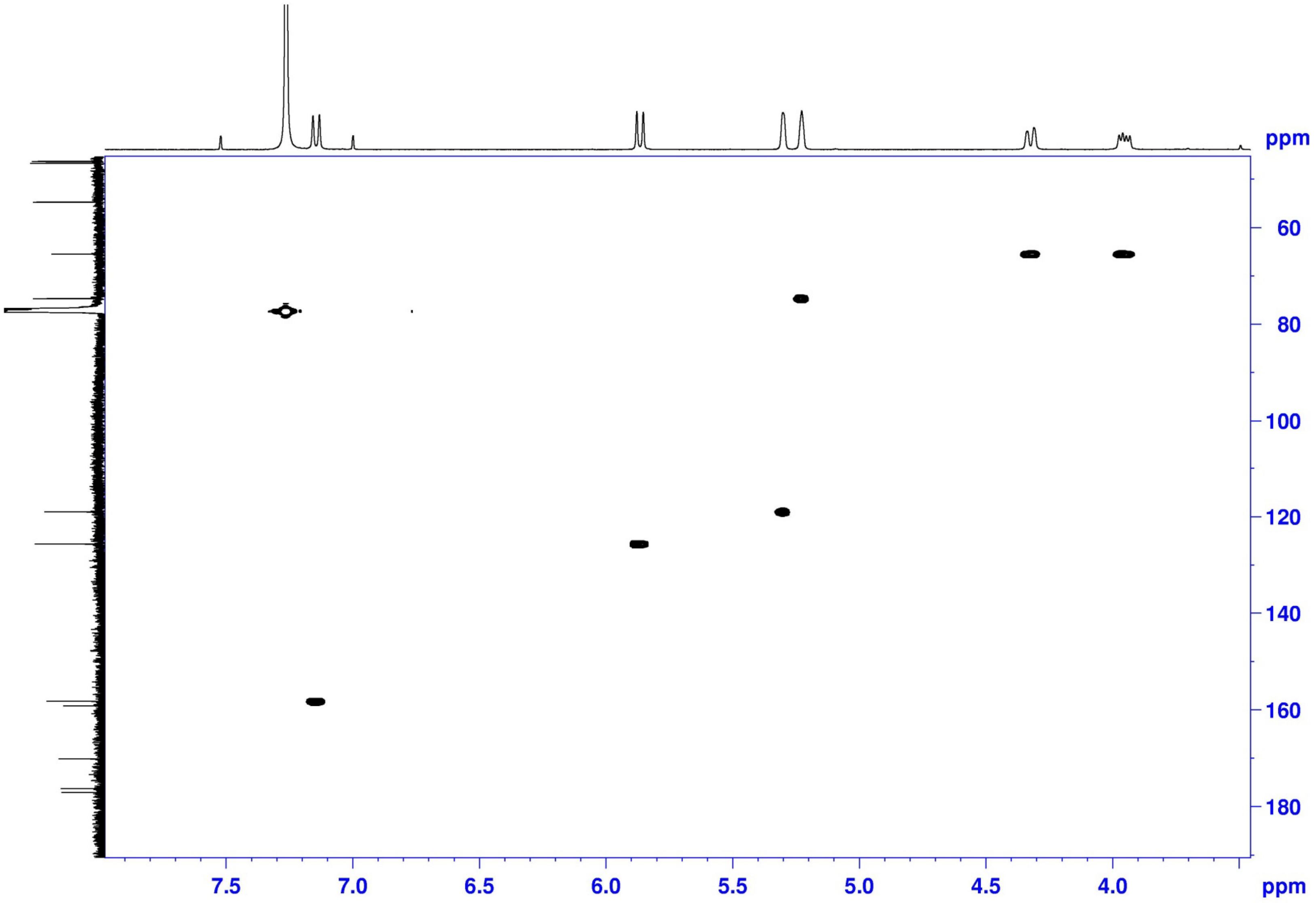

HSQC (400 MHz) spectrum of compound **10** in $CDCl_3$

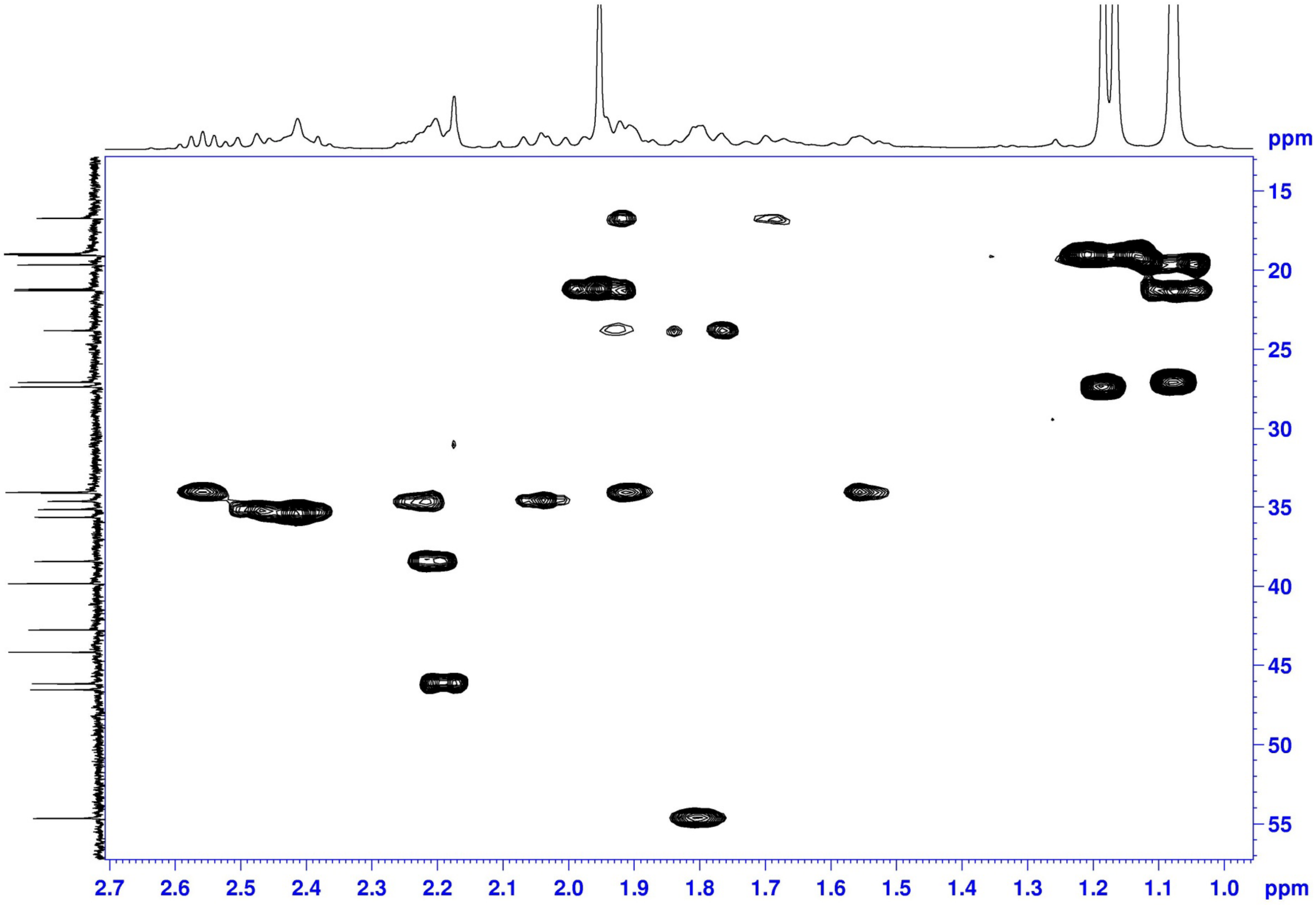

HMBC (400 MHz) spectrum of compound **10** in $CDCl_3$

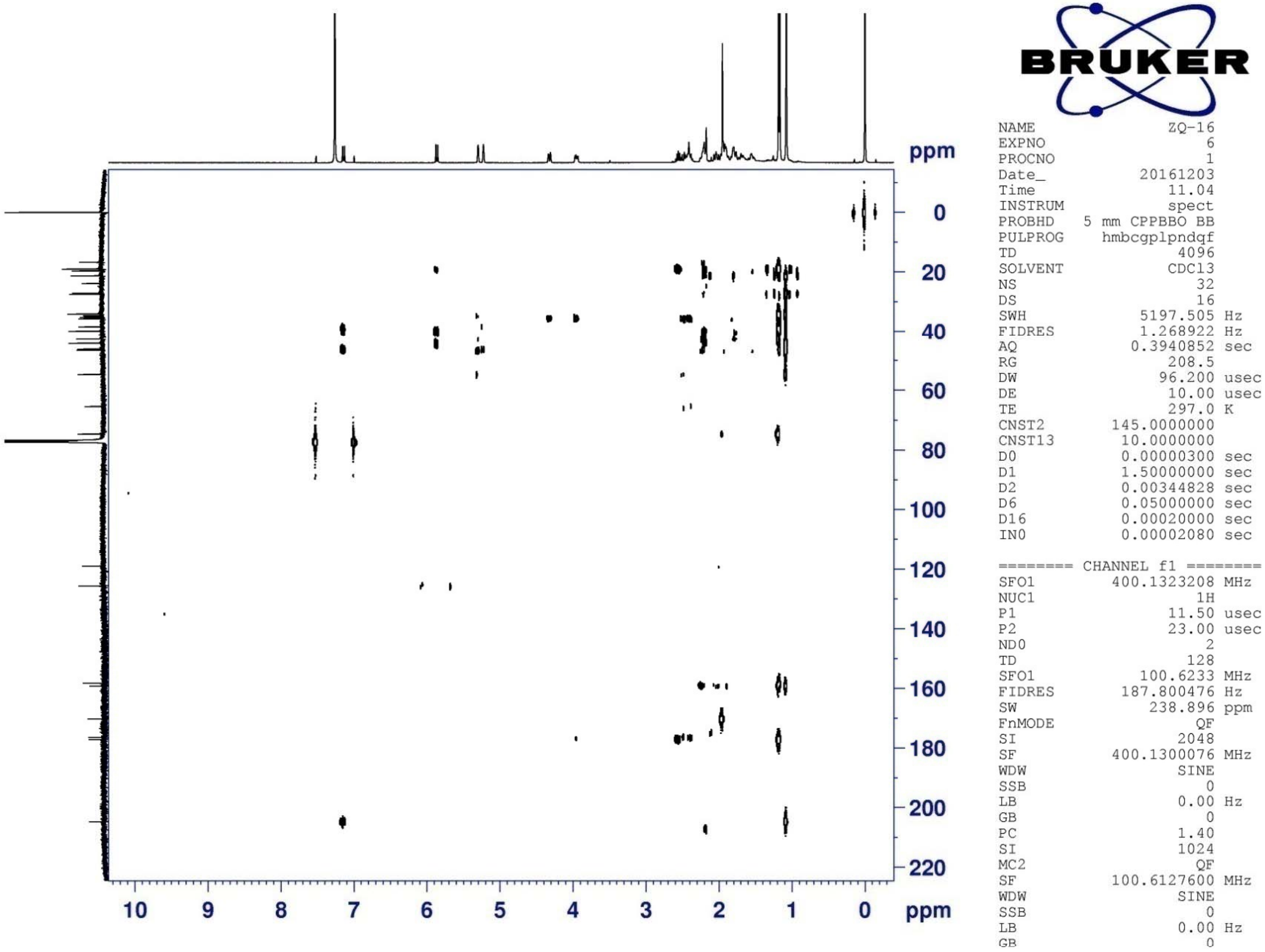

HMBC (400 MHz) spectrum of compound **10** in $CDCl_3$

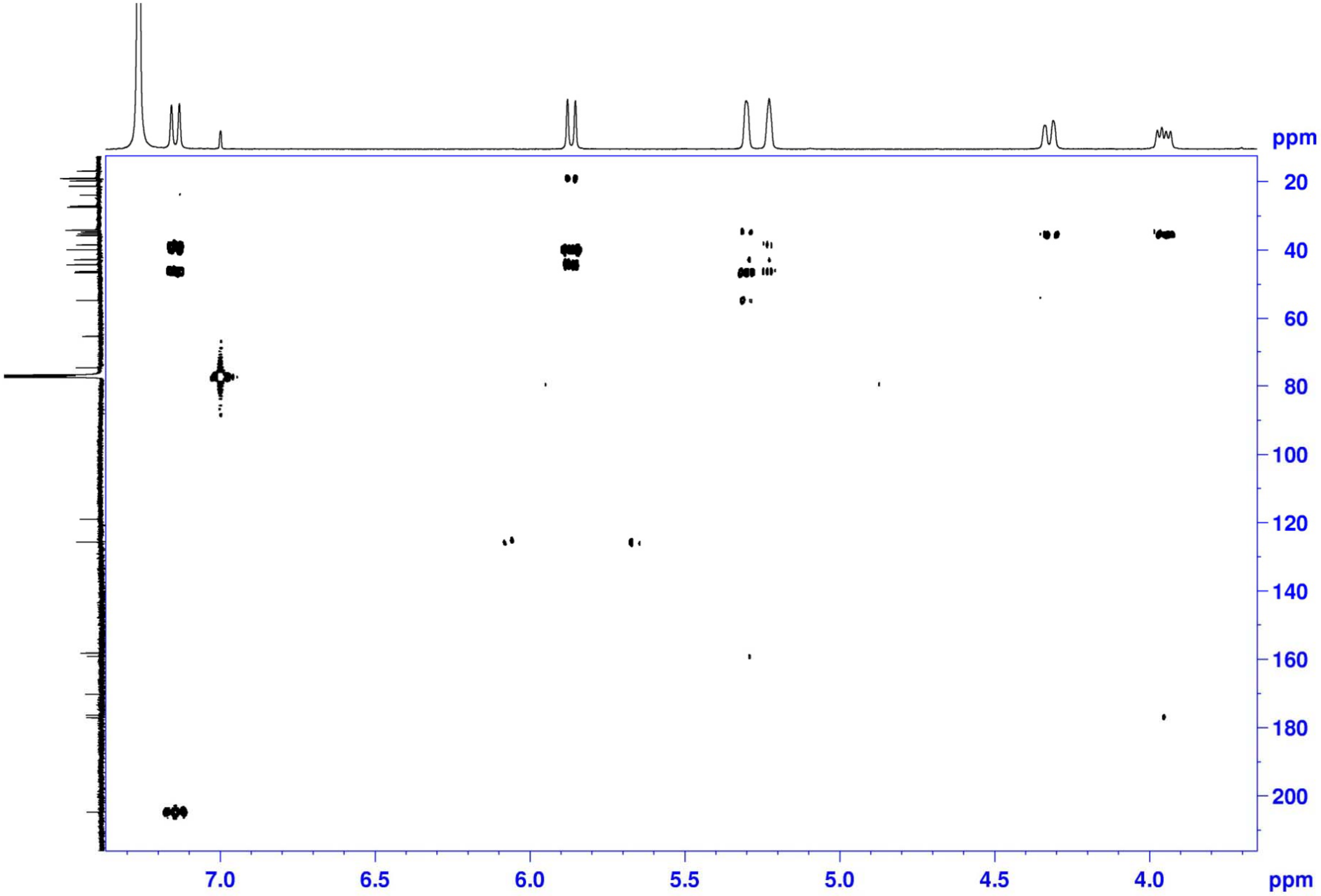

HMBC (400 MHz) spectrum of compound **10** in $CDCl_3$

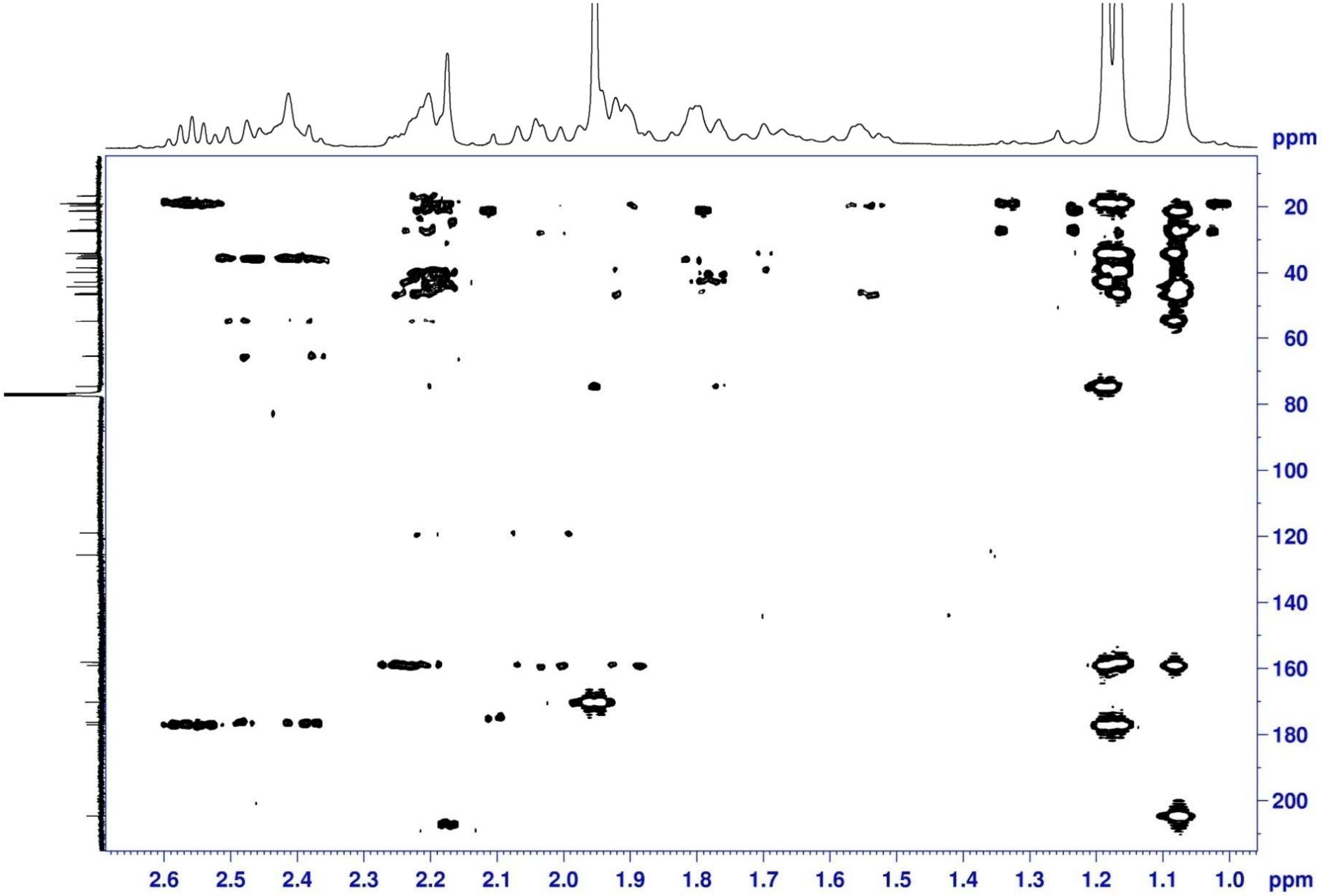

HMBC (400 MHz) spectrum of compound **10** in $CDCl_3$

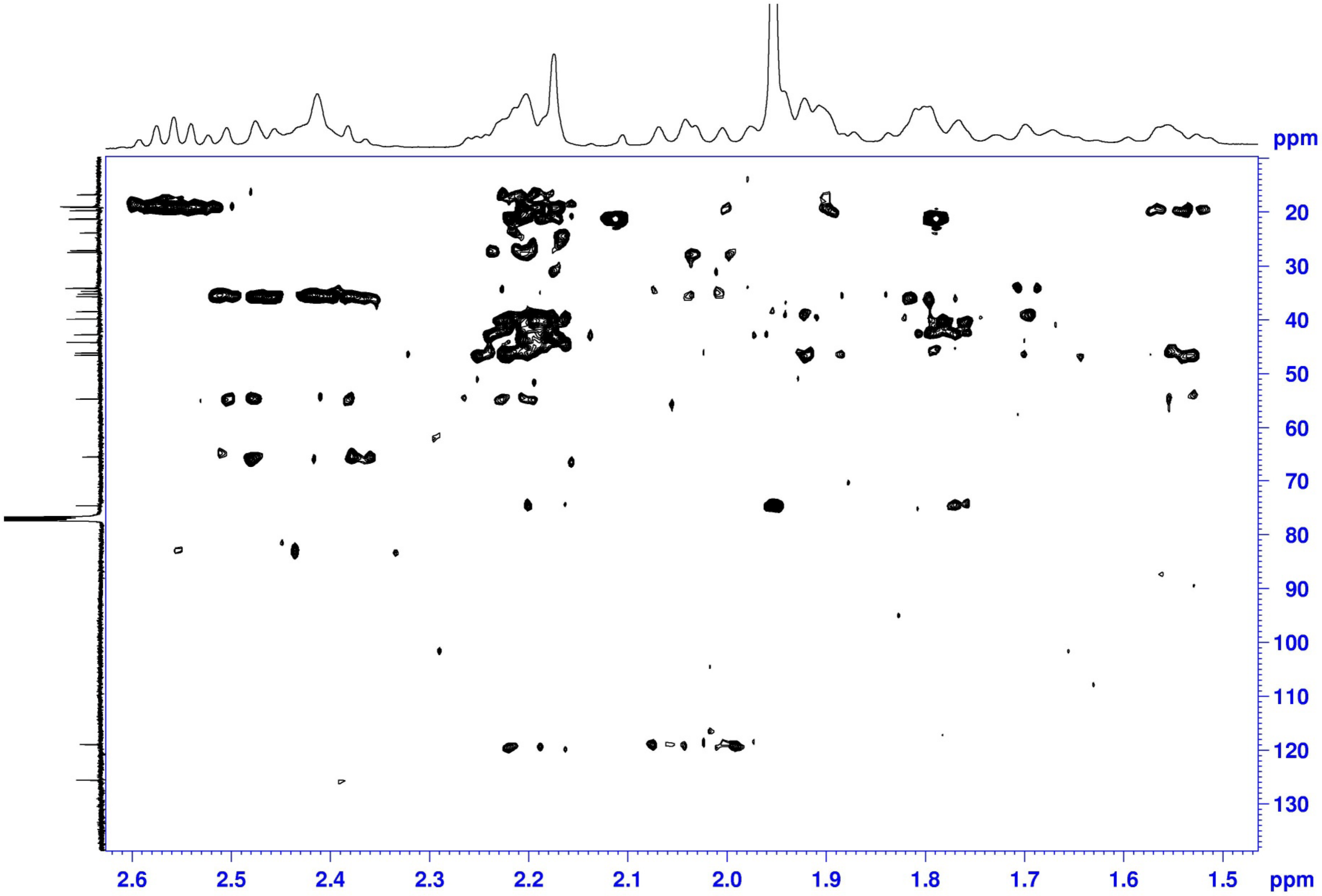

HMBC (400 MHz) spectrum of compound **10** in $CDCl_3$

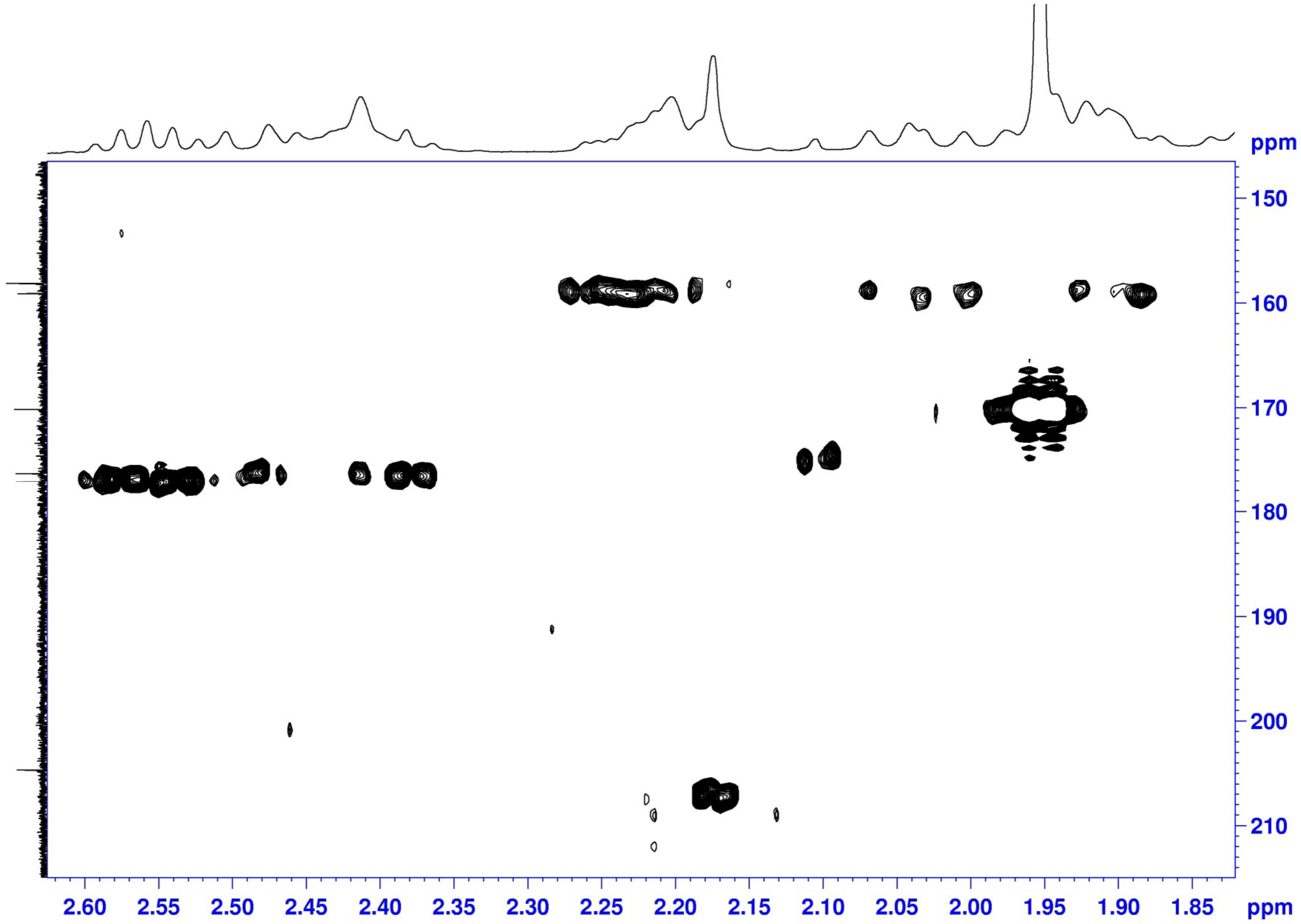

HMBC (400 MHz) spectrum of compound **10** in $CDCl_3$

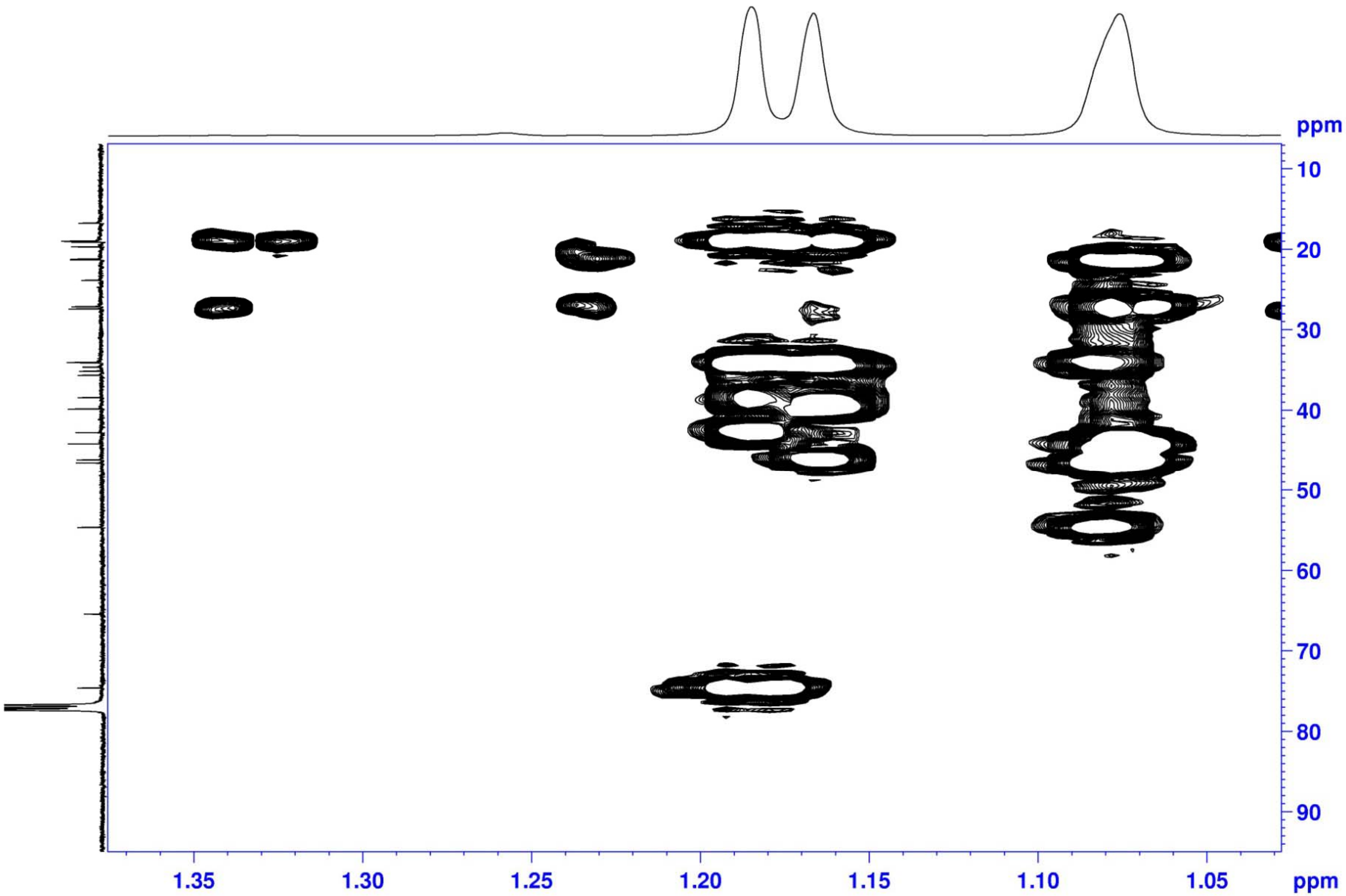

HMBC (400 MHz) spectrum of compound **10** in $CDCl_3$

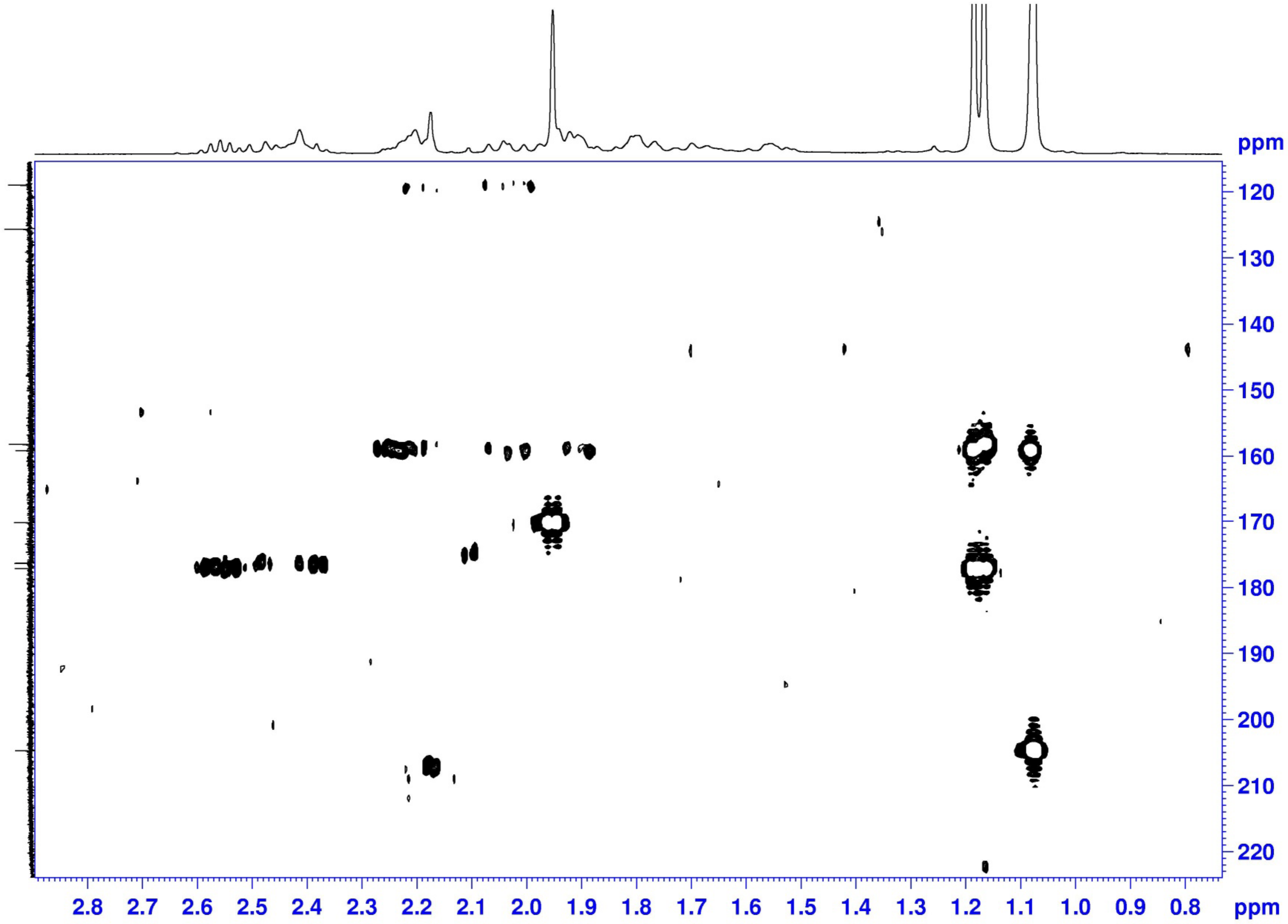

NOESY (400 MHz) spectrum of compound **10** in $CDCl_3$

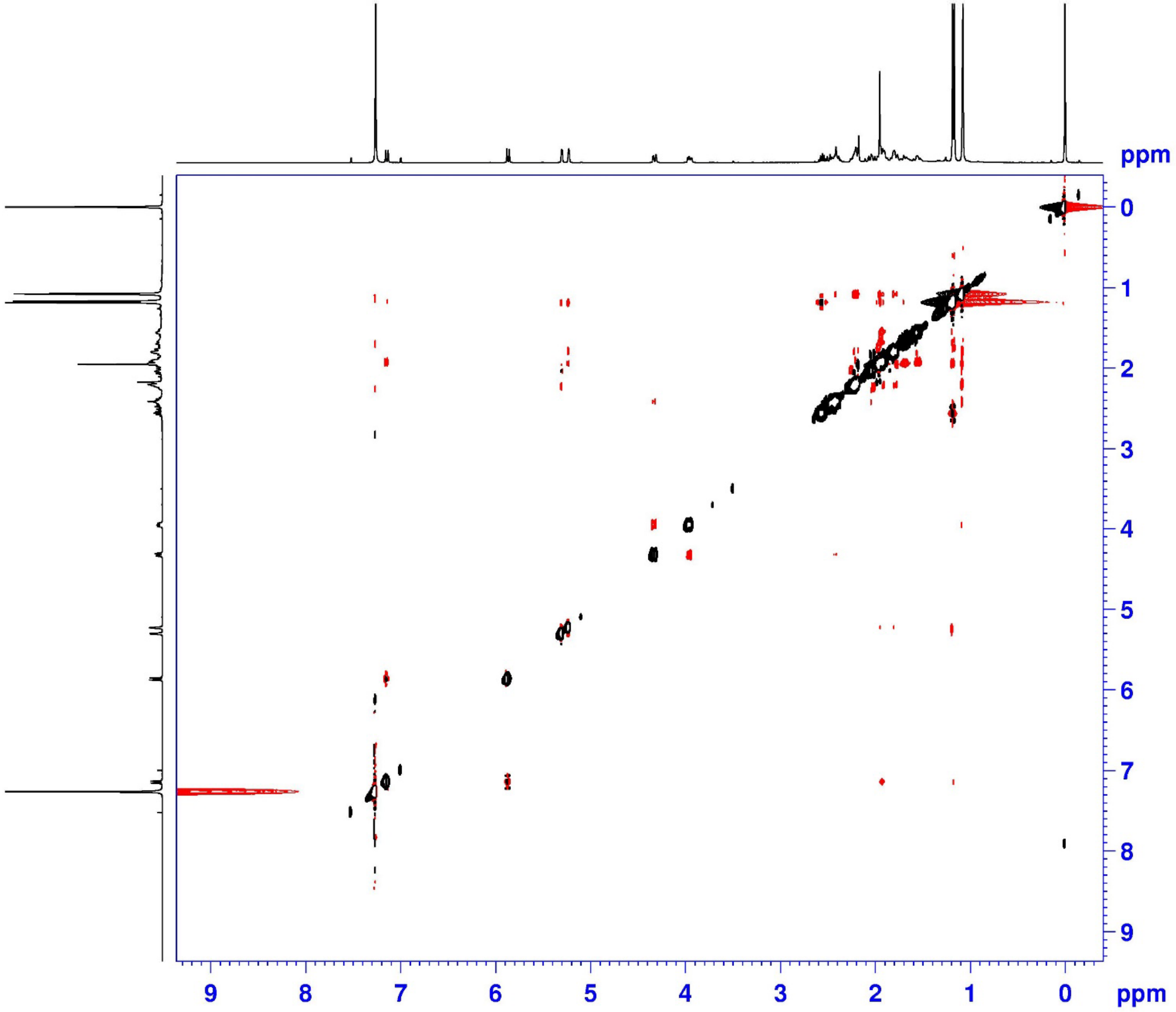


```
NAME                ZQ-16
EXPNO                   7
PROCNO                  1
Date_            20161203
Time                13.20
INSTRUM             spect
PROBHD   5 mm CPPBBO BB
PULPROG      noesygpphpp
TD                   2048
SOLVENT             CDCl3
NS                     16
DS                     32
SWH              4000.000 Hz
FIDRES           1.953125 Hz
AQ              0.2560500 se
RG                  208.5
DW                125.000 us
DE                  10.00 us
TE                  297.0 K
D0             0.00011036 se
D1             1.99385595 se
D8             0.30000001 se
D11            0.03000000 se
D12            0.00002000 se
D16            0.00020000 se
IN0            0.00025000 se

======== CHANNEL f1 ======
SFO1          400.1318006 MH
NUC1                   1H
P1                  11.50 us
P2                  23.00 us
P17               2500.00 us
ND0                     1
TD                    256
SFO1             400.1318 MH
FIDRES          15.625000 Hz
SW                  9.997 pp
FnMODE         States-TPPI
SI                   1024
SF            400.1300061 MH
WDW                 QSINE
SSB                     2
LB                   0.00 Hz
GB                      0
PC                   1.00
SI                   1024
MC2            States-TPPI
SF            400.1300088 MH
WDW                 QSINE
SSB                     2
```

NOESY (400 MHz) spectrum of compound **10** in $CDCl_3$

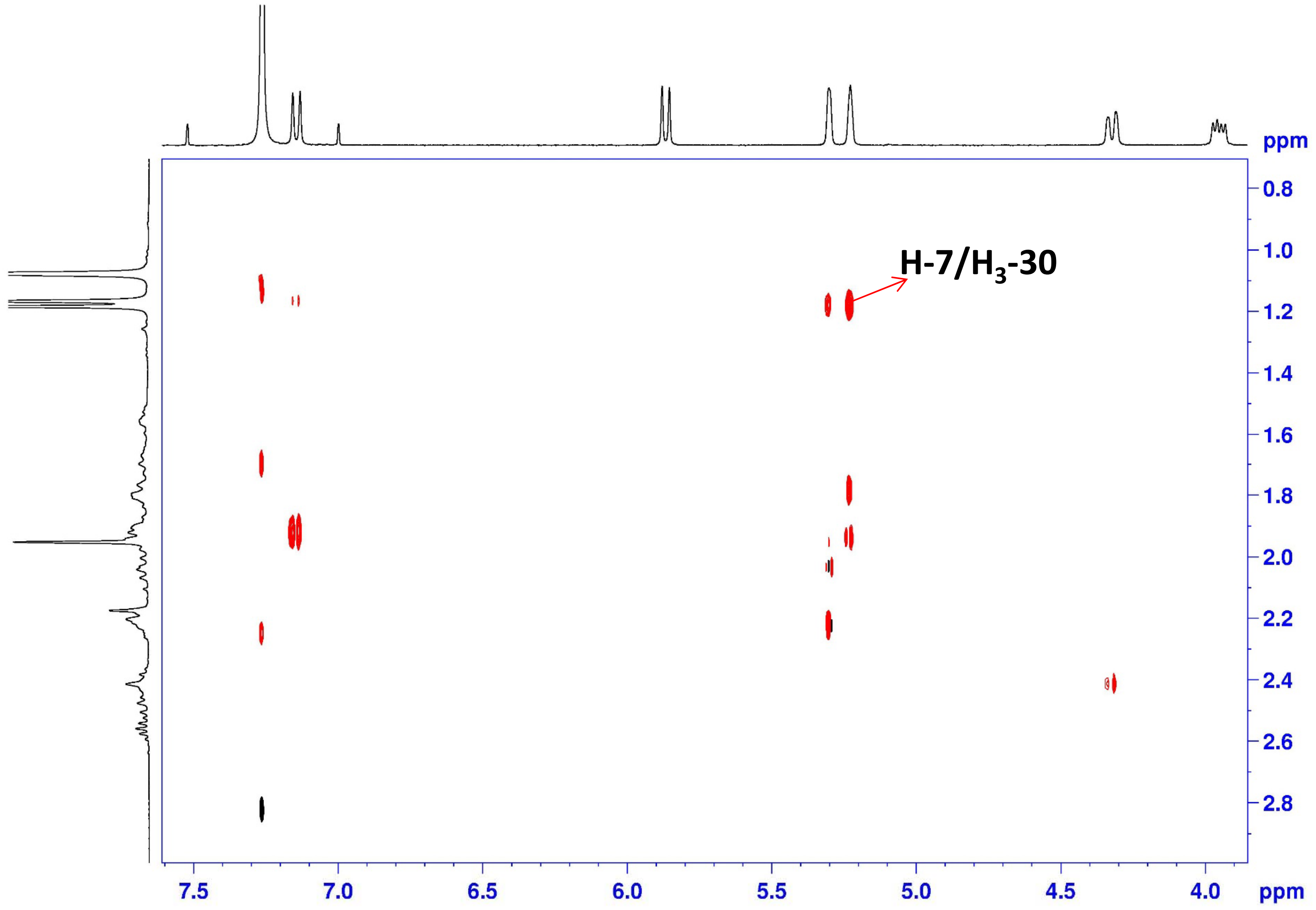

NOESY (400 MHz) spectrum of compound **10** in $CDCl_3$

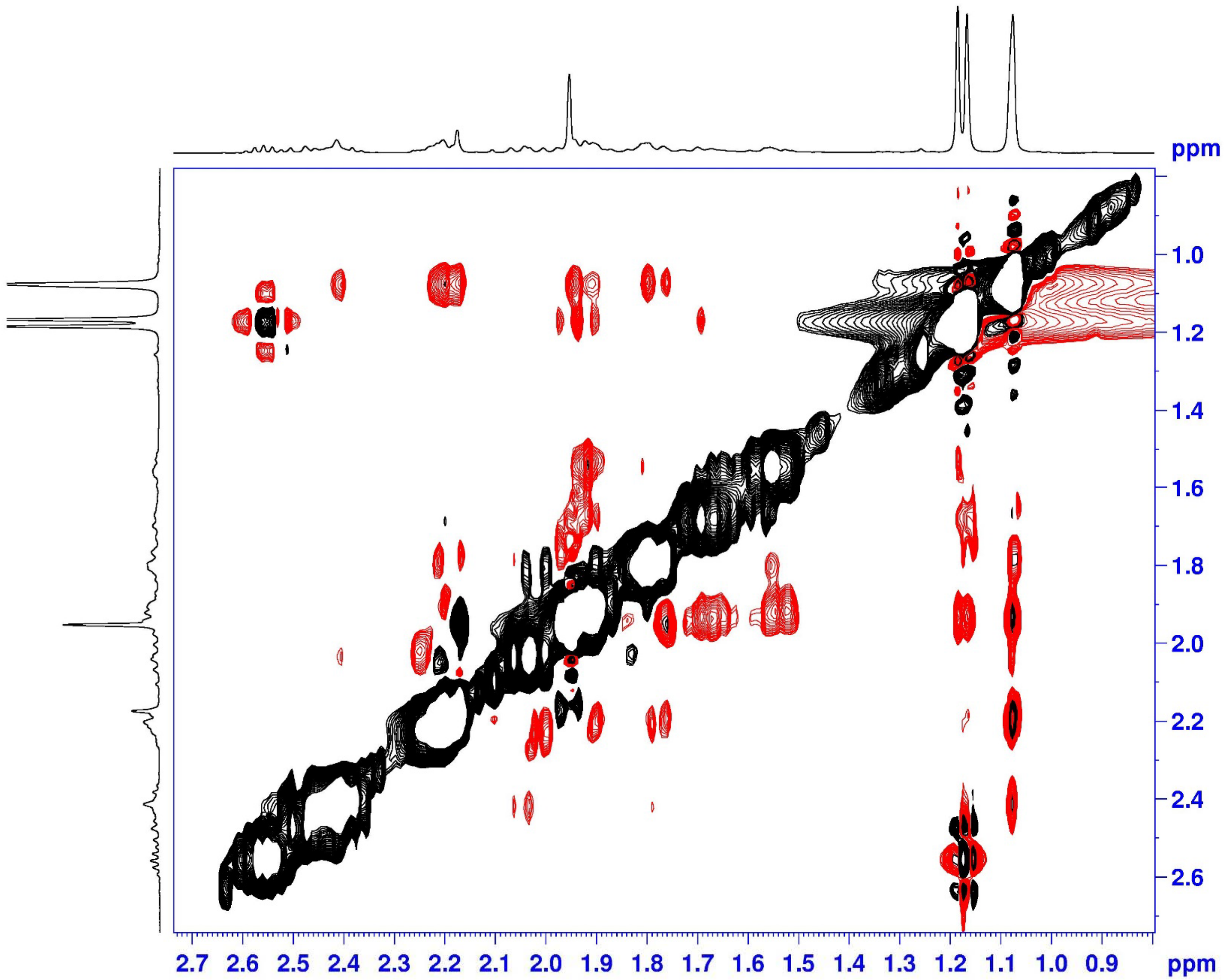

NOESY (400 MHz) spectrum of compound **10** in $CDCl_3$

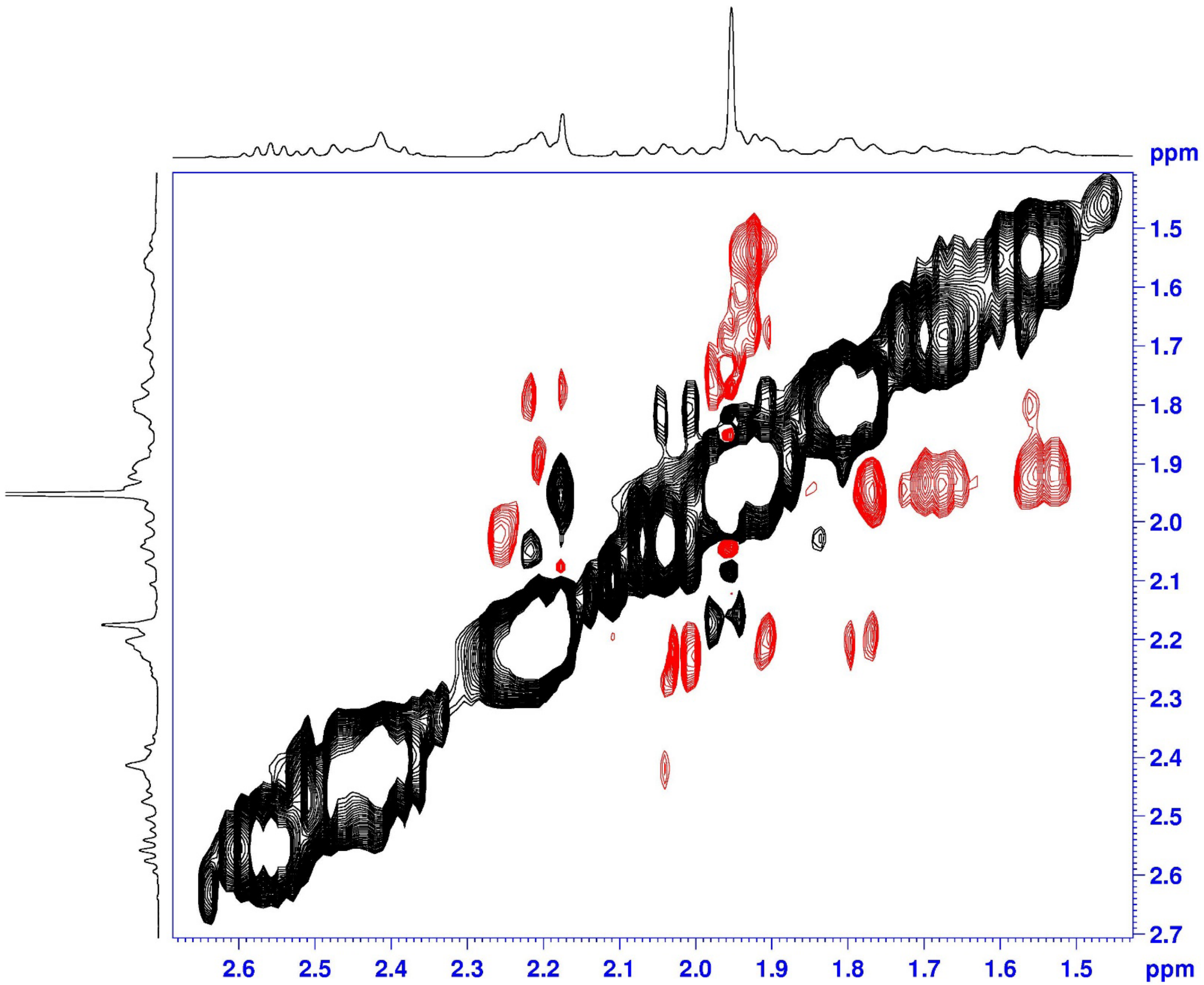

NOESY (400 MHz) spectrum of compound **10** in $CDCl_3$

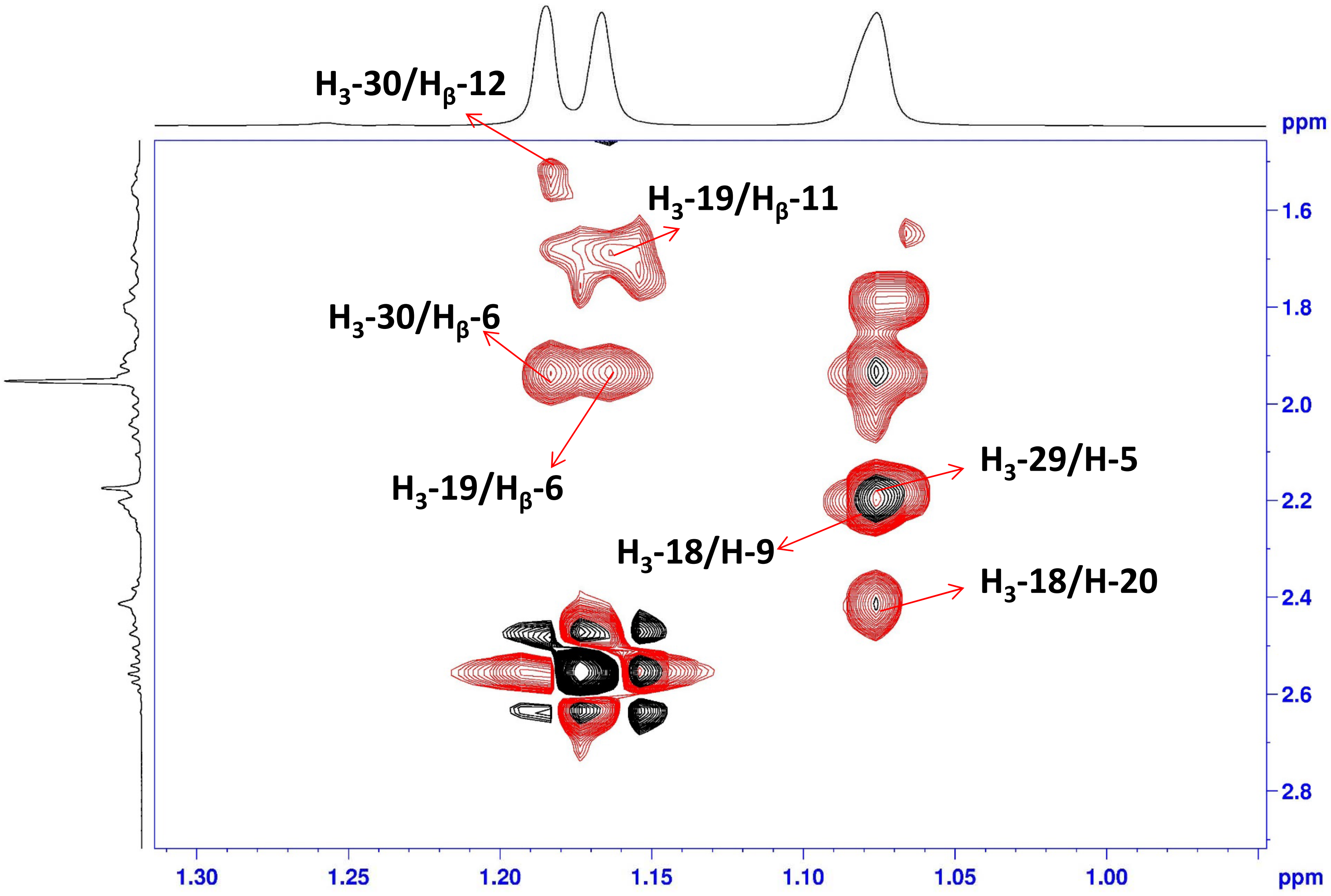

HR-ESIMS for compound **4s**

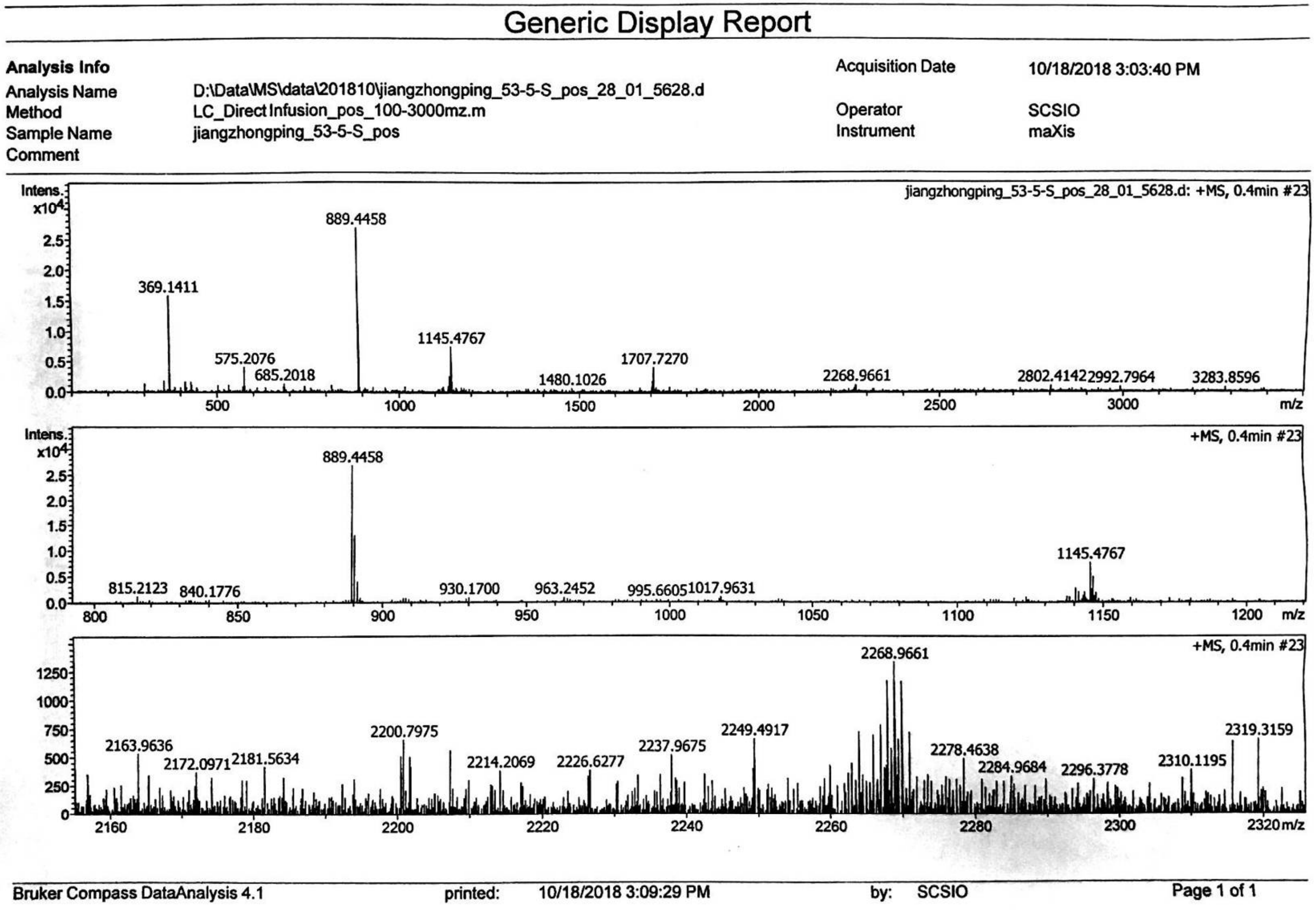

HR-ESIMS for compound **4s**

Mass Spectrum SmartFormula Report

Analysis Info

Analysis Name	D:\Data\MS\data\201810\jiangzhongping_53-5-S_pos_28_01_5628.d	Acquisition Date	10/18/2018 3:03:40 PM
Method	LC_Direct Infusion_pos_100-3000mz.m	Operator	SCSIO
Sample Name	jiangzhongping_53-5-S_pos	Instrument	maXis 255552.00029
Comment			

Acquisition Parameter

Source Type	ESI	Ion Polarity	Positive	Set Nebulizer	0.4 Bar
Focus	Active	Set Capillary	4500 V	Set Dry Heater	180 °C
Scan Begin	100 m/z	Set End Plate Offset	0 V	Set Dry Gas	4.0 l/min
Scan End	3500 m/z	Set Charging Voltage	0 V	Set Divert Valve	Waste
		Set Corona	0 nA	Set APCI Heater	0 °C

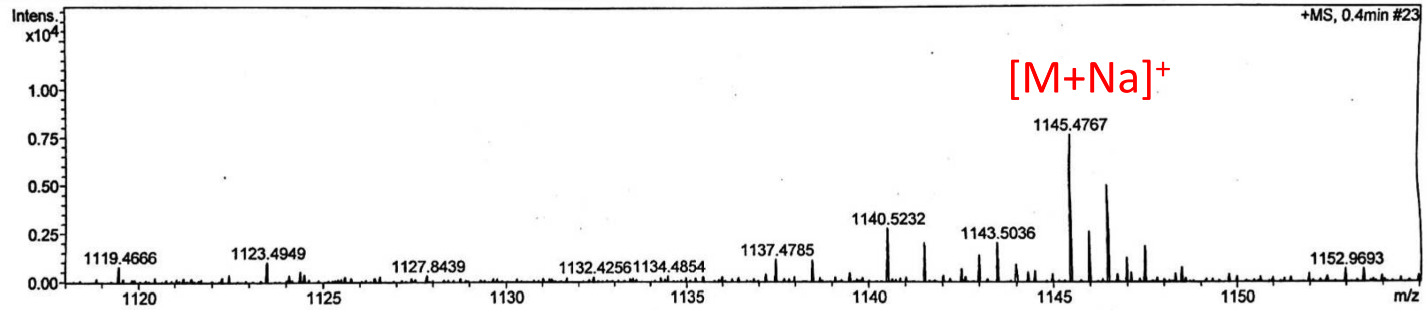

Meas. m/z	#	Ion Formula	Score	m/z	err [ppm]	err [mDa]	mSigma	rdb	e⁻ Conf	N-Rule
1123.4949	1	C60H72F9O10	100.00	1123.4976	2.4	2.7	121.2	20.5	even	ok
1145.4767	1	C60H71F9NaO10	100.00	1145.4796	-2.5	-2.8	18.1	20.5	even	ok

jiangzhongping_53-5-S_pos_28_01_5628.d

Bruker Compass DataAnalysis 4.1 printed: 10/18/2018 3:08:57 PM by: SCSIO Page 1 of 1

^{1}H NMR (400 MHz) spectrum of compound **4s** in $CDCl_3$

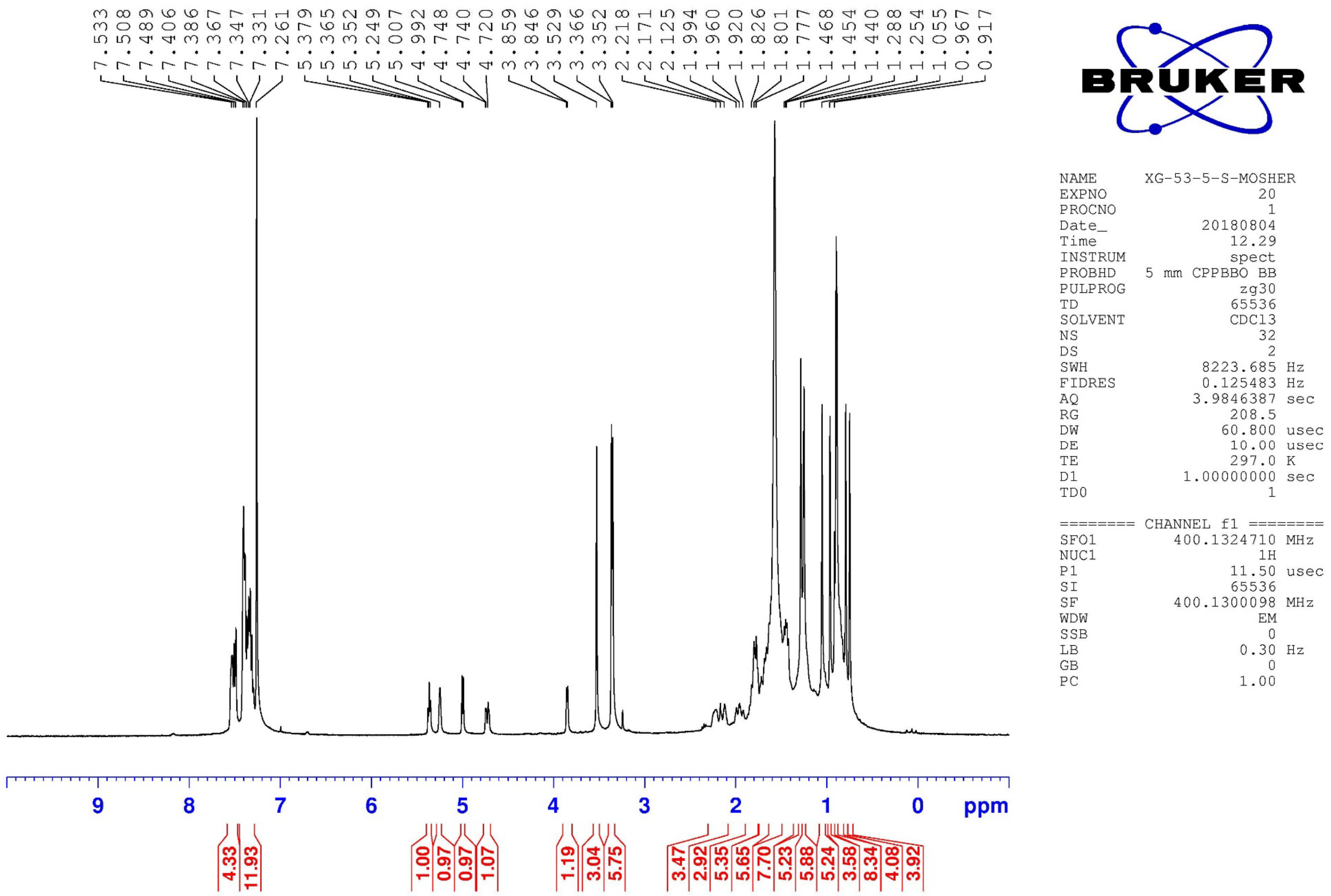

^{1}H NMR (400 MHz) spectrum of compound **4s** in $CDCl_3$

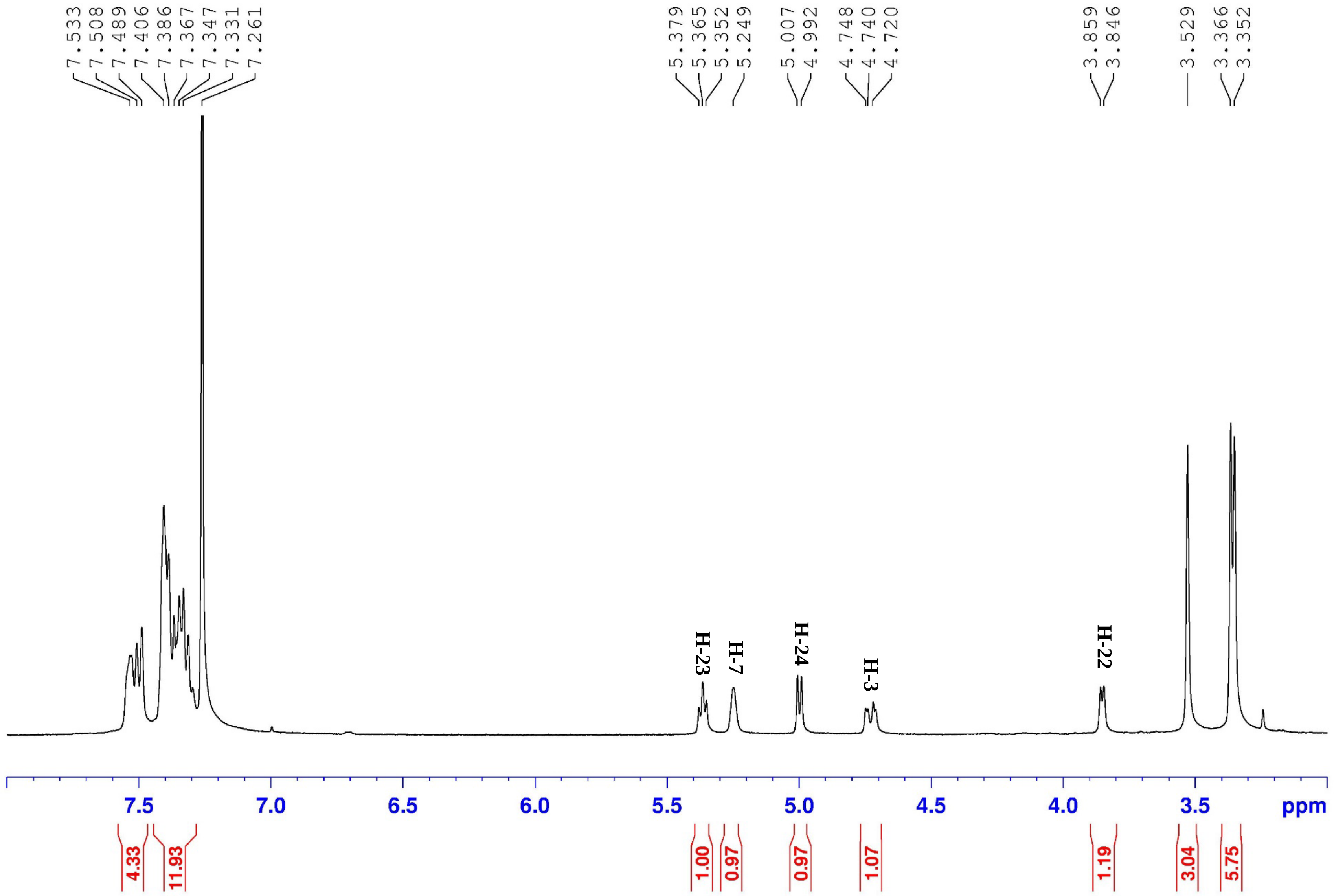

^{1}H NMR (400 MHz) spectrum of compound **4s** in $CDCl_3$

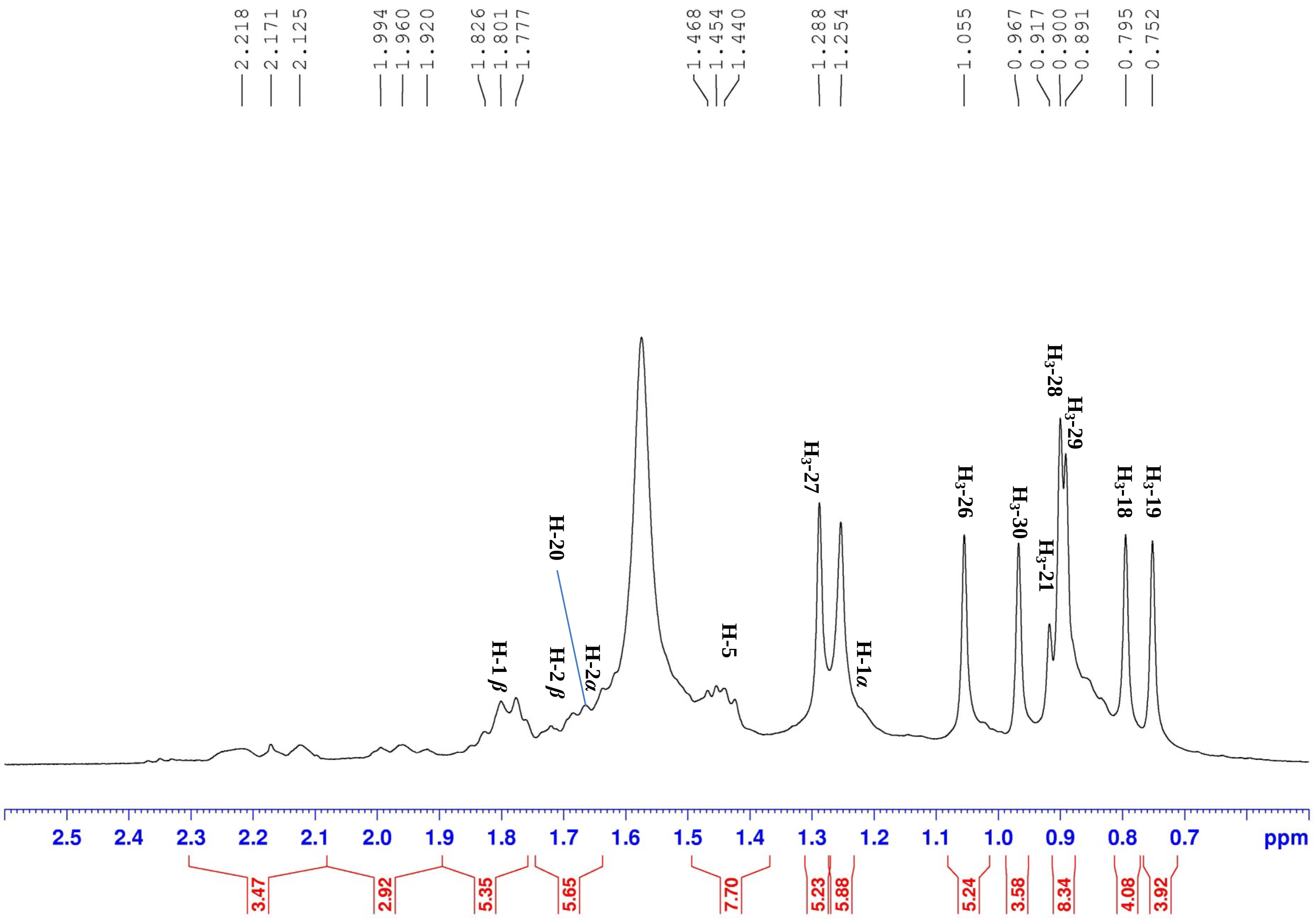

HR-ESIMS for compound **4r**

Generic Display Report

**Analysis Info**		Acquisition Date	10/18/2018 3:00:09 PM
Analysis Name	D:\Data\MS\data\201810\jiangzhongping_53-5-R_pos_27_01_5627.d		
Method	LC_DirectInfusion_pos_100-3000mz.m	Operator	SCSIO
Sample Name	jiangzhongping_53-5-R_pos	Instrument	maXis
Comment			

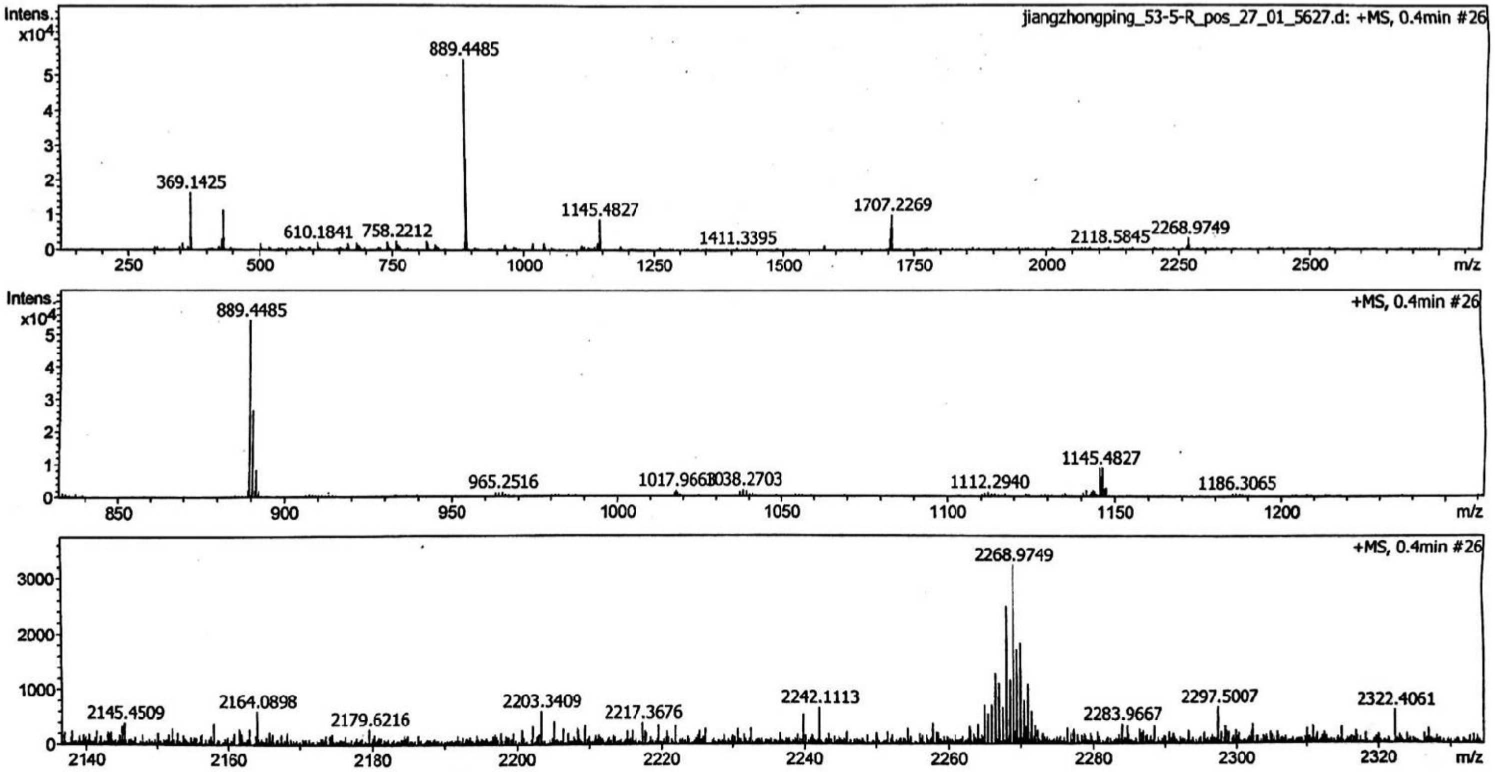

Bruker Compass DataAnalysis 4.1 printed: 10/18/2018 3:07:18 PM by: SCSIO Page 1 of 1

HR-ESIMS for compound **4r**

Mass Spectrum SmartFormula Report

Analysis Info

Analysis Name	D:\Data\MS\data\201810\jiangzhongping_53-5-R_pos_27_01_5627.d	Acquisition Date	10/18/2018 3:00:09 PM
Method	LC_Direct Infusion_pos_100-3000mz.m	Operator	SCSIO
Sample Name	jiangzhongping_53-5-R_pos	Instrument	maXis 255552.00029
Comment			

Acquisition Parameter

Source Type	ESI	Ion Polarity	Positive	Set Nebulizer	0.4 Bar
Focus	Active	Set Capillary	4500 V	Set Dry Heater	180 °C
Scan Begin	100 m/z	Set End Plate Offset	0 V	Set Dry Gas	4.0 l/min
Scan End	3500 m/z	Set Charging Voltage	0 V	Set Divert Valve	Waste
		Set Corona	0 nA	Set APCI Heater	0 °C

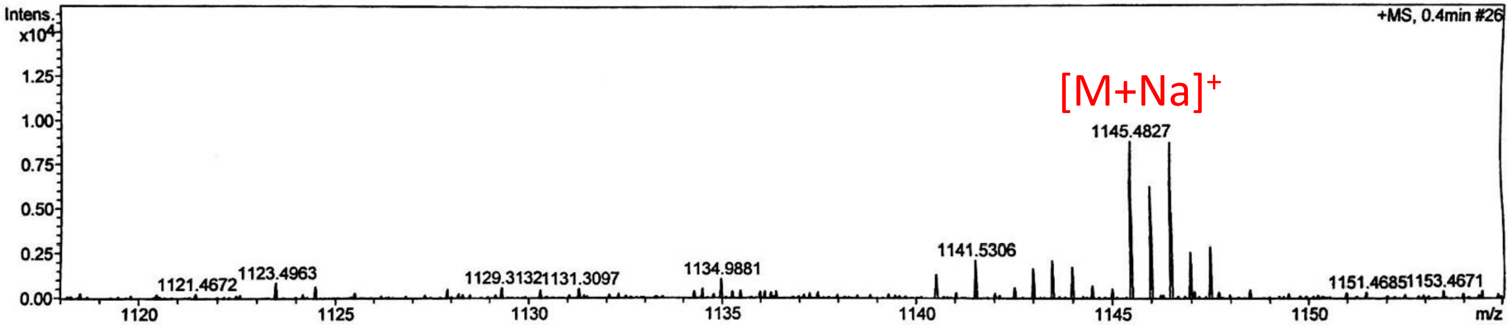

Meas. m/z	#	Ion Formula	Score	m/z	err [ppm]	err [mDa]	mSigma	rdb	e⁻ Conf	N-Rule
1123.4963	1	C60H72F9O10	100.00	1123.4976	-1.2	-1.3	113.8	20.5	even	ok
1145.4827	1	C60H71F9NaO10	100.00	1145.4796	-2.7	-3.1	154.1	20.5	even	ok

jiangzhongping_53-5-R_pos_27_01_5627.d

Bruker Compass DataAnalysis 4.1 printed: 10/18/2018 3:06:11 PM by: SCSIO Page 1 of 1

^{1}H NMR (400 MHz) spectrum of compound **4r** in $CDCl_3$

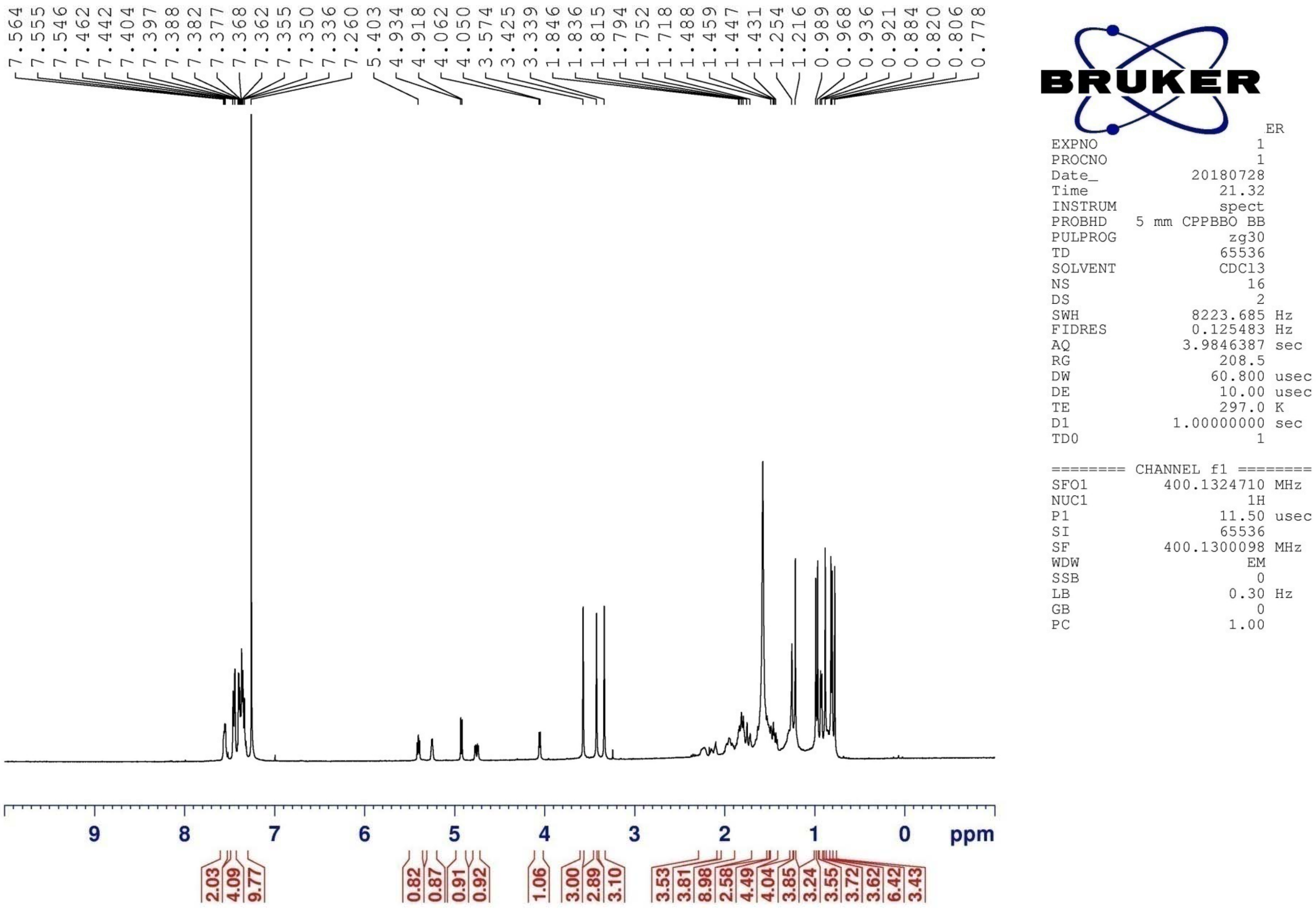

^{1}H NMR (400 MHz) spectrum of compound **4r** in $CDCl_3$

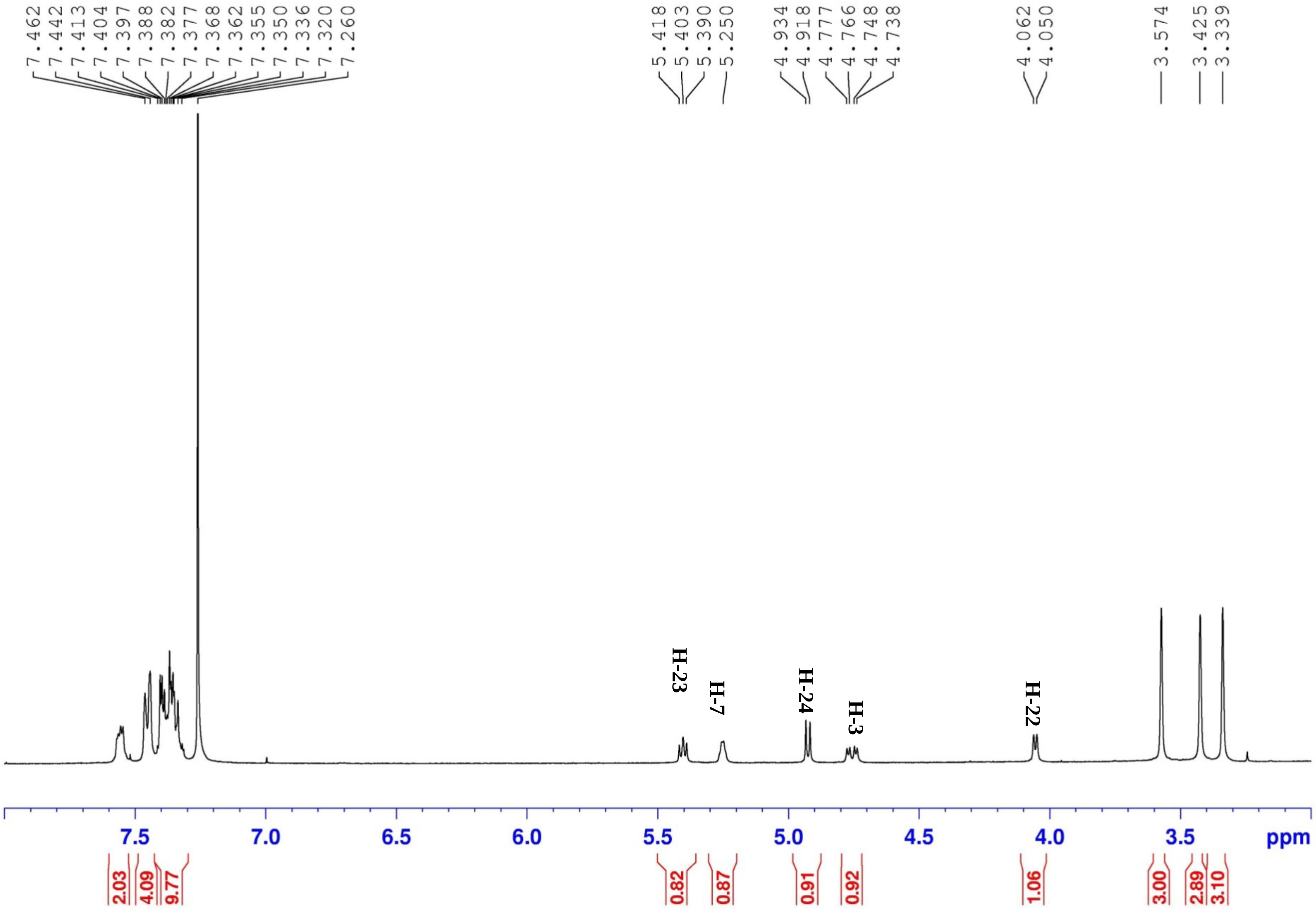

^1H NMR (400 MHz) spectrum of compound **4r** in $CDCl_3$

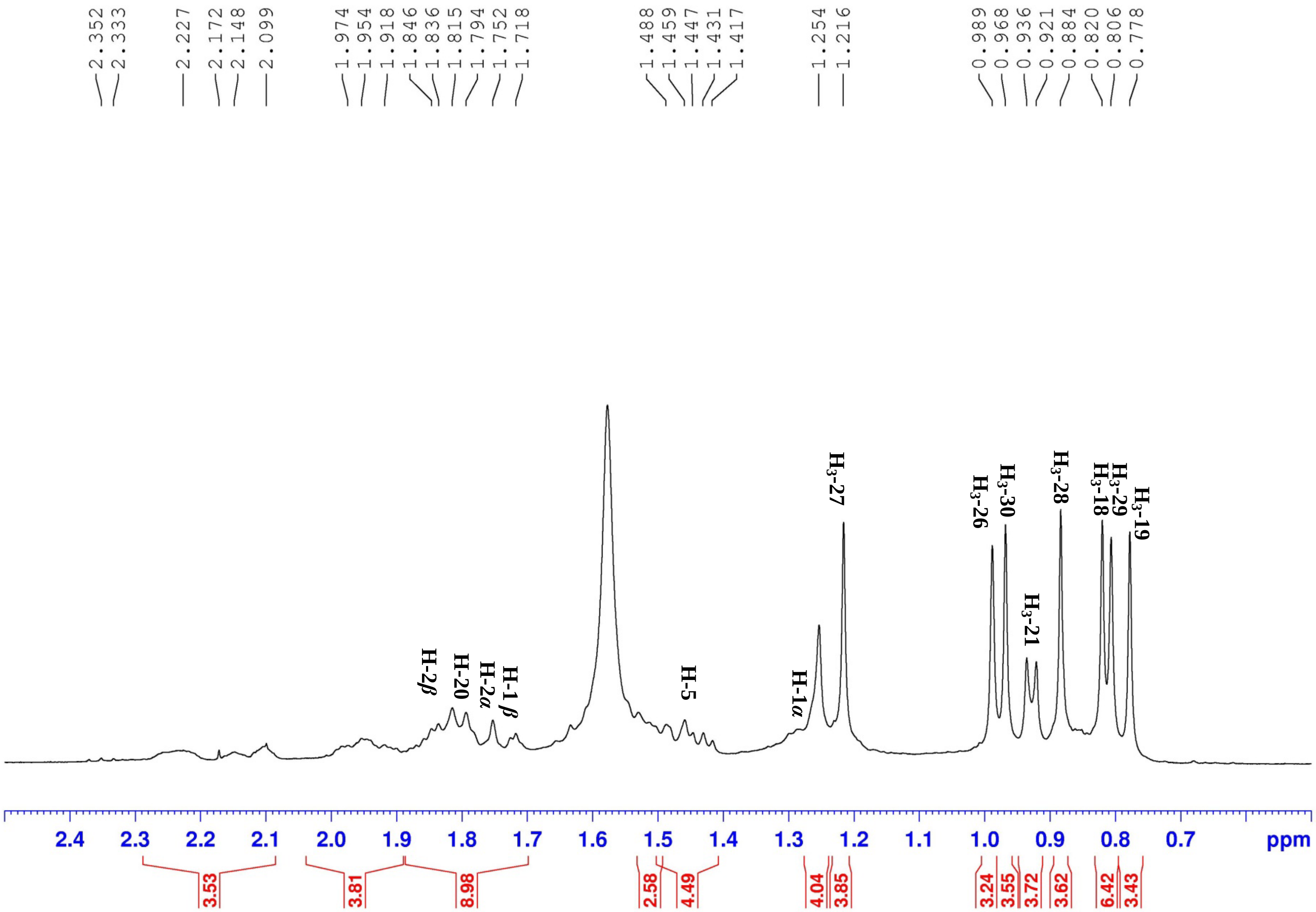

HR-ESIMS for compound **6s**

Elemental Composition Report **Page 1**

Single Mass Analysis
Tolerance = 10.0 PPM / DBE: min = -1.5, max = 50.0
Element prediction: Off
Number of isotope peaks used for i-FIT = 3

Monoisotopic Mass, Even Electron Ions
66 formula(e) evaluated with 1 results within limits (up to 50 best isotopic matches for each mass)
Elements Used:
C: 0-50 H: 0-100 O: 0-8 F: 0-6

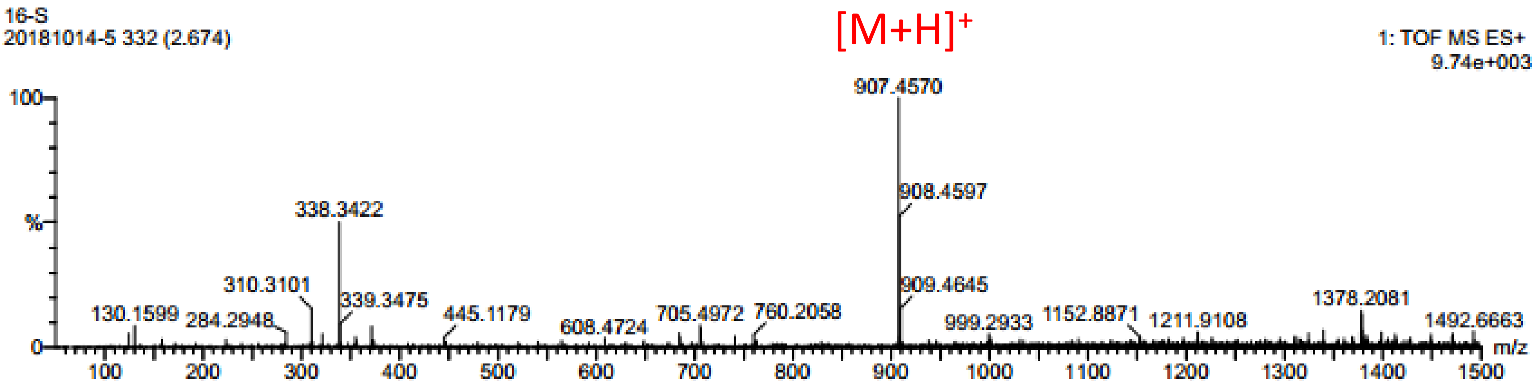

Minimum:				-1.5				
Maximum:		2.0	10.0	50.0				
Mass	Calc. Mass	mDa	PPM	DBE	i-FIT	Norm	Conf(%)	Formula
907.4570	907.4584	-1.4	-1.5	15.5	142.9	n/a	n/a	C50 H65 O8 F6

^{1}H NMR (400 MHz) spectrum of compound **6s** in $CDCl_3$

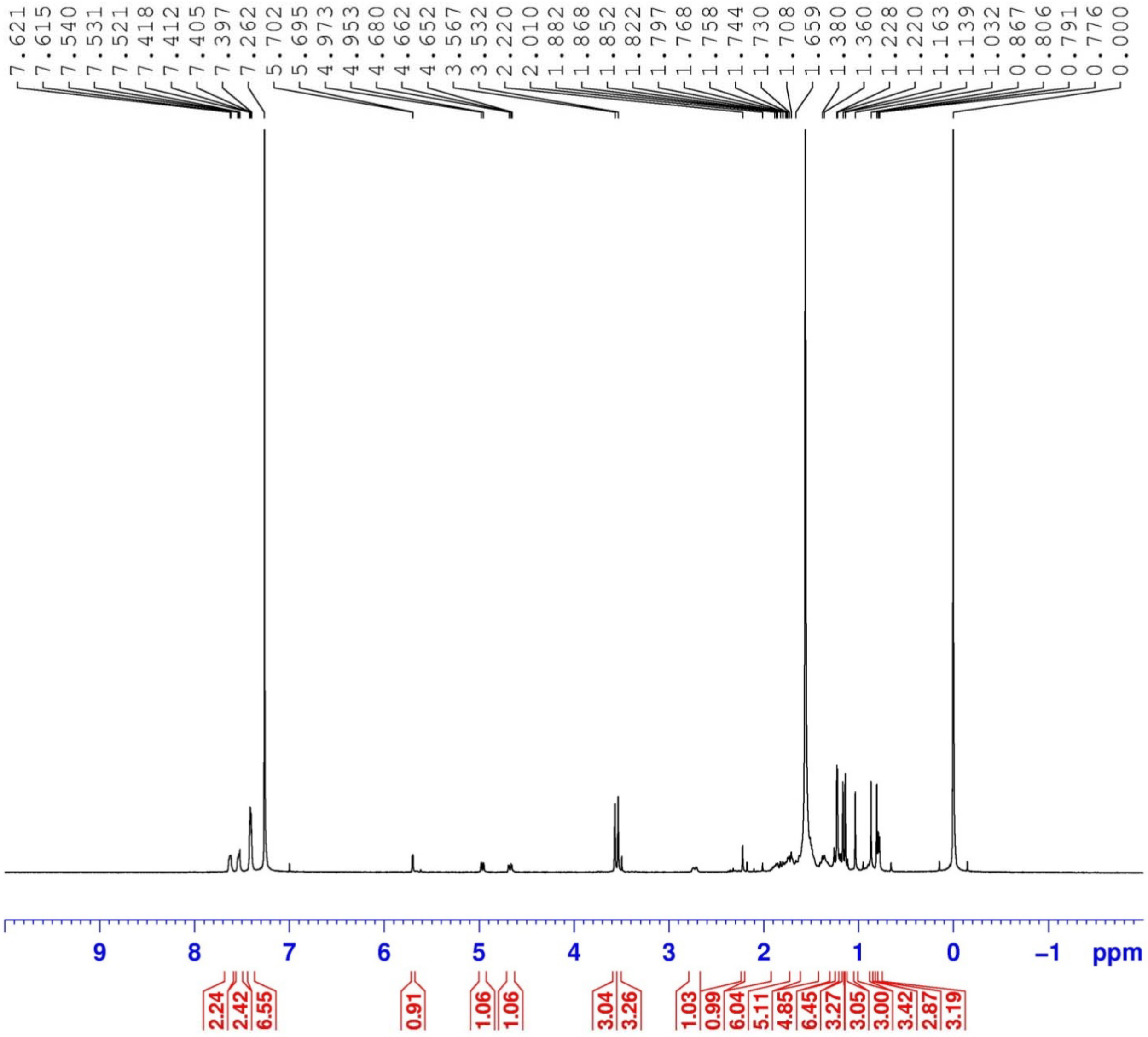

NAME	EA-16-S-1	
EXPNO	1	
PROCNO	1	
Date_	20170119	
Time	23.04	
INSTRUM	spect	
PROBHD	5 mm CPPBBO BB	
PULPROG	zg30	
TD	65536	
SOLVENT	CDCl3	
NS	32	
DS	2	
SWH	8223.685	Hz
FIDRES	0.125483	Hz
AQ	3.9846387	sec
RG	208.5	
DW	60.800	usec
DE	10.00	usec
TE	297.0	K
D1	1.00000000	sec
TD0	1	
======== CHANNEL f1 ========		
SFO1	400.1324710	MHz
NUC1	1H	
P1	11.50	usec
SI	65536	
SF	400.1300091	MHz
WDW	EM	
SSB	0	
LB	0.30	Hz
GB	0	
PC	1.00	

^{1}H NMR (400 MHz) spectrum of compound **6s** in $CDCl_3$

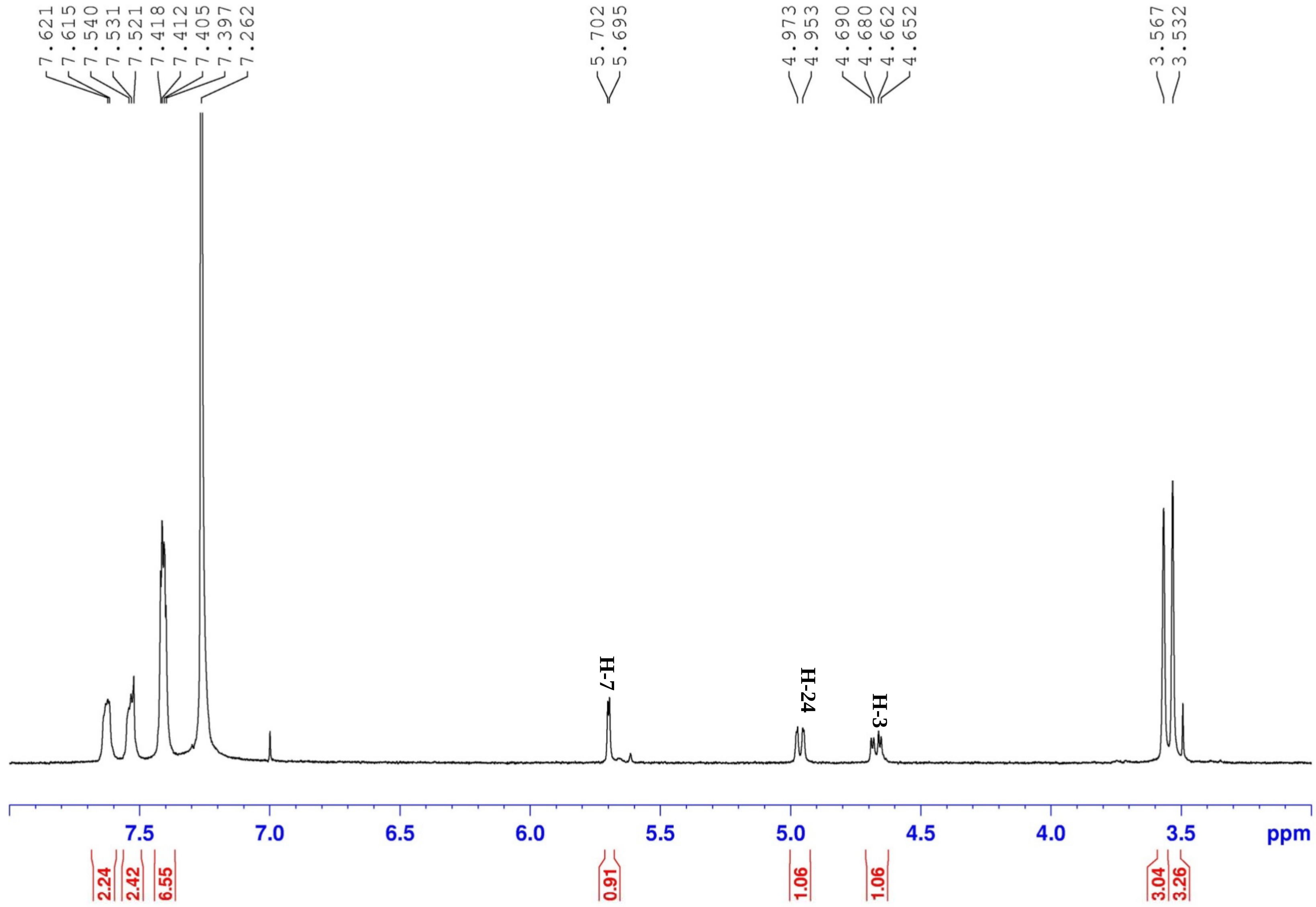

^{1}H NMR (400 MHz) spectrum of compound **6s** in $CDCl_3$

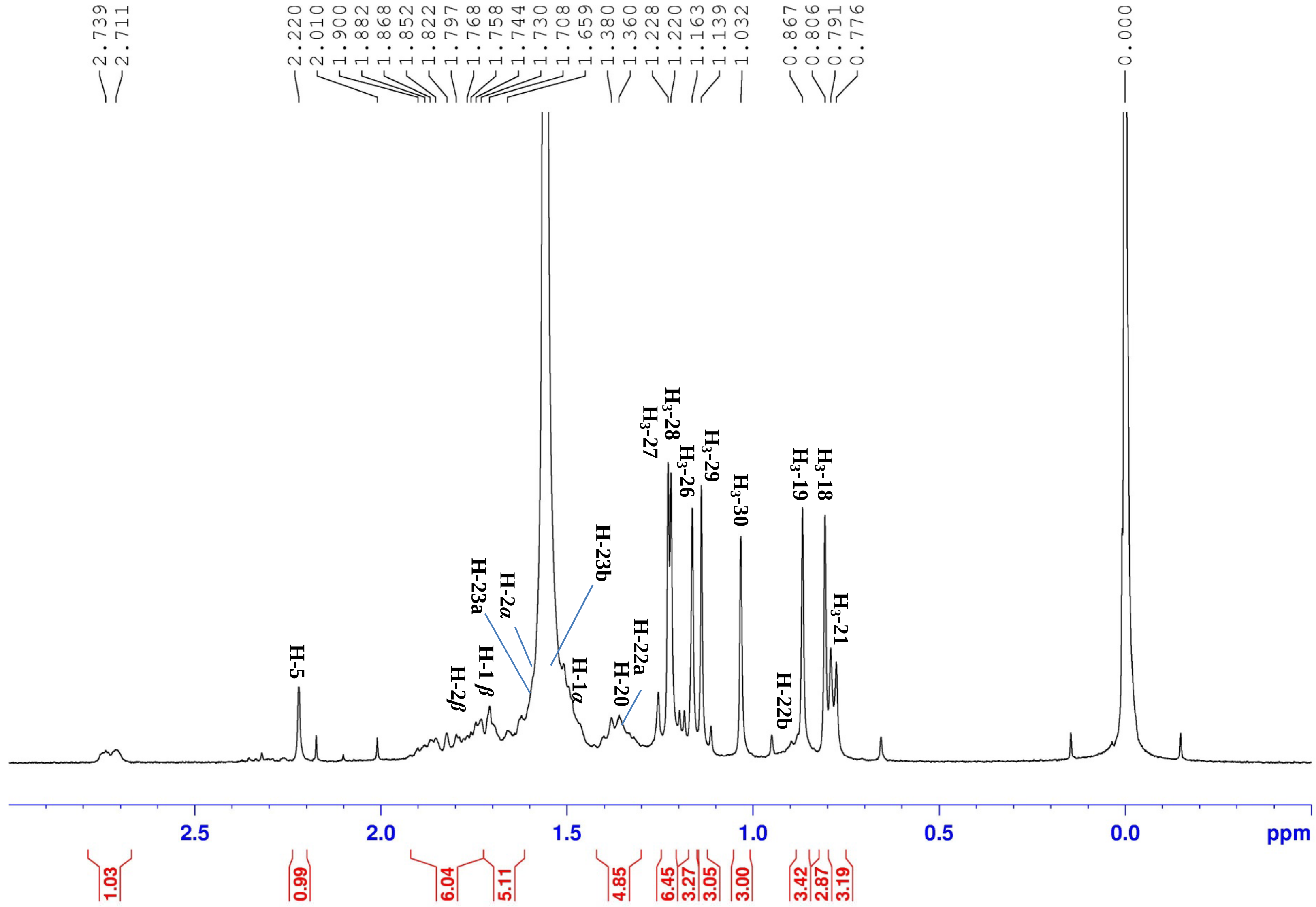

HR-ESIMS for compound **6r**

Elemental Composition Report **Page 1**

Single Mass Analysis
Tolerance = 10.0 PPM / DBE: min = -1.5, max = 50.0
Element prediction: Off
Number of isotope peaks used for i-FIT = 3

Monoisotopic Mass, Even Electron Ions
66 formula(e) evaluated with 1 results within limits (up to 50 best isotopic matches for each mass)
Elements Used:
C: 0-50 H: 0-100 O: 0-8 F: 0-6

16-R
20181014-4 331 (2.666) 1: TOF MS ES+ 1.13e+004

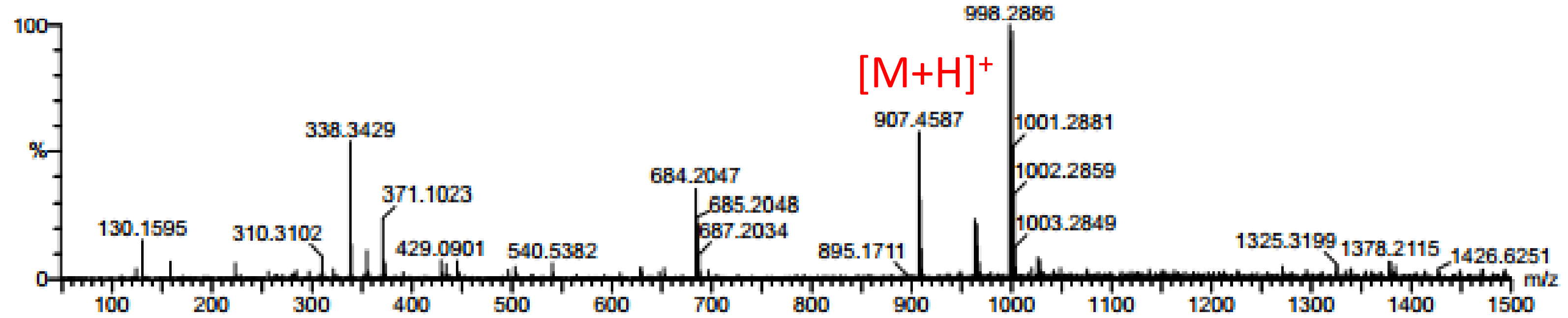

Minimum:				-1.5				
Maximum:		2.0	10.0	50.0				
Mass	Calc. Mass	mDa	PPM	DBE	i-FIT	Norm	Conf(%)	Formula
907.4587	907.4584	0.3	0.3	15.5	157.4	n/a	n/a	C50 H65 O8 F6

^{1}H NMR (400 MHz) spectrum of compound **6r** in $CDCl_3$

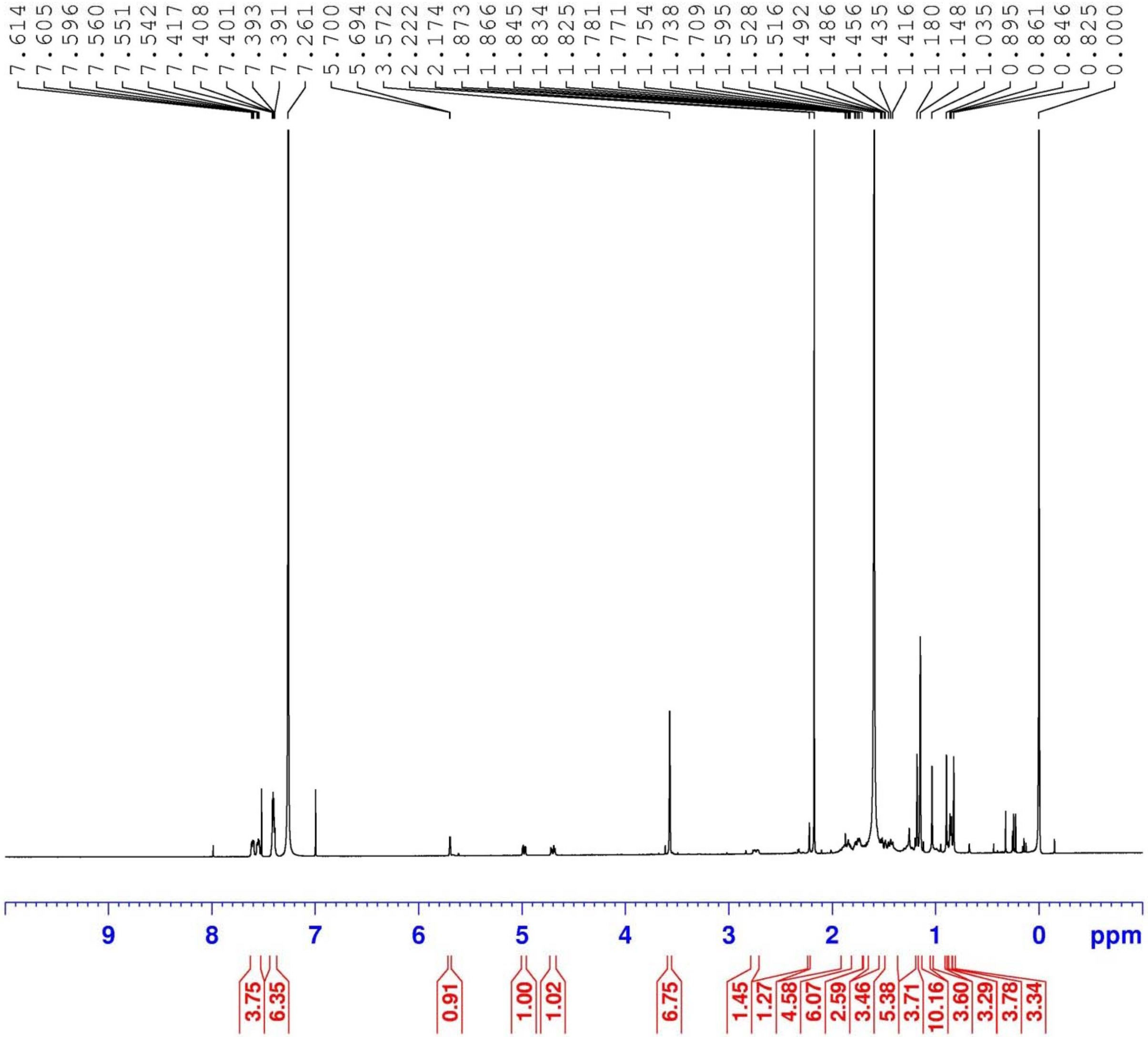


```
NAME       EA-16-R-MOSHER-2
EXPNO                     1
PROCNO                    1
Date_              20170310
Time                   5.07
INSTRUM               spect
PROBHD      5 mm CPPBBO BB
PULPROG                zg30
TD                    65536
SOLVENT               CDCl3
NS                      128
DS                        2
SWH                8223.685 Hz
FIDRES             0.125483 Hz
AQ                3.9846387 sec
RG                    208.5
DW                   60.800 usec
DE                    10.00 usec
TE                    297.0 K
D1               1.00000000 sec
TD0                       1

======== CHANNEL f1 ========
SFO1            400.1324710 MHz
NUC1                     1H
P1                    11.50 usec
SI                    65536
SF              400.1300091 MHz
WDW                      EM
SSB                       0
LB                     0.30 Hz
GB                        0
PC                     1.00
```

^{1}H NMR (400 MHz) spectrum of compound **6r** in $CDCl_3$

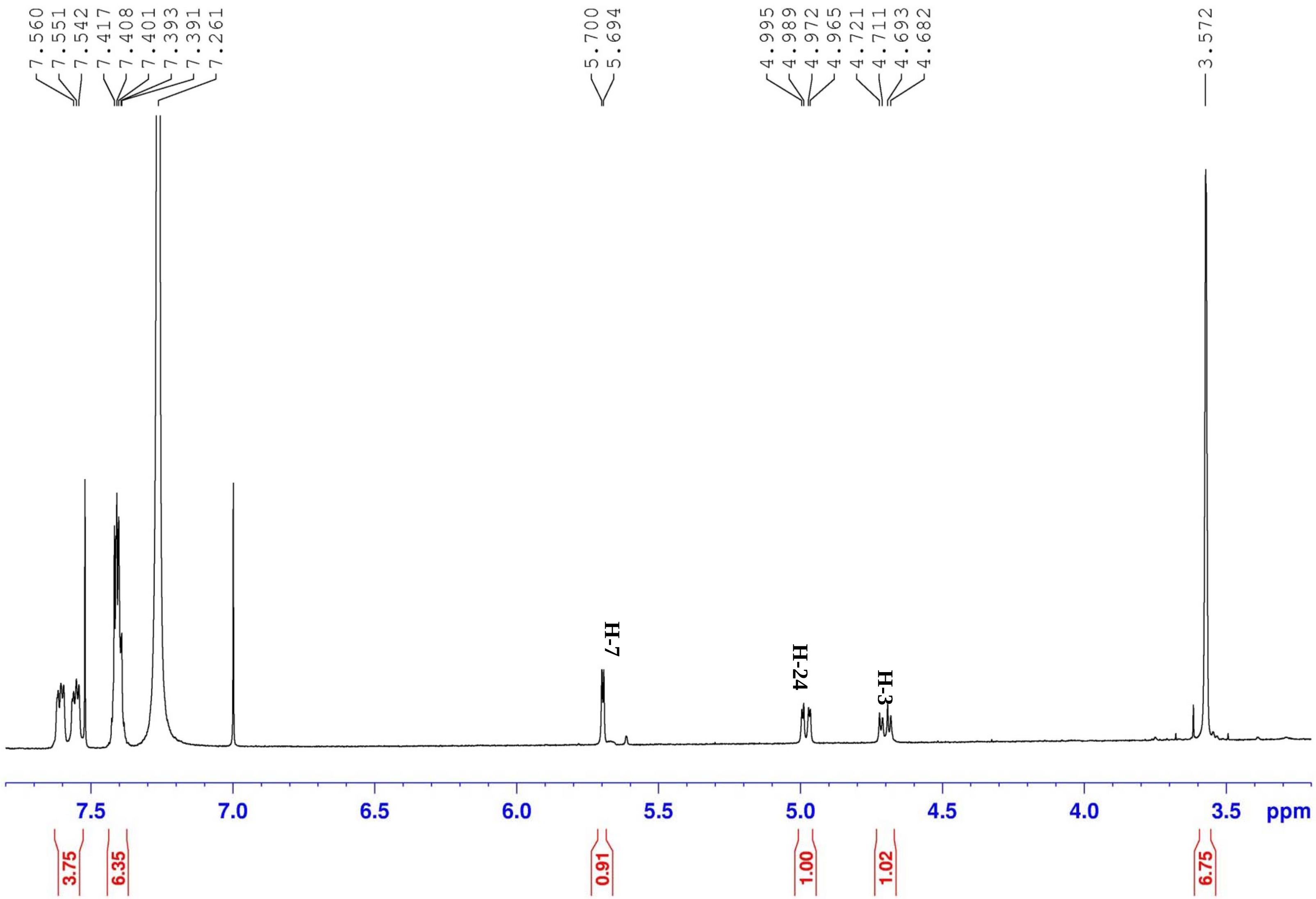

^{1}H NMR (400 MHz) spectrum of compound **6r** in $CDCl_3$

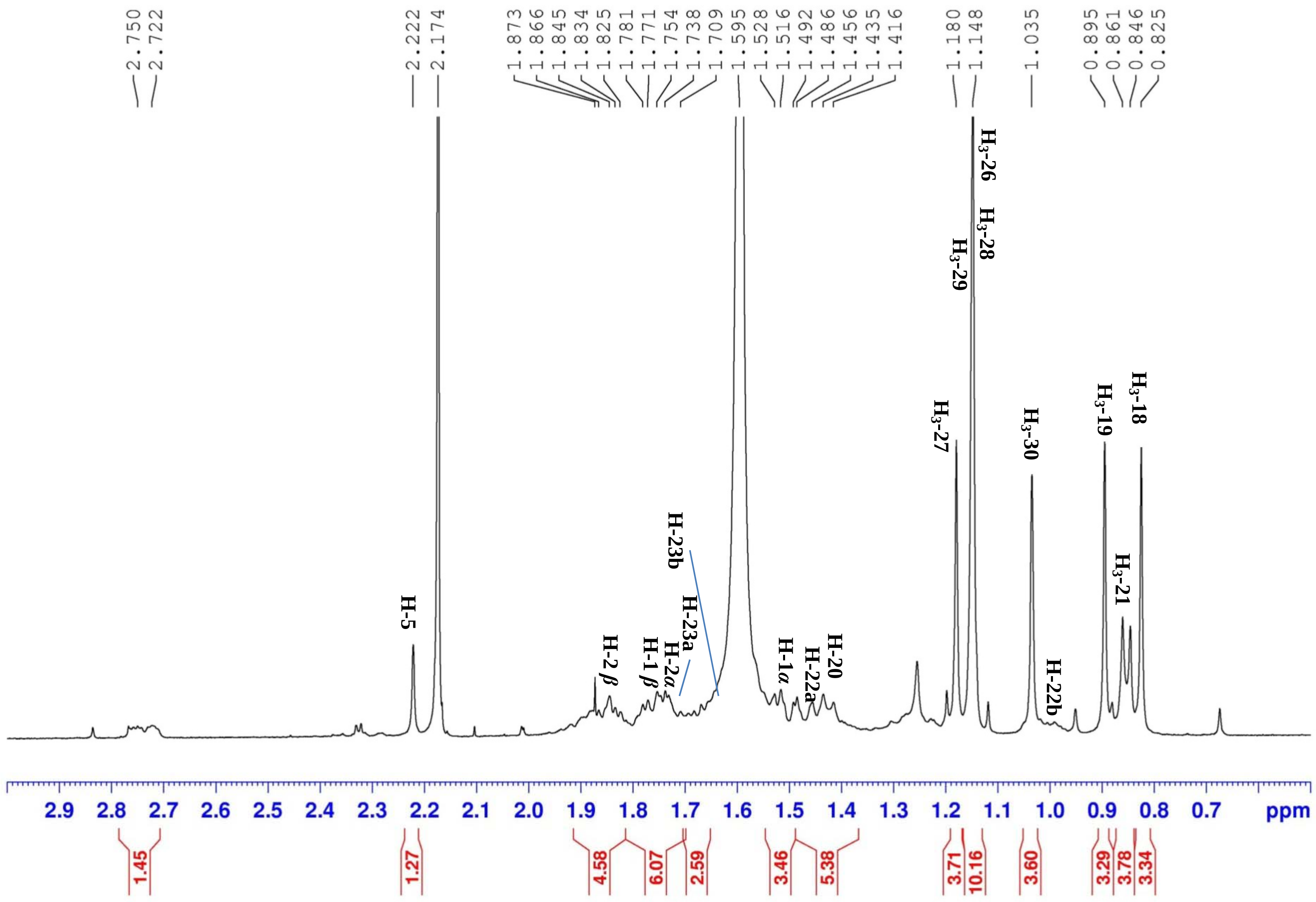

HR-ESIMS for compound **7s**

Elemental Composition Report **Page 1**

Single Mass Analysis
Tolerance = 10.0 PPM / DBE: min = -1.5, max = 50.0
Element prediction: Off
Number of isotope peaks used for i-FIT = 3

Monoisotopic Mass, Even Electron Ions
66 formula(e) evaluated with 1 results within limits (up to 50 best isotopic matches for each mass)
Elements Used:
C: 0-50 H: 0-100 O: 0-8 F: 0-6

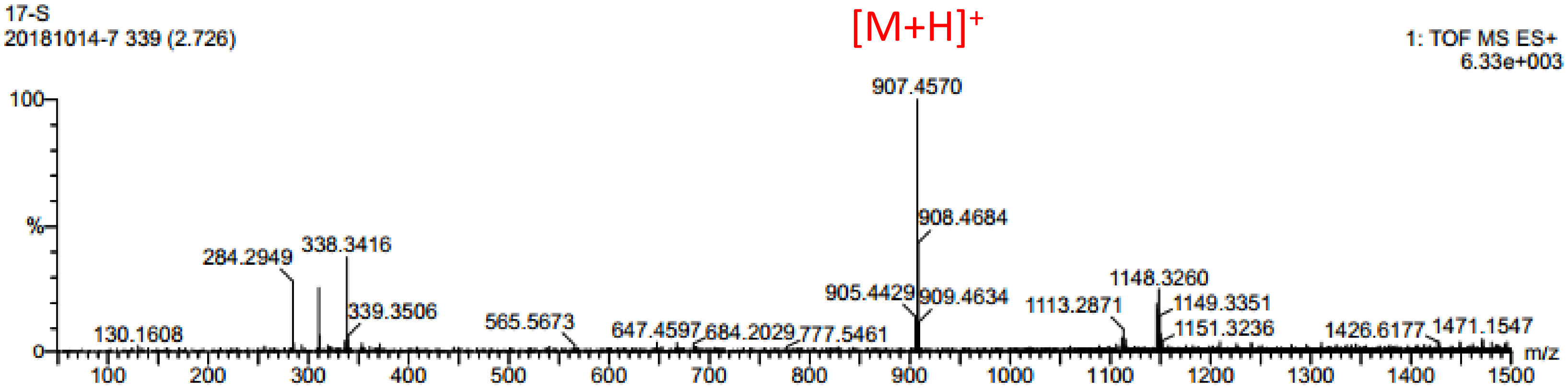

Minimum:				-1.5				
Maximum:		2.0	10.0	50.0				
Mass	Calc. Mass	mDa	PPM	DBE	i-FIT	Norm	Conf(%)	Formula
907.4570	907.4584	-1.4	-1.5	15.5	120.6	n/a	n/a	C50 H65 O8 F6

^{1}H NMR (400 MHz) spectrum of compound **7s** in $CDCl_3$

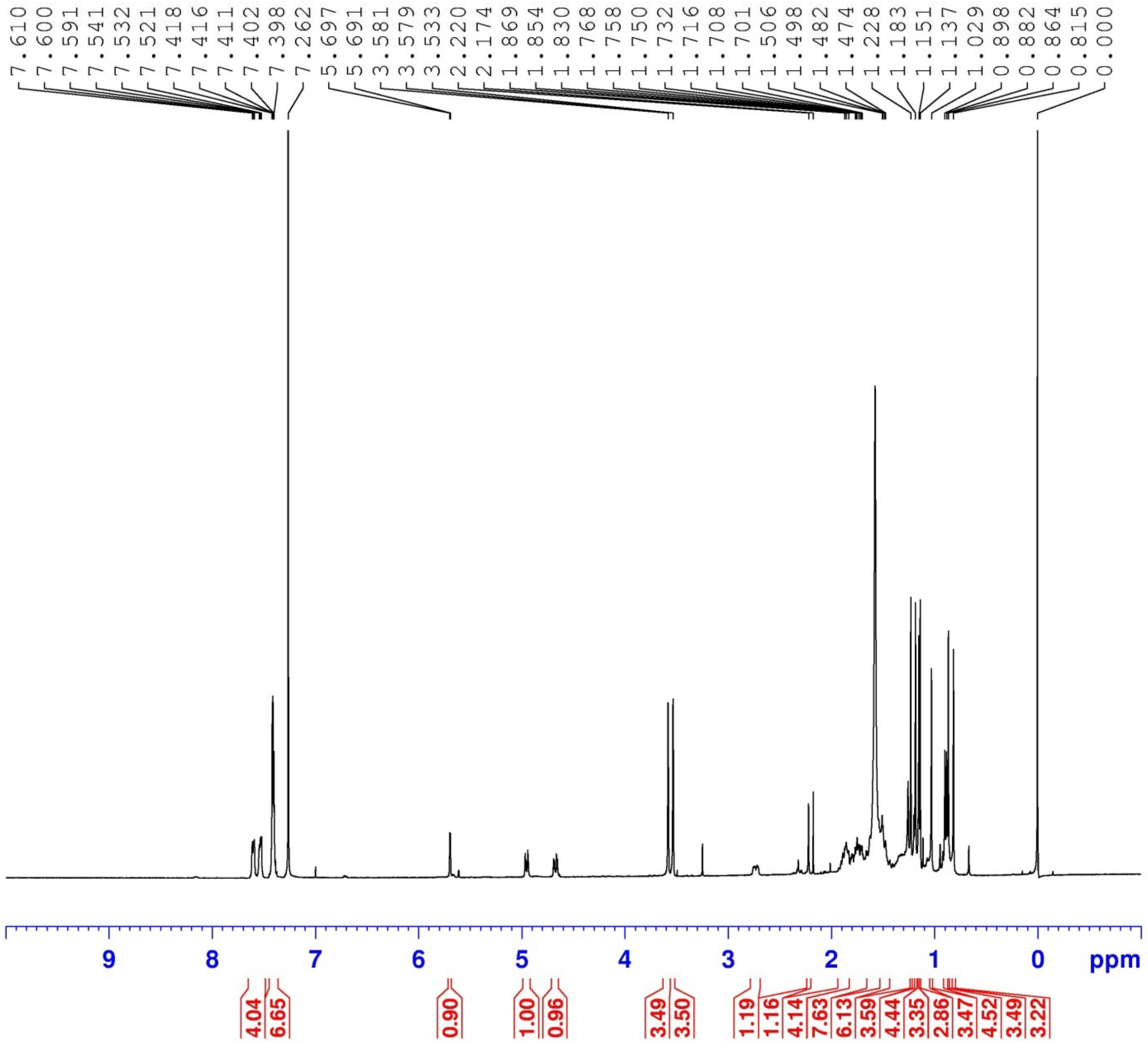

NAME	EA-17-S-MOSHER
EXPNO	1
PROCNO	1
Date_	20180714
Time	21.56
INSTRUM	spect
PROBHD	5 mm CPPBBO BB
PULPROG	zg30
TD	65536
SOLVENT	CDCl3
NS	16
DS	2
SWH	8223.685 Hz
FIDRES	0.125483 Hz
AQ	3.9846387 sec
RG	208.5
DW	60.800 usec
DE	10.00 usec
TE	297.0 K
D1	1.00000000 sec
TD0	1
======== CHANNEL f1 ========	
SFO1	400.1324710 MHz
NUC1	1H
P1	11.50 usec
SI	65536
SF	400.1300089 MHz
WDW	EM
SSB	0
LB	0.30 Hz
GB	0
PC	1.00

^{1}H NMR (400 MHz) spectrum of compound **7s** in $CDCl_3$

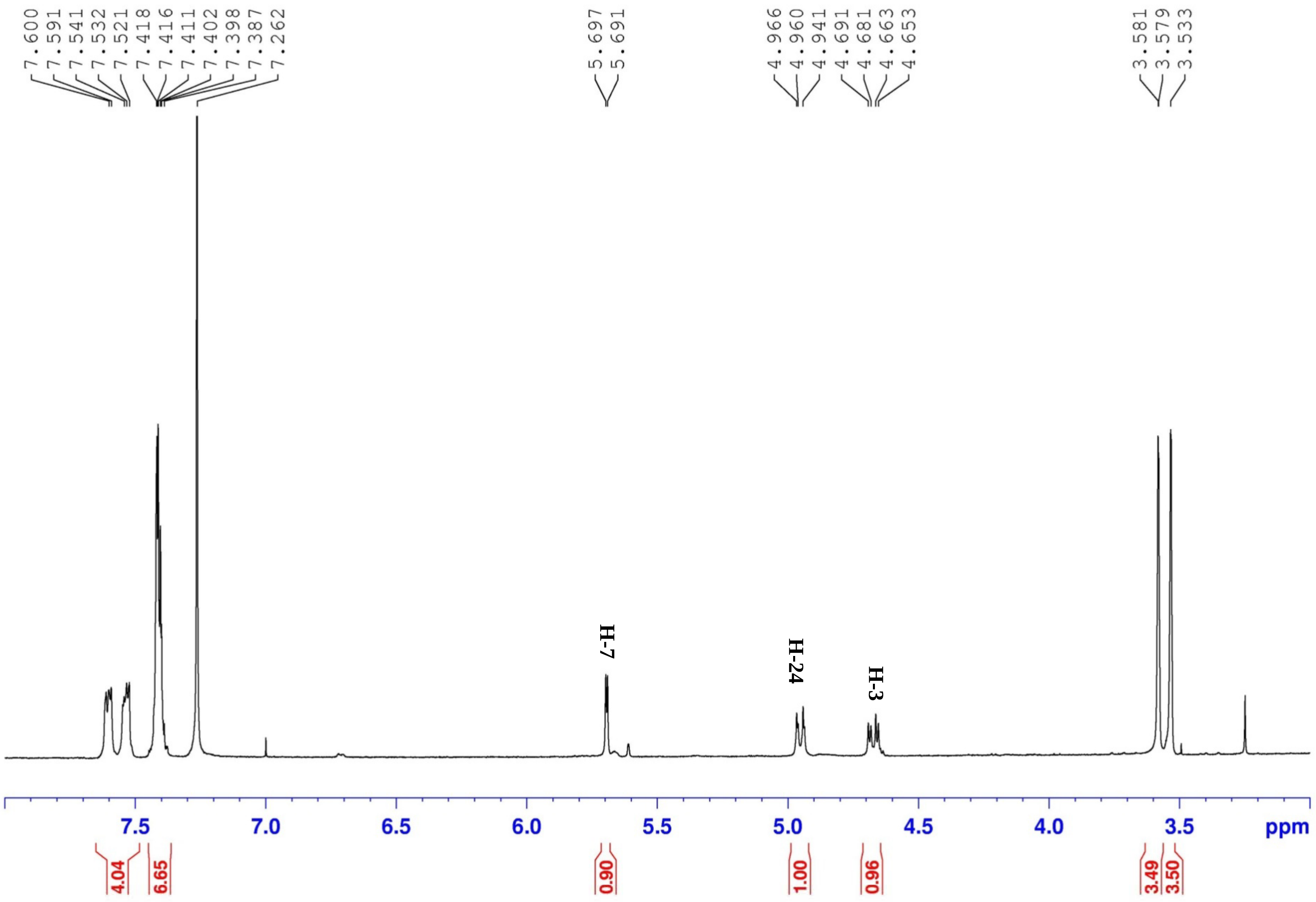

^{1}H NMR (400 MHz) spectrum of compound **7s** in $CDCl_3$

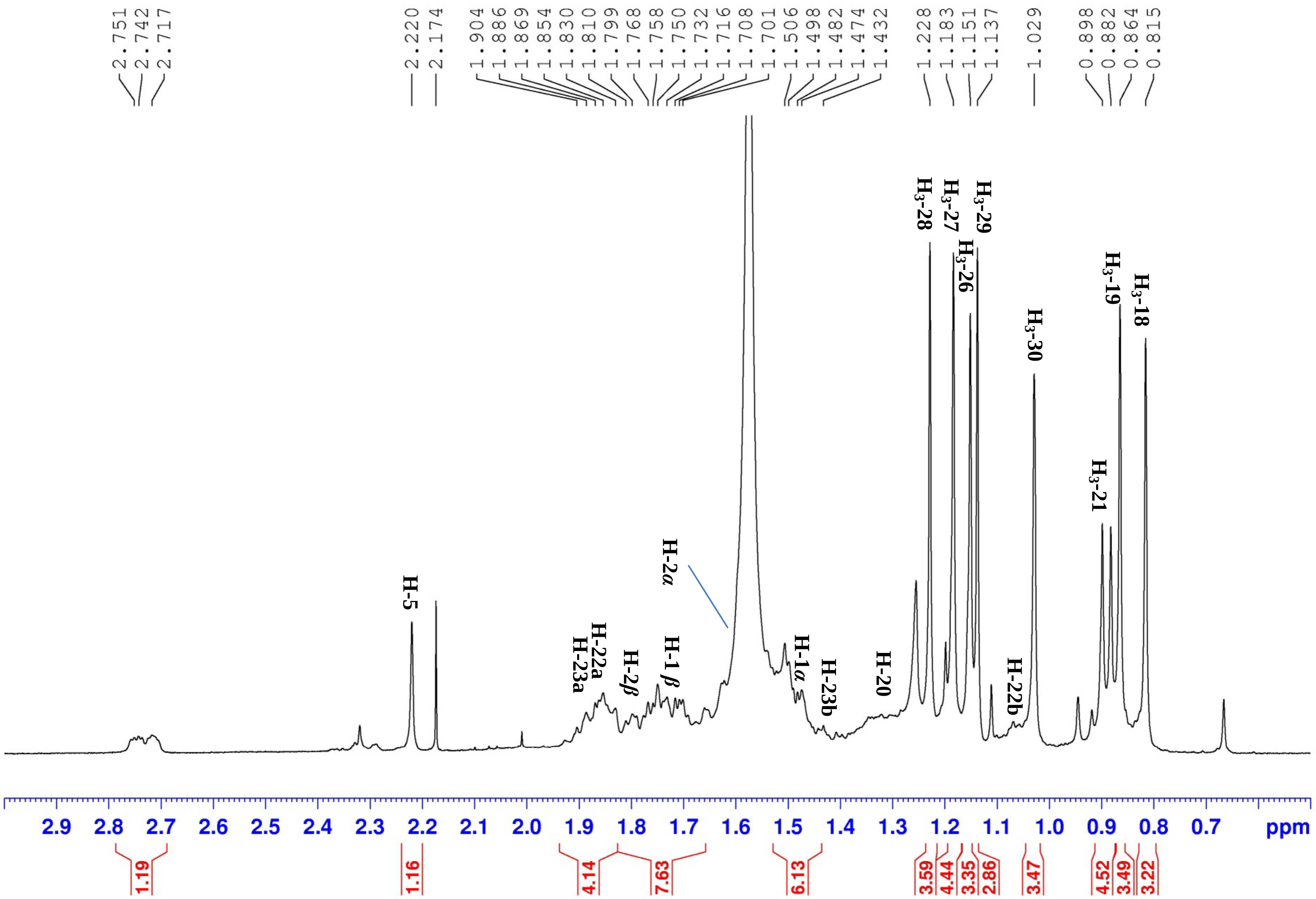

HR-ESIMS for compound **7r**

Elemental Composition Report **Page 1**

Single Mass Analysis
Tolerance = 10.0 PPM / DBE: min = -1.5, max = 50.0
Element prediction: Off
Number of isotope peaks used for i-FIT = 3

Monoisotopic Mass, Even Electron Ions
66 formula(e) evaluated with 1 results within limits (up to 50 best isotopic matches for each mass)
Elements Used:
C: 0-50 H: 0-100 O: 0-8 F: 0-6

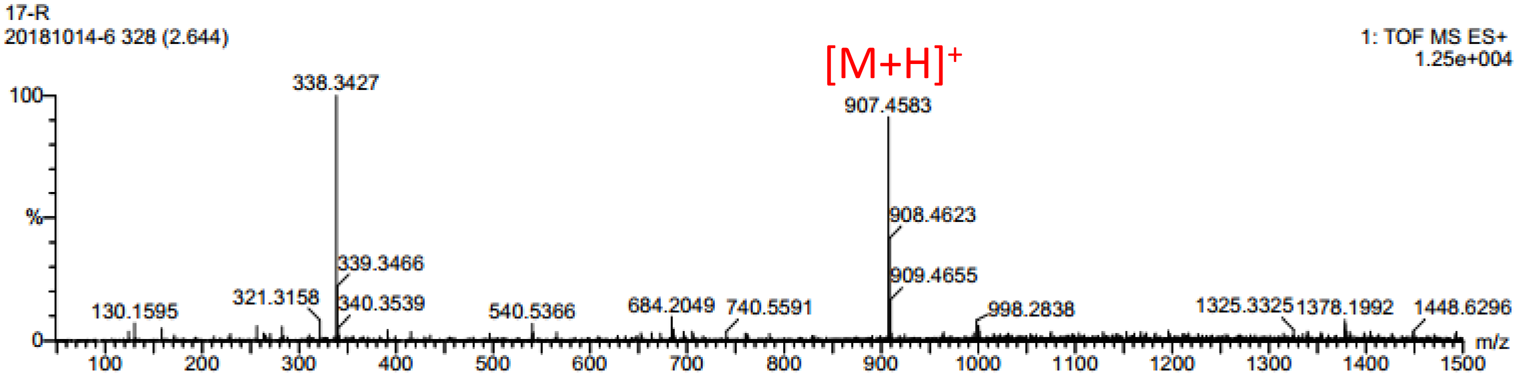

Minimum:				-1.5				
Maximum:		2.0	10.0	50.0				

Mass	Calc. Mass	mDa	PPM	DBE	i-FIT	Norm	Conf(%)	Formula
907.4583	907.4584	-0.1	-0.1	15.5	191.0	n/a	n/a	C50 H65 O8 F6

^{1}H NMR (400 MHz) spectrum of compound **7r** in $CDCl_3$

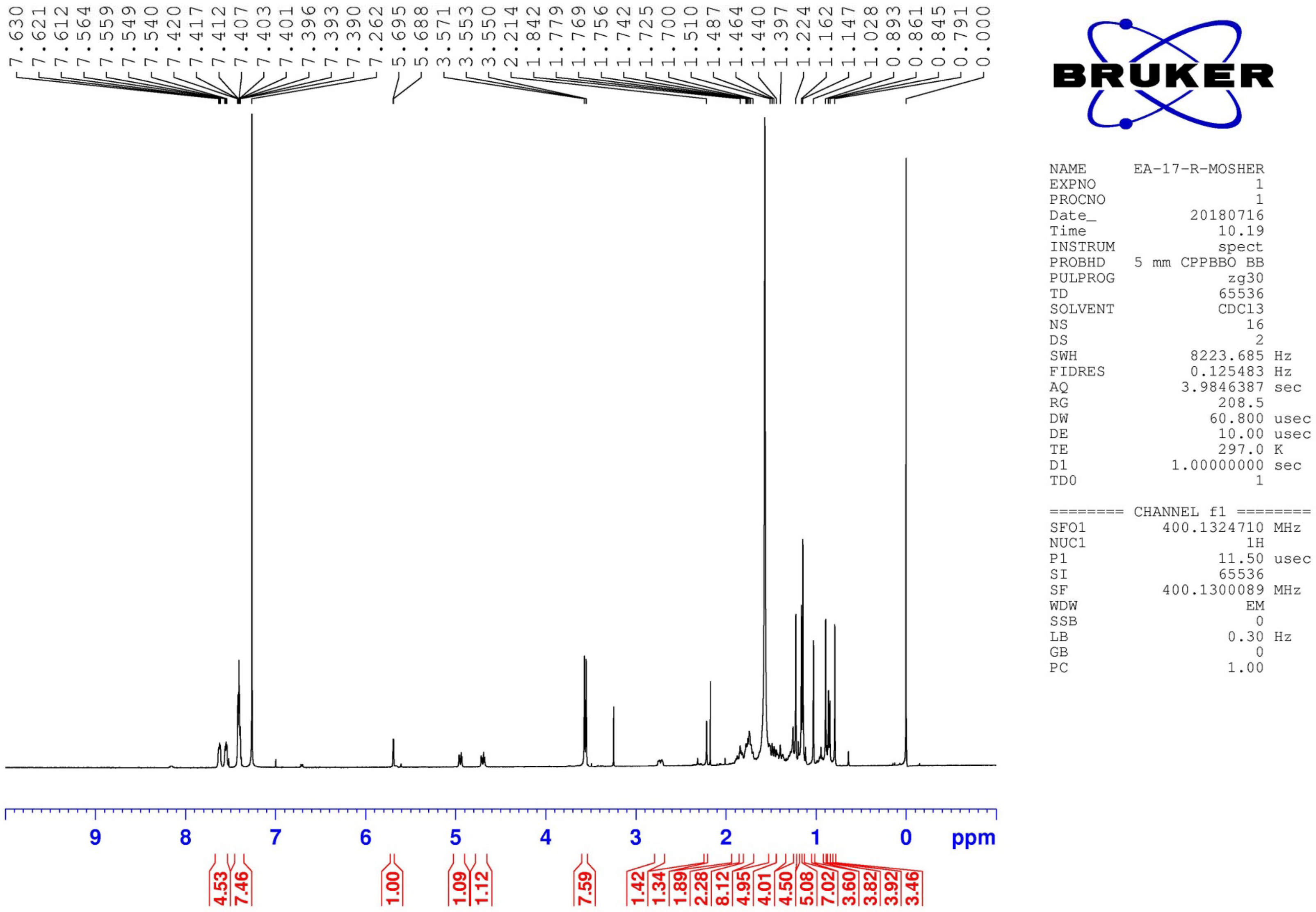

^{1}H NMR (400 MHz) spectrum of compound **7r** in $CDCl_3$

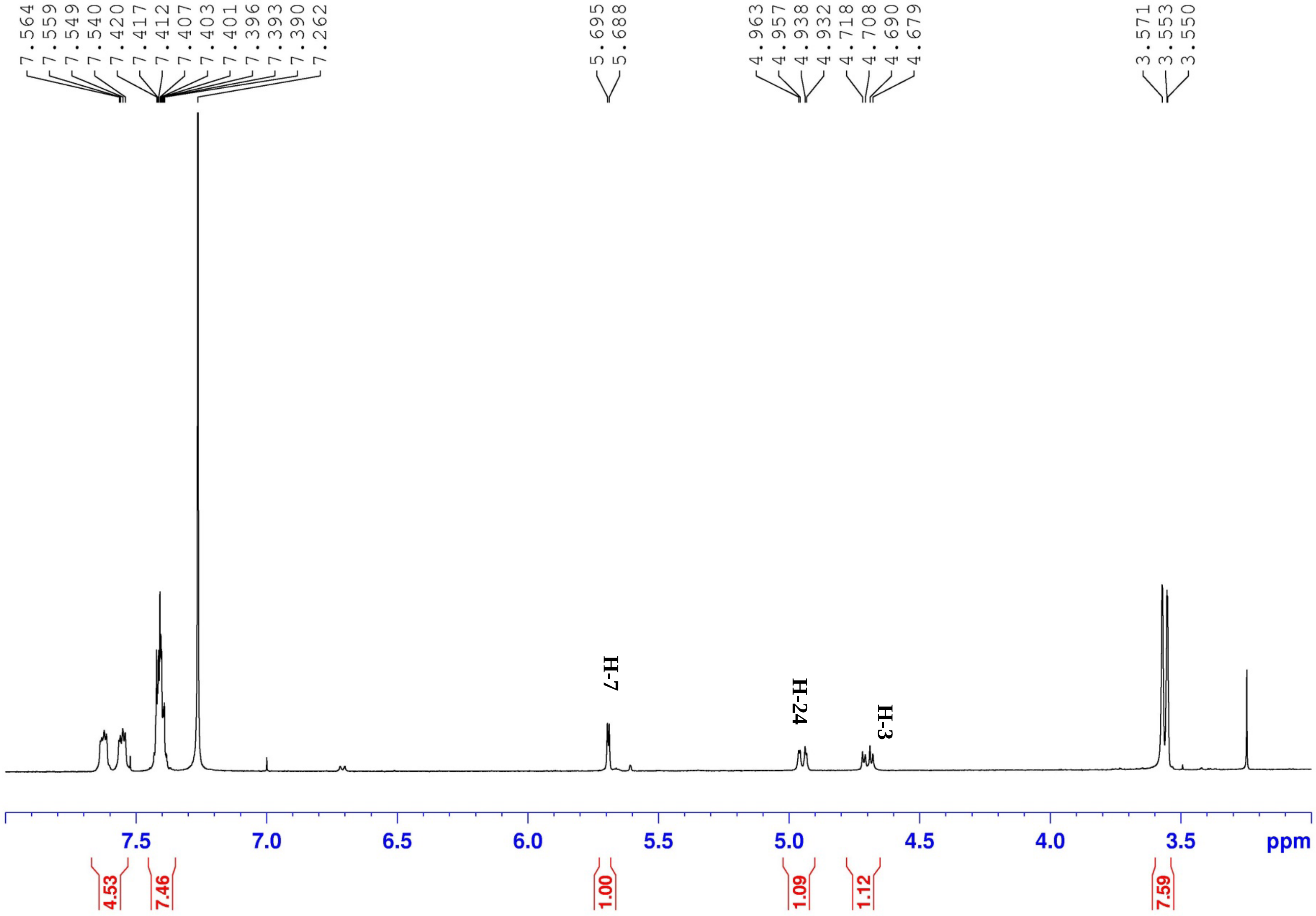

^{1}H NMR (400 MHz) spectrum of compound **7r** in $CDCl_3$

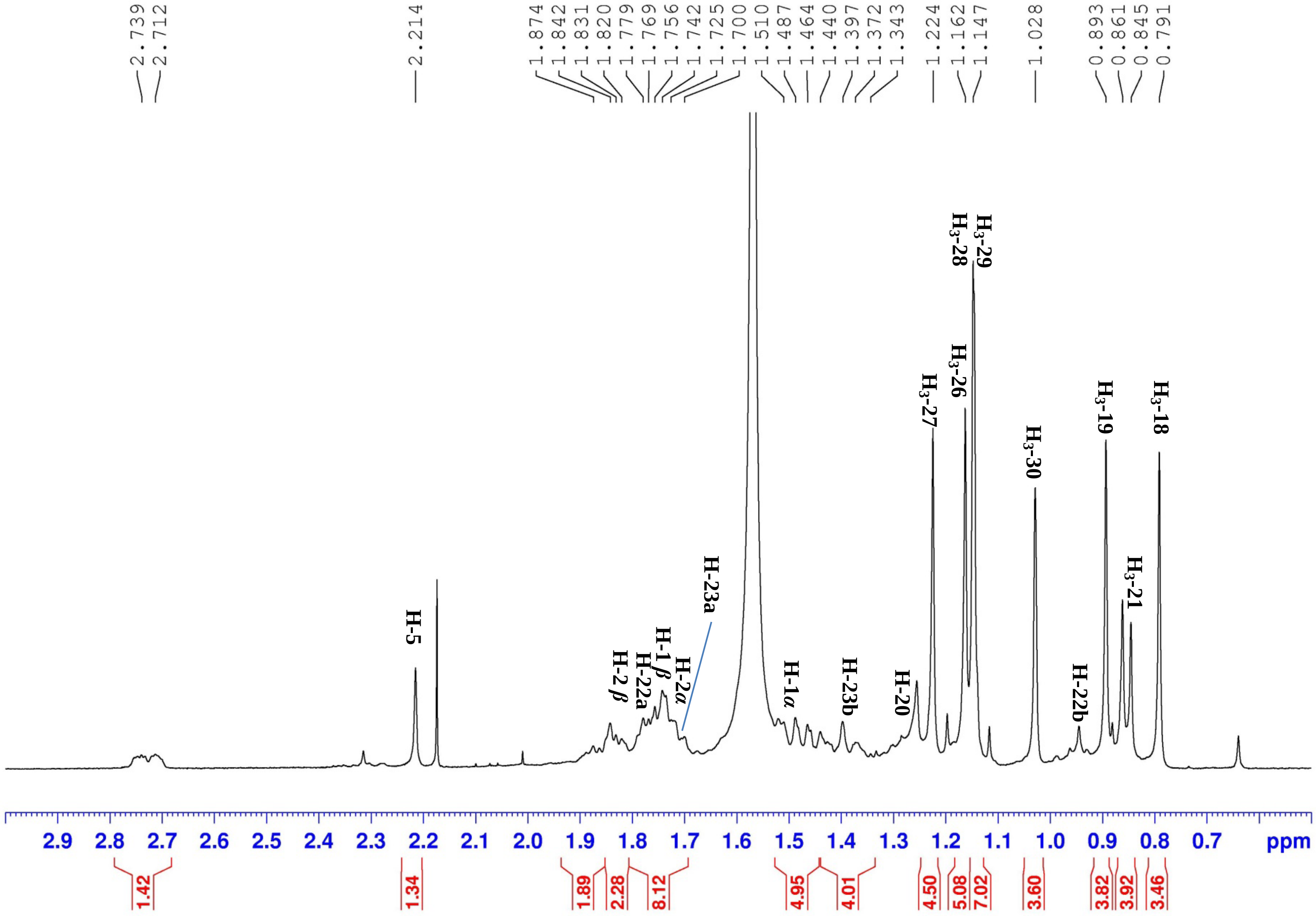

Supplement: Supplementary file 1 [file marinedrugs-16-00488-s001.zip › marinedrugs-394744-SI.pdf]
